# Genetic characterization of an almond germplasm collection and volatilome profiling of raw and roasted kernels

**DOI:** 10.1038/s41438-021-00465-7

**Published:** 2021-02-01

**Authors:** M. Di Guardo, B. Farneti, I. Khomenko, G. Modica, A. Mosca, G. Distefano, L. Bianco, M. Troggio, F. Sottile, S. La Malfa, F. Biasioli, A. Gentile

**Affiliations:** 1 Department of Agriculture, Food and Environment (Di3A), University of Catania, via Valdisavoia 5, 95123 Catania, Italy; 2 Research and Innovation Centre, Fondazione Edmund Mach, San Michele all’ Adige, Trento, Italy; 3 Dipartimento di Architettura, University of Palermo, Viale delle Scienze, Ed. 14 90128, Palermo, Italy; 4 National Center for Citrus Improvement, College of Horticulture and Landscape, Hunan Agricultural University, Changsha, China

**Keywords:** Plant breeding, Plant physiology

## Abstract

Almond is appreciated for its nutraceutical value and for the aromatic profile of the kernels. In this work, an almond collection composed of 96 Sicilian accessions complemented with 10 widely cultivated cultivars was phenotyped for the production of volatile organic compounds using a proton-transfer time-of-flight mass spectrometer and genotyped using the Illumina Infinium^®^18 K Peach SNP array. The profiling of the aroma was carried out on fresh and roasted kernels enabling the detection of 150 mass peaks. Sixty eight, for the most related with sulfur compounds, furan containing compounds, and aldehydes formed by Strecker degradation, significantly increased during roasting, while the concentration of fifty-four mass peaks, for the most belonging to alcohols and terpenes, significantly decreased. Four hundred and seventy-one robust SNPs were selected and employed for population genetic studies. Structure analysis detected three subpopulations with the Sicilian accessions characterized by a different genetic stratification compared to those collected in Apulia (South Italy) and the International cultivars. The linkage-disequilibrium (LD) decay across the genome was equal to *r*^2^ = 0.083. Furthermore, a high level of collinearity (*r*^2^ = 0.96) between almond and peach was registered confirming the high synteny between the two genomes. A preliminary application of a genome-wide association analysis allowed the detection of significant marker-trait associations for 31 fresh and 33 roasted almond mass peaks respectively. An accurate genetic and phenotypic characterization of novel germplasm can represent a valuable tool for the set-up of marker-assisted selection of novel cultivars with an enhanced aromatic profile.

## Introduction

Almond (*Prunus dulcis* Mill. D.A. Webb; syn. *Prunus amygdalus* Batsch.; *Amygdalus communis* L.; *Amygdalus dulcis* Mill.), belongs to the genus *Prunus*, family *Rosaceae* a taxonomic group that includes numerous species of agronomical interest such as: apple, pear, peach, apricot, cherry, prune, and several berry fruits. Among tree nuts, almond ranks third in worldwide production behind cashew and walnut, with the US being the largest producer^1^. In ancient times, its cultivation rapidly spread throughout the Mediterranean regions from central Asia reaching Sicily during the Greek domination^2^. Nowadays, almond is widely cultivated all over the Mediterranean basin.

Almond cultivation relies mainly on a few highly productive cultivars. However, almond germplasm is composed by thousands of accessions showing wide variability in terms of adaptation to different pedoclimatic conditions, resistance to biotic and abiotic stress and kernel quality traits^3–5^. The self-incompatibility of most of the almond cultivars, together with the extensive use of seeds for propagation, played an important role in the differentiation of such massive genetic diversity within the almond species^6,7^.

One of the leading aspects guiding the choice of the almond cultivar is the kernel quality. Such multi-factorial trait encompasses the physical appearance (colour, texture, size), its nutritional properties and the flavour (aroma and taste)^3^. Almonds are particularly valued for their sensory, nutritional, and health attributes^4^ and kernels are often consumed fresh or as ingredients in processed foods^5^. Considering their wide use for fresh consumption or for confectionery, the flavour of both raw and roasted almond kernels greatly influences their economic value. While taste is determined primarily by non-volatile metabolites (sugars, organic acids, amino acids) and it is perceived in the mouth, aroma is the result of the interplay of a large array of volatile organic compounds (VOCs) and it is perceived largely by the olfactory receptors. In light of this, aroma profiles of raw and roasted almond have been dissected through several approaches at harvest and during storage. VOC profile of raw almonds is composed for the most by aldehydes such as hexanal, nonanal and benzaldehyde^6–9^, although several ketones, alcohols, alkanes and heterocyclic compounds have been reported^10^. Pyrazines, pyrroles, furans and aldehydes comprised the main volatile compound classes in roasted almonds^10^. The chemical reactions behind the formation of the majority of VOCs in roasted almonds are the Maillard reactions^11^ which produce branched-chain aldehydes, alcohols, sulfur-containing and heterocyclic compounds, while straight chain volatiles reflect heat-induced oxidation during roasting^8^. Several sulfur-containing aroma compounds are de novo produced during roasting by the degradation of sulfur-containing amino-acids, such as dimethyltrisulfide and 2-furfurylthiol, respectively formed by methionine and cysteine^9,12^.

Since aroma involves the perception of a plethora of VOCs, their assessment is crucial to guarantee the selection and marketability of high-quality fruit. Thus, high priority should be given to replace poor flavour cultivars with favourable ones, exploiting the variability already available in nature. However, the analysis of the aroma trait in many samples, necessary to overcome the significant biological and genetic variability among samples, may be laborious and time consuming. VOC phenotyping is currently a limiting step in breeding programs, due to high costs and complex analytical techniques. Another limitation also raised by the elevated, and difficult to be controlled, the interaction between fruit genetics, environmental effects, and product transformation. Even though different cultivars are often characterized by substantial variations in flavour^8^, most plant breeding programs have historically neglected this trait, given its intrinsic complexity and costs to phenotype^9,10^. To correct this inconsistency and incorporate flavour into breeding program routines, it is necessary to identify the sources of flavour variability, understand their genetic architecture, and define cost-effective methods of selection.

According to recent publications, direct injection mass spectrometry (DI-MS) techniques, like Proton Transfer Reaction -Time of Flight- Mass Spectrometry (PTR-ToF-MS), are powerful high-throughput phenotyping tool for both genetic and quality-related studies^11,12^. The rapidity and the moderate cost of DI-MS analysis may allow to perform a detailed aroma characterization with a peculiar attention to the VOC fold changes caused by ad hoc storage and transformation experiments. Indeed, this technique has been already applied for the VOC characterization of transformed products, such as fermented cocoa^13^ and coffee beans^14^, and for genetic association studies of different fruit species^12,15,16^.

The production of these VOCs is controlled by two classes of genes: those encoding enzymes responsible for the synthesis of the end products and those encoding factors regulating the biochemical pathways^9^.

A significant increase in the use of both high-throughput DNA-derived data and advanced phenotyping approaches to dissect the causative genes underlying traits of agronomical interest through marker-trait association approaches^17–19^ has happened in the last decades. To this extent several segregating populations were developed to build the first genetic maps of almond^20–22^. Such studies laid the foundations for QTL analysis approaches to detect genomic regions linked to phytosterol content^21,23^ and other traits related to the physical traits of almond nut and kernel^22^. However, none of these genetic association studies was focused on understanding the genetic aroma regulation of almond kernels.

The high genetic similarity between peach and almond^19^ allowed the development of interspecific almond x peach segregating population and their use for QTL analyses for traits of agronomical interest such as chilling and heat requirement^24^, brown rot resistance^25^ and ‘stone-adhesion/flesh-texture’^26^. The availability of high-throughput genotyping platforms enabled the use of genome-wide association study (GWAS) approaches on germplasm collections composed by unrelated individuals^27^. GWAS approaches proved its efficiency in almond^28^ as well as in many other outcrossing tree crops, since they are capable of assessing higher allelic variability and smaller linkage blocks compared to other methods.

In light of this, the set-up of an ex situ germplasm collection is a strategic step both for conservation and breeding purposes. The present work is based on the analysis of an ex situ germplasm collection that was already characterized both phenotypically and genetically highlighting a variability both within Sicilian accessions and between those and the Italian and international elite cultivars^29,30^. Overall, such almond germplasm collection encompasses accessions showing both high resistance/tolerance to biotic and abiotic stresses and/or quality traits of interest. Such a genetic reservoir could play a fundamental role in future breeding plans in which specific traits characterizing local selections could be introgressed into elite cultivars through marker-assisted breeding selection approaches.

In this survey, our almond collection was both phenotyped using a proton-transfer time-of-flight mass spectrometer and genotyped using the Illumina Infinium^®^18 K Peach SNP array developed by RosBREED consortium^31^. Genetic data were employed for synteny analysis and to decipher both the genetic stratification and the linkage disequilibrium (LD) extent within the collection in the analysis. The same germplasm was phenotyped for the production of VOCs both on raw and roasted kernels using a PTR-ToF-MS.

The aims of this work were (i) to estimate the volatilome variability among almond different genotypes; (ii) to evaluate the effect of roasting on the VOC composition of the almond kernel; (iii) to identify the best performing accessions to be used as superior parental lines for future breeding programs; (iv) to detect molecular markers linked to VOCs of interest. In addition, the results of this study might be useful in defining an objective VOC phenotyping protocol to apply in all production stages, from breeding to the food industry transformation. This study is a first, preliminary, step toward the definition of molecular markers that can be readily employed for marker-assisted selection (MAS) approaches and provide novel insights on the genetic mechanisms regulating the VOCs profile in almond.

## Results and discussion

### High-resolution VOC phenotyping

Almond VOC profile was assessed on raw and roasted kernels in triplicate by PTR-ToF-MS analysis as described in Farneti et al.^11^. VOC mass peaks from the raw PTR-ToF-MS spectra were reduced from 422 to 150, applying noise and correlation coefficient thresholds (Table 1, Supplementary Fig. 1, Supplementary Tables 1–2). Tentative identification (t.i.) of each mass peak, detected by PTR-ToF-MS, was based on in-house library of pure standards and on literature review^32–36^. VOC profile was considerably altered during roasting, as 122 mass peaks significantly differed between raw and roasted almonds (Table 1, Supplementary Fig. 1, Supplementary Tables 1–2). To our knowledge, this is the first work about PTR-MS application on almond kernels; this technique has already been successfully applied for the characterization of fermented cocoa and green and roasted coffee beans^13,14^ and for the online monitoring of coffee roasting^37–39^.

**Table 1:** Volatile organic compounds detected by proton transfer reaction time of flight mass spectrometer (PTR-ToF-MS) on fresh and roasted almond kernels, over 106 *Prunus dulcis* accessions

*m/z*	Formula	Tentative identification	Raw				Roasted				*P* value	Variation
			mean	std	min	max	mean	std	min	max		
26.016	C2H2+	Common fragment	6.6	4.3	1.4	32.3	7.2	5.0	1.5	34.7	*	↗
28.019	C2H4+	Common fragment	3.8	19.5	0.3	229.2	0.0	0.0	0.0	0.0	***	▢
31.018	CH2OH+		33.7	17.5	4.0	210.0	112.5	39.0	24.0	345.9	***	↗
33.033	CH4OH+	Methanol	1626.1	841.5	91.0	8627.8	5057.5	1832.2	842.9	15728.8	***	▢
34.996	H2SH+	Hydrogen sulfide	0.0	0.0	0.0	0.0	0.1	1.1	0.0	21.4	*	↗
39.023	C3H3+	Common fragment	118.3	72.8	33.9	810.5	118.3	108.2	30.2	1322.3	NS	−
41.039	C3H5+	Common fragment	60.4	34.3	17.4	334.0	45.5	37.8	11.0	408.7	***	▢
42.012			2.4	1.6	0.0	13.7	4.1	2.3	0.0	18.1	***	↗
42.022			12.3	7.5	1.4	107.3	35.5	12.0	8.0	112.9	***	↗
43.018	C2H3O+	Common fragment	220.6	111.8	42.6	758.1	257.9	195.6	40.5	2232.2	**	↗
43.030	CH2N2H+	Cyanamide	32.5	15.7	4.8	162.7	87.0	29.6	0.0	231.1	***	↗
43.055	C3H7+	Common fragment	36.8	18.4	13.2	177.8	21.2	15.0	5.4	103.4	***	▢
44.025			9.5	4.5	2.9	31.8	9.0	5.2	1.7	44.9	NS	−
45.033	C2H4OH+	Acetaldehyde	912.5	1012.1	121.8	9916.9	1066.6	954.7	152.2	9763.6	*	↗
47.049	C2H6OH+	Ethanol	457.8	453.9	23.0	3352.0	316.7	362.3	16.2	2623.1	***	▢
49.011	CH4SH+	Methanethiol	0.3	0.4	0.1	6.4	18.1	16.4	0.7	141.0	***	↗
53.004			2.3	1.3	0.6	14.6	2.6	2.0	0.6	19.2	*	↗
53.040	C4H5+	Common fragment	1.4	2.8	0.3	36.6	3.3	4.3	0.4	41.6	***	▢
53.048			1.9	1.1	0.0	10.3	4.1	2.1	0.0	13.9	***	↗
55.054	C4H7+	Butanal, common fragment	13.2	14.7	3.9	210.9	20.3	34.4	0.4	354.4	***	↗
56.026			0.5	0.3	0.1	2.6	0.0	0.0	0.0	0.0	***	▢
57.035	C3H4OH+	Common fragment	14.9	7.4	4.4	68.4	8.8	6.5	2.2	46.9	***	▢
57.043	C2H4N2H+	Aminoacetonitrile	8.2	3.0	1.0	16.7	4.9	2.4	0.0	15.0	***	▢
57.070	C4H9+	1-Butanol	47.9	38.9	10.4	492.2	19.8	17.6	5.7	155.7	***	▢
59.049	C3H6OH+	Acetone	114.2	147.2	19.7	1455.2	289.7	279.1	48.0	1746.2	***	↗
61.028	C2H4O2H+	Acetic acid, fragment of esters	219.3	110.3	48.6	814.8	276.7	186.4	43.6	1297.5	***	↗
61.055	C3H8OH+		3.2	2.2	1.1	35.8	6.6	13.9	1.0	172.2	***	↗
63.012	CO2H3O+	Water cluster of carbon dioxide	0.7	0.5	0.0	6.3	0.8	0.4	0.0	3.6	***	↗
63.029	C2H6SH+	Dimethyl sulfide	2.2	4.3	0.0	71.4	15.4	12.0	0.0	145.5	***	↗
63.043	C2H6O2H+	Water cluster of acetaldehyde	116.0	177.3	3.6	1634.1	81.7	105.2	1.7	1036.0	**	↗
65.944			0.0	0.0	0.0	0.2	0.0	0.0	0.0	0.2	***	▢
67.032	C3H2N2H+	Propanedinitrile	0.1	0.0	0.0	0.7	0.1	0.1	0.0	0.6	***	↗
67.057	C5H7+	Pentanal, common fragment	1.6	1.2	0.4	7.4	2.5	1.9	0.6	20.4	***	↗
67.992			0.0	0.0	0.0	0.1	0.0	0.0	0.0	0.2	***	↗
69.003			0.0	0.0	0.0	0.0	0.1	0.1	0.0	1.0	***	↗
69.033	C4H4OH+	Furan	0.8	0.2	0.2	1.9	2.8	3.1	0.3	34.0	***	↗
69.056			11.8	6.7	0.5	52.6	22.6	12.9	1.6	117.0	***	↗
69.071	C5H9+	Isoprene, common fragment	5.7	3.1	1.5	18.3	22.0	40.1	0.4	549.3	***	↗
71.051	C4H6OH+	Butenal	1.2	0.5	0.6	5.8	3.8	4.8	0.8	62.9	***	↗
71.086	C5H11+	2-Pentanol, 2-Methyl-1-butanol+3-Methyl-1-butanol, Pentanol	29.2	21.9	5.2	188.4	14.7	12.3	3.2	106.5	***	▢
72.962			0.0	0.0	0.0	0.0	0.1	0.2	0.0	1.1	***	↗
73.029	C3H4O2H+	Propiolactone, Propenoic acid	1.4	0.3	0.7	2.3	2.0	1.0	0.1	6.7	***	↗
73.051			13.1	5.9	0.7	33.4	10.1	10.5	0.0	121.3	***	▢
73.064	C4H8OH+	2-Methyl-Propanal	19.2	13.5	5.5	127.1	141.8	220.7	8.3	1530.9	***	↗
75.035			0.4	0.1	0.2	0.7	2.6	1.2	0.0	8.4	***	↗
75.045	C3H6O2H+	1-Hydroxy-2-Propanone	8.0	4.3	2.4	26.9	15.8	10.7	2.8	76.4	***	↗
75.072			0.6	0.4	0.1	3.9	0.9	0.9	0.1	6.6	***	↗
76.954	CS2H+	Carbon disulfide	0.1	0.1	0.0	0.5	0.0	0.0	0.0	0.3	***	▢
77.008			0.0	0.0	0.0	0.0	0.1	0.1	0.0	0.6	***	↗
77.037			0.2	0.2	0.1	2.4	0.4	0.6	0.0	5.3	***	↗
79.040	C2H6O3H+	Cluster of ms61.028	34.5	16.8	5.0	109.5	34.6	24.0	3.8	161.3	NS	−
79.060	C6H7+	Benzene, aromatic ring fragment	4.2	5.8	0.7	67.2	5.7	40.8	0.0	613.2	NS	−
79.078			3.1	2.7	0.5	22.2	2.5	3.5	0.0	30.6	*	▢
80.060			0.3	0.8	0.0	10.5	0.3	0.9	0.0	7.9	NS	−
81.041	C4H4N2H+	Pyrazine	1.1	0.7	0.2	8.4	1.1	0.9	0.2	8.1	NS	−
81.070	C6H9+		2.6	1.7	0.9	13.1	1.2	0.9	0.4	9.7	***	▢
83.051	C5H6OH+	Methylfuran	3.4	1.8	0.6	17.5	4.1	1.9	0.8	16.6	***	↗
83.076			7.2	7.4	0.5	61.2	2.8	4.3	0.0	30.7	***	▢
83.087	C6H11+	Hexenol, Hexanal	1.7	1.9	0.0	15.5	9.3	34.5	0.0	466.1	***	↗
84.087			0.7	2.3	0.1	27.6	0.0	0.0	0.0	0.0	***	▢
85.030			0.0	0.0	0.0	0.0	0.6	0.3	0.3	3.4	***	↗
85.067	C5H8OH+	Pentanal, Pentanone	1.0	0.8	0.4	8.3	2.4	1.7	0.5	12.3	***	↗
85.102	C6H13+	Hexanol	13.1	20.5	0.5	220.2	5.2	11.2	0.2	128.2	***	▢
86.009			0.0	0.0	0.0	0.0	0.2	0.1	0.0	1.6	***	↗
87.045	C4H6O2H+	γ-Butyrolactone	5.4	2.9	1.7	23.1	13.4	12.0	2.7	114.6	***	↗
87.081	C5H10OH+	2-Methyl Butanal, 3-Methyl Butanal, 2-Pentanone	4.8	14.7	0.9	268.5	29.5	47.7	1.9	566.8	***	↗
89.061	C4H8O2H+	Ethyl acetate	3.4	2.7	1.0	23.6	6.7	13.4	0.8	164.3	***	↗
91.057	C4H10SH+	Diethyl sulfide	2.4	3.8	0.6	41.8	3.2	7.0	0.6	67.2	*	↗
91.075	C4H10O2H+	Butanediol	1.2	0.9	0.2	8.5	4.7	7.5	0.0	58.0	***	↗
93.040			0.0	0.0	0.0	0.0	6.5	1.8	3.2	14.2	***	↗
93.073	C7H9+	Toluene	2.4	1.2	0.9	9.3	1.5	0.9	0.4	4.9	***	▢
93.091			4.4	9.2	0.2	110.1	1.6	5.2	0.0	56.9	***	▢
95.051	C6H6OH+	Phenol	1.8	1.4	1.1	18.8	2.4	5.1	1.1	47.7	*	↗
95.088	C7H11+	Heptenal	0.7	0.6	0.3	8.5	0.5	0.5	0.0	5.9	***	▢
97.048	C5H4O2H+	Furfural	0.7	0.3	0.2	1.8	2.7	5.9	0.4	61.6	***	↗
97.066	C6H8OH+	Ethylfuran	0.3	0.1	0.2	0.7	1.0	0.5	0.3	4.6	***	↗
97.102	C7H13+	Heptanal	0.5	0.7	0.2	8.0	0.3	0.6	0.1	7.9	***	▢
99.046	C5H6O2H+	2-Furan methanol	0.5	0.1	0.2	0.9	0.7	0.5	0.3	5.7	***	↗
99.082	C6H10OH+	Hexenal	0.4	0.2	0.2	1.5	0.6	0.3	0.3	4.6	***	↗
99.117	C7H15+	Heptanol	0.7	1.3	0.0	23.6	0.2	0.5	0.0	9.2	***	▢
99.951			0.0	0.0	0.0	0.0	0.0	0.0	0.0	0.1	***	↗
101.062	C5H8O2H+	2,3-Pentanedione	1.4	0.6	0.5	4.9	2.1	1.5	0.6	12.6	***	↗
101.097	C6H12OH+	Hexanal	2.5	10.6	0.2	131.8	3.4	11.3	0.2	143.5	NS	−
103.052			0.2	0.1	0.1	1.0	0.6	0.7	0.1	6.4	***	↗
103.077	C5H10O2H+	C5 esters and acids	0.6	0.3	0.2	4.3	0.6	0.4	0.2	3.6	NS	−
103.115			0.1	0.2	0.0	2.9	0.0	0.1	0.0	1.2	***	▢
105.039	C4H8OSH+	Methional	0.4	0.3	0.0	2.4	0.6	1.9	0.1	16.1	**	↗
105.068	C8H9+	Phenyl ethyl alcohol	2.7	4.7	0.4	44.0	1.5	1.9	0.0	16.9	***	▢
105.090	C5H12O2H+		0.0	0.0	0.0	0.0	2.4	3.6	0.1	40.2	***	↗
107.044	C7H6OH+	Benzaldehyde	15.4	70.0	0.3	955.3	13.5	93.4	0.3	1119.1	NS	−
107.089	C8H11+	Ethyl benzene, p-Xylene, m-Xylene, o-Xylene	2.3	4.4	0.5	52.9	1.5	6.9	0.0	118.0	NS	−
109.065	C7H8OH+	Benzyl alcohol, Cresol	0.3	0.3	0.1	3.2	0.4	0.9	0.1	7.5	**	↗
109.103	C8H13+	Octenal	0.5	0.4	0.2	4.9	0.5	0.7	0.0	8.4	NS	−
111.047	C6H6O2H+		2.3	0.6	0.5	3.8	1.8	0.9	0.5	9.1	***	▢
111.084			0.3	0.1	0.2	0.7	0.5	0.3	0.1	2.1	***	↗
111.118	C8H15+	1-Octen-3-ol	0.5	0.8	0.1	11.2	0.3	0.4	0.1	6.9	***	▢
113.064	C6H8O2H+	2(5H)-Furanone, 5,5-dimethyl-	0.3	0.1	0.2	0.5	0.2	0.1	0.1	0.6	***	▢
113.099	C7H12OH+	Heptanal, Heptanone	0.1	0.1	0.1	1.7	0.3	0.5	0.1	5.5	***	↗
113.133	C8H17+	2-Ethyl-1-Hexanol, Octanol	1.0	0.5	0.1	2.4	0.2	0.1	0.0	0.9	***	▢
115.078	C6H10O2H+	Caprolactone	0.5	0.7	0.2	12.5	0.5	0.5	0.2	8.2	NS	−
115.114	C7H14OH+	2-Heptanal (E o Z)	0.6	2.4	0.1	38.8	0.4	1.3	0.1	19.2	NS	−
117.062	C9H9+		0.2	0.1	0.0	0.5	0.2	0.1	0.1	0.5	***	▢
117.092	C6H12O2H+	Butanoic acid ethyl ester, butyl acetate, Hexanoic acid	0.5	1.0	0.1	18.5	0.6	0.7	0.1	12.1	NS	−
117.958			0.0	0.0	0.0	0.0	0.0	0.0	0.0	0.4	***	↗
119.106	C6H14O2H+		0.5	2.4	0.0	29.4	0.7	1.9	0.0	23.7	NS	−
121.067	C8H8OH+	Benzeneacetaldehyde	0.4	0.3	0.2	4.8	1.9	1.9	0.3	23.9	***	↗
121.104	C9H13+		0.2	0.1	0.1	1.1	0.1	0.1	0.0	0.4	***	▢
121.120			0.2	0.4	0.0	4.4	0.1	0.1	0.0	1.1	***	▢
123.048			0.2	0.0	0.1	0.3	0.2	0.1	0.1	0.4	***	▢
123.118	C9H15+	Nonenal	0.1	0.1	0.1	0.7	0.1	0.1	0.1	0.7	*	▢
125.059	C7H8O2H+	Benzyl alcohol	0.6	0.9	0.1	7.0	0.4	1.3	0.1	9.6	NS	−
125.100	C8H12OH+	Octadienone	0.2	0.2	0.0	1.9	0.2	0.3	0.0	2.6	NS	−
125.134	C9H17+	Nonanal, Nonenol	0.3	0.2	0.1	2.4	0.1	0.1	0.0	1.4	***	▢
127.042	C6H6O3H+	Maltol	0.0	0.0	0.0	0.1	0.1	0.1	0.0	0.7	***	↗
127.075	C7H10O2H+		0.1	0.0	0.1	0.3	0.1	0.0	0.0	0.5	***	▢
127.114	C8H14OH+	6-Methyl-5-Hepten-2-one	0.2	0.3	0.1	5.5	0.3	0.6	0.0	8.3	NS	−
127.148	C9H19+	Nonanol	1.6	0.9	0.0	6.3	0.2	0.2	0.0	2.2	***	▢
129.056	C6H8O3H+	Furaneol	0.2	0.0	0.1	0.3	0.2	0.1	0.1	0.4	NS	−
129.094	C7H12O2H+		0.1	0.0	0.1	0.6	0.1	0.1	0.1	0.7	NS	−
129.129	C8H16OH+	Octanal	0.5	1.2	0.1	12.6	0.3	0.9	0.0	9.6	*	▢
131.108	C7H14O2H+	Isoamyl acetate	0.1	0.1	0.0	1.2	0.1	0.1	0.0	1.3	NS	
133.121	C7H16O2H+		0.1	0.1	0.0	1.9	0.1	0.2	0.0	1.7	NS	−
134.973			0.0	0.0	0.0	0.2	0.0	0.0	0.0	0.2	***	↗
135.120	C10H15+	Cymene	0.1	0.1	0.0	1.1	0.1	0.1	0.0	0.9	***	▢
136.993			0.1	0.1	0.0	0.5	0.1	0.1	0.0	0.4	***	▢
137.134	C10H17+	Limonene	0.9	0.7	0.2	5.3	0.3	0.2	0.1	1.7	***	▢
139.114	C9H14OH+	2-Pentyl Furan	0.3	0.5	0.1	5.2	0.5	1.2	0.0	12.2	***	↗
141.130	C9H16OH+	Nonenal, Nonenone	0.1	0.1	0.0	0.6	0.1	0.1	0.0	0.9	NS	−
143.110	C8H14O2H+		0.2	0.2	0.1	3.8	0.2	0.2	0.1	2.5	NS	−
143.145	C9H18OH+	Nonanal	0.8	1.1	0.1	18.0	0.4	0.6	0.1	8.0	***	▢
145.123	C8H16O2H+	Hexyl acetate	0.1	0.1	0.0	0.7	0.1	0.1	0.0	0.9	**	↗
147.137	C8H18O2H+		0.0	0.1	0.0	1.4	0.0	0.1	0.0	0.8	NS	−
149.059	C9H8O2H+	Cinnamic acid	0.0	0.0	0.0	0.0	0.1	0.1	0.0	1.1	***	↗
149.119	C7H16O3H+		0.1	0.1	0.0	1.3	0.0	0.1	0.0	0.8	***	▢
153.092	C9H12O2H+		0.0	0.0	0.0	0.0	0.0	0.0	0.0	0.3	***	↗
153.131	C10H16OH+		0.1	0.0	0.0	0.3	0.0	0.0	0.0	0.3	***	▢
155.003			0.0	0.0	0.0	0.2	0.0	0.0	0.0	0.1	***	▢
155.178	C11H23+	Undecanol	0.6	0.3	0.0	1.6	0.1	0.1	0.0	0.6	***	▢
157.123	C9H16O2H+	Whiskey lactone	0.1	0.0	0.0	0.3	0.1	0.0	0.0	0.3	***	▢
157.161	C10H20OH+	Decanal	0.1	0.1	0.0	0.7	0.1	0.0	0.0	0.5	***	▢
159.139	C9H18O2H+	C9 esters and acids	0.1	0.3	0.0	5.7	0.1	0.1	0.0	1.6	NS	−
161.151	C9H20O2H+		0.1	0.1	0.0	1.2	0.0	0.0	0.0	0.4	***	▢
163.132	C8H18O3H+		0.0	0.0	0.0	0.2	0.0	0.0	0.0	0.1	**	▢
165.079			0.0	0.0	0.0	0.3	0.0	0.0	0.0	0.5	***	▢
167.056			0.2	0.1	0.1	1.2	0.2	0.2	0.1	2.1	***	▢
169.194	C12H25+	Dodecanol	0.4	0.2	0.0	1.2	0.1	0.0	0.0	0.2	***	▢
189.175	C11H24O2H+		0.0	0.0	0.0	0.1	0.0	0.0	0.0	0.0	***	▢
197.086			0.0	0.0	0.0	0.0	0.0	0.0	0.0	0.0	***	↗
205.197	C15H25+	Sesquiterpenes	0.1	0.3	0.0	4.8	0.0	0.2	0.0	3.2	NS	−
223.066			1.3	5.1	0.1	56.7	1.2	4.4	0.1	57.0	NS	−

Each VOC mass peak was tentatively identified based on an in-house library of pure standards and on literature review. VOC mass peak values were reported as concentration (ppb_v_). The average (*n* = 3), standard deviation, minimum and maximum values were reported

*p value < 0.05; **p value < 0.01; ***p value < 0.001; ↗ increase during roasting; ↘decrease during roasting

**Fig. 1:**
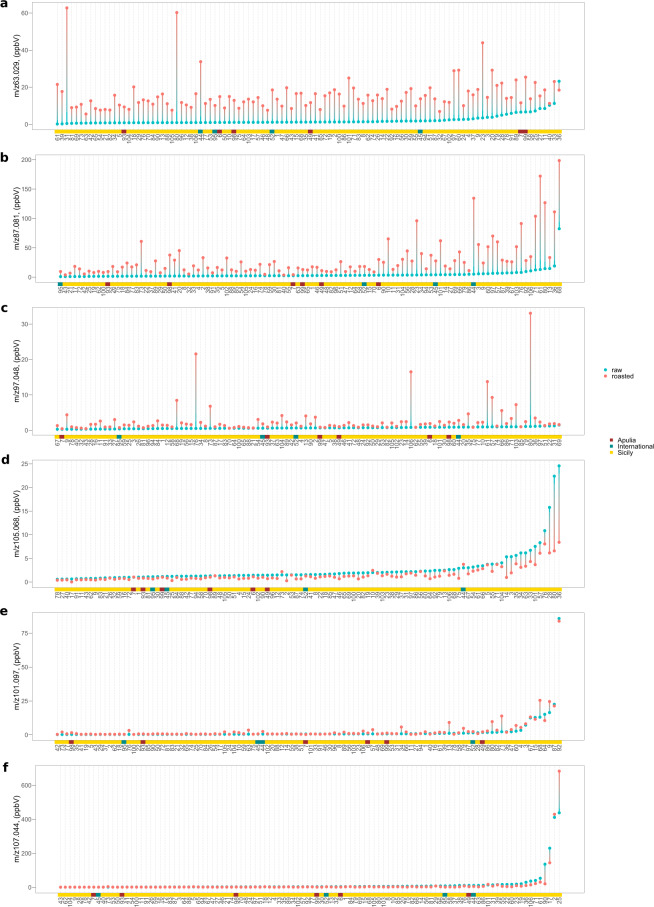
Lollipop graph of six VOC mass peaks characteristic of almond aroma profile (out of 150 detected in total by proton transfer reaction time of flight mass spectrometer, PTR-ToF-MS). **a** 63.029 (t.i. dimethyl sulfide), **b** 87.081 (t.i. 2- and 3-methylbutanal), **c** 97.048 (t.i. furfural), **d** 105.068 (t.i. phenyl ethyl alcohol), **e** 101.097 (t.i. hexanal), **f** 107.044 (t.i. benzaldehyde). Each graph illustrates the average value of three measurements recorded on fresh (blue) and roasted (pink) almond kernels. The corresponding complete names of the accessions were reported in supplementary table 1. In each graph, accessions were ordered based on the VOC mass peak concentration recorded on the raw kernel. The coloured line below the lollipop graph summarized the origin of each accession (red: Apulia, blue: International, yellow: Sicily). Lollipop graphs, together with violin plots and correlation plots of all 150 VOC mass peaks, are reported in Fig. S1

Among the 150 mass peaks, 68 significantly increased their content during roasting (Table 1, Fig. 1, Supplementary Fig. 1). Most of these masses were related with sulfur compounds, such as *m/z* 34.996 (t.i. hydrogen sulfide), *m/z* 49.011 (t.i. methanethiol), m/z 63.029 (t.i. dimethyl sulfide, Fig. 1a), *m/z* 91.057 (t.i. diethyl sulfide), and *m/z* 105.039 (t.i. methional), and with furan containing compounds produced by thermal degradation of sugars such as *m/z* 69.033 (t.i. furan), *m/z* 83.051 (t.i. methylfuran), *m/z* 97.048 (t.i. furfural, Fig. 1c), *m/z* 97.066 (t.i. ethylfuran), *m/z* 99.046 (t.i. 2-furan methanol), and *m/z* 139.114 (t.i. 2-pentyl furan). Moreover, roasting enhanced the concentration of aldehydes formed by Strecker degradation of valine, isoleucine, leucine and phenylalanine^40^, such as 2-methylpropanal (*m/z* 73.064), 2- and 3-methylbutanal (*m/z* 87.081, Fig. 1b), and benzeneacetaldehyde (*m/z* 121.066). Other relevant VOC mass peaks that significantly increased during roasting were methanol (*m/z* 33,033), cyanamide (*m/z* 43.03), acetic acid (*m/z* 61.028), 1-hydroxy-2-propanone (*m/z* 75.044), and γ-Butyrolactone (*m/z* 87,0453) (Table 1). Many of these compounds were found in roasted almonds and other nuts such as hazelnuts^41^, walnuts^42^, pecans^43^, peanuts^44^. Some similarities were also found with the aroma compound formation during coffee roasting which is well studied both by GC-MS and PTR-MS. However, the aroma profile of roasted coffee is usually richer in pyrroles, pyrazines, and other products of Maillard reaction, since coffee beans undergo the roasting for longer time and higher temperatures. These compounds were also found in almonds after a longer roasting time (data not shown).

On the contrary, fewer mass peaks (54 over 150) were significantly reduced during roasting (Table 1). Many of them were related with alcohol compounds, in particular ethanol (*m/z* 47.049), butanol (*m/z* 57.07), 2-pentanol (*m/z* 71.086), hexanol (*m/z* 85.10), phenyl ethyl alcohol (*m/z* 105.068, Figs. 1d), 1-octen-3-ol (*m/z* 111.118), and nonanol (*m/z* 127.148). Other relevant VOCs that significantly decreased during roasting were aminoacetonitrile (*m/z* 57.043) and limonene (*m/z* 137.13) (Table 1).

Only a few mass peaks (28 over 150) were not significantly modified by roasting. Among them, several compounds have an important role in the characterization of almond aroma^33,35^, such as benzaldehyde (*m/z* 107.043, Fig. 1f), benzene (*m/z* 79.04), ethyl benzene (*m/z* 107.088), pyrazine (*m/z* 81.041) and hexanal (*m/z* 101.097, Fig. 1e).

**Fig. 2:**
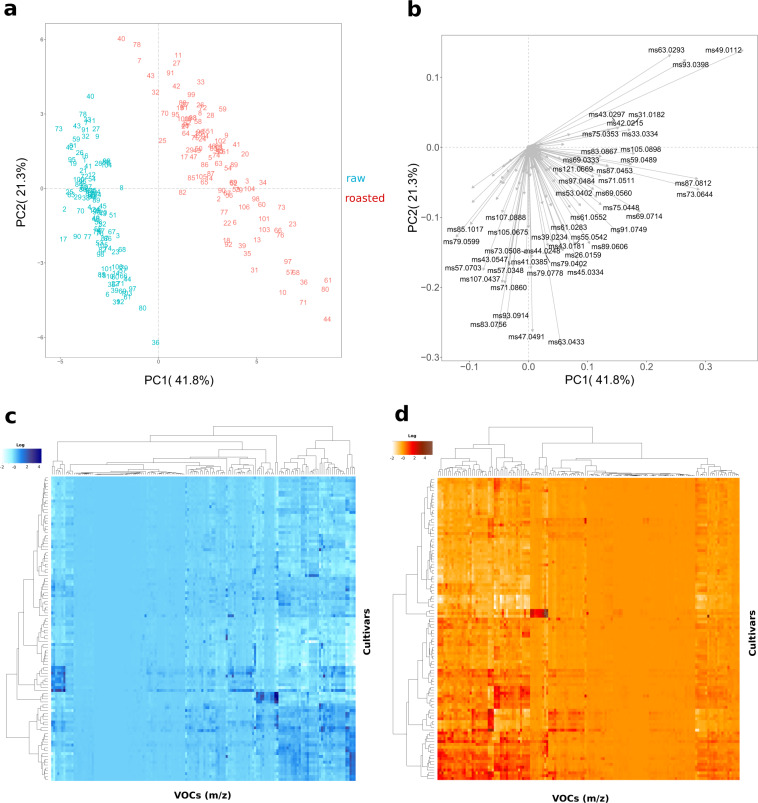
Almond VOCs assessment. Principal component analysis (PCA) (**a**), loading plot (**b**) and heatmaps with two-dimensional hierarchical dendrograms of the fresh (**c**) and roasted (**d**) VOC mass peaks detected in the 106 Prunus dulcis accessions assessed by proton transfer reaction time of flight mass spectrometer (PTR-ToF-MS). Each VOC concentration is the average of three biological replicates

The VOC variability, assessed on raw and roasted almonds, is graphically represented by the PCA plot (Fig. 2a, b) defined by the first two PCs (PC1: 41.8 % and PC2: 21.3% of the total phenotypic variability). VOC differences related to roasting were mostly explainable by PC1, while differences among almond genotypes, in particular for fresh kernels, were mostly related to PC2. Cultivars defined by negative values of PC2 had a more intense VOC profile for both fresh and roasted kernels, as it was also validated by the hierarchical clustering and heatmap (Fig. 2c, d). Almond VOC profile seemed to be mostly influenced by roasting, but still with significant interaction with genetic variability. As a result, fresh and roasted almond genotypes were significantly clustered into two groups (Fig. 2a) based on PC1 variability.

According to solely to the VOC profile assessed on fresh kernels, an accurate prediction of the profile after roasting is quite complex, since several compounds, like sulfur compounds, furans, and few aldehydes, are produced by the degradation of primary metabolites only during roasting (Fig. 1 and Supplementary figure 1). However, based on results of both PCA analysis (Fig. 2a, b) and hierarchical clustering (Fig. 2c, d), most of the accessions considered in this study maintained a comparable topological structure of the cluster’s tree (Supplementary Fig. 2). In particular, it was possible to identify two clusters of accessions composed respectively by “Angelica” (#3), “Baggiana” (#4), “Belvedere” (#9), “Cacciatura” (#15), “Montagna” (#64), “Mullisa Piccola” (#67), and “Sarbaggia di Sciascia” (#87); and by “Amara di Martorana” (#2), “Calamonaci” (#17) and “Cesaro 1” (#25) that maintained their VOC characteristics after roasting. These two clusters were characterized, respectively, by an elevated concentration of *m/z* 55.054 (t.i. butanal), *m/z* 83.086 (t.i. hexenol), *m/z* 85.102 (t.i. hexanol), *m/z* 101.097 (t.i. hexanal) and *m/z* 119.105; and of *m/z* 79.06 (t.i. benzene), *m/z* 91.057 (t.i. diethyl sulfide), *m/z* 105.039 (methional), *m/z* 107.044 (t.i. benzaldehyde), *m/z* 107.088 (t.i. ethyl benzene) and *m/z* 125.059 (t.i. benzyl alcohol).

Moreover, these volatilome results evidenced that all almond elite cultivars assessed in this study, except “Ferraduel” (#44), were characterized by a less intense VOC profile than many of the Sicilian accessions. As for many other horticulture products, this lower VOC content might be the indirect consequence of a cultivar selection for the most oriented to the fruit productivity rather than to the quality^9^. Noticeably, it was possible to define several clusters of cultivars, among the Sicilian accessions, characterized by a considerable high content of compounds with a specific, and easy to be perceived, aroma note, like benzaldehyde (Fig. 1f) or phenyl ethyl alcohol (Fig. 1d). While benzaldehyde is the characteristic and predominant odour compound of bitter almond^45^, phenyl ethyl alcohol, associated with floral and rose aroma note, was already detected in several almond genotypes, but at low concentrations^45,46^. “Don Pitrino” (#36), “Pizzuta grande” (#80), “Comunista” (#28), “Pilusedda” (#76), “Mennula du nigliu” (#57) and “Vaiana” (#101) were some of the cultivars of our germplasm collection characterized by highest phenyl ethyl alcohol concentration. This feature might be interesting not only for the agro-food sector but also for the cosmetic industry^47^.

Taking into account the high genetic variability considered in this study, we aimed to uncover most of the possible VOC variability among *Prunus dulcis* genotypes. However, without a detailed sensory analysis, quantifying the relevance of each VOC might be too speculative, bearing also in mind the non-linear interaction of these molecules in determining consumer preference. For this reason, in order to reduce any possible statistical bias in the result interpretation, all data were analysed with unsupervised multivariate statistical methodologies (PCA and hierarchical clustering). Nonetheless, considering each quality trait independently (i.e. Supplementary Fig. 1) might be useful for a breeding approach aimed to introduce, or improve, a distinct quality trait to an elite breeding line.

To simplify the application of these results, we limited the number of VOC traits that have to be considered (Supplementary Fig. 3), according to the loading plots of the principal component analysis and to the results of previously published articles on almond aroma^32,33,48–51^. The content of each trait (including also some pomological feature such as fruit and kernel weights, kernel thickness, or flavour) was grouped based on the distribution quantile (low: 0–25%; middle-low: 25–50%; middle-high: 50–75%; high: 75–100%), calculated for both raw and roasted assessment (Supplementary Fig. 3). Accessions employed in the study can be consequently sorted and clustered according to the content of the trait of interest, which can be arbitrarily chosen as implemented in the dedicated webpage https://iuliiakhomenko-fmach.shinyapps.io/QualySort/^52^.

### Definition of a robust SNP set and peach/almond synteny analysis

The original set of 16,038 SNPs was filtered using the ASSIsT software^53^ resulting in the detection of 471 (2.9%) robust polymorphic markers spanning the eight almond chromosomes. Among the discarded markers, 11,743 (73.2%) were monomorphic, 2321 (14.5%) failed in the amplification and the remaining 1503 (9.3%) were characterized by the presence of null alleles. The relatively low number of failed SNPs confirmed the high synteny between peach and almond genomes, nevertheless, the high fraction of monomorphic markers well reflected the fact that the probes were designed to target SNPs characterizing a different, although similar, species.

**Fig. 3:**
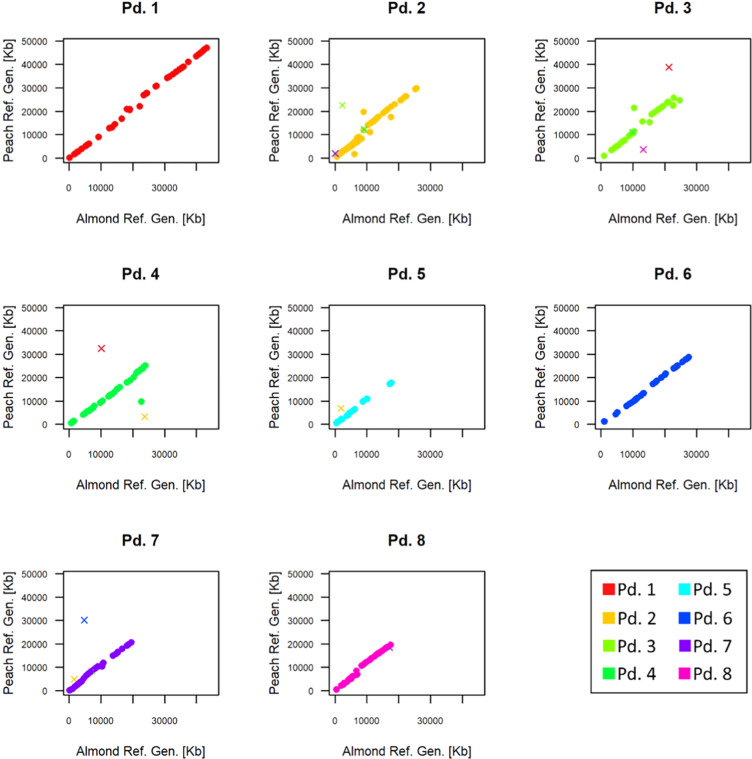
Collinearity plots between almond (*x* axis) and peach (*y* axis). Physical coordinates for SNP markers were retrieved from the Prunus dulcis Texas Genome v2.0 and the Prunus persica Whole-Genome Assembly v2.0 respectively. SNPs mapped in the same linkage groups in both species were represented as full dots while SNPs mapped in different linkage groups were represented as cross

SNPs spanned over 199.7 Mb, covering most of the almond genome which is characterized by a genome size ranging from 227 Mb (cultivar ‘Texas’)^54^ to 246 Mb (‘cultivar ‘Laurenne’)^55^. Pd1 was the longest linkage group (43.1 Mb) while the remaining ranged from 27.5 Mb (Pd6) to 17.5 Mb (Pd8) (Fig. 3, Supplementary Fig. 4). The number of mapped SNPs per chromosome spanned from 22 (Pd5) to 123 (Pd2), with a mean value of 59, the average marker density was 1 marker every 424 Kb (Supplementary Table 3).

The physical position of the 471 SNPs on the almond^54^ and peach genome^56^ was highly consistent (*r*^2^ = 0.96) highlighting a high synteny and collinearity between the two species. 13 SNPs (2.7%) mapped on different chromosomes in the two species. Pd1 and Pd6 did not show any inconsistencies and the SNP positions along the almond and peach linkage groups showed an *r*^2 ^= 0.997 (Pd1) and 0.998 (Pd6), (Fig. 3). The other linkage groups were characterized by the occurrence of 1–3 SNP(s) mapped in different linkage groups in the two species (Fig. 3). The high synteny between peach and almond was in agreement with previous studies highlighting that most of the genomes of the Prunus species can be considered as a single entity^19,54,57^.

### Analysis of genetic structure

The level of genetic stratification was assessed using the Bayesian approach implemented in the software STRUCTURE^58^. Among the different number of subpopulations postulated, *K* = 3 showed the highest likelihood (Δ*K* = 346) followed by *K* = 7 and *K* = 2 showing similar likelihoods (Δ*K* = 151 and 116 respectively, Supplementary Fig. 5).

**Fig. 4:**
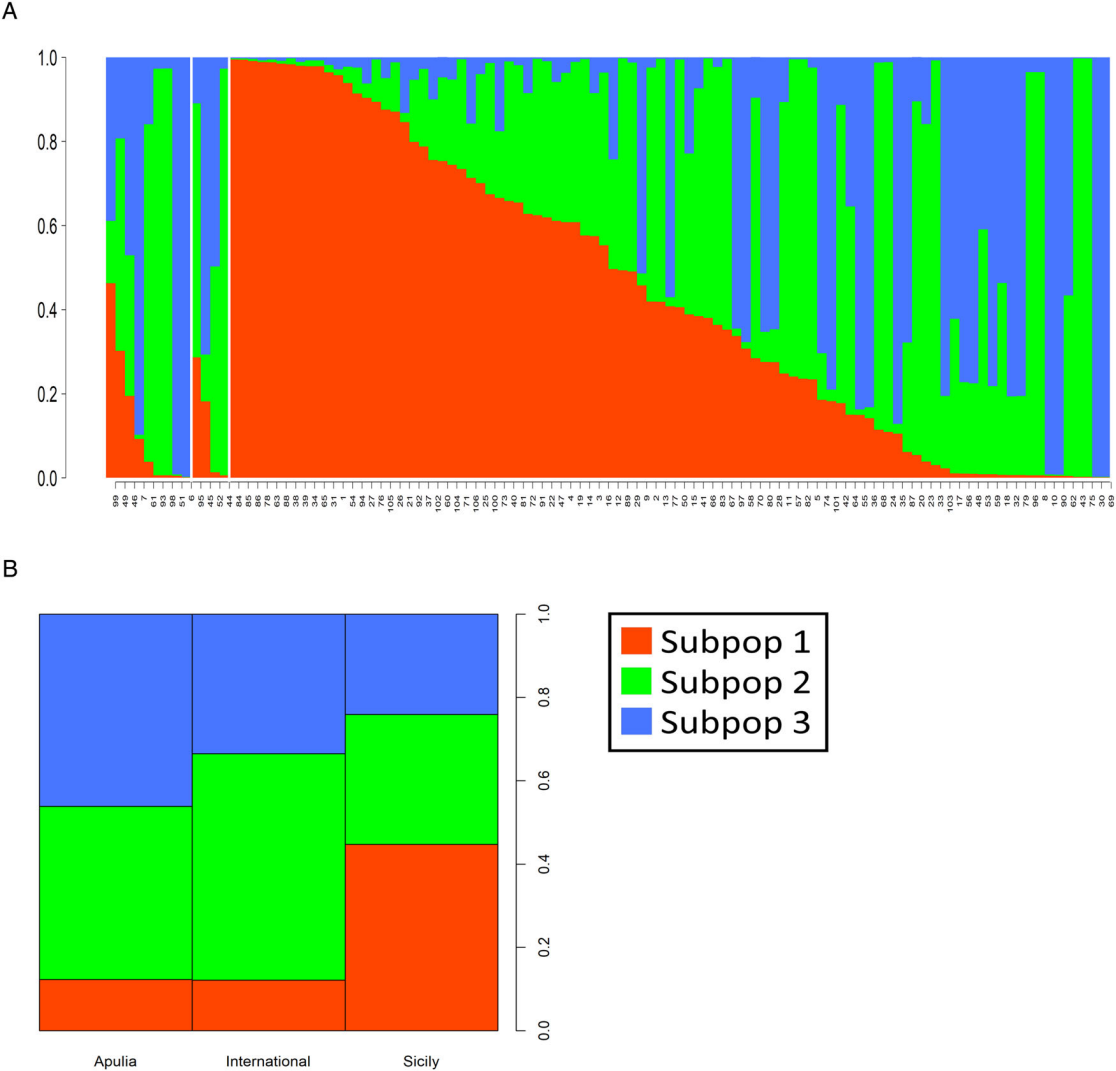
Structure plots for *K* = 3. The three subpopulations postulated were coloured in red (Subpop1), in green (Subpop2) and blue (Subpop3) as specified in the legend. **A** Individual results of the Structure analysis: the 106 accessions were ordered from left to right according to the different origin: Apulian (9), International (4), Sicilian (93), each group is separated by a white vertical line. **B** Contribution of the three subpopulations according to the different origin of the accessions

Figure 4A showed the genetic configuration of the 106 individuals for *K* = 3; 45 accessions were characterized by a clear predominance (Qi ≥ 0.8) of one of the three subpopulations, in particular: 19 accessions were predominantly characterized by Subpop1 while both Subpop2 and Subpop3 were represented by 13 accessions each. The remaining 61 genotypes showed a higher level of admixture (Supplementary Table 4). The SNP data analysis and the structure results confirmed the origin of the self-compatible cultivar “Supernova” (#93) as a mutant of the self-incompatible “Tuono” (#98)^59^ with the two cultivars characterized by an identical genotypic profile for all the SNP tested (and consequently an identical genetic structure for all the *K*s postulated, Supplementary Table 4, Supplementary Fig. 6). Overall, the Apulian and International accessions were characterized by a similar contribution of Subpop1 (12.3% and 12.1% respectively); then the most represented subpopulations were Subpop2 for the International group (54.3%) and Subpop3 (46.1%) for the Apulian accessions (Fig. 4b). Conversely, the Sicilian accessions were characterized by a much higher contribution of Subpop1 (44.8%) while Subpop2 and Subpop3 (31.1% and 24.1% respectively) were less represented compared to the Apulian and International accessions (Fig. 4b). The widely cultivated Sicilian accessions “Pizzuta d’Avola” (#78) and “Fascionello” (#39), both characterized by a high prevalence of Subpop1 (Qi = 0.99 and 0.98 respectively), were genetically distant from the Apulian and International cultivars (Fig. 4a, Supplementary Table 4), in agreement with previous genetic population studies based on SSRs^29^.

### Identification of genomic regions underlying VOCs production

Marker-trait association approaches were successfully employed in most of the tree crops to identify molecular markers in strong LD with the causative gene(s) influencing a trait of agronomical interest. In this study, molecular and phenotypic data were employed for a preliminary application of a GWAS analysis to identify molecular markers linked to the VOC production of the fresh and roasted almond kernel.

Among the 150 mass peaks related to the VOC profile of fresh almond, 31 were characterized by significant marker-trait associations for at least one of the SNP tested. Although with different relative frequencies, significant SNPs were detected in all linkage groups except Pd7. Pd8 resulted significantly associated with 21 VOC mass peaks while for the other linkage groups, the number of VOC mass peaks exceeding the significance threshold ranged from 1 (Pd1, Pd5 and Pd6) to 10 (Pd2) (Table 2). As for the VOCs contributing the roasted volatile profile of almonds, 33 mass peaks showed significant association (Table 3). Similarly, to what registered for the VOC profiling of fresh almonds, the highest number of signals were detected in Pd8 (21) while no significant associations were observed for Pd6 and Pd7 (Table 3).

**Table 2:** Table summarizing the GWAS analysis for the fresh aromatic compounds characterizing almond kernels

SNP	LG	Bp	adjusted *P* value	Mass peak (*m/z*)
Peach_AO_0868502	Pd 8	17442905	4.201470892	44.025
Peach_AO_0868502	Pd 8	17442905	4.071816888	45.033
SNP_IGA_269327	Pd 2	16486499	4.028810556	67.992
SNP_IGA_829830	Pd 8	5512121	4.157652959	73.064
SNP_IGA_574988	Pd 5	6207994	3.990959552	80.06
Peach_AO_0868502	Pd 8	17442905	4.075803665	81.041
SNP_IGA_269327	Pd 2	16486499	4.097908525	84.087
SNP_IGA_881173	Pd 8	17475606	5.503711552	
SNP_IGA_829830	Pd 8	5512121	4.14734958	85.067
SNP_IGA_269327	Pd 2	16486499	4.480595251	85.102
SNP_IGA_881173	Pd 8	17475606	4.988874658	
Peach_AO_0684073	Pd 6	26559511	4.288725249	87.081
Peach_AO_0868502	Pd 8	17442905	4.50974781	89.061
Peach_AO_0423401	Pd 4	4607767	4.416783526	97.066
SNP_IGA_881173	Pd 8	17475606	6.013452832	
Peach_AO_0423401	Pd 4	4607767	4.039290176	99.082
SNP_IGA_353861	Pd 3	18886145	6.774104184	99.117
SNP_IGA_269327	Pd 2	16486499	4.080811033	101.097
SNP_IGA_881173	Pd 8	17475606	5.125383663	
SNP_IGA_269327	Pd 2	16486499	4.033428856	103.115
SNP_IGA_881173	Pd 8	17475606	4.71831783	
SNP_IGA_269327	Pd 2	16486499	4.626074734	109.103
SNP_IGA_881173	Pd 8	17475606	8.525897285	111.118
SNP_IGA_881173	Pd 8	17475606	4.109750783	113.099
Peach_AO_0070614	Pd 1	22157470	4.988201194	115.114
SNP_IGA_269327	Pd 2	16486499	4.139158493	119.106
SNP_IGA_881173	Pd 8	17475606	5.081147321	
SNP_IGA_269327	Pd 2	16486499	4.101227804	121.12
SNP_IGA_881173	Pd 8	17475606	4.561356555	
SNP_IGA_269327	Pd 2	16486499	4.830909992	127.114
SNP_IGA_881173	Pd 8	17475606	5.85241464	
SNP_IGA_881173	Pd 8	17475606	5.650769836	129.129
Peach_AO_0868502	Pd 8	17442905	4.056555312	134.973
SNP_IGA_881173	Pd 8	17475606	5.979463513	143.11
SNP_IGA_353861	Pd 3	18886145	4.55573974	143.145
SNP_IGA_881173	Pd 8	17475606	6.358830393	147.137
SNP_IGA_881173	Pd 8	17475606	4.053380905	157.161
SNP_IGA_182843	Pd 2	5229709	4.034321274	159.139
SNP_IGA_353861	Pd 3	18886145	7.72892178	

For each SNP exceeding the GWAS significance threshold, the corresponding physical position according to the Prunus dulcis Texas Genome v2.0 was reported together with the relative FDR-adjusted *p* value (expressed as −log10 *p* value) and the corresponding volatile organic compounds (VOC) mass peak

**Table 3:** Table summarizing the GWAS analysis for the roasted aromatic compounds characterizing almond kernels

SNP	LG	Bp	adjusted *P* value	Mass peak (*m/z*)
Peach_AO_0260252	Pd 2	11738618	5.480565119	34.996
Peach_AO_0267535	Pd 2	12792357	5.445052149	
SNP_IGA_811258	Pd 3	13261568	5.549766949	43.018
SNP_IGA_811258	Pd 3	13261568	4.240584024	44.025
SNP_IGA_811258	Pd 3	13261568	4.523506694	61.028
Peach_AO_0280324	Pd 2	8937322	5.55589741	63.029
SNP_IGA_829830	Pd 8	5512121	4.241860664	73.064
SNP_IGA_829830	Pd 8	5512121	4.269403089	75.072
Peach_AO_0539745	Pd 5	1973367	4.2981529	83.076
SNP_IGA_881173	Pd 8	17475606	4.330316917	83.087
SNP_IGA_269327	Pd 2	16486499	4.152037221	85.102
SNP_IGA_881173	Pd 8	17475606	4.918056605	
SNP_IGA_829830	Pd 8	5512121	4.441318507	91.075
Peach_AO_0539745	Pd 5	1973367	4.206372997	93.091
SNP_IGA_881173	Pd 8	17475606	5.016508288	97.102
SNP_IGA_353861	Pd 3	18886145	7.040643097	99.117
SNP_IGA_881173	Pd 8	17475606	4.298774607	101.097
Peach_AO_0238372	Pd 2	10989084	4.31862644	103.052
SNP_IGA_829830	Pd 8	5512121	4.352859143	
SNP_IGA_881173	Pd 8	17475606	4.484961281	103.115
SNP_IGA_829830	Pd 8	5512121	4.288455871	105.09
SNP_IGA_574988	Pd 5	6207994	4.359176154	107.044
SNP_IGA_269327	Pd 2	16486499	4.511247633	109.103
SNP_IGA_881173	Pd 8	17475606	4.867016526	
Peach_AO_0047516	Pd 1	14445732	4.776994667	111.118
SNP_IGA_881173	Pd 8	17475606	5.582937641	
SNP_IGA_881173	Pd 8	17475606	4.671631163	113.099
Peach_AO_0070614	Pd 1	22157470	4.010715179	115.114
SNP_IGA_829830	Pd 8	5512121	4.172855296	121.067
SNP_IGA_563930	Pd 5	4387202	4.000740594	125.134
SNP_IGA_881173	Pd 8	17475606	5.591961603	
SNP_IGA_269327	Pd 2	16486499	4.139867267	127.114
SNP_IGA_881173	Pd 8	17475606	4.667857362	
Peach_AO_0814869	Pd 8	5093115	4.148932031	127.148
SNP_IGA_881173	Pd 8	17475606	5.254941658	129.129
SNP_IGA_881173	Pd 8	17475606	4.346475813	143.11
SNP_IGA_881173	Pd 8	17475606	4.205182451	147.137
Peach_AO_0423401	Pd 4	4607767	4.183997572	155.178
SNP_IGA_881173	Pd 8	17475606	4.404726319	157.161
SNP_IGA_353861	Pd 3	18886145	4.229546071	159.139

For each SNP exceeding the GWAS significance threshold, the corresponding physical position according to the Prunus dulcis Texas Genome v2.0 was reported together with the relative FDR-adjusted *p* value (expressed as −log10 *p* value) and the corresponding volatile organic compounds (VOC) mass peak

Among the VOC mass peaks showing a significant association, 15 were in common between fresh and roasted almond kernels (Tables 2 and 3). All those mass peaks were mapped in the same genetic regions in both VOC assessments except for m/z 44.025 (unknown molecule, mapped in Pd8 and in Pd3 respectively, Tables 2 and 3). Among the 15 VOCs detected in both fresh and roasted treatments, *m/z* 73.064, *m/z* 85.102, *m/z* 99.117, *m/z* 103.115, *m/z* 111.118, *m/z* 113.099, *m/z* 127.114, *m/z* 143.11 and *m/z* 157.161 showed significant differences between the two treatments (Table 1), suggesting that, even if the quantity of the VOC changed significantly during roasting, the genetic region associated to the trait remained the same.

**Fig. 5:**
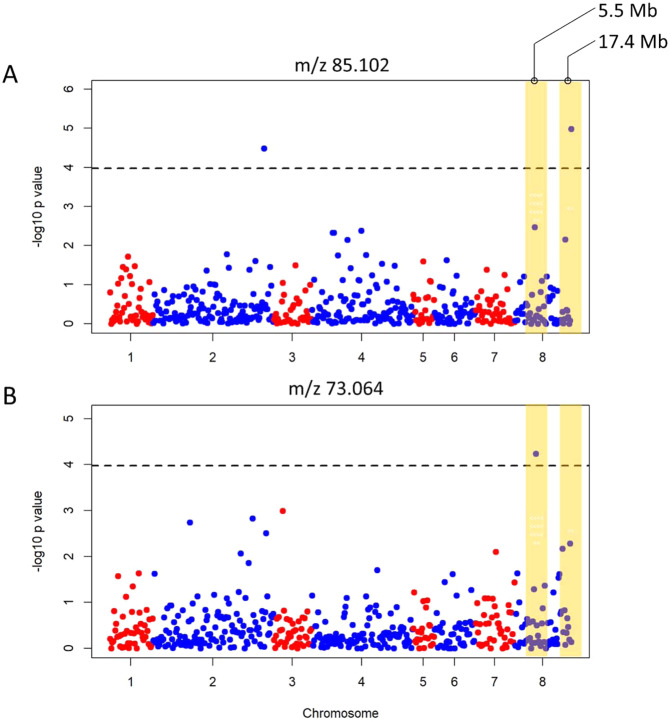
Manhattan plots illustrating the significant marker-trait association between genetic data and two mass peaks: *m/z* 85.102 (fresh assessment) and *m/z* 73.064 (roasted assessment). The two genomic regions in Pd8 in which the significant signals were detected were highlighted in yellow

In both fresh and roasted phenotypes, the significant signals in Pd8 were detected in two genetic regions: at around 5.5 Mb (2 and 7 SNPs respectively for fresh and roasted kernels) and 17.4 Mb (19 and 14 SNPs respectively) suggesting the existence of either a cluster of genes underlying the synthesis of different aromatic compounds or the presence of common genetic regulation systems (Fig. 5). Further study with higher marker density will help to clarify the number and function of the genes located in Pd8.).

### Analysis of LD

**Fig. 6:**
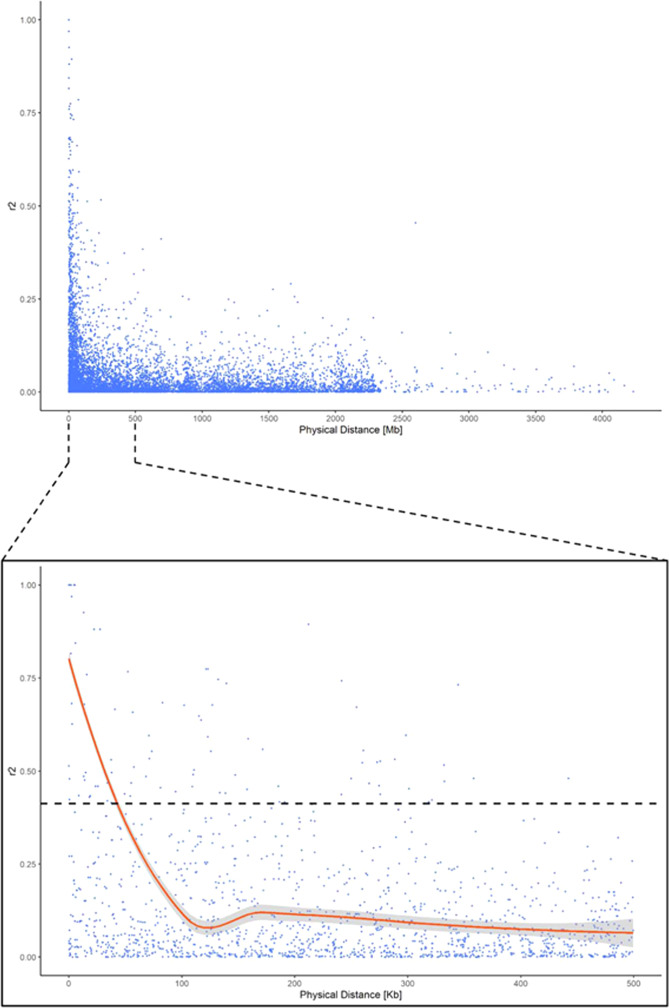
Genome-wide scatterplot of linkage disequilibrium decay (*r*^2^, *y* axis) against the genetic distance (Mb, *x* axis) for pairs of linked SNPs across the eight linkage groups. In the window below, only the first 500 Mb were displayed together with a LOESS fitting curve summarizing the linkage disequilibrium decay at increasing physical distances (red continuous line) and the relative confidence interval (grey area). The intersection between the LOESS fitting curve and the 95th percentile of the *r*^2^ distribution (black dashed line) was taken as the threshold value to consider two markers in close linkage disequilibrium

The analysis of the non-random association between loci through a whole-genome LD decay scan provides insights on the population genetic forces structuring the germplasm collection in the analysis. The mean *r*^2^ for all intrachromosomal loci pairs was equal to 0.083, while the chromosome-wise LD ranged from 0.076 (Pd2) to 0.181 (Pd5). The LD decay detected in this study was slightly higher than in previous reports (mean *r*^2^ = 0.04^23^). The highly significant *r*^2^ threshold, calculated as the 95th percentile of the *r*^2^ distribution, was 0.41, corresponding to LD blocks of ~60 Kb (Fig. 6). LD in almond decayed faster than peach^60–63^, apricot^64^ and cherry^65^. Among the different causes taking part in the species-specific LD decay, the mating system (out-crossing versus self-compatible) is probably the most important factor influencing the different LD decays in *Prunus*. While almond and cherry, with some rare exceptions, are out-crossing species, peach and apricot are self-compatible and the subsequent self-pollination results in lower heterozygosity and slower LD decay^66^.

## Conclusion

Future breeding programs, focused on the optimization of consumer perceived quality, need to consider almond VOC modification related to genetic variability, environmental effect, and transformation. This can be achieved only with a more objective and precise identification of the best performing cultivars to be used as superior parental lines in combination with a reliable phenotyping methodology and genotyping assay. This investigation supports the use of PTR-ToF-MS as an accurate and objective phenotyping tool to evaluate the VOC profile of almonds. Indeed, most of the molecules that were previously identified on a limited number of almond accessions by gas chromatographic analysis^33,35,36^ were detected in this broad germplasm collection.

A preliminary GWAS analysis enabled the identification of 63 VOC mass peaks (related to fresh and/or roasted treatment) showing a significant phenotype–genotype association. The detection of molecular markers in close linkage to several aroma components could be of great interest for the set-up of marker-assisted selection (MAS) approaches in novel breeding schemes to enhance the almond aroma. However, a better understanding of genes and enzymes involved in the VOC production, during kernel ripening or during roasting, is still needed. Further studies aimed at a real-time VOC assessment during almond roasting will provide a more complete overview of the volatilome of almond kernel, while the availability of a dedicated almond SNP array will allow a better genetic resolution for the detection of candidate genes regulating the aromatic characteristics of almond.

## Materials and methods

### Plant materials

The germplasm was composed by 106 almond accessions maintained in the ex situ germplasm collection held at the ‘Museo vivente del mandorlo Francesco Monastra’, located in Sicily (latitude: 37.2921094, longitude: 13.5817574, altitude: 121 m above sea level). The germplasm collection was mainly composed of Sicilian almond accessions selected through centuries for their agronomic traits of interest (e.g. fruit quality, resistance to biotic or abiotic stress, shell hardiness) complemented with widely known national and international cultivars as outlined in Supplementary Table 5. For each accession, almond kernels were harvested from four plants, grown under standard agronomical practices. Data related to the pomological characteristics of the fruit and kernel were retrieved from ref. ^67^.

### VOC analysis by proton transfer—time of flight—mass spectrometer

Almond kernels were collected at full ripening stage according to the maturity period of the different accessions (from mid-August to mid-September 2019) and conserved at 4 °C prior to the analysis. Three biological replicates of 3 g of sliced sample, each obtained by five fresh unpeeled almond kernels, were inserted into 20 mL glass vials equipped with PTFE/silicone septa (Agilent, Cernusco sul Naviglio, Italy). Measurements of almond VOCs were performed on three biological replicates with a commercial PTR-ToF–MS 8000 (Ionicon Analytik GmbH, Innsbruck, Austria^12^). The drift tube conditions were as follows: 110 °C drift tube temperature, 2.8 mbar drift pressure, 428 V drift voltage, ion funnel (18 V). This leads to an E/N ratio of about 130 Townsend (Td), with E corresponding to the electric field strength and N to the gas number density (1 Td = 10^−17^ Vcm^−2^). The sampling time per channel of ToF acquisition was 0.1 ns, amounting to 350,000 channels for a mass spectrum ranging up to *m/z* = 400. The sample headspace was withdrawn through PTR-MS inlet with 40 sccm flow for 60 cycles resulting in an analysis time of 60 s/sample. Pure nitrogen was flushed continuously through the vial to prevent pressure drop. Each measurement was conducted automatically after 25 min of sample incubation at 40 °C and 5 min between each measurement was applied in order to prevent memory effect. All steps of measurements were automated by an adapted GC autosampler (MPS Multipurpose Sampler, GERSTEL) coupled to PTR-ToF-MS. After the PTR-ToF-MS measurement of fresh almonds, each vial, without cup, was transferred into an oven (WTB Binder, Germany) at 150 °C for 15 min to achieve a medium roast. These roasting conditions were decided based on literature information^35,36^ and on preliminary tests performed on almond kernel genotypes, characterized by different shapes and sizes profile of roasted almonds was assessed in the same way of fresh samples.

The analysis of PTR-ToF–MS spectra proceeded as described in Farneti et al.^11^. The array of masses detected with PTR-ToF-MS was reduced by applying noise and correlation coefficient thresholds. The first removed peaks that were not significantly different from blank samples; the latter excluded peaks with over 99% correlation, which mostly correspond to isotopes of monoisotopic masses^11^.

R.4.0.2^68^ internal statistical functions and the external packages “mixOmics”, “heatmap3”, “dendextend”, and “ggplot2” were used for the multivariate statistical methods (PCA, heatmap, hierarchical clustering, and tanglegram) and for the “Lollipop graph” employed in this work^69–72^.

### SNP Genotyping and synteny analysis

Total DNA was extracted from fresh leaf tissue using the CTAB extraction method proposed by Doyle and Doyle^73^ following the protocol described by Distefano and colleagues^29^. The almond germplasm collection was genotyped employing the Illumina Infinium^®^18 K Peach SNP array^31^. The use of an SNP array developed for peach is due both to the lack of SNP arrays specifically designed for almond and the high marker transferability between the two species^57^. Robust SNPs were filtered using the ASSIsT software^53^ with default parameters (allowed missing data = 0.05, unexpected genotype threshold = 0.003, frequency rare allele = 0.05). Markers were ordered along the eight linkage groups using the *Prunus dulcis* Texas Genome v2.0^54^, while the *Prunus persica* Whole-Genome Assembly v2.0^56^ was employed for collinearity analysis.

### Deciphering the population structure of the almond collection

The most probable number of subpopulations (*K*) characterizing the 106 accessions was assessed using the STRUCTURE software v2.3.4^58^. The *K* tested ranged from 1 to 10. For each *K*, five independent runs were carried out with a burn-in period of 10,000 and 100,000 Markov chain Monte Carlo replications after burn-in. The *K* value that best fits the data was assessed by calculating the DeltaK value^74^ as implemented in the STRUCTURE HARVESTER program^75^ (http://taylor0.biology.ucla.edu/structureHarvester/). The five independent runs were integrated using the CLUMPP software^76^ and resulting *Q* matrices were displayed in R^68^. Accessions showing a membership coefficient (*Q*_i_) equal or higher than 0.8 were assigned to a subpopulation, while the others were classified as ‘admixed’^77^.

### Phenotype–genotype association analysis

Phenotypic and genotypic data were integrated in a GWAS analysis using the Efficient Mixed-Model Association eXpedited (EMMAX) implemented in the ‘GWAS’ function of the rrBLUP R package^78^. The GWAS model employed in the analysis is expressed as follows:

$$y = X\beta + Zg + S\tau + \varepsilon$$

where *β* is a vector of fixed effects modelling both environmental factors and population structure, the variables *g* and *τ* models the genetic background of each line as a random effect and the additive SNP effect as a fixed effect respectively; *ε* summarizes the residual variance. The GWAS model employed takes genetic structure and kinship matrix as covariates to correct for genetic stratification and parental relationship. To minimize type-one errors, significant associations were detected after correcting the *p* value for multiple testing using the false discovery rate (FDR) method^79^. FDR is computed using the *q* value package in R^80^; SNPs exceeding the FDR threshold rate of 0.05 (represented by a dashed line in the Manhattan plot) were considered significantly associated with the phenotype.

### LD, QTL anchoring and in silico gene annotation

The LD decay was calculated using the R package sommer v2.9^81^. The genome-wide LD decay was visualized using the R software^68^ plotting the LD parameter *r*^2^ against the corresponding physical distance. The 95th percentile of the *r*^2^ distribution was taken as the threshold value to consider two markers in close LD^82^. The *r*^2^ threshold baseline was matched to the locally weighted polynomial regression-based fitting curve (LOESS) to estimate the average LD decay distance using the ‘stats’ R package^68^.

## Supplementary information

Supplementary table 1

Supplementary table 2

Supplementary Table 3

Supplementary Table 4

Supplementary Table 5

Supplementary figure 1

Supplementary figure 2

Supplementary figure 3

Supplementary figure 4

Supplementary figure 5

Supplementary figure 6
