# Supplementary material for: Genetic characterization of an almond germplasm collection and volatilome profiling of raw and roasted kernels
Source: Hortic Res. 2021 Feb 1;8:27. doi: 10.1038/s41438-021-00465-7 (PMC7848010; doi:10.1038/s41438-021-00465-7)
Supplement: Supplementary file 4 — Supplementary Table 4 [file 41438_2021_465_MOESM4_ESM.docx]

| **Supplementary Table 4**: Individual membership coefficient for the most probable number of subpopulations detected by STRUCTURE (K = 3, 7 and 2) |
| --- |

|  | K = 3 | | |  | K = 7 | | | | | | |  | K = 2 | |
| --- | --- | --- | --- | --- | --- | --- | --- | --- | --- | --- | --- | --- | --- | --- |
| 1 | 0.9572 | 0.0142 | 0.0286 |  | 0.002 | 0.462 | 0.523 | 0.002 | 0.003 | 0.007 | 0.001 |  | 0.9818 | 0.0182 |
| 2 | 0.4191 | 0.5565 | 0.0244 |  | 0.02 | 0.342 | 0.198 | 0.186 | 0.131 | 0.008 | 0.116 |  | 0.9576 | 0.0424 |
| 3 | 0.5749 | 0.3401 | 0.085 |  | 0.106 | 0.518 | 0.057 | 0.003 | 0.26 | 0.007 | 0.051 |  | 0.9124 | 0.0876 |
| 4 | 0.6079 | 0.3551 | 0.037 |  | 0.025 | 0.649 | 0.004 | 0.021 | 0.216 | 0.005 | 0.08 |  | 0.968 | 0.032 |
| 5 | 0.2338 | 0.7416 | 0.0246 |  | 0.005 | 0.23 | 0.014 | 0.011 | 0.709 | 0.006 | 0.026 |  | 0.9678 | 0.0322 |
| 6 | 0.001 | 0.001 | 0.998 |  | 0.438 | 0 | 0.001 | 0.001 | 0.001 | 0.559 | 0.001 |  | 0.001 | 0.999 |
| 7 | 0.0926 | 0.0096 | 0.8978 |  | 0.819 | 0.002 | 0.001 | 0.001 | 0.001 | 0.174 | 0.001 |  | 0.0102 | 0.9898 |
| 8 | 0.0058 | 0.9586 | 0.0356 |  | 0.043 | 0.002 | 0.002 | 0.005 | 0.942 | 0.001 | 0.005 |  | 0.8982 | 0.1018 |
| 9 | 0.4576 | 0.0286 | 0.5138 |  | 0.016 | 0.02 | 0.507 | 0.002 | 0.003 | 0.411 | 0.041 |  | 0.4602 | 0.5398 |
| 10 | 0.0048 | 0.003 | 0.9922 |  | 0.001 | 0.001 | 0.001 | 0.001 | 0.001 | 0.996 | 0.001 |  | 0.003 | 0.997 |
| 11 | 0.2478 | 0.646 | 0.1062 |  | 0.046 | 0.13 | 0.159 | 0.001 | 0.657 | 0.005 | 0.002 |  | 0.8482 | 0.1518 |
| 12 | 0.4964 | 0.2608 | 0.2428 |  | 0.246 | 0.453 | 0.079 | 0.102 | 0.028 | 0.067 | 0.026 |  | 0.7294 | 0.2706 |
| 13 | 0.4185 | 0.5775 | 0.004 |  | 0.001 | 0.002 | 0.982 | 0.002 | 0.006 | 0.001 | 0.006 |  | 0.9958 | 0.0042 |
| 14 | 0.5762 | 0.4196 | 0.0042 |  | 0.002 | 0.582 | 0.002 | 0.003 | 0.406 | 0.002 | 0.003 |  | 0.9958 | 0.0042 |
| 15 | 0.3884 | 0.3826 | 0.229 |  | 0.013 | 0.238 | 0.295 | 0.009 | 0.288 | 0.155 | 0.004 |  | 0.7486 | 0.2514 |
| 16 | 0.5527 | 0.4111 | 0.0362 |  | 0.007 | 0.305 | 0.355 | 0.009 | 0.309 | 0.011 | 0.004 |  | 0.962 | 0.038 |
| 17 | 0.0104 | 0.3676 | 0.622 |  | 0.293 | 0.003 | 0.002 | 0.003 | 0.359 | 0.339 | 0.002 |  | 0.2452 | 0.7548 |
| 18 | 0.0068 | 0.4564 | 0.5368 |  | 0.142 | 0.003 | 0.004 | 0.019 | 0.43 | 0.397 | 0.005 |  | 0.3042 | 0.6958 |
| 19 | 0.6077 | 0.3813 | 0.011 |  | 0.007 | 0.561 | 0.089 | 0.017 | 0.32 | 0.005 | 0.002 |  | 0.9872 | 0.0128 |
| 20 | 0.0538 | 0.8413 | 0.105 |  | 0.053 | 0.006 | 0.235 | 0.003 | 0.668 | 0.011 | 0.023 |  | 0.8902 | 0.1098 |
| 21 | 0.846 | 0.0214 | 0.1326 |  | 0.126 | 0.533 | 0.302 | 0.001 | 0.021 | 0.016 | 0.001 |  | 0.8714 | 0.1286 |
| 22 | 0.619 | 0.3714 | 0.0096 |  | 0.003 | 0.641 | 0.001 | 0.015 | 0.325 | 0.005 | 0.009 |  | 0.9898 | 0.0102 |
| 23 | 0.0386 | 0.8024 | 0.159 |  | 0.001 | 0 | 0.001 | 0.001 | 0.001 | 0 | 0.997 |  | 0.8382 | 0.1618 |
| 24 | 0.1092 | 0.8792 | 0.0116 |  | 0.011 | 0.037 | 0.079 | 0.015 | 0.78 | 0.004 | 0.075 |  | 0.957 | 0.043 |
| 25 | 0.7008 | 0.259 | 0.0402 |  | 0.038 | 0.637 | 0.033 | 0.01 | 0.123 | 0.009 | 0.15 |  | 0.9616 | 0.0384 |
| 26 | 0.8706 | 0.1172 | 0.0122 |  | 0.002 | 0.651 | 0.32 | 0.003 | 0.014 | 0.006 | 0.003 |  | 0.9894 | 0.0106 |
| 27 | 0.9036 | 0.0338 | 0.0626 |  | 0.035 | 0.844 | 0.057 | 0.008 | 0.01 | 0.041 | 0.005 |  | 0.946 | 0.054 |
| 28 | 0.275 | 0.0784 | 0.6466 |  | 0.125 | 0.001 | 0.543 | 0.001 | 0.001 | 0.326 | 0.003 |  | 0.3142 | 0.6858 |
| 29 | 0.4905 | 0.4969 | 0.0126 |  | 0.006 | 0.338 | 0.301 | 0.06 | 0.27 | 0.005 | 0.02 |  | 0.9856 | 0.0144 |
| 30 | 0.001 | 0.001 | 0.998 |  | 0.431 | 0 | 0.001 | 0.001 | 0.001 | 0.566 | 0.001 |  | 0.001 | 0.999 |
| 31 | 0.964 | 0.0176 | 0.0184 |  | 0.004 | 0.651 | 0.327 | 0.003 | 0.005 | 0.004 | 0.005 |  | 0.9838 | 0.0162 |
| 32 | 0.0064 | 0.1872 | 0.8064 |  | 0.002 | 0.001 | 0.001 | 0.001 | 0.204 | 0.788 | 0.002 |  | 0.0254 | 0.9746 |
| 33 | 0.0304 | 0.9624 | 0.0072 |  | 0.004 | 0.021 | 0.002 | 0.028 | 0.852 | 0.002 | 0.09 |  | 0.9896 | 0.0104 |
| 34 | 0.9784 | 0.0142 | 0.0074 |  | 0.004 | 0.976 | 0.001 | 0.004 | 0.004 | 0.002 | 0.01 |  | 0.9926 | 0.0074 |
| 35 | 0.105 | 0.0234 | 0.8716 |  | 0.868 | 0.001 | 0.001 | 0.002 | 0.002 | 0.125 | 0.001 |  | 0.011 | 0.989 |
| 36 | 0.1417 | 0.0258 | 0.8325 |  | 0.644 | 0.012 | 0.001 | 0.032 | 0.002 | 0.307 | 0.001 |  | 0.0492 | 0.9508 |
| 37 | 0.788 | 0.1846 | 0.0274 |  | 0.006 | 0.375 | 0.586 | 0.005 | 0.018 | 0.005 | 0.005 |  | 0.9806 | 0.0194 |
| 38 | 0.9834 | 0.0136 | 0.003 |  | 0.002 | 0.958 | 0.003 | 0.031 | 0.003 | 0.001 | 0.002 |  | 0.9978 | 0.0022 |
| 39 | 0.9794 | 0.0096 | 0.011 |  | 0.003 | 0.973 | 0.001 | 0.005 | 0.002 | 0.004 | 0.011 |  | 0.9862 | 0.0138 |
| 40 | 0.6581 | 0.3319 | 0.01 |  | 0.003 | 0.624 | 0.048 | 0.003 | 0.311 | 0.008 | 0.002 |  | 0.9908 | 0.0092 |
| 41 | 0.3843 | 0.5421 | 0.0736 |  | 0.039 | 0.265 | 0.184 | 0.004 | 0.473 | 0.031 | 0.004 |  | 0.883 | 0.117 |
| 42 | 0.1774 | 0.7096 | 0.113 |  | 0.123 | 0.147 | 0.006 | 0.006 | 0.694 | 0.021 | 0.004 |  | 0.778 | 0.222 |
| 43 | 0.0022 | 0.9948 | 0.003 |  | 0.001 | 0.001 | 0.002 | 0.005 | 0.985 | 0.001 | 0.005 |  | 0.977 | 0.023 |
| 44 | 0.006 | 0.9668 | 0.0272 |  | 0.001 | 0 | 0 | 0.997 | 0.001 | 0 | 0 |  | 0.8726 | 0.1274 |
| 45 | 0.1815 | 0.1118 | 0.7067 |  | 0.227 | 0.046 | 0.07 | 0.002 | 0.168 | 0.484 | 0.003 |  | 0.2236 | 0.7764 |
| 46 | 0.1946 | 0.3346 | 0.4708 |  | 0.234 | 0.003 | 0.33 | 0.007 | 0.012 | 0.171 | 0.243 |  | 0.4804 | 0.5196 |
| 47 | 0.6109 | 0.3305 | 0.0586 |  | 0.032 | 0.609 | 0.07 | 0.05 | 0.194 | 0.033 | 0.013 |  | 0.9342 | 0.0658 |
| 48 | 0.0094 | 0.2152 | 0.7754 |  | 0.995 | 0.001 | 0.001 | 0.001 | 0.001 | 0.001 | 0.001 |  | 0.0132 | 0.9868 |
| 49 | 0.3017 | 0.5055 | 0.1928 |  | 0.025 | 0.006 | 0.452 | 0.012 | 0.052 | 0.084 | 0.369 |  | 0.7822 | 0.2178 |
| 50 | 0.4058 | 0.5888 | 0.0054 |  | 0.002 | 0.001 | 0.938 | 0.006 | 0.007 | 0.001 | 0.045 |  | 0.994 | 0.006 |
| 51 | 0.0048 | 0.003 | 0.9922 |  | 0.001 | 0.001 | 0.001 | 0.001 | 0.001 | 0.996 | 0.001 |  | 0.003 | 0.997 |
| 52 | 0.013 | 0.4896 | 0.4974 |  | 0.098 | 0.004 | 0.07 | 0.406 | 0.072 | 0.342 | 0.008 |  | 0.4404 | 0.5596 |
| 53 | 0.0082 | 0.5824 | 0.4094 |  | 0.005 | 0.002 | 0.001 | 0.001 | 0.641 | 0.347 | 0.002 |  | 0.4836 | 0.5164 |
| 54 | 0.9386 | 0.039 | 0.0224 |  | 0.155 | 0.801 | 0.004 | 0.003 | 0.034 | 0.001 | 0.002 |  | 0.9738 | 0.0262 |
| 55 | 0.1494 | 0.013 | 0.8376 |  | 0.862 | 0.004 | 0.003 | 0.003 | 0.002 | 0.123 | 0.003 |  | 0.0282 | 0.9718 |
| 56 | 0.0098 | 0.2174 | 0.7728 |  | 0.995 | 0.001 | 0.001 | 0.001 | 0.001 | 0.001 | 0.001 |  | 0.0136 | 0.9864 |
| 57 | 0.2409 | 0.7543 | 0.0048 |  | 0.002 | 0.018 | 0.262 | 0.005 | 0.71 | 0.001 | 0.001 |  | 0.994 | 0.006 |
| 58 | 0.3071 | 0.0154 | 0.6775 |  | 0.203 | 0.257 | 0.014 | 0.001 | 0.004 | 0.482 | 0.039 |  | 0.257 | 0.743 |
| 59 | 0.0082 | 0.2098 | 0.782 |  | 0.995 | 0.001 | 0.001 | 0.001 | 0.001 | 0.001 | 0.001 |  | 0.0098 | 0.9902 |
| 60 | 0.7531 | 0.199 | 0.048 |  | 0.011 | 0.659 | 0.114 | 0.005 | 0.17 | 0.04 | 0.002 |  | 0.9518 | 0.0482 |
| 61 | 0.0378 | 0.803 | 0.1592 |  | 0.001 | 0 | 0.001 | 0.001 | 0.001 | 0 | 0.997 |  | 0.8384 | 0.1616 |
| 62 | 0.004 | 0.4298 | 0.5662 |  | 0.349 | 0.001 | 0.003 | 0.003 | 0.406 | 0.234 | 0.004 |  | 0.2558 | 0.7442 |
| 63 | 0.988 | 0.0066 | 0.0054 |  | 0.007 | 0.974 | 0.007 | 0.004 | 0.002 | 0.001 | 0.005 |  | 0.9948 | 0.0052 |
| 64 | 0.1502 | 0.4952 | 0.3546 |  | 0.11 | 0.016 | 0.189 | 0.005 | 0.048 | 0.222 | 0.41 |  | 0.6 | 0.4 |
| 65 | 0.9784 | 0.014 | 0.0076 |  | 0.004 | 0.975 | 0.001 | 0.004 | 0.003 | 0.002 | 0.01 |  | 0.9926 | 0.0074 |
| 66 | 0.3804 | 0.6166 | 0.003 |  | 0.001 | 0.351 | 0.014 | 0.072 | 0.559 | 0.001 | 0.002 |  | 0.997 | 0.003 |
| 67 | 0.3523 | 0.6437 | 0.004 |  | 0.002 | 0.08 | 0.531 | 0.004 | 0.38 | 0.001 | 0.002 |  | 0.9948 | 0.0052 |
| 68 | 0.1142 | 0.8734 | 0.0124 |  | 0.002 | 0.023 | 0.005 | 0.003 | 0.957 | 0.008 | 0.002 |  | 0.9854 | 0.0146 |
| 69 | 0.001 | 0.001 | 0.998 |  | 0.438 | 0 | 0.001 | 0.001 | 0.001 | 0.56 | 0.001 |  | 0.001 | 0.999 |
| 70 | 0.2841 | 0.62 | 0.096 |  | 0.163 | 0.036 | 0.349 | 0.267 | 0.168 | 0.002 | 0.016 |  | 0.859 | 0.141 |
| 71 | 0.7342 | 0.2608 | 0.005 |  | 0.004 | 0.772 | 0.003 | 0.069 | 0.02 | 0.001 | 0.132 |  | 0.9958 | 0.0042 |
| 72 | 0.6276 | 0.2874 | 0.085 |  | 0.011 | 0.293 | 0.594 | 0.001 | 0.08 | 0.012 | 0.009 |  | 0.9214 | 0.0786 |
| 73 | 0.6656 | 0.1582 | 0.1762 |  | 0.17 | 0.538 | 0.174 | 0.009 | 0.049 | 0.048 | 0.012 |  | 0.8204 | 0.1796 |
| 74 | 0.1854 | 0.1108 | 0.7038 |  | 0.219 | 0.048 | 0.072 | 0.002 | 0.169 | 0.487 | 0.004 |  | 0.2262 | 0.7738 |
| 75 | 0.002 | 0.995 | 0.003 |  | 0.001 | 0.001 | 0.002 | 0.005 | 0.985 | 0.001 | 0.005 |  | 0.977 | 0.023 |
| 76 | 0.8942 | 0.1004 | 0.0054 |  | 0.011 | 0.89 | 0.01 | 0.004 | 0.081 | 0.002 | 0.003 |  | 0.995 | 0.005 |
| 77 | 0.4078 | 0.0214 | 0.5708 |  | 0.394 | 0.003 | 0.453 | 0.002 | 0.001 | 0.142 | 0.005 |  | 0.3944 | 0.6056 |
| 78 | 0.9886 | 0.0042 | 0.0072 |  | 0.005 | 0.978 | 0.002 | 0.002 | 0.001 | 0.002 | 0.011 |  | 0.9908 | 0.0092 |
| 79 | 0.0064 | 0.1883 | 0.8053 |  | 0.002 | 0.002 | 0.001 | 0.001 | 0.204 | 0.788 | 0.002 |  | 0.0264 | 0.9736 |
| 80 | 0.2752 | 0.072 | 0.6528 |  | 0.133 | 0.001 | 0.539 | 0.001 | 0.001 | 0.322 | 0.002 |  | 0.3076 | 0.6924 |
| 81 | 0.6547 | 0.3263 | 0.019 |  | 0.003 | 0.619 | 0.049 | 0.084 | 0.207 | 0.032 | 0.006 |  | 0.9816 | 0.0184 |
| 82 | 0.2356 | 0.7596 | 0.0048 |  | 0.002 | 0.017 | 0.259 | 0.005 | 0.714 | 0.001 | 0.001 |  | 0.9942 | 0.0058 |
| 83 | 0.3638 | 0.6134 | 0.0228 |  | 0.102 | 0.26 | 0.09 | 0.003 | 0.477 | 0.002 | 0.066 |  | 0.9642 | 0.0358 |
| 84 | 0.9944 | 0.003 | 0.0026 |  | 0.002 | 0.992 | 0.002 | 0.001 | 0.001 | 0.001 | 0.001 |  | 0.9968 | 0.0032 |
| 85 | 0.9942 | 0.0032 | 0.0026 |  | 0.002 | 0.992 | 0.002 | 0.001 | 0.001 | 0.001 | 0.001 |  | 0.9968 | 0.0032 |
| 86 | 0.9908 | 0.0052 | 0.004 |  | 0.001 | 0.519 | 0.475 | 0.001 | 0.001 | 0.002 | 0.001 |  | 0.995 | 0.005 |
| 87 | 0.0614 | 0.2602 | 0.6784 |  | 0.185 | 0.022 | 0.027 | 0.009 | 0.255 | 0.499 | 0.002 |  | 0.2314 | 0.7686 |
| 88 | 0.9846 | 0.0072 | 0.0082 |  | 0.009 | 0.966 | 0.01 | 0.005 | 0.003 | 0.006 | 0.002 |  | 0.9926 | 0.0074 |
| 89 | 0.4931 | 0.5039 | 0.003 |  | 0.004 | 0.565 | 0.005 | 0.166 | 0.132 | 0.001 | 0.128 |  | 0.997 | 0.003 |
| 90 | 0.0046 | 0.0028 | 0.9926 |  | 0.001 | 0.001 | 0.001 | 0.001 | 0.001 | 0.996 | 0.001 |  | 0.003 | 0.997 |
| 91 | 0.6242 | 0.3718 | 0.004 |  | 0.011 | 0.506 | 0.149 | 0.011 | 0.307 | 0.001 | 0.014 |  | 0.9964 | 0.0036 |
| 92 | 0.7988 | 0.1476 | 0.0536 |  | 0.014 | 0.738 | 0.012 | 0.003 | 0.151 | 0.078 | 0.005 |  | 0.9414 | 0.0586 |
| 93 | 0.006 | 0.967 | 0.027 |  | 0.001 | 0 | 0 | 0.997 | 0.001 | 0 | 0 |  | 0.8736 | 0.1264 |
| 94 | 0.914 | 0.0616 | 0.0244 |  | 0.041 | 0.905 | 0.01 | 0.011 | 0.023 | 0.005 | 0.006 |  | 0.9806 | 0.0194 |
| 95 | 0.2865 | 0.6041 | 0.1094 |  | 0.025 | 0.187 | 0.241 | 0.09 | 0.384 | 0.048 | 0.025 |  | 0.8674 | 0.1326 |
| 96 | 0.006 | 0.9584 | 0.0356 |  | 0.043 | 0.002 | 0.002 | 0.005 | 0.942 | 0.001 | 0.005 |  | 0.8986 | 0.1014 |
| 97 | 0.3372 | 0.0176 | 0.6452 |  | 0.015 | 0.314 | 0.042 | 0.021 | 0.005 | 0.581 | 0.022 |  | 0.3024 | 0.6976 |
| 98 | 0.0058 | 0.9674 | 0.0268 |  | 0.001 | 0 | 0 | 0.997 | 0 | 0 | 0 |  | 0.8734 | 0.1266 |
| 99 | 0.4628 | 0.1484 | 0.3888 |  | 0.055 | 0.024 | 0.606 | 0.003 | 0.007 | 0.251 | 0.054 |  | 0.5954 | 0.4046 |
| 100 | 0.6743 | 0.3119 | 0.0138 |  | 0.086 | 0.576 | 0.22 | 0.066 | 0.044 | 0.002 | 0.008 |  | 0.9866 | 0.0134 |
| 101 | 0.1821 | 0.0274 | 0.7905 |  | 0.78 | 0.05 | 0.005 | 0.003 | 0.006 | 0.155 | 0.001 |  | 0.0916 | 0.9084 |
| 102 | 0.7554 | 0.1446 | 0.1 |  | 0.077 | 0.595 | 0.191 | 0.004 | 0.087 | 0.041 | 0.005 |  | 0.892 | 0.108 |
| 103 | 0.0226 | 0.172 | 0.8054 |  | 0.03 | 0.004 | 0.159 | 0.007 | 0.031 | 0.702 | 0.068 |  | 0.1218 | 0.8782 |
| 104 | 0.7442 | 0.203 | 0.0528 |  | 0.094 | 0.582 | 0.229 | 0.009 | 0.08 | 0.004 | 0.003 |  | 0.9522 | 0.0478 |
| 105 | 0.8756 | 0.0748 | 0.0496 |  | 0.041 | 0.635 | 0.187 | 0.002 | 0.005 | 0.005 | 0.126 |  | 0.9646 | 0.0354 |
| 106 | 0.7131 | 0.1292 | 0.1576 |  | 0.258 | 0.596 | 0.032 | 0.004 | 0.101 | 0.004 | 0.005 |  | 0.8286 | 0.1714 |
