# Supplementary material for: Genetic characterization of an almond germplasm collection and volatilome profiling of raw and roasted kernels
Source: Hortic Res. 2021 Feb 1;8:27. doi: 10.1038/s41438-021-00465-7 (PMC7848010; doi:10.1038/s41438-021-00465-7)
Supplement: Supplementary file 5 — Supplementary Table 5 [file 41438_2021_465_MOESM5_ESM.docx]

**Supplementary table 5**: List of the 106 Prunus dulcis accessions with origin and pomological information. The qualitative traits were measured as specified in appendix.

| Id_number | Full name | Origin | Fruit weight (g) | Kernel weight (g) | Kernel thickness (mm) | Shell texture (N) | % of double seeds | Kernel shape* | Production** | Flavour*** |
| --- | --- | --- | --- | --- | --- | --- | --- | --- | --- | --- |
| 1 | Acquaviva | Sicily | 4,97 | 1,14 | 7,4 | 22,91 | 0 | 1 | 2 | 2 |
| 2 | Amara di Martorana | Sicily | 5 | 1,18 | 6,6 | 23,56 | 11 | 3 | 2 | 3 |
| 3 | Angelica | Sicily | 4,52 | 1,09 | 6 | 24,16 | 0 | 5 | 1 | 2 |
| 4 | Baggiana | Sicily | 5,18 | 0,94 | 6,7 | 18,14 | 1 | 1 | 1 | 1 |
| 5 | Bargellera | Sicily | 6,56 | 1,09 | 6,4 | 16,56 | 38 | 5 | 2 | 1 |
| 6 | Bari Flores | Apulia | 5,58 | 1,02 | 7 | 18,35 | 13 | 5 | 2 | 2 |
| 7 | Bari Sabittisa | Apulia | 6,29 | 0,98 | 6,5 | 15,6 | 2 | 2 | 2 | 2 |
| 8 | Barunissa | Sicily | 4,39 | 1,06 | 6,9 | 24,16 | 2 | 1 | 2 | 1 |
| 9 | Belvedere | Sicily | 4,86 | 0,88 | 6,7 | 18,16 | 16 | 1 | 1 | 1 |
| 10 | Bennici | Sicily | 4,36 | 0,74 | 6,4 | 16,92 | 1 | 2 | 3 | 1 |
| 11 | Bianculidda di Pezzino | Sicily | 4,72 | 0,97 | 6,3 | 20,55 | 0 | 3 | 2 | 2 |
| 12 | Bottara | Sicily | 6,95 | 1,6 | 7,5 | 23,01 | 21 | 1 | 2 | 1 |
| 13 | Bronte 1 | Sicily | 4,86 | 1,05 | 6,7 | 21,68 | 3 | 1 | 2 | 1 |
| 14 | Buscemi | Sicily | 5,13 | 1,26 | 8,2 | 24,55 | 0 | 5 | 1 | 1 |
| 15 | Cacciatura | Sicily | 2,99 | 0,96 | 6,8 | 32,28 | 7 | 3 | 2 | 1 |
| 16 | Cacinova | Sicily | 5,63 | 1,42 | 6,5 | 25,16 | 0 | 4 | 2 | 1 |
| 17 | Calamonaci | Sicily | 5,01 | 0,92 | 6,5 | 18,46 | 54 | 1 | 3 | 3 |
| 18 | Callara | Sicily | 5,48 | 1,23 | 7,3 | 22,41 | 22 | 1 | 2 | 1 |
| 19 | Caluriedda | Sicily | 5,44 | 1,31 | 8 | 24,03 | 1 | 1 | 2 | 2 |
| 20 | Carrubina | Sicily | 3,9 | 1,11 | 8,1 | 28,56 | 13 | 2 | 2 | 1 |
| 21 | Catrubba | Sicily | 5,76 | 1,43 | 10 | 24,78 | 2 | 5 | 1 | 1 |
| 22 | Cavalera Aragona | Sicily | 2,36 | 1,11 | 8,9 | 46,88 | 0 | 1 | 1 | 1 |
| 23 | Cavalera Naro | Sicily | 5,06 | 1,09 | 6,8 | 21,62 | 45 | 2 | 3 | 2 |
| 24 | Cavaliere | Sicily | 5,87 | 1,29 | 8,4 | 21,94 | 6 | 1 | 2 | 1 |
| 25 | Cesaro 1 | Sicily | 4,34 | 1,05 | 6,5 | 24,25 | 1 | 1 | 2 | 3 |
| 26 | Chiarchiara | Sicily | 5,49 | 1,16 | 8,1 | 21,07 | 5 | 5 | 3 | 1 |
| 27 | Chiricupara | Sicily | 5,63 | 1,09 | 8 | 19,39 | 15 | 1 | 3 | 1 |
| 28 | Comunista | Sicily | 4,76 | 1,15 | 8 | 24,13 | 21 | 1 | 3 | 1 |
| 29 | Cumma | Sicily | 4,27 | 1,12 | 7,4 | 26,28 | 1 | 2 | 2 | 2 |
| 30 | Cuore | Sicily | 4,9 | 1,02 | 7,1 | 20,9 | 8 | 5 | 2 | 1 |
| 31 | Cupani piccola | Sicily | 4,19 | 0,84 | 5,9 | 19,91 | 7 | 1 | 2 | 1 |
| 32 | Cuti | Sicily | 2,54 | 1,14 | 7,5 | 44,74 | 0 | 1 | 1 | 1 |
| 33 | Di Giorgio | Sicily | 2,92 | 0,91 | 6,4 | 31,09 | 5 | 1 | 3 | 1 |
| 34 | Don Filippo | Sicily | 2,7 | 1,06 | 7,6 | 39,26 | 1 | 1 | 2 | 1 |
| 35 | Don Peppino | Sicily | 5,24 | 0,79 | 5,8 | 15,1 | 23 | 1 | 1 | 1 |
| 36 | Don Pitrino | Sicily | 4,64 | 0,67 | 6 | 14,37 | 23 | 1 | 2 | 1 |
| 37 | Enna 2 | Sicily | 5,83 | 1,15 | 7,1 | 16,93 | 16 | 3 | 1 | 1 |
| 38 | Falsa pizzuta | Sicily | 5,24 | 1,08 | 7,1 | 20,54 | 20 | 3 | 2 | 2 |
| 39 | Fascionello | Sicily | 5,04 | 1,14 | 6,8 | 22,67 | 24 | 3 | 2 | 1 |
| 40 | Fastuchina | Sicily | 3,84 | 0,81 | 6,5 | 21,07 | 0 | 4 | 2 | 1 |
| 41 | Favaro | Sicily | 4,88 | 1,26 | 8 | 25,77 | 22 | 1 | 2 | 1 |
| 42 | Favaro’ 2 | Sicily | 4 | 0,81 | 6,3 | 20,19 | 62 | 1 | 2 | 2 |
| 43 | Fellamasa Casteltermini | Sicily | 3,75 | 1,18 | 9,7 | 31,36 | 15 | 4 | 3 | 2 |
| 44 | Ferraduel | International | 2,63 | 1,51 | 9,7 | 57,63 | 1 | 5 | 1 | 1 |
| 45 | Ferragnes | International | 4,89 | 1,46 | 7,6 | 29,76 | 0 | 3 | 1 | 1 |
| 46 | Filippoceo | Sicily | 4,16 | 1,2 | 8,3 | 28,86 | 9 | 5 | 3 | 1 |
| 47 | Filippazzo | Sicily | 4,28 | 0,89 | 6,9 | 20,79 | 0 | 3 | 1 | 1 |
| 48 | Gaglio | Sicily | 4,5 | 1,03 | 7,3 | 22,81 | 5 | 1 | 2 | 2 |
| 49 | Genco | Apulia | 4,27 | 1,35 | 8,8 | 31,56 | 1 | 1 | 2 | 1 |
| 50 | Giardinella | Sicily | 4,98 | 1,12 | 6,3 | 22,55 | 5 | 5 | 2 | 3 |
| 51 | Griddetta | Sicily | 5,04 | 0,95 | 6,2 | 18,77 | 0 | 1 | 3 | 1 |
| 52 | Laurenne | International | 4,46 | 1,22 | 6,9 | 27,45 | 0 | 3 | 2 | 1 |
| 53 | Lisciannarisa | Sicily | 4,29 | 1,08 | 7,5 | 25,26 | 31 | 1 | 3 | 1 |
| 54 | Lumia | Sicily | 6,59 | 1,19 | 7 | 18,09 | 15 | 1 | 1 | 1 |
| 55 | Marra Mennuladi | Sicily | 4,43 | 1,23 | 7,8 | 27,86 | 8 | 1 | 3 | 1 |
| 56 | Mastraciccia | Sicily | 3,62 | 0,99 | 8 | 27,4 | 1 | 5 | 1 | NA |
| 57 | Mennula du nigliu | Sicily | 7,57 | 1,28 | 7,3 | 16,94 | 5 | 2 | 1 | 1 |
| 58 | Mennula du vattiu | Sicily | 5,68 | 1,13 | 6,9 | 19,96 | 7 | 3 | 1 | 1 |
| 59 | Mezza lira | Sicily | 3,93 | 1 | 8,3 | 25,48 | 1 | 2 | 1 | 1 |
| 60 | Milocca | Sicily | 3,36 | 0,77 | 6,4 | 22,78 | 3 | 1 | 1 | 1 |
| 61 | Mirabile | Sicily | 4,8 | 1,08 | 7,2 | 22,62 | 11 | 1 | 1 | 2 |
| 62 | Miricanedda | Sicily | 4,93 | 1,1 | 7,2 | 22,26 | 36 | 2 | 1 | 1 |
| 63 | Miuzza | Sicily | 6,59 | 1,33 | 8 | 20,19 | 9 | 1 | 2 | 1 |
| 64 | Montagna | Sicily | 4,45 | 0,75 | 6,2 | 16,8 | 0 | 2 | 2 | 1 |
| 65 | Mullisa Tonda | Sicily | 2,42 | 1,36 | 10,4 | 56,22 | 2 | 1 | 3 | 1 |
| 66 | Mullisa Grande | Sicily | 5,52 | 1,28 | 7,4 | 23,29 | 0 | 1 | 2 | 1 |
| 67 | Mullisa Piccola | Sicily | 4,85 | 1 | 6,9 | 20,67 | 12 | 5 | 3 | 2 |
| 68 | Nambaredda | Sicily | 3,04 | 0,87 | 7,4 | 28,64 | 34 | 5 | 1 | 1 |
| 69 | Naro 1 | Sicily | 5,46 | 1,16 | 7,5 | 21,33 | 8 | 4 | 1 | 1 |
| 70 | Niveramanza | Sicily | 5,25 | 1,1 | 7,9 | 20,86 | 34 | 1 | 2 | 1 |
| 71 | Nuciddara | Sicily | 5,18 | 1,09 | 6,8 | 20,96 | 37 | 2 | 2 | 1 |
| 72 | Palma | Sicily | 4,25 | 1,18 | 7,5 | 27,9 | 2 | 4 | 2 | 2 |
| 73 | Perciavisazza | Sicily | 9,16 | 1,2 | 7,4 | 13,15 | 15 | 3 | 2 | 2 |
| 74 | Persichina | Sicily | 4,58 | 1,55 | 8,3 | 33,75 | 0 | 3 | 1 | 1 |
| 75 | Piattamollisa | Sicily | 4,39 | 1,24 | 8,5 | 28,18 | 13 | 5 | 3 | 1 |
| 76 | Pilusedda | Sicily | 3,55 | 0,95 | 7,9 | 27,15 | 0 | 2 | 2 | 2 |
| 77 | Pizzuta Contino | Sicily | 6 | 0,91 | 5,9 | 15,18 | 29 | 3 | 3 | 1 |
| 78 | Pizzuta d'Avola | Sicily | 4,71 | 1,05 | 7,2 | 22,35 | 42 | 3 | 2 | 2 |
| 79 | Pizzutella | Sicily | 6,56 | 1,31 | 8,6 | 19,95 | 0 | 1 | 1 | 1 |
| 80 | Pizzuta Grande | Sicily | 4,58 | 0,93 | 7,5 | 20,23 | 34 | 1 | 1 | 1 |
| 81 | Rapparina | Sicily | 3,98 | 1,05 | 7,4 | 26,45 | 3 | 1 | 1 | 1 |
| 82 | Regina | Sicily | 3,9 | 0,98 | 7,7 | 25,21 | 18 | 1 | 2 | 2 |
| 83 | Reginella | Sicily | 4,01 | 0,85 | 7,9 | 21,28 | 40 | 5 | 2 | 2 |
| 84 | Romana Ispica | Sicily | 3,75 | 1,01 | 7,8 | 27 | 24 | 1 | 1 | 1 |
| 85 | Romana Licata | Sicily | 5,9 | 0,97 | 6,8 | 16,44 | 27 | 5 | 2 | 1 |
| 86 | Sancisuca | Sicily | 3,54 | 0,86 | 7 | 24,18 | 7 | 2 | 2 | 1 |
| 87 | Sarbaggia di Sciascia | Sicily | 5,24 | 1,06 | 7,1 | 20,24 | 2 | 1 | 1 | 1 |
| 88 | Sarbaggia di Vitello | Sicily | 5,25 | 1,03 | 7,7 | 19,55 | 17 | 1 | 1 | 1 |
| 89 | Sarbaggia di Patito | Sicily | 5,96 | 1,17 | 6,3 | 19,62 | 28 | 2 | 1 | 1 |
| 90 | Scummissa | Sicily | 6,07 | 1,09 | 5,9 | 18,02 | 4 | 2 | 2 | 1 |
| 91 | Selvatica Favata | Sicily | 5,92 | 1,33 | 7,9 | 22,51 | 11 | 5 | 1 | 1 |
| 92 | Staccia | Sicily | 4,73 | 1,26 | 7,3 | 26,68 | 8 | 4 | 2 | 2 |
| 93 | Supernova | Apulia | 4,65 | 1,5 | 7,9 | 32,32 | 3 | 1 | 3 | 1 |
| 94 | Tabacchina | Sicily | 2,41 | 0,96 | 7,5 | 39,92 | 0 | 1 | 1 | 1 |
| 95 | Texas | International | 2,85 | 0,98 | 8,7 | 34,39 | 6 | 1 | 2 | 1 |
| 96 | Tricula | Sicily | 4,52 | 1,09 | 6 | 24,16 | 0 | 1 | 2 | 1 |
| 97 | Tunnulidda | Sicily | 4,59 | 0,85 | 7,2 | 18,44 | 17 | 5 | 3 | 1 |
| 98 | Tuono | Apulia | 5,01 | 1,38 | 7,5 | 27,62 | 7 | 1 | 2 | 1 |
| 99 | Universo | Apulia | 8,24 | 1,3 | 7,3 | 15,75 | 23 | 1 | 3 | 1 |
| 100 | Uova di Cucco | Sicily | 5,59 | 0,89 | 6 | 15,99 | 0 | 4 | 2 | 2 |
| 101 | Vaiana | Sicily | 4,67 | 1,02 | 6,4 | 21,9 | 3 | 5 | 1 | 2 |
| 102 | Zaccaneddara | Sicily | 5,32 | 1,05 | 7,1 | 19,67 | 10 | 3 | 2 | 2 |
| 103 | Zagarri’ | Sicily | 3,76 | 1,06 | 7,1 | 28,22 | 15 | 5 | 2 | 1 |
| 104 | Zammuto 2 | Sicily | 5,69 | 1,11 | 7,1 | 19,54 | 27 | 2 | 2 | 2 |
| 105 | Zarbara | Sicily | 3,61 | 1,15 | 7,3 | 31,84 | 3 | 3 | 2 | 1 |
| 106 | Zottafunnuta | Sicily | 4,99 | 1,4 | 8,6 | 28,06 | 1 | 3 | 3 | 1 |

* Kernel shape: 1 very thin, 2 thin, 3 medium, 4 thick. 5 very thick

** Production: 1 low, 2 medium, 3 high

*** Flavour: 1 sweet, 2 slightly bitter, 3 bitter
