# Supplementary figures and images for: Genetic characterization of an almond germplasm collection and volatilome profiling of raw and roasted kernels
Source: Hortic Res. 2021 Feb 1;8:27. doi: 10.1038/s41438-021-00465-7 (PMC7848010; doi:10.1038/s41438-021-00465-7)

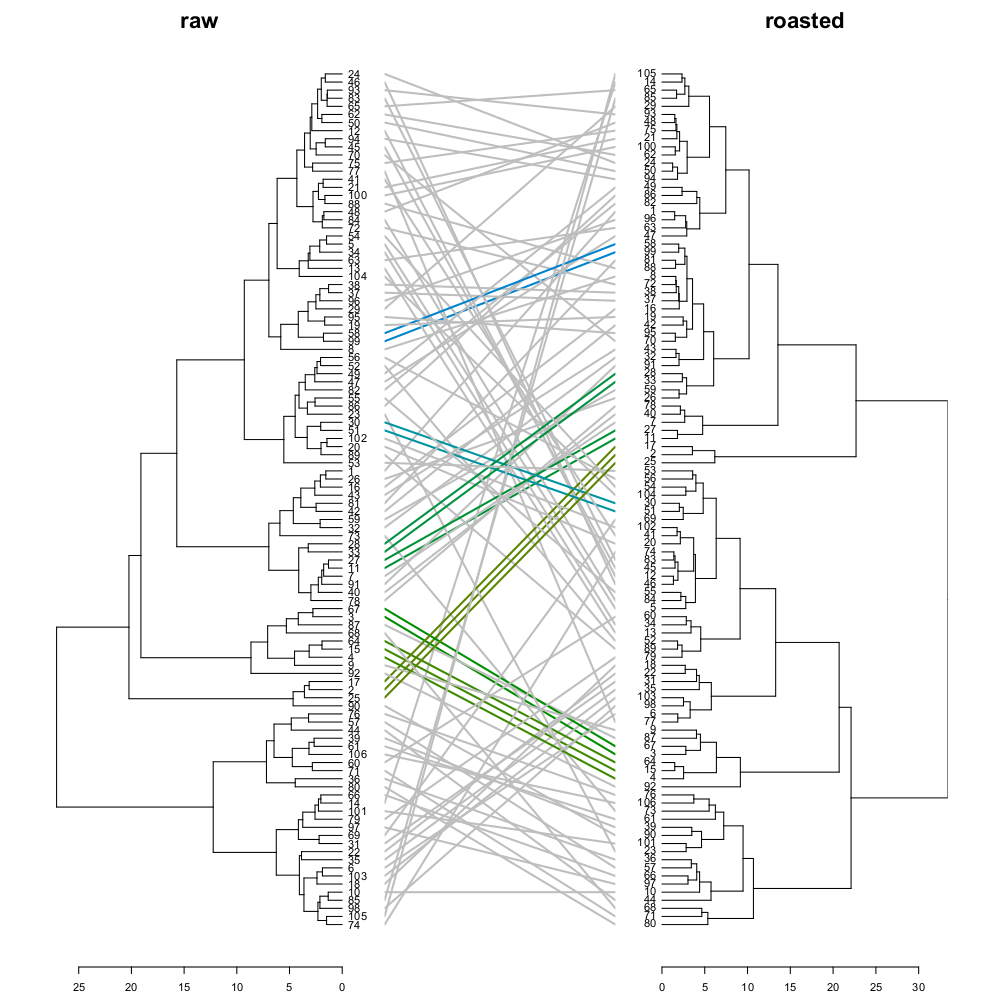

Supplement: Supplementary file 7 — Supplementary figure 2 [file 41438_2021_465_MOESM7_ESM.tif]

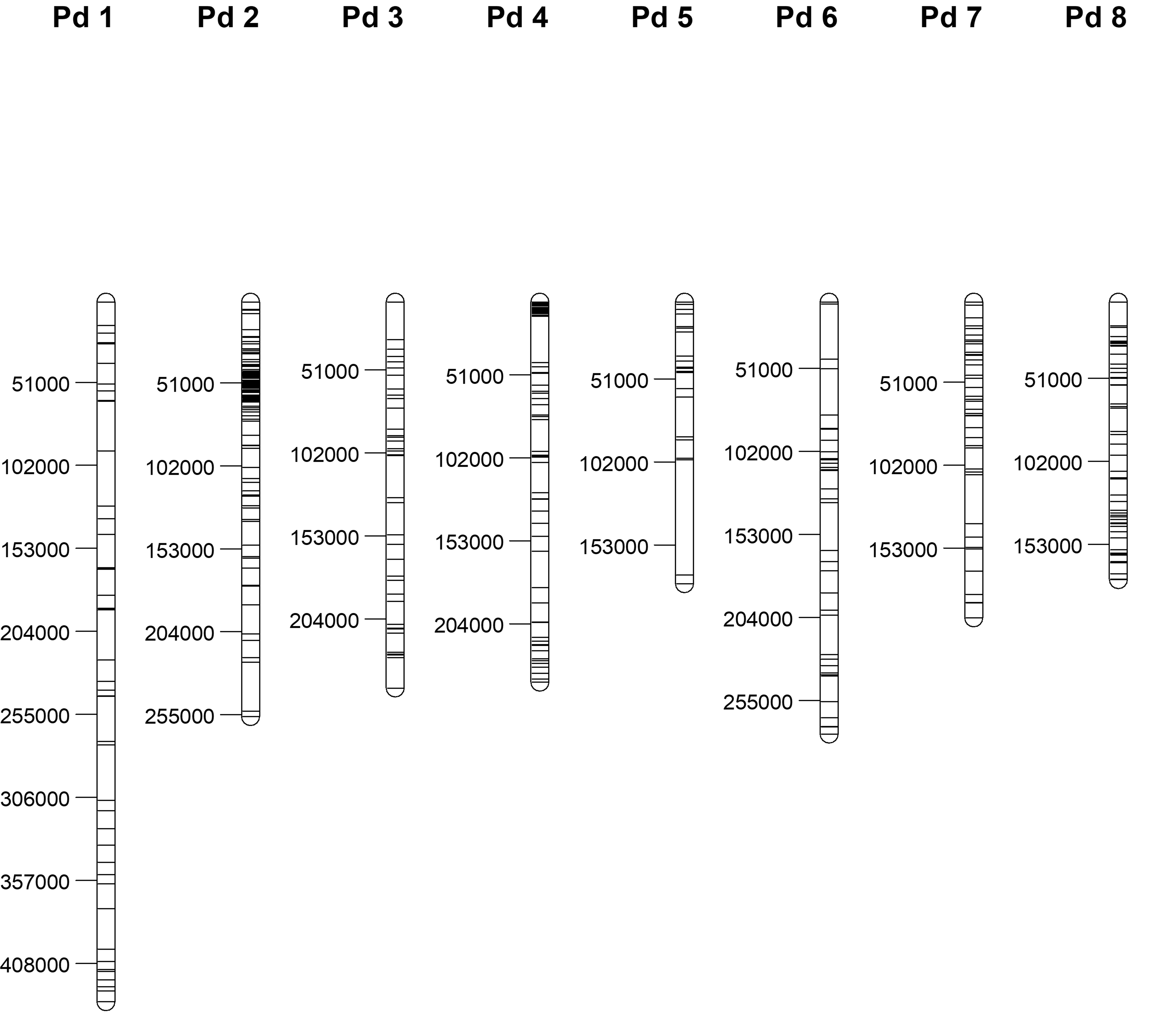

Supplement: Supplementary file 9 — Supplementary figure 4 [file 41438_2021_465_MOESM9_ESM.tif]

$$\text{DeltaK} = \text{mean}(|L''(K)|) / \text{sd}(L(K))$$

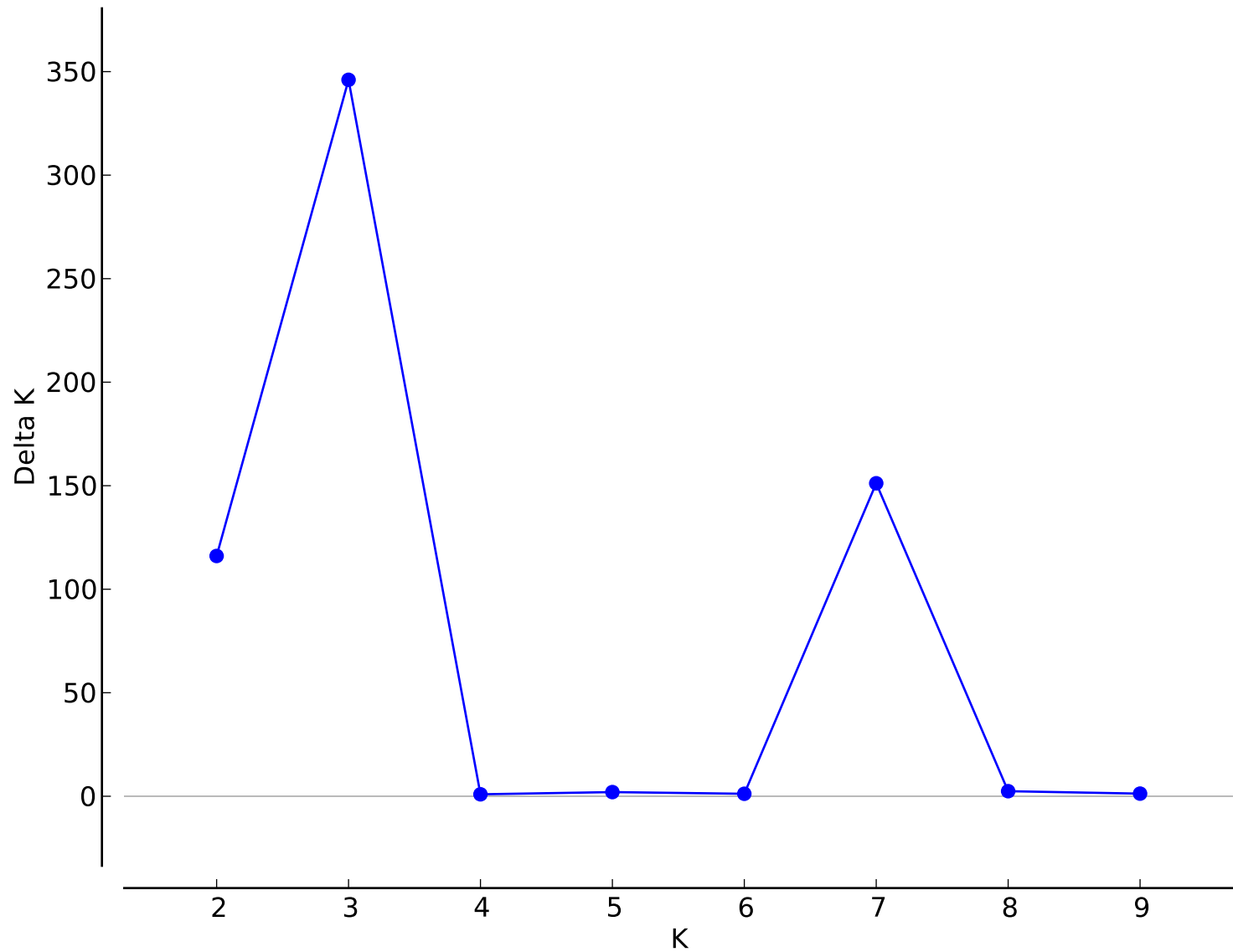

Supplement: Supplementary file 10 — Supplementary figure 5 [file 41438_2021_465_MOESM10_ESM.pdf]

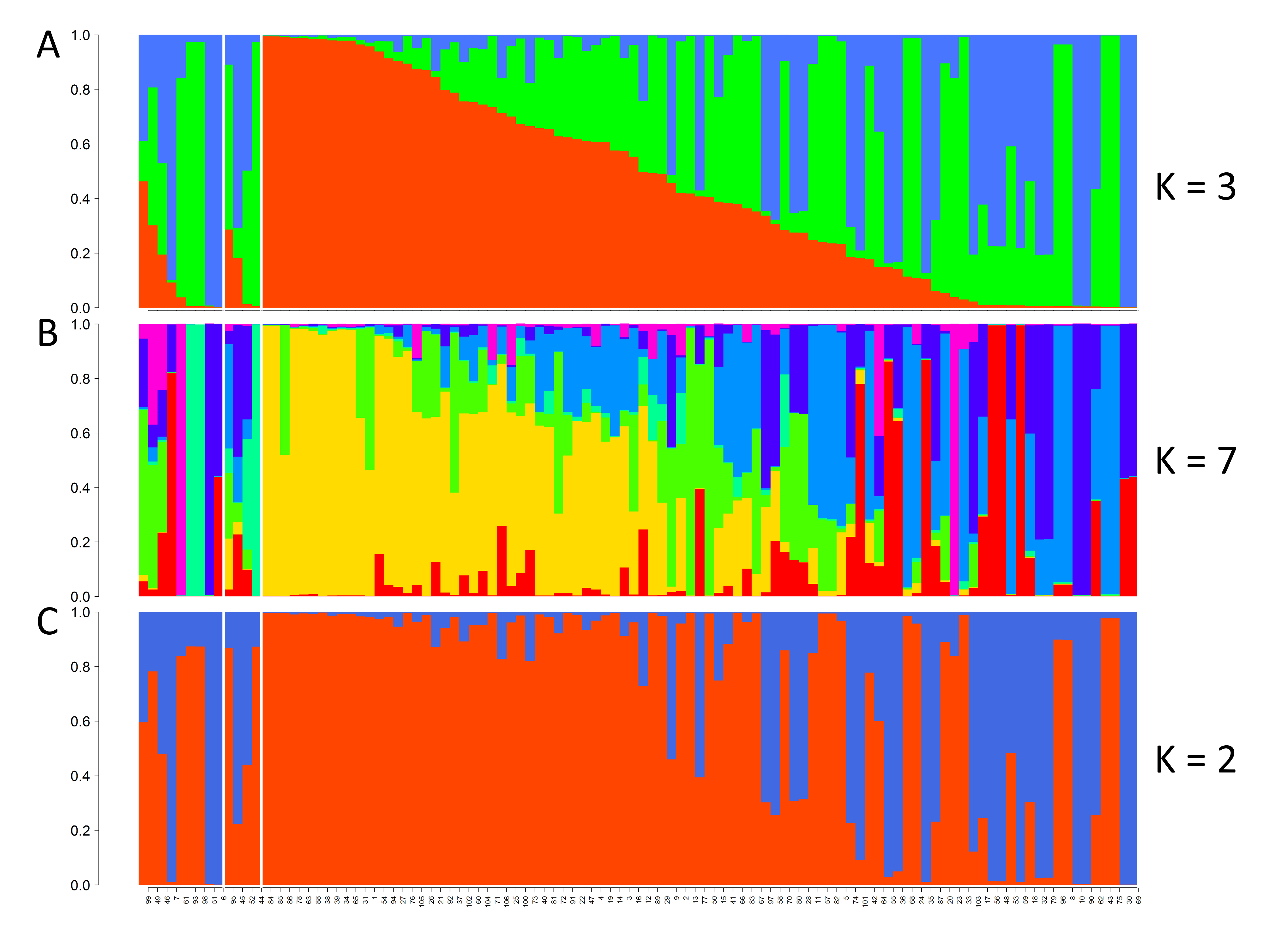

Supplement: Supplementary file 11 — Supplementary figure 6 [file 41438_2021_465_MOESM11_ESM.tif]
