# Supplementary material for: Genetic characterization of an almond germplasm collection and volatilome profiling of raw and roasted kernels
Source: Hortic Res. 2021 Feb 1;8:27. doi: 10.1038/s41438-021-00465-7 (PMC7848010; doi:10.1038/s41438-021-00465-7)

Example #1

QualySort 1.1 - Almond VOCs

Calculate

Select All

Choose type

- [ ] roasted
- [x] raw

Select All

Choose Origin

- [x] Sicily
- [x] International
- [x] Apulia

Select All

Choose parameters

- [ ] Shell texture
- [ ] Production entity
- [ ] Fruit weight
- [ ] % of double seeds
- [ ] Fruit flavor
- [ ] Kernel weight
- [ ] Kernel shape
- [ ] Kernel thickness
- [ ] 26.016 C2H2+ common fragment
- [ ] 28.019 C2H4+ common fragment
- [ ] 31.018 CH2OH+
- [ ] 33.033 CH4OH+ Methanol
- [ ] 34.996 H2SH+ Hydrogen sulfide
- [ ] 39.023 C3H3+ common fragment
- [ ] 41.039 C3H5+ common fragment
- [ ] 42.012
- [ ] 42.022
- [ ] 43.018 C2H3O+ common fragment
- [x] 43.03 CH2N2H+ Cyanamide
- [ ] 43.055 C3H7+ common fragment
- [ ] 44.025
- [x] 45.033 C2H4OH+ Acetaldehyde
- [ ] 47.049 C2H6OH+ Ethanol
- [ ] 49.011 CH4SH+ Methanethiol
- [ ] 53.004
- [ ] 53.04 C4H5+ common fragment
- [ ] 53.048
- [ ] 55.054 C4H7+ Butanal, common fragment
- [ ] 56.026
- [ ] 57.035 C3H4OH+ common fragment
- [ ] 57.043 C2H4N2H+ Aminoacetonitrile
- [ ] 57.07 C4H9+ 1-Butanol
- [ ] 59.049 C3H6OH+ Acetone
- [ ] 61.028 C2H4O2H+ Acetic Acid, fragment of esters
- [ ] 61.055 C3H8OH+
- [ ] 63.012 CO2H3O+ water cluster of Carbon dioxide
- [ ] 63.029 C2H6SH+ Dimethyl sulfide
- [ ] 63.043 C2H6O2H+ water cluster of Acetaldehyde
- [ ] 65.944
- [ ] 67.032 C3H2N2H+ Propanedinitrile
- [ ] 67.057 C5H7+ Pentanal, common fragment
- [ ] 67.992
- [ ] 69.003
- [ ] 69.033 C4H4OH+ Furan
- [ ] 69.056
- [ ] 69.071 C5H9+ Isoprene, common fragment
- [ ] 71.051 C4H6OH+ Butenal
- [x] 71.086 C5H11+ 2-Pentanol, 2-Methyl-1-butanol+3-Methyl-1-butanol, Pentanol
- [ ] 72.962
- [ ] 73.029 C3H4O2H+ Propiolactone, Propenoic acid
- [ ] 73.051
- [x] 73.064 C4H8OH+ 2-Methyl-Propanal
- [ ] 75.035
- [ ] 75.045 C3H6O2H+ 1-Hydroxy-2-Propanone
- [ ] 75.072
- [ ] 76.954 CS2H+ Carbon disulfide
- [ ] 77.008
- [ ] 77.037
- [ ] 79.04 C2H6O3H+ cluster of ms61.028
- [ ] 79.06 C6H7+ Benzene, aromatic ring fragment
- [ ] 79.078
- [ ] 80.06
- [ ] 81.041 C4H4N2H+ Pyrazine
- [ ] 81.07 C6H9+
- [ ] 83.051 C5H6OH+ Methylfuran
- [ ] 83.076
- [ ] 83.087 C6H11+ Hexenol, Hexenal
- [ ] 84.087
- [ ] 85.03
- [ ] 85.067 C5H8OH+ Pentanal, Pentanone
- [x] 85.102 C6H13+ Hexanol
- [ ] 86.009
- [x] 87.045 C4H6O2H+ gamma Butyrolactone
- [x] 87.081 C5H10OH+ 2-Methyl Butanal, 3-Methyl Butanal, 2-Pentanone
- [ ] 89.061 C4H8O2H+ Ethyl Acetate
- [ ] 91.057 C4H10SH+ Diethyl sulfide
- [ ] 91.075 C4H10O2H+ Butanediol
- [ ] 93.04
- [ ] 93.073 C7H9+ Toluene
- [ ] 93.091
- [ ] 95.051 C6H6OH+ Phenol
- [ ] 95.088 C7H11+ Heptenal
- [ ] 97.048 C5H4O2H+ Furfural
- [ ] 97.066 C6H8OH+ Ethylfuran
- [ ] 97.102 C7H13+ Heptanal
- [ ] 99.046 C5H6O2H+ 2-Furan Methanol
- [ ] 99.082 C6H10OH+ Hexenal
- [ ] 99.117 C7H15+ Heptanol
- [ ] 99.951
- [ ] 101.062 C5H8O2H+ 2,3-Pentanedione
- [x] 101.097 C6H12OH+ Hexanal
- [ ] 103.052
- [ ] 103.077 C5H10O2H+ C5 esters and acids
- [ ] 103.115
- [ ] 105.039 C4H8OSH+ Methional
- [x] 105.068 C8H9+ Phenyl Ethyl Alcohol
- [ ] 105.09 C5H12O2H+
- [x] 107.044 C7H6OH+ Benzaldehyde
- [x] 107.089 C8H11+ Ethyl Benzene, p-Xylene, m-Xylene, o-Xylene
- [ ] 109.065 C7H8OH+ Benzyl alcohol, Cresol
- [ ] 109.103 C8H13+ Octenal
- [ ] 111.047 C6H6O2H+
- [ ] 111.084
- [ ] 111.118 C8H15+ 1-Octen-3-ol
- [ ] 113.064 C6H8O2H+ 2(5H)-Furanone, 5,5-dimethyl-
- [ ] 113.099 C7H12OH+ Heptanal, Heptanone
- [ ] 113.133 C8H17+ 2-Ethyl-1-Hexanol, Octanol
- [ ] 115.078 C6H10O2H+ Caprolactone
- [x] 115.114 C7H14OH+ 2-Heptanal (E o Z)
- [ ] 117.062 C9H9+
- [ ] 117.092 C6H12O2H+ Butanoic Acid Ethyl Ester, Butyl Acetate, Hexanoic Acid
- [ ] 117.958
- [ ] 119.106 C6H14O2H+
- [ ] 121.067 C8H8OH+ Benzeneacetaldehyde
- [ ] 121.104 C9H13+
- [ ] 121.12
- [ ] 123.048
- [ ] 123.118 C9H15+ Nonenal
- [ ] 125.059 C7H8O2H+ Benzyl Alcohol
- [ ] 125.1 C8H12OH+ Octadienone
- [ ] 125.134 C9H17+ Nonanal, Nonenol
- [ ] 127.042 C6H6O3H+ Maltol
- [ ] 127.075 C7H10O2H+
- [ ] 127.114 C8H14OH+ 6-Methyl-5-Hepten-2-one
- [ ] 127.148 C9H19+ Nonanol
- [ ] 129.056 C6H8O3H+ Furaneol
- [ ] 129.094 C7H12O2H+
- [ ] 129.129 C8H16OH+ Octanal
- [ ] 131.108 C7H14O2H+ Isoamyl acetate
- [ ] 133.121 C7H16O2H+
- [ ] 134.973
- [ ] 135.12 C10H15+ Cymene
- [ ] 136.993
- [x] 137.134 C10H17+ Limonene
- [ ] 139.114 C9H14OH+ 2-Pentyl Furan
- [ ] 141.13 C9H16OH+ Nonenal, Nonenone
- [ ] 143.11 C8H14O2H+
- [ ] 143.145 C9H18OH+ Nonanal
- [ ] 145.123 C8H16O2H+ Hexyl Acetate
- [ ] 147.137 C8H18O2H+
- [ ] 149.059 C9H8O2H+ Cinnamic acid
- [ ] 149.119 C7H16O3H+
- [ ] 153.092 C9H12O2H+
- [ ] 153.131 C10H16OH+
- [ ] 155.003
- [ ] 155.178 C11H23+ Undecanol
- [ ] 157.123 C9H16O2H+ Whiskey lactone
- [ ] 157.161 C10H20OH+ Decanal
- [ ] 159.139 C9H18O2H+ C9 esters and acids
- [ ] 161.151 C9H20O2H+
- [ ] 163.132 C8H18O3H+
- [ ] 165.079
- [ ] 167.056
- [ ] 169.194 C12H25+ Dodecanol
- [ ] 189.175 C11H24O2H+
- [ ] 197.086
- [ ] 205.197 C15H25+ Sesquiterpenes
- [ ] 223.066

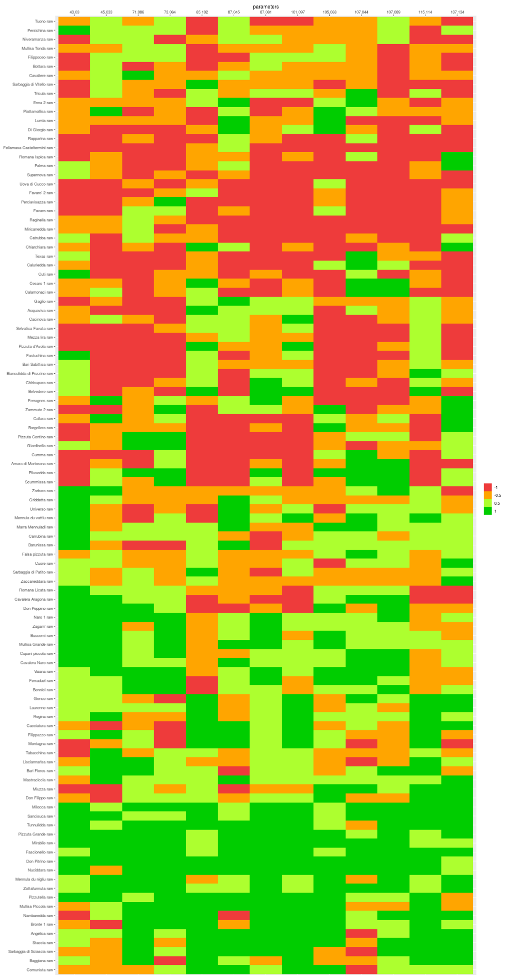

Example #2

QualySort 1.1 - Almond VOCs

Calculate

Select All

Choose type

- [x] roasted
- [ ] raw

Select All

Choose Origin

- [x] Sicily
- [x] International
- [x] Apulia

Select All

Choose parameters

- [ ] Shell texture
- [ ] Production entity
- [ ] Fruit weight
- [ ] % of double seeds
- [ ] Fruit flavor
- [ ] Kernel weight
- [ ] Kernel shape
- [ ] Kernel thickness
- [ ] 26.016 C2H2+ common fragment
- [ ] 28.019 C2H4+ common fragment
- [ ] 31.018 CH2OH+
- [ ] 33.033 CH4OH+ Methanol
- [ ] 34.996 H2SH+ Hydrogen sulfide
- [ ] 39.023 C3H3+ common fragment
- [ ] 41.039 C3H5+ common fragment
- [ ] 42.012
- [ ] 42.022
- [ ] 43.018 C2H3O+ common fragment
- [x] 43.03 CH2N2H+ Cyanamide
- [ ] 43.055 C3H7+ common fragment
- [ ] 44.025
- [x] 45.033 C2H4OH+ Acetaldehyde
- [ ] 47.049 C2H6OH+ Ethanol
- [ ] 49.011 CH4SH+ Methanethiol
- [ ] 53.004
- [ ] 53.04 C4H5+ common fragment
- [ ] 53.048
- [ ] 55.054 C4H7+ Butanal, common fragment
- [ ] 56.026
- [ ] 57.035 C3H4OH+ common fragment
- [ ] 57.043 C2H4N2H+ Aminoacetonitrile
- [ ] 57.07 C4H9+ 1-Butanol
- [ ] 59.049 C3H6OH+ Acetone
- [ ] 61.028 C2H4O2H+ Acetic Acid, fragment of esters
- [ ] 61.055 C3H8OH+
- [ ] 63.012 CO2H3O+ water cluster of Carbon dioxide
- [ ] 63.029 C2H6SH+ Dimethyl sulfide
- [ ] 63.043 C2H6O2H+ water cluster of Acetaldehyde
- [ ] 65.944
- [ ] 67.032 C3H2N2H+ Propanedinitrile
- [ ] 67.057 C5H7+ Pentanal, common fragment
- [ ] 67.992
- [ ] 69.003
- [ ] 69.033 C4H4OH+ Furan
- [ ] 69.056
- [ ] 69.071 C5H9+ Isoprene, common fragment
- [ ] 71.051 C4H6OH+ Butenal
- [x] 71.086 C5H11+ 2-Pentanol, 2-Methyl-1-butanol+3-Methyl-1-butanol, Pentanol
- [ ] 72.962
- [ ] 73.029 C3H4O2H+ Propiolactone, Propenoic acid
- [ ] 73.051
- [x] 73.064 C4H8OH+ 2-Methyl-Propanal
- [ ] 75.035
- [ ] 75.045 C3H6O2H+ 1-Hydroxy-2-Propanone
- [ ] 75.072
- [ ] 76.954 CS2H+ Carbon disulfide
- [ ] 77.008
- [ ] 77.037
- [ ] 79.04 C2H6O3H+ cluster of ms61.028
- [ ] 79.06 C6H7+ Benzene, aromatic ring fragment
- [ ] 79.078
- [ ] 80.06
- [ ] 81.041 C4H4N2H+ Pyrazine
- [ ] 81.07 C6H9+
- [ ] 83.051 C5H6OH+ Methylfuran
- [ ] 83.076
- [ ] 83.087 C6H11+ Hexenol, Hexanal
- [ ] 84.087
- [ ] 85.03
- [ ] 85.067 C5H8OH+ Pentanal, Pentanone
- [x] 85.102 C6H13+ Hexanol
- [ ] 86.009
- [x] 87.045 C4H6O2H+ gamma Butyrolactone
- [x] 87.081 C5H10OH+ 2-Methyl Butanal, 3-Methyl Butanal, 2-Pentanone
- [ ] 89.061 C4H8O2H+ Ethyl Acetate
- [ ] 91.057 C4H10SH+ Diethyl sulfide
- [ ] 91.075 C4H10O2H+ Butanediol
- [ ] 93.04
- [ ] 93.073 C7H9+ Toluene
- [ ] 93.091
- [ ] 95.051 C6H6OH+ Phenol
- [ ] 95.088 C7H11+ Heptenal
- [ ] 97.048 C5H4O2H+ Furfural
- [ ] 97.066 C6H8OH+ Ethylfuran
- [ ] 97.102 C7H13+ Heptanal
- [ ] 99.046 C5H6O2H+ 2-Furan Methanol
- [ ] 99.082 C6H10OH+ Hexenal
- [ ] 99.117 C7H15+ Heptanol
- [ ] 99.951
- [ ] 101.062 C5H8O2H+ 2,3-Pentanedione
- [x] 101.097 C6H12OH+ Hexanal
- [ ] 103.052
- [ ] 103.077 C5H10O2H+ C5 esters and acids
- [ ] 103.115
- [ ] 105.039 C4H8OSH+ Methional
- [x] 105.068 C8H9+ Phenyl Ethyl Alcohol
- [ ] 105.09 C5H12O2H+
- [x] 107.044 C7H6OH+ Benzaldehyde
- [x] 107.089 C8H11+ Ethyl Benzene, p-Xylene, m-Xylene, o-Xylene
- [ ] 109.065 C7H8OH+ Benzyl alcohol, Cresol
- [ ] 109.103 C8H13+ Octenal
- [ ] 111.047 C6H6O2H+
- [ ] 111.084
- [ ] 111.118 C8H15+ 1-Octen-3-ol
- [ ] 113.064 C6H8O2H+ 2(5H)-Furanone, 5,5-dimethyl-
- [ ] 113.099 C7H12OH+ Heptanal, Heptanone
- [ ] 113.133 C8H17+ 2-Ethyl-1-Hexanol, Octanol
- [ ] 115.078 C6H10O2H+ Caprolactone
- [x] 115.114 C7H14OH+ 2-Heptanal (E o Z)
- [ ] 117.062 C9H9+
- [ ] 117.092 C6H12O2H+ Butanoic Acid Ethyl Ester, Butyl Acetate, Hexanoic Acid
- [ ] 117.958
- [ ] 119.106 C6H14O2H+
- [ ] 121.067 C8H8OH+ Benzeneacetaldehyde
- [ ] 121.104 C9H13+
- [ ] 121.12
- [ ] 123.048
- [ ] 123.118 C9H15+ Nonenal
- [ ] 125.059 C7H8O2H+ Benzyl Alcohol
- [ ] 125.1 C8H12OH+ Octadienone
- [ ] 125.134 C9H17+ Nonanal, Nonenol
- [ ] 127.042 C6H6O3H+ Maltol
- [ ] 127.075 C7H10O2H+
- [ ] 127.114 C8H14OH+ 6-Methyl-5-Hepten-2-one
- [ ] 127.148 C9H19+ Nonanol
- [ ] 129.056 C6H8O3H+ Furaneol
- [ ] 129.094 C7H12O2H+
- [ ] 129.129 C8H16OH+ Octanal
- [ ] 131.108 C7H14O2H+ Isoamyl acetate
- [ ] 133.121 C7H16O2H+
- [ ] 134.973
- [ ] 135.12 C10H15+ Cymene
- [ ] 136.993
- [x] 137.134 C10H17+ Limonene
- [ ] 139.114 C9H14OH+ 2-Pentyl Furan
- [ ] 141.13 C9H16OH+ Nonenal, Nonenone
- [ ] 143.11 C8H14O2H+
- [ ] 143.145 C9H18OH+ Nonanal
- [ ] 145.123 C8H16O2H+ Hexyl Acetate
- [ ] 147.137 C8H18O2H+
- [ ] 149.059 C9H8O2H+ Cinnamic acid
- [ ] 149.119 C7H16O3H+
- [ ] 153.092 C9H12O2H+
- [ ] 153.131 C10H16OH+
- [ ] 155.003
- [ ] 155.178 C11H23+ Undecanol
- [ ] 157.123 C9H16O2H+ Whiskey lactone
- [ ] 157.161 C10H20OH+ Decanal
- [ ] 159.139 C9H18O2H+ C9 esters and acids
- [ ] 161.151 C9H20O2H+
- [ ] 163.132 C8H18O3H+
- [ ] 165.079
- [ ] 167.056
- [ ] 169.194 C12H25+ Dodecanol
- [ ] 189.175 C11H24O2H+
- [ ] 197.086
- [ ] 205.197 C15H25+ Sesquiterpenes
- [ ] 223.066

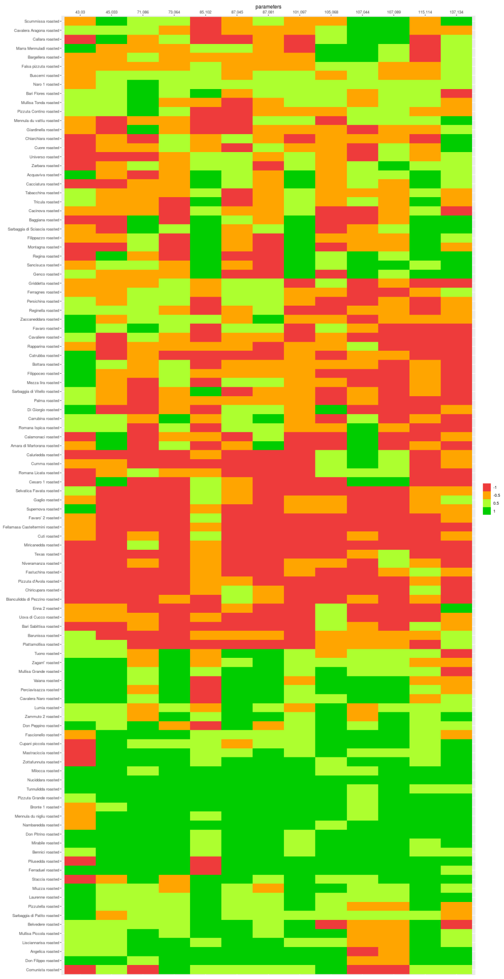

Example #3

QualySort 1.1 - Almond VOCs

Calculate

Select All

Choose type

- [ ] roasted
- [x] raw

Select All

Choose Origin

- [x] Sicily
- [x] International
- [x] Apulia

Select All (https://iuliiakhomenko-fmach.shinyapps.io/QualySort/#)

Choose parameters

- [ ] Shell texture
- [ ] Production entity
- [ ] Fruit weight
- [ ] % of double seeds
- [ ] Fruit flavor
- [ ] Kernel weight
- [ ] Kernel shape
- [ ] Kernel thickness
- [ ] 26.016 C2H2+ common fragment
- [ ] 28.019 C2H4+ common fragment
- [ ] 31.018 CH2OH+
- [ ] 33.033 CH4OH+ Methanol
- [ ] 34.996 H2SH+ Hydrogen sulfide
- [ ] 39.023 C3H3+ common fragment
- [ ] 41.039 C3H5+ common fragment
- [ ] 42.012
- [ ] 42.022
- [ ] 43.018 C2H3O+ common fragment
- [ ] 43.03 CH2N2H+ Cyanamide
- [ ] 43.055 C3H7+ common fragment
- [ ] 44.025
- [ ] 45.033 C2H4OH+ Acetaldehyde
- [ ] 47.049 C2H6OH+ Ethanol
- [x] 49.011 CH4SH+ Methanethiol
- [ ] 53.004
- [ ] 53.04 C4H5+ common fragment
- [ ] 53.048
- [ ] 55.054 C4H7+ Butanal, common fragment
- [ ] 56.026
- [ ] 57.035 C3H4OH+ common fragment
- [ ] 57.043 C2H4N2H+ Aminoacetonitrile
- [ ] 57.07 C4H9+ 1-Butanol
- [ ] 59.049 C3H6OH+ Acetone
- [ ] 61.028 C2H4O2H+ Acetic Acid, fragment of esters
- [ ] 61.055 C3H8OH+
- [ ] 63.012 CO2H3O+ water cluster of Carbon dioxide
- [x] 63.029 C2H6SH+ Dimethyl sulfide
- [ ] 63.043 C2H6O2H+ water cluster of Acetaldehyde
- [ ] 65.944
- [ ] 67.032 C3H2N2H+ Propanedinitrile
- [ ] 67.057 C5H7+ Pentanal, common fragment
- [ ] 67.992
- [ ] 69.003
- [ ] 69.033 C4H4OH+ Furan
- [ ] 69.056
- [ ] 69.071 C5H9+ Isoprene, common fragment
- [ ] 71.051 C4H6OH+ Butenal
- [ ] 71.086 C5H11+ 2-Pentanol, 2-Methyl-1-butanol+3-Methyl-1-butanol, Pentanol
- [ ] 72.962
- [ ] 73.029 C3H4O2H+ Propiolactone, Propenoic acid
- [ ] 73.051
- [ ] 73.064 C4H8OH+ 2-Methyl-Propanal
- [ ] 75.035
- [ ] 75.045 C3H6O2H+ 1-Hydroxy-2-Propanone
- [ ] 75.072
- [ ] 76.954 CS2H+ Carbon disulfide
- [ ] 77.008
- [ ] 77.037
- [ ] 79.04 C2H6O3H+ cluster of ms61.028
- [ ] 79.06 C6H7+ Benzene, aromatic ring fragment
- [ ] 79.078
- [ ] 80.06
- [ ] 81.041 C4H4N2H+ Pyrazine
- [ ] 81.07 C6H9+
- [ ] 83.051 C5H6OH+ Methylfuran
- [ ] 83.076
- [ ] 83.087 C6H11+ Hexenol, Hexenal
- [ ] 84.087
- [ ] 85.03
- [ ] 85.067 C5H8OH+ Pentanal, Pentanone
- [ ] 85.102 C6H13+ Hexanol
- [ ] 86.009
- [ ] 87.045 C4H6O2H+ gamma Butyrolactone
- [ ] 87.081 C5H10OH+ 2-Methyl Butanal, 3-Methyl Butanal, 2-Pentanone
- [ ] 89.061 C4H8O2H+ Ethyl Acetate
- [x] 91.057 C4H10SH+ Diethyl sulfide
- [ ] 91.075 C4H10O2H+ Butanediol
- [ ] 93.04
- [ ] 93.073 C7H9+ Toluene
- [ ] 93.091
- [ ] 95.051 C6H6OH+ Phenol
- [ ] 95.088 C7H11+ Heptenal
- [ ] 97.048 C5H4O2H+ Furfural
- [ ] 97.066 C6H8OH+ Ethylfuran
- [ ] 97.102 C7H13+ Heptanal
- [ ] 99.046 C5H6O2H+ 2-Furan Methanol
- [ ] 99.082 C6H10OH+ Hexenal
- [ ] 99.117 C7H15+ Heptanol
- [ ] 99.951
- [ ] 101.062 C5H8O2H+ 2,3-Pentanedione
- [ ] 101.097 C6H12OH+ Hexanal
- [ ] 103.052
- [ ] 103.077 C5H10O2H+ C5 esters and acids
- [ ] 103.115
- [x] 105.039 C4H8OSH+ Methional
- [ ] 105.068 C8H9+ Phenyl Ethyl Alcohol
- [ ] 105.09 C5H12O2H+
- [ ] 107.044 C7H6OH+ Benzaldehyde
- [ ] 107.089 C8H11+ Ethyl Benzene, p-Xylene, m-Xylene, o-Xylene
- [ ] 109.065 C7H8OH+ Benzyl alcohol, Cresol
- [ ] 109.103 C8H13+ Octenal
- [ ] 111.047 C6H6O2H+
- [ ] 111.084
- [ ] 111.118 C8H15+ 1-Octen-3-ol
- [ ] 113.064 C6H8O2H+ 2(5H)-Furanone, 5,5-dimethyl-
- [ ] 113.099 C7H12OH+ Heptanal, Heptanone
- [ ] 113.133 C8H17+ 2-Ethyl-1-Hexanol, Octanol
- [ ] 115.078 C6H10O2H+ Caprolactone
- [ ] 115.114 C7H14OH+ 2-Heptanal (E o Z)
- [ ] 117.062 C9H9+
- [ ] 117.092 C6H12O2H+ Butanoic Acid Ethyl Ester, Butyl Acetate, Hexanoic Acid
- [ ] 117.958
- [ ] 119.106 C6H14O2H+
- [ ] 121.067 C8H8OH+ Benzeneacetaldehyde
- [ ] 121.104 C9H13+
- [ ] 121.12
- [ ] 123.048
- [ ] 123.118 C9H15+ Nonenal
- [ ] 125.059 C7H8O2H+ Benzyl Alcohol
- [ ] 125.1 C8H12OH+ Octadienone
- [ ] 125.134 C9H17+ Nonanal, Nonenol
- [ ] 127.042 C6H6O3H+ Maltol
- [ ] 127.075 C7H10O2H+
- [ ] 127.114 C8H14OH+ 6-Methyl-5-Hepten-2-one
- [ ] 127.148 C9H19+ Nonanol
- [ ] 129.056 C6H8O3H+ Furaneol
- [ ] 129.094 C7H12O2H+
- [ ] 129.129 C8H16OH+ Octanal
- [ ] 131.108 C7H14O2H+ Isoamyl acetate
- [ ] 133.121 C7H16O2H+
- [ ] 134.973
- [ ] 135.12 C10H15+ Cymene
- [ ] 136.993
- [ ] 137.134 C10H17+ Limonene
- [ ] 139.114 C9H14OH+ 2-Pentyl Furan
- [ ] 141.13 C9H16OH+ Nonenal, Nonenone
- [ ] 143.11 C8H14O2H+
- [ ] 143.145 C9H18OH+ Nonanal
- [ ] 145.123 C8H16O2H+ Hexyl Acetate
- [ ] 147.137 C8H18O2H+
- [ ] 149.059 C9H8O2H+ Cinnamic acid
- [ ] 149.119 C7H16O3H+
- [ ] 153.092 C9H12O2H+
- [ ] 153.131 C10H16OH+
- [ ] 155.003
- [ ] 155.178 C11H23+ Undecanol
- [ ] 157.123 C9H16O2H+ Whiskey lactone
- [ ] 157.161 C10H20OH+ Decanal
- [ ] 159.139 C9H18O2H+ C9 esters and acids
- [ ] 161.151 C9H20O2H+
- [ ] 163.132 C8H18O3H+
- [ ] 165.079
- [ ] 167.056
- [ ] 169.194 C12H25+ Dodecanol
- [ ] 189.175 C11H24O2H+
- [ ] 197.086
- [ ] 205.197 C15H25+ Sesquiterpenes
- [ ] 223.066

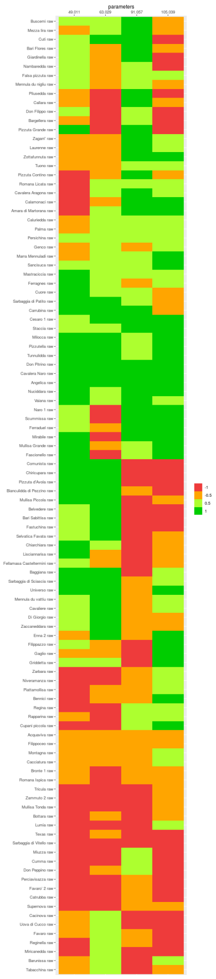

Example #4

QualySort 1.1 - Almond VOCs

Calculate

Select All

Choose type

- [x] roasted
- [ ] raw

Select All

Choose Origin

- [x] Sicily
- [x] International
- [x] Apulia

Select All (https://iuliiakhomenko-fmach.shinyapps.io/QualySort/#)

Choose parameters

- [ ] Shell texture
- [ ] Production entity
- [ ] Fruit weight
- [ ] % of double seeds
- [ ] Fruit flavor
- [ ] Kernel weight
- [ ] Kernel shape
- [ ] Kernel thickness
- [ ] 26.016 C2H2+ common fragment
- [ ] 28.019 C2H4+ common fragment
- [ ] 31.018 CH2OH+
- [ ] 33.033 CH4OH+ Methanol
- [x] 34.996 H2SH+ Hydrogen sulfide
- [ ] 39.023 C3H3+ common fragment
- [ ] 41.039 C3H5+ common fragment
- [ ] 42.012
- [ ] 42.022
- [ ] 43.018 C2H3O+ common fragment
- [ ] 43.03 CH2N2H+ Cyanamide
- [ ] 43.055 C3H7+ common fragment
- [ ] 44.025
- [ ] 45.033 C2H4OH+ Acetaldehyde
- [ ] 47.049 C2H6OH+ Ethanol
- [x] 49.011 CH4SH+ Methanethiol
- [ ] 53.004
- [ ] 53.04 C4H5+ common fragment
- [ ] 53.048
- [ ] 55.054 C4H7+ Butanal, common fragment
- [ ] 56.026
- [ ] 57.035 C3H4OH+ common fragment
- [ ] 57.043 C2H4N2H+ Aminoacetonitrile
- [ ] 57.07 C4H9+ 1-Butanol
- [ ] 59.049 C3H6OH+ Acetone
- [ ] 61.028 C2H4O2H+ Acetic Acid, fragment of esters
- [ ] 61.055 C3H8OH+
- [ ] 63.012 CO2H3O+ water cluster of Carbon dioxide
- [x] 63.029 C2H6SH+ Dimethyl sulfide
- [ ] 63.043 C2H6O2H+ water cluster of Acetaldehyde
- [ ] 65.944
- [ ] 67.032 C3H2N2H+ Propanedinitrile
- [ ] 67.057 C5H7+ Pentanal, common fragment
- [ ] 67.992
- [ ] 69.003
- [ ] 69.033 C4H4OH+ Furan
- [ ] 69.056
- [ ] 69.071 C5H9+ Isoprene, common fragment
- [ ] 71.051 C4H6OH+ Butenal
- [ ] 71.086 C5H11+ 2-Pentanol, 2-Methyl-1-butanol+3-Methyl-1-butanol, Pentanol
- [ ] 72.962
- [ ] 73.029 C3H4O2H+ Propiolactone, Propenoic acid
- [ ] 73.051
- [ ] 73.064 C4H8OH+ 2-Methyl-Propanal
- [ ] 75.035
- [ ] 75.045 C3H6O2H+ 1-Hydroxy-2-Propanone
- [ ] 75.072
- [ ] 76.954 CS2H+ Carbon disulfide
- [ ] 77.008
- [ ] 77.037
- [ ] 79.04 C2H6O3H+ cluster of ms61.028
- [ ] 79.06 C6H7+ Benzene, aromatic ring fragment
- [ ] 79.078
- [ ] 80.06
- [ ] 81.041 C4H4N2H+ Pyrazine
- [ ] 81.07 C6H9+
- [ ] 83.051 C5H6OH+ Methylfuran
- [ ] 83.076
- [ ] 83.087 C6H11+ Hexenol, Hexenal
- [ ] 84.087
- [ ] 85.03
- [ ] 85.067 C5H8OH+ Pentanal, Pentanone
- [ ] 85.102 C6H13+ Hexanol
- [ ] 86.009
- [ ] 87.045 C4H6O2H+ gamma Butyrolactone
- [ ] 87.081 C5H10OH+ 2-Methyl Butanal, 3-Methyl Butanal, 2-Pentanone
- [ ] 89.061 C4H8O2H+ Ethyl Acetate
- [x] 91.057 C4H10SH+ Diethyl sulfide
- [ ] 91.075 C4H10O2H+ Butanediol
- [ ] 93.04
- [ ] 93.073 C7H9+ Toluene
- [ ] 93.091
- [ ] 95.051 C6H6OH+ Phenol
- [ ] 95.088 C7H11+ Heptenal
- [ ] 97.048 C5H4O2H+ Furfural
- [ ] 97.066 C6H8OH+ Ethylfuran
- [ ] 97.102 C7H13+ Heptenal
- [ ] 99.046 C5H6O2H+ 2-Furan Methanol
- [ ] 99.082 C6H10OH+ Hexenal
- [ ] 99.117 C7H15+ Heptanol
- [ ] 99.951
- [ ] 101.062 C5H8O2H+ 2,3-Pentanedione
- [ ] 101.097 C6H12OH+ Hexanal
- [ ] 103.052
- [ ] 103.077 C5H10O2H+ C5 esters and acids
- [ ] 103.115
- [x] 105.039 C4H8OSH+ Methional
- [ ] 105.068 C8H9+ Phenyl Ethyl Alcohol
- [ ] 105.09 C5H12O2H+
- [ ] 107.044 C7H6OH+ Benzaldehyde
- [ ] 107.089 C8H11+ Ethyl Benzene, p-Xylene, m-Xylene, o-Xylene
- [ ] 109.065 C7H8OH+ Benzyl alcohol, Cresol
- [ ] 109.103 C8H13+ Octenal
- [ ] 111.047 C6H6O2H+
- [ ] 111.084
- [ ] 111.118 C8H15+ 1-Octen-3-ol
- [ ] 113.064 C6H8O2H+ 2(5H)-Furanone, 5,5-dimethyl-
- [ ] 113.099 C7H12OH+ Heptanal, Heptanone
- [ ] 113.133 C8H17+ 2-Ethyl-1-Hexanol, Octanol
- [ ] 115.078 C6H10O2H+ Caprolactone
- [ ] 115.114 C7H14OH+ 2-Heptanal (E o Z)
- [ ] 117.062 C9H9+
- [ ] 117.092 C6H12O2H+ Butanoic Acid Ethyl Ester, Butyl Acetate, Hexanoic Acid
- [ ] 117.958
- [ ] 119.106 C6H14O2H+
- [ ] 121.067 C8H8OH+ Benzeneacetaldehyde
- [ ] 121.104 C9H13+
- [ ] 121.12
- [ ] 123.048
- [ ] 123.118 C9H15+ Nonenal
- [ ] 125.059 C7H8O2H+ Benzyl Alcohol
- [ ] 125.1 C8H12OH+ Octadienone
- [ ] 125.134 C9H17+ Nonanal, Nonenol
- [ ] 127.042 C6H6O3H+ Maltol
- [ ] 127.075 C7H10O2H+
- [ ] 127.114 C8H14OH+ 6-Methyl-5-Hepten-2-one
- [ ] 127.148 C9H19+ Nonanol
- [ ] 129.056 C6H8O3H+ Furaneol
- [ ] 129.094 C7H12O2H+
- [ ] 129.129 C8H16OH+ Octanal
- [ ] 131.108 C7H14O2H+ Isoamyl acetate
- [ ] 133.121 C7H16O2H+
- [ ] 134.973
- [ ] 135.12 C10H15+ Cymene
- [ ] 136.993
- [ ] 137.134 C10H17+ Limonene
- [ ] 139.114 C9H14OH+ 2-Pentyl Furan
- [ ] 141.13 C9H16OH+ Nonenal, Nonenone
- [ ] 143.11 C8H14O2H+
- [ ] 143.145 C9H18OH+ Nonanal
- [ ] 145.123 C8H16O2H+ Hexyl Acetate
- [ ] 147.137 C8H18O2H+
- [ ] 149.059 C9H8O2H+ Cinnamic acid
- [ ] 149.119 C7H16O3H+
- [ ] 153.092 C9H12O2H+
- [ ] 153.131 C10H16OH+
- [ ] 155.003
- [ ] 155.178 C11H23+ Undecanol
- [ ] 157.123 C9H16O2H+ Whiskey lactone
- [ ] 157.161 C10H20OH+ Decanal
- [ ] 159.139 C9H18O2H+ C9 esters and acids
- [ ] 161.151 C9H20O2H+
- [ ] 163.132 C8H18O3H+
- [ ] 165.079
- [ ] 167.056
- [ ] 169.194 C12H25+ Dodecanol
- [ ] 189.175 C11H24O2H+
- [ ] 197.086
- [ ] 205.197 C15H25+ Sesquiterpenes
- [ ] 223.066

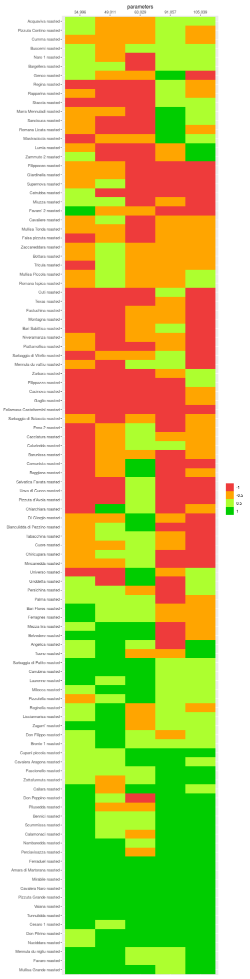

Example #5

QualySort 1.1 - Almond VOCs

Calculate

Select All

Choose type

- [ ] roasted
- [x] raw

Select All

Choose Origin

- [x] Sicily
- [x] International
- [x] Apulia

Select All (https://iuliiakhomenko-fmach.shinyapps.io/QualySort/#)

Choose parameters

- [ ] Shell texture
- [ ] Production entity
- [ ] Fruit weight
- [ ] % of double seeds
- [ ] Fruit flavor
- [ ] Kernel weight
- [ ] Kernel shape
- [ ] Kernel thickness
- [ ] 26.016 C2H2+ common fragment
- [ ] 28.019 C2H4+ common fragment
- [ ] 31.018 CH2OH+
- [ ] 33.033 CH4OH+ Methanol
- [ ] 34.996 H2SH+ Hydrogen sulfide
- [ ] 39.023 C3H3+ common fragment
- [ ] 41.039 C3H5+ common fragment
- [ ] 42.012
- [ ] 42.022
- [ ] 43.018 C2H3O+ common fragment
- [x] 43.03 CH2N2H+ Cyanamide
- [ ] 43.055 C3H7+ common fragment
- [ ] 44.025
- [ ] 45.033 C2H4OH+ Acetaldehyde
- [ ] 47.049 C2H6OH+ Ethanol
- [ ] 49.011 CH4SH+ Methanethiol
- [ ] 53.004
- [ ] 53.04 C4H5+ common fragment
- [ ] 53.048
- [ ] 55.054 C4H7+ Butanal, common fragment
- [ ] 56.026
- [ ] 57.035 C3H4OH+ common fragment
- [ ] 57.043 C2H4N2H+ Aminoacetonitrile
- [ ] 57.07 C4H9+ 1-Butanol
- [ ] 59.049 C3H6OH+ Acetone
- [x] 61.028 C2H4O2H+ Acetic Acid, fragment of esters
- [ ] 61.055 C3H8OH+
- [ ] 63.012 CO2H3O+ water cluster of Carbon dioxide
- [ ] 63.029 C2H6SH+ Dimethyl sulfide
- [ ] 63.043 C2H6O2H+ water cluster of Acetaldehyde
- [ ] 65.944
- [ ] 67.032 C3H2N2H+ Propanedinitrile
- [ ] 67.057 C5H7+ Pentanal, common fragment
- [ ] 67.992
- [ ] 69.003
- [x] 69.033 C4H4OH+ Furan
- [ ] 69.056
- [ ] 69.071 C5H9+ Isoprene, common fragment
- [ ] 71.051 C4H6OH+ Butenal
- [ ] 71.086 C5H11+ 2-Pentanol, 2-Methyl-1-butanol+3-Methyl-1-butanol, Pentanol
- [ ] 72.962
- [ ] 73.029 C3H4O2H+ Propiolactone, Propenoic acid
- [ ] 73.051
- [x] 73.064 C4H8OH+ 2-Methyl-Propanal
- [ ] 75.035
- [x] 75.045 C3H6O2H+ 1-Hydroxy-2-Propanone
- [ ] 75.072
- [ ] 76.954 CS2H+ Carbon disulfide
- [ ] 77.008
- [ ] 77.037
- [ ] 79.04 C2H6O3H+ cluster of ms61.028
- [ ] 79.06 C6H7+ Benzene, aromatic ring fragment
- [ ] 79.078
- [ ] 80.06
- [ ] 81.041 C4H4N2H+ Pyrazine
- [ ] 81.07 C6H9+
- [x] 83.051 C5H6OH+ Methylfuran
- [ ] 83.076
- [ ] 83.087 C6H11+ Hexenol, Hexenal
- [ ] 84.087
- [ ] 85.03
- [ ] 85.067 C5H8OH+ Pentanal, Pentanone
- [ ] 85.102 C6H13+ Hexanol
- [ ] 86.009
- [x] 87.045 C4H6O2H+ gamma Butyrolactone
- [x] 87.081 C5H10OH+ 2-Methyl Butanal, 3-Methyl Butanal, 2-Pentanone
- [ ] 89.061 C4H8O2H+ Ethyl Acetate
- [ ] 91.057 C4H10SH+ Diethyl sulfide
- [ ] 91.075 C4H10O2H+ Butanediol
- [ ] 93.04
- [ ] 93.073 C7H9+ Toluene
- [ ] 93.091
- [ ] 95.051 C6H6OH+ Phenol
- [ ] 95.088 C7H11+ Heptenal
- [x] 97.048 C5H4O2H+ Furfural
- [x] 97.066 C6H8OH+ Ethylfuran
- [ ] 97.102 C7H13+ Heptenal
- [x] 99.046 C5H6O2H+ 2-Furan Methanol
- [ ] 99.082 C6H10OH+ Hexenal
- [ ] 99.117 C7H15+ Heptanol
- [ ] 99.951
- [ ] 101.062 C5H8O2H+ 2,3-Pentanedione
- [ ] 101.097 C6H12OH+ Hexanal
- [ ] 103.052
- [ ] 103.077 C5H10O2H+ C5 esters and acids
- [ ] 103.115
- [ ] 105.039 C4H8OSH+ Methional
- [ ] 105.068 C8H9+ Phenyl Ethyl Alcohol
- [ ] 105.09 C5H12O2H+
- [ ] 107.044 C7H6OH+ Benzaldehyde
- [ ] 107.089 C8H11+ Ethyl Benzene, p-Xylene, m-Xylene, o-Xylene
- [ ] 109.065 C7H8OH+ Benzyl alcohol, Cresol
- [ ] 109.103 C8H13+ Octenal
- [ ] 111.047 C6H6O2H+
- [ ] 111.084
- [ ] 111.118 C8H15+ 1-Octen-3-ol
- [ ] 113.064 C6H8O2H+ 2(5H)-Furanone, 5,5-dimethyl-
- [ ] 113.099 C7H12OH+ Heptanal, Heptanone
- [ ] 113.133 C8H17+ 2-Ethyl-1-Hexanol, Octanol
- [ ] 115.078 C6H10O2H+ Caprolactone
- [ ] 115.114 C7H14OH+ 2-Heptanal (E o Z)
- [ ] 117.062 C9H9+
- [ ] 117.092 C6H12O2H+ Butanoic Acid Ethyl Ester, Butyl Acetate, Hexanoic Acid
- [ ] 117.958
- [ ] 119.106 C6H14O2H+
- [x] 121.067 C8H8OH+ Benzeneacetaldehyde
- [ ] 121.104 C9H13+
- [ ] 121.12
- [ ] 123.048
- [ ] 123.118 C9H15+ Nonenal
- [ ] 125.059 C7H8O2H+ Benzyl Alcohol
- [ ] 125.1 C8H12OH+ Octadienone
- [ ] 125.134 C9H17+ Nonanal, Nonenol
- [ ] 127.042 C6H6O3H+ Maltol
- [ ] 127.075 C7H10O2H+
- [ ] 127.114 C8H14OH+ 6-Methyl-5-Hepten-2-one
- [ ] 127.148 C9H19+ Nonanol
- [ ] 129.056 C6H8O3H+ Furaneol
- [ ] 129.094 C7H12O2H+
- [ ] 129.129 C8H16OH+ Octanal
- [ ] 131.108 C7H14O2H+ Isoamyl acetate
- [ ] 133.121 C7H16O2H+
- [ ] 134.973
- [ ] 135.12 C10H15+ Cymene
- [ ] 136.993
- [ ] 137.134 C10H17+ Limonene
- [x] 139.114 C9H14OH+ 2-Pentyl Furan
- [ ] 141.13 C9H16OH+ Nonenal, Nonenone
- [ ] 143.11 C8H14O2H+
- [ ] 143.145 C9H18OH+ Nonanal
- [ ] 145.123 C8H16O2H+ Hexyl Acetate
- [ ] 147.137 C8H18O2H+
- [ ] 149.059 C9H8O2H+ Cinnamic acid
- [ ] 149.119 C7H16O3H+
- [ ] 153.092 C9H12O2H+
- [ ] 153.131 C10H16OH+
- [ ] 155.003
- [ ] 155.178 C11H23+ Undecanol
- [ ] 157.123 C9H16O2H+ Whiskey lactone
- [ ] 157.161 C10H20OH+ Decanal
- [ ] 159.139 C9H18O2H+ C9 esters and acids
- [ ] 161.151 C9H20O2H+
- [ ] 163.132 C8H18O3H+
- [ ] 165.079
- [ ] 167.056
- [ ] 169.194 C12H25+ Dodecanol
- [ ] 189.175 C11H24O2H+
- [ ] 197.086
- [ ] 205.197 C15H25+ Sesquiterpenes
- [ ] 223.066

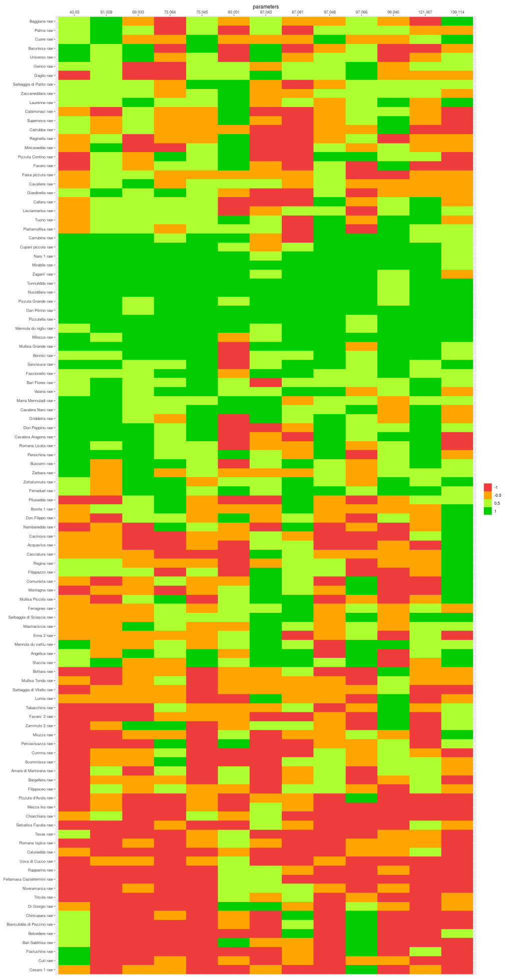

Example #6

QualySort 1.1 - Almond VOCs

Calculate

Select All

Choose type

- [x] roasted
- [ ] raw

Select All

Choose Origin

- [x] Sicily
- [x] International
- [x] Apulia

Select All (https://iuliiakhomenko-fmach.shinyapps.io/QualySort/#)

Choose parameters

- [ ] Shell texture
- [ ] Production entity
- [ ] Fruit weight
- [ ] % of double seeds
- [ ] Fruit flavor
- [ ] Kernel weight
- [ ] Kernel shape
- [ ] Kernel thickness
- [ ] 26.016 C2H2+ common fragment
- [ ] 28.019 C2H4+ common fragment
- [ ] 31.018 CH2OH+
- [ ] 33.033 CH4OH+ Methanol
- [ ] 34.996 H2SH+ Hydrogen sulfide
- [ ] 39.023 C3H3+ common fragment
- [ ] 41.039 C3H5+ common fragment
- [ ] 42.012
- [ ] 42.022
- [ ] 43.018 C2H3O+ common fragment
- [x] 43.03 CH2N2H+ Cyanamide
- [ ] 43.055 C3H7+ common fragment
- [ ] 44.025
- [ ] 45.033 C2H4OH+ Acetaldehyde
- [ ] 47.049 C2H6OH+ Ethanol
- [ ] 49.011 CH4SH+ Methanethiol
- [ ] 53.004
- [ ] 53.04 C4H5+ common fragment
- [ ] 53.048
- [ ] 55.054 C4H7+ Butanal, common fragment
- [ ] 56.026
- [ ] 57.035 C3H4OH+ common fragment
- [ ] 57.043 C2H4N2H+ Aminoacetonitrile
- [ ] 57.07 C4H9+ 1-Butanol
- [ ] 59.049 C3H6OH+ Acetone
- [x] 61.028 C2H4O2H+ Acetic Acid, fragment of esters
- [ ] 61.055 C3H8OH+
- [ ] 63.012 CO2H3O+ water cluster of Carbon dioxide
- [ ] 63.029 C2H6SH+ Dimethyl sulfide
- [ ] 63.043 C2H6O2H+ water cluster of Acetaldehyde
- [ ] 65.944
- [ ] 67.032 C3H2N2H+ Propanedinitrile
- [ ] 67.057 C5H7+ Pentanal, common fragment
- [ ] 67.992
- [ ] 69.003
- [x] 69.033 C4H4OH+ Furan
- [ ] 69.056
- [ ] 69.071 C5H9+ Isoprene, common fragment
- [ ] 71.051 C4H6OH+ Butenal
- [ ] 71.086 C5H11+ 2-Pentanol, 2-Methyl-1-butanol+3-Methyl-1-butanol, Pentanol
- [ ] 72.962
- [ ] 73.029 C3H4O2H+ Propiolactone, Propenoic acid
- [ ] 73.051
- [x] 73.064 C4H8OH+ 2-Methyl-Propanal
- [ ] 75.035
- [x] 75.045 C3H6O2H+ 1-Hydroxy-2-Propanone
- [ ] 75.072
- [ ] 76.954 CS2H+ Carbon disulfide
- [ ] 77.008
- [ ] 77.037
- [ ] 79.04 C2H6O3H+ cluster of ms61.028
- [ ] 79.06 C6H7+ Benzene, aromatic ring fragment
- [ ] 79.078
- [ ] 80.06
- [ ] 81.041 C4H4N2H+ Pyrazine
- [ ] 81.07 C6H9+
- [x] 83.051 C5H6OH+ Methylfuran
- [ ] 83.076
- [ ] 83.087 C6H11+ Hexenol, Hexanal
- [ ] 84.087
- [ ] 85.03
- [ ] 85.067 C5H8OH+ Pentanal, Pentanone
- [ ] 85.102 C6H13+ Hexanol
- [ ] 86.009
- [x] 87.045 C4H6O2H+ gamma Butyrolactone
- [x] 87.081 C5H10OH+ 2-Methyl Butanal, 3-Methyl Butanal, 2-Pentanone
- [ ] 89.061 C4H8O2H+ Ethyl Acetate
- [ ] 91.057 C4H10SH+ Diethyl sulfide
- [ ] 91.075 C4H10O2H+ Butanediol
- [ ] 93.04
- [ ] 93.073 C7H9+ Toluene
- [ ] 93.091
- [ ] 95.051 C6H6OH+ Phenol
- [ ] 95.088 C7H11+ Heptenal
- [x] 97.048 C5H4O2H+ Furfural
- [x] 97.066 C6H8OH+ Ethylfuran
- [ ] 97.102 C7H13+ Heptanal
- [x] 99.046 C5H6O2H+ 2-Furan Methanol
- [ ] 99.082 C6H10OH+ Hexenal
- [ ] 99.117 C7H15+ Heptanol
- [ ] 99.951
- [ ] 101.062 C5H8O2H+ 2,3-Pentanedione
- [ ] 101.097 C6H12OH+ Hexanal
- [ ] 103.052
- [ ] 103.077 C5H10O2H+ C5 esters and acids
- [ ] 103.115
- [ ] 105.039 C4H8OSH+ Methional
- [ ] 105.068 C8H9+ Phenyl Ethyl Alcohol
- [ ] 105.09 C5H12O2H+
- [ ] 107.044 C7H6OH+ Benzaldehyde
- [ ] 107.089 C8H11+ Ethyl Benzene, p-Xylene, m-Xylene, o-Xylene
- [ ] 109.065 C7H8OH+ Benzyl alcohol, Cresol
- [ ] 109.103 C8H13+ Octenal
- [ ] 111.047 C6H6O2H+
- [ ] 111.084
- [ ] 111.118 C8H15+ 1-Octen-3-ol
- [ ] 113.064 C6H8O2H+ 2(5H)-Furanone, 5,5-dimethyl-
- [ ] 113.099 C7H12OH+ Heptanal, Heptanone
- [ ] 113.133 C8H17+ 2-Ethyl-1-Hexanol, Octanol
- [ ] 115.078 C6H10O2H+ Caprolactone
- [ ] 115.114 C7H14OH+ 2-Heptanal (E o Z)
- [ ] 117.062 C9H9+
- [ ] 117.092 C6H12O2H+ Butanoic Acid Ethyl Ester, Butyl Acetate, Hexanoic Acid
- [ ] 117.958
- [ ] 119.106 C6H14O2H+
- [x] 121.067 C8H8OH+ Benzeneacetaldehyde
- [ ] 121.104 C9H13+
- [ ] 121.12
- [ ] 123.048
- [ ] 123.118 C9H15+ Nonenal
- [ ] 125.059 C7H8O2H+ Benzyl Alcohol
- [ ] 125.1 C8H12OH+ Octadienone
- [ ] 125.134 C9H17+ Nonanal, Nonenol
- [ ] 127.042 C6H6O3H+ Maltol
- [ ] 127.075 C7H10O2H+
- [ ] 127.114 C8H14OH+ 6-Methyl-5-Hepten-2-one
- [ ] 127.148 C9H19+ Nonanol
- [ ] 129.056 C6H8O3H+ Furaneol
- [ ] 129.094 C7H12O2H+
- [ ] 129.129 C8H16OH+ Octanal
- [ ] 131.108 C7H14O2H+ Isoamyl acetate
- [ ] 133.121 C7H16O2H+
- [ ] 134.973
- [ ] 135.12 C10H15+ Cymene
- [ ] 136.993
- [ ] 137.134 C10H17+ Limonene
- [x] 139.114 C9H14OH+ 2-Pentyl Furan
- [ ] 141.13 C9H16OH+ Nonenal, Nonenone
- [ ] 143.11 C8H14O2H+
- [ ] 143.145 C9H18OH+ Nonanal
- [ ] 145.123 C8H16O2H+ Hexyl Acetate
- [ ] 147.137 C8H18O2H+
- [ ] 149.059 C9H8O2H+ Cinnamic acid
- [ ] 149.119 C7H16O3H+
- [ ] 153.092 C9H12O2H+
- [ ] 153.131 C10H16OH+
- [ ] 155.003
- [ ] 155.178 C11H23+ Undecanol
- [ ] 157.123 C9H16O2H+ Whiskey lactone
- [ ] 157.161 C10H20OH+ Decanal
- [ ] 159.139 C9H18O2H+ C9 esters and acids
- [ ] 161.151 C9H20O2H+
- [ ] 163.132 C8H18O3H+
- [ ] 165.079
- [ ] 167.056
- [ ] 169.194 C12H25+ Dodecanol
- [ ] 189.175 C11H24O2H+
- [ ] 197.086
- [ ] 205.197 C15H25+ Sesquiterpenes
- [ ] 223.066

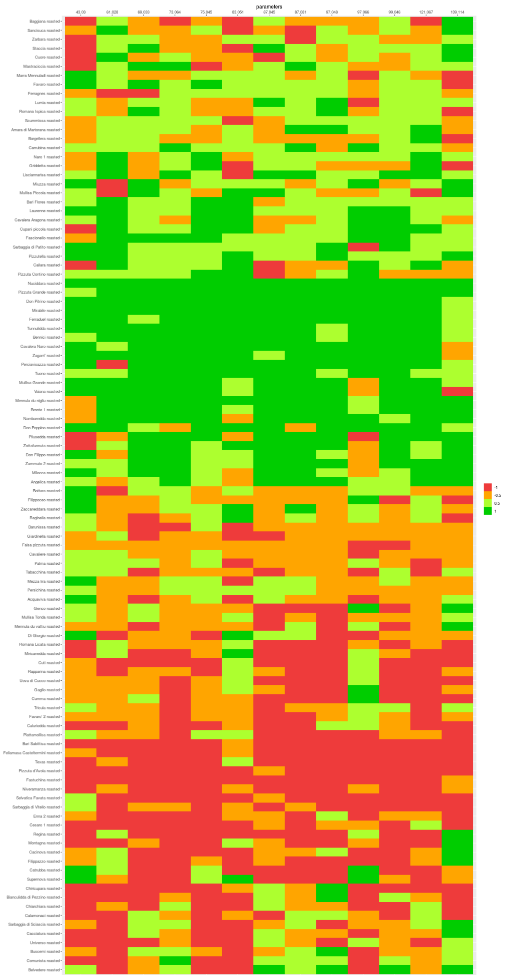

Supplement: Supplementary file 8 — Supplementary figure 3 [file 41438_2021_465_MOESM8_ESM.pdf]
