# Supplementary material for: Genetic characterization of an almond germplasm collection and volatilome profiling of raw and roasted kernels
Source: Hortic Res. 2021 Feb 1;8:27. doi: 10.1038/s41438-021-00465-7 (PMC7848010; doi:10.1038/s41438-021-00465-7)
Supplement: Supplementary file 6 — Supplementary figure 1 [file 41438_2021_465_MOESM6_ESM.pdf]

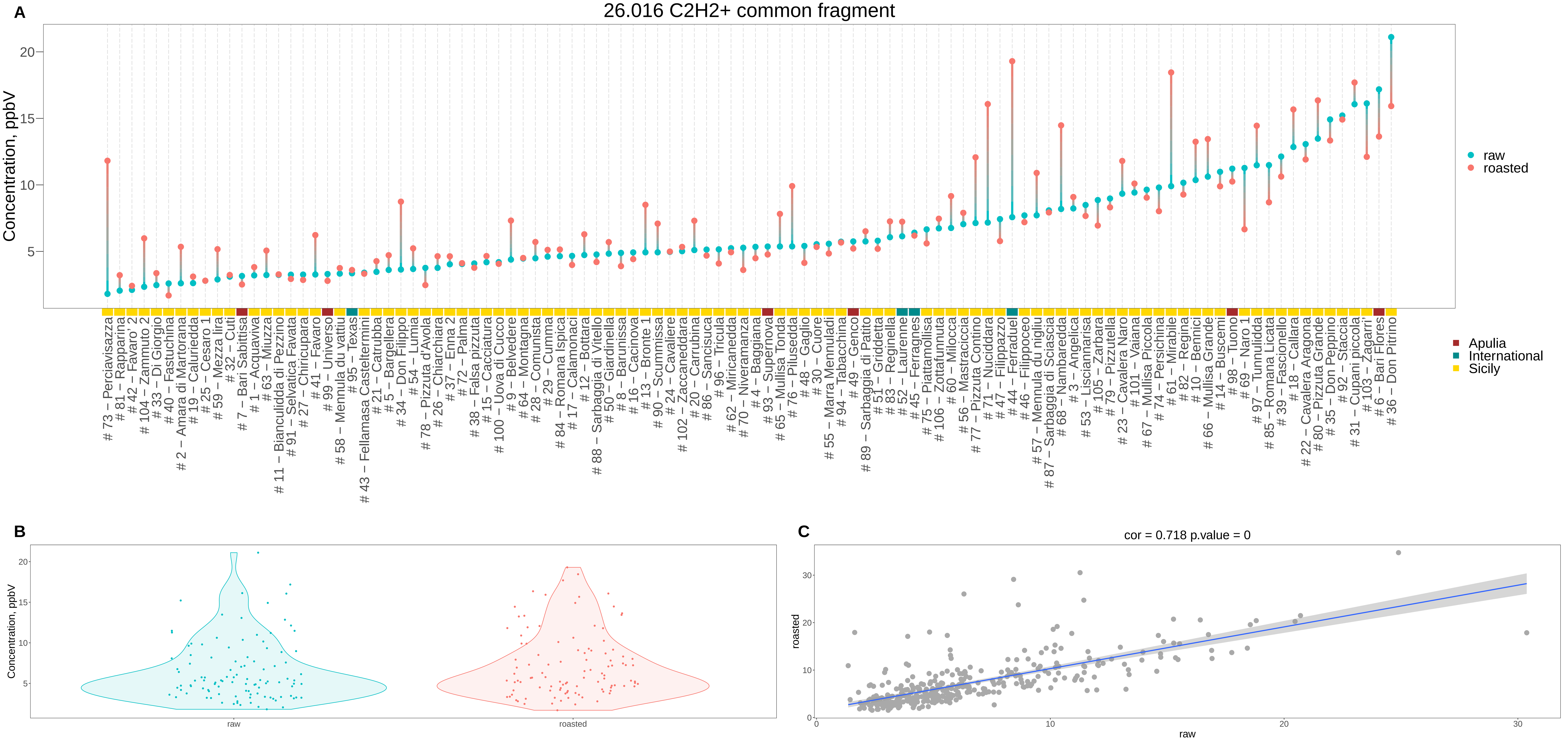
A
26.016 C2H2+ common fragment
Concentration, ppbV
20
15
10
5
raw
roasted
Apulia
International
Sicily
73 – Perciavisazza
81 – Rapparina
42 – Favaro' 2
104 – Zammuto 2
33 – Di Giorgio
40 – Fastuchina
2 – Amara di Martorana
19 – Caluriedda
25 – Cesaro 1
59 – Mezza lira
32 – Cuti
7 – Bari Sabittisa
1 – Acquaviva
63 – Miuzza
11 – Bianculidda di Pezzino
91 – Selvatica Favata
27 – Chiricupara
41 – Favaro
99 – Universo
58 – Mennula du vattiu
95 – Texas
43 – Fellamasa Casteltermini
21 – Catrubba
5 – Bargellera
34 – Don Filippo
54 – Lumia
78 – Pizzuta d'Avola
26 – Chiarchiara
37 – Enna 2
72 – Palma
38 – Falsa pizzuta
15 – Cacciatura
100 – Uova di Cucco
9 – Belvedere
64 – Montagna
28 – Comunista
29 – Cumma
84 – Romana Ispica
17 – Calamonaci
12 – Bottara
88 – Sarbaggia di Vitello
50 – Giardinella
8 – Barunissa
16 – Cacinova
13 – Bronte 1
90 – Scummissa
24 – Cavaliere
102 – Zaccaneddara
20 – Carrubina
86 – Sancisuca
96 – Tricula
62 – Miricanedda
70 – Niveramanza
4 – Baggiana
93 – Supernova
65 – Mullisa Tonda
76 – Pilusedda
48 – Gaglio
30 – Cuore
55 – Marra Mennuladi
94 – Tabacchina
49 – Genco
89 – Sarbaggia di Patito
51 – Griddetta
83 – Reginella
52 – Laurenne
45 – Ferragnes
75 – Piattamollisa
106 – Zottafunnuta
60 – Milocca
56 – Mastraciccia
77 – Pizzuta Contino
71 – Nuciddara
47 – Filippazzo
44 – Ferraduel
46 – Filippoceo
57 – Mennula du nigliu
87 – Sarbaggia di Sciascia
68 – Nambaredda
3 – Angelica
53 – Liscianarisa
105 – Zarbara
79 – Pizzutella
23 – Cavalera Naro
101 – Vaiana
67 – Mullisa Piccola
74 – Persichina
61 – Mirabile
82 – Regina
10 – Bennici
66 – Mullisa Grande
14 – Buscemi
98 – Tuono
69 – Naro 1
97 – Tunnulidda
85 – Romana Licata
39 – Fascionello
18 – Callara
22 – Cavalera Aragona
80 – Pizzuta Grande
35 – Don Peppino
92 – Staccia
31 – Cupani piccola
103 – Zagarri'
6 – Bari Flores
36 – Don Pitrino
B
Concentration, ppbV
20
15
10
5
raw
roasted
C
cor = 0.718 p.value = 0
roasted
30
20
10
0
0
10
20
30
raw

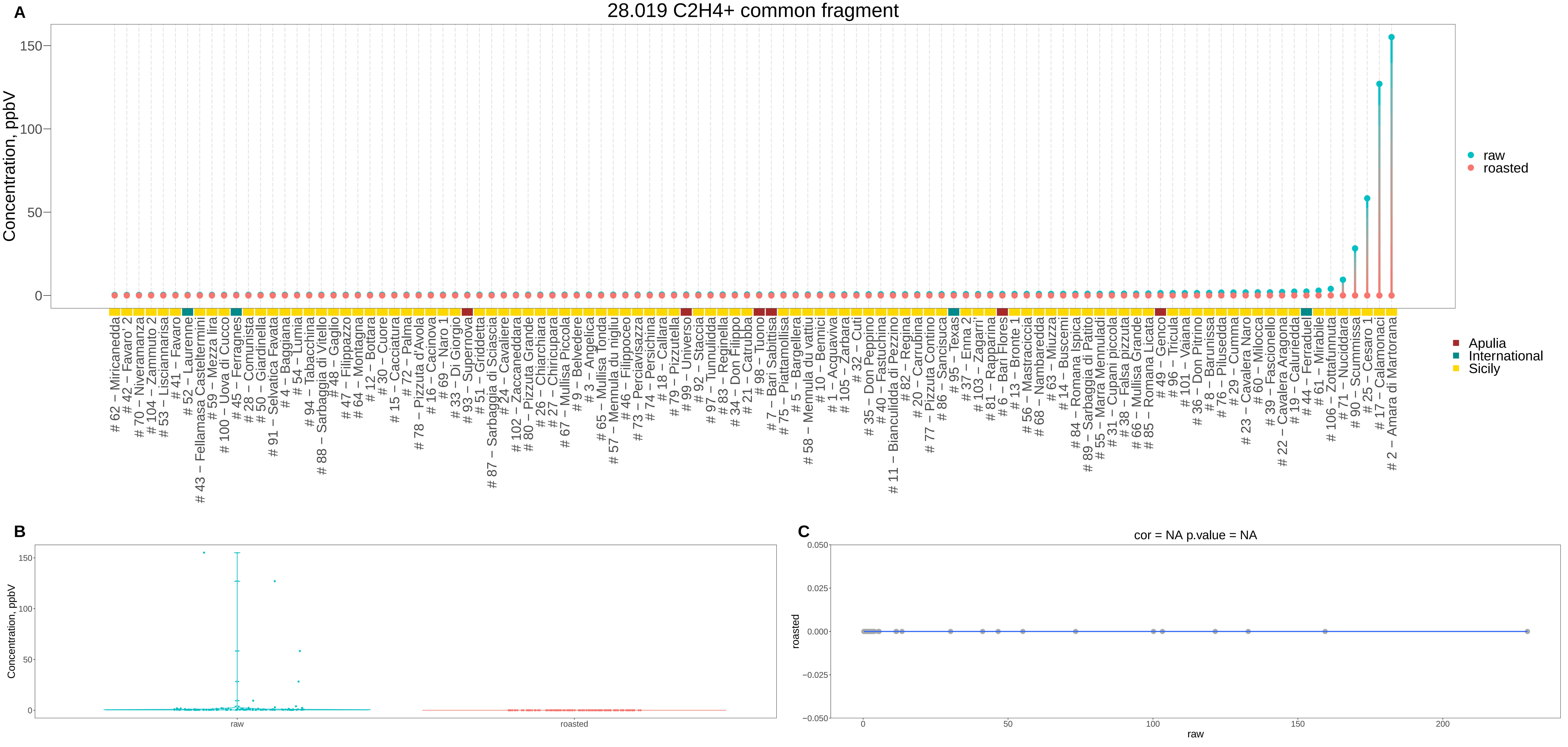
A
28.019 C2H4+ common fragment
Concentration, ppbV
raw
roasted
Apulia
International
Sicily
B
C
cor = NA p.value = NA
raw
roasted

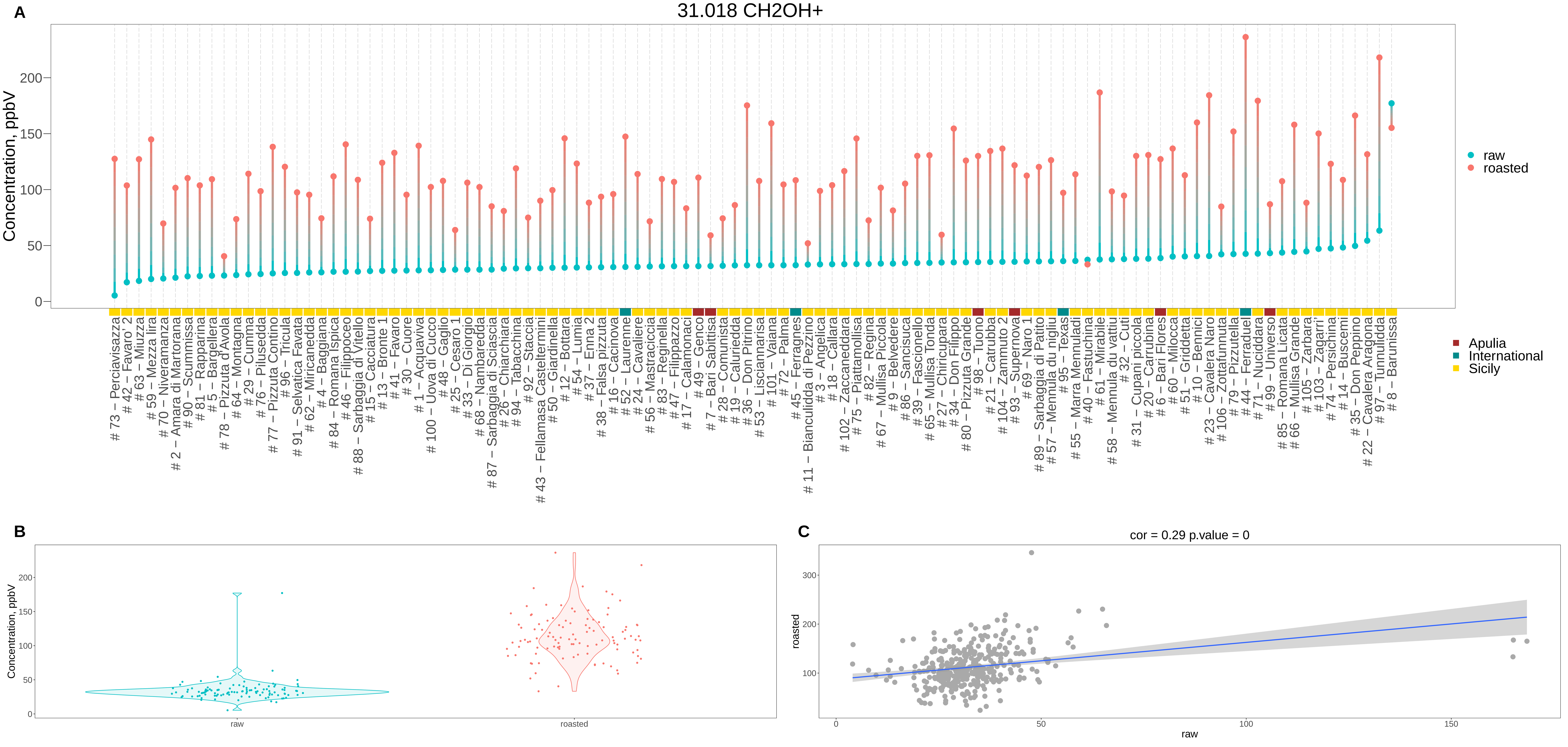

A
31.018 CH2OH+
Concentration, ppbV
0
50
100
150
200
raw
roasted
Apulia
International
Sicily
73 – Perciavisazza
42 – Favaro' 2
63 – Miuzza
59 – Mezza lira
70 – Niveramanza
2 – Amara di Martorana
90 – Scummissa
81 – Rapparina
5 – Bargellera
78 – Pizzuta d'Avola
64 – Montagna
29 – Cumma
76 – Pilusedda
77 – Pizzuta Contino
96 – Tricula
91 – Selvatica Favata
62 – Miricanedda
4 – Baggiana
84 – Romana Ispica
46 – Filippoceo
88 – Sarbaggia di Vitello
15 – Cacciatura
13 – Bronte 1
41 – Favaro
30 – Cuore
1 – Acquaviva
100 – Uova di Cucco
48 – Gaglio
25 – Cesaro 1
33 – Di Giorgio
68 – Nambaredda
87 – Sarbaggia di Sciascia
26 – Chiarchiara
94 – Tabacchina
92 – Staccia
43 – Fellamasa Casteltermini
50 – Giardinella
12 – Bottara
54 – Lumia
37 – Enna 2
38 – Falsa pizzuta
16 – Cacinova
52 – Laurenne
24 – Cavaliere
56 – Mastraciccia
83 – Reginella
47 – Filippazzo
17 – Calamonaci
49 – Genco
7 – Bari Sabittisa
28 – Comunista
19 – Caluriedda
36 – Don Pitrino
53 – Lisciannarisa
101 – Vaiana
72 – Palma
45 – Ferragnes
11 – Bianculidda di Pezzino
3 – Angelica
18 – Callara
102 – Zaccaneddara
75 – Piattamollisa
82 – Regina
67 – Mullisa Piccola
9 – Belvedere
86 – Sancisuca
39 – Fascionello
65 – Mullisa Tonda
27 – Chiricupara
34 – Don Filippo
80 – Pizzuta Grande
98 – Tuono
21 – Catrubba
104 – Zammuto 2
93 – Supernova
69 – Naro 1
89 – Sarbaggia di Patito
57 – Mennula du nigliu
95 – Texas
55 – Marra Mennuladi
40 – Fastuchina
61 – Mirabile
58 – Mennula du vattiu
32 – Cuti
31 – Cupani piccola
20 – Carrubina
6 – Bari Flores
60 – Milocca
51 – Griddetta
10 – Bennici
23 – Cavalera Naro
106 – Zottafunnuta
79 – Pizzutella
44 – Ferraduel
71 – Nuciddara
99 – Universo
85 – Romana Licata
66 – Mullisa Grande
105 – Zarbara
103 – Zagarri'
74 – Persichina
14 – Buscemi
35 – Don Peppino
22 – Cavalera Aragona
97 – Tunnulidda
8 – Barunissa
B
Concentration, ppbV
0
50
100
150
200
raw
roasted
C
cor = 0.29 p.value = 0
roasted
100
200
300
0
50
100
150
raw

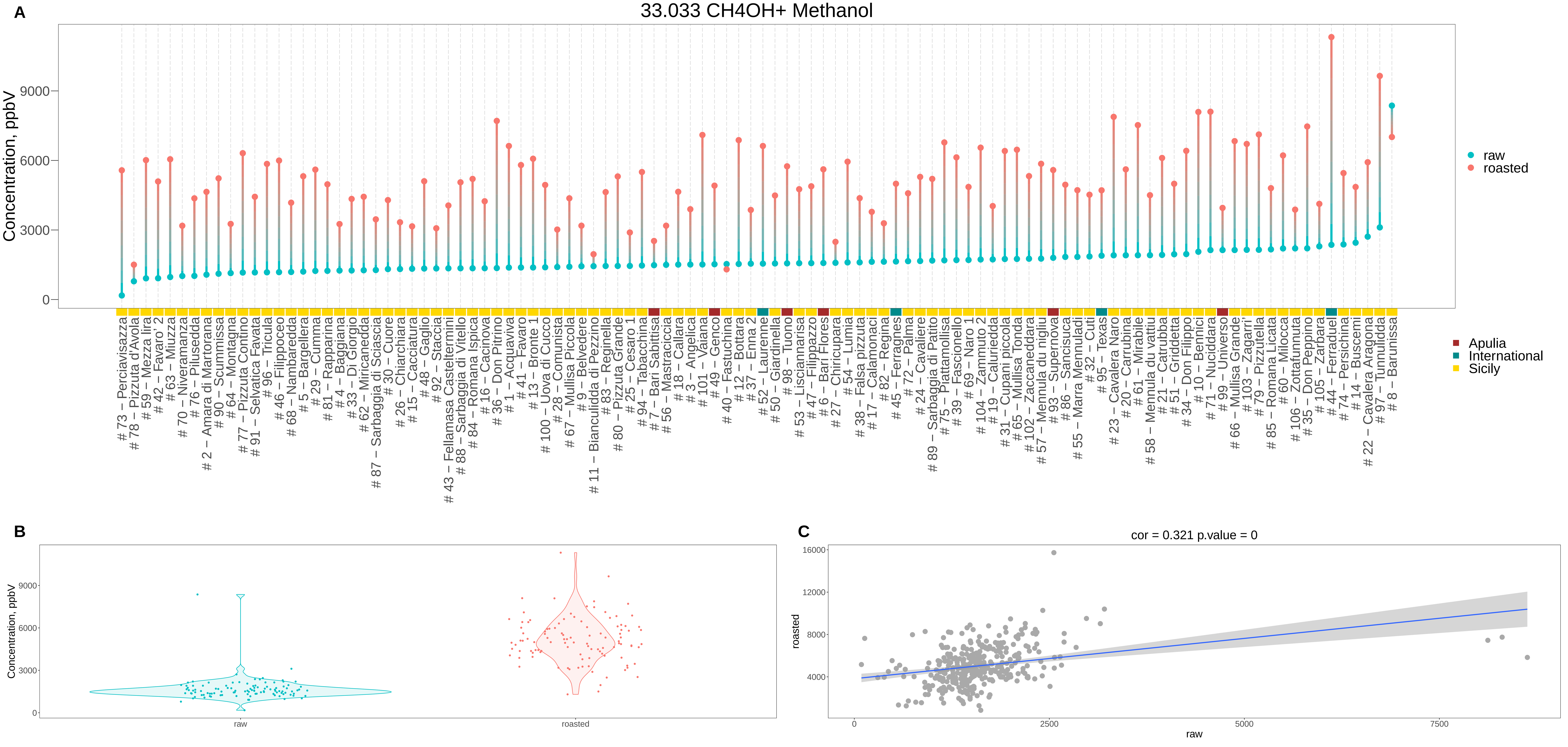
A
33.033 CH4OH+ Methanol
Concentration, ppbV
0
3000
6000
9000
raw
roasted
Apulia
International
Sicily
73 – Perciavisazza
78 – Pizzuta d'Avola
59 – Mezza lira
42 – Favaro' 2
63 – Miuzza
70 – Niveramanza
76 – Pilusedda
2 – Amara di Martorana
90 – Scummissa
64 – Montagna
77 – Pizzuta Contino
91 – Selvatica Favata
96 – Tricula
46 – Filippoceo
68 – Nambaredda
5 – Bargellera
29 – Cumma
81 – Rapparina
4 – Baggiana
33 – Di Giorgio
62 – Miricanedda
87 – Sarbaggia di Sciascia
30 – Cuore
26 – Chiarchiara
15 – Cacciatura
48 – Gaglio
92 – Staccia
43 – Fellamasa Casteltermini
88 – Sarbaggia di Vitello
84 – Romana Ispica
16 – Cacinova
36 – Don Pitrino
1 – Acquaviva
41 – Favaro
13 – Bronte 1
100 – Uova di Cucco
28 – Comunista
67 – Mullisa Piccola
9 – Belvedere
11 – Biancuilidda di Pezzino
83 – Reginella
80 – Pizzuta Grande
25 – Cesaro 1
94 – Tabacchina
7 – Bari Sabittisa
56 – Mastraciccia
18 – Callara
3 – Angelica
101 – Vaiana
49 – Genco
40 – Fastuchina
12 – Bottara
37 – Enna 2
52 – Laurenne
50 – Giardinella
98 – Tuono
53 – Liscianarisa
47 – Filippazzo
6 – Bari Flores
27 – Chiricupara
54 – Lumia
38 – Falsa pizzuta
17 – Calamonaci
82 – Regina
45 – Ferragnes
72 – Palma
24 – Cavaliere
89 – Sarbaggia di Patito
75 – Piattamollisa
39 – Fascionello
69 – Naro 1
104 – Zammuto 2
19 – Caluriedda
31 – Cupani piccola
65 – Mullisa Tonda
102 – Zaccaneddara
57 – Mennula du nigliu
93 – Supernova
86 – Sancisuca
55 – Marra Mennuladi
32 – Cuti
95 – Texas
23 – Cavalera Naro
20 – Carrubina
61 – Mirabile
58 – Mennula du vattiu
21 – Catrubba
51 – Griddetta
34 – Don Filippo
10 – Bennici
71 – Nuciddara
99 – Universo
66 – Mullisa Grande
103 – Zagarri'
79 – Pizzutella
85 – Romana Licata
60 – Milocca
106 – Zottafunnuta
35 – Don Peppino
105 – Zarbara
44 – Ferraduel
74 – Persichina
14 – Buscemi
22 – Cavalera Aragona
97 – Tunnulidda
8 – Barunissa
B
Concentration, ppbV
0
3000
6000
9000
raw
roasted
C
cor = 0.321 p.value = 0
roasted
4000
8000
12000
16000
0
2500
5000
7500
raw

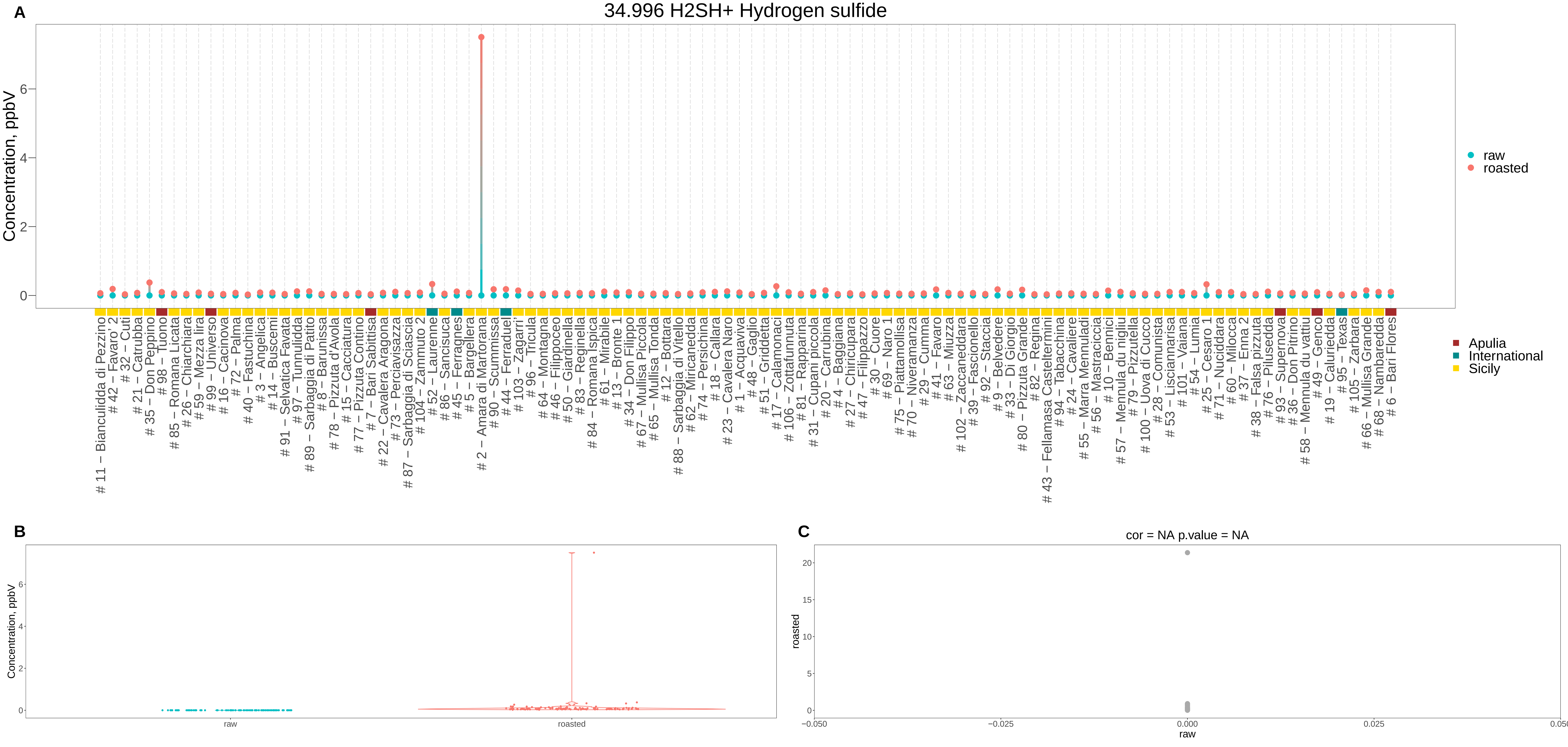
A
34.996 H2SH+ Hydrogen sulfide
Concentration, ppbV
raw
roasted
Apulia
International
Sicily
B
C
cor = NA p.value = NA
raw
roasted

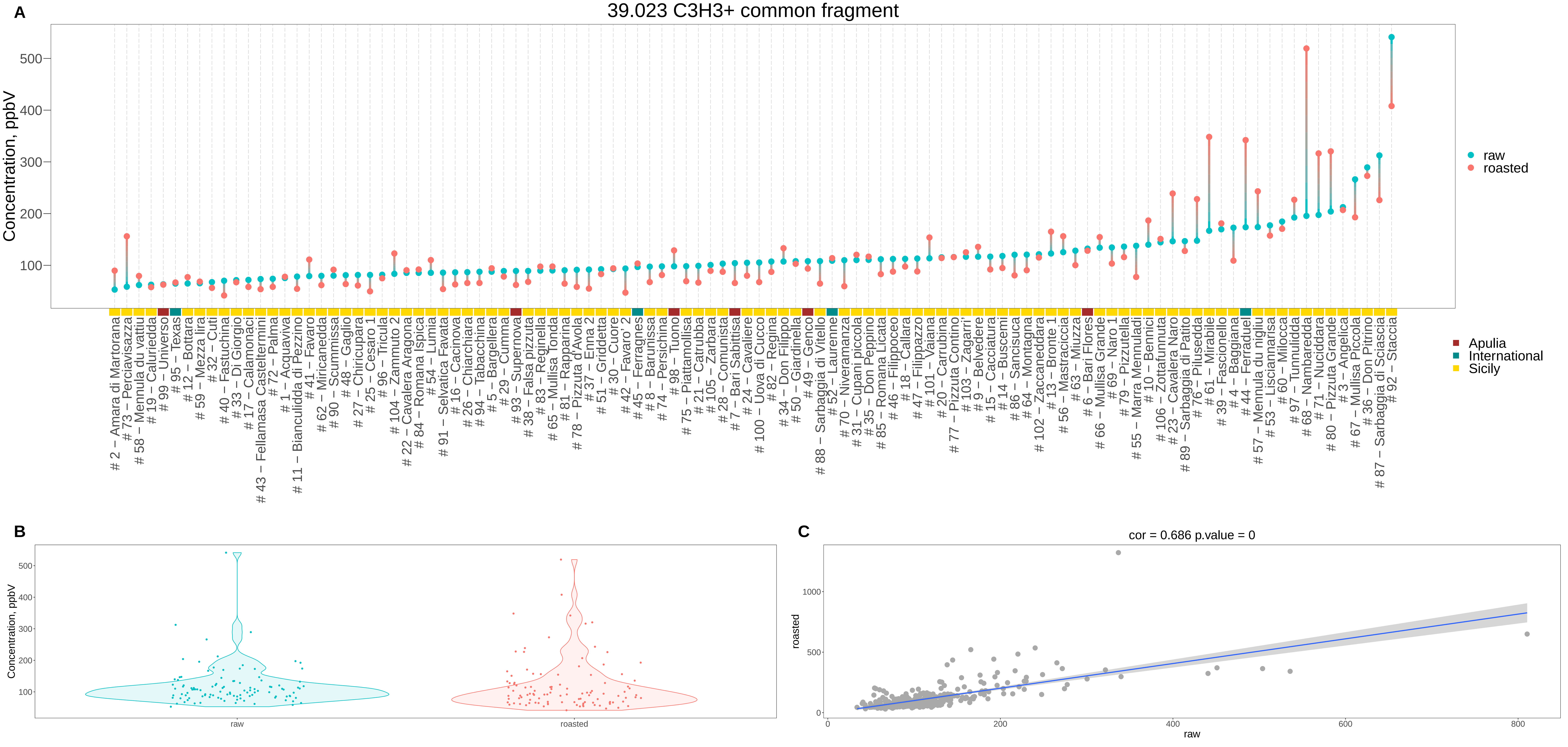

A
39.023 C3H3+ common fragment
Concentration, ppbV
100
200
300
400
500
raw
roasted
Apulia
International
Sicily
2 – Amara di Martorana
73 – Perciavisazza
58 – Mennula du vattiu
19 – Caluriedda
99 – Universo
95 – Texas
12 – Bottara
59 – Mezza lira
32 – Cuti
40 – Fastuchina
33 – Di Giorgio
17 – Calamonaci
43 – Fellamasa Casteltermini
72 – Palma
1 – Acquaviva
11 – Bianculidda di Pezzino
41 – Favaro
62 – Miricanedda
90 – Scummissa
48 – Gaglio
27 – Chiricupara
25 – Cesaro 1
96 – Tricula
104 – Zammuto 2
22 – Cavalera Aragona
84 – Romana Ispica
54 – Lumia
91 – Selvatica Favata
16 – Cacinova
26 – Chiarchiara
94 – Tabacchina
5 – Bargellera
29 – Cumma
93 – Supernova
38 – Falsa pizzuta
83 – Reginella
65 – Mullisa Tonda
81 – Rapparina
78 – Pizzuta d'Avola
37 – Enna 2
51 – Griddetta
30 – Cuore
42 – Favaro' 2
45 – Ferragnes
8 – Barunissa
74 – Persichina
98 – Tuono
75 – Piattamollisa
21 – Catrubba
105 – Zarbara
28 – Comunista
7 – Bari Sabittisa
24 – Cavaliere
100 – Uova di Cucco
82 – Regina
34 – Don Filippo
50 – Giardinella
49 – Genco
88 – Sarbaggia di Vitello
52 – Laurenne
70 – Niveramanza
31 – Cupani piccola
35 – Don Peppino
85 – Romana Licata
46 – Filippoceo
18 – Callara
47 – Filippazzo
101 – Vaiana
20 – Carrubina
77 – Pizzuta Contino
103 – Zagarri'
9 – Belvedere
15 – Cacciatura
14 – Buscemi
86 – Sancisuca
64 – Montagna
102 – Zaccaneddara
13 – Bronte 1
56 – Mastracicia
63 – Miuzza
6 – Bari Flores
66 – Mullisa Grande
69 – Naro 1
79 – Pizzutella
55 – Marra Mennuladi
10 – Bennici
106 – Zottafunnuta
23 – Cavalera Naro
89 – Sarbaggia di Patito
76 – Pilusedda
61 – Mirabile
39 – Fascionello
4 – Baggiana
44 – Ferraduel
57 – Mennula du nigliu
53 – Liscianarisa
60 – Milocca
97 – Tunnulidda
68 – Nambaredda
71 – Nuciddara
80 – Pizzuta Grande
3 – Angelica
67 – Mullisa Piccola
36 – Don Pitrino
87 – Sarbaggia di Sciascia
92 – Staccia
B
Concentration, ppbV
100
200
300
400
500
raw
roasted
C
cor = 0.686 p.value = 0
roasted
0
500
1000
raw
0
200
400
600
800

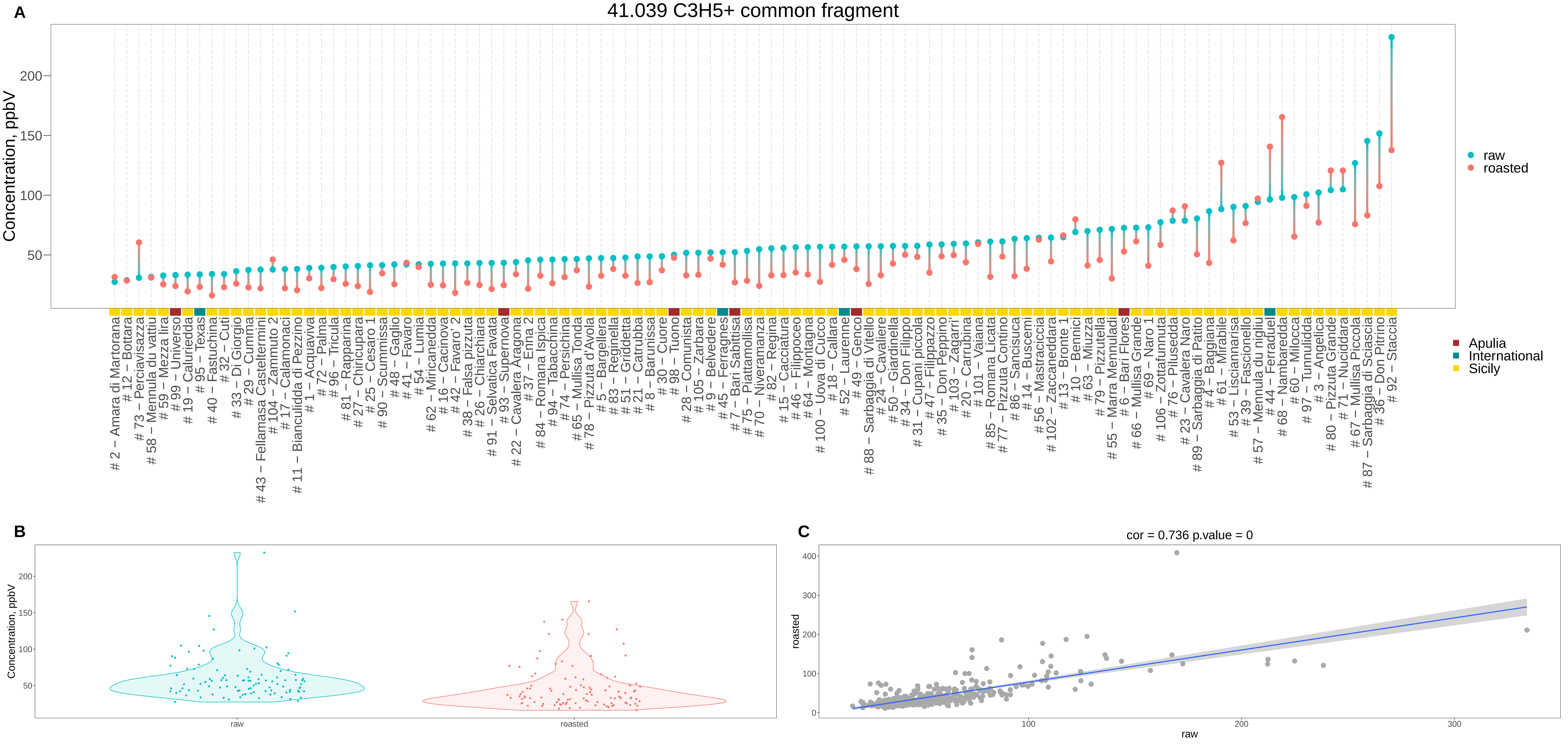
A
41.039 C3H5+ common fragment
Concentration, ppbV
200
150
100
50
raw
roasted
Apulia
International
Sicily
2 – Amara di Martorana
12 – Bottara
73 – Perciavisazza
58 – Mennula du vattiu
59 – Mezza lira
99 – Universo
19 – Caluriedda
95 – Texas
40 – Fastuchina
32 – Cuti
33 – Di Giorgio
29 – Cumma
43 – Fellamasa Casteltermini
104 – Zammuto 2
17 – Calamonaci
11 – Bianculidda di Pezzino
1 – Acquaviva
72 – Palma
96 – Tricula
81 – Rapparina
27 – Chiricupara
25 – Cesaro 1
90 – Scummissa
48 – Gaglio
41 – Favaro
54 – Lumia
62 – Miricanedda
16 – Cacinova
42 – Favaro' 2
38 – Falsa pizzuta
26 – Chiarchiara
91 – Selvatica Favata
93 – Supernova
22 – Cavalera Aragona
37 – Enna 2
84 – Romana Ispica
94 – Tabacchina
74 – Persichina
65 – Mullisa Tonda
78 – Pizzuta d'Avola
5 – Bargellera
83 – Reginella
51 – Griddetta
21 – Catrubba
8 – Barunissa
30 – Cuore
98 – Tuono
28 – Comunista
105 – Zarbara
9 – Belvedere
45 – Ferragnes
7 – Bari Sabittisa
75 – Piattamollisa
70 – Niveramanza
82 – Regina
15 – Cacciatura
46 – Filippoceo
64 – Montagna
100 – Uova di Cucco
18 – Callara
52 – Laurenne
49 – Genco
88 – Sarbaggia di Vitello
24 – Cavaliere
50 – Giardinella
34 – Don Filippo
31 – Cupani piccola
47 – Filippazzo
35 – Don Peppino
103 – Zagarri'
20 – Carrubina
101 – Vaiana
85 – Romana Licata
77 – Pizzuta Contino
86 – Sancisuca
14 – Buscemi
56 – Mastracicia
102 – Zaccaneddara
13 – Bronte 1
10 – Bennici
63 – Miuzza
79 – Pizzutella
55 – Marra Mennuladi
6 – Bari Flores
66 – Mullisa Grande
69 – Naro 1
106 – Zottafunnuta
76 – Pilusedda
23 – Cavalera Naro
89 – Sarbaggia di Patito
4 – Baggiana
61 – Mirabile
53 – Liscianarisa
39 – Fascionello
57 – Mennula du nigliu
44 – Ferraduel
68 – Nambaredda
60 – Milocca
97 – Tunnulidda
3 – Angelica
80 – Pizzuta Grande
71 – Nuciddara
67 – Mullisa Piccola
87 – Sarbaggia di Sciascia
36 – Don Pitrino
92 – Staccia
B
Concentration, ppbV
200
150
100
50
raw
roasted
C
cor = 0.736 p.value = 0
roasted
400
300
200
100
0
raw
100
200
300

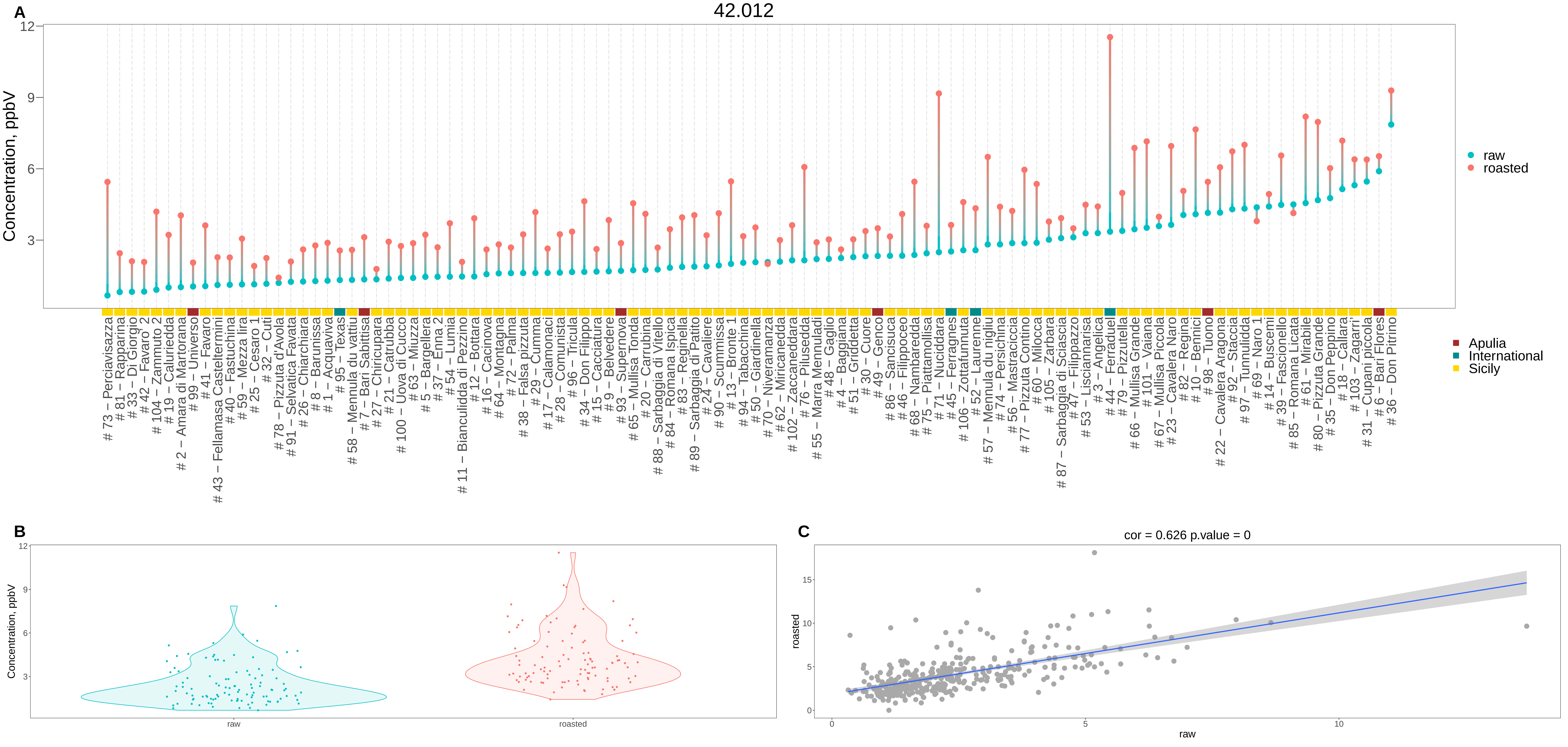
A
42.012
Concentration, ppbV
12
9
6
3
raw
roasted
Apulia
International
Sicily
73 – Perciavisazza
81 – Rapparina
33 – Di Giorgio
42 – Favaro' 2
104 – Zammuto 2
19 – Caluriedda
2 – Amara di Martorana
99 – Universo
41 – Favaro
43 – Fellamasa Casteltermini
40 – Fastuchina
59 – Mezza lira
25 – Cesaro 1
32 – Cuti
78 – Pizzuta d'Avola
91 – Selvatica Favata
26 – Chiarchiara
8 – Barunissa
1 – Acquaviva
95 – Texas
58 – Mennula du vattiu
7 – Bari Sabittisa
27 – Chiricupara
21 – Catrubba
100 – Uova di Cucco
63 – Miuzza
5 – Bargellera
37 – Enna 2
54 – Lumia
11 – Bianculidda di Pezzino
12 – Bottara
16 – Cacinova
64 – Montagna
72 – Palma
38 – Falsa pizzuta
29 – Cumma
17 – Calamonaci
28 – Comunista
96 – Tricula
34 – Don Filippo
15 – Cacciatura
9 – Belvedere
93 – Supernova
65 – Mullisa Tonda
20 – Carrubina
88 – Sarbaggia di Vitello
84 – Romana Ispica
83 – Reginella
89 – Sarbaggia di Patito
24 – Cavaliere
90 – Scummissa
13 – Bronte 1
94 – Tabacchina
50 – Giardinella
70 – Niveramanza
62 – Miricanedda
102 – Zaccaneddara
76 – Pilusedda
55 – Marra Mennuladi
48 – Gaglio
4 – Baggiana
51 – Griddetta
30 – Cuore
49 – Genco
86 – Sancisuca
46 – Filippoceo
68 – Nambaredda
75 – Piattamollisa
71 – Nuciddara
45 – Ferragnes
106 – Zottafunnuta
52 – Laurenne
57 – Mennula du nigliu
74 – Persichina
56 – Mastraciccia
77 – Pizzuta Contino
60 – Milocca
105 – Zarbara
87 – Sarbaggia di Sciascia
47 – Filippazzo
53 – Lisciannarisa
3 – Angelica
44 – Ferraduel
79 – Pizzutella
66 – Mullisa Grande
101 – Vaiana
67 – Mullisa Piccola
23 – Cavalera Naro
82 – Regina
10 – Bennici
98 – Tuono
22 – Cavalera Aragona
92 – Staccia
97 – Tunnulidda
69 – Naro 1
14 – Buscemi
39 – Fascionello
85 – Romana Licata
61 – Mirabile
80 – Pizzuta Grande
35 – Don Peppino
18 – Callara
103 – Zagarri'
31 – Cupani piccola
6 – Bari Flores
36 – Don Pitrino
B
Concentration, ppbV
12
9
6
3
raw
roasted
C
cor = 0.626 p.value = 0
roasted
15
10
5
0
0
5
10
raw

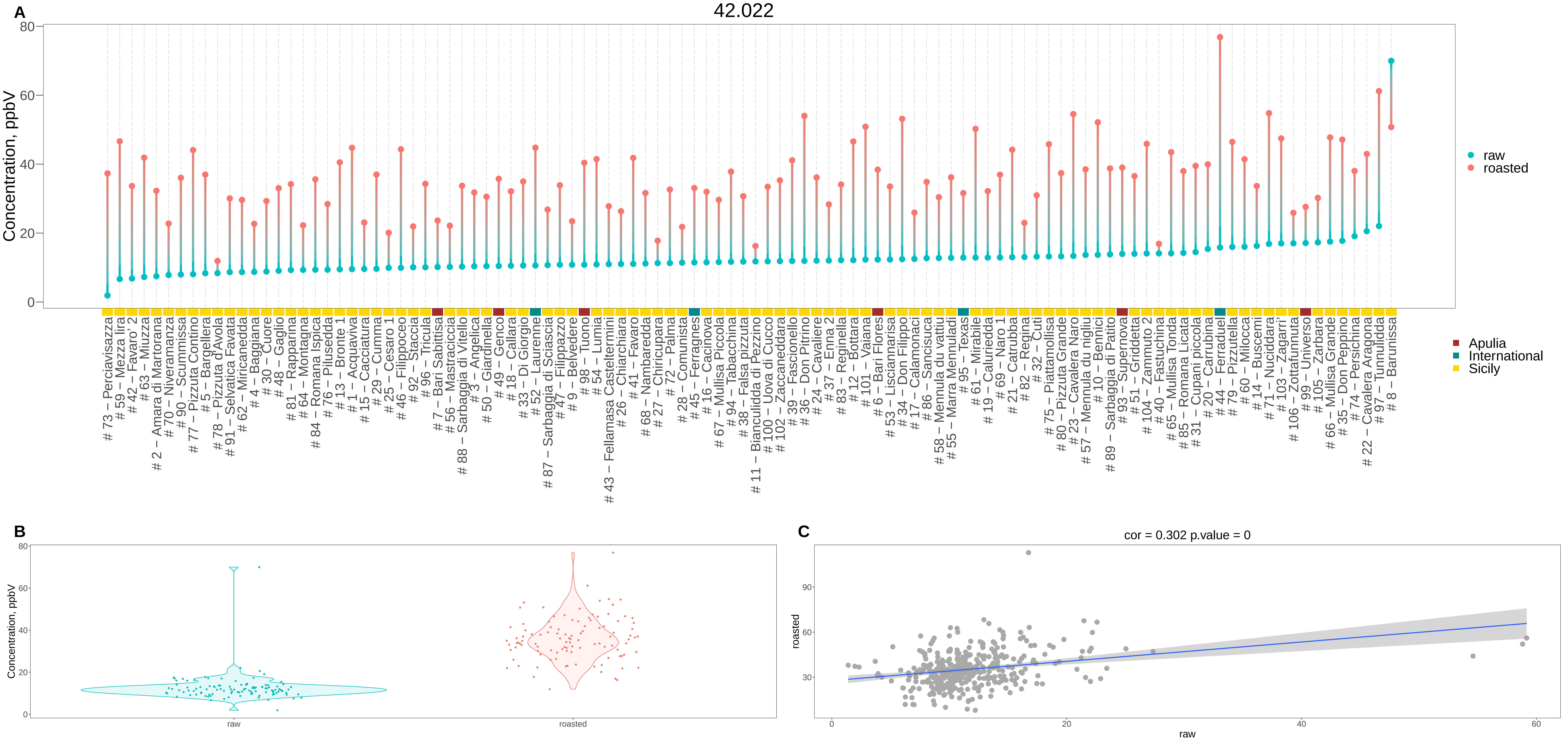
A
42.022
Concentration, ppbV
80
60
40
20
0
raw
roasted
Apulia
International
Sicily
73 – Perciavisazza
59 – Mezza lira
42 – Favaro' 2
63 – Miuzza
2 – Amara di Martorana
70 – Niveramanza
90 – Scummissa
77 – Pizzuta Contino
5 – Bargellera
78 – Pizzuta d'Avola
91 – Selvatica Favata
62 – Miricanedda
4 – Baggiana
30 – Cuore
48 – Gaglio
81 – Rapparina
64 – Montagna
84 – Romana Ispica
76 – Pilusedda
13 – Bronte 1
1 – Acquaviva
15 – Cacciatura
29 – Cumma
25 – Cesaro 1
46 – Filippoceo
92 – Staccia
96 – Tricula
7 – Bari Sabittisa
56 – Mastraciccia
88 – Sarbaggia di Vitello
3 – Angelica
50 – Giardinella
49 – Genco
18 – Callara
33 – Di Giorgio
52 – Laurenne
87 – Sarbaggia di Sciascia
47 – Filippazzo
9 – Belvedere
98 – Tuono
54 – Lumia
43 – Fellamasa Casteltermini
26 – Chiarchiara
41 – Favaro
68 – Nambaredda
27 – Chiricupara
72 – Palma
28 – Comunista
45 – Ferragnes
16 – Cacinova
67 – Mullisa Piccola
94 – Tabacchina
38 – Falsa pizzuta
11 – Bianculidda di Pezzino
100 – Uova di Cucco
102 – Zaccaneddara
39 – Fascionello
36 – Don Pitrino
24 – Cavaliere
37 – Enna 2
83 – Reginella
12 – Bottara
101 – Vaiana
6 – Bari Flores
53 – Lisciannarisa
34 – Don Filippo
17 – Calamonaci
86 – Sancisuca
58 – Mennula du vattiu
55 – Marra Mennuladi
95 – Texas
61 – Mirabile
19 – Caluriedda
69 – Naro 1
21 – Catrubba
82 – Regina
32 – Cuti
75 – Piattamollisa
80 – Pizzuta Grande
23 – Cavalera Naro
57 – Mennula du nigliu
10 – Bennici
89 – Sarbaggia di Patito
93 – Supernova
51 – Griddetta
104 – Zammuto 2
40 – Fastuchina
65 – Mullisa Tonda
85 – Romana Licata
31 – Cupani piccola
20 – Carrubina
44 – Ferraduel
79 – Pizzutella
60 – Milocca
14 – Buscemi
71 – Nuciddara
103 – Zagarri'
106 – Zottafunnuta
99 – Universo
105 – Zarbara
66 – Mullisa Grande
35 – Don Peppino
74 – Persichina
22 – Cavalera Aragona
97 – Tunnulidda
8 – Barunissa
B
Concentration, ppbV
80
60
40
20
0
raw
roasted
C
cor = 0.302 p.value = 0
roasted
90
60
30
0
20
40
60
raw

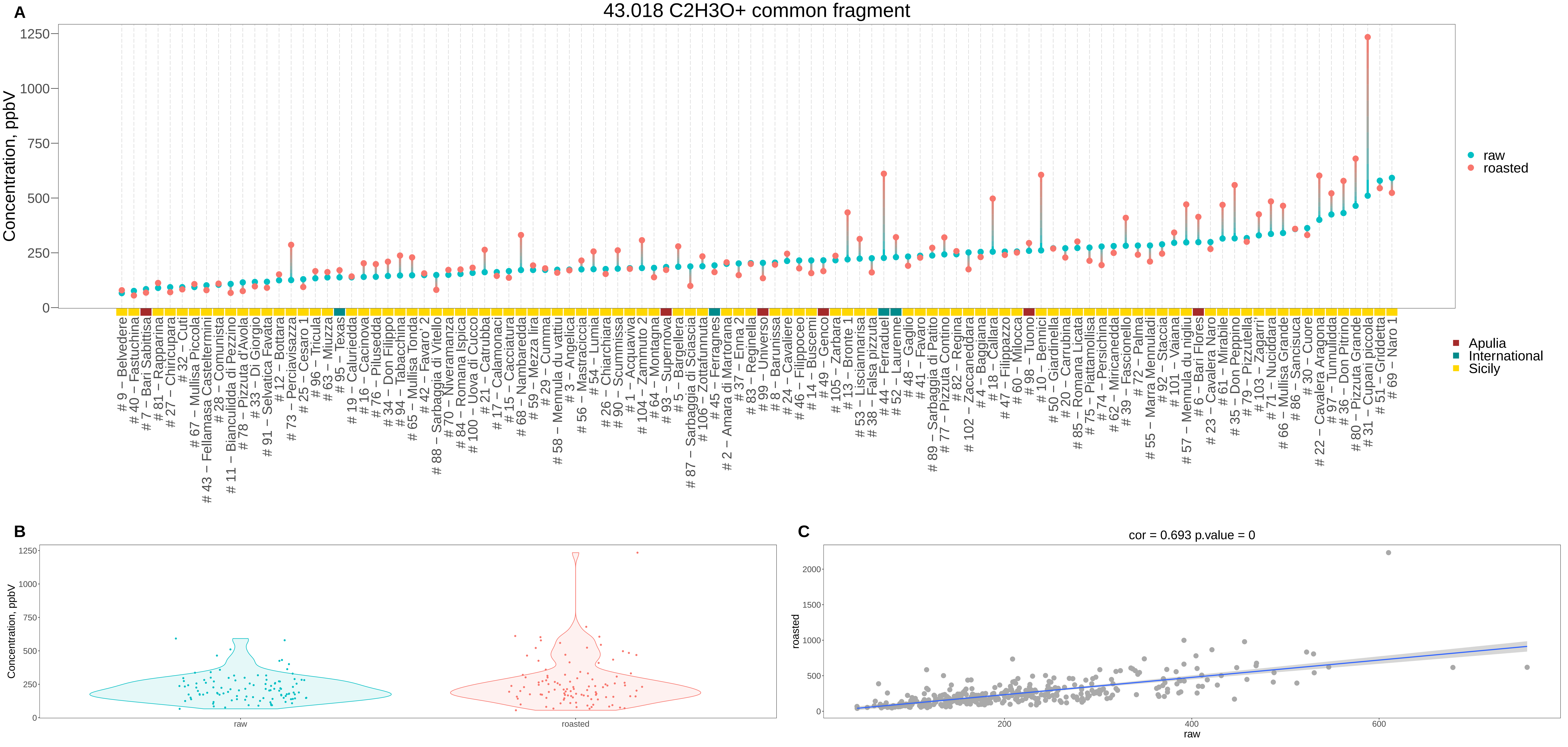
A
43.018 C2H3O+ common fragment
Concentration, ppbV
0
250
500
750
1000
1250
raw
roasted
Apulia
International
Sicily
9 – Belvedere
40 – Fastuchina
7 – Bari Sabitissa
81 – Rapparina
27 – Chiricupara
32 – Cuti
67 – Mullisa Piccola
43 – Fellamasa Casteltermini
28 – Comunista
11 – Bianculidda di Pezzino
78 – Pizzuta d'Avola
33 – Di Giorgio
91 – Selvatica Favata
12 – Bottara
73 – Perciavisazza
25 – Cesaro 1
96 – Tricula
63 – Miuzza
95 – Texas
19 – Caluriedda
16 – Cacinova
76 – Pilusedda
34 – Don Filippo
94 – Tabacchina
65 – Mullisa Tonda
42 – Favaro' 2
88 – Sarbaggia di Vitello
70 – Niveramanza
84 – Romana Ispica
100 – Uova di Cucco
21 – Catrubba
17 – Calamonaci
15 – Cacciatura
68 – Nambaredda
59 – Mezza lira
29 – Cumma
58 – Mennula du vattiu
3 – Angelica
56 – Mastraciccia
54 – Lumia
26 – Chiarchiara
90 – Scummissa
1 – Acquaviva
104 – Zammuto 2
64 – Montagna
93 – Supernova
5 – Bargellera
87 – Sarbaggia di Sciascia
106 – Zottafunnuta
45 – Ferragnes
2 – Amara di Martorana
37 – Enna 2
83 – Reginella
99 – Universo
8 – Barunissa
24 – Cavaliere
46 – Filippoceo
14 – Buscemi
49 – Genco
105 – Zarbara
13 – Bronte 1
53 – Liscianarisa
38 – Falsa pizzuta
44 – Ferraduel
52 – Laurenne
48 – Gaglio
41 – Favaro
89 – Sarbaggia di Patito
77 – Pizzuta Contino
82 – Regina
102 – Zaccaneddara
4 – Baggiana
18 – Callara
47 – Filippazzo
60 – Milocca
98 – Tuono
10 – Bennici
50 – Giardinella
20 – Carrubina
85 – Romana Licata
75 – Piattamollisa
74 – Persichina
62 – Miricanedda
39 – Fascionello
72 – Palma
55 – Marra Mennuladi
92 – Staccia
101 – Vaiana
57 – Mennula du nigliu
6 – Bari Flores
23 – Cavalera Naro
61 – Mirabile
35 – Don Peppino
79 – Pizzutella
103 – Zagarri'
71 – Nuciddara
66 – Mullisa Grande
86 – Sancisuca
30 – Cuore
22 – Cavalera Aragona
97 – Tunnulidda
36 – Don Pitrino
80 – Pizzuta Grande
31 – Cupani piccola
51 – Griddetta
69 – Naro 1
B
Concentration, ppbV
0
250
500
750
1000
1250
raw
roasted
C
cor = 0.693 p.value = 0
roasted
0
500
1000
1500
2000
raw
200
400
600

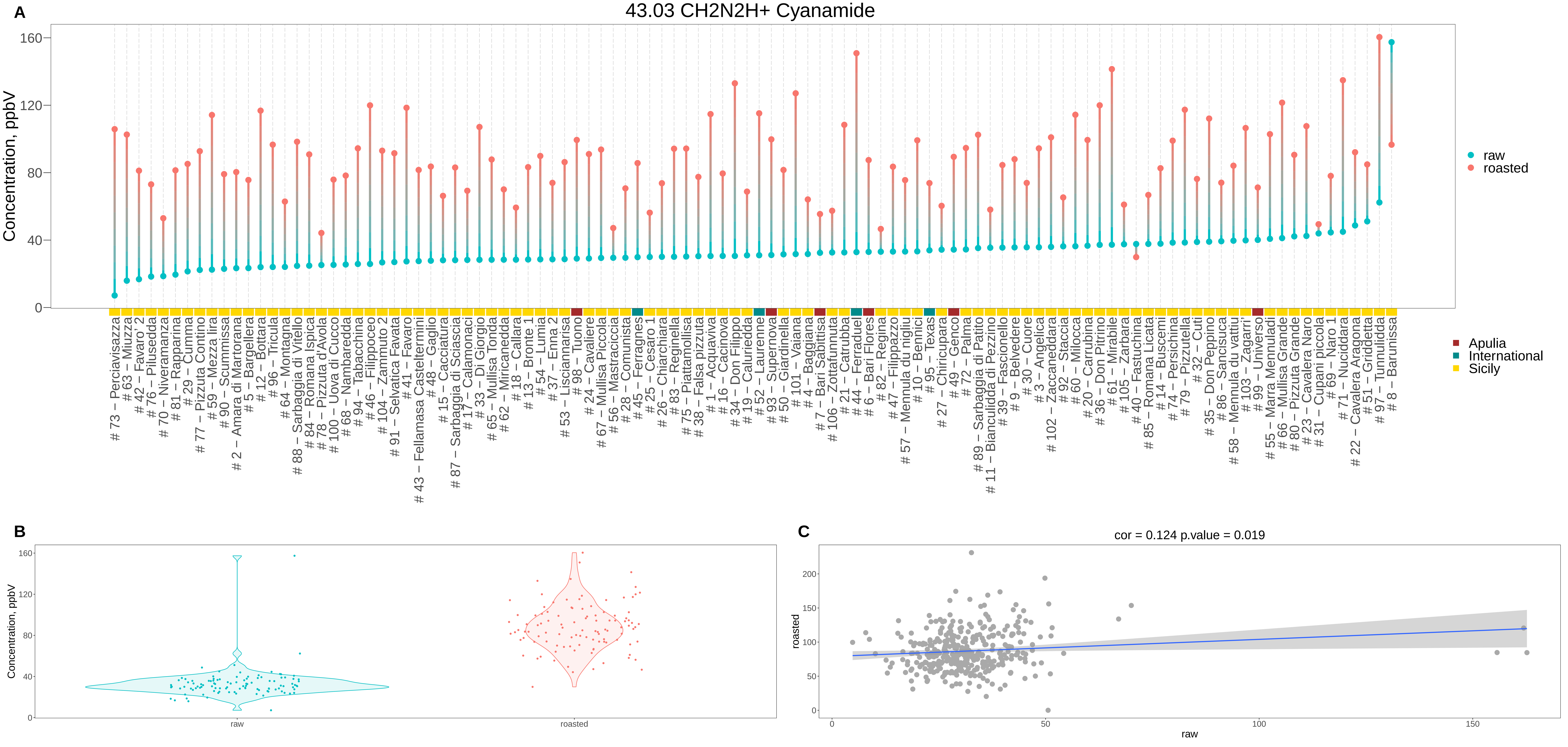

A
43.03 CH2N2H+ Cyanamide
Concentration, ppbV
0
40
80
120
160
raw
roasted
Apulia
International
Sicily
73 – Perciavisazza
63 – Miuzza
42 – Favaro' 2
76 – Pilusedda
70 – Niveramanza
81 – Rapparina
29 – Cumma
77 – Pizzuta Contino
59 – Mezza lira
90 – Scummissa
2 – Amara di Martorana
5 – Bargellera
12 – Bottara
96 – Tricula
64 – Montagna
88 – Sarbaggia di Vitello
84 – Romana Ispica
78 – Pizzuta d'Avola
100 – Uova di Cucco
68 – Nambaredda
94 – Tabacchina
46 – Filippoceo
104 – Zammuto 2
91 – Selvatica Favata
41 – Favaro
43 – Fellamasa Casteltermini
48 – Gaglio
15 – Cacciatura
87 – Sarbaggia di Sciascia
17 – Calamonaci
33 – Di Giorgio
65 – Mullisa Tonda
62 – Miricanedda
18 – Callara
13 – Bronte 1
54 – Lumia
37 – Enna 2
53 – Liscianarisa
98 – Tuono
24 – Cavaliere
67 – Mullisa Piccola
56 – Mastracicia
28 – Comunista
45 – Ferragnes
25 – Cesaro 1
26 – Chiarchiara
83 – Reginella
75 – Piattamollisa
38 – Falsa pizzuta
1 – Acquaviva
16 – Cacinova
34 – Don Filippo
19 – Caluriedda
52 – Laurenne
93 – Supernova
50 – Giardinella
101 – Vaiana
4 – Baggiana
7 – Bari Sabittisa
106 – Zottafunnuta
21 – Catrubba
44 – Ferraduel
6 – Bari Flores
82 – Regina
47 – Filippazzo
57 – Mennula du nigliu
10 – Bennici
95 – Texas
27 – Chiricupara
49 – Genco
72 – Palma
89 – Sarbaggia di Patito
11 – Biancuilda di Pezzino
39 – Fascionello
9 – Belvedere
30 – Cuore
3 – Angelica
102 – Zaccaneddara
92 – Staccia
60 – Milocca
20 – Carrubina
36 – Don Pitrino
61 – Mirabile
105 – Zarbara
40 – Fastuchina
85 – Romana Licata
14 – Buscemi
74 – Persichina
79 – Pizzutella
32 – Cuti
35 – Don Peppino
86 – Sancisuca
58 – Mennula du vattiu
103 – Zagarri'
99 – Universo
55 – Marra Mennuladi
66 – Mullisa Grande
80 – Pizzuta Grande
23 – Cavalera Naro
31 – Cupani piccola
69 – Naro 1
71 – Nuciddara
22 – Cavalera Aragona
51 – Griddetta
97 – Tunnulidda
8 – Barunissa
B
Concentration, ppbV
raw
roasted
C
cor = 0.124 p.value = 0.019
roasted
raw
0
50
100
150
200

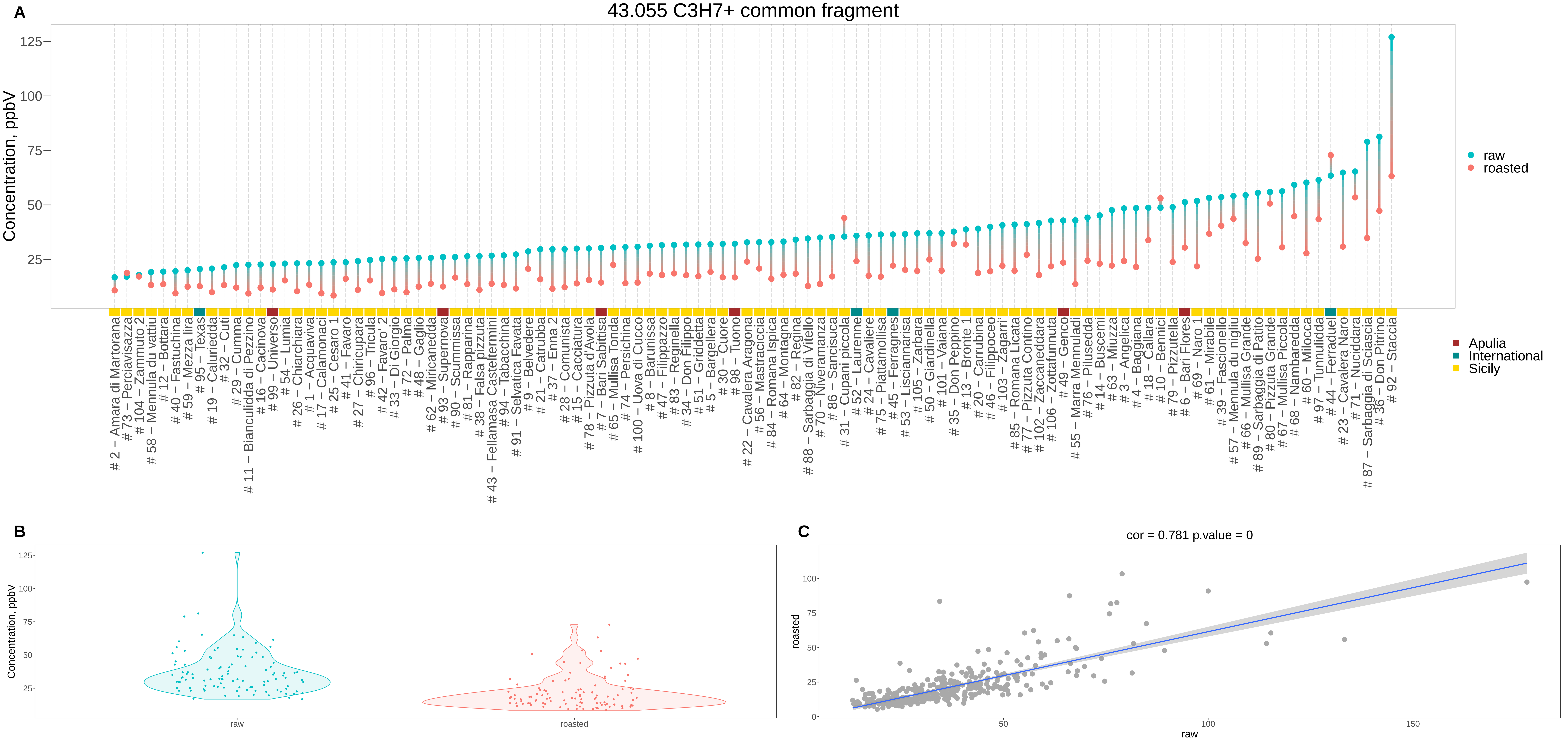
A
43.055 C3H7+ common fragment
Concentration, ppbV
125
100
75
50
25
raw
roasted
Apulia
International
Sicily
2 – Amara di Martorana
73 – Perciavisazza
104 – Zammuto 2
58 – Mennula du vattiu
12 – Bottara
40 – Fastuchina
59 – Mezza lira
95 – Texas
19 – Caluriedda
32 – Cuti
29 – Cumma
11 – Bianculidda di Pezzino
16 – Cacinova
99 – Universo
54 – Lumia
26 – Chiarchiara
1 – Acquaviva
17 – Calamonaci
25 – Cesaro 1
41 – Favaro
27 – Chiricupara
96 – Tricula
42 – Favaro' 2
33 – Di Giorgio
72 – Palma
48 – Gaglio
62 – Miricanedda
93 – Supernova
90 – Scummissa
81 – Rappanina
38 – Falsa pizzuta
43 – Fellamasa Casteltermini
94 – Tabacchina
91 – Selvatica Favata
9 – Belvedere
21 – Catrubba
37 – Enna 2
28 – Comunista
15 – Cacciatura
78 – Pizzuta d'Avola
7 – Bari Sabittisa
65 – Mullisa Tonda
74 – Persichina
100 – Uova di Cucco
8 – Barunissa
47 – Filippazzo
83 – Reginella
34 – Don Filippo
51 – Griddetta
5 – Bargellera
30 – Cuore
98 – Tuono
22 – Cavalera Aragona
56 – Mastraciccia
84 – Romana Ispica
64 – Montagna
82 – Regina
88 – Sarbaggia di Vitello
70 – Niveramanza
86 – Sancisuca
31 – Cupani piccola
52 – Laurenne
24 – Cavaliere
75 – Piattamollisa
45 – Ferragnes
53 – Liscianarisa
105 – Zarbara
50 – Giardinella
101 – Vaiana
35 – Don Peppino
13 – Bronte 1
20 – Carrubina
46 – Filippoceo
103 – Zagarri'
85 – Romana Licata
77 – Pizzuta Contino
102 – Zaccaneddara
106 – Zottafunnuta
49 – Genco
55 – Marra Mennuladi
76 – Pilusedda
14 – Busceni
63 – Miuzza
3 – Angelica
4 – Baggianna
18 – Callara
10 – Bennici
79 – Pizzutella
6 – Bari Flores
69 – Naro 1
61 – Mirabile
39 – Fascionello
57 – Mennula du nigliu
66 – Mullisa Grande
89 – Sarbaggia di Patito
80 – Pizzuta Grande
67 – Mullisa Piccola
68 – Nambaredda
60 – Milocca
97 – Tunnulidda
44 – Ferraduel
23 – Cavalera Naro
71 – Nuciddara
87 – Sarbaggia di Sciascia
36 – Don Pitrino
92 – Staccia
B
Concentration, ppbV
125
100
75
50
25
raw
roasted
C
cor = 0.781 p.value = 0
roasted
100
75
50
25
0
50
100
150
raw

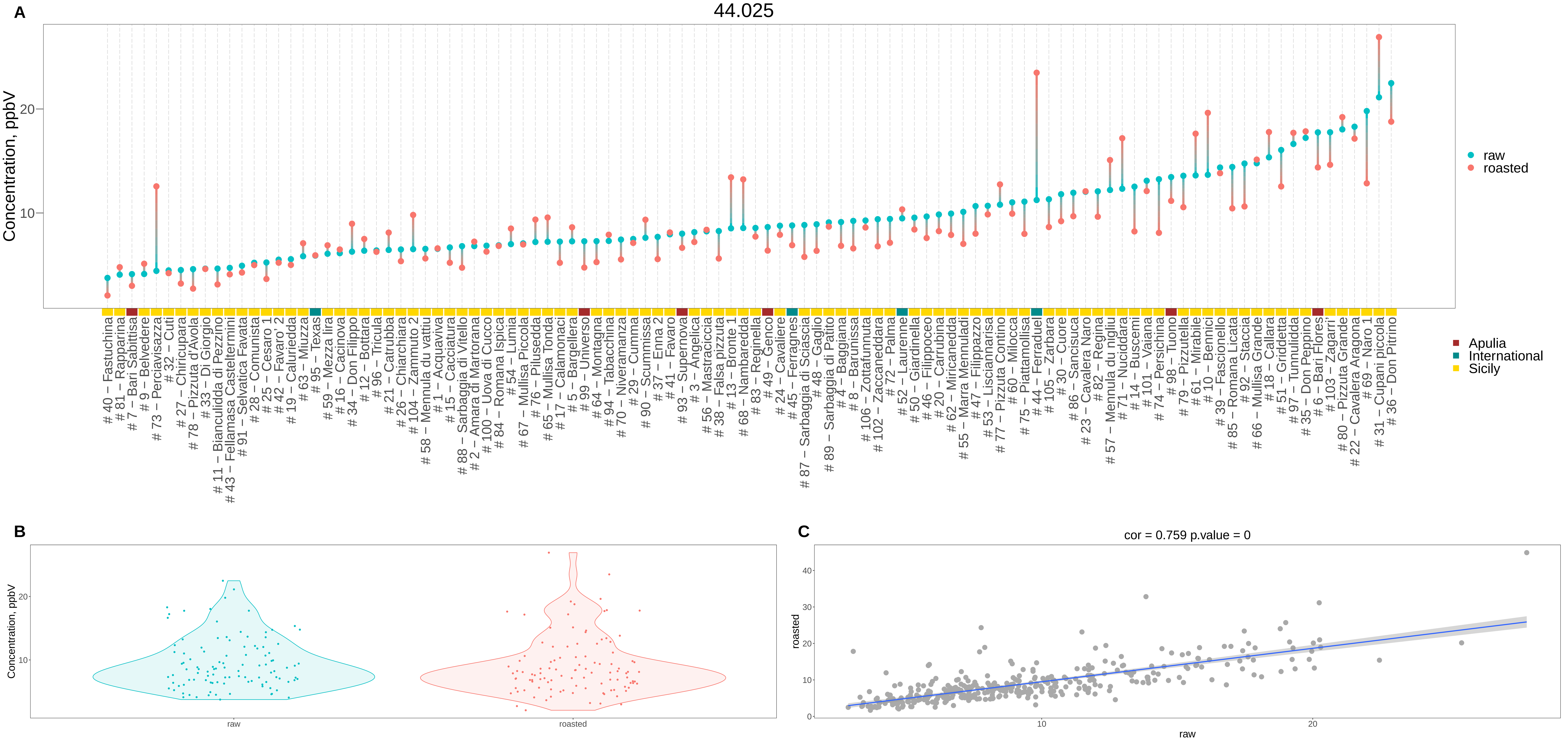
A
44.025
Concentration, ppbV
10
20
raw
roasted
Apulia
International
Sicily
40 – Fastuchina
81 – Rapparina
7 – Bari Sabittisa
9 – Belvedere
73 – Perciavisazza
32 – Cuti
27 – Chiricupara
78 – Pizzuta d'Avola
33 – Di Giorgio
11 – Bianculidda di Pezzino
43 – Fellamasa Casteltermini
91 – Selvatica Favata
28 – Comunista
25 – Cesaro 1
42 – Favaro' 2
19 – Caluriedda
63 – Miuzza
95 – Texas
59 – Mezza lira
16 – Cacinova
34 – Don Filippo
12 – Bottara
96 – Tricula
21 – Catrubba
26 – Chiarchiara
104 – Zammuto 2
58 – Mennula du vattiu
1 – Acquaviva
15 – Cacciatura
88 – Sarbaggia di Vitello
2 – Amara di Martorana
100 – Uova di Cucco
84 – Romana Ispica
54 – Lumia
67 – Mullisa Piccola
76 – Pilusedda
65 – Mullisa Tonda
17 – Calamonaci
5 – Bargellera
99 – Universo
64 – Montagna
94 – Tabacchina
70 – Niveramanza
29 – Cumma
90 – Scumissa
37 – Enna 2
41 – Favaro
93 – Supernova
3 – Angelica
56 – Mastraciccia
38 – Falsa pizzuta
13 – Bronte 1
68 – Nambaredda
83 – Reginella
49 – Genco
24 – Cavaliere
45 – Ferragnes
87 – Sarbaggia di Sciascia
48 – Gaglio
89 – Sarbaggia di Patito
4 – Baggiana
8 – Barunissa
106 – Zottafunnuta
102 – Zaccaneddara
72 – Palma
52 – Laurenne
50 – Giardinella
46 – Filippoceo
20 – Carrubina
62 – Miricanedda
55 – Marra Mennuladi
47 – Filippazzo
53 – Lisciannarisa
77 – Pizzuta Contino
60 – Milocca
75 – Piattamollisa
44 – Ferraduel
105 – Zarbara
30 – Cuore
86 – Sancisuca
23 – Cavalera Naro
82 – Regina
57 – Mennula du nigliu
71 – Nuciddara
14 – Buscemi
101 – Vaiana
74 – Persichina
98 – Tuono
79 – Pizzutella
61 – Mirabile
10 – Bennici
39 – Fascionello
85 – Romana Licata
92 – Staccia
66 – Mullisa Grande
18 – Callara
51 – Griddetta
97 – Tunnulidda
35 – Don Peppino
6 – Bari Flores
103 – Zagarri'
80 – Pizzuta Grande
22 – Cavalera Aragona
69 – Naro 1
31 – Cupani piccola
36 – Don Pitrino
B
Concentration, ppbV
10
20
raw
roasted
C
cor = 0.759 p.value = 0
roasted
0
10
20
30
40
raw
10
20

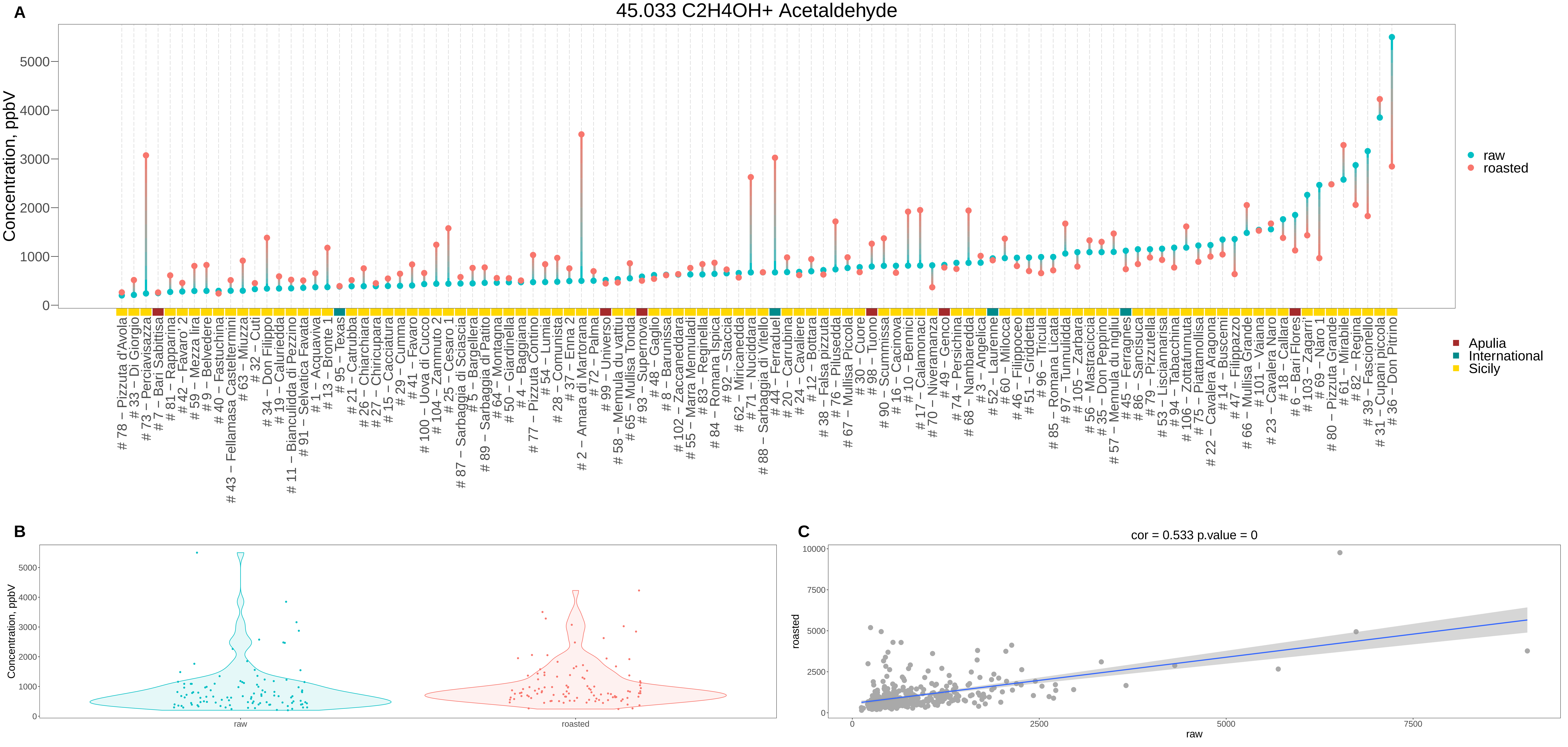

A
45.033 C2H4OH+ Acetaldehyde
Concentration, ppbV
raw
roasted
Apulia
International
Sicily
B
Concentration, ppbV
raw
roasted
C
cor = 0.533 p.value = 0
roasted
raw

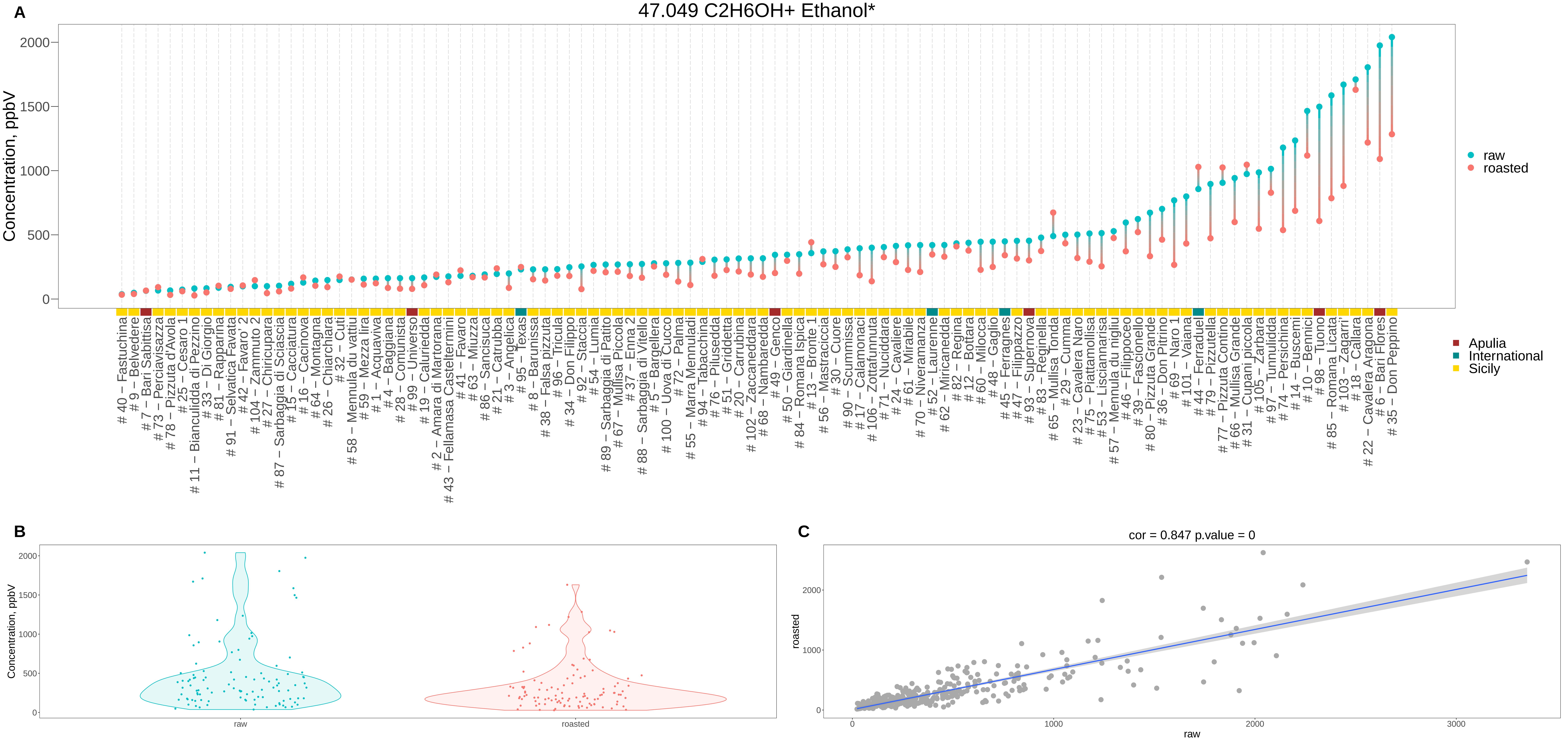

A
47.049 C2H6OH+ Ethanol*
Concentration, ppbV
2000
1500
1000
500
0
raw
roasted
Apulia
International
Sicily
40 – Fastuchina
9 – Belvedere
7 – Bari Sabitisa
73 – Perciavisazza
78 – Pizzuta d'Avola
25 – Cesaro 1
11 – Bianculidda di Pezzino
33 – Di Giorgio
81 – Rapparina
91 – Selvatica Favata
42 – Favaro' 2
104 – Zammuto 2
27 – Chiricupara
87 – Sarbaggia di Sciascia
15 – Cacciatura
16 – Cacinova
64 – Montagna
26 – Chiarchiara
32 – Cuti
58 – Mennula du vattiu
59 – Mezza lira
1 – Acquaviva
4 – Baggiana
28 – Comunista
99 – Universo
19 – Caluriedda
2 – Amara di Martorana
43 – Fellamasa Casteltermini
41 – Favaro
63 – Miuzza
86 – Sancisuca
21 – Catrubba
3 – Angelica
95 – Texas
8 – Barunissa
38 – Falsa pizzuta
96 – Tricula
34 – Don Filippo
92 – Staccia
54 – Lumia
89 – Sarbaggia di Patito
67 – Mullisa Piccola
37 – Enna 2
88 – Sarbaggia di Vitello
5 – Bargellera
100 – Uova di Cucco
72 – Palma
55 – Marra Mennuladi
94 – Tabacchina
76 – Pilusedda
51 – Griddetta
20 – Carrubina
102 – Zaccaneddara
68 – Nambaredda
49 – Genco
50 – Giardinella
84 – Romana Ispica
13 – Bronte 1
56 – Mastracicca
30 – Cuore
90 – Scummissa
17 – Calamonaci
106 – Zottafunnuta
71 – Nuciddara
24 – Cavaliere
61 – Mirabile
70 – Niveramanza
52 – Laurenne
62 – Miricanedda
82 – Regina
12 – Bottara
60 – Milocca
48 – Gaglio
45 – Ferragnes
47 – Filippazzo
93 – Supernova
83 – Reginella
65 – Mullisa Tonda
29 – Cumma
23 – Cavalera Naro
75 – Piattamollisa
53 – Liscianarisa
57 – Mennula du nigliu
46 – Filippoceo
39 – Fascionello
80 – Pizzuta Grande
36 – Don Pitrino
69 – Naro 1
101 – Vaiana
44 – Ferraduel
79 – Pizzutella
77 – Pizzuta Contino
66 – Mullisa Grande
31 – Cupani piccola
105 – Zarbara
97 – Tunnulidda
74 – Persichina
14 – Buscemi
10 – Bennici
98 – Tuono
85 – Romana Licata
103 – Zagarri'
18 – Callara
22 – Cavalera Aragona
6 – Bari Flores
35 – Don Peppino
B
Concentration, ppbV
2000
1500
1000
500
0
raw
roasted
C
cor = 0.847 p.value = 0
roasted
2000
1000
0
0
1000
2000
3000
raw

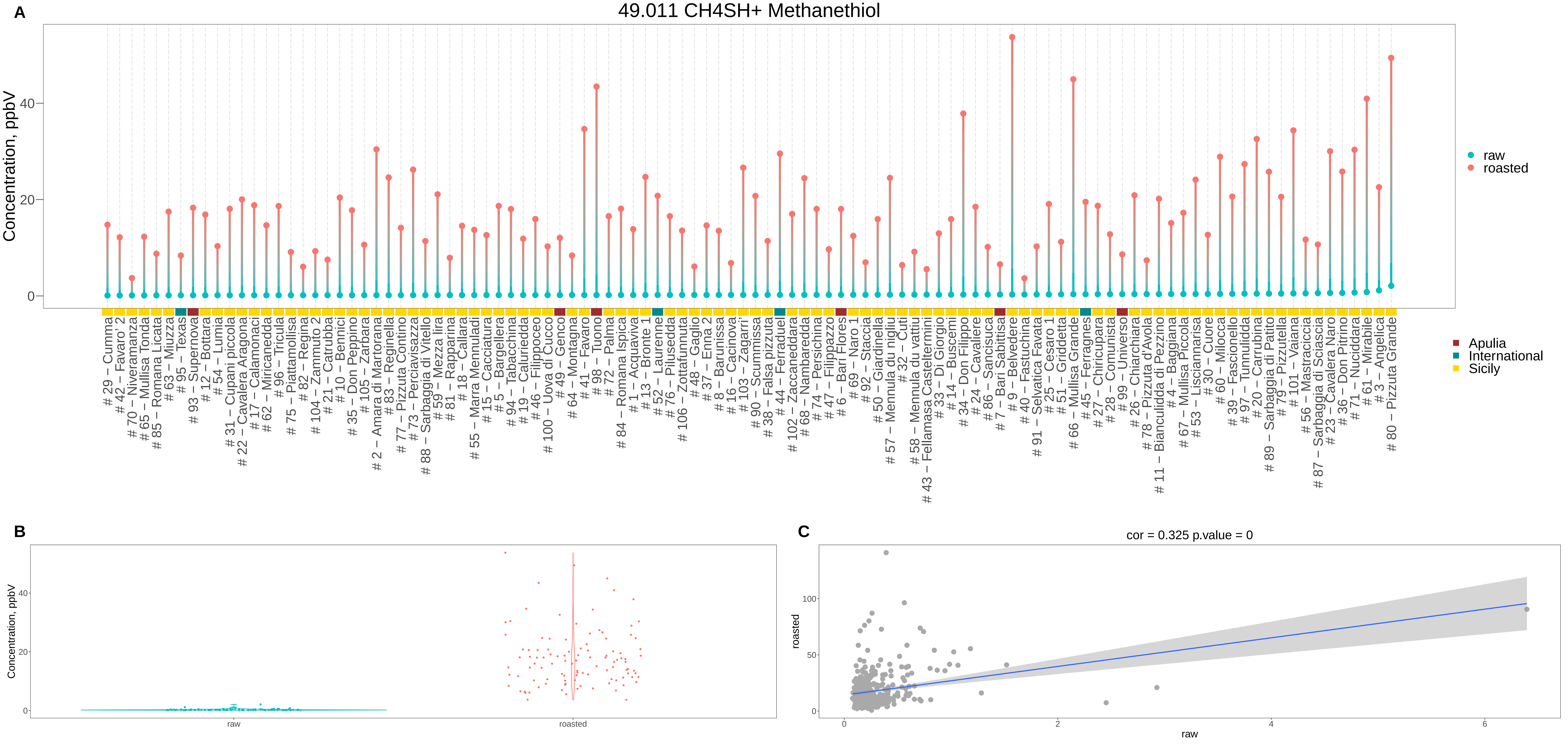
A
49.011 CH4SH+ Methanethiol
Concentration, ppbV
0
20
40
raw
roasted
Apulia
International
Sicily
29 – Cumma
42 – Favaro' 2
70 – Niveramanza
65 – Mullisa Tonda
85 – Romana Licata
63 – Miuzza
95 – Texas
93 – Supernova
12 – Bottara
54 – Lumia
31 – Cupani piccola
22 – Cavalera Aragona
17 – Calamonaci
62 – Miricanedda
96 – Tricula
75 – Piattamollisa
82 – Regina
104 – Zammuto 2
21 – Catrubba
10 – Bennici
35 – Don Peppino
105 – Zarbara
2 – Amara di Martorana
83 – Reginella
77 – Pizzuta Contino
73 – Perciavisazza
88 – Sarbaggia di Vitello
59 – Mezza lira
81 – Rapparina
18 – Callara
55 – Marra Mennuladi
15 – Cacciatura
5 – Bargellera
94 – Tabacchina
19 – Caluriedda
46 – Filippoceo
100 – Uova di Cucco
49 – Genco
64 – Montagna
41 – Favaro
98 – Tuono
72 – Palma
84 – Romana Ispica
1 – Acquaviva
13 – Bronte 1
52 – Laurenne
76 – Pilusedda
106 – Zottafunnuta
48 – Gaglio
37 – Enna 2
8 – Barunissa
16 – Cacinova
103 – Zagarri'
90 – Scummissa
38 – Falsa pizzuta
44 – Ferraduel
102 – Zaccaneddara
68 – Nambaredda
74 – Persichina
47 – Filippazzo
6 – Bari Flores
69 – Naro 1
92 – Staccia
50 – Giardinella
57 – Mennula du nigliu
32 – Cuti
58 – Mennula du vattiu
43 – Fellamasa Casteltermini
33 – Di Giorgio
14 – Buscemi
34 – Don Filippo
24 – Cavaliere
86 – Sancisuca
7 – Bari Sabitissa
9 – Belvedere
40 – Fastuchina
91 – Selvatica Favata
25 – Cesaro 1
51 – Griddetta
66 – Mullisa Grande
45 – Ferragnes
27 – Chiricupara
28 – Comunista
99 – Universo
26 – Chiarchiara
78 – Pizzuta d'Avola
11 – Bianculidda di Pezzino
4 – Baggiana
67 – Mullisa Piccola
53 – Liscianarisa
30 – Cuore
60 – Milocca
39 – Fascionello
97 – Tunnulidda
20 – Carrubina
89 – Sarbaggia di Patito
79 – Pizzutella
101 – Vaiana
56 – Mastraciccia
87 – Sarbaggia di Sciascia
23 – Cavalera Naro
36 – Don Pitrino
71 – Nucidara
61 – Mirabile
3 – Angelica
80 – Pizzuta Grande
B
Concentration, ppbV
0
20
40
raw
roasted
C
cor = 0.325 p.value = 0
roasted
0
50
100
raw
0
2
4
6

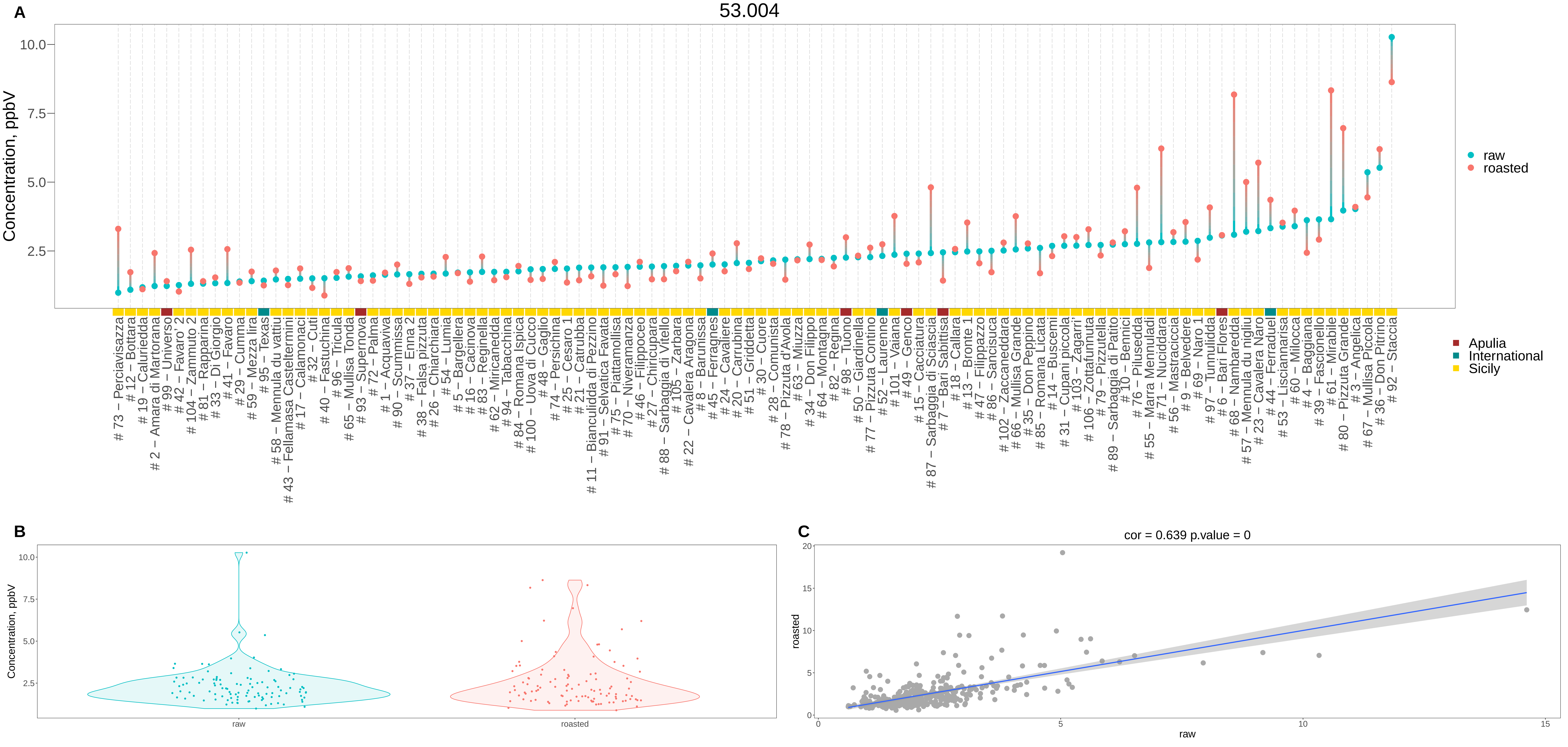
A
53.004
Concentration, ppbV
10.0
7.5
5.0
2.5
raw
roasted
Apulia
International
Sicily
73 – Perciavisazza
12 – Bottara
19 – Caluriedda
2 – Amara di Martorana
99 – Universo
42 – Favaro' 2
104 – Zammuto 2
81 – Rapparina
33 – Di Giorgio
41 – Favaro
29 – Cumma
59 – Mezza lira
95 – Texas
58 – Mennula du vattiu
43 – Fellamasa Casteltermini
17 – Calamonaci
32 – Cuti
40 – Fastuchina
96 – Tricula
65 – Mullisa Tonda
93 – Supernova
72 – Palma
1 – Acquaviva
90 – Scummissa
37 – Enna 2
38 – Falsa pizzuta
26 – Chiarchiara
54 – Lumia
5 – Bargellera
16 – Cacinova
83 – Reginella
62 – Miricanedda
94 – Tabacchina
84 – Romana Ispica
100 – Uova di Cucco
48 – Gaglio
74 – Persichina
25 – Cesaro 1
21 – Catrubba
11 – Bianculidda di Pezzino
91 – Selvatica Favata
75 – Piattamollisa
70 – Niveramanza
46 – Filippoceo
27 – Chiricupara
88 – Sarbaggia di Vitello
105 – Zarbara
22 – Cavalera Aragona
8 – Barunissa
45 – Ferragnes
24 – Cavaliere
20 – Carrubina
51 – Griddetta
30 – Cuore
28 – Comunista
78 – Pizzuta d'Avola
63 – Miuzza
34 – Don Filippo
64 – Montagna
82 – Regina
98 – Tuono
50 – Giardinella
77 – Pizzuta Contino
52 – Laurenne
101 – Vaiana
49 – Genco
15 – Cacciatura
87 – Sarbaggia di Sciascia
7 – Bari Sabittisa
18 – Callara
13 – Bronte 1
47 – Filippazzo
86 – Sancisuca
102 – Zaccaneddara
66 – Mullisa Grande
35 – Don Peppino
85 – Romana Licata
14 – Buscemi
31 – Cupani piccola
103 – Zagarri'
106 – Zottafunnuta
79 – Pizzutella
89 – Sarbaggia di Patito
10 – Bennici
76 – Pilusedda
55 – Marra Mennuladi
71 – Nuciddara
56 – Mastraciccia
9 – Belvedere
69 – Naro 1
97 – Tunnulidda
6 – Bari Flores
68 – Nambaredda
57 – Mennula du nigliu
23 – Cavalera Naro
44 – Ferraduel
53 – Liscianarisa
60 – Milocca
4 – Baggiana
39 – Fascionello
61 – Mirabile
80 – Pizzuta Grande
3 – Angelica
67 – Mullisa Piccola
36 – Don Pitrino
92 – Staccia
B
Concentration, ppbV
10.0
7.5
5.0
2.5
raw
roasted
C
cor = 0.639 p.value = 0
roasted
20
15
10
5
0
raw
0
5
10
15

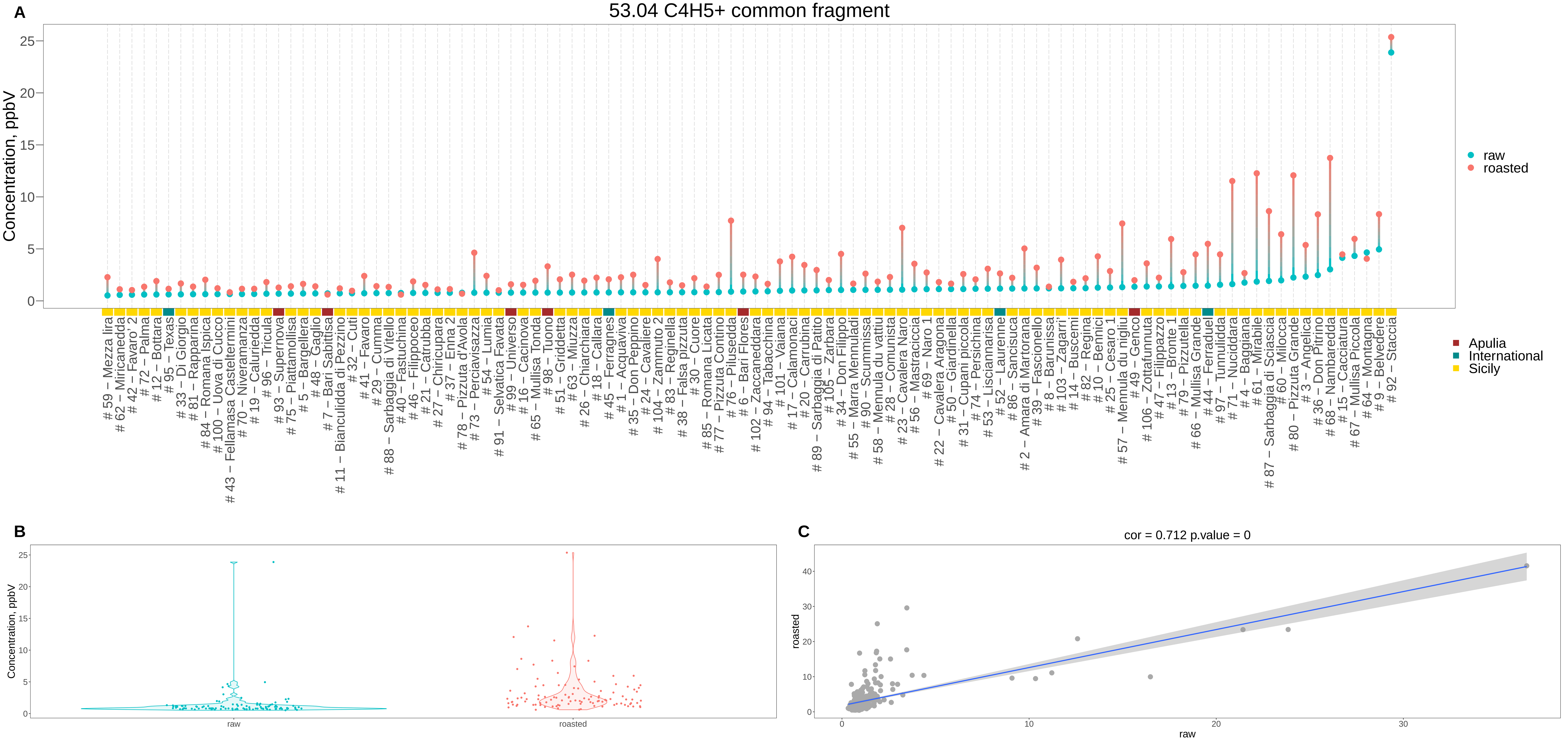
A
53.04 C4H5+ common fragment
Concentration, ppbV
0
5
10
15
20
25
raw
roasted
Apulia
International
Sicily
59 – Mezza lira
62 – Miricanedda
42 – Favaro' 2
72 – Palma
12 – Bottara
95 – Texas
33 – Di Giorgio
81 – Rapparina
84 – Romana Ispica
100 – Uova di Cucco
43 – Fellamasa Casteltermini
70 – Niveramanza
19 – Caluriedda
96 – Tricula
93 – Supernova
75 – Piattamollisa
5 – Bargellera
48 – Gaglio
7 – Bari Sabitisa
11 – Bianculidda di Pezzino
32 – Cuti
41 – Favaro
29 – Cumma
88 – Sarbaggia di Vitello
40 – Fastuchina
46 – Filippoceo
21 – Catrubba
27 – Chiricupara
37 – Enna 2
78 – Pizzuta d'Avola
73 – Perciavisazza
54 – Lumia
91 – Selvatica Favata
99 – Universo
16 – Cacinova
65 – Mullisa Tonda
98 – Tuono
51 – Griddetta
63 – Miuzza
26 – Chiarchiara
18 – Callara
45 – Ferragnes
1 – Acquaviva
35 – Don Peppino
24 – Cavaliere
104 – Zammuto 2
83 – Reginella
38 – Falsa pizzuta
30 – Cuore
85 – Romana Licata
77 – Pizzuta Contino
76 – Pilusedda
6 – Bari Flores
102 – Zaccaneddara
94 – Tabacchina
101 – Vaiana
17 – Calamonaci
20 – Carrubina
89 – Sarbaggia di Patito
105 – Zarbara
34 – Don Filippo
55 – Marra Mennuladi
90 – Scummissa
58 – Mennula du vattiu
28 – Comunista
23 – Cavalera Naro
56 – Mastraciccia
69 – Naro 1
22 – Cavalera Aragona
50 – Giardinella
31 – Cupani piccola
74 – Persichina
53 – Lisciannarisa
52 – Laurenne
86 – Sancisuca
2 – Amara di Martorana
39 – Fascionello
8 – Barunissa
103 – Zagarri'
14 – Buscemi
82 – Regina
10 – Bennici
25 – Cesaro 1
57 – Mennula du nigliu
49 – Genco
106 – Zottafunnuta
47 – Filippazzo
13 – Bronte 1
79 – Pizzutella
66 – Mullisa Grande
44 – Ferraduel
97 – Tunnulidda
71 – Nuciddara
4 – Baggiana
61 – Mirabile
87 – Sarbaggia di Sciascia
60 – Milocca
80 – Pizzuta Grande
3 – Angelica
36 – Don Pitrino
68 – Nambaredda
15 – Cacciatura
67 – Mullisa Piccola
64 – Montagna
9 – Belvedere
92 – Staccia
B
Concentration, ppbV
0
5
10
15
20
25
raw
roasted
C
cor = 0.712 p.value = 0
roasted
0
10
20
30
40
raw
0
10
20
30

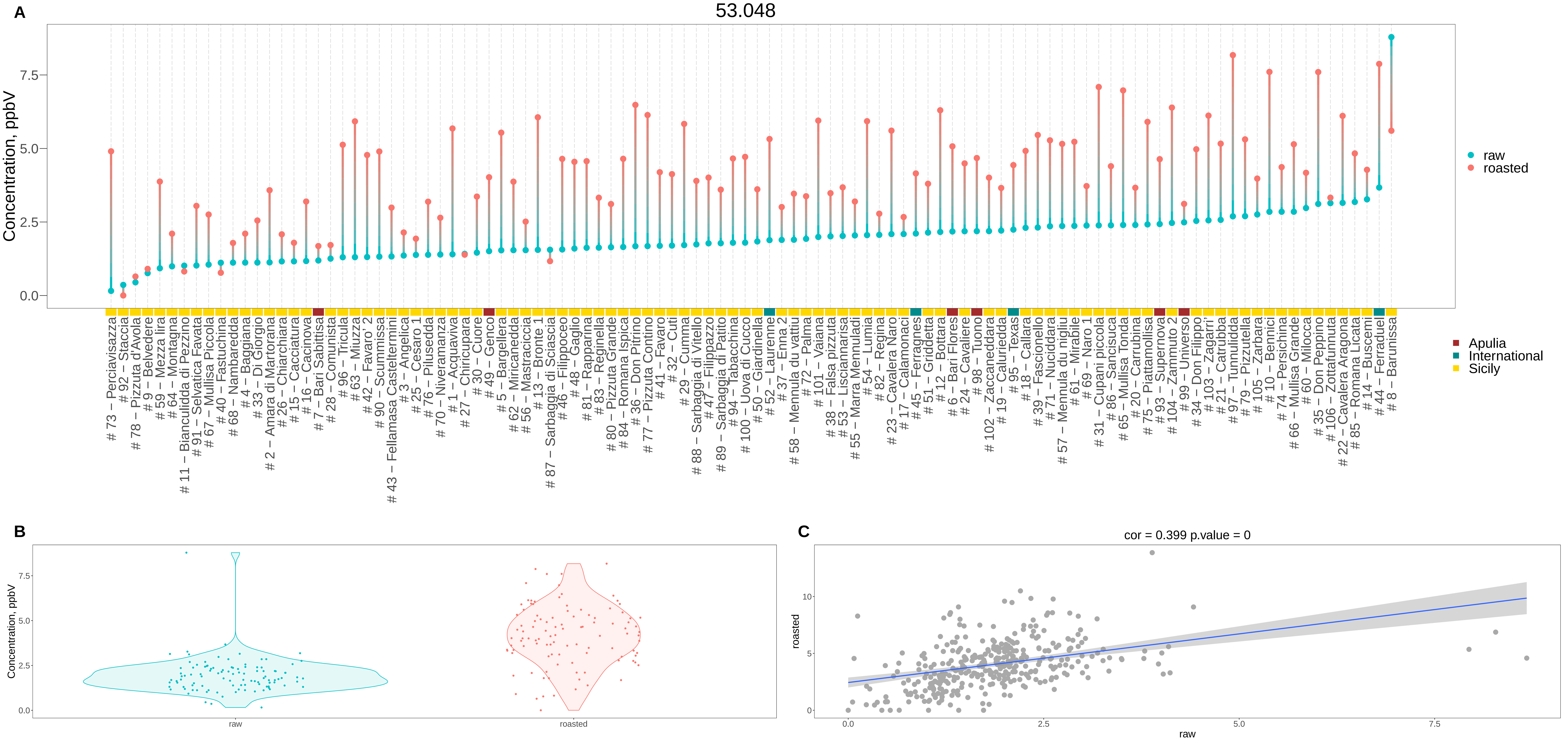
A
53.048
Concentration, ppbV
0.0
2.5
5.0
7.5
raw
roasted
Apulia
International
Sicily
73 – Perciavisazza
92 – Staccia
78 – Pizzuta d'Avola
9 – Belvedere
59 – Mezza lira
64 – Montagna
11 – Bianculidda di Pezzino
91 – Selvatica Favata
67 – Mullisa Piccola
40 – Fastuchina
68 – Nambaredda
4 – Baggiana
33 – Di Giorgio
2 – Amara di Martorana
26 – Chiarchiara
15 – Cacciatura
16 – Cacinova
7 – Bari Sabittisa
28 – Comunista
96 – Tricula
63 – Miuzza
42 – Favaro' 2
90 – Scummissa
43 – Fellamasa Castelltermini
3 – Angelica
25 – Cesaro 1
76 – Pilusedda
70 – Niveramanza
1 – Acquaviva
27 – Chiricupara
30 – Cuore
49 – Genco
5 – Bargellera
62 – Miricanedda
56 – Mastraciccia
13 – Bronte 1
87 – Sarbaggia di Sciascia
46 – Filippoceo
48 – Gaglio
81 – Rapparina
83 – Reginella
80 – Pizzuta Grande
84 – Romana Ispica
36 – Don Pitrino
77 – Pizzuta Contino
41 – Favaro
32 – Cuti
29 – Cumma
88 – Sarbaggia di Vitello
47 – Filippazzo
89 – Sarbaggia di Patito
94 – Tabacchina
100 – Uova di Cucco
50 – Giardinella
52 – Laurenne
37 – Enna 2
58 – Mennula du vattiu
72 – Palma
101 – Vaiana
38 – Falsa pizzuta
53 – Liscianarisa
55 – Marra Mennuladi
54 – Lumia
82 – Regina
23 – Cavalera Naro
17 – Calamonaci
45 – Ferragnes
51 – Griddetta
12 – Bottara
6 – Bari Flores
24 – Cavaliere
98 – Tuono
102 – Zaccaneddara
19 – Caluriedda
95 – Texas
18 – Callara
39 – Fascionello
71 – Nuciddara
57 – Mennula du nigliu
61 – Mirabile
69 – Naro 1
31 – Cupani piccola
86 – Sancisuca
65 – Mullisa Tonda
20 – Carrubina
75 – Piattamollisa
93 – Supernova
104 – Zammuto 2
99 – Universo
34 – Don Filippo
103 – Zagarri'
21 – Catrubba
97 – Tunnulidda
79 – Pizzutella
105 – Zarbara
10 – Bennici
74 – Persichina
66 – Mullisa Grande
60 – Milocca
35 – Don Peppino
106 – Zottafunnuta
22 – Cavalera Aragona
85 – Romana Licata
14 – Buscemi
44 – Ferraduel
8 – Barunissa
B
Concentration, ppbV
0.0
2.5
5.0
7.5
raw
roasted
C
cor = 0.399 p.value = 0
roasted
0
5
10
raw
0.0
2.5
5.0
7.5

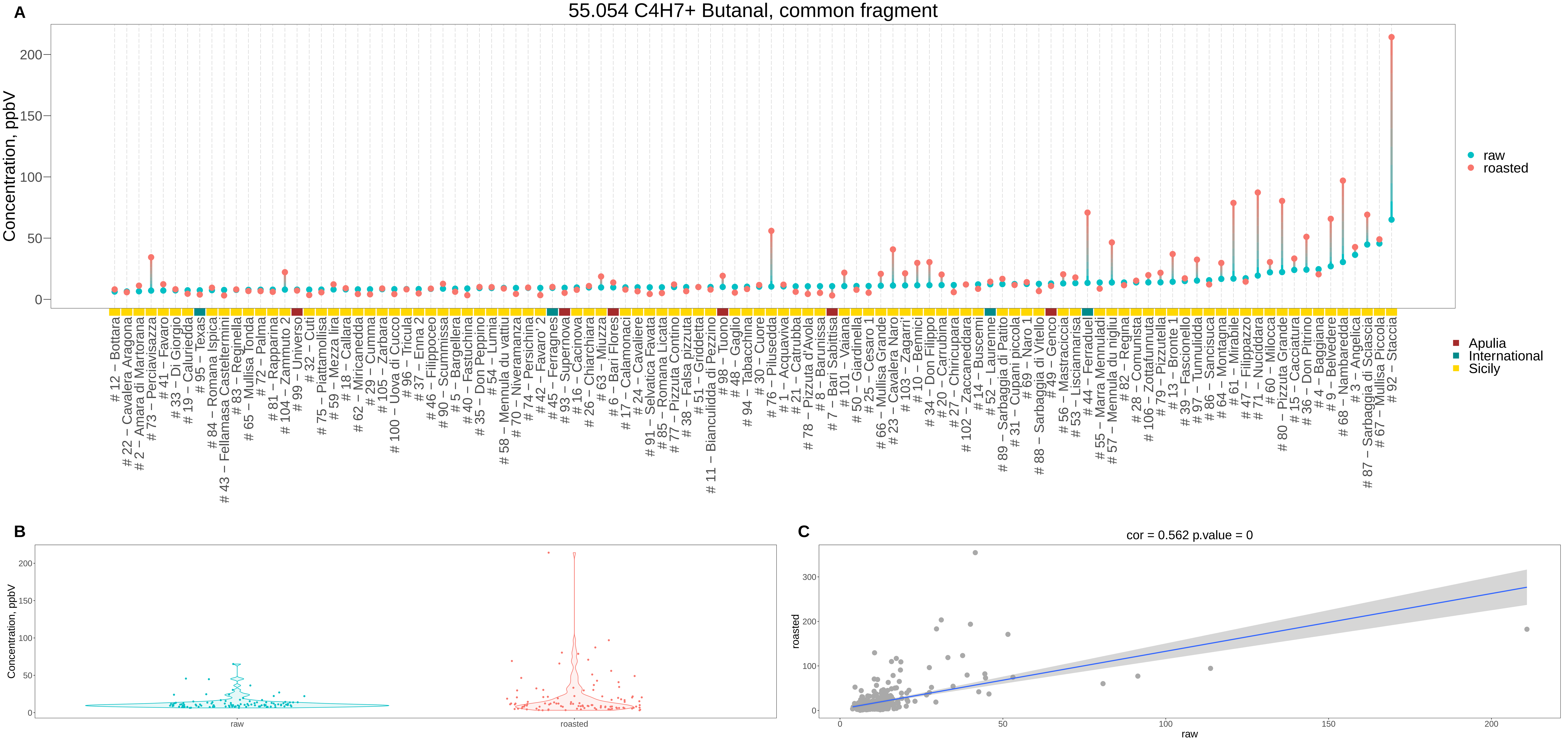

A
55.054 C4H7+ Butanal, common fragment
Concentration, ppbV
0
50
100
150
200
raw
roasted
Apulia
International
Sicily
12 – Bottara
22 – Cavalera Aragona
2 – Amara di Martorana
73 – Perciavisazza
41 – Favaro
33 – Di Giorgio
19 – Caluriedda
95 – Texas
84 – Romana Ispica
43 – Fellamasa Casteltermini
83 – Reginella
65 – Mullisa Tonda
72 – Palma
81 – Rapparina
104 – Zammuto 2
99 – Universo
32 – Cuti
75 – Piattamollisa
59 – Mezza lira
18 – Callara
62 – Miricanedda
29 – Cumma
105 – Zarbara
100 – Uova di Cucco
96 – Tricula
37 – Enna 2
46 – Filippoceo
90 – Scummissa
5 – Bargellera
40 – Fastuchina
35 – Don Peppino
54 – Lumia
58 – Mennula du vattiu
70 – Niveramanza
74 – Persichina
42 – Favaro' 2
45 – Ferragnes
93 – Supernova
16 – Cacinova
26 – Chiarchiara
63 – Miuzza
6 – Bari Flores
17 – Calamonaci
24 – Cavaliere
91 – Selvatica Favata
85 – Romana Licata
77 – Pizzuta Contino
38 – Falsa pizzuta
51 – Griddetta
11 – Bianculidda di Pezzino
98 – Tuono
48 – Gaglio
94 – Tabacchina
30 – Cuore
76 – Pilusedda
1 – Acquaviva
21 – Catrubba
78 – Pizzuta d'Avola
8 – Barunissa
7 – Bari Sabittisa
101 – Vaiana
50 – Giardinella
25 – Cesaro 1
66 – Mullisa Grande
23 – Cavalera Naro
103 – Zagarri'
10 – Bennici
34 – Don Filippo
20 – Carrubina
27 – Chiricupara
102 – Zaccaneddara
14 – Buscemi
52 – Laurenne
89 – Sarbaggia di Patito
31 – Cupani piccola
69 – Naro 1
88 – Sarbaggia di Vitello
49 – Genco
56 – Mastracicia
53 – Liscianarisa
44 – Ferraduel
55 – Marra Mennuladi
57 – Mennula du nigliu
82 – Regina
28 – Comunista
106 – Zottafunnuta
79 – Pizzutella
13 – Bronte 1
39 – Fascionello
97 – Tunnulidda
86 – Sancisuca
64 – Montagna
61 – Mirabile
47 – Filippazzo
71 – Nuciddara
60 – Milocca
80 – Pizzuta Grande
15 – Cacciatura
36 – Don Pitrino
4 – Baggiana
9 – Belvedere
68 – Nambaredda
3 – Angelica
87 – Sarbaggia di Sciascia
67 – Mullisa Piccola
92 – Staccia
B
Concentration, ppbV
0
50
100
150
200
raw
roasted
C
cor = 0.562 p.value = 0
roasted
0
100
200
300
0
50
100
150
200
raw

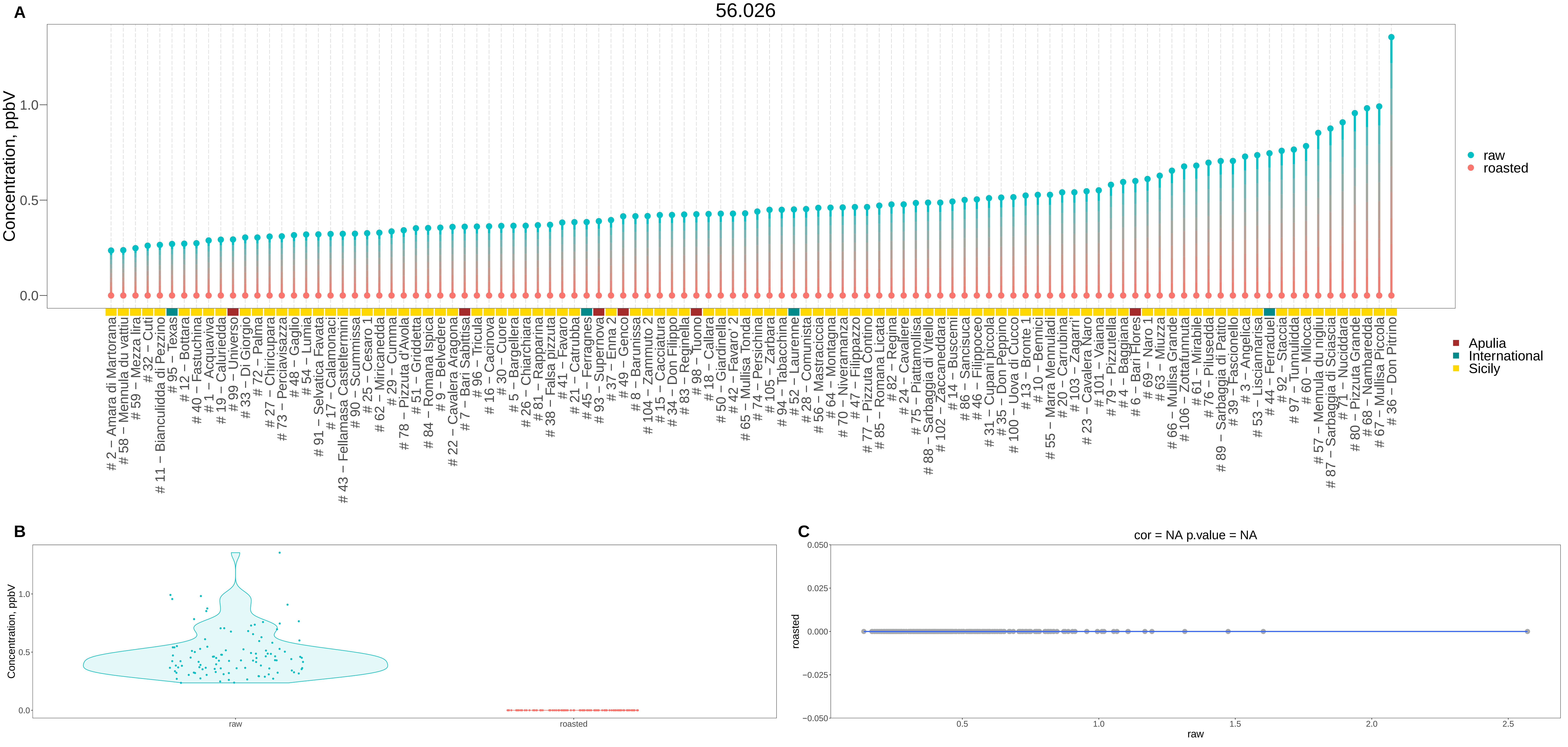

A
56.026
Concentration, ppbV
raw
roasted
Apulia
International
Sicily
B
Concentration, ppbV
raw
roasted
C
cor = NA p.value = NA
roasted
raw

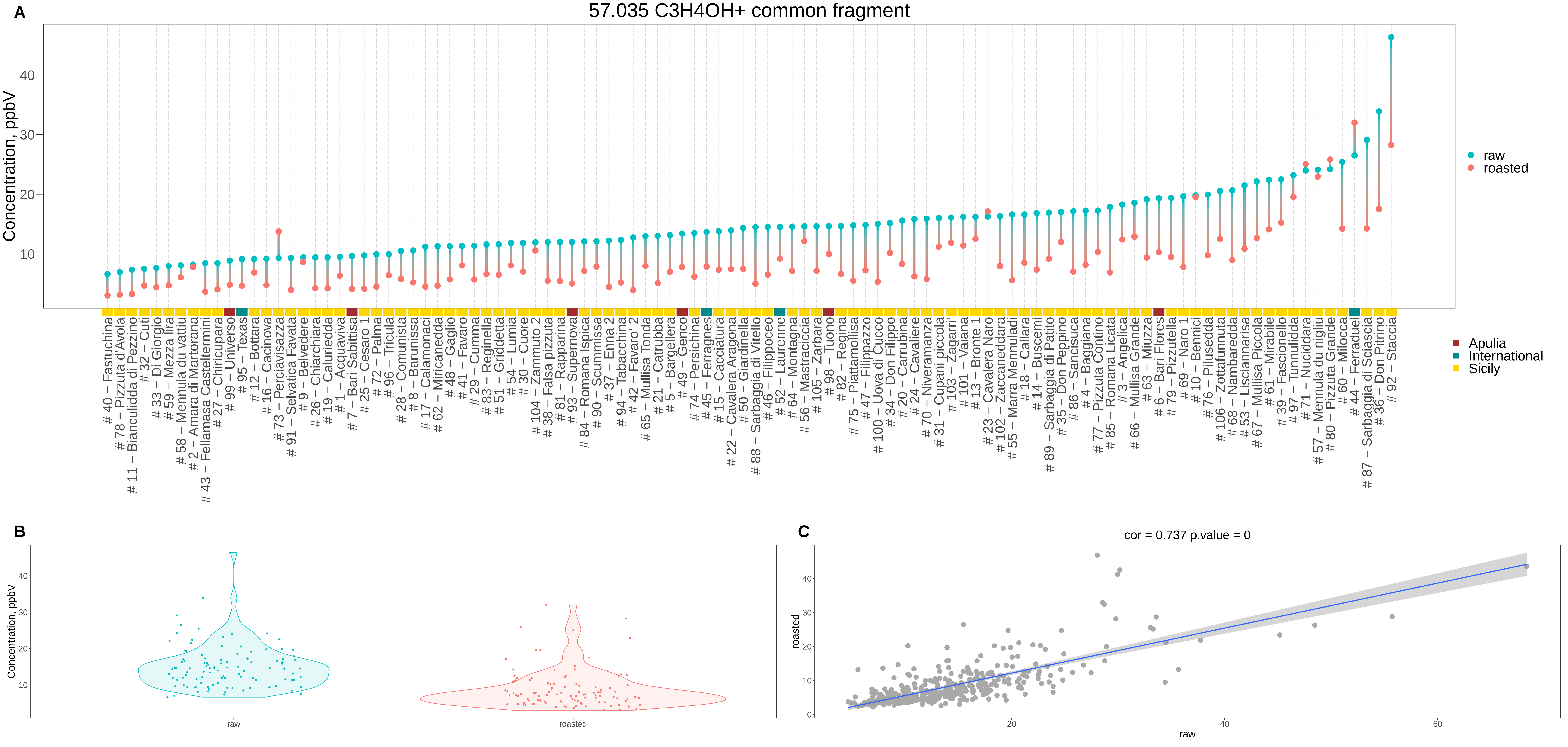
A
57.035 C3H4OH+ common fragment
Concentration, ppbV
10
20
30
40
raw
roasted
Apulia
International
Sicily
40 – Fastuchina
78 – Pizzuta d'Avola
11 – Bianculidda di Pezzino
32 – Cuti
33 – Di Giorgio
59 – Mezza lira
58 – Mennula du vattiu
2 – Amara di Martorana
43 – Fellamasa Casteltermini
27 – Chiricupara
99 – Universo
95 – Texas
12 – Bottara
16 – Cacinova
73 – Perciavisazza
91 – Selvatica Favata
9 – Belvedere
26 – Chiarchiara
19 – Caluriedda
1 – Acquaviva
7 – Bari Sabittisa
25 – Cesaro 1
72 – Palma
96 – Tricula
28 – Comunista
8 – Barunissa
17 – Calamonaci
62 – Miricanedda
48 – Gaglio
41 – Favaro
29 – Cumma
83 – Reginella
51 – Griddetta
54 – Lumia
30 – Cuore
104 – Zammuto 2
38 – Falsa pizzuta
81 – Rapparina
93 – Supernova
84 – Romana Ispica
90 – Scummissa
37 – Enna 2
94 – Tabacchina
42 – Favaro' 2
65 – Mullisa Tonda
21 – Catrubba
5 – Bargellera
49 – Genco
74 – Persichina
45 – Ferragnes
15 – Cacciatura
22 – Cavalera Aragona
50 – Giardinella
88 – Sarbaggia di Vitello
46 – Filippoceo
52 – Laurenne
64 – Montagna
56 – Mastraciccia
105 – Zarbara
98 – Tuono
82 – Regina
75 – Piattamollisa
47 – Filippazzo
100 – Uova di Cucco
34 – Don Filippo
20 – Carrubina
24 – Cavaliere
70 – Niveramanza
31 – Cupani piccola
103 – Zagarri'
101 – Vaiana
13 – Bronte 1
23 – Cavalera Naro
102 – Zaccaneddari
55 – Marra Mennuladi
18 – Callara
14 – Buscemi
89 – Sarbaggia di Patito
35 – Don Peppino
86 – Sancisuca
4 – Baggiana
77 – Pizzuta Contino
85 – Romana Licata
3 – Angelica
66 – Mullisa Grande
63 – Miuzza
6 – Bari Flores
79 – Pizzutella
69 – Naro 1
10 – Bennici
76 – Pilusedda
106 – Zottafunnuta
68 – Nambaredda
53 – Liscianarisa
67 – Mullisa Piccola
61 – Mirabile
39 – Fascionello
97 – Tunnulidda
71 – Nuciddara
57 – Mennula du nigliu
80 – Pizzuta Grande
60 – Milocca
44 – Ferraduel
87 – Sarbaggia di Sciascia
36 – Don Pitrino
92 – Staccia
B
Concentration, ppbV
10
20
30
40
raw
roasted
C
cor = 0.737 p.value = 0
roasted
0
10
20
30
40
raw
20
40
60

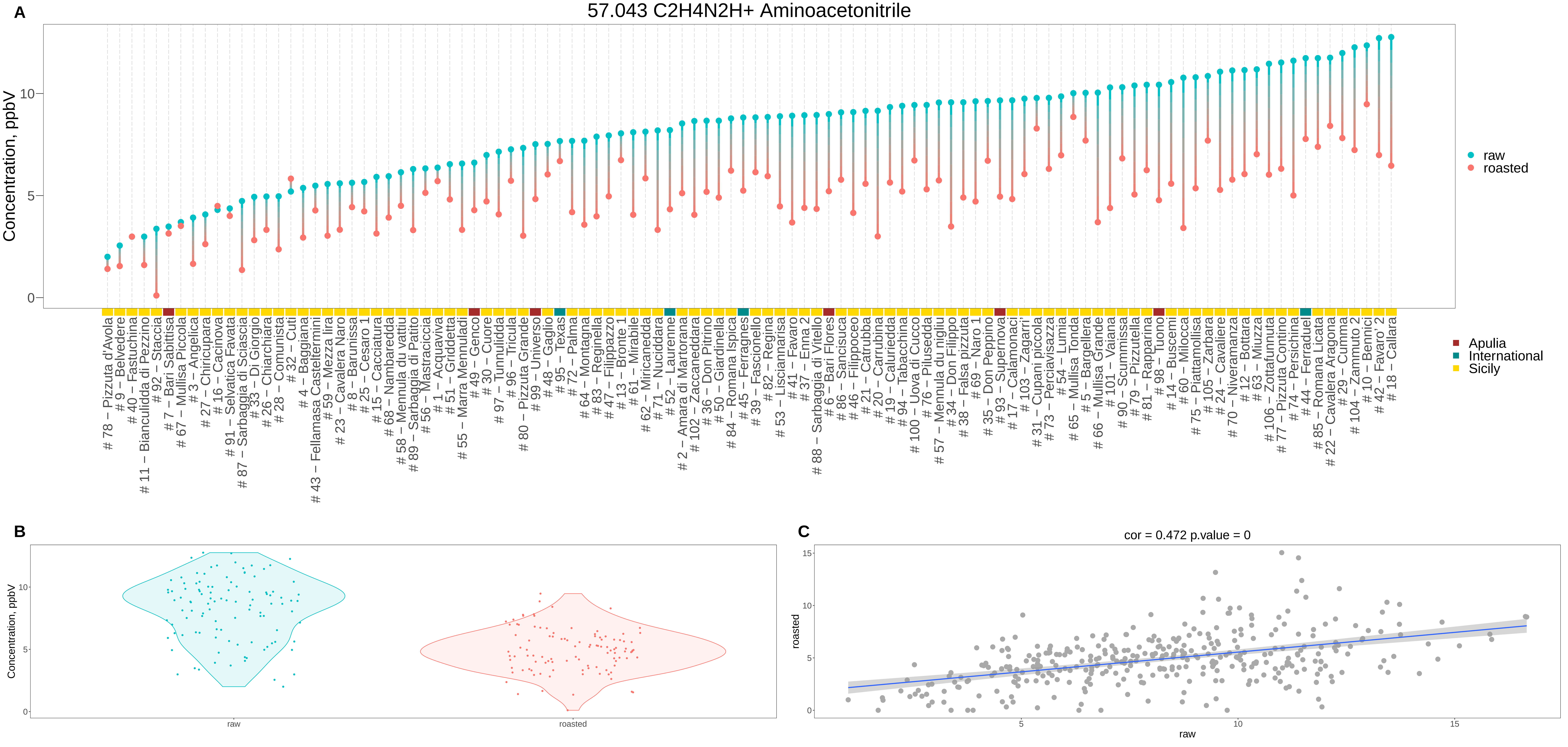
A
57.043 C2H4N2H+ Aminoacetonitrile
Concentration, ppbV
0
5
10
raw
roasted
Apulia
International
Sicily
78 – Pizzuta d'Avola
9 – Belvedere
40 – Fastuchina
11 – Bianculidda di Pezzino
92 – Staccia
7 – Bari Sabitisa
67 – Mullisa Piccola
3 – Angelica
27 – Chiricupara
16 – Cacinova
91 – Selvatica Favata
87 – Sarbaggia di Sciascia
33 – Di Giorgio
26 – Chiarchiara
28 – Comunista
32 – Cuti
4 – Baggiana
43 – Fellamasa Casteltermini
59 – Mezza lira
23 – Cavalera Naro
8 – Barunissa
25 – Cesaro 1
15 – Cacciatura
68 – Nambaredda
58 – Mennula du vattiu
89 – Sarbaggia di Patito
56 – Mastraciccia
1 – Acquaviva
51 – Griddetta
55 – Marra Mennuladi
49 – Genco
30 – Cuore
97 – Tunnulidda
96 – Tricula
80 – Pizzuta Grande
99 – Universo
48 – Gaglio
95 – Texas
72 – Palma
64 – Montagna
83 – Reginella
47 – Filippazzo
13 – Bronte 1
61 – Mirabile
62 – Miricanedda
71 – Nuciddara
52 – Laurenne
2 – Amara di Martorana
102 – Zaccaneddara
36 – Don Pitrino
50 – Giardinella
84 – Romana Ispica
45 – Ferragnes
39 – Fascionello
82 – Regina
53 – Lisciannarisa
41 – Favaro
37 – Enna 2
88 – Sarbaggia di Vitello
6 – Bari Flores
86 – Sancisuca
46 – Filippoceo
21 – Catrubba
20 – Carrubina
19 – Caluriedda
94 – Tabacchina
100 – Uova di Cucco
76 – Pilusedda
57 – Mennula du nigliu
34 – Don Filippo
38 – Falsa pizzuta
69 – Naro 1
35 – Don Peppino
93 – Supernova
17 – Calamonaci
103 – Zagarri
31 – Cupani piccola
73 – Perciavisazza
54 – Lumia
65 – Mullisa Tonda
5 – Bargellera
66 – Mullisa Grande
101 – Vaiana
90 – Scummissa
79 – Pizzutella
81 – Rapparina
98 – Tuono
14 – Buscemi
60 – Milocca
75 – Piattamollisa
105 – Zarbara
24 – Cavaliere
70 – Niveramanza
12 – Bottara
63 – Miuzza
106 – Zottafunnuta
77 – Pizzuta Contino
74 – Persichina
44 – Ferraduel
85 – Romana Licata
22 – Cavalera Aragona
29 – Cumma
104 – Zammuto 2
10 – Bennici
42 – Favaro' 2
18 – Callara
B
Concentration, ppbV
0
5
10
raw
roasted
C
cor = 0.472 p.value = 0
roasted
0
5
10
15
raw
5
10
15

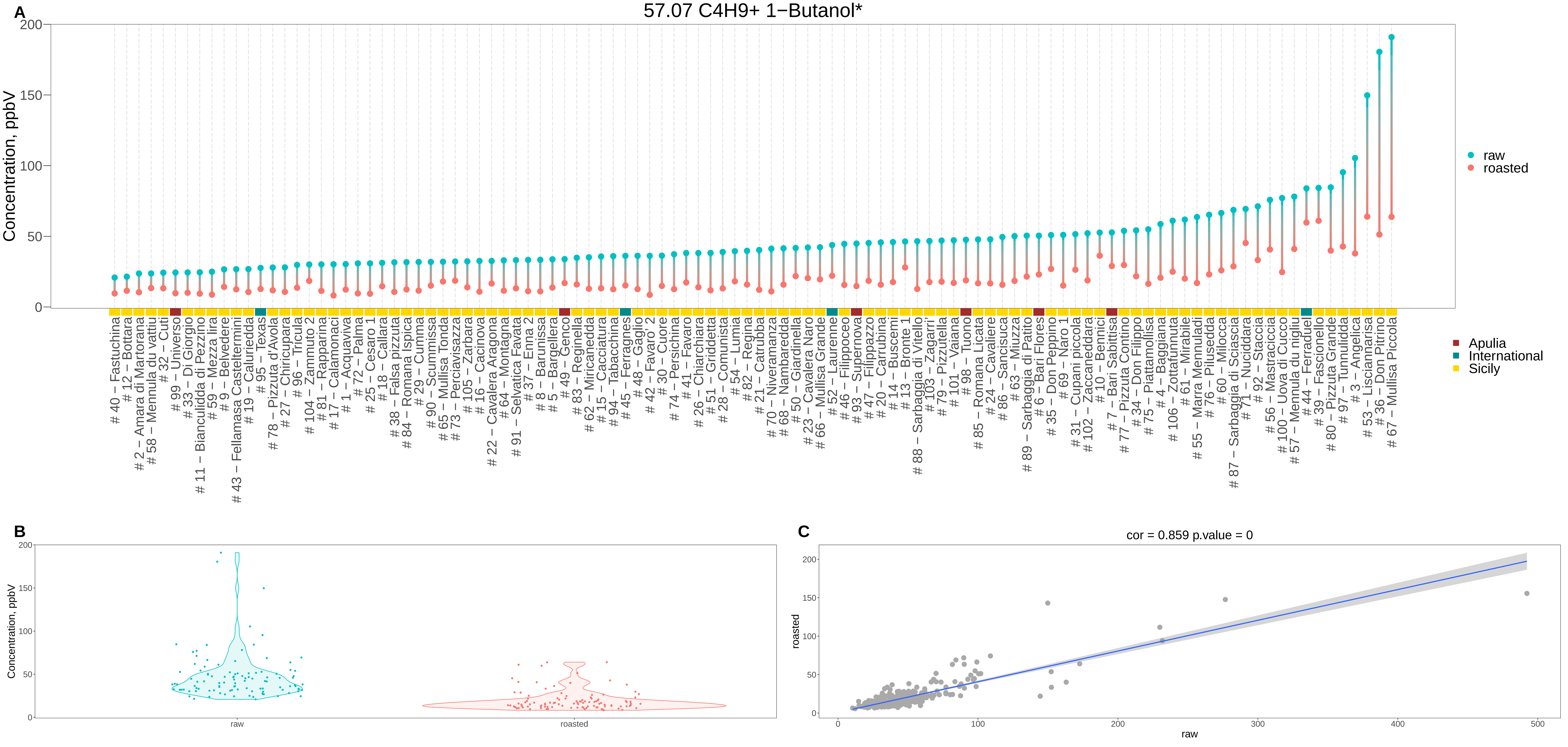

A
57.07 C4H9+ 1−Butanol*
Concentration, ppbV
0
50
100
150
200
raw
roasted
Apulia
International
Sicily
40 − Fastuchina
12 − Bottara
2 − Amara di Martorana
58 − Mennula du vattiu
32 − Cuti
99 − Universo
33 − Di Giorgio
11 − Bianculidda di Pezzino
59 − Mezza lira
9 − Belvedere
43 − Fellamasa Casteltermini
19 − Caluriedda
95 − Texas
78 − Pizzuta d'Avola
27 − Chiricupara
96 − Tricula
104 − Zammuto 2
81 − Rapparina
17 − Calamonaci
1 − Acquaviva
72 − Palma
25 − Cesaro 1
18 − Callara
38 − Falsa pizzuta
84 − Romana Ispica
29 − Cumma
90 − Scummissa
65 − Mullisa Tonda
73 − Perciavisazza
105 − Zarbara
16 − Cacinova
22 − Cavalera Aragona
64 − Montagna
91 − Selvatica Favata
37 − Enna 2
8 − Barunissa
5 − Bargellera
49 − Genco
83 − Reginella
62 − Miricanedda
15 − Cacciatura
94 − Tabacchina
45 − Ferragnes
48 − Gaglio
42 − Favaro' 2
30 − Cuore
74 − Persichina
41 − Favaro
26 − Chiarchiara
51 − Griddetta
28 − Comunista
54 − Lumia
82 − Regina
21 − Catrubba
70 − Niveramanza
68 − Nambaredda
50 − Giardinella
23 − Cavalera Naro
66 − Mullisa Grande
52 − Laurenne
46 − Filippoceo
93 − Supernova
47 − Filippazzo
20 − Carrubina
14 − Buscemi
13 − Bronte 1
88 − Sarbaggia di Vitello
103 − Zagarri'
79 − Pizzutella
101 − Vaiana
98 − Tuono
85 − Romana Licata
24 − Cavaliere
86 − Sancisuca
63 − Miuzza
89 − Sarbaggia di Patito
6 − Bari Flores
35 − Don Peppino
69 − Naro 1
31 − Cupani piccola
102 − Zaccaneddara
10 − Bennici
7 − Bari Sabittisa
77 − Pizzuta Contino
34 − Don Filippo
75 − Piattamollisa
4 − Baggiana
106 − Zottafunnuta
61 − Mirabile
55 − Marra Mennuladi
76 − Pilusedda
60 − Milocca
87 − Sarbaggia di Sciascia
71 − Nuciddara
92 − Staccia
56 − Mastraciccia
100 − Uova di Cucco
57 − Mennula du nigliu
44 − Ferraduel
39 − Fascionello
80 − Pizzuta Grande
97 − Tunnulidda
3 − Angelica
53 − Liscianarisa
36 − Don Pitrino
67 − Mullisa Piccola
B
Concentration, ppbV
0
50
100
150
200
raw
roasted
C
cor = 0.859 p.value = 0
roasted
0
50
100
150
200
raw
0
100
200
300
400
500

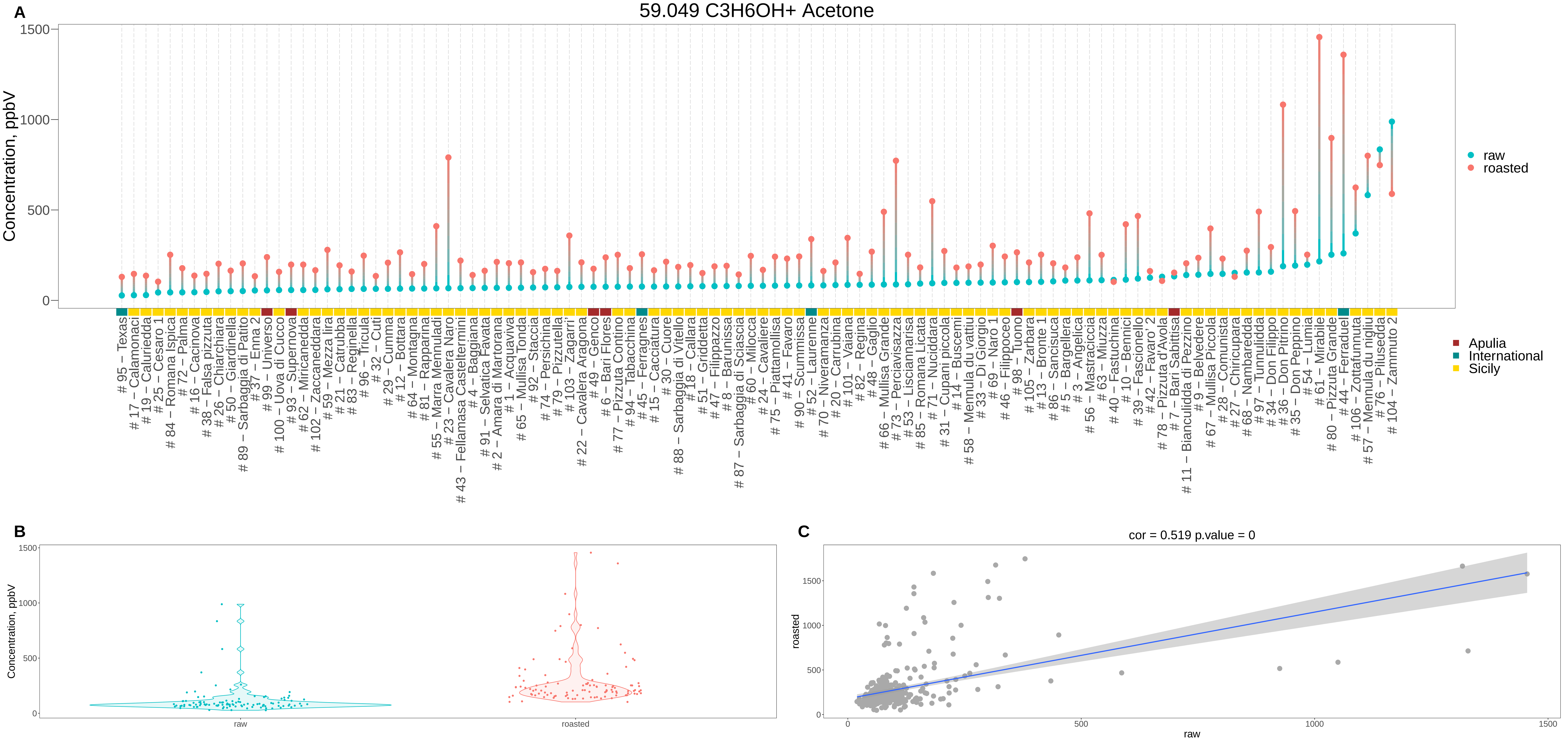

A
59.049 C3H6OH+ Acetone
Concentration, ppbV
1500
1000
500
0
raw
roasted
Apulia
International
Sicily
95 – Texas
17 – Calamonaci
19 – Caluriedda
25 – Cesaro 1
84 – Romana Ispica
72 – Palma
16 – Cacinova
38 – Falsa pizzuta
26 – Chiarchiara
50 – Giardinella
89 – Sarbaggia di Patito
37 – Enna 2
99 – Universo
100 – Uova di Cucco
93 – Supernova
62 – Miricanedda
102 – Zaccaneddara
59 – Mezza lira
21 – Catrubba
83 – Reginella
96 – Tricula
32 – Cuti
29 – Cumma
12 – Bottara
64 – Montagna
81 – Rapparina
55 – Marra Mennuladi
23 – Cavalera Naro
43 – Fellamasa Casteltermini
4 – Baggiana
91 – Selvatica Favata
2 – Amara di Martorana
1 – Acquaviva
65 – Mullisa Tonda
92 – Staccia
74 – Persichina
79 – Pizzutella
103 – Zagarri'
22 – Cavalera Aragona
49 – Genco
6 – Bari Flores
77 – Pizzuta Contino
94 – Tabacchina
45 – Ferragnes
15 – Cacciatura
30 – Cuore
88 – Sarbaggia di Vitello
18 – Callara
51 – Griddetta
47 – Filippazzo
8 – Barunissa
87 – Sarbaggia di Sciascia
60 – Milocca
24 – Cavaliere
75 – Piattamollisa
41 – Favaro
90 – Scummissa
52 – Laurenne
70 – Niveramanza
20 – Carrubina
101 – Vaiana
82 – Regina
48 – Gaglio
66 – Mullisa Grande
73 – Perciavisazza
53 – Liscianarisa
85 – Romana Licata
71 – Nuciddara
31 – Cupani piccola
14 – Buscemi
58 – Mennula du vattiu
33 – Di Giorgio
69 – Naro 1
46 – Filippoceo
98 – Tuono
105 – Zarbara
13 – Bronte 1
86 – Sancisuca
5 – Bargellera
3 – Angelicia
56 – Mastraciccia
63 – Miuzza
40 – Fastuchina
10 – Bennici
39 – Fascionello
42 – Favaro' 2
78 – Pizzuta d'Avola
7 – Bari Sabittisa
11 – Biancuilidda di Pezzino
9 – Belvedere
67 – Mullisa Piccola
28 – Comunista
27 – Chiricupara
68 – Nambaredda
97 – Tunnulidda
34 – Don Filippo
36 – Don Pitrino
35 – Don Peppino
54 – Lumia
61 – Mirabile
80 – Pizzuta Grande
44 – Ferraduel
106 – Zottafunnuta
57 – Mennula du nigliu
76 – Pilusedda
104 – Zammuto 2
B
Concentration, ppbV
1500
1000
500
0
raw
roasted
C
cor = 0.519 p.value = 0
roasted
1500
1000
500
0
0
500
1000
1500
raw

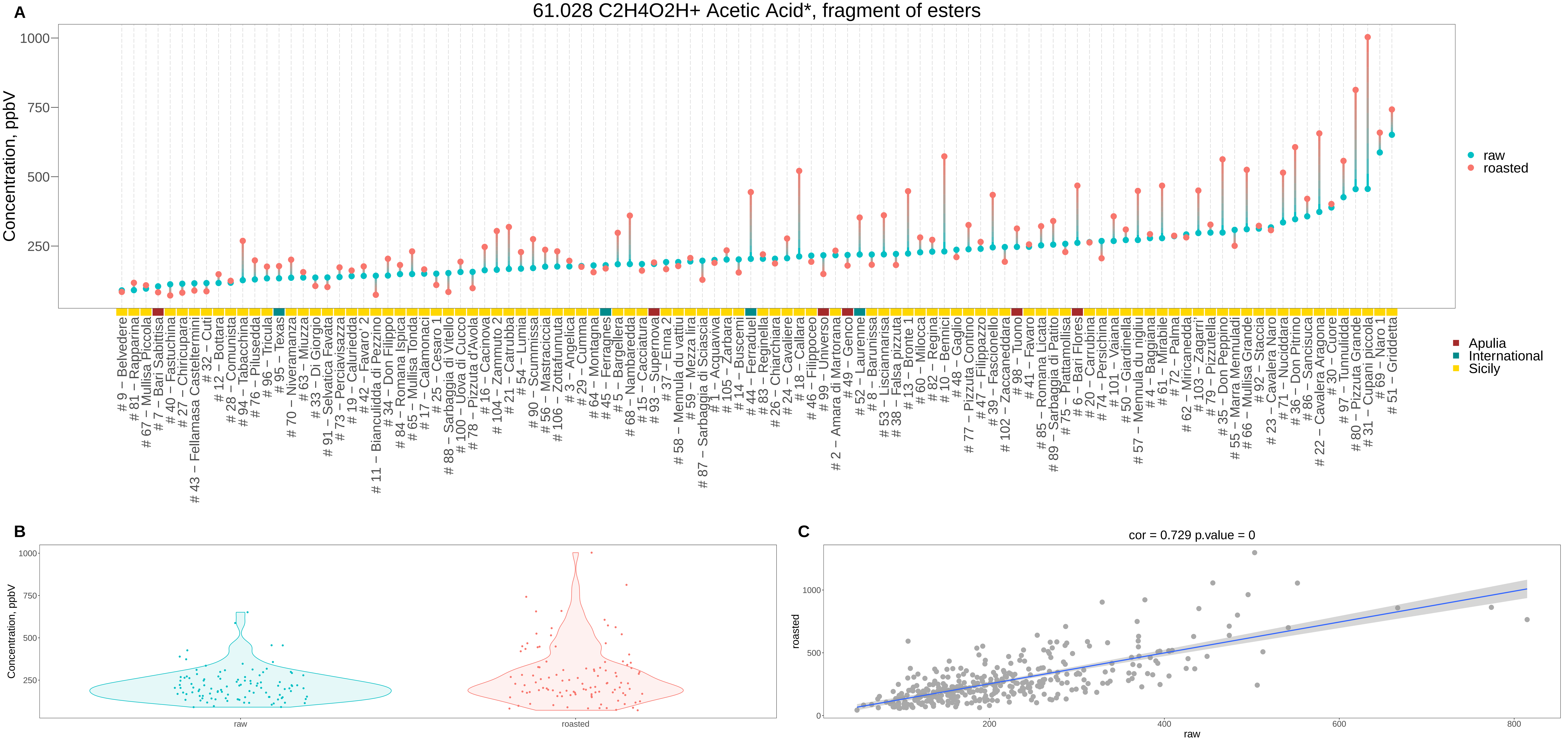
A
61.028 C2H4O2H+ Acetic Acid*, fragment of esters
Concentration, ppbV
1000
750
500
250
raw
roasted
Apulia
International
Sicily
9 – Belvedere
81 – Rapparina
67 – Mullisa Piccola
7 – Bari Sabittisa
40 – Fastuchina
27 – Chiricupara
43 – Fellamasa Casteltermini
32 – Cuti
12 – Bottara
28 – Comunista
94 – Tabacchina
76 – Pilusedda
96 – Tricula
95 – Texas
70 – Niveramanza
63 – Miuzza
33 – Di Giorgio
91 – Selvatica Favata
73 – Perciavisazza
19 – Caluriedda
42 – Favaro' 2
11 – Bianculidda di Pezzino
34 – Don Filippo
84 – Romana Ispica
65 – Mullisa Tonda
17 – Calamonaci
25 – Cesaro 1
88 – Sarbaggia di Vitello
100 – Uova di Cucco
78 – Pizzuta d'Avola
16 – Cacinova
104 – Zammuto 2
21 – Catrubba
54 – Lumia
90 – Scummissa
56 – Mastracicia
106 – Zottafunnuta
3 – Angelica
29 – Cumma
64 – Montagna
45 – Ferragnes
5 – Bargellera
68 – Nambaredda
15 – Cacciatura
93 – Supernova
37 – Enna 2
58 – Mennula du vattiu
59 – Mezza lira
87 – Sarbaggia di Sciascia
1 – Acquaviva
105 – Zarbara
14 – Buscemi
44 – Ferraduel
83 – Reginella
26 – Chiarchiara
24 – Cavaliere
18 – Callara
46 – Filippoceo
99 – Universo
2 – Amara di Martorana
49 – Genco
52 – Laurenne
8 – Barunissa
53 – Lisciannarisa
38 – Falsa pizzuta
13 – Bronte 1
60 – Milocca
82 – Regina
10 – Bennici
48 – Gaglio
77 – Pizzuta Contino
47 – Filippazzo
39 – Fascionello
102 – Zaccanedda
98 – Tuono
41 – Favaro
85 – Romana Licata
89 – Sarbaggia di Patito
75 – Piattamollisa
6 – Bari Flores
20 – Carrubina
74 – Persichina
101 – Vaiana
50 – Giardinella
57 – Mennula du nigliu
4 – Baggiana
61 – Mirabile
72 – Palma
62 – Miricanedda
103 – Zagarri'
79 – Pizzutella
35 – Don Peppino
55 – Marra Mennuladi
66 – Mullisa Grande
92 – Staccia
23 – Cavalera Naro
71 – Nuciddara
36 – Don Pitrino
86 – Sancisuca
22 – Cavalera Aragona
30 – Cuore
97 – Tunnulidda
80 – Pizzuta Grande
31 – Cupani piccola
69 – Naro 1
51 – Griddetta
B
Concentration, ppbV
1000
750
500
250
raw
roasted
C
cor = 0.729 p.value = 0
roasted
1000
500
0
raw
200
400
600
800

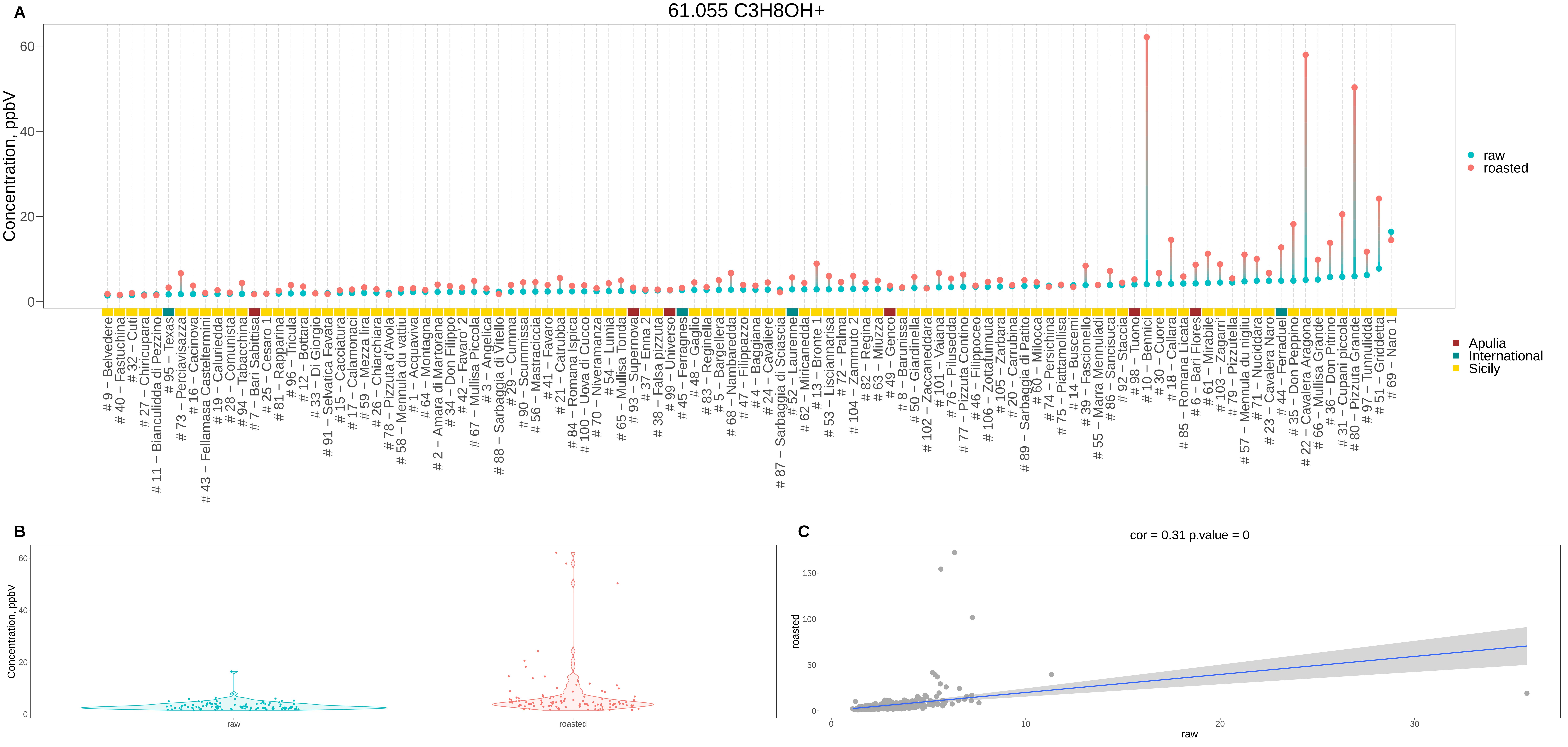

A
61.055 C3H8OH+
Concentration, ppbV
0
20
40
60
raw
roasted
Apulia
International
Sicily
9 – Belvedere
40 – Fastuchina
32 – Cuti
27 – Chiricupara
11 – Biancuilidda di Pezzino
95 – Texas
73 – Perciavisazza
16 – Cacinova
43 – Fellamasa Casteltermini
19 – Caluriedda
28 – Comunista
94 – Tabacchina
7 – Bari Sabittisa
25 – Cesaro 1
81 – Rapparina
96 – Tricula
12 – Bottara
33 – Di Giorgio
91 – Selvatica Favata
15 – Cacciatura
17 – Calamonaci
59 – Mezza lira
26 – Chiarchiara
78 – Pizzuta d'Avola
58 – Mennula du vattiu
1 – Acquaviva
64 – Montagna
2 – Amara di Martorana
34 – Don Filippo
42 – Favaro 2
67 – Mullisa Piccola
3 – Angelica
88 – Sarbaggia di Vitello
29 – Cummisa
90 – Scummissa
56 – Mastraciccia
41 – Favaro
21 – Catrubba
84 – Romana Ispica
100 – Uova di Cucco
70 – Niveramanza
54 – Lumia
65 – Mullisa Tonda
93 – Supernova
37 – Enna 2
38 – Falsa pizzuta
99 – Universo
45 – Ferragnes
48 – Gaglio
83 – Reginella
5 – Bargellera
68 – Nambaredda
47 – Filippazzo
4 – Baggiana
24 – Cavaliere
87 – Sarbaggia di Sciascia
52 – Laurenne
62 – Miricanedda
13 – Bronte 1
53 – Liscianarisa
72 – Palma
104 – Zammuto 2
82 – Regina
63 – Miuzza
49 – Genco
8 – Barunissa
50 – Giardinella
102 – Zaccaneddara
101 – Vaiana
76 – Pilusedda
77 – Pizzuta Contino
46 – Filippoceo
106 – Zottafumuta
105 – Zarbara
20 – Carrubina
89 – Sarbaggia di Patito
60 – Milocca
74 – Persichina
75 – Piattamollisa
14 – Buscemi
39 – Fascionello
55 – Marra Mennuladi
86 – Sancisuca
92 – Staccia
98 – Tuono
10 – Bennici
30 – Cuore
18 – Callara
85 – Romana Licata
6 – Bari Flores
61 – Mirabile
103 – Zagarri
79 – Pizzutella
57 – Mennula du nigliu
71 – Nuciddara
23 – Cavalera Naro
44 – Ferraduel
35 – Don Peppino
22 – Cavalera Aragona
66 – Mullisa Grande
36 – Don Pitrino
31 – Cupani piccola
80 – Pizzuta Grande
97 – Tunnulidda
51 – Griddetta
69 – Naro 1
B
Concentration, ppbV
0
20
40
60
raw
roasted
C
cor = 0.31 p.value = 0
roasted
0
50
100
150
raw
0
10
20
30

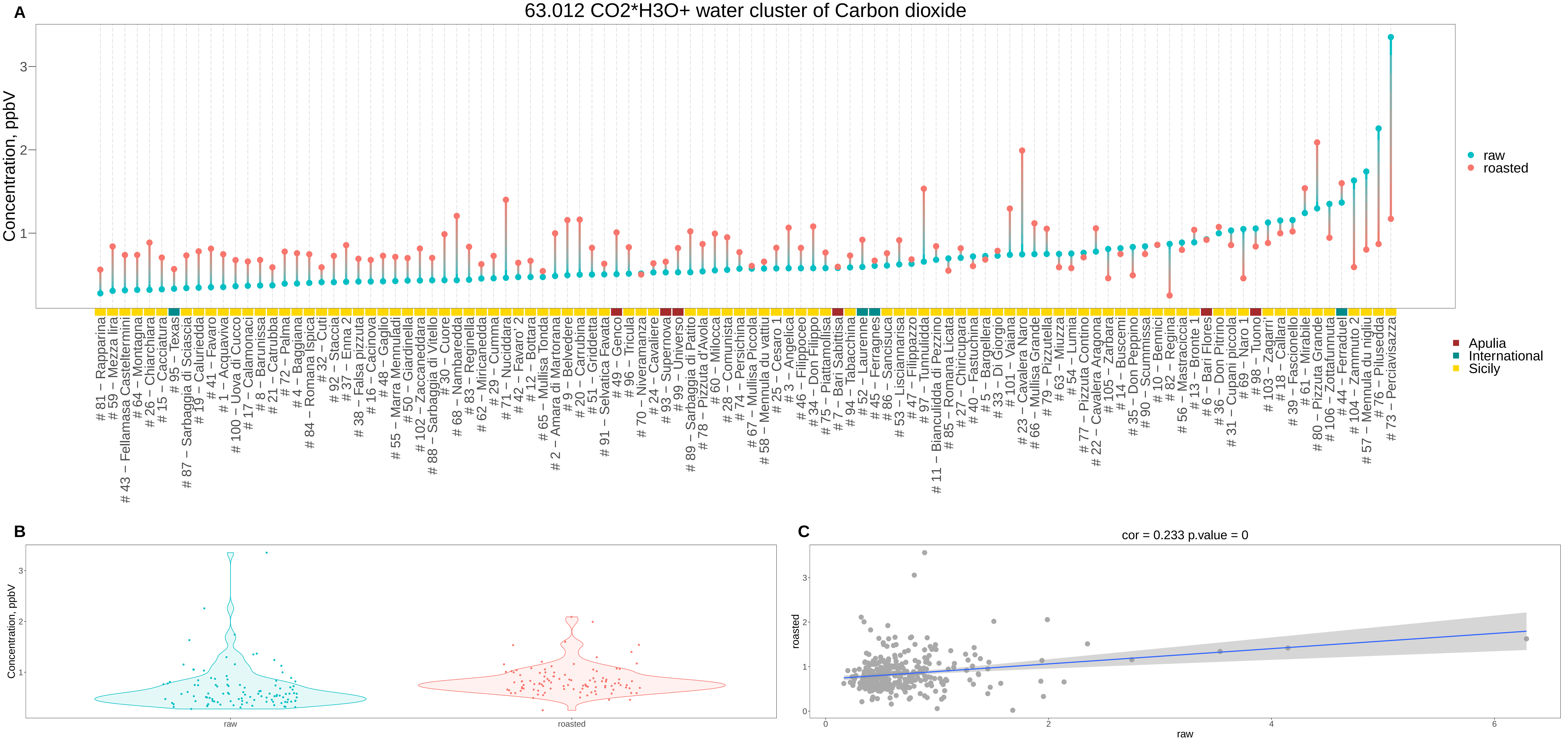
A
63.012 CO2*H3O+ water cluster of Carbon dioxide
Concentration, ppbV
raw
roasted
Apulia
International
Sicily
B
Concentration, ppbV
raw
roasted
C
cor = 0.233 p.value = 0
roasted
raw

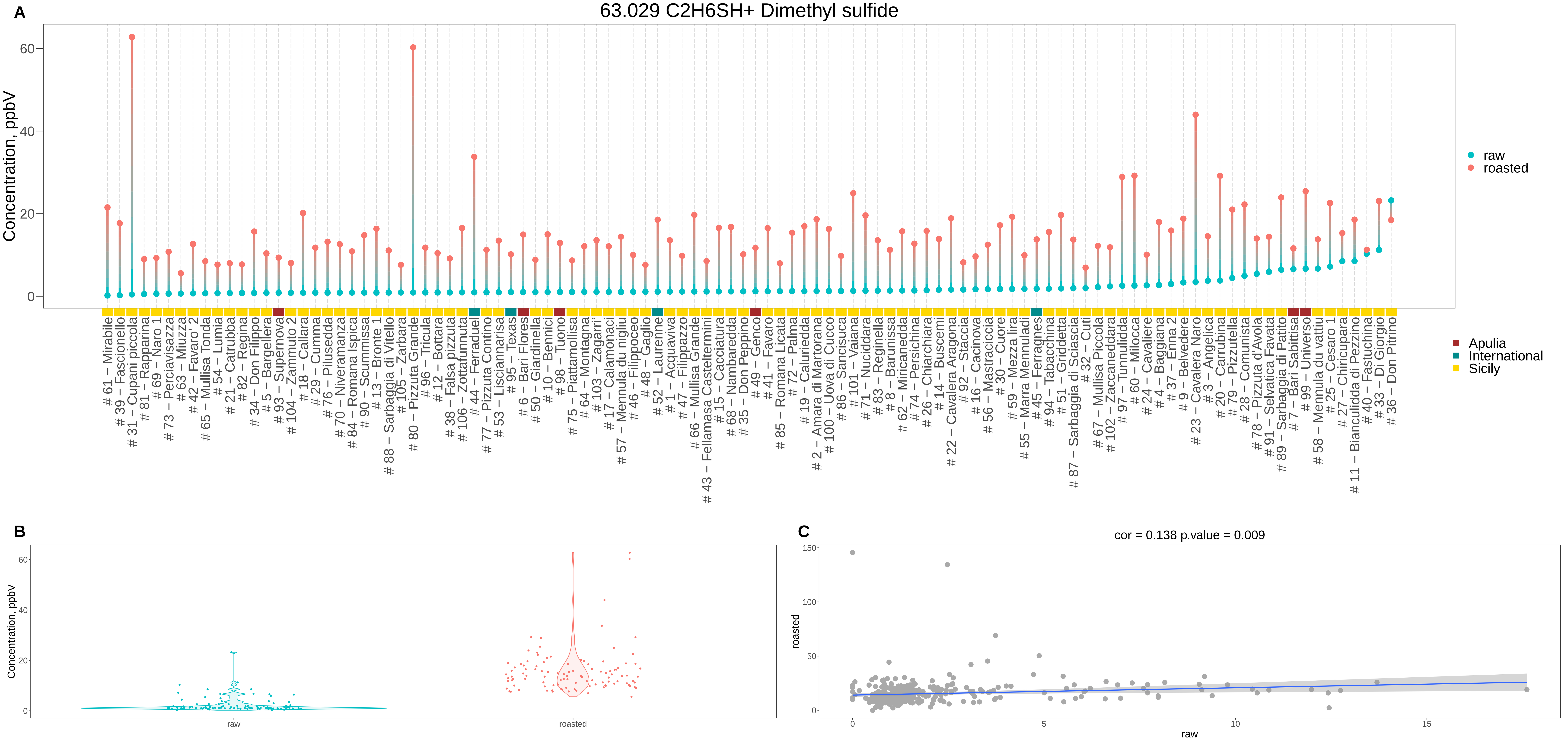

A
63.029 C2H6SH+ Dimethyl sulfide
Concentration, ppbV
0
20
40
60
raw
roasted
Apulia
International
Sicily
61 – Mirabile
39 – Fascionello
31 – Cupani piccola
81 – Rapparina
69 – Naro 1
73 – Perciavisazza
63 – Miuzza
42 – Favaro' 2
65 – Mullisa Tonda
54 – Lumia
21 – Catrubba
82 – Regina
34 – Don Filippo
5 – Bargellera
93 – Supernova
104 – Zammuto 2
18 – Callara
29 – Cumma
76 – Pilusedda
70 – Niveramanza
84 – Romana Ispica
90 – Scummissa
13 – Bronte 1
88 – Sarbaggia di Vitello
105 – Zarbara
80 – Pizzuta Grande
96 – Tricula
12 – Bottara
38 – Falsa pizzuta
106 – Zottafunnuta
44 – Ferraduel
77 – Pizzuta Contino
53 – Lisciannarisa
95 – Texas
6 – Bari Flores
50 – Giardinella
10 – Bennici
98 – Tuono
75 – Piattamollisa
64 – Montagna
103 – Zagarri'
17 – Calamonaci
57 – Mennula du nigliu
46 – Filippoceo
48 – Gaglio
52 – Laurenne
1 – Acquaviva
47 – Filippazzo
66 – Mullisa Grande
43 – Fellamasa Casteltermini
15 – Cacciatura
68 – Nambaredda
35 – Don Peppino
49 – Genco
41 – Favaro
85 – Romana Licata
72 – Palma
19 – Caluriedda
2 – Amara di Martorana
100 – Uova di Cucco
86 – Sancisuca
101 – Vaiana
71 – Nuciddara
83 – Reginella
8 – Barunissa
62 – Miricanedda
74 – Persichina
26 – Chiarchiara
14 – Buscemi
22 – Cavalera Aragona
92 – Staccia
16 – Cacinova
56 – Mastraciccia
30 – Cuore
59 – Mezza lira
55 – Marra Mennuladi
45 – Ferragnes
94 – Tabacchina
51 – Griddetta
87 – Sarbaggia di Sciascia
32 – Cuti
67 – Mullisa Piccola
102 – Zaccaneddara
97 – Tunnulidda
60 – Milocca
24 – Cavaliere
4 – Baggiana
37 – Enna 2
9 – Belvedere
23 – Cavalera Naro
3 – Angelica
20 – Carrubina
79 – Pizzutella
28 – Comunista
78 – Pizzuta d'Avola
91 – Selvatica Favata
89 – Sarbaggia di Patito
7 – Bari Sabitisa
99 – Universo
58 – Mennula du vattiu
25 – Cesaro 1
27 – Chiricupara
11 – Bianculidda di Pezzino
40 – Fastuchina
33 – Di Giorgio
36 – Don Pitrino
B
Concentration, ppbV
0
20
40
60
raw
roasted
C
cor = 0.138 p.value = 0.009
roasted
0
50
100
150
raw
0
5
10
15

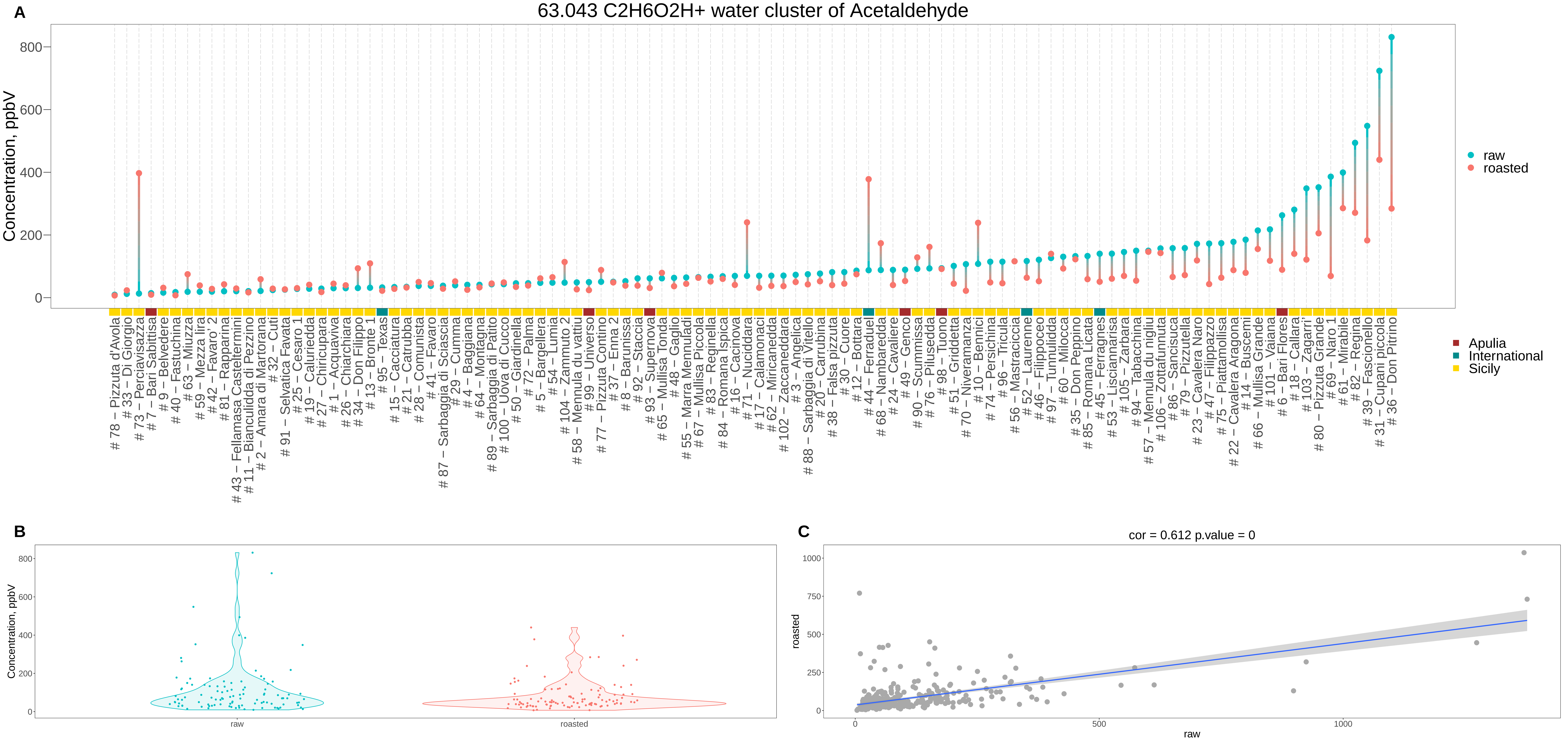
A
63.043 C2H6O2H+ water cluster of Acetaldehyde
Concentration, ppbV
0
200
400
600
800
raw
roasted
Apulia
International
Sicily
78 – Pizzuta d'Avola
33 – Di Giorgio
73 – Perciavisazza
7 – Bari Sabittisa
9 – Belvedere
40 – Fastuchina
63 – Miuzza
59 – Mezza lira
42 – Favaro' 2
81 – Rapparina
43 – Fellamasa Casteltermini
11 – Bianculidda di Pezzino
2 – Amara di Martorana
32 – Cuti
91 – Selvatica Favata
25 – Cesaro 1
19 – Caluriedda
27 – Chiricupara
1 – Acquaviva
26 – Chiarchiara
34 – Don Filippo
13 – Bronte 1
95 – Texas
15 – Cacciatura
21 – Catrubba
28 – Comunista
41 – Favaro
87 – Sarbaggia di Sciascia
29 – Cumma
4 – Baggiana
64 – Montagna
89 – Sarbaggia di Patito
100 – Uova di Cucco
50 – Giardinella
72 – Palma
5 – Bargellera
54 – Lumia
104 – Zammuto 2
58 – Mennula du vattiu
99 – Universo
77 – Pizzuta Contino
37 – Enna 2
8 – Barunissa
92 – Staccia
93 – Supernova
65 – Mullisa Tonda
48 – Gaglio
55 – Marra Mennuladi
67 – Mullisa Piccola
83 – Reginella
84 – Romana Ispica
16 – Cacinova
71 – Nuciddara
17 – Calamonaci
62 – Miricanedda
102 – Zaccaneddara
3 – Angelica
88 – Sarbaggia di Vitello
20 – Carrubina
38 – Falsa pizzuta
30 – Cuore
12 – Bottara
44 – Ferraduel
68 – Nambaredda
24 – Cavaliere
49 – Genco
90 – Scummissa
76 – Pilusedda
98 – Tuono
51 – Griddetta
70 – Niveramanza
10 – Bennici
74 – Persichina
96 – Tricula
56 – Mastraciccia
52 – Laurenne
46 – Filippoceo
97 – Tunnulidda
60 – Milocca
35 – Don Peppino
85 – Romana Licata
45 – Ferragnes
53 – Lisciannarisa
105 – Zarbara
94 – Tabacchina
57 – Mennula du nigliu
106 – Zottafunnuta
86 – Sancisuca
79 – Pizzutella
23 – Cavalera Naro
47 – Filippazzo
75 – Piattamollisa
22 – Cavalera Aragona
14 – Buscemi
66 – Mullisa Grande
101 – Vaiana
6 – Bari Flores
18 – Callara
103 – Zagarri'
80 – Pizzuta Grande
69 – Naro 1
61 – Mirabile
82 – Regina
39 – Fascionello
31 – Cupani piccola
36 – Don Pitrino
B
Concentration, ppbV
0
200
400
600
800
raw
roasted
C
cor = 0.612 p.value = 0
roasted
0
250
500
750
1000
raw
0
500
1000

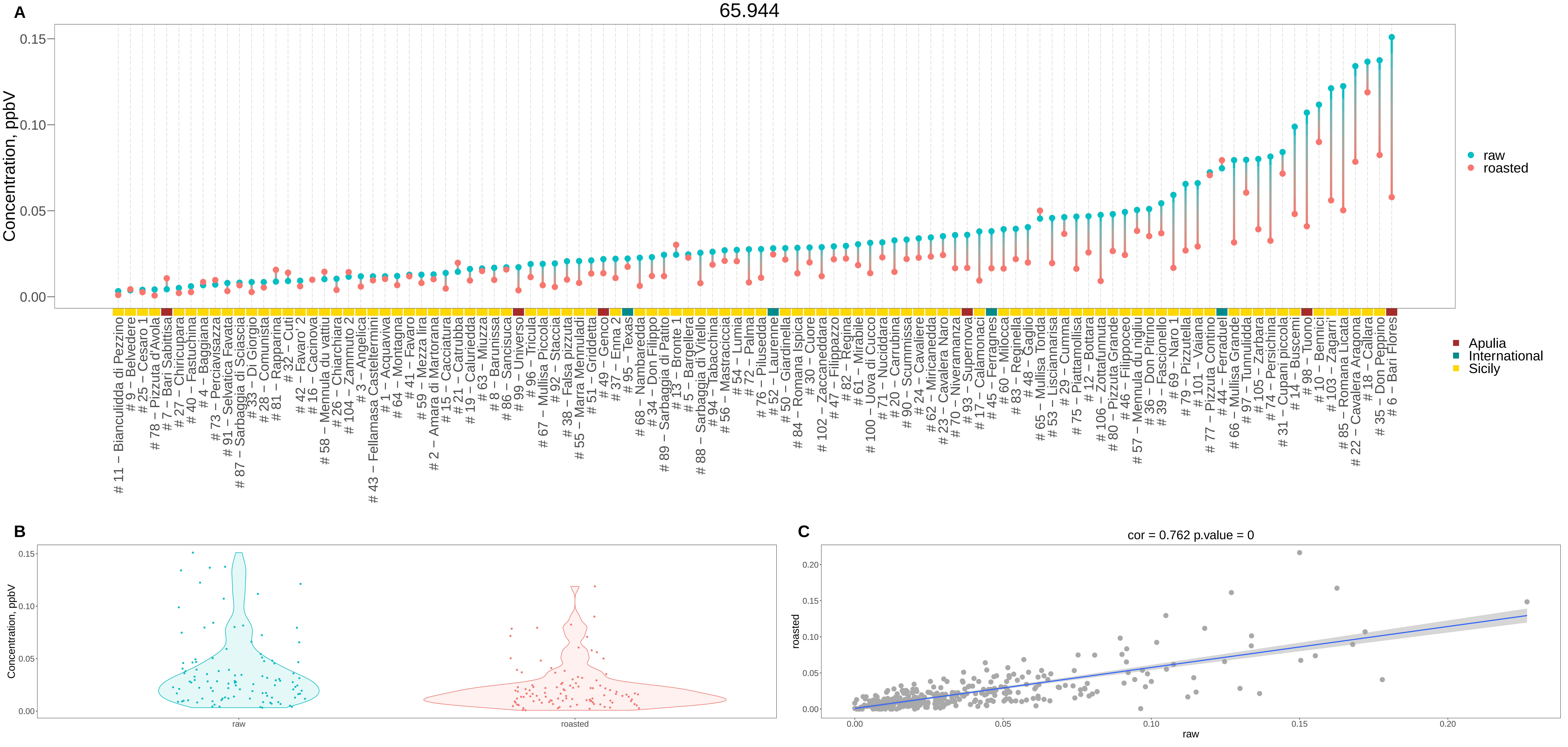

A
65.944
Concentration, ppbV
raw
roasted
Apulia
International
Sicily
B
Concentration, ppbV
raw
roasted
C
cor = 0.762 p.value = 0
roasted
raw

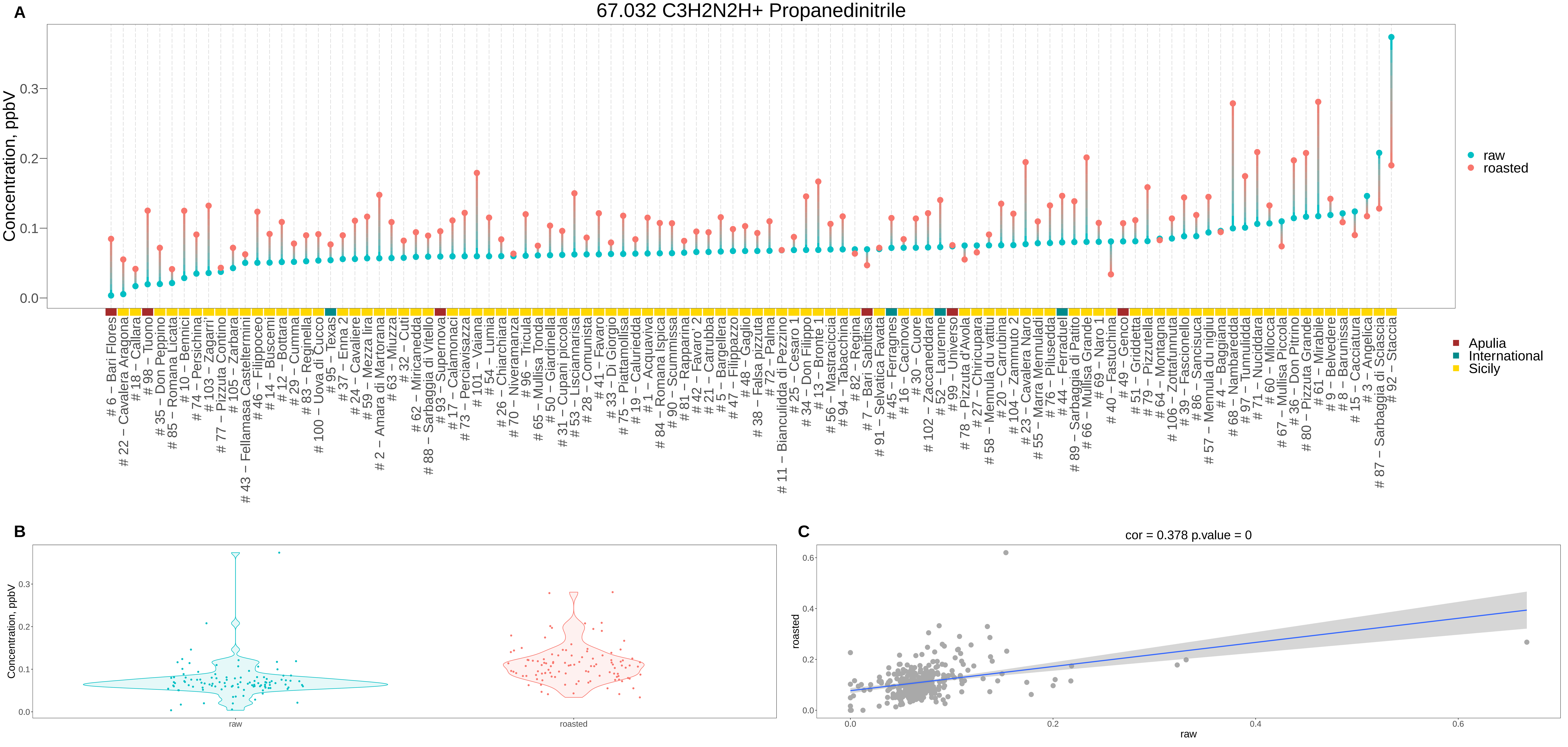
A
67.032 C3H2N2H+ Propanedinitrile
Concentration, ppbV
0.0
0.1
0.2
0.3
raw
roasted
Apulia
International
Sicily
6 – Bari Flores
22 – Cavalera Aragona
18 – Callara
98 – Tuono
35 – Don Peppino
85 – Romana Licata
10 – Bennici
74 – Persichina
103 – Zagarri'
77 – Pizzuta Contino
105 – Zarbara
43 – Fellamasa Casteltermini
46 – Filippoceo
14 – Buscemi
12 – Bottara
29 – Cumma
83 – Reginella
100 – Uova di Cucco
95 – Texas
37 – Enna 2
24 – Cavaliere
59 – Mezza lira
2 – Amara di Martorana
63 – Miuzza
32 – Cuti
62 – Miricanedda
88 – Sarbaggia di Vitello
93 – Supernova
17 – Calamonaci
73 – Perciavisazza
101 – Vaiana
54 – Lumia
26 – Chiarchiara
70 – Niveramanza
96 – Tricula
65 – Mullisa Tonda
50 – Giardinella
31 – Cupani piccola
53 – Lisciannarisa
28 – Comunista
41 – Favaro
33 – Di Giorgio
75 – Piattamollisa
19 – Caluriedda
1 – Acquaviva
84 – Romana Ispica
90 – Scummissa
81 – Rapparina
42 – Favaro' 2
21 – Catrubba
5 – Bargellera
47 – Filippazzo
48 – Gaglio
38 – Falsa pizzuta
72 – Palma
11 – Bianculidda di Pezzino
25 – Cesaro 1
34 – Don Filippo
13 – Bronte 1
56 – Mastraciccia
94 – Tabacchina
82 – Regina
7 – Bari Sabittisa
91 – Selvatica Favata
45 – Ferragnes
16 – Cacinova
30 – Cuore
102 – Zaccaneddara
52 – Laurenne
99 – Universo
78 – Pizzuta d'Avola
27 – Chiricupara
58 – Mennula du vattiu
20 – Carrubina
104 – Zammuto 2
23 – Cavalera Naro
55 – Marra Mennuladi
76 – Pilusedda
44 – Ferraduel
89 – Sarbaggia di Patito
66 – Mullisa Grande
69 – Naro 1
40 – Fastuchina
49 – Genco
51 – Griddetta
79 – Pizzutella
64 – Montagna
106 – Zottafunnuta
39 – Fascionello
86 – Sancisuca
57 – Mennula du nigliu
4 – Baggiana
68 – Nambaredda
97 – Tunnulidda
71 – Nuciddara
60 – Milocca
67 – Mullisa Piccola
36 – Don Pitrino
80 – Pizzuta Grande
61 – Mirabile
9 – Belvedere
8 – Barunissa
15 – Cacciatura
3 – Angelica
87 – Sarbaggia di Sciascia
92 – Staccia
B
Concentration, ppbV
0.0
0.1
0.2
0.3
raw
roasted
C
cor = 0.378 p.value = 0
roasted
raw
0.0
0.2
0.4
0.6

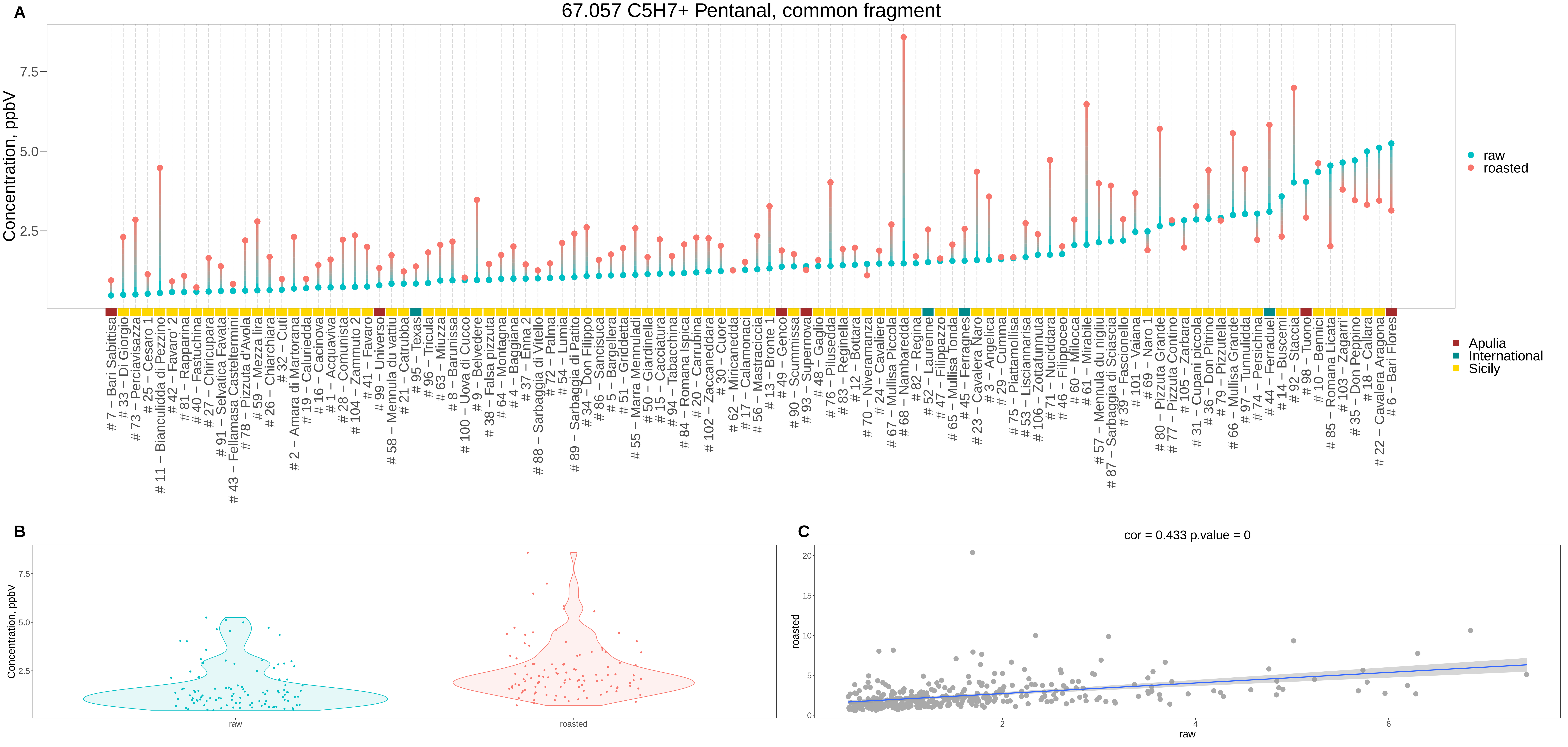

A
67.057 C5H7+ Pentanal, common fragment
Concentration, ppbV
raw
roasted
Apulia
International
Sicily
B
Concentration, ppbV
raw
roasted
C
cor = 0.433 p.value = 0
roasted
raw

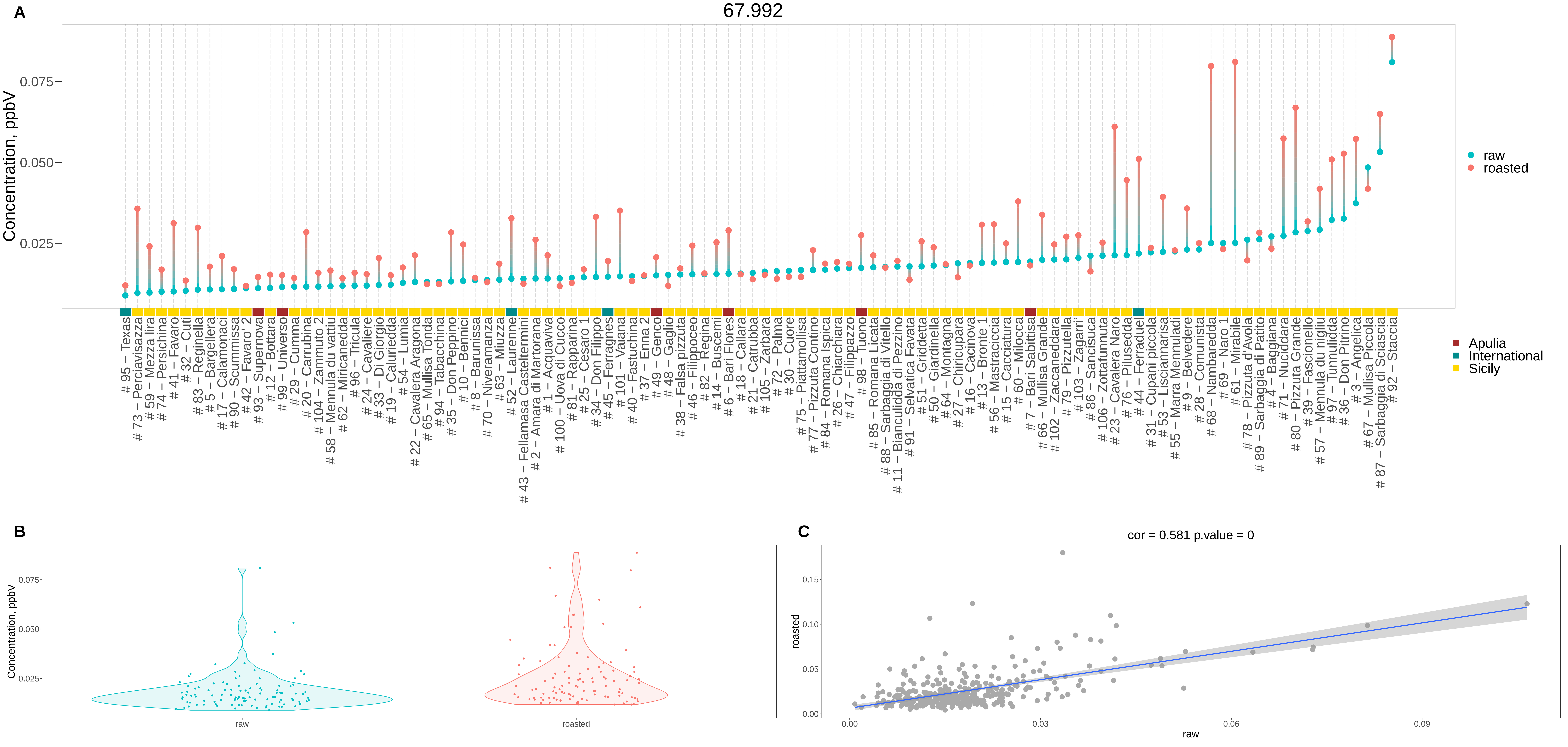
A
67.992
Concentration, ppbV
0.025
0.050
0.075
raw
roasted
Apulia
International
Sicily
95 – Texas
73 – Perciavisazza
59 – Mezza lira
74 – Persichina
41 – Favaro
32 – Cuti
83 – Reginella
5 – Bargellera
17 – Calamonaci
90 – Scummissa
42 – Favaro' 2
93 – Supernova
12 – Bottara
99 – Universo
29 – Cumma
20 – Carrubina
104 – Zammuto 2
58 – Mennula du vattiu
62 – Miricanedda
96 – Tricula
24 – Cavaliere
33 – Di Giorgio
19 – Caluriedda
54 – Lumia
22 – Cavalera Aragona
65 – Mullisa Tonda
94 – Tabacchina
35 – Don Peppino
10 – Bennici
8 – Barunissa
70 – Niveramanza
63 – Miuzza
52 – Laurenne
43 – Fellamasa Casteltermini
2 – Amara di Martorana
1 – Acquaviva
100 – Uova di Cucco
81 – Rapparina
25 – Cesaro 1
34 – Don Filippo
45 – Ferragnes
101 – Valana
40 – Fastuchina
37 – Enna 2
49 – Genco
48 – Gaglio
38 – Falsa pizzuta
46 – Filippoceo
82 – Regina
14 – Buscemi
6 – Bari Flores
18 – Callara
21 – Catrubba
105 – Zarbara
72 – Palma
30 – Cuore
75 – Piattamollisa
77 – Pizzuta Contino
84 – Romana Ispica
26 – Chiarchiara
47 – Filippazzo
98 – Tuono
85 – Romana Licata
88 – Sarbaggia di Vitello
11 – Bianculidda di Pezzino
91 – Selvatica Favata
51 – Griddetta
50 – Giardinella
64 – Montagna
27 – Chiricupara
16 – Cacinova
13 – Bronte 1
56 – Mastraciccia
15 – Cacciatura
60 – Milocca
7 – Bari Sabitisa
66 – Mullisa Grande
102 – Zaccaneddara
79 – Pizzutella
103 – Zagarri'
86 – Sancisuca
106 – Zottafunnuta
23 – Cavalera Naro
76 – Pilusedda
44 – Ferraduel
31 – Cupani piccola
53 – Liscianarisa
55 – Marra Mennuladi
9 – Belvedere
28 – Comunista
68 – Nambaredda
69 – Naro 1
61 – Mirabile
78 – Pizzuta d'Avola
89 – Sarbaggia di Patito
4 – Baggiana
71 – Nuciddara
80 – Pizzuta Grande
39 – Fascionello
57 – Mennula du niglìu
97 – Tunnulidda
36 – Don Pitrino
3 – Angelica
67 – Mullisa Piccola
87 – Sarbaggia di Sciascia
92 – Staccia
B
Concentration, ppbV
0.025
0.050
0.075
raw
roasted
C
cor = 0.581 p.value = 0
roasted
0.00
0.05
0.10
0.15
0.00
0.03
0.06
0.09
raw

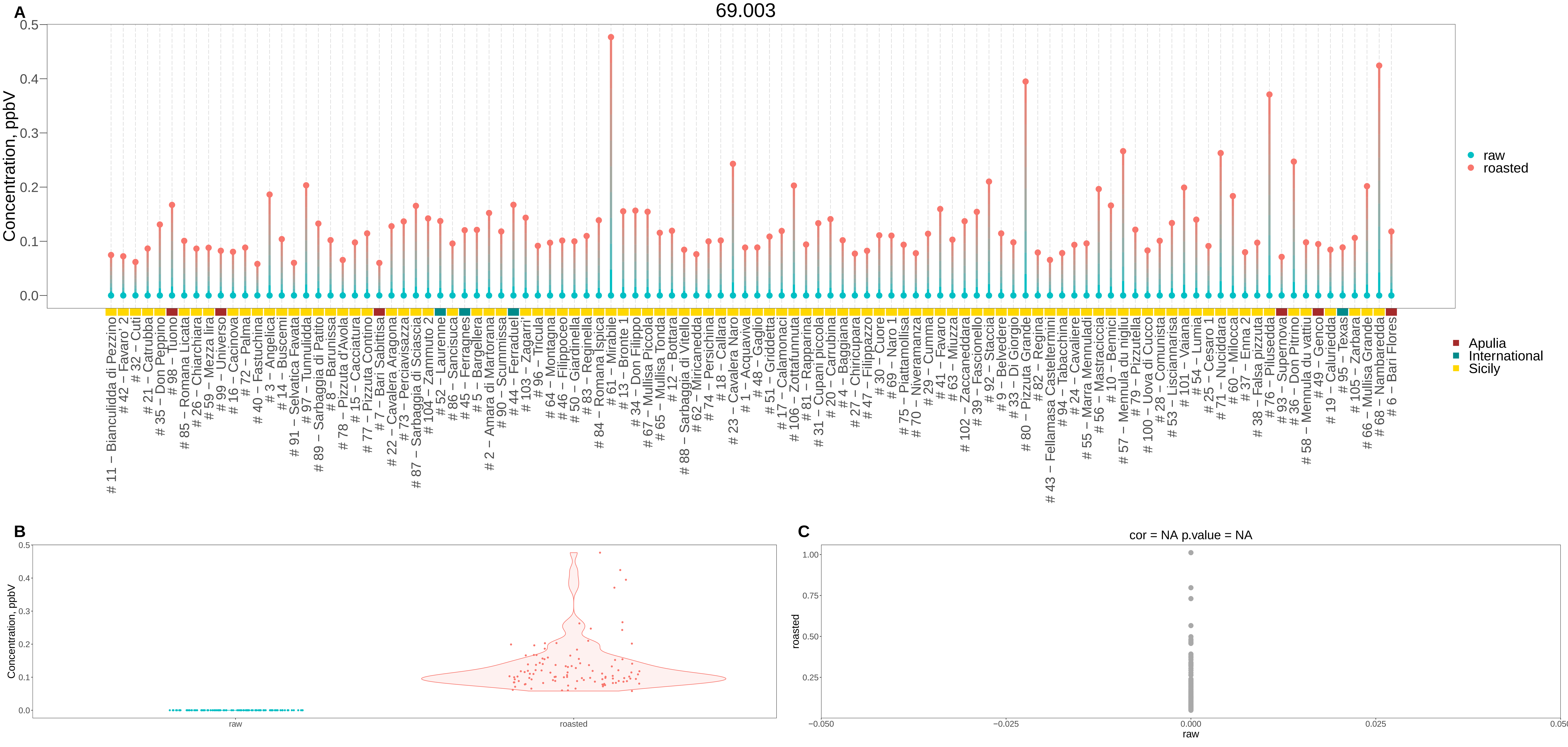

A
69.003
Concentration, ppbV
0.0
0.1
0.2
0.3
0.4
0.5
raw
roasted
Apulia
International
Sicily
11 – Bianculidda di Pezzino
42 – Favaro' 2
32 – Cuti
21 – Catrubba
35 – Don Peppino
98 – Tuono
85 – Romana Licata
26 – Chiarchiara
59 – Mezza lira
99 – Universo
16 – Cacinova
72 – Palma
40 – Fastuchina
3 – Angelica
14 – Buscemi
91 – Selvatica Favata
97 – Tunnulidda
89 – Sarbaggia di Patito
8 – Barunissa
78 – Pizzuta d'Avola
15 – Cacciatura
77 – Pizzuta Contino
7 – Bari Sabittisa
22 – Cavalera Aragona
73 – Perciavisazza
87 – Sarbaggia di Sciascia
104 – Zammuto 2
52 – Laurenne
86 – Sancisuca
45 – Ferragnes
5 – Bargellera
2 – Amara di Martorana
90 – Scummissa
44 – Ferraduel
103 – Zagarri
96 – Tricula
64 – Montagna
46 – Filippoceo
50 – Giardinella
83 – Reginella
84 – Romana Ispica
61 – Mirabile
13 – Bronte 1
34 – Don Filippo
67 – Mullisa Piccola
65 – Mullisa Tonda
12 – Bottara
88 – Sarbaggia di Vitello
62 – Miricanedda
74 – Persichina
18 – Callara
23 – Cavalera Naro
1 – Acquaviva
48 – Gaglio
51 – Griddetta
17 – Calamonaci
106 – Zottafunnuta
81 – Rapparina
31 – Cupani piccola
20 – Carrubina
4 – Baggiana
27 – Chiricupara
47 – Filipazzo
30 – Cuore
69 – Naro 1
75 – Piattamollisa
70 – Niveramanza
29 – Cumma
41 – Favaro
63 – Miuzza
102 – Zaccaneddara
39 – Fascionello
92 – Staccia
9 – Belvedere
33 – Di Giorgio
80 – Pizzuta Grande
82 – Regina
43 – Fellamasa Castelterminі
94 – Tabacchina
24 – Cavaliere
55 – Marra Mennuladi
56 – Mastracicia
10 – Bennici
57 – Mennula du nigliu
79 – Pizzutella
100 – Uova di Cucco
28 – Comunnisa
53 – Liscianarisa
101 – Vaiana
54 – Lumia
25 – Cesaro 1
71 – Nuciddara
60 – Milocca
37 – Enna 2
38 – Falsa pizzuta
76 – Pilusedda
93 – Supernova
36 – Don Pitrino
58 – Mennula du vattiu
49 – Genco
19 – Caluriedda
95 – Texas
105 – Zarbara
66 – Mullisa Grande
68 – Nambaredda
6 – Bari Flores
B
Concentration, ppbV
0.0
0.1
0.2
0.3
0.4
0.5
raw
roasted
C
cor = NA p.value = NA
roasted
0.25
0.50
0.75
1.00
−0.050
−0.025
0.000
0.025
0.050
raw

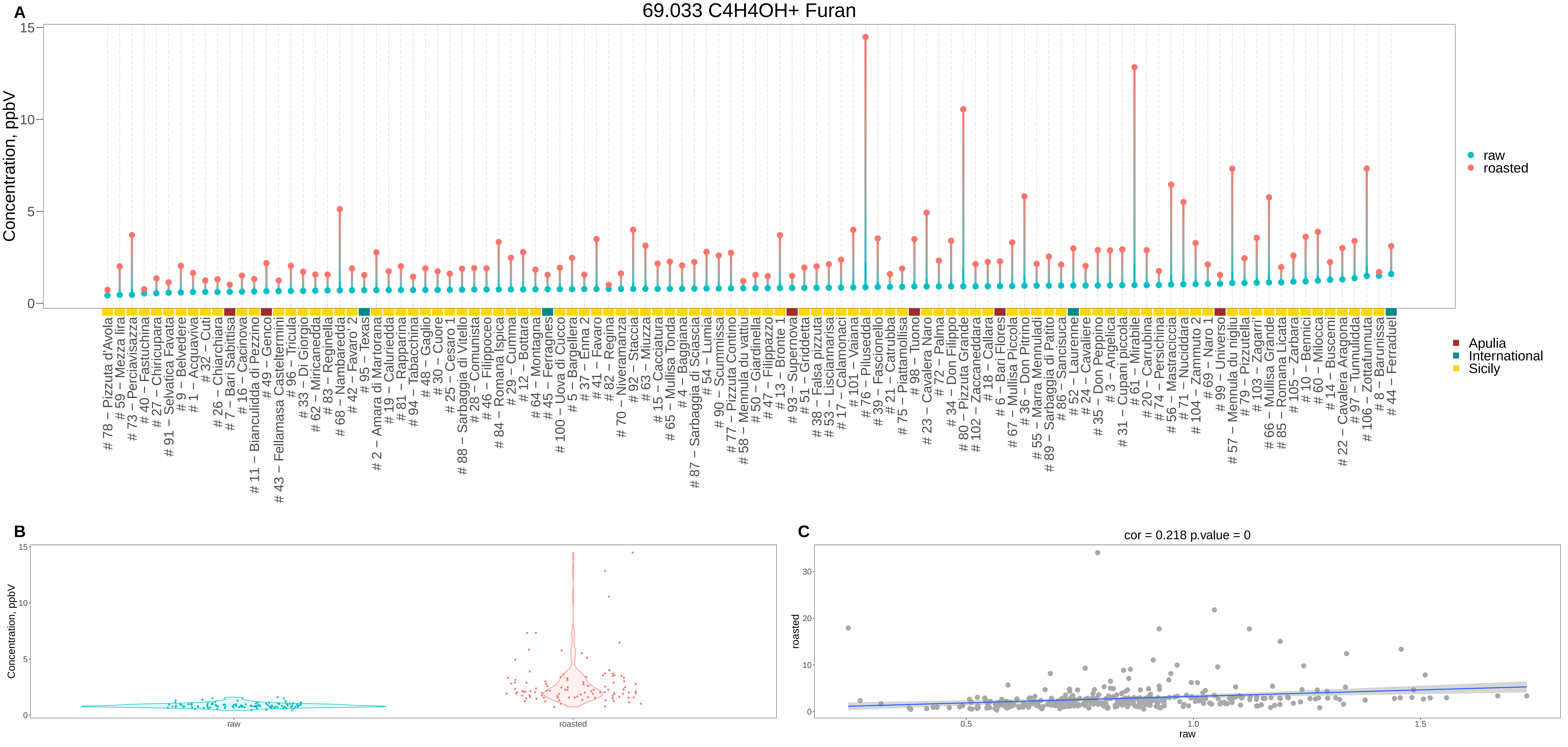

A
69.033 C4H4OH+ Furan
Concentration, ppbV
0
5
10
15
raw
roasted
Apulia
International
Sicily
78 – Pizzuta d'Avola
59 – Mezza lira
73 – Perciavisazza
40 – Fastuchina
27 – Chiricupara
91 – Selvatica Favata
9 – Belvedere
1 – Acquaviva
32 – Cuti
26 – Chiarchiara
7 – Bari Sabittisa
16 – Cacinova
11 – Bianculidda di Pezzino
49 – Genco
43 – Fellamasa Casteltermini
96 – Tricula
33 – Di Giorgio
62 – Miricanedda
83 – Reginella
68 – Nambaredda
42 – Favaro' 2
95 – Texas
2 – Amara di Martorana
19 – Caluriedda
81 – Rapparina
94 – Tabacchina
48 – Gaglio
30 – Cuore
25 – Cesaro 1
88 – Sarbaggia di Vitello
28 – Comunista
46 – Filippoceo
84 – Romana Ispica
29 – Cumma
12 – Bottara
64 – Montagna
45 – Ferragnes
100 – Uova di Cucco
5 – Bargellera
37 – Enna 2
41 – Favaro
82 – Regina
70 – Niveramanza
92 – Staccia
63 – Miuzza
15 – Cacciatura
65 – Mullisa Tonda
4 – Baggiana
87 – Sarbaggia di Sciascia
54 – Lumia
90 – Scummissa
77 – Pizzuta Contino
58 – Mennula du vattiu
50 – Giardinella
47 – Filippazzo
13 – Bronte 1
93 – Supernova
51 – Griddetta
38 – Falsa pizzuta
53 – Liscianarisa
17 – Calamonaci
101 – Vaiana
76 – Pilusedda
39 – Fascionello
21 – Catrubba
75 – Piattamollisa
98 – Tuono
23 – Cavalera Naro
72 – Palma
34 – Don Filippo
80 – Pizzuta Grande
102 – Zaccaneddara
18 – Callara
6 – Bari Flores
67 – Mullisa Piccola
36 – Don Pitrino
55 – Marra Mennuladi
89 – Sarbaggia di Patito
86 – Sancisuca
52 – Laurenne
24 – Cavaliere
35 – Don Peppino
3 – Angelica
31 – Cupani piccola
61 – Mirabile
20 – Carrubina
74 – Persichina
56 – Mastracicca
71 – Nuciddara
104 – Zammuto 2
69 – Naro 1
99 – Universo
57 – Mennula du nigliu
79 – Pizzutella
103 – Zagarri'
66 – Mullisa Grande
85 – Romana Licata
105 – Zarbara
10 – Bennici
60 – Milocca
14 – Buscemi
22 – Cavalera Aragona
97 – Tunnulidda
106 – Zottafunnuta
8 – Barunissa
44 – Ferraduel
B
Concentration, ppbV
0
5
10
15
raw
roasted
C
cor = 0.218 p.value = 0
roasted
0
10
20
30
0.5
1.0
1.5
raw

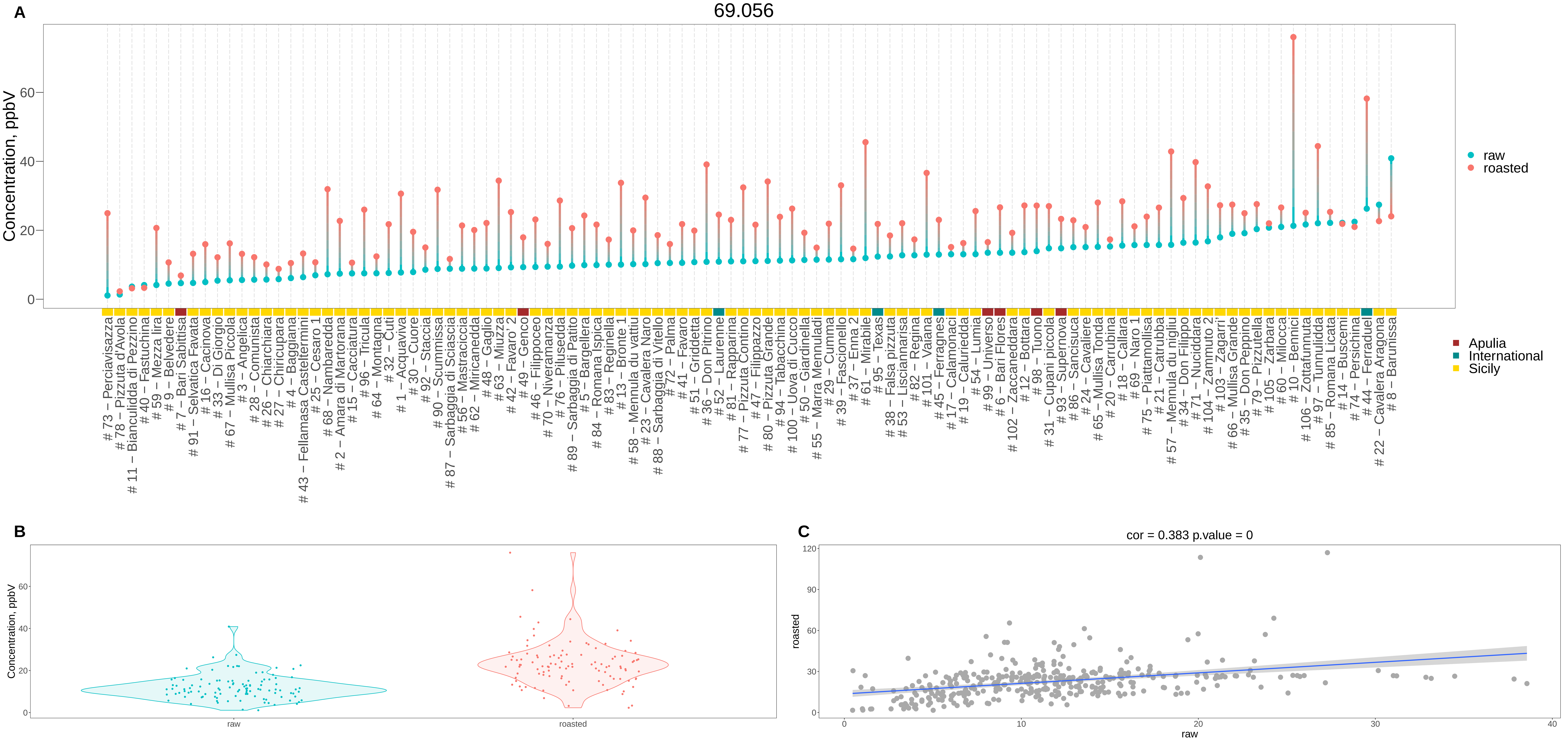
A
69.056
Concentration, ppbV
0
20
40
60
raw
roasted
Apulia
International
Sicily
73 – Perciavisazza
78 – Pizzuta d'Avola
11 – Bianculidda di Pezzino
40 – Fastuchina
59 – Mezza lira
9 – Belvedere
7 – Bari Sabittisa
91 – Selvatica Favata
16 – Cacinova
33 – Di Giorgio
67 – Mullisa Piccola
3 – Angelica
28 – Comunista
26 – Chiarchiara
27 – Chiricupara
4 – Baggiana
43 – Fellamasa Casteltermini
25 – Cesaro 1
68 – Nambaredda
2 – Amara di Martorana
15 – Cacciatura
96 – Tricula
64 – Montagna
32 – Cuti
1 – Acquaviva
30 – Cuore
92 – Staccia
90 – Scummissa
87 – Sarbaggia di Sciascia
56 – Mastraciccia
62 – Miricanedda
48 – Gaglio
63 – Miuzza
42 – Favaro' 2
49 – Genco
46 – Filippoceo
70 – Niveramanza
76 – Pilusedda
89 – Sarbaggia di Patito
5 – Bargellera
84 – Romana Ispica
83 – Reginella
13 – Bronte 1
58 – Mennula du vattiu
23 – Cavalera Naro
88 – Sarbaggia di Vitello
72 – Palma
41 – Favaro
51 – Griddetta
36 – Don Pitrino
52 – Laurenne
81 – Rapparina
77 – Pizzuta Contino
47 – Filippazzo
80 – Pizzuta Grande
94 – Tabacchina
100 – Uova di Cucco
50 – Giardinella
55 – Marra Mennuladi
29 – Cumma
39 – Fascionello
37 – Enna 2
61 – Mirabile
95 – Texas
38 – Falsa pizzuta
53 – Lisciannarisa
82 – Regina
101 – Vaiana
45 – Ferragnes
17 – Calamonaci
19 – Caluriedda
54 – Lumia
99 – Universo
6 – Bari Flores
102 – Zaccaneddara
12 – Bottara
98 – Tuono
31 – Cupani piccola
93 – Supernova
86 – Sancisuca
24 – Cavaliere
65 – Mullisa Tonda
20 – Carrubina
18 – Callara
69 – Naro 1
75 – Piattamollisa
21 – Catrubba
57 – Mennula du nigliu
34 – Don Filippo
71 – Nuciddara
104 – Zammuto 2
103 – Zagarri'
66 – Mullisa Grande
35 – Don Peppino
79 – Pizzutella
105 – Zarbara
60 – Milocca
10 – Bennici
106 – Zottafunnuta
97 – Tunnulidda
85 – Romana Licata
14 – Buscemi
74 – Persichina
44 – Ferraduel
22 – Cavalera Aragona
8 – Barunissa
B
Concentration, ppbV
0
20
40
60
raw
roasted
C
cor = 0.383 p.value = 0
roasted
0
30
60
90
120
raw
0
10
20
30
40

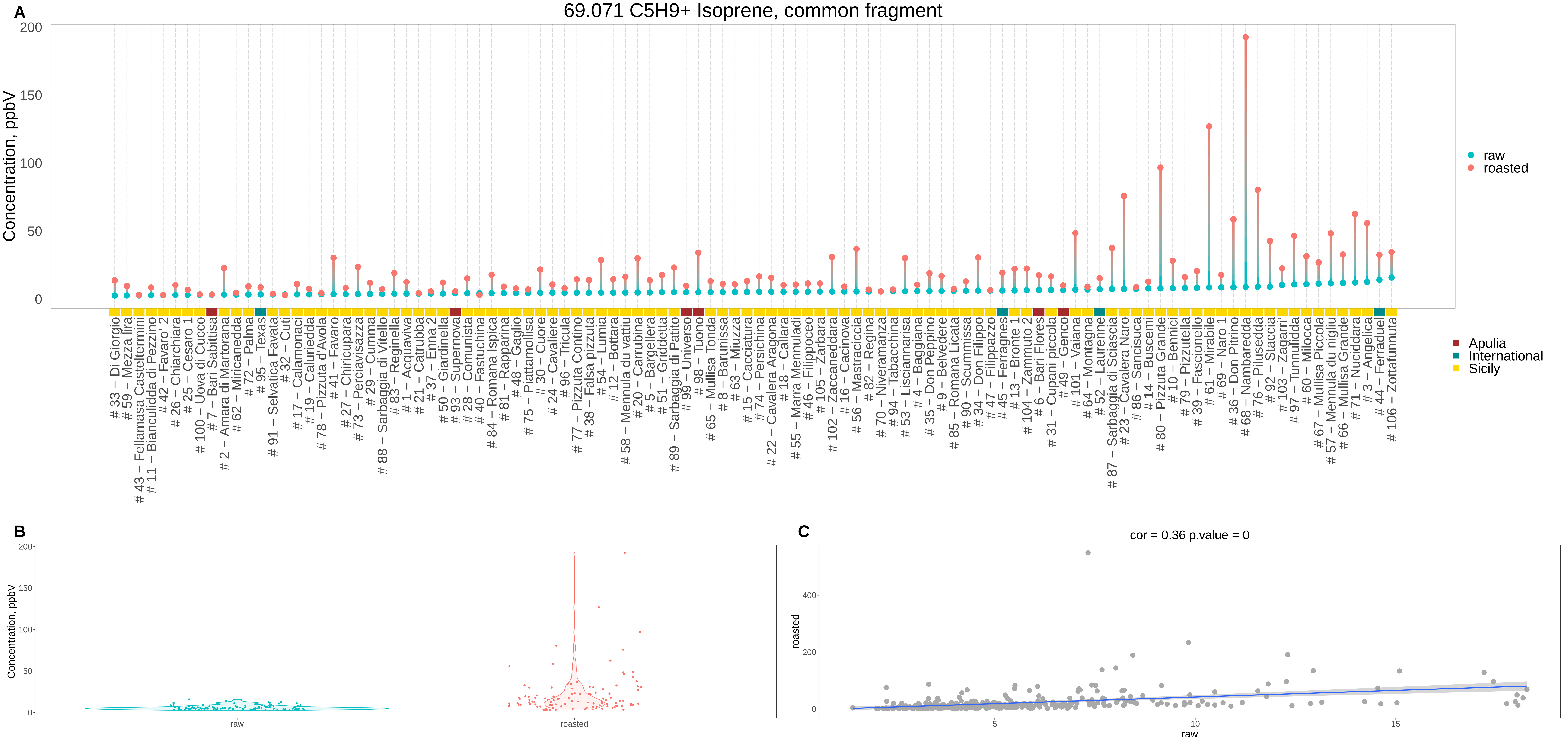

A
69.071 C5H9+ Isoprene, common fragment
Concentration, ppbV
200
150
100
50
0
raw
roasted
Apulia
International
Sicily
33 – Di Giorgio
59 – Mezza lira
43 – Fellamasa Casteltermini
11 – Bianculidda di Pezzino
42 – Favaro' 2
26 – Chiarchiara
25 – Cesaro 1
100 – Uova di Cucco
7 – Bari Sabitisa
2 – Amara di Martorana
62 – Miricanedda
72 – Palma
95 – Texas
91 – Selvatica Favata
32 – Cuti
17 – Calamonaci
19 – Caluriedda
78 – Pizzuta d'Avola
41 – Favaro
27 – Chiricupara
73 – Perciavisazza
29 – Cumma
88 – Sarbaggia di Vitello
83 – Reginella
1 – Acquaviva
21 – Catrubba
37 – Enna 2
50 – Giardinella
93 – Supernova
28 – Comunista
40 – Fastuchina
84 – Romana Ispica
81 – Rapparina
48 – Gaglio
75 – Piattamollisa
30 – Cuore
24 – Cavaliere
96 – Tricula
77 – Pizzuta Contino
38 – Falsa pizzuta
54 – Lumia
12 – Bottara
58 – Mennula du vattiu
20 – Carrubina
5 – Bargellera
51 – Griddetta
89 – Sarbaggia di Patito
99 – Universo
98 – Tuono
65 – Mullisa Tonda
8 – Barunissa
63 – Miuzza
15 – Cacciatura
74 – Persichina
22 – Cavalera Aragona
18 – Callara
55 – Marra Mennuladi
46 – Filippoceo
105 – Zarbara
102 – Zaccaneddara
16 – Cacinova
56 – Mastraciccia
82 – Regina
70 – Niveramanza
94 – Tabacchina
53 – Liscianarisa
4 – Baggiana
35 – Don Peppino
9 – Belvedere
85 – Romana Licata
90 – Scummissa
34 – Don Filippo
47 – Filippazzo
45 – Ferragnes
13 – Bronte 1
104 – Zammuto 2
6 – Bari Flores
31 – Cupani piccola
49 – Genco
101 – Vaiana
64 – Montagna
52 – Laurenne
87 – Sarbaggia di Sciascia
23 – Cavalera Naro
86 – Sancisuca
14 – Buscemi
80 – Pizzuta Grande
10 – Bennici
79 – Pizzutella
39 – Fascionello
61 – Mirabile
69 – Naro 1
36 – Don Pitrino
68 – Nambaredda
76 – Pilusedda
92 – Staccia
103 – Zagarri'
97 – Tunnulidda
60 – Milocca
67 – Mullisa Piccola
57 – Mennula du nigliu
66 – Mullisa Grande
71 – Nuciddara
3 – Angelica
44 – Ferraduel
106 – Zottafunnuta
B
Concentration, ppbV
200
150
100
50
0
raw
roasted
C
cor = 0.36 p.value = 0
roasted
400
200
0
5
10
15
raw

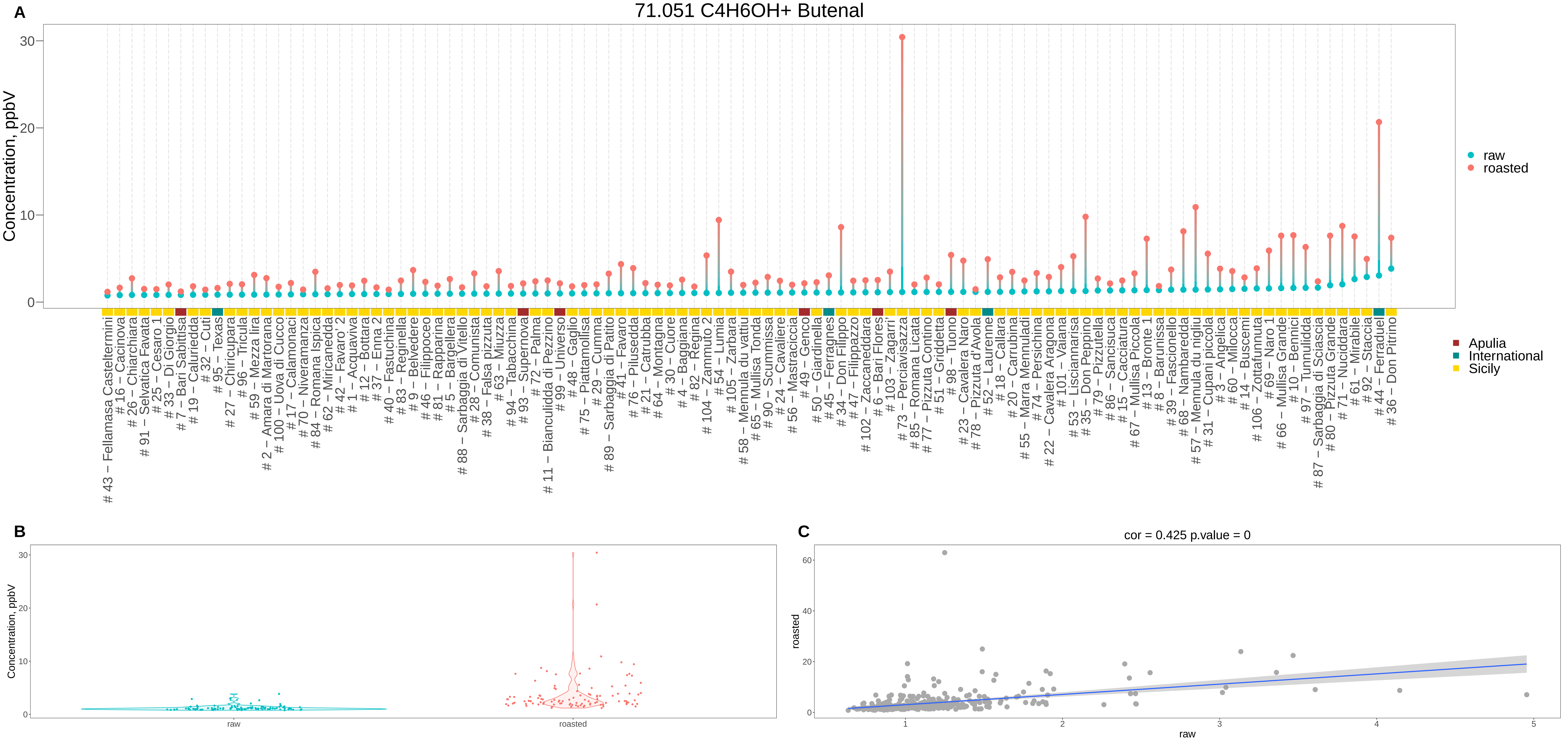
A
71.051 C4H6OH+ Butenal
Concentration, ppbV
0
10
20
30
raw
roasted
Apulia
International
Sicily
43 – Fellamasa Casteltermini
16 – Cacinova
26 – Chiarchiara
91 – Selvatica Favata
25 – Cesaro 1
33 – Di Giorgio
7 – Bari Sabitisa
19 – Caluriedda
32 – Cuti
95 – Texas
27 – Chiricupara
96 – Tricula
59 – Mezza lira
2 – Amara di Martorana
100 – Uova di Cucco
17 – Calamonaci
70 – Niveramanza
84 – Romana Ispica
62 – Miricanedda
42 – Favaro' 2
1 – Acquaviva
12 – Bottara
37 – Enna 2
40 – Fastuchina
83 – Reginella
9 – Belvedere
46 – Filippoceo
81 – Rapparina
5 – Bargellera
88 – Sarbaggia di Vitello
28 – Comunista
38 – Falsa pizzuta
63 – Miuzza
94 – Tabacchina
93 – Supernova
72 – Palma
11 – Bianculidda di Pezzino
99 – Universo
48 – Gaglio
75 – Piattamollisa
29 – Cumma
89 – Sarbaggia di Patito
41 – Favaro
76 – Piluseddа
21 – Catrubba
64 – Montagna
30 – Cuore
4 – Baggiana
82 – Regina
104 – Zammuto 2
54 – Lumia
105 – Zarbara
58 – Mennula du vattiu
65 – Mullisa Tonda
90 – Scummissa
24 – Cavaliere
56 – Mastraciccia
49 – Genco
50 – Giardinella
45 – Ferragnes
34 – Don Filippo
47 – Filippazzo
102 – Zaccaneddara
6 – Bari Flores
103 – Zagarri'
73 – Perciavisazza
85 – Romana Licata
77 – Pizzuta Contino
51 – Griddetta
98 – Tuono
23 – Cavalera Naro
78 – Pizzuta d'Avola
52 – Laurenne
18 – Callara
20 – Carrubina
55 – Marra Mennuladi
74 – Persichina
22 – Cavalera Aragona
101 – Vaiana
53 – Lisciannarisa
35 – Don Peppino
79 – Pizzutella
86 – Sancisuca
15 – Cacciatura
67 – Mullisa Piccola
13 – Bronte 1
8 – Barunissa
39 – Fascionello
68 – Nambaredda
57 – Mennula du nigliu
31 – Cupani piccola
3 – Angelica
60 – Milocca
14 – Buscemi
106 – Zottafunnuta
69 – Naro 1
66 – Mullisa Grande
10 – Bennici
97 – Tunnulidda
87 – Sarbaggia di Sciascia
80 – Pizzuta Grande
71 – Nuciddara
61 – Mirabile
92 – Staccia
44 – Ferraduel
36 – Don Pitrino
B
Concentration, ppbV
0
10
20
30
raw
roasted
C
cor = 0.425 p.value = 0
roasted
raw
0
20
40
60
1
2
3
4
5

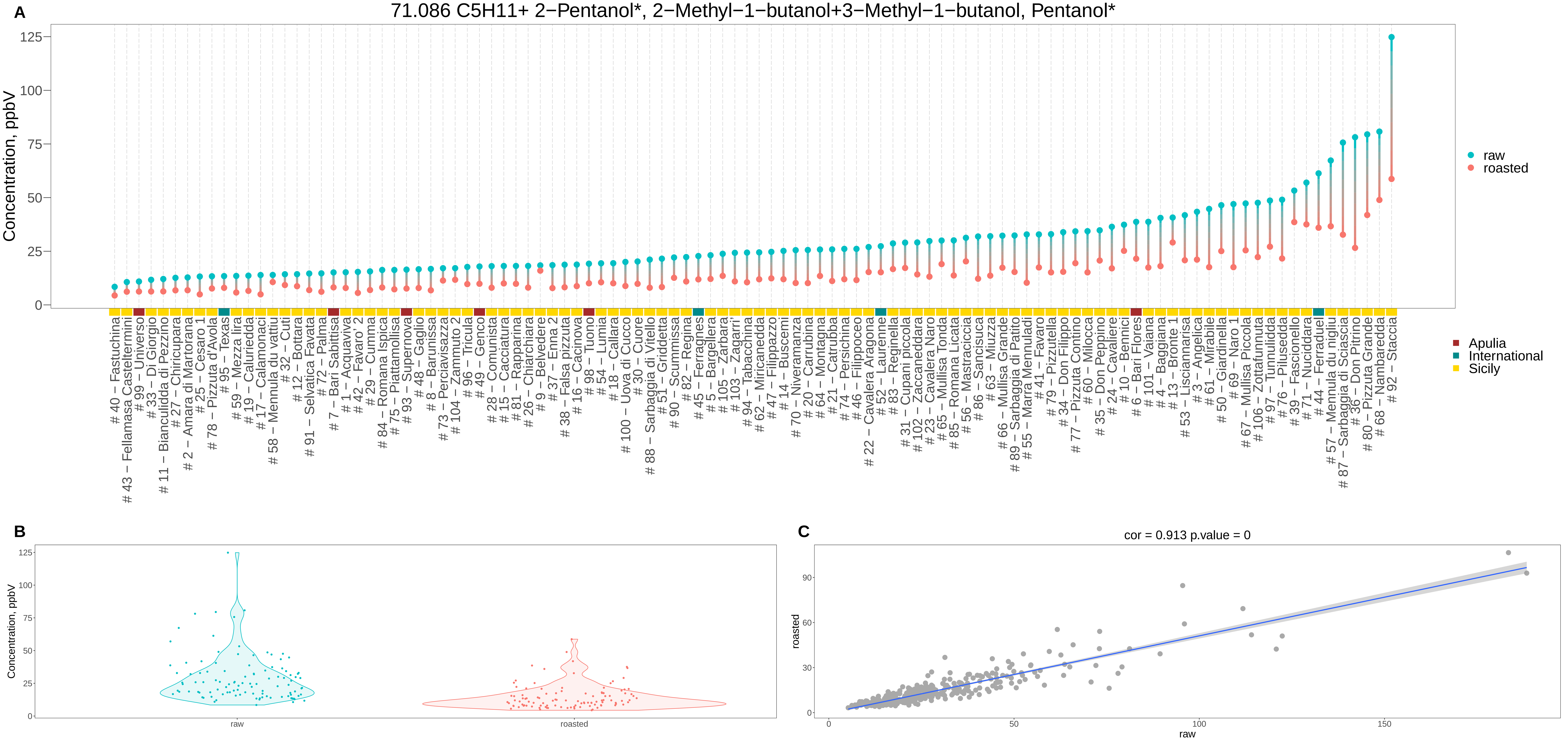
A
71.086 C5H11+ 2−Pentanol*, 2−Methyl−1−butanol+3−Methyl−1−butanol, Pentanol*
Concentration, ppbV
125
100
75
50
25
0
raw
roasted
Apulia
International
Sicily
40 − Fastuchina
43 − Fellamasa Casteltermini
99 − Universo
33 − Di Giorgio
11 − Bianculidda di Pezzino
27 − Chiricupara
2 − Amara di Martorana
25 − Cesaro 1
78 − Pizzuta d'Avola
95 − Texas
59 − Mezza lira
19 − Calurieddа
17 − Calamonaci
58 − Mennula du vattiu
32 − Cuti
12 − Bottara
91 − Selvatica Favata
72 − Palma
7 − Bari Sabitisa
1 − Acquaviva
42 − Favaro' 2
29 − Cumma
84 − Romana Ispica
75 − Piattamollisa
93 − Supernova
48 − Gaglio
8 − Barunissa
73 − Perciavisazza
104 − Zammuto 2
96 − Tricula
49 − Genco
28 − Comunista
15 − Cacciatura
81 − Rapparina
26 − Chiarchiara
9 − Belvedere
37 − Enna 2
38 − Falsa pizzuta
16 − Cacinova
98 − Tuono
54 − Lumia
18 − Callara
100 − Uova di Cucco
30 − Cuore
88 − Sarbaggia di Vitello
51 − Griddetta
90 − Scummissa
82 − Regina
45 − Ferragnes
5 − Bargellera
105 − Zarbara
103 − Zagarri'
94 − Tabacchina
62 − Miricanedda
47 − Filippazzo
14 − Buscemi
70 − Niveramanza
20 − Carrubina
64 − Montagna
21 − Catrubba
74 − Persichina
46 − Filippoceo
22 − Cavalera Aragona
52 − Laurenne
83 − Reginella
31 − Cupani piccola
102 − Zaccaneddara
23 − Cavalera Naro
65 − Mullisa Tonda
85 − Romana Licata
56 − Mastracicia
86 − Sancisuca
63 − Miuzza
66 − Mullisa Grande
89 − Sarbaggia di Patito
55 − Marra Mennuladi
41 − Favaro
79 − Pizzutella
34 − Don Filippo
77 − Pizzuta Contino
60 − Milocca
35 − Don Peppino
24 − Cavaliere
10 − Bennici
6 − Bari Flores
101 − Vaiana
4 − Baggiana
13 − Bronte 1
53 − Lisciannarisa
3 − Angelica
61 − Mirabile
50 − Giardinella
69 − Naro 1
67 − Mullisa Piccola
106 − Zottafunnuta
97 − Tunnulidda
76 − Pilusedda
39 − Fascionello
71 − Nuciddara
44 − Ferraduel
57 − Mennula du nigliu
87 − Sarbaggia di Sciascia
36 − Don Pitrino
80 − Pizzuta Grande
68 − Nambaredda
92 − Staccia
B
Concentration, ppbV
125
100
75
50
25
0
raw
roasted
C
cor = 0.913 p.value = 0
roasted
90
60
30
0
0
50
100
150
raw

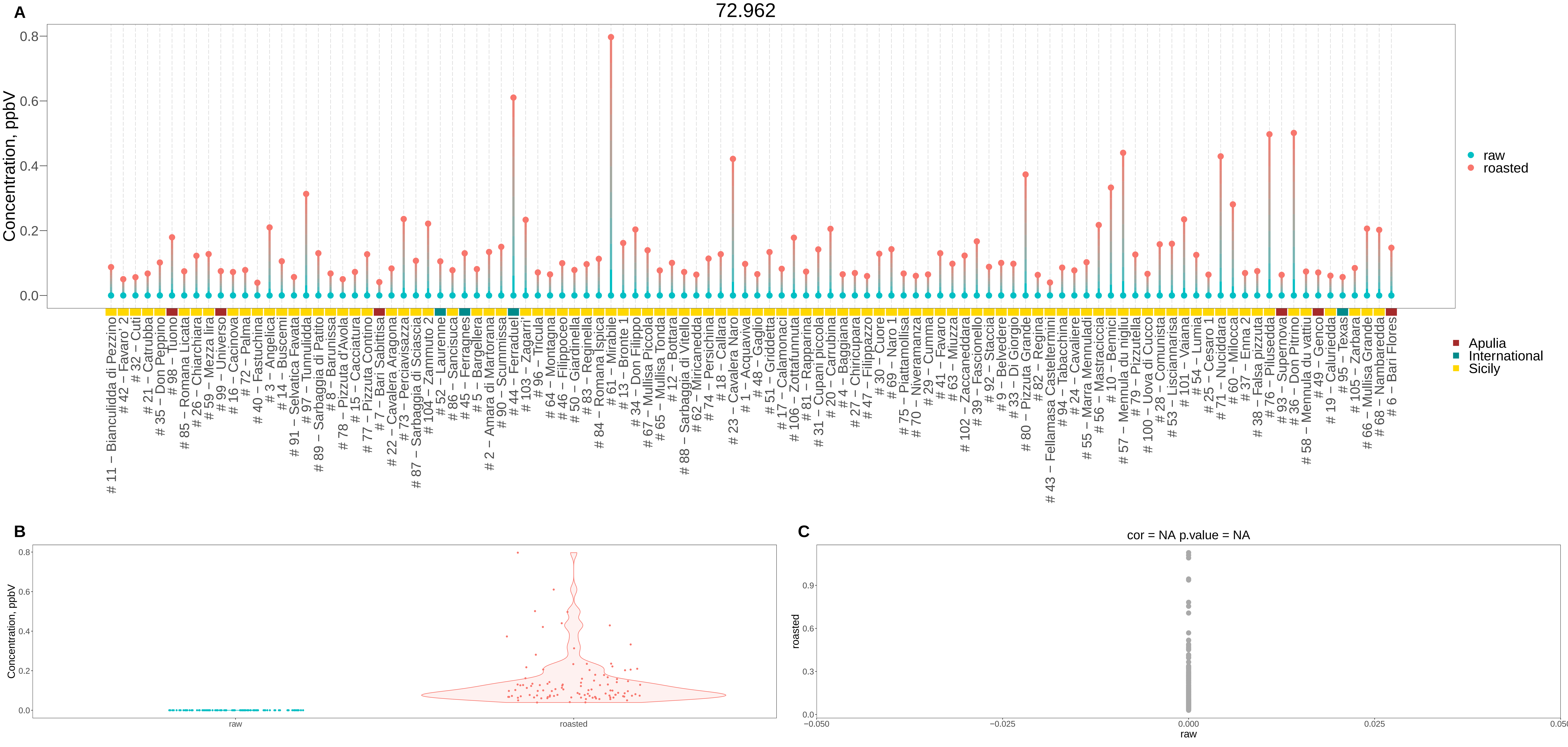
A
72.962
Concentration, ppbV
0.8
0.6
0.4
0.2
0.0
raw
roasted
Apulia
International
Sicily
11 – Bianculidda di Pezzino
42 – Favaro' 2
32 – Cuti
21 – Catrubba
35 – Don Peppino
98 – Tuono
85 – Romana Licata
26 – Chiarchiara
59 – Mezza lira
99 – Universo
16 – Cacinova
72 – Palma
40 – Fastuchina
3 – Angelica
14 – Buscemi
91 – Selvatica Favata
97 – Tunnulidda
89 – Sarbaggia di Patito
8 – Barunissa
78 – Pizzuta d'Avola
15 – Cacciatura
77 – Pizzuta Contino
7 – Bari Sabittisa
22 – Cavalera Aragona
73 – Perciavisazza
87 – Sarbaggia di Sciascia
104 – Zammuto 2
52 – Laurenne
86 – Sancisuca
45 – Ferragnes
5 – Bargellera
2 – Amara di Martorana
90 – Scummissa
44 – Ferraduel
103 – Zagarri
96 – Tricula
64 – Montagna
46 – Filippoceo
50 – Giardinella
83 – Reginella
84 – Romana Ispica
61 – Mirabile
13 – Bronte 1
34 – Don Filippo
67 – Mullisa Piccola
65 – Mullisa Tonda
12 – Bottara
88 – Sarbaggia di Vitello
62 – Miricanedda
74 – Persichina
18 – Callara
23 – Cavalera Naro
1 – Acquaviva
48 – Gaglio
51 – Griddetta
17 – Calamonaci
106 – Zottafunnuta
81 – Rapparina
31 – Cupani piccola
20 – Carrubina
4 – Baggiana
27 – Chiricupara
47 – Filipazzo
30 – Cuore
69 – Naro 1
75 – Piattamollisa
70 – Niveramanza
29 – Cumma
41 – Favaro
63 – Miuzza
102 – Zaccaneddara
39 – Fascionello
92 – Staccia
9 – Belvedere
33 – Di Giorgio
80 – Pizzuta Grande
82 – Regina
43 – Fellamasa Casteltermini
94 – Tabacchina
24 – Cavaliere
55 – Marra Mennuladi
56 – Mastracicia
10 – Bennici
57 – Mennula du nigliu
79 – Pizzutella
100 – Uova di Cucco
28 – Comunista
53 – Lisciannarisa
101 – Vaiana
54 – Lumia
25 – Cesaro 1
71 – Nuciddara
60 – Milocca
37 – Enna 2
38 – Falsa pizzuta
76 – Pilusedda
93 – Supernova
36 – Don Pitrino
58 – Mennula du vattiu
49 – Genco
19 – Caluriedda
95 – Texas
105 – Zarbara
66 – Mullisa Grande
68 – Nambaredda
6 – Bari Flores
B
Concentration, ppbV
0.8
0.6
0.4
0.2
0.0
raw
roasted
C
cor = NA p.value = NA
roasted
0.9
0.6
0.3
0.0
−0.050
−0.025
0.000
0.025
raw

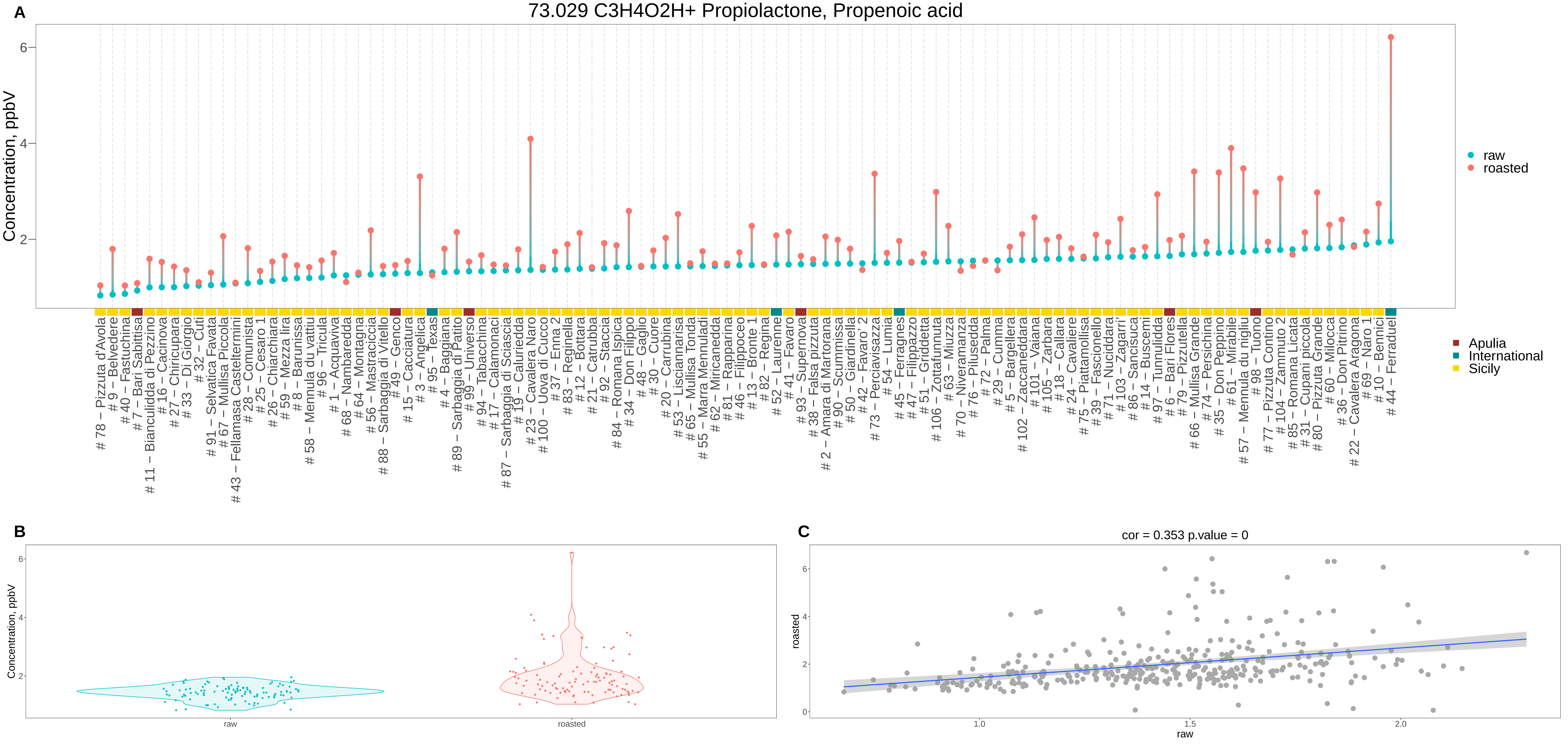

A
73.029 C3H4O2H+ Propiolactone, Propenoic acid
Concentration, ppbV
2
4
6
raw
roasted
Apulia
International
Sicily
78 – Pizzuta d'Avola
9 – Belvedere
40 – Fastuchina
7 – Bari Sabitisa
11 – Bianculidda di Pezzino
16 – Cacinova
27 – Chiricupara
33 – Di Giorgio
32 – Cuti
91 – Selvatica Favata
67 – Mullisa Piccola
43 – Fellamasa Casteltermini
28 – Comunista
25 – Cesaro 1
26 – Chiarchiara
59 – Mezza lira
8 – Barunissa
58 – Mennula du vattiu
96 – Tricula
1 – Acquaviva
68 – Nambaredda
64 – Montagna
56 – Mastracicca
88 – Sarbaggia di Vitello
49 – Genco
15 – Cacciatura
3 – Angelica
95 – Texas
4 – Baggiana
89 – Sarbaggia di Patito
99 – Universo
94 – Tabacchina
17 – Calamonaci
87 – Sarbaggia di Sciascia
19 – Caluriedda
23 – Cavalera Naro
100 – Uova di Cucco
37 – Enna 2
83 – Reginella
12 – Bottara
21 – Catrubba
92 – Staccia
84 – Romana Ispica
34 – Don Filippo
48 – Gaglio
30 – Cuore
20 – Carrubina
53 – Liscianarisa
65 – Mullisa Tonda
55 – Marra Mennuladi
62 – Miricanedda
81 – Rapparina
46 – Filippoceo
13 – Bronte 1
82 – Regina
52 – Laurenne
41 – Favaro
93 – Supernova
38 – Falsa pizzuta
2 – Amara di Martorana
90 – Scummissa
50 – Giardinella
42 – Favaro 2
73 – Perciavisazza
54 – Lumia
45 – Ferragnes
47 – Filippazzo
51 – Griddetta
106 – Zottafunnuta
63 – Miuzza
70 – Niveramanza
76 – Pilusedda
72 – Palma
29 – Cumma
5 – Bargellera
102 – Zaccaneddara
101 – Vaiana
105 – Zarbara
18 – Callara
24 – Cavaliere
75 – Piattamollisa
39 – Fascionello
71 – Nuciddara
103 – Zagarri'
86 – Sancisuca
14 – Buscemi
97 – Tunnulidda
6 – Bari Flores
79 – Pizzutella
66 – Mullisa Grande
74 – Persichina
35 – Don Peppino
61 – Mirabile
57 – Mennula du nigliu
98 – Tuono
77 – Pizzuta Contino
104 – Zammuto 2
85 – Romana Licata
31 – Cupani piccola
80 – Pizzuta Grande
60 – Milocca
36 – Don Pitrino
22 – Cavalera Aragona
69 – Naro 1
10 – Bennici
44 – Ferraduel
B
Concentration, ppbV
2
4
6
raw
roasted
C
cor = 0.353 p.value = 0
roasted
raw
0
2
4
6
1.0
1.5
2.0

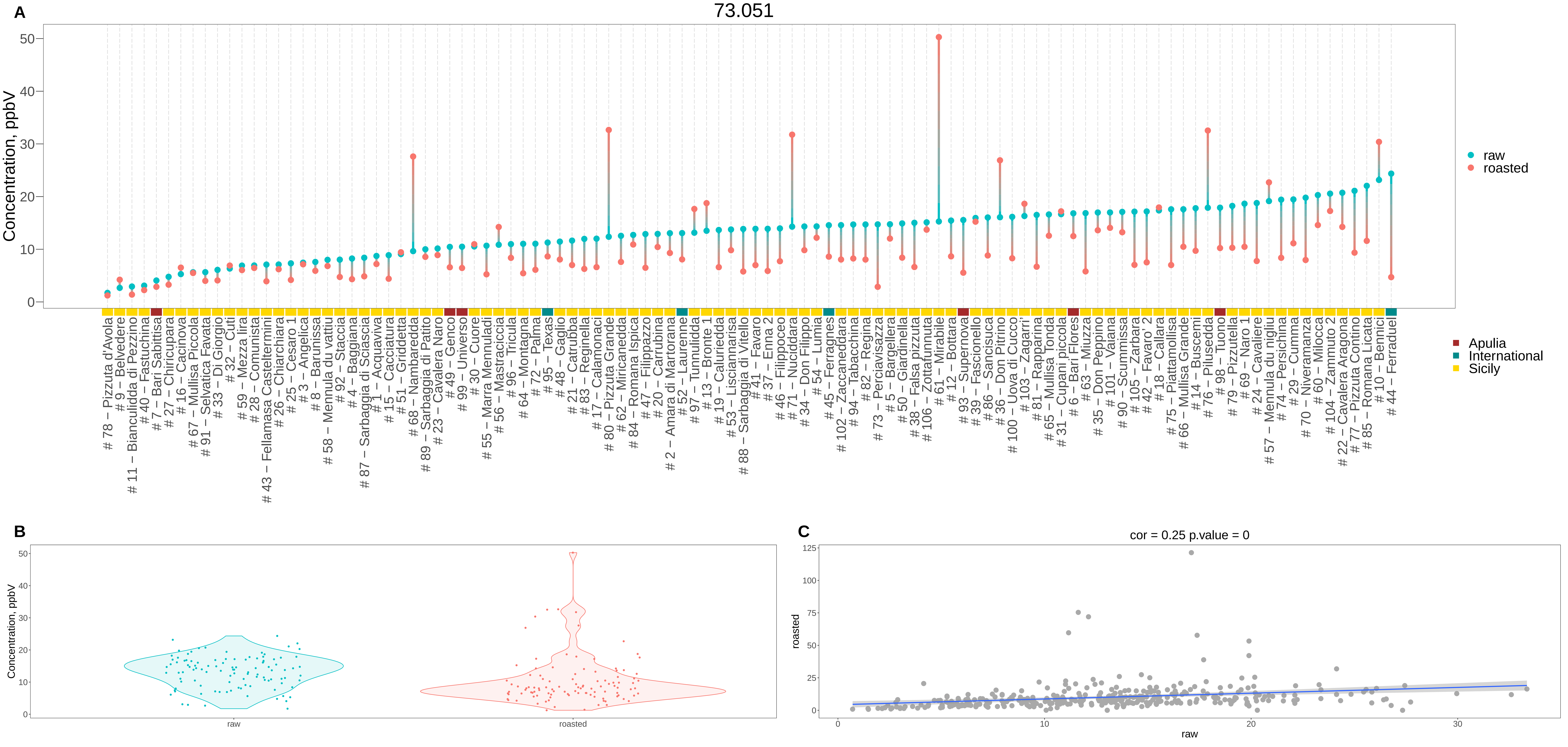
A
73.051
Concentration, ppbV
0
10
20
30
40
50
raw
roasted
78 – Pizzuta d'Avola
9 – Belvedere
11 – Biancuilidda di Pezzino
40 – Fastuchina
7 – Bari Sabittisa
27 – Chiricupara
16 – Cacinova
67 – Mullisa Piccola
91 – Selvatica Favata
33 – Di Giorgio
32 – Cuti
59 – Mezza lira
28 – Comunista
43 – Fellamasa Casteltermini
26 – Chiarchiara
25 – Cesaro 1
3 – Angelica
8 – Barunissa
58 – Mennula du vattiu
92 – Staccia
4 – Baggiana
87 – Sarbaggia di Sciascia
1 – Acquaviva
15 – Cacciatura
51 – Griddetta
68 – Nambaredda
89 – Sarbaggia di Patito
23 – Cavalera Naro
49 – Genco
99 – Universo
30 – Cuore
55 – Marra Mennuladi
56 – Mastracicia
96 – Tricula
64 – Montagna
72 – Palma
95 – Texas
48 – Gaglio
21 – Catrubba
83 – Reginella
17 – Calamonaci
80 – Pizzuta Grande
62 – Miricanedda
84 – Romana Ispica
47 – Filippazzo
20 – Carrubina
2 – Amara di Martorana
52 – Laurenne
97 – Tunnulidda
13 – Bronte 1
19 – Caluriedda
53 – Liscianarisa
88 – Sarbaggia di Vitello
41 – Favaro
37 – Enna 2
46 – Filippoceo
71 – Nuciddara
34 – Don Filippo
54 – Lumia
45 – Ferragnes
102 – Zaccaneddara
94 – Tabacchina
82 – Regina
73 – Perciavisazza
5 – Bargellera
50 – Giardinella
38 – Falsa pizzuta
106 – Zottafunnuta
61 – Mirabile
12 – Bottara
93 – Supernova
39 – Fascionello
86 – Sancisuca
36 – Don Pitrino
100 – Uova di Cucco
103 – Zagarri'
81 – Rapparina
65 – Mullisa Tonda
31 – Cupani piccola
6 – Bari Flores
63 – Miuzza
35 – Don Peppino
101 – Vaiana
90 – Scummissa
105 – Zarbara
42 – Favaro' 2
18 – Callara
75 – Piattamollisa
66 – Mullisa Grande
14 – Buscemi
76 – Pilusedda
98 – Tuono
79 – Pizzutella
69 – Naro 1
24 – Cavaliere
57 – Mennula du nigliu
74 – Persichina
29 – Cumma
70 – Niveramanza
60 – Milocca
104 – Zammuto 2
22 – Cavalera Aragona
77 – Pizzuta Contino
85 – Romana Licata
10 – Bennici
44 – Ferraduel
Apulia
International
Sicily
B
Concentration, ppbV
0
10
20
30
40
50
raw
roasted
C
cor = 0.25 p.value = 0
roasted
0
25
50
75
100
125
raw
0
10
20
30

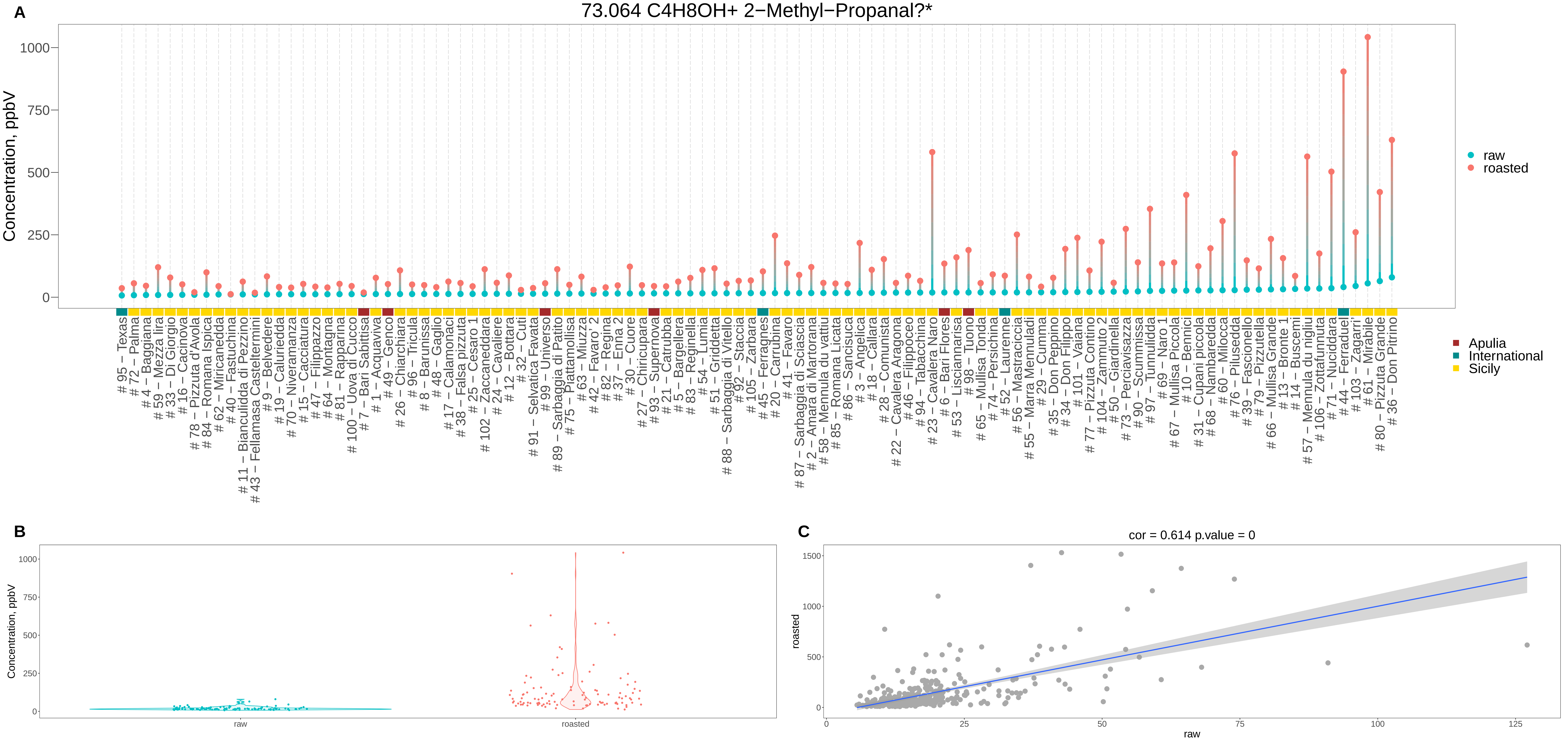

A
73.064 C4H8OH+ 2−Methyl−Propanal?*
Concentration, ppbV
0
250
500
750
1000
raw
roasted
Apulia
International
Sicily
95 – Texas
72 – Palma
4 – Baggiana
59 – Mezza lira
33 – Di Giorgio
16 – Cacinova
78 – Pizzuta d'Avola
84 – Romana Ispica
62 – Miricaneddu
40 – Fastuchina
11 – Bianculidda di Pezzino
43 – Fellamasa Casteltermini
9 – Belvedere
19 – Calurieddu
70 – Niveramanza
15 – Cacciatura
47 – Filippazzo
64 – Montagna
81 – Rapparina
100 – Uova di Cucco
7 – Bari Sabittisa
1 – Acquaviva
49 – Genco
26 – Chiarchiara
96 – Tricula
8 – Barunissa
48 – Gaglio
17 – Calamonaci
38 – Falsa pizzuta
25 – Cesaro 1
102 – Zaccaneddara
24 – Cavaliere
12 – Bottara
32 – Cuti
91 – Selvatica Favata
99 – Universo
89 – Sarbaggia di Patito
75 – Piattamollisa
63 – Miuzza
42 – Favaro' 2
82 – Regina
37 – Enna 2
30 – Cuore
27 – Chiricupara
93 – Supernova
21 – Catrubba
5 – Bargellera
83 – Reginella
54 – Lumia
51 – Griddetta
88 – Sarbaggia di Vitello
92 – Staccia
105 – Zarbara
45 – Ferragnes
20 – Carrubina
41 – Favaro
87 – Sarbaggia di Sciascia
2 – Amara di Martorana
58 – Mennula du vattiu
85 – Romana Licata
86 – Sancisuca
3 – Angelica
18 – Callara
28 – Comunista
22 – Cavalera Aragona
46 – Filippoceo
94 – Tabacchina
23 – Cavalera Naro
6 – Bari Flores
53 – Lisciannarisa
98 – Tuono
65 – Mullisa Tonda
74 – Persichina
52 – Laurenne
56 – Mastracicca
55 – Marra Mennuladi
29 – Cumma
35 – Don Peppino
34 – Don Filippo
101 – Vaiana
77 – Pizzuta Contino
104 – Zammuto 2
50 – Giardinella
73 – Perciavisazza
90 – Scummissa
97 – Tunnulidda
69 – Naro 1
67 – Mullisa Piccola
10 – Bennici
31 – Cupani piccola
68 – Nambaredda
60 – Milocca
76 – Pilusedda
39 – Fascionello
79 – Pizzutella
66 – Mullisa Grande
13 – Bronte 1
14 – Buscemi
57 – Mennula du nigliu
106 – Zottafunnuta
71 – Nuciddara
44 – Ferraduel
103 – Zagarri'
61 – Mirabile
80 – Pizzuta Grande
36 – Don Pitrino
B
Concentration, ppbV
0
250
500
750
1000
raw
roasted
C
cor = 0.614 p.value = 0
roasted
0
500
1000
1500
0
25
50
75
100
125
raw

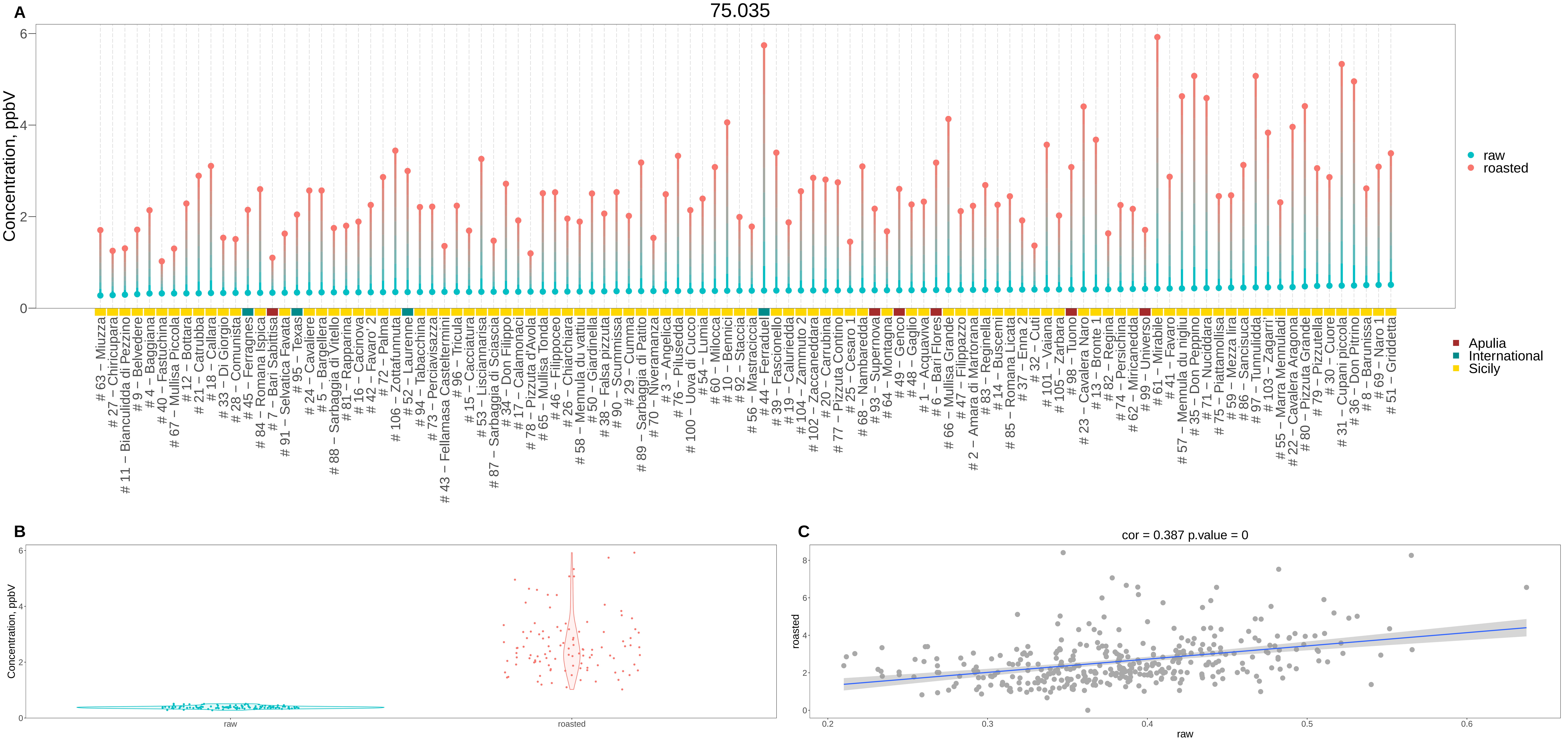
A
75.035
Concentration, ppbV
0
2
4
6
raw
roasted
Apulia
International
Sicily
63 – Miuzza
27 – Chiricupara
11 – Bianculidda di Pezzino
9 – Belvedere
4 – Baggiana
40 – Fastuchina
67 – Mullisa Piccola
12 – Bottara
21 – Catrubba
18 – Callara
33 – Di Giorgio
28 – Comunista
45 – Ferragnes
84 – Romana Ispica
7 – Bari Sabittisa
91 – Selvatica Favata
95 – Texas
24 – Cavaliere
5 – Bargellera
88 – Sarbaggia di Vitello
81 – Rapparina
16 – Cacinova
42 – Favaro' 2
72 – Palma
106 – Zottafunnuta
52 – Laurenne
94 – Tabacchina
73 – Perciavisazza
43 – Fellamasa Casteltermini
96 – Tricula
15 – Cacciatura
53 – Lisciannarisa
87 – Sarbaggia di Sciascia
34 – Don Filippo
17 – Calamonaci
78 – Pizzuta d'Avola
65 – Mullisa Tonda
46 – Filippoceo
26 – Chiarchiara
58 – Mennula du vattiu
50 – Giardinella
38 – Falsa pizzuta
90 – Scummissa
29 – Cumma
89 – Sarbaggia di Patito
70 – Niveramanza
3 – Angelica
76 – Pilusedda
100 – Uova di Cucco
54 – Lumia
60 – Milocca
10 – Bennici
92 – Staccia
56 – Mastraciccia
44 – Ferraduel
39 – Fascionello
19 – Caluriedda
104 – Zammuto 2
102 – Zaccaneddara
20 – Carrubina
77 – Pizzuta Contino
25 – Cesaro 1
68 – Nambaredda
93 – Supernova
64 – Montagna
49 – Genco
48 – Gaglio
1 – Acquaviva
6 – Bari Flores
66 – Mullisa Grande
47 – Filippazzo
2 – Amara di Martorana
83 – Reginella
14 – Buscemi
85 – Romana Licata
37 – Enna 2
32 – Cuti
101 – Vaiana
105 – Zarbara
98 – Tuono
23 – Cavalera Naro
13 – Bronte 1
82 – Regina
74 – Persichina
62 – Miricanedda
99 – Universo
61 – Mirabile
41 – Favaro
57 – Mennula du nigliu
35 – Don Peppino
71 – Nuciddara
75 – Piattamollisa
59 – Mezza lira
86 – Sancisuca
97 – Tunnulidda
103 – Zagarri'
55 – Marra Mennuladi
22 – Cavalera Aragona
80 – Pizzuta Grande
79 – Pizzutella
30 – Cuore
31 – Cupani piccola
36 – Don Pitrino
8 – Barunissa
69 – Naro 1
51 – Griddetta
B
Concentration, ppbV
0
2
4
6
raw
roasted
C
cor = 0.387 p.value = 0
roasted
0
2
4
6
8
raw
0.2
0.3
0.4
0.5
0.6

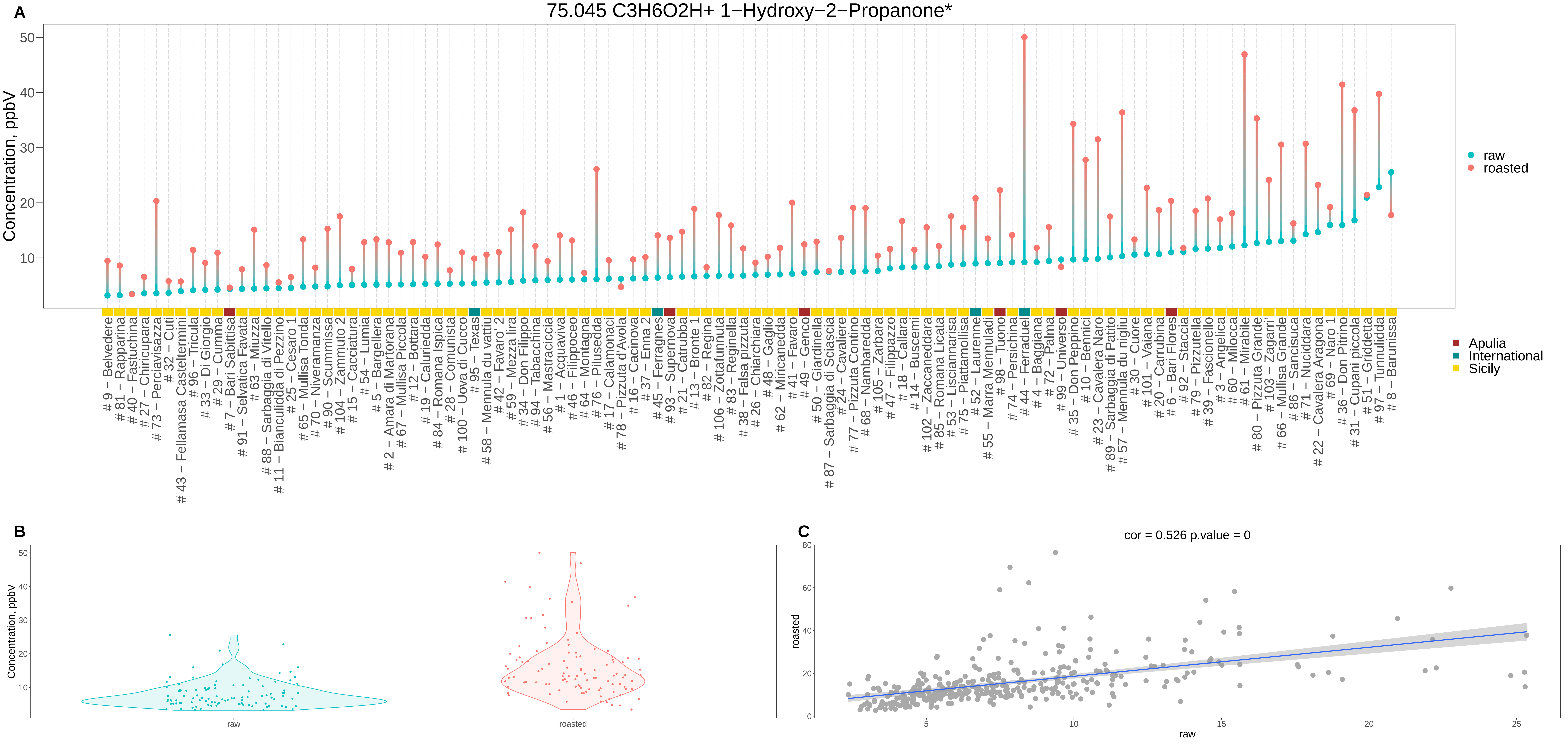

A
75.045 C3H6O2H+ 1−Hydroxy−2−Propanone*
Concentration, ppbV
raw
roasted
Apulia
International
Sicily
B
Concentration, ppbV
raw
roasted
C
cor = 0.526 p.value = 0
roasted
raw

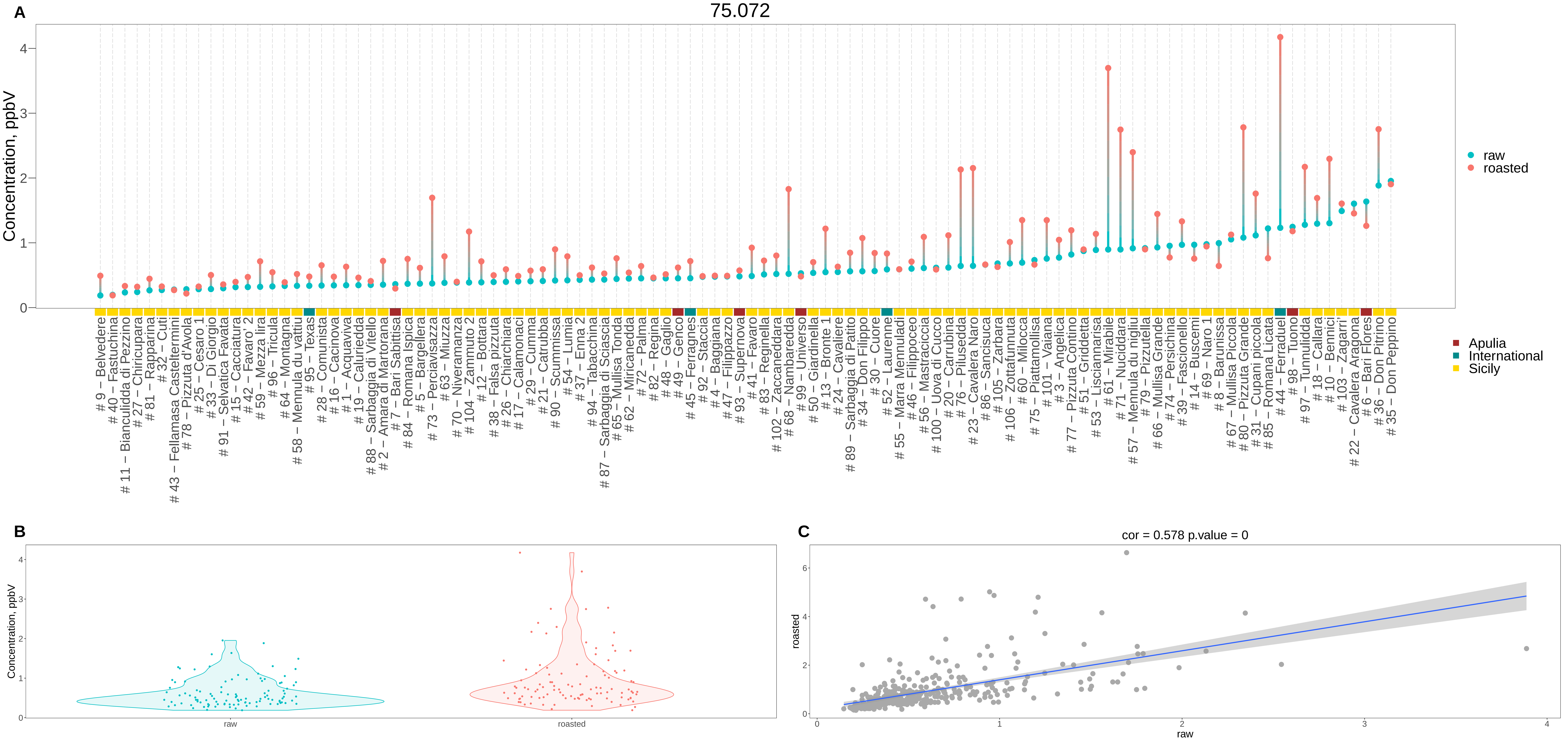

A
75.072
Concentration, ppbV
raw
roasted
Apulia
International
Sicily
9 – Belvedere
40 – Fastuchina
11 – Bianculidda di Pezzino
27 – Chiricupara
81 – Rapparina
32 – Cuti
43 – Fellamasa Casteltermini
78 – Pizzuta d'Avola
25 – Cesaro 1
33 – Di Giorgio
91 – Selvatica Favata
15 – Cacciatura
42 – Favaro' 2
59 – Mezza lira
96 – Tricula
64 – Montagna
58 – Mennula du vattiu
95 – Texas
28 – Comunista
16 – Cacinova
1 – Acquaviva
19 – Caluriedda
88 – Sarbaggia di Vitello
2 – Amara di Martorana
7 – Bari Sabitisa
84 – Romana Ispica
5 – Bargellera
73 – Perciavisazza
63 – Miuzza
70 – Niveramanza
104 – Zammuto 2
12 – Bottara
38 – Falsa pizzuta
26 – Chiarchiara
17 – Calamonaci
29 – Cumma
21 – Catrubba
90 – Scummissa
54 – Lumia
37 – Enna 2
94 – Tabacchina
87 – Sarbaggia di Sciascia
65 – Mullisa Tonda
62 – Miricanedda
72 – Palma
82 – Regina
48 – Gaglio
49 – Genco
45 – Ferragnes
92 – Staccia
4 – Baggiana
47 – Filippazzo
93 – Supernova
41 – Favaro
83 – Reginella
102 – Zaccaneddara
68 – Nambaredda
99 – Universo
50 – Giardinella
13 – Bronte 1
24 – Cavaliere
89 – Sarbaggia di Patito
34 – Don Filippo
30 – Cuore
52 – Laurenne
55 – Marra Mennuladi
46 – Filippoceo
56 – Mastraciccia
100 – Uova di Cucco
20 – Carrubina
76 – Pilusedda
23 – Cavalera Naro
86 – Sancisuca
105 – Zarbara
106 – Zottafunnuta
60 – Milocca
75 – Piattamollisa
101 – Vaiana
3 – Angelica
77 – Pizzuta Contino
51 – Griddetta
53 – Lisciannarisa
61 – Mirabile
71 – Nuciddara
57 – Mennula du nigliu
79 – Pizzutella
66 – Mullisa Grande
74 – Persichina
39 – Fascionello
14 – Busceni
69 – Naro 1
8 – Barunissa
67 – Mullisa Piccola
80 – Pizzuta Grande
31 – Cupani piccola
85 – Romana Licata
44 – Ferraduel
98 – Tuono
97 – Tunnulidda
18 – Callara
10 – Bennici
103 – Zagarri'
22 – Cavalera Aragona
6 – Bari Flores
36 – Don Pitrino
35 – Don Peppino
B
Concentration, ppbV
raw
roasted
C
cor = 0.578 p.value = 0
roasted
raw

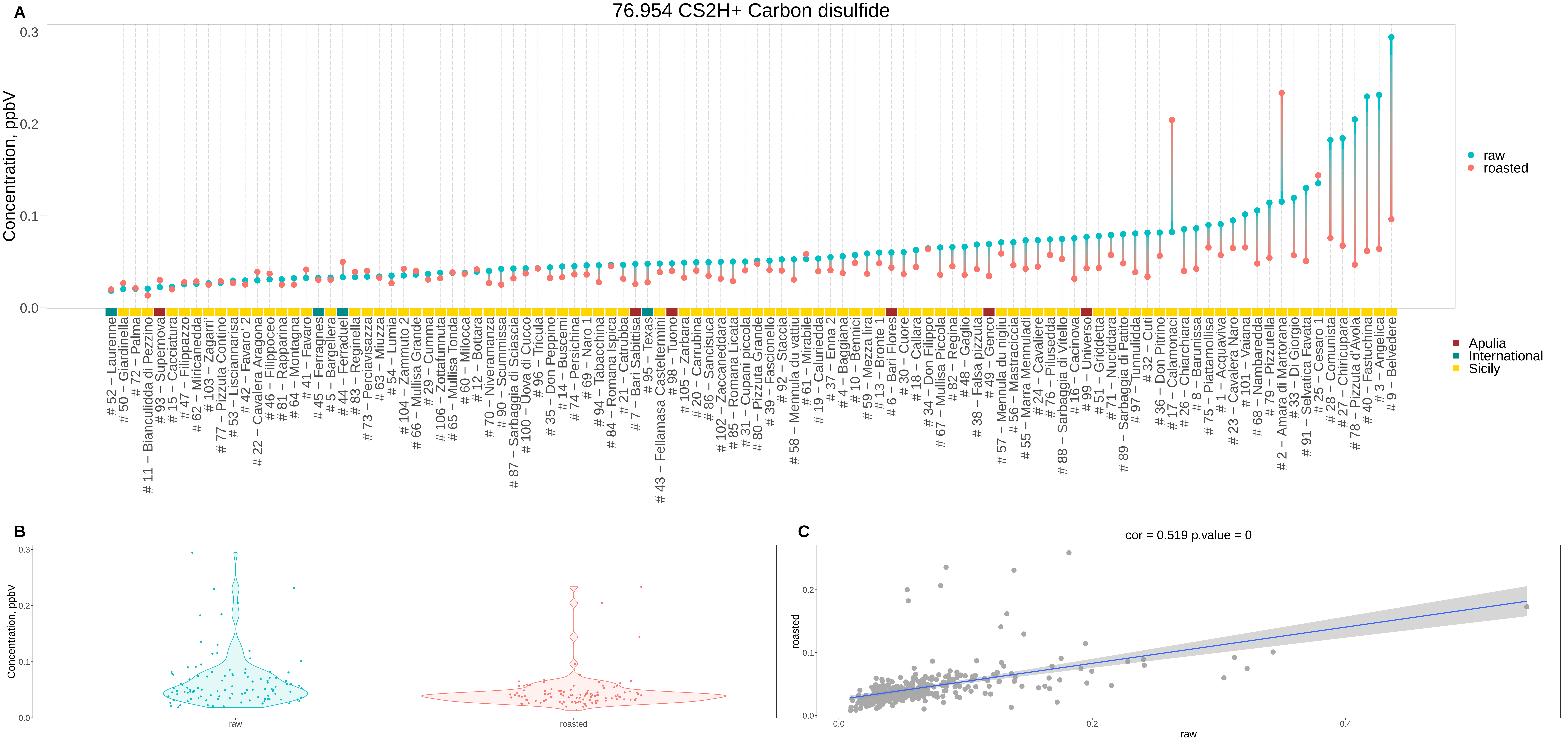
A
76.954 CS2H+ Carbon disulfide
Concentration, ppbV
0.0
0.1
0.2
0.3
raw
roasted
Apulia
International
Sicily
52 – Laurenne
50 – Giardinella
72 – Palma
11 – Biancullidda di Pezzino
93 – Supernova
15 – Cacciatura
47 – Filippazzo
62 – Miricanedda
103 – Zagarri'
77 – Pizzuta Contino
53 – Lisciannarisa
42 – Favaro' 2
22 – Cavalera Aragona
46 – Filippoceo
81 – Rappanina
64 – Montagna
41 – Favaro
45 – Ferragnes
5 – Bargellera
44 – Ferraduel
83 – Reginella
73 – Perciavisazza
63 – Miuzza
54 – Lumia
104 – Zammuto 2
66 – Mullisa Grande
29 – Cumma
106 – Zottafunnuta
65 – Mullisa Tonda
60 – Milocca
12 – Bottara
70 – Niveramanza
90 – Scummissa
87 – Sarbaggia di Sciascia
100 – Uova di Cucco
96 – Tricula
35 – Don Peppino
14 – Buscemi
74 – Persichina
69 – Naro 1
94 – Tabacchina
84 – Romana Ispica
21 – Catrubba
7 – Bari Sabittisa
95 – Texas
43 – Fellamasa Casteltermini
98 – Tuono
105 – Zarbara
20 – Carrubina
86 – Sancisuca
102 – Zaccaneddara
85 – Romana Licata
31 – Cupani piccola
80 – Pizzuta Grande
39 – Fascionello
92 – Staccia
58 – Mennula du vattiu
61 – Mirabile
19 – Caluriedda
37 – Enna 2
4 – Baggiana
10 – Bennici
59 – Mezza lira
13 – Bronte 1
6 – Bari Flores
30 – Cuore
18 – Callara
34 – Don Filippo
67 – Mullisa Piccola
82 – Regina
48 – Gaglio
38 – Falsa pizzuta
49 – Genco
57 – Mennula du nigliu
56 – Mastraciccia
55 – Marra Mennuladi
24 – Cavaliere
76 – Pilusedda
88 – Sarbaggia di Vitello
16 – Cacinova
99 – Universo
51 – Griddetta
71 – Nuciddara
89 – Sarbaggia di Patito
97 – Tunnulidda
32 – Cuti
36 – Don Pitrino
17 – Calamonaci
26 – Chiarchiara
8 – Barunissa
75 – Piattamollisa
1 – Acquaviva
23 – Cavalera Naro
101 – Vaiana
68 – Nambaredda
79 – Pizzutella
2 – Amara di Martorana
33 – Di Giorgio
91 – Selvatica Favata
25 – Cesaro 1
28 – Comunista
27 – Chiricupara
78 – Pizzuta d'Avola
40 – Fastuchina
3 – Angelica
9 – Belvedere
B
Concentration, ppbV
raw
roasted
C
cor = 0.519 p.value = 0
roasted
raw
0.0
0.2
0.4

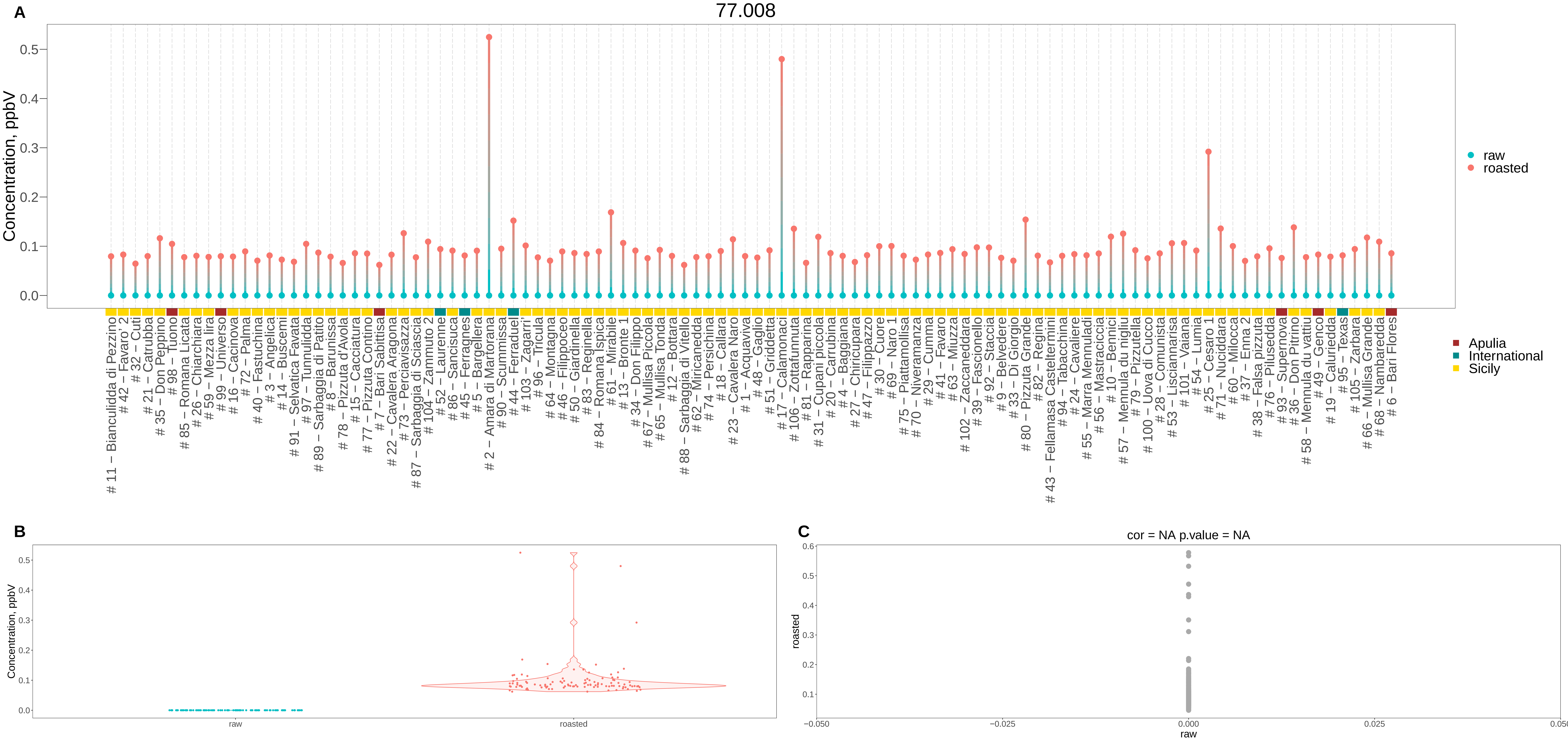
A
77.008
Concentration, ppbV
0.0
0.1
0.2
0.3
0.4
0.5
raw
roasted
Apulia
International
Sicily
11 – Bianculidda di Pezzino
42 – Favaro' 2
32 – Cuti
21 – Catrubba
35 – Don Peppino
98 – Tuono
85 – Romana Licata
26 – Chiarchiara
59 – Mezza lira
99 – Universo
16 – Cacinova
72 – Palma
40 – Fastuchina
3 – Angelica
14 – Buscemi
91 – Selvatica Favata
97 – Tunnulidda
89 – Sarbaggia di Patito
8 – Barunissa
78 – Pizzuta d'Avola
15 – Cacciatura
77 – Pizzuta Contino
7 – Bari Sabittisa
22 – Cavalera Aragona
73 – Perciavisazza
87 – Sarbaggia di Sciascia
104 – Zammuto 2
52 – Laurenne
86 – Sancisuca
45 – Ferragnes
5 – Bargellera
2 – Amara di Martorana
90 – Scummissa
44 – Ferraduel
103 – Zagarri
96 – Tricula
64 – Montagna
46 – Filippoceo
50 – Giardinella
83 – Reginella
84 – Romana Ispica
61 – Mirabile
13 – Bronte 1
34 – Don Filippo
67 – Mullisa Piccola
65 – Mullisa Tonda
12 – Bottara
88 – Sarbaggia di Vitello
62 – Miricanedda
74 – Persichina
18 – Callara
23 – Cavalera Naro
1 – Acquaviva
48 – Gaglio
51 – Griddetta
17 – Calamonaci
106 – Zottafunnuta
81 – Rapparina
31 – Cupani piccola
20 – Carrubina
4 – Baggiana
27 – Chiricupara
47 – Filipazzo
30 – Cuore
69 – Naro 1
75 – Piattamollisa
70 – Niveramanza
29 – Cummia
41 – Favaro
63 – Miuzza
102 – Zaccaneddara
39 – Fascionello
92 – Staccia
9 – Belvedere
33 – Di Giorgio
80 – Pizzuta Grande
82 – Regina
43 – Fellamasa Casteltermini
94 – Tabacchina
24 – Cavaliere
55 – Marra Mennuladi
56 – Mastraciccia
10 – Bennici
57 – Mennula du nigliu
79 – Pizzutella
100 – Uova di Cucco
28 – Comunista
53 – Lisciannarisa
101 – Vaiana
54 – Lumia
25 – Cesaro 1
71 – Nuciddara
60 – Milocca
37 – Enna 2
38 – Falsa pizzuta
76 – Pilusedda
93 – Supernova
36 – Don Pitrino
58 – Mennula du vattiu
49 – Genco
19 – Caluriedda
95 – Texas
105 – Zarbara
66 – Mullisa Grande
68 – Nambaredda
6 – Bari Flores
B
Concentration, ppbV
0.0
0.1
0.2
0.3
0.4
0.5
raw
roasted
C
cor = NA p.value = NA
roasted
0.1
0.2
0.3
0.4
0.5
0.6
−0.050
−0.025
0.000
0.025
raw

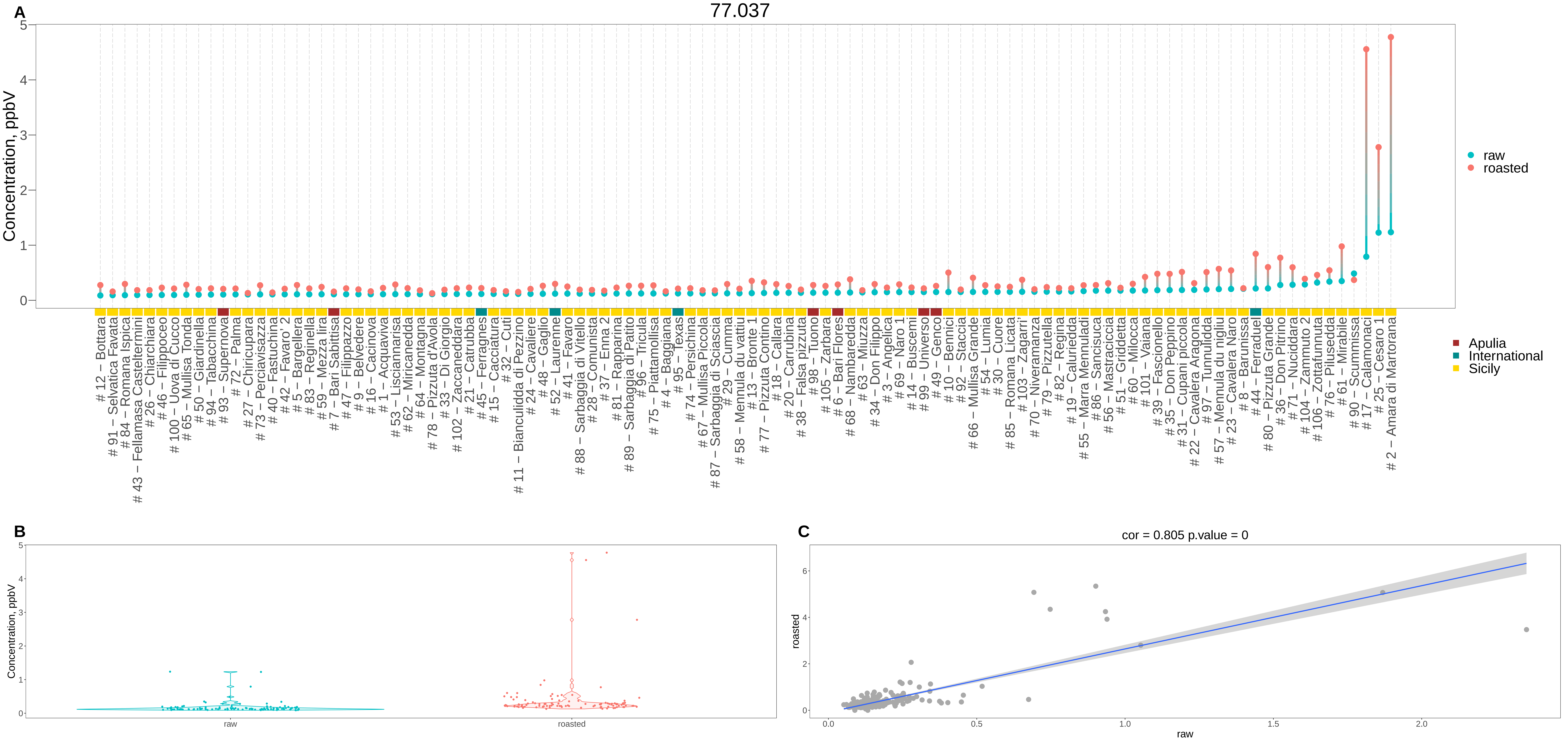

A
77.037
Concentration, ppbV
0
1
2
3
4
5
raw
roasted
Apulia
International
Sicily
12 – Bottara
91 – Selvatica Favata
84 – Romana Ispica
43 – Fellamasa Casteltermini
26 – Chiarchiara
46 – Filippoceo
100 – Uova di Cucco
65 – Mullisa Tonda
50 – Giardinella
94 – Tabacchina
93 – Supernova
72 – Palma
27 – Chiricupara
73 – Perciavisazza
40 – Fastuchina
42 – Favaro' 2
5 – Bargellera
83 – Reginella
59 – Mezza lira
7 – Bari Sabitissa
47 – Filippazzo
9 – Belvedere
16 – Cacinova
1 – Acquaviva
53 – Liscianarisa
62 – Miricannedda
64 – Montagna
78 – Pizzuta d'Avola
33 – Di Giorgio
102 – Zaccaneddara
21 – Catrubba
45 – Ferragnes
15 – Cacciatura
32 – Cuti
11 – Biancudda di Pezzino
24 – Cavaliere
48 – Gaglio
52 – Laurenne
41 – Favaro
88 – Sarbaggia di Vitello
28 – Comunista
37 – Enna 2
81 – Rappanina
89 – Sarbaggia di Patito
96 – Tricula
75 – Piattamollisa
4 – Baggiana
95 – Texas
74 – Persichina
67 – Mullisa Piccola
87 – Sarbaggia di Sciascia
29 – Cumma
58 – Mennula du vattiu
13 – Bronte 1
77 – Pizzuta Contino
18 – Callara
20 – Carrubina
38 – Falsa pizzuta
98 – Tuono
105 – Zarbara
6 – Bari Flores
68 – Nambaredda
63 – Miuzza
34 – Don Filippo
3 – Angelica
69 – Naro 1
14 – Busceni
99 – Universo
49 – Genco
10 – Bennici
92 – Staccia
66 – Mullisa Grande
54 – Lumia
30 – Cuore
85 – Romana Licata
103 – Zagarri'
70 – Niveramanza
79 – Pizzutella
82 – Regina
19 – Caluriedda
55 – Marra Mennuladi
86 – Sancisuca
56 – Mastracicia
51 – Griddetta
60 – Milocca
101 – Vaiana
39 – Fascionello
35 – Don Peppino
31 – Cupani piccola
22 – Cavalera Aragona
97 – Tunnulidda
57 – Mennula du nigliu
23 – Cavalera Naro
8 – Barunissa
44 – Ferraduel
80 – Pizzuta Grande
36 – Don Pitrino
71 – Nuciddara
104 – Zammuto 2
106 – Zottafunnuta
76 – Pilusedda
61 – Mirabile
90 – Scummissa
17 – Calamonaci
25 – Cesaro 1
2 – Amara di Martorana
B
Concentration, ppbV
raw
roasted
C
cor = 0.805 p.value = 0
roasted
raw
0.0
0.5
1.0
1.5
2.0
0
2
4
6

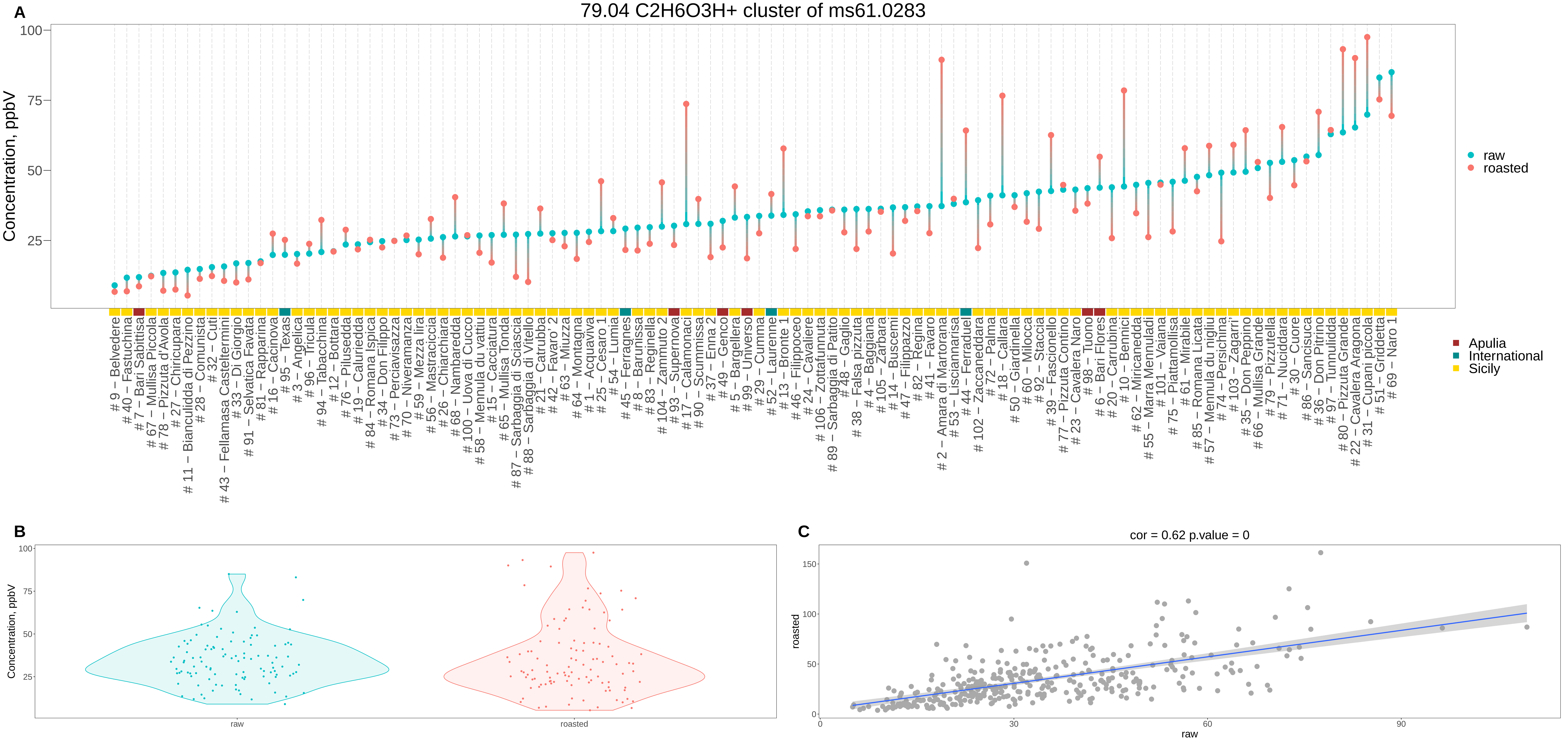
A
79.04 C2H6O3H+ cluster of ms61.0283
Concentration, ppbV
raw
roasted
Apulia
International
Sicily
9 – Belvedere
40 – Fastuchina
7 – Bari Sabitissa
67 – Mullisa Piccola
78 – Pizzuta d'Avola
27 – Chiricupara
11 – Bianculidda di Pezzino
28 – Comunista
32 – Cuti
43 – Fellamasa Casteltermini
33 – Di Giorgio
91 – Selvatica Favata
81 – Rapparina
16 – Cacinova
95 – Texas
3 – Angelica
96 – Tricula
94 – Tabacchina
12 – Bottara
76 – Pilusedda
19 – Caluriedda
84 – Romana Ispica
34 – Don Filippo
73 – Perciavisazza
70 – Niveramanza
59 – Mezza lira
56 – Mastraciccia
26 – Chiarchiara
68 – Nambaredda
100 – Uova di Cucco
58 – Mennula du vattiu
15 – Cacciatura
65 – Mullisa Tonda
87 – Sarbaggia di Sciascia
88 – Sarbaggia di Vitello
21 – Catrubba
42 – Favaro' 2
63 – Miuzza
64 – Montagna
1 – Acquaviva
25 – Cesaro 1
54 – Lumia
45 – Ferragnes
8 – Barunissa
83 – Reginella
104 – Zammuto 2
93 – Supernova
17 – Calamonaci
90 – Scummissa
37 – Enna 2
49 – Genco
5 – Bargellera
99 – Universo
29 – Cumma
52 – Laurenne
13 – Bronte 1
46 – Filippoceo
24 – Cavaliere
106 – Zottafunnuta
89 – Sarbaggia di Patito
48 – Gaglio
38 – Falsa pizzuta
4 – Baggiana
105 – Zarbara
14 – Buscemi
47 – Filippazzo
82 – Regina
41 – Favaro
2 – Amara di Martorana
53 – Liscianarisa
44 – Ferraduel
102 – Zaccaneddara
72 – Palma
18 – Callara
50 – Giardinella
60 – Milocca
92 – Stacia
39 – Fascionello
77 – Pizzuta Contino
23 – Cavalera Naro
98 – Tuono
6 – Bari Flores
20 – Carrubina
10 – Bennici
62 – Miricanedda
55 – Marra Mennuladi
101 – Vaiana
75 – Piattamollisa
61 – Mirabile
85 – Romana Licata
57 – Mennula du nigliu
74 – Persichina
103 – Zagarri'
35 – Don Peppino
66 – Mullisa Grande
79 – Pizzutella
71 – Nuciddara
30 – Cuore
86 – Sancisuca
36 – Don Pitrino
97 – Tunnulidda
80 – Pizzuta Grande
22 – Cavalera Aragona
31 – Cupani piccola
51 – Griddetta
69 – Naro 1
B
Concentration, ppbV
raw
roasted
C
cor = 0.62 p.value = 0
roasted
raw

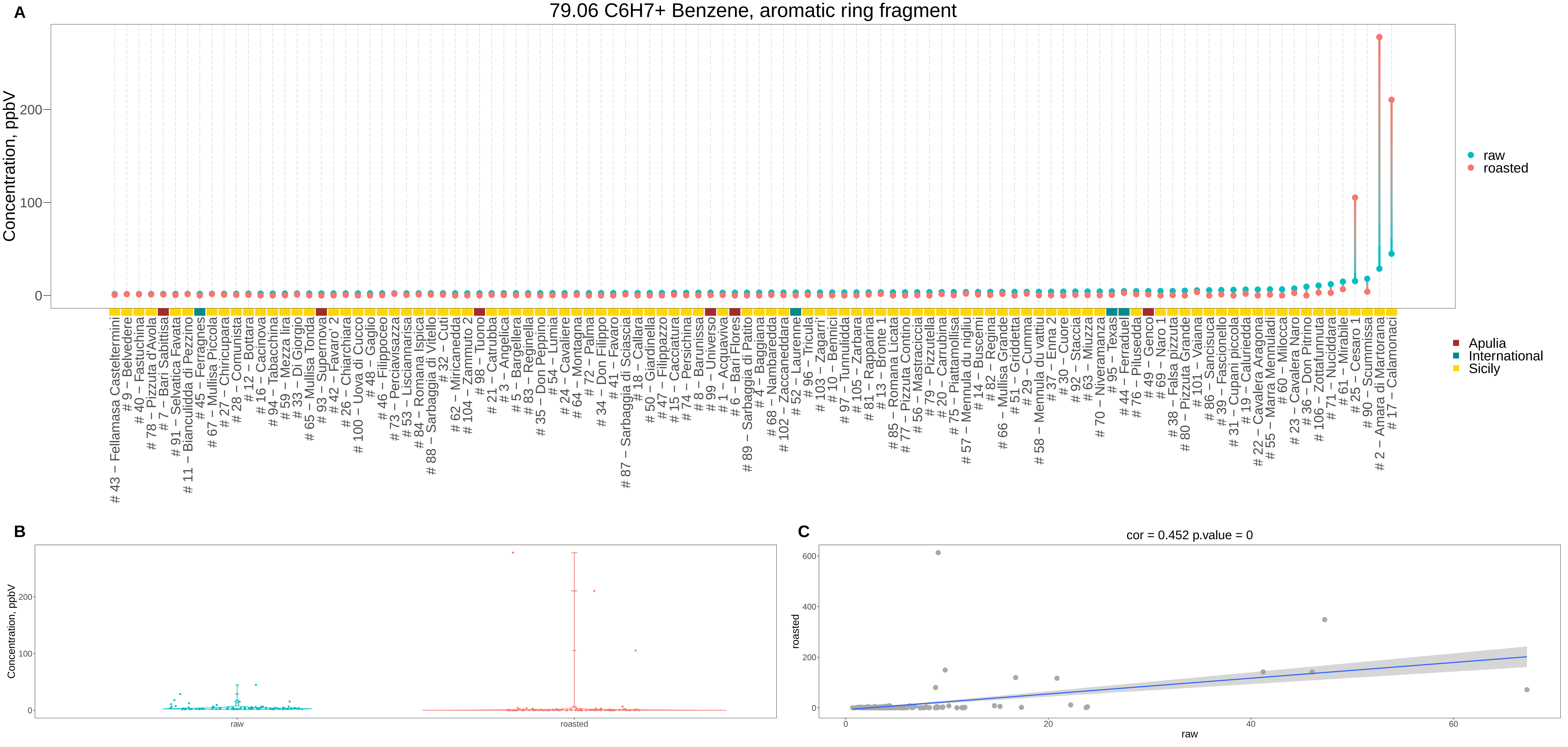
A
79.06 C6H7+ Benzene, aromatic ring fragment
Concentration, ppbV
0
100
200
raw
roasted
Apulia
International
Sicily
43 – Fellamasa Casteltermini
9 – Belvedere
40 – Fastuchina
78 – Pizzuta d'Avola
7 – Bari Sabitissa
91 – Selvatica Favata
11 – Bianculidda di Pezzino
45 – Ferragnes
67 – Mullisa Piccola
27 – Chiricupara
28 – Comunista
12 – Bottara
16 – Cacinova
94 – Tabacchina
59 – Mezza lira
33 – Di Giorgio
65 – Mullisa Tonda
93 – Supernova
42 – Favaro' 2
26 – Chiarchiara
100 – Uova di Cucco
48 – Gaglio
46 – Filippoceo
73 – Perciavisazza
53 – Lisciannarisa
84 – Romana Ispica
88 – Sarbaggia di Vitello
32 – Cuti
62 – Miricanedda
104 – Zammuto 2
98 – Tuono
21 – Catrubba
3 – Angelica
5 – Bargellera
83 – Reginella
35 – Don Peppino
54 – Lumia
24 – Cavaliere
64 – Montagna
72 – Palma
34 – Don Filippo
41 – Favaro
87 – Sarbaggia di Sciascia
18 – Callara
50 – Giardinella
47 – Filippazzo
15 – Cacciatura
74 – Persichina
8 – Barunissa
99 – Universo
1 – Acquaviva
6 – Bari Flores
89 – Sarbaggia di Patito
4 – Baggiana
68 – Nambaredda
102 – Zaccaneddara
52 – Laurenne
96 – Tricula
103 – Zagarri'
10 – Bennici
97 – Tunnulidda
105 – Zarbara
81 – Rapparina
13 – Bronte 1
85 – Romana Licata
77 – Pizzuta Contino
56 – Mastracicca
79 – Pizzutella
20 – Carrubina
75 – Piattamollisa
57 – Mennula du nigliu
14 – Buscemi
82 – Regina
66 – Mullisa Grande
51 – Griddetta
29 – Cumma
58 – Mennula du vattiu
37 – Enna 2
30 – Cuore
92 – Staccia
63 – Miuzza
70 – Niveramanza
95 – Texas
44 – Ferraduel
76 – Pilusedda
49 – Genco
69 – Naro 1
38 – Falsa pizzuta
80 – Pizzuta Grande
101 – Vaiana
86 – Sancisuca
39 – Fascionello
31 – Cupani piccola
19 – Caluriedda
22 – Cavalera Aragona
55 – Marra Mennuladi
60 – Milocca
23 – Cavalera Naro
36 – Don Pitrino
106 – Zottafunnuta
71 – Nuciddara
61 – Mirabile
25 – Cesaro 1
90 – Scummissa
2 – Amara di Martorana
17 – Calamonaci
B
Concentration, ppbV
0
100
200
raw
roasted
C
cor = 0.452 p.value = 0
roasted
0
200
400
600
raw
0
20
40
60

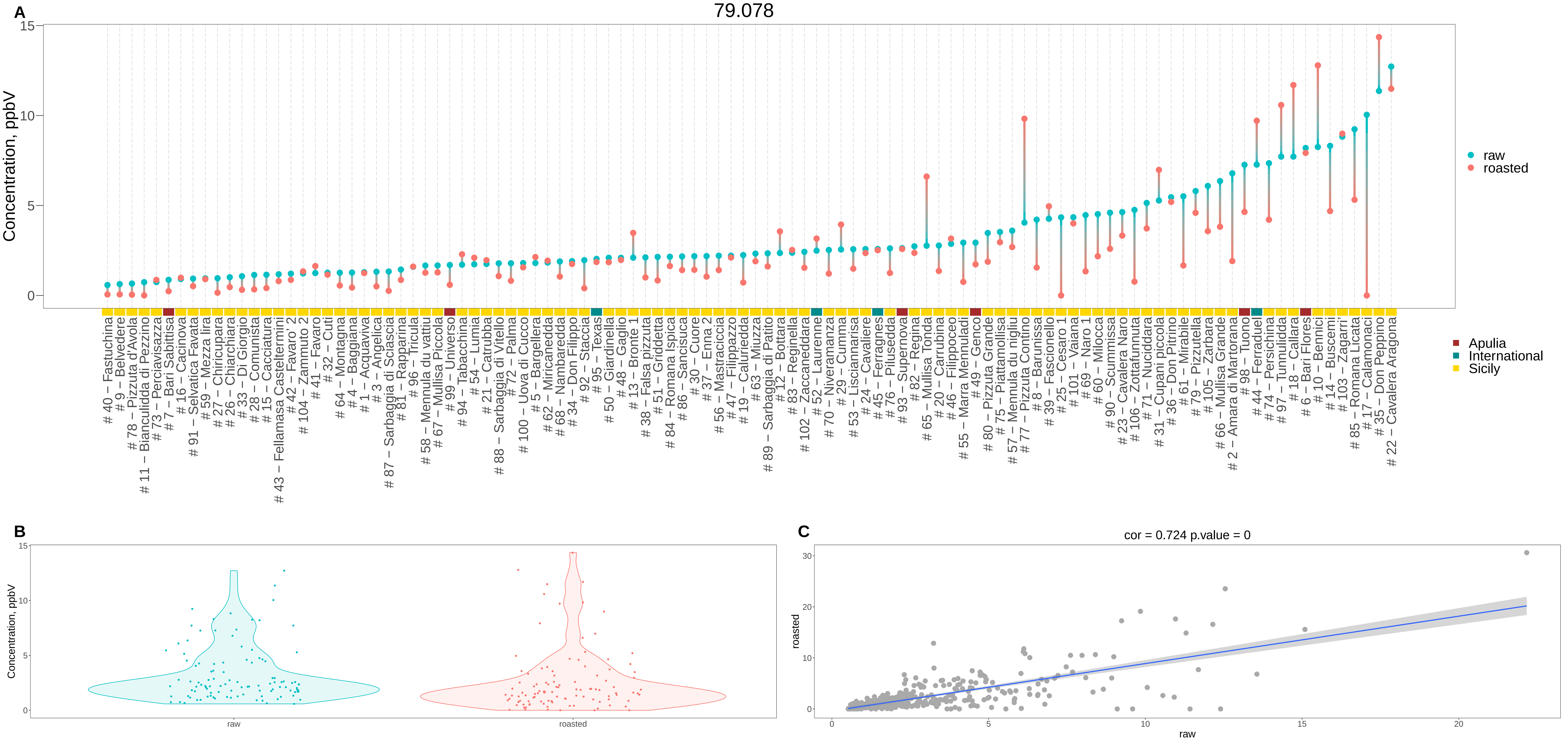

A
79.078
Concentration, ppbV
0
5
10
15
raw
roasted
Apulia
International
Sicily
40 – Fastuchina
9 – Belvedere
78 – Pizzuta d'Avola
11 – Bianculidda di Pezzino
73 – Perciavisazza
7 – Bari Sabitisa
16 – Cacinova
91 – Selvatica Favata
59 – Mezza lira
27 – Chiricupara
26 – Chiarchiara
33 – Di Giorgio
28 – Comunista
15 – Cacciatura
43 – Fellamasa Casteltermini
42 – Favaro' 2
104 – Zammuto 2
41 – Favaro
32 – Cuti
64 – Montagna
4 – Baggiana
1 – Acquaviva
3 – Angelica
87 – Sarbaggia di Sciascia
81 – Rapparina
96 – Tricula
58 – Mennula du vattiu
67 – Mullisa Piccola
99 – Universo
94 – Tabacchina
54 – Lumia
21 – Catrubba
88 – Sarbaggia di Vitello
72 – Palma
100 – Uova di Cucco
5 – Bargellera
62 – Miricanedda
68 – Nambaredda
34 – Don Filippo
92 – Staccia
95 – Texas
50 – Giardinella
48 – Gaglio
13 – Bronte 1
38 – Falsa pizzuta
51 – Griddetta
84 – Romana Ispica
86 – Sancisuca
30 – Cuore
37 – Enna 2
56 – Mastraciccia
47 – Filippazzo
19 – Caluriedda
63 – Miuzza
89 – Sarbaggia di Patito
12 – Bottara
83 – Reginella
102 – Zaccaneddara
52 – Laurenne
70 – Niveramanza
29 – Cumma
53 – Lisciannarisa
24 – Cavaliere
45 – Ferragnes
76 – Pilusedda
93 – Supernova
82 – Regina
65 – Mullisa Tonda
20 – Carrubina
46 – Filippoceo
55 – Marra Mennuladi
49 – Genco
80 – Pizzuta Grande
75 – Piattamollisa
57 – Mennula du nigliu
77 – Pizzuta Continio
8 – Barunissa
39 – Fascionello
25 – Cesaro 1
101 – Vaiana
69 – Naro 1
60 – Milocca
90 – Scummissa
23 – Cavalera Naro
106 – Zottafunnuta
71 – Nuciddara
31 – Cupani piccola
36 – Don Pitrino
61 – Mirabile
79 – Pizzutella
105 – Zarbara
66 – Mullisa Grande
2 – Amara di Martorana
98 – Tuono
44 – Ferraduel
74 – Persichina
97 – Tunnulidda
18 – Callara
6 – Bari Flores
10 – Bennici
14 – Buscemi
103 – Zagarri'
85 – Romana Licata
17 – Calamonaci
35 – Don Peppino
22 – Cavalera Aragona
B
Concentration, ppbV
0
5
10
15
raw
roasted
C
cor = 0.724 p.value = 0
roasted
raw
0
10
20
30
0
5
10
15
20

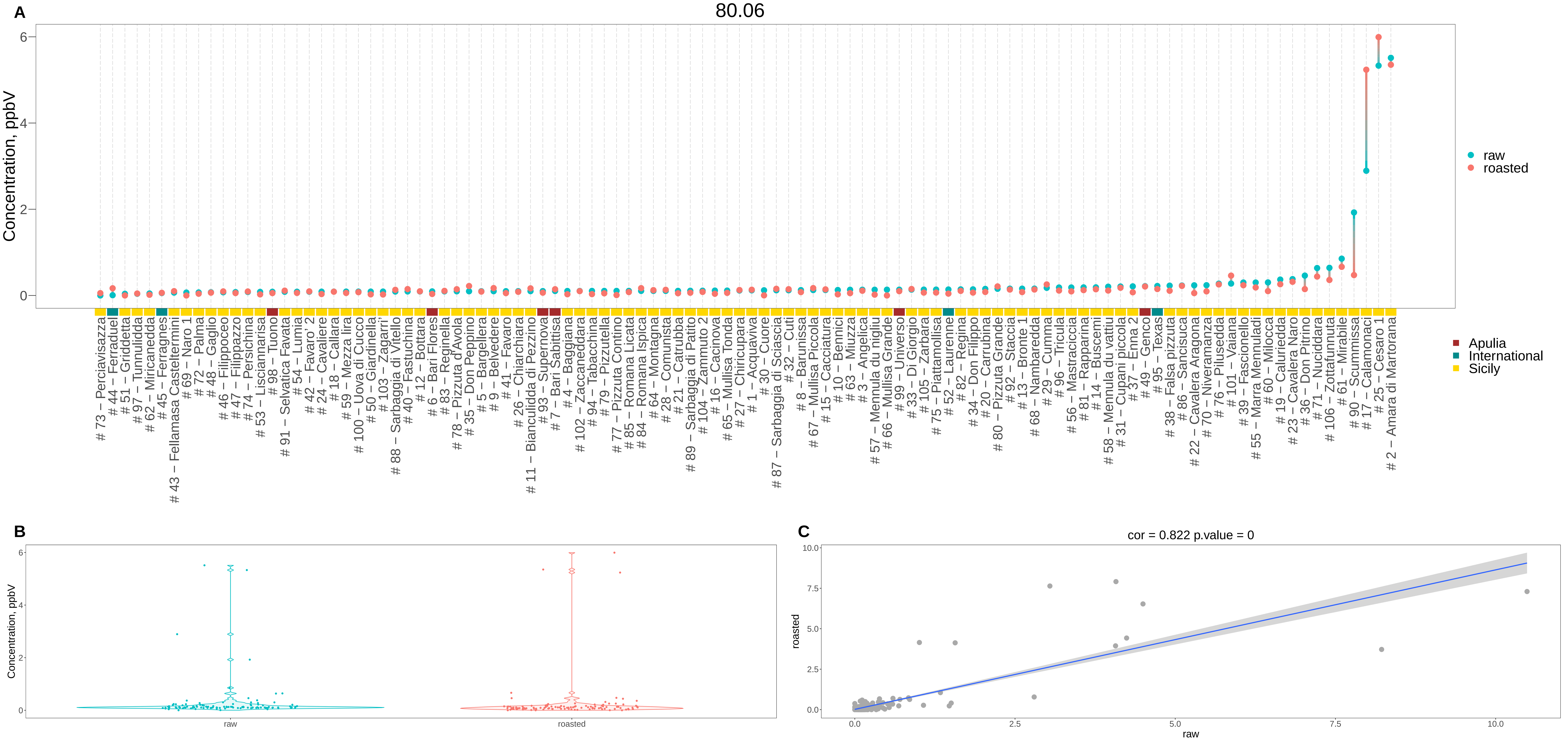

A
80.06
Concentration, ppbV
0
2
4
6
raw
roasted
Apulia
International
Sicily
73 – Perciavisazza
44 – Ferraduel
51 – Griddetta
97 – Tunnulidda
62 – Miricanedda
45 – Ferragnes
43 – Fellamasa Casteltermini
69 – Naro 1
72 – Palma
48 – Gaglio
46 – Filippoceo
47 – Filippazzo
74 – Persichina
53 – Lisciannarisa
98 – Tuono
91 – Selvatica Favata
54 – Lumia
42 – Favaro' 2
24 – Cavaliere
18 – Callara
59 – Mezza lira
100 – Uova di Cucco
50 – Giardinella
103 – Zagarri'
88 – Sarbaggia di Vitello
40 – Fastuchina
12 – Bottara
6 – Bari Flores
83 – Reginella
78 – Pizzuta d'Avola
35 – Don Peppino
5 – Bargellera
9 – Belvedere
41 – Favaro
26 – Chiarchiara
11 – Bianculidda di Pezzino
93 – Supernova
7 – Bari Sabitisa
4 – Baggiana
102 – Zaccaneddara
94 – Tabacchina
79 – Pizzutella
77 – Pizzuta Contino
85 – Romana Licata
84 – Romana Ispica
64 – Montagna
28 – Comunista
21 – Catrubba
89 – Sarbaggia di Patito
104 – Zammuto 2
16 – Cacinova
65 – Mullisa Tonda
27 – Chiricupara
1 – Acquaviva
30 – Cuore
87 – Sarbaggia di Sciascia
32 – Cuti
8 – Barunissa
67 – Mullisa Piccola
15 – Cacciatura
10 – Bennici
63 – Miuzza
3 – Angelica
57 – Mennula du nigliu
66 – Mullisa Grande
99 – Universo
33 – Di Giorgio
105 – Zarbara
75 – Piattamollisa
52 – Laurenne
82 – Regina
34 – Don Filippo
20 – Carrubina
80 – Pizzuta Grande
92 – Staccia
13 – Bronte 1
68 – Nambaredda
29 – Cumma
96 – Tricula
56 – Mastracicia
81 – Rapparina
14 – Buscemi
58 – Mennula du vattiu
31 – Cupani piccola
37 – Enna 2
49 – Genco
95 – Texas
38 – Falsa pizzuta
86 – Sancisuca
22 – Cavalera Aragona
70 – Niveramanza
76 – Pilusedda
101 – Vaiana
39 – Fascionello
55 – Marra Mennuladi
60 – Milocca
19 – Caluriedda
23 – Cavalera Naro
36 – Don Pitrino
71 – Nuciddara
106 – Zottafunnuta
61 – Mirabile
90 – Scummissa
17 – Calamonaci
25 – Cesaro 1
2 – Amara di Martorana
B
Concentration, ppbV
0
2
4
6
raw
roasted
C
cor = 0.822 p.value = 0
roasted
raw
0.0
2.5
5.0
7.5
10.0

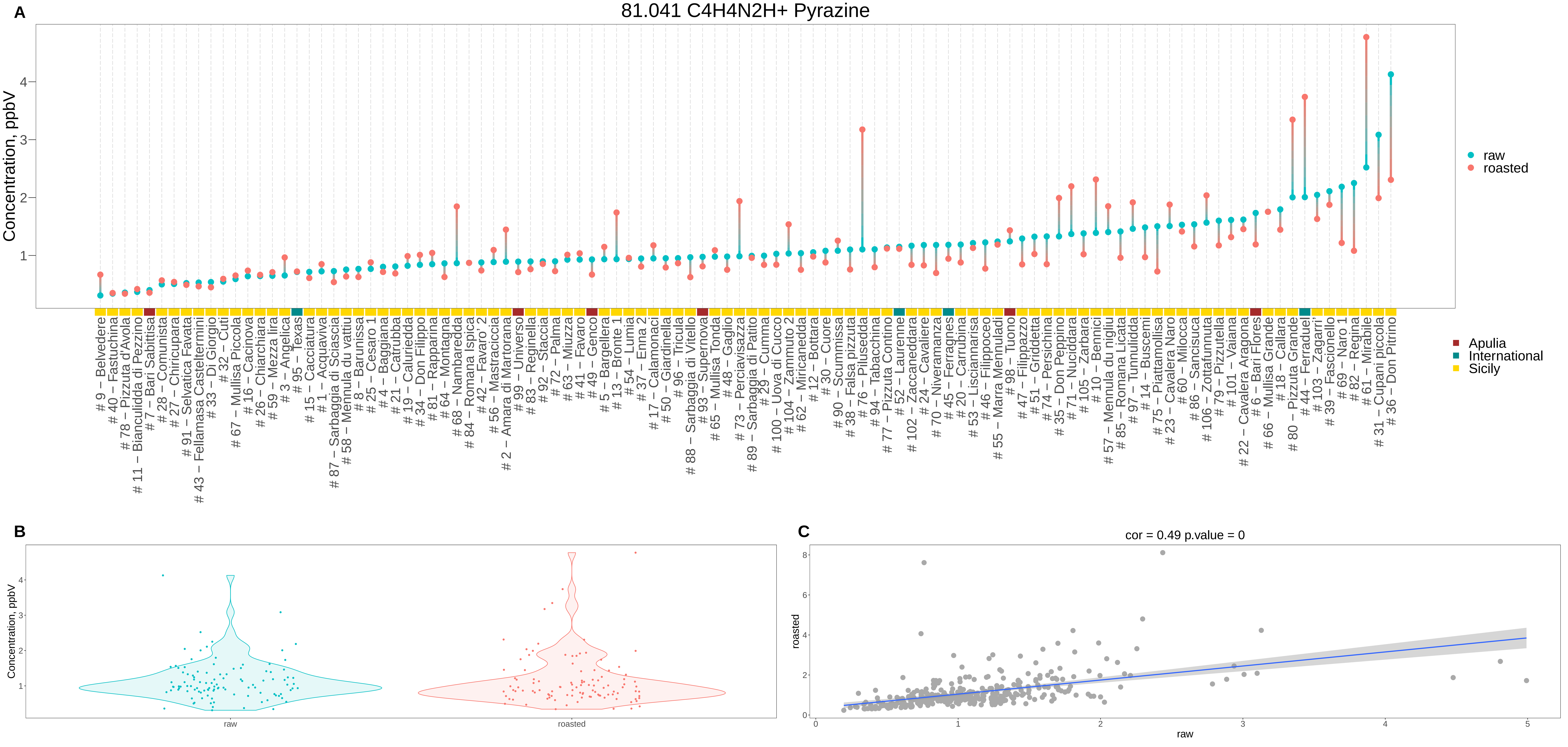

A
81.041 C4H4N2H+ Pyrazine
Concentration, ppbV
1
2
3
4
raw
roasted
Apulia
International
Sicily
9 – Belvedere
40 – Fastuchina
78 – Pizzuta d'Avola
11 – Bianculidda di Pezzino
7 – Bari Sabittisa
28 – Comunista
27 – Chiricupara
91 – Selvatica Favata
43 – Fellamasa Casteltermini
33 – Di Giorgio
32 – Cuti
67 – Mullisa Piccola
16 – Cacinova
26 – Chiarchiara
59 – Mezza lira
3 – Angelica
95 – Texas
15 – Cacciatura
1 – Acquaviva
87 – Sarbaggia di Sciascia
58 – Mennula du vattiu
8 – Barunissa
25 – Cesaro 1
4 – Baggiana
21 – Catrubba
19 – Caluriedda
34 – Don Filippo
81 – Rapparina
64 – Montagna
68 – Nambaredda
84 – Romana Ispica
42 – Favaro' 2
56 – Mastraciccia
2 – Amara di Martorana
99 – Universo
83 – Reginella
92 – Staccia
72 – Palma
63 – Miuzza
41 – Favaro
49 – Genco
5 – Bargellera
13 – Bronte 1
54 – Lumia
37 – Enna 2
17 – Calamonaci
50 – Giardinella
96 – Tricula
88 – Sarbaggia di Vitello
93 – Supernova
65 – Mullisa Tonda
48 – Gaglio
73 – Perciavisazza
89 – Sarbaggia di Patito
29 – Cumma
100 – Uova di Cucco
104 – Zammuto 2
62 – Miricanedda
12 – Bottara
30 – Cuore
90 – Scummissa
38 – Falsa pizzuta
76 – Pilusedda
94 – Tabacchina
77 – Pizzuta Contino
52 – Laurenne
102 – Zaccaneddara
24 – Cavaliere
70 – Niveramanza
45 – Ferragnes
20 – Carrubina
53 – Lisciannarisa
46 – Filippoceo
55 – Marra Mennuladi
98 – Tuono
47 – Filippazzo
51 – Griddetta
74 – Persichina
35 – Don Peppino
71 – Nuciddara
105 – Zarbara
10 – Bennici
57 – Mennula du nigliu
85 – Romana Licata
97 – Tunnulidda
14 – Buscemi
75 – Piattamollisa
23 – Cavalera Naro
60 – Milocca
86 – Sancisuca
106 – Zottafunnuta
79 – Pizzutella
101 – Vaiana
22 – Cavalera Aragona
6 – Bari Flores
66 – Mullisa Grande
18 – Callara
80 – Pizzuta Grande
44 – Ferraduel
103 – Zagarri'
39 – Fascionello
69 – Naro 1
82 – Regina
61 – Mirabile
31 – Cupani piccola
36 – Don Pitrino
B
Concentration, ppbV
raw
roasted
C
cor = 0.49 p.value = 0
roasted
raw
0
1
2
3
4
5
8
6
4
2
0

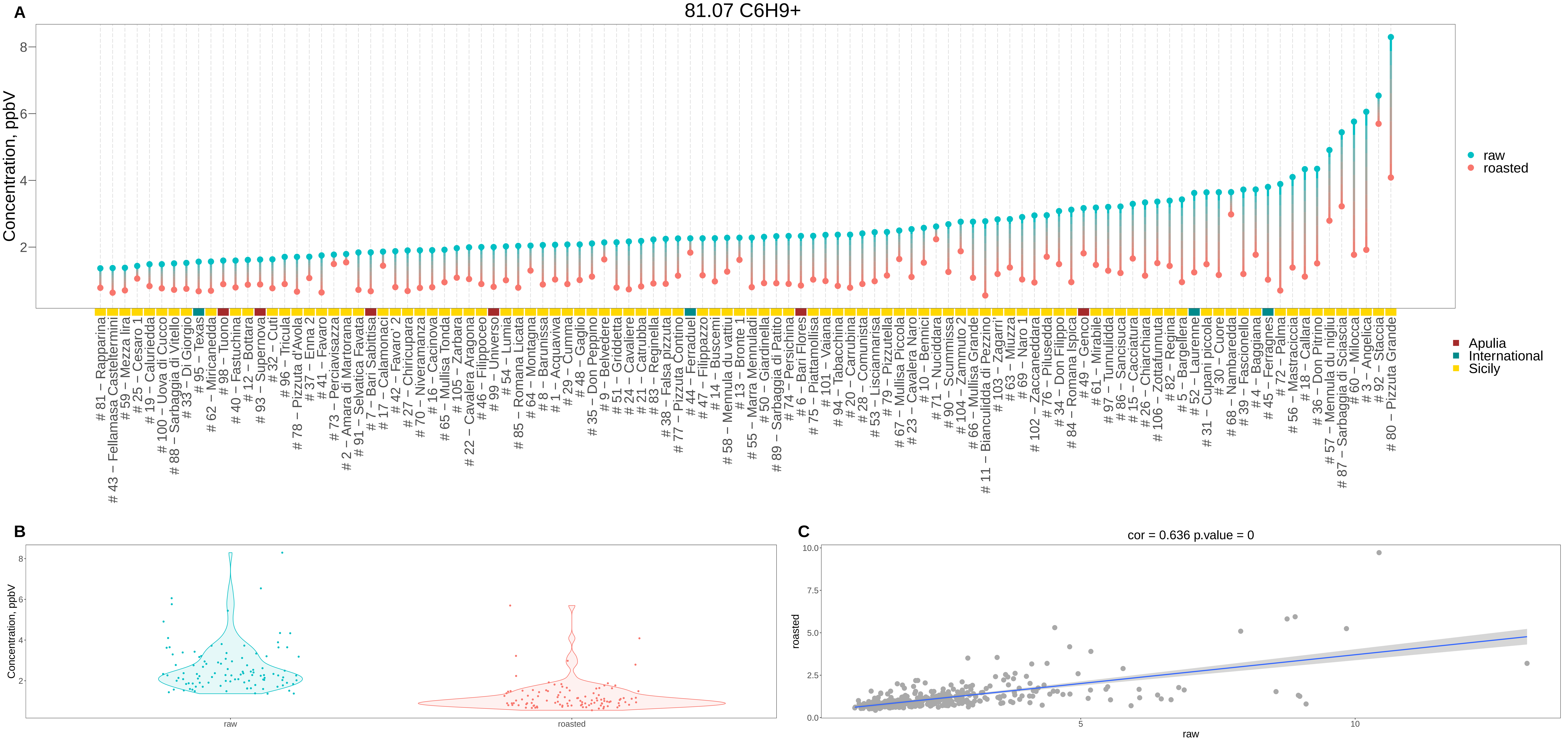
A
81.07 C6H9+
Concentration, ppbV
raw
roasted
Apulia
International
Sicily
B
Concentration, ppbV
raw
roasted
C
cor = 0.636 p.value = 0
roasted
raw

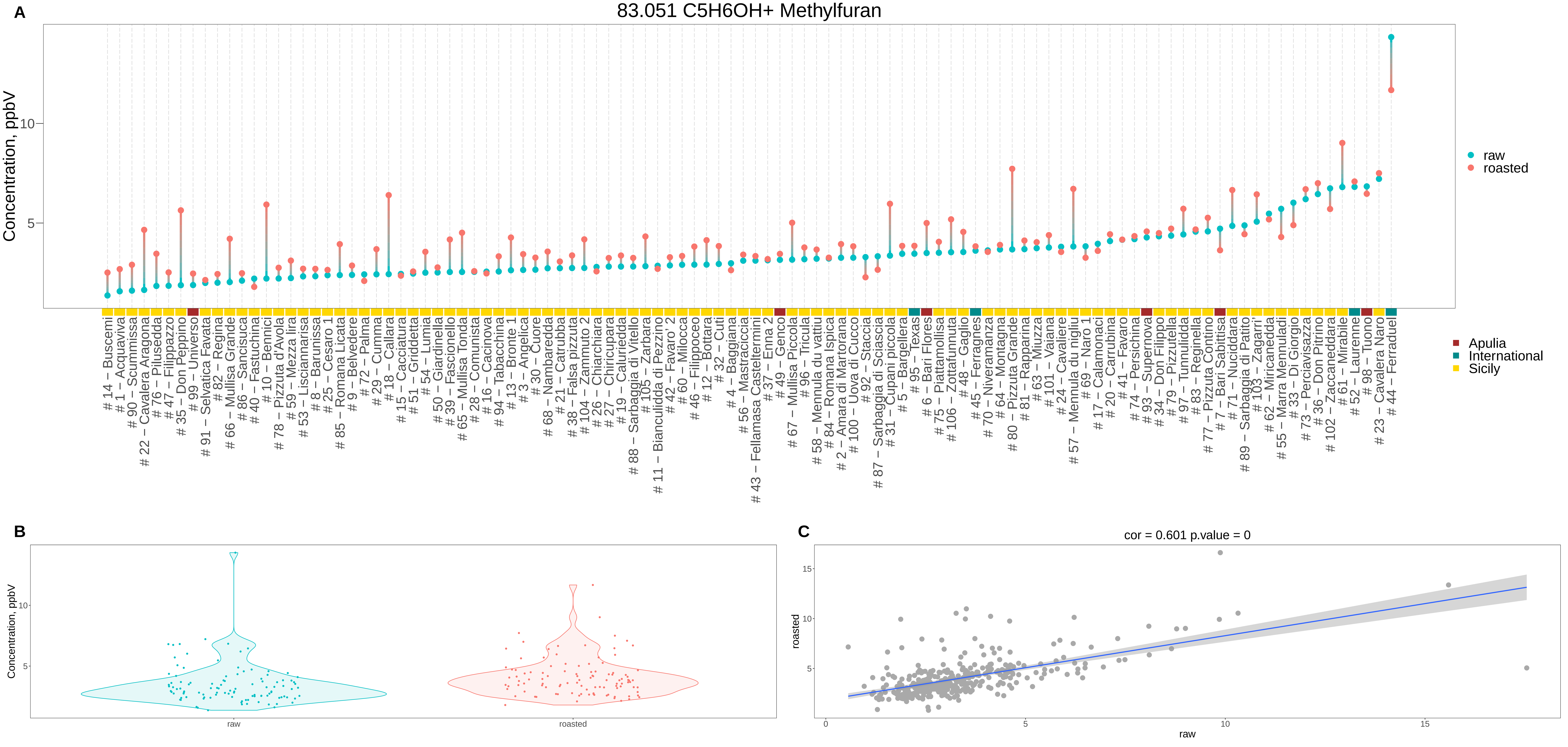

A
83.051 C5H6OH+ Methylfuran
Concentration, ppbV
5
10
raw
roasted
Apulia
International
Sicily
14 – Buscemi
1 – Acquaviva
90 – Scummissa
22 – Cavalera Aragona
76 – Pilusedda
47 – Filippazzo
35 – Don Peppino
99 – Universo
91 – Selvatica Favata
82 – Regina
66 – Mullisa Grande
86 – Sancisuca
40 – Fastuchina
10 – Bennici
78 – Pizzuta d'Avola
59 – Mezza lira
53 – Lisciannarisa
8 – Barunissa
25 – Cesaro 1
85 – Romana Licata
9 – Belvedere
72 – Palma
29 – Cumma
18 – Callara
15 – Cacciatura
51 – Griddetta
54 – Lumia
50 – Giardinella
39 – Fascionello
65 – Mullisa Tonda
28 – Comunista
16 – Cacinova
94 – Tabacchina
13 – Bronte 1
3 – Angelica
30 – Cuore
68 – Nambaredda
21 – Catrubba
38 – Falsa pizzuta
104 – Zammuto 2
26 – Chiarchiara
27 – Chiricupara
19 – Calurieddu
88 – Sarbaggia di Vitello
105 – Zarbara
11 – Biancuddida di Pezzino
42 – Favaro' 2
60 – Milocca
46 – Filippoceo
12 – Bottara
32 – Cuti
4 – Baggiana
56 – Mastraciccia
43 – Fellamasa Casteltermini
37 – Enna 2
49 – Genco
67 – Mullisa Piccola
96 – Tricula
58 – Mennula du vattiu
84 – Romana Ispica
2 – Amara di Martorana
100 – Uova di Cucco
92 – Staccia
87 – Sarbaggia di Sciascia
31 – Cupani piccola
5 – Bargellera
95 – Texas
6 – Bari Flores
75 – Piattamollisa
106 – Zottafunnuta
48 – Gaglio
45 – Ferragnes
70 – Niveramanza
64 – Montagna
80 – Pizzuta Grande
81 – Rapparina
63 – Miuzza
101 – Vaiana
24 – Cavaliere
57 – Mennula du nigliu
69 – Naro 1
17 – Calamonaci
20 – Carrubina
41 – Favaro
74 – Persichina
93 – Supernova
34 – Don Filippo
79 – Pizzutella
97 – Tunnulidda
83 – Reginella
77 – Pizzuta Contino
7 – Bari Sabittisa
71 – Nuciddara
89 – Sarbaggia di Patito
103 – Zagarri'
62 – Miricanedda
55 – Marra Mennuladi
33 – Di Giorgio
73 – Perciavisazza
36 – Don Pitrino
102 – Zaccaneddara
61 – Mirabile
52 – Laurenne
98 – Tuono
23 – Cavalera Naro
44 – Ferraduel
B
Concentration, ppbV
5
10
raw
roasted
C
cor = 0.601 p.value = 0
roasted
raw
0
5
10
15

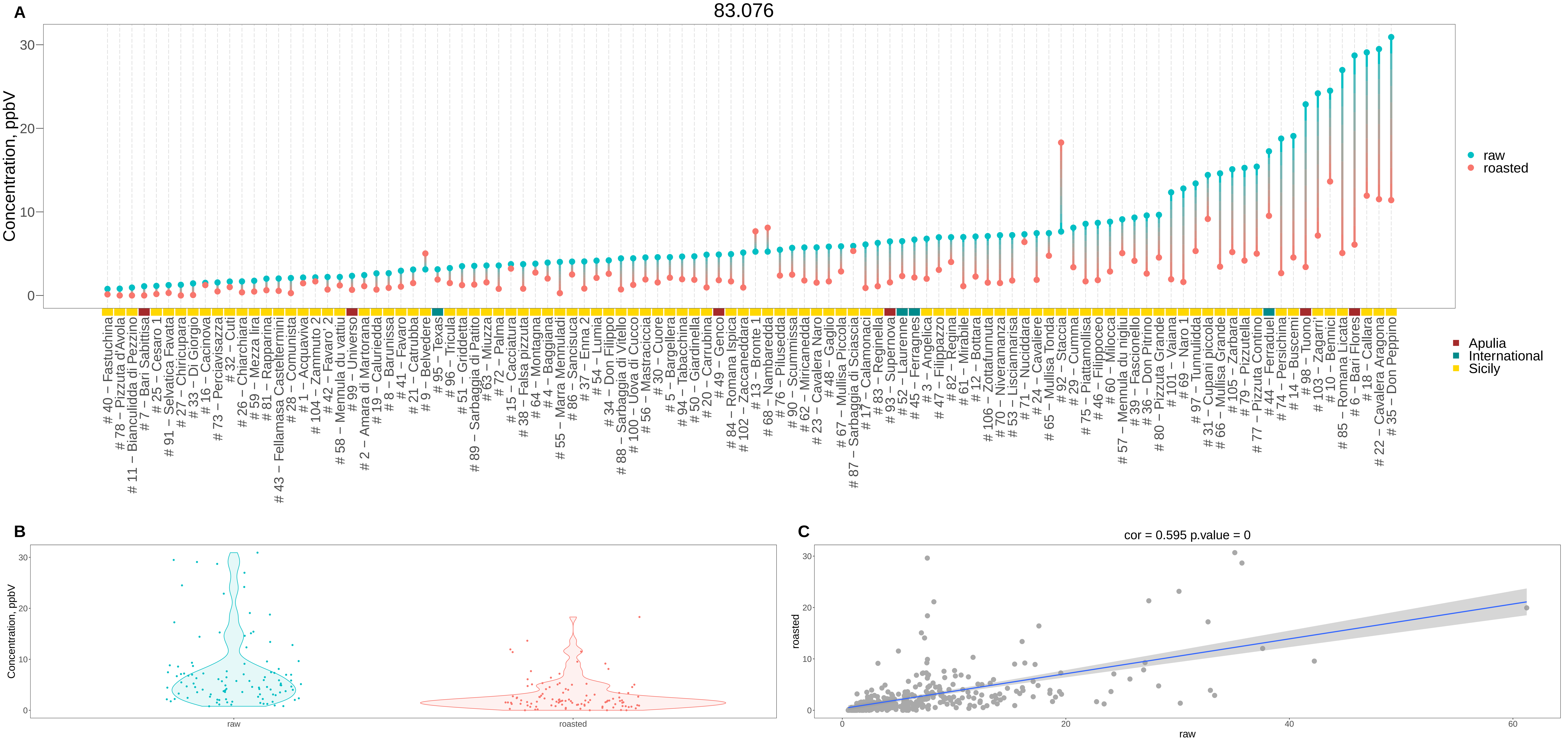

A
83.076
Concentration, ppbV
0
10
20
30
raw
roasted
Apulia
International
Sicily
40 – Fastuchina
78 – Pizzuta d'Avola
11 – Bianculidda di Pezzino
7 – Bari Sabitisa
25 – Cesaro 1
91 – Selvatica Favata
27 – Chiricupara
33 – Di Giorgio
16 – Cacinova
73 – Perciavisazza
32 – Cuti
26 – Chiarchiara
59 – Mezza lira
81 – Rapparina
43 – Fellamasa Casteltermini
28 – Comunista
1 – Acquaviva
104 – Zammuto 2
42 – Favaro' 2
58 – Mennula du vattiu
99 – Universo
2 – Amara di Martorana
19 – Caluriedda
8 – Barunissa
41 – Favaro
21 – Catrubba
9 – Belvedere
95 – Texas
96 – Tricula
51 – Griddetta
89 – Sarbaggia di Patito
63 – Miuzza
72 – Palma
15 – Cacciatura
38 – Falsa pizzuta
64 – Montagna
4 – Baggiana
55 – Marra Mennuladi
86 – Sancisuca
37 – Enna 2
54 – Lumia
34 – Don Filippo
88 – Sarbaggia di Vitello
100 – Uova di Cucco
56 – Mastracicia
30 – Cuore
5 – Bargellera
94 – Tabacchina
50 – Giardinella
20 – Carrubina
49 – Genco
84 – Romana Ispica
102 – Zaccaneddara
13 – Bronte 1
68 – Nambaredda
76 – Pilusedda
90 – Scummissa
62 – Miricanedda
23 – Cavalera Naro
48 – Gaglio
67 – Mullisa Piccola
87 – Sarbaggia di Sciascia
17 – Calamonaci
83 – Reginella
93 – Supernova
52 – Laurenne
45 – Ferragnes
3 – Angelica
47 – Filippazzo
82 – Regina
61 – Mirabile
12 – Bottara
106 – Zottafunnuta
70 – Niveramanza
53 – Lisciannarisa
71 – Nuciddara
24 – Cavaliere
65 – Mullisa Tonda
92 – Staccia
29 – Cumma
75 – Piattamollisa
46 – Filippoceo
60 – Milocca
57 – Mennula du nigliu
39 – Fascionello
36 – Don Pitrino
80 – Pizzuta Grande
101 – Vaiana
69 – Naro 1
97 – Tunnulidda
31 – Cupani piccola
66 – Mullisa Grande
105 – Zarbara
79 – Pizzutella
77 – Pizzuta Contino
44 – Ferraduel
74 – Persichina
14 – Buscemi
98 – Tuono
103 – Zagarri'
10 – Bennici
85 – Romana Licata
6 – Bari Flores
18 – Callara
22 – Cavalera Aragona
35 – Don Peppino
B
Concentration, ppbV
0
10
20
30
raw
roasted
C
cor = 0.595 p.value = 0
roasted
raw
0
10
20
30
0
20
40
60

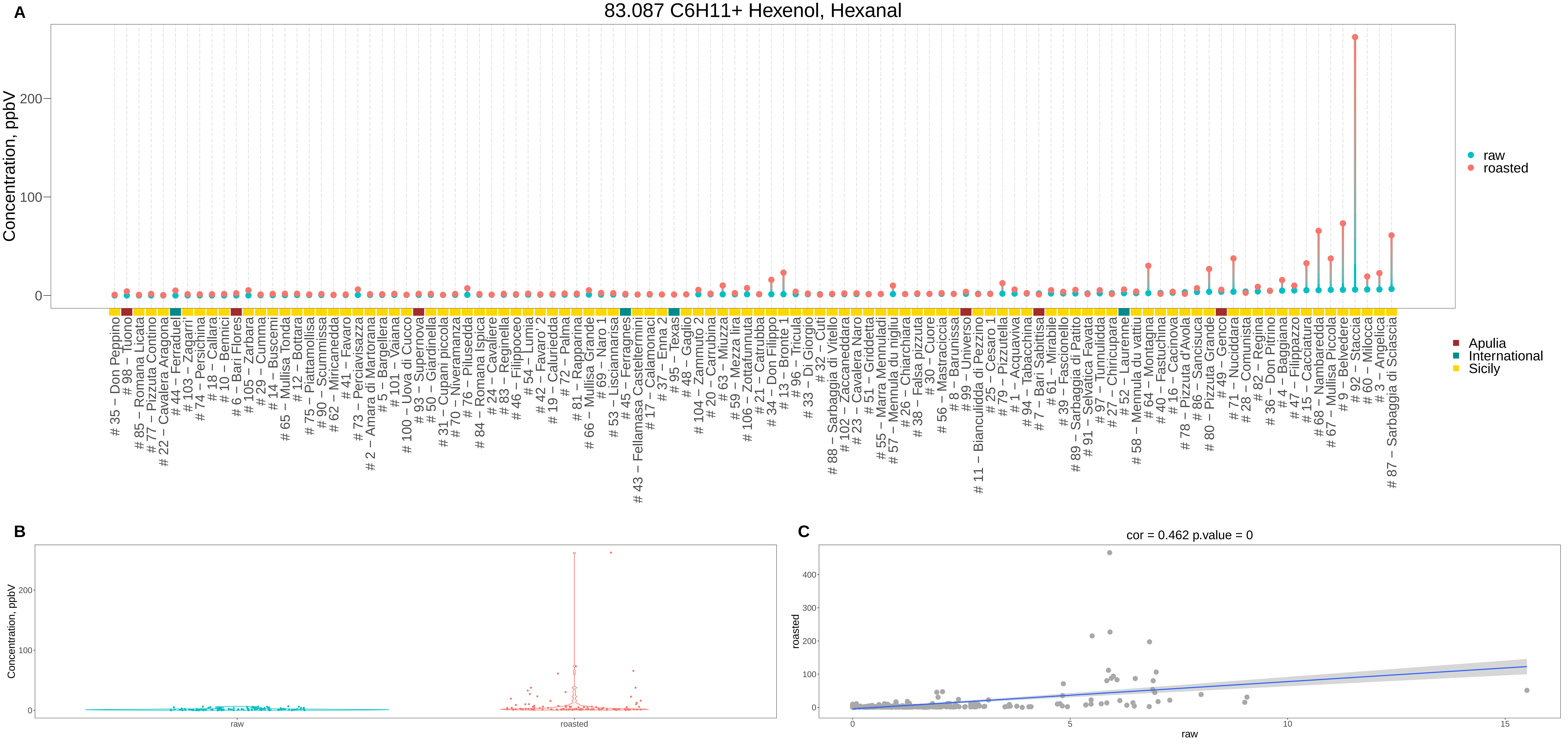

A
83.087 C6H11+ Hexenol, Hexanal
Concentration, ppbV
0
100
200
raw
roasted
Apulia
International
Sicily
35 – Don Peppino
98 – Tuono
85 – Romana Licata
77 – Pizzuta Contino
22 – Cavalera Aragona
44 – Ferraduel
103 – Zagarri'
74 – Persichina
18 – Callara
10 – Bennici
6 – Bari Flores
105 – Zarbara
29 – Cumma
14 – Busceni
65 – Mullisa Tonda
12 – Bottara
75 – Piattamollisa
90 – Scummissa
62 – Miricanedda
41 – Favaro
73 – Perciavisazza
2 – Amara di Martorana
5 – Bargellera
101 – Vaiana
100 – Uova di Cucco
93 – Supernova
50 – Giardinella
31 – Cupani piccola
70 – Niveramanza
76 – Pilusedda
84 – Romana Ispica
24 – Cavaliere
83 – Reginella
46 – Filippoceo
54 – Lumia
42 – Favaro' 2
19 – Caluriedda
72 – Palma
81 – Rapparina
66 – Mullisa Grande
69 – Naro 1
53 – Lisciannarisa
45 – Ferragnes
43 – Fellamasa Casteltermini
17 – Calamonaci
37 – Enna 2
95 – Texas
48 – Gaglio
104 – Zammuto 2
20 – Carrubina
63 – Miuzza
59 – Mezza lira
106 – Zottafunnuta
21 – Catrubba
34 – Don Filippo
13 – Bronte 1
96 – Tricula
33 – Di Giorgio
32 – Cuti
88 – Sarbaggia di Vitello
102 – Zaccaneddara
23 – Cavalera Naro
51 – Griddetta
55 – Marra Mennuladi
57 – Mennula du nigliu
26 – Chiarchiara
38 – Falsa pizzuta
30 – Cuore
56 – Mastraciccia
8 – Barunissa
99 – Universo
11 – Bianculidda di Pezzino
25 – Cesaro 1
79 – Pizzutella
1 – Acquaviva
94 – Tabacchina
7 – Bari Sabitisa
61 – Mirabile
39 – Fascionello
89 – Sarbaggia di Patito
91 – Selvatica Favata
97 – Tunnulidda
27 – Chiricupara
52 – Laurenne
58 – Mennula du vattiu
64 – Montagna
40 – Fastuchina
16 – Cacinova
78 – Pizzuta d'Avola
86 – Sancisuca
80 – Pizzuta Grande
49 – Genco
71 – Nuciddara
28 – Comunista
82 – Regina
36 – Don Pitrino
4 – Baggiana
47 – Filippazzo
15 – Cacciatura
68 – Nambaredda
67 – Mullisa Piccola
9 – Belvedere
92 – Staccia
60 – Milocca
3 – Angelica
87 – Sarbaggia di Sciascia
B
Concentration, ppbV
0
100
200
raw
roasted
C
cor = 0.462 p.value = 0
roasted
0
100
200
300
400
0
5
10
15
raw

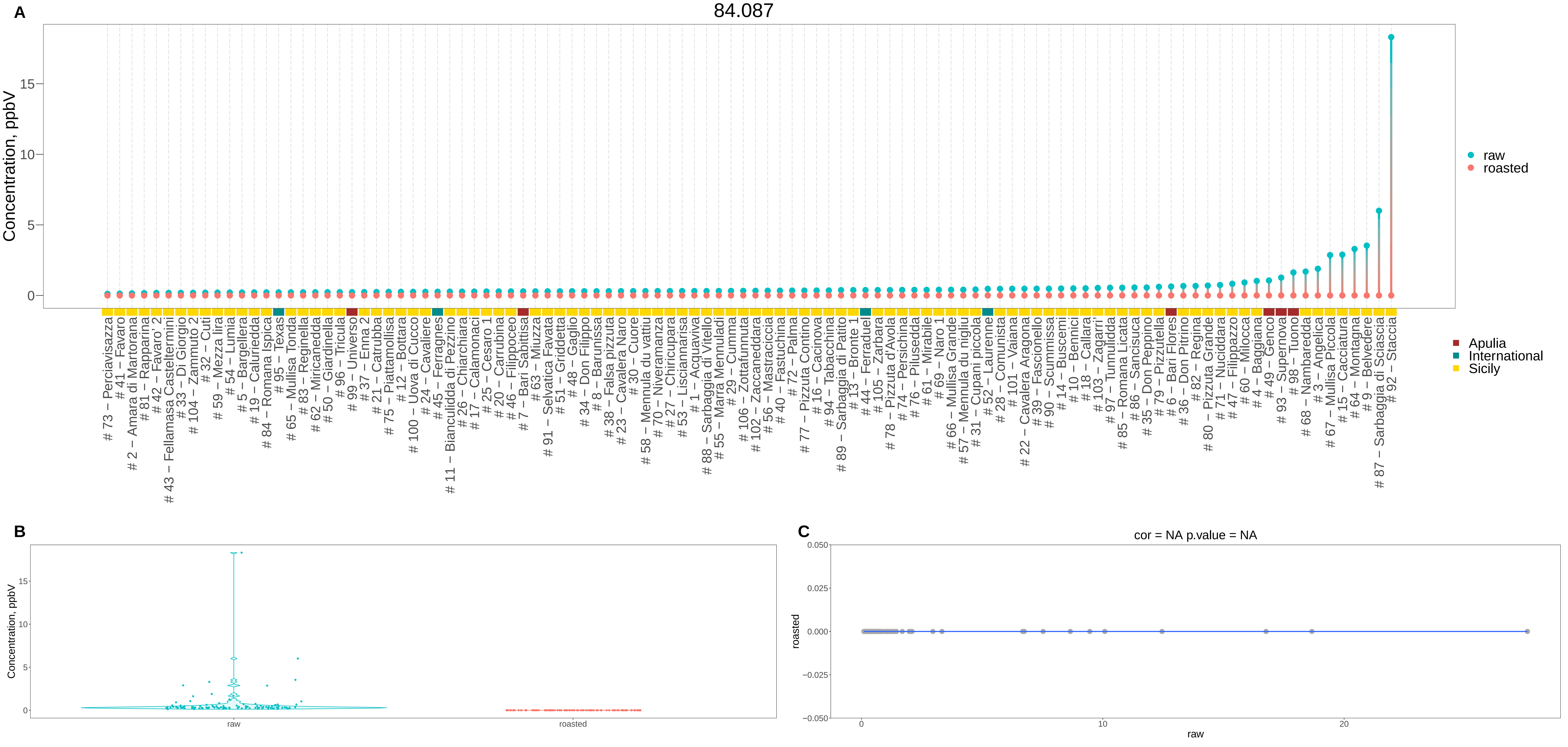

A
84.087
Concentration, ppbV
0
5
10
15
raw
roasted
Apulia
International
Sicily
73 – Perciavisazza
41 – Favaro
2 – Amara di Martorana
81 – Rapparino
42 – Favaro' 2
43 – Fellamasa Casteltermini
33 – Di Giorgio
104 – Zammuto 2
32 – Cuti
59 – Mezza lira
54 – Lumia
5 – Bargellera
19 – Caluriedda
84 – Romana Ispica
95 – Texas
65 – Mullisa Tonda
83 – Reginella
62 – Miricanedda
50 – Giardinella
96 – Tricula
99 – Universo
37 – Enna 2
21 – Catrubba
75 – Piattamollisa
12 – Bottara
100 – Uova di Cucco
24 – Cavaliere
45 – Ferragnes
11 – Bianculidda di Pezzino
26 – Chiarchiara
17 – Calamonaci
25 – Cesaro 1
20 – Carrubina
46 – Filippoceo
7 – Bari Sabittisa
63 – Miuzza
91 – Selvatica Favata
51 – Griddetta
48 – Gaglio
34 – Don Filippo
8 – Barunissa
38 – Falsa pizzuta
23 – Cavalera Naro
30 – Cuore
58 – Mennula du vattiu
70 – Niveramanza
27 – Chiricupara
53 – Lisciannarisa
1 – Acquaviva
88 – Sarbaggia di Vitello
55 – Marra Mennuladi
29 – Cumma
106 – Zottafunnuta
102 – Zaccaneddara
56 – Mastracicia
40 – Fastuchina
72 – Palma
77 – Pizzuta Contino
16 – Cacinova
94 – Tabacchina
89 – Sarbaggia di Patito
13 – Bronte 1
44 – Ferraduel
105 – Zarbara
78 – Pizzuta d'Avola
74 – Persichina
76 – Pilusedda
61 – Mirabile
69 – Naro 1
66 – Mullisa Grande
57 – Mennula du nigliu
31 – Cupani piccola
52 – Laurenne
28 – Comunista
101 – Vaiana
22 – Cavalera Aragona
39 – Fascionello
90 – Scummissa
14 – Buscemi
10 – Bennici
18 – Callara
103 – Zagarri'
97 – Tunnulidda
85 – Romana Licata
86 – Sancisuca
35 – Don Peppino
79 – Pizzutella
6 – Bari Flores
36 – Don Pitrino
82 – Regina
80 – Pizzuta Grande
71 – Nuciddara
47 – Filippazzo
60 – Milocca
4 – Baggiana
49 – Genco
93 – Supernova
98 – Tuono
68 – Nambaredda
3 – Angelica
67 – Mullisa Piccola
15 – Cacciatura
64 – Montagna
9 – Belvedere
87 – Sarbaggia di Sciascia
92 – Staccia
B
Concentration, ppbV
0
5
10
15
raw
roasted
C
cor = NA p.value = NA
roasted
0.050
0.025
0.000
−0.025
−0.050
0
10
20
raw

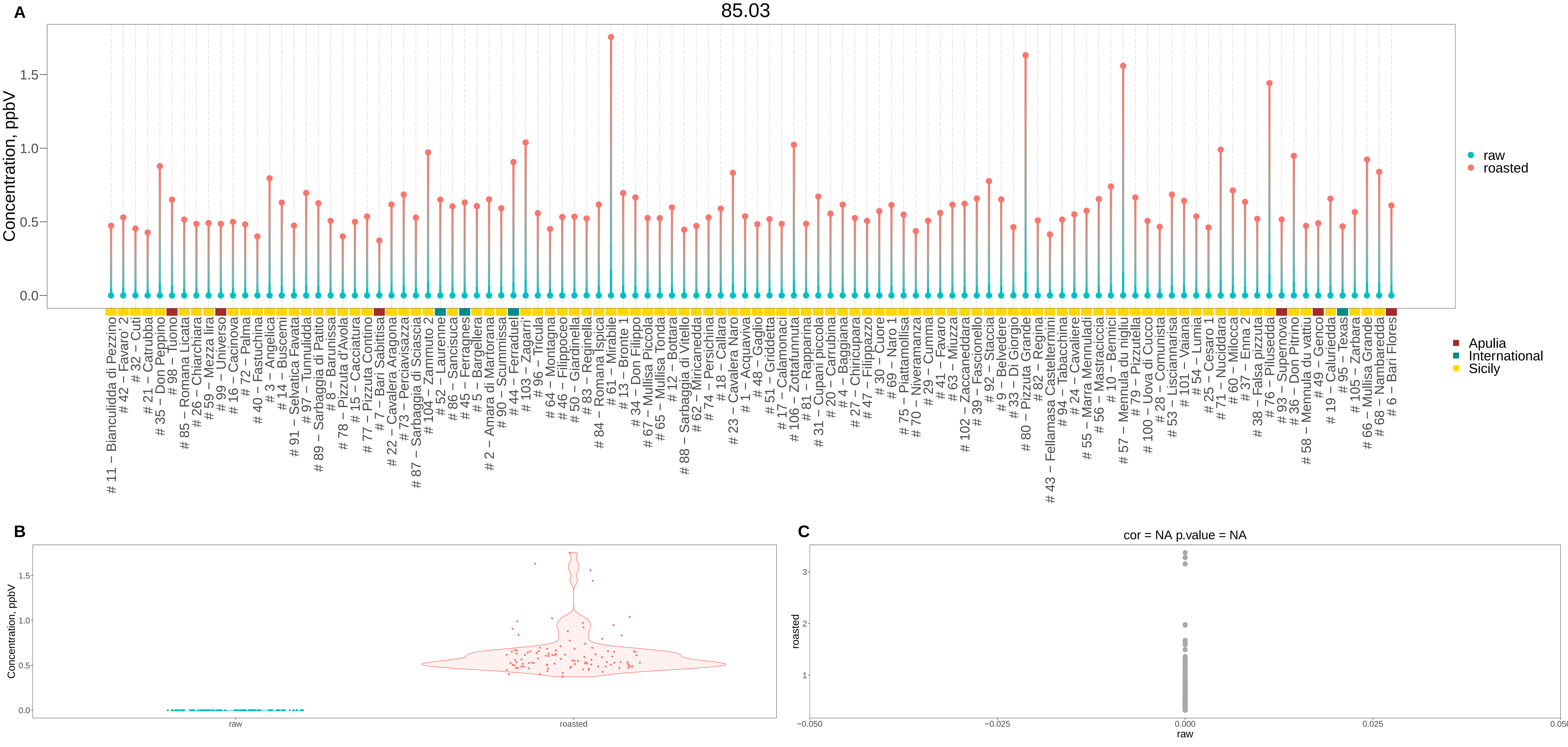
A
85.03
Concentration, ppbV
raw
roasted
Apulia
International
Sicily
B
Concentration, ppbV
raw
roasted
C
cor = NA p.value = NA
roasted
raw

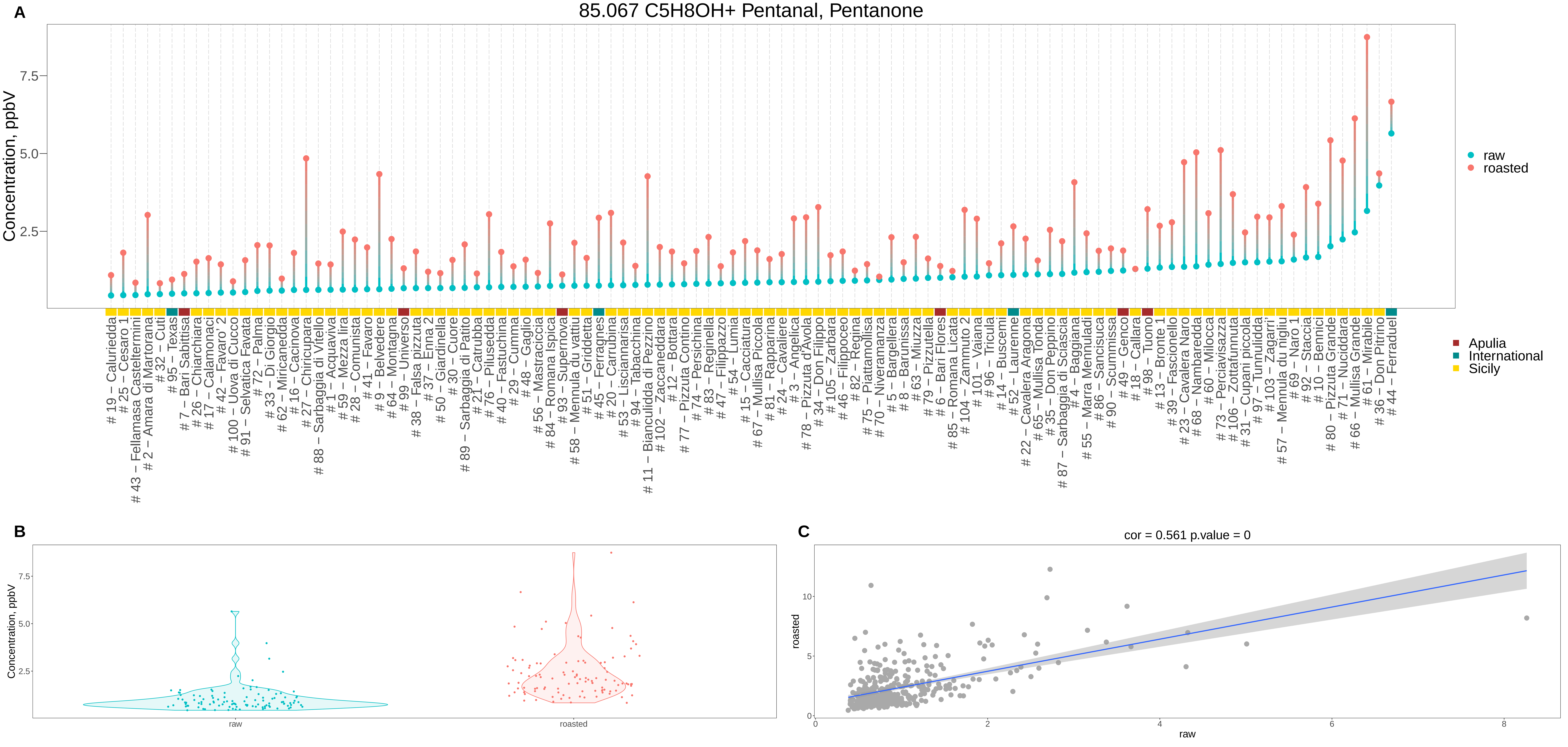
A
85.067 C5H8OH+ Pentanal, Pentanone
Concentration, ppbV
2.5
5.0
7.5
raw
roasted
Apulia
International
Sicily
19 – Caluriedda
25 – Cesaro 1
43 – Fellamasa Casteltermini
2 – Amara di Martorana
32 – Cuti
95 – Texas
7 – Bari Sabittisa
26 – Chiarchiara
17 – Calamonaci
42 – Favaro' 2
100 – Uova di Cucco
91 – Selvatica Favata
72 – Palma
33 – Di Giorgio
62 – Miricanedda
16 – Cacinova
27 – Chiricupara
88 – Sarbaggia di Vitello
1 – Acquaviva
59 – Mezza lira
28 – Comunista
41 – Favaro
9 – Belvedere
64 – Montagna
99 – Universo
38 – Falsa pizzuta
37 – Enna 2
50 – Giardinella
30 – Cuore
89 – Sarbaggia di Patito
21 – Catrubba
76 – Pilusedda
40 – Fastuchina
29 – Cumma
48 – Gaglio
56 – Mastraciccia
84 – Romana Ispica
93 – Supernova
58 – Mennula du vattiu
51 – Griddetta
45 – Ferragnes
20 – Carrubina
53 – Lisciannarisa
94 – Tabacchina
11 – Bianculidda di Pezzino
102 – Zaccaneddara
12 – Bottara
77 – Pizzuta Contino
74 – Persichina
83 – Reginella
47 – Filipazzo
54 – Lumia
15 – Cacciatura
67 – Mullisa Piccola
81 – Rapparina
24 – Cavaliere
3 – Angelica
78 – Pizzuta d'Avola
34 – Don Filippo
105 – Zarbara
46 – Filippoceo
82 – Regina
75 – Piattamollisa
70 – Niveramanza
5 – Bargellera
8 – Barunissa
63 – Miuzza
79 – Pizzutella
6 – Bari Flores
85 – Romana Licata
104 – Zammuto 2
101 – Vaiana
96 – Tricula
14 – Buscemi
52 – Laurenne
22 – Cavalera Aragona
65 – Mullisa Tonda
35 – Don Peppino
87 – Sarbaggia di Sciascia
4 – Baggiana
55 – Marra Mennuladi
86 – Sancisuca
90 – Scummissa
49 – Genco
18 – Callara
98 – Tuono
13 – Bronte 1
39 – Fascionello
23 – Cavalera Naro
68 – Nambaredda
60 – Milocca
73 – Perciavisazza
106 – Zottafunnuta
31 – Cupani piccola
97 – Tunnulidda
103 – Zagarri'
57 – Mennula du nigliu
69 – Naro 1
92 – Staccia
10 – Bennici
80 – Pizzuta Grande
71 – Nuciddara
66 – Mullisa Grande
61 – Mirabile
36 – Don Pitrino
44 – Ferraduel
B
Concentration, ppbV
2.5
5.0
7.5
raw
roasted
C
cor = 0.561 p.value = 0
roasted
0
5
10
raw
0
2
4
6
8

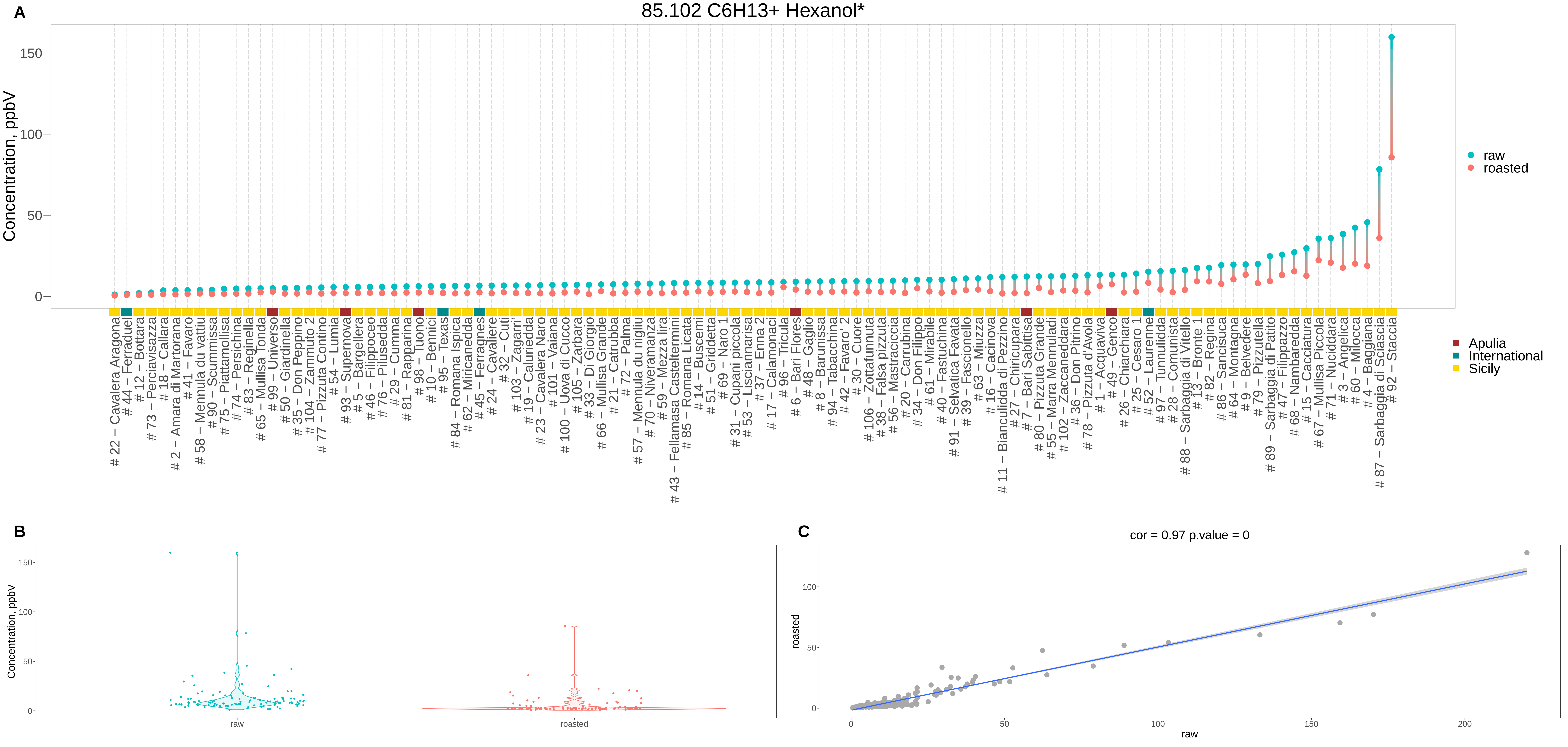

A
85.102 C6H13+ Hexanol*
Concentration, ppbV
0
50
100
150
raw
roasted
Apulia
International
Sicily
22 – Cavalera Aragona
44 – Ferraduel
12 – Bottara
73 – Perciavisazza
18 – Callara
2 – Amara di Martorana
41 – Favaro
58 – Mennula du vattiu
90 – Scummissa
75 – Piattamollisa
74 – Persichina
83 – Reginella
65 – Mullisa Tonda
99 – Universo
50 – Giardinella
35 – Don Peppino
104 – Zammuto 2
77 – Pizzuta Contino
54 – Lumia
93 – Supernova
5 – Bargellero
46 – Filippoceo
76 – Pilusedda
29 – Cumma
81 – Rapparina
98 – Tuono
10 – Bennici
95 – Texas
84 – Romana Ispica
62 – Miricanedda
45 – Ferragnes
24 – Cavaliere
32 – Cuti
103 – Zagarri'
19 – Caluriedda
23 – Cavalera Naro
101 – Vaiana
100 – Uova di Cucco
105 – Zarbara
33 – Di Giorgio
66 – Mullisa Grande
21 – Catrubba
72 – Palma
57 – Mennula du nigliu
70 – Niveramanza
59 – Mezza lira
43 – Fellamasa Casteltermini
85 – Romana Licata
14 – Buscemi
51 – Griddetta
69 – Naro 1
31 – Cupani piccola
53 – Lisciannarisa
37 – Enna 2
17 – Calamonaci
96 – Tricula
6 – Bari Flores
48 – Gaglio
8 – Barunissa
94 – Tabacchina
42 – Favaro' 2
30 – Cuore
106 – Zottafunnuta
38 – Falsa pizzuta
56 – Mastracicca
20 – Carrubina
34 – Don Filippo
61 – Mirabile
40 – Fastuchina
91 – Selvatica Favata
39 – Fascionello
63 – Miuzza
16 – Cacinova
11 – Biancuilidda di Pezzino
27 – Chiricupara
7 – Bari Sabittisa
80 – Pizzuta Grande
55 – Marra Mennuladi
102 – Zaccaneddara
36 – Don Pitrino
78 – Pizzuta d'Avola
1 – Acquaviva
49 – Genco
26 – Chiarchiara
25 – Cesaro 1
52 – Laurenne
97 – Tunnulidda
28 – Comunista
88 – Sarbaggia di Vitello
13 – Bronte 1
82 – Regina
86 – Sancisuca
64 – Montagna
9 – Belvedere
79 – Pizzutella
89 – Sarbaggia di Patito
47 – Filippazzo
68 – Nambaredda
15 – Cacciatura
67 – Mullisa Piccola
71 – Nuciddara
3 – Angelica
60 – Milocca
4 – Baggiana
87 – Sarbaggia di Sciascia
92 – Staccia
B
Concentration, ppbV
0
50
100
150
raw
roasted
C
cor = 0.97 p.value = 0
roasted
0
50
100
raw
0
50
100
150
200

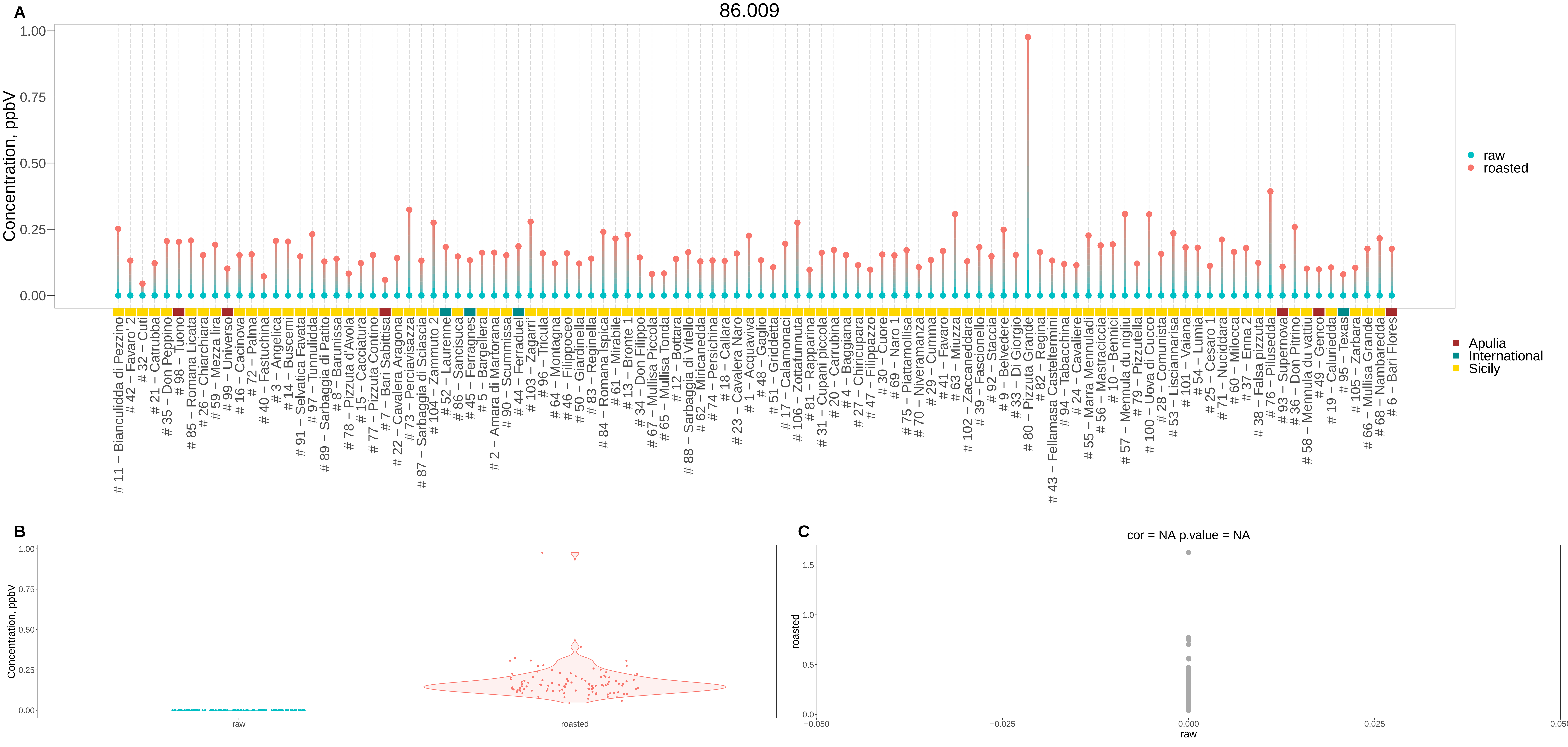
A
86.009
Concentration, ppbV
1.00
0.75
0.50
0.25
0.00
raw
roasted
Apulia
International
Sicily
11 – Bianculidda di Pezzino
42 – Favaro' 2
32 – Cuti
21 – Catrubba
35 – Don Peppino
98 – Tuono
85 – Romana Licata
26 – Chiarchiara
59 – Mezza lira
99 – Universo
16 – Cacinova
72 – Palma
40 – Fastuchina
3 – Angelica
14 – Buscemi
91 – Selvatica Favata
97 – Tunnulidda
89 – Sarbaggia di Patito
8 – Barunissa
78 – Pizzuta d'Avola
15 – Cacciatura
77 – Pizzuta Contino
7 – Bari Sabitissa
22 – Cavalera Aragona
73 – Perciavisazza
87 – Sarbaggia di Sciascia
104 – Zammuto 2
52 – Laurenne
86 – Sancisuca
45 – Ferragnes
5 – Bargellera
2 – Amara di Martorana
90 – Scummissa
44 – Ferraduel
103 – Zagarri'
96 – Tricula
64 – Montagna
46 – Filippoceo
50 – Giardinella
83 – Reginella
84 – Romana Ispica
61 – Mirabile
13 – Bronte 1
34 – Don Filippo
67 – Mullisa Piccola
65 – Mullisa Tonda
12 – Bottara
88 – Sarbaggia di Vitello
62 – Miricanedda
74 – Persichina
18 – Callara
23 – Cavalera Naro
1 – Acquaviva
48 – Gaglio
51 – Griddetta
17 – Calamonaci
106 – Zottafunnuta
81 – Rapparina
31 – Cupani piccola
20 – Carrubina
4 – Baggiana
27 – Chiricupara
47 – Filippazzo
30 – Cuore
69 – Naro 1
75 – Piattamollisa
70 – Niveramanza
29 – Cumma
41 – Favaro
63 – Miuzza
102 – Zaccaneddara
39 – Fascionello
92 – Staccia
9 – Belvedere
33 – Di Giorgio
80 – Pizzuta Grande
82 – Regina
43 – Fellamasa Casteltermini
94 – Tabacchina
24 – Cavaliere
55 – Marra Mennuladi
56 – Mastraciccia
10 – Bennici
57 – Mennula du nigliu
79 – Pizzutella
100 – Uova di Cucco
28 – Comunista
53 – Liscianarisa
101 – Vaiana
54 – Lumia
25 – Cesaro 1
71 – Nuciddara
60 – Milocca
37 – Enna 2
38 – Falsa pizzuta
76 – Pilusedda
93 – Supernova
36 – Don Pitrino
58 – Mennula du vattiu
49 – Genco
19 – Caluriedda
95 – Texas
105 – Zarbara
66 – Mullisa Grande
68 – Nambaredda
6 – Bari Flores
B
Concentration, ppbV
1.00
0.75
0.50
0.25
0.00
raw
roasted
C
cor = NA p.value = NA
roasted
1.5
1.0
0.5
0.0
−0.050
−0.025
0.000
0.025
0.050
raw

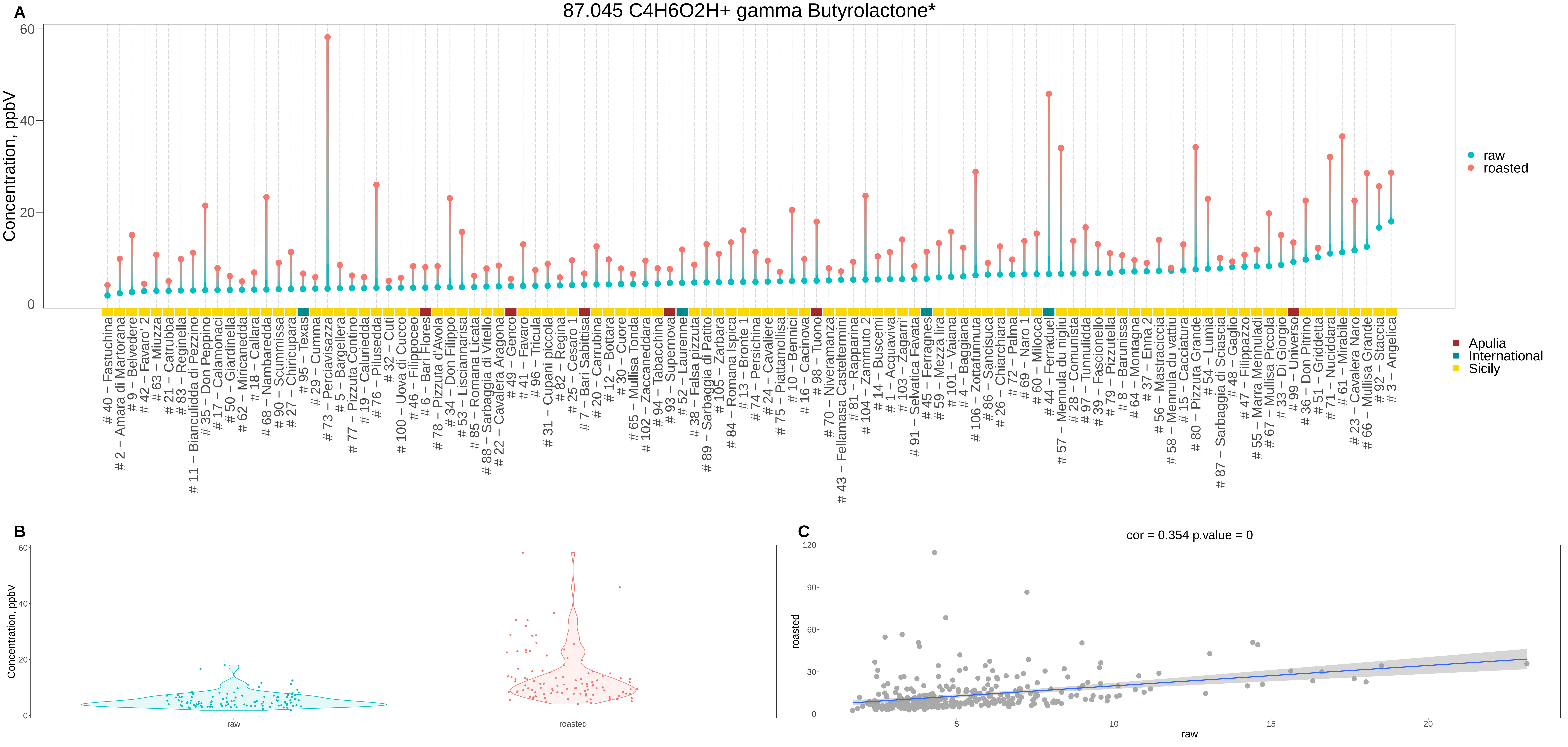
A
87.045 C4H6O2H+ gamma Butyrolactone*
Concentration, ppbV
0
20
40
60
raw
roasted
Apulia
International
Sicily
40 – Fastuchina
2 – Amara di Martorana
9 – Belvedere
42 – Favaro' 2
63 – Miuzza
21 – Catrubba
83 – Reginella
11 – Biancuilidda di Pezzino
35 – Don Peppino
17 – Calamonaci
50 – Giardinella
62 – Miricanedda
18 – Callara
68 – Nambaredda
90 – Scummissa
27 – Chiricupara
95 – Texas
29 – Cumma
73 – Perciavisazza
5 – Bargellera
77 – Pizzuta Contino
19 – Caluriedda
76 – Pilusedda
32 – Cuti
100 – Uova di Cucco
46 – Filippoceo
6 – Bari Flores
78 – Pizzuta d'Avola
34 – Don Filippo
53 – Liscianarisa
85 – Romana Licata
88 – Sarbaggia di Vitello
22 – Cavalera Aragona
49 – Genco
41 – Favaro
96 – Tricula
31 – Cupani piccola
82 – Regina
25 – Cesaro 1
7 – Bari Sabittisa
20 – Carrubina
12 – Bottara
30 – Cuore
65 – Mullisa Tonda
102 – Zaccaneddara
94 – Tabacchina
93 – Supernova
52 – Laurenne
38 – Falsa pizzuta
89 – Sarbaggia di Patito
105 – Zarbara
84 – Romana Ispica
13 – Bronte 1
74 – Persichina
24 – Cavaliere
75 – Piattamollisa
10 – Bennici
16 – Cacinova
98 – Tuono
70 – Niveramanza
43 – Fellamasa Casteltermini
81 – Rapparina
104 – Zammuto 2
14 – Buscemi
1 – Acquaviva
103 – Zagarri'
91 – Selvatica Favata
45 – Ferragnes
59 – Mezza lira
101 – Vaiana
4 – Baggiana
106 – Zottafunnuta
86 – Sancisuca
26 – Chiarchiara
72 – Palma
69 – Naro 1
60 – Milocca
44 – Ferraduel
57 – Mennula du nigliu
28 – Comunista
97 – Tunnulidda
39 – Fascionello
79 – Pizzutella
8 – Barunissa
64 – Montagna
37 – Enna 2
56 – Mastraciccia
58 – Mennula du vattiu
15 – Cacciatura
80 – Pizzuta Grande
54 – Lumia
87 – Sarbaggia di Sciascia
48 – Gaglio
47 – Filippazzo
55 – Marra Mennuladi
67 – Mullisa Piccola
33 – Di Giorgio
99 – Universo
36 – Don Pitrino
51 – Griddetta
71 – Nuciddara
61 – Mirabile
23 – Cavalera Naro
66 – Mullisa Grande
92 – Staccia
3 – Angelica
B
Concentration, ppbV
0
20
40
60
raw
roasted
C
cor = 0.354 p.value = 0
roasted
0
30
60
90
120
raw
5
10
15
20

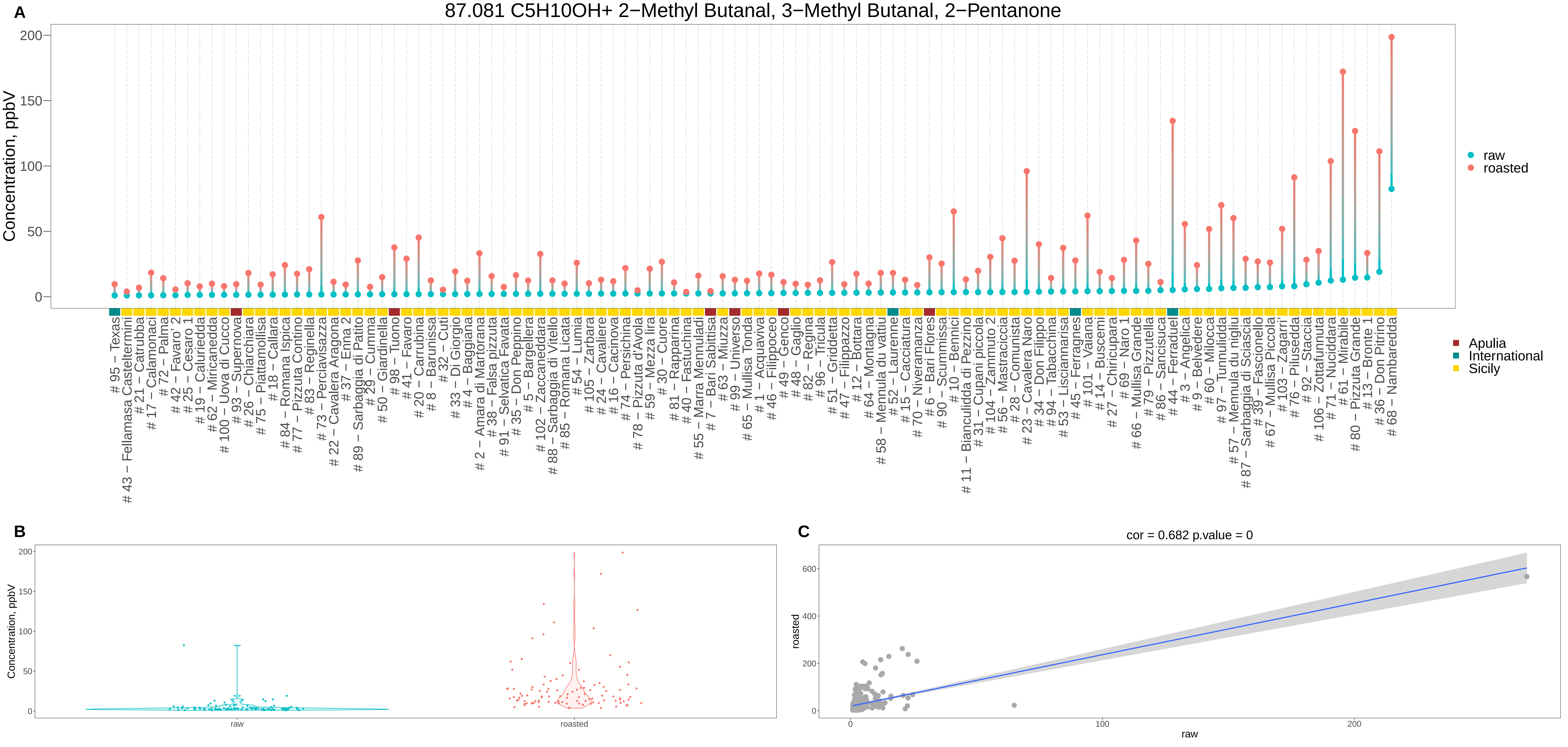
A
87.081 C5H10OH+ 2−Methyl Butanal, 3−Methyl Butanal, 2−Pentanone
Concentration, ppbV
raw
roasted
Apulia
International
Sicily
B
raw
roasted
C
cor = 0.682 p.value = 0

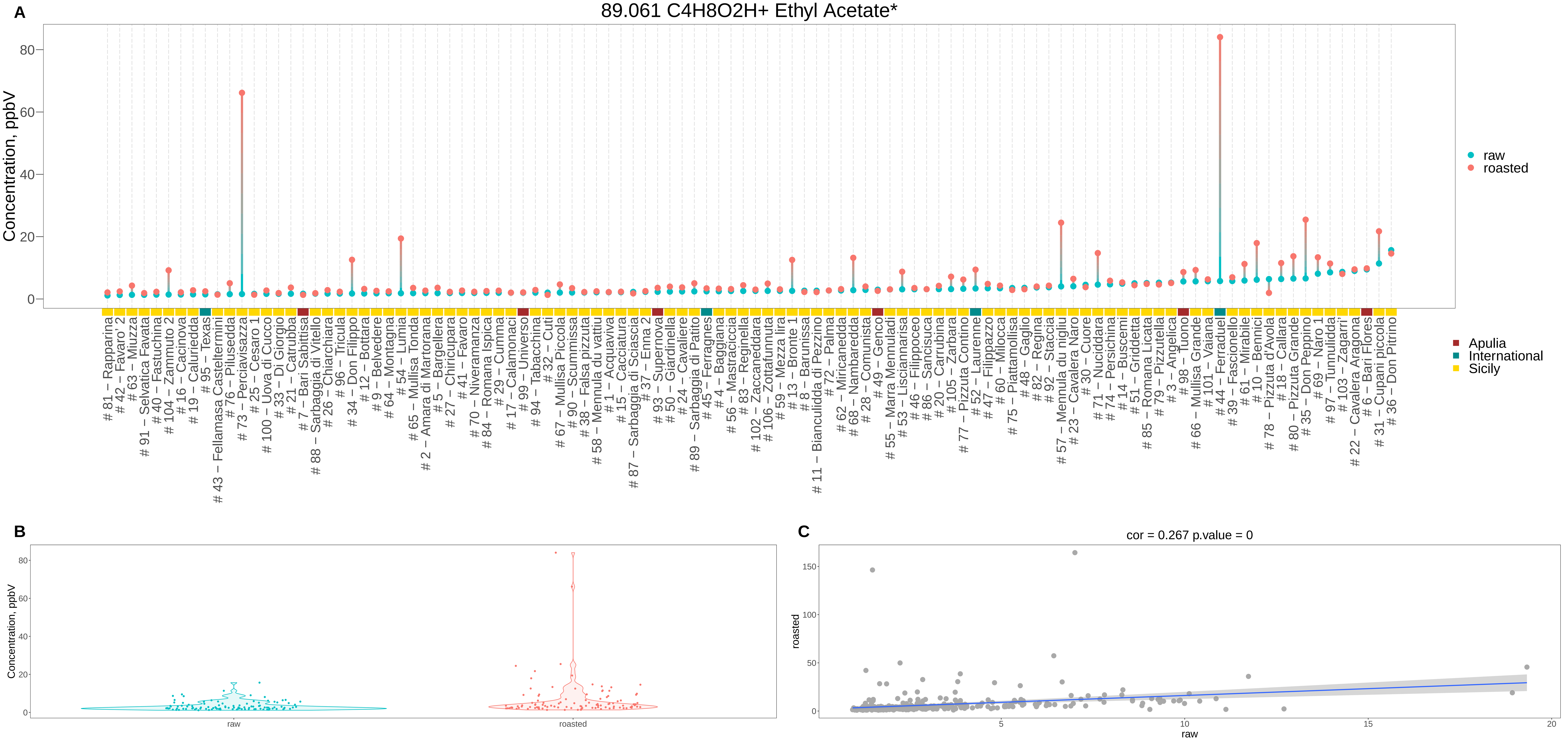
A
89.061 C4H8O2H+ Ethyl Acetate*
Concentration, ppbV
raw
roasted
Apulia
International
Sicily
B
Concentration, ppbV
raw
roasted
C
cor = 0.267 p.value = 0
roasted
raw

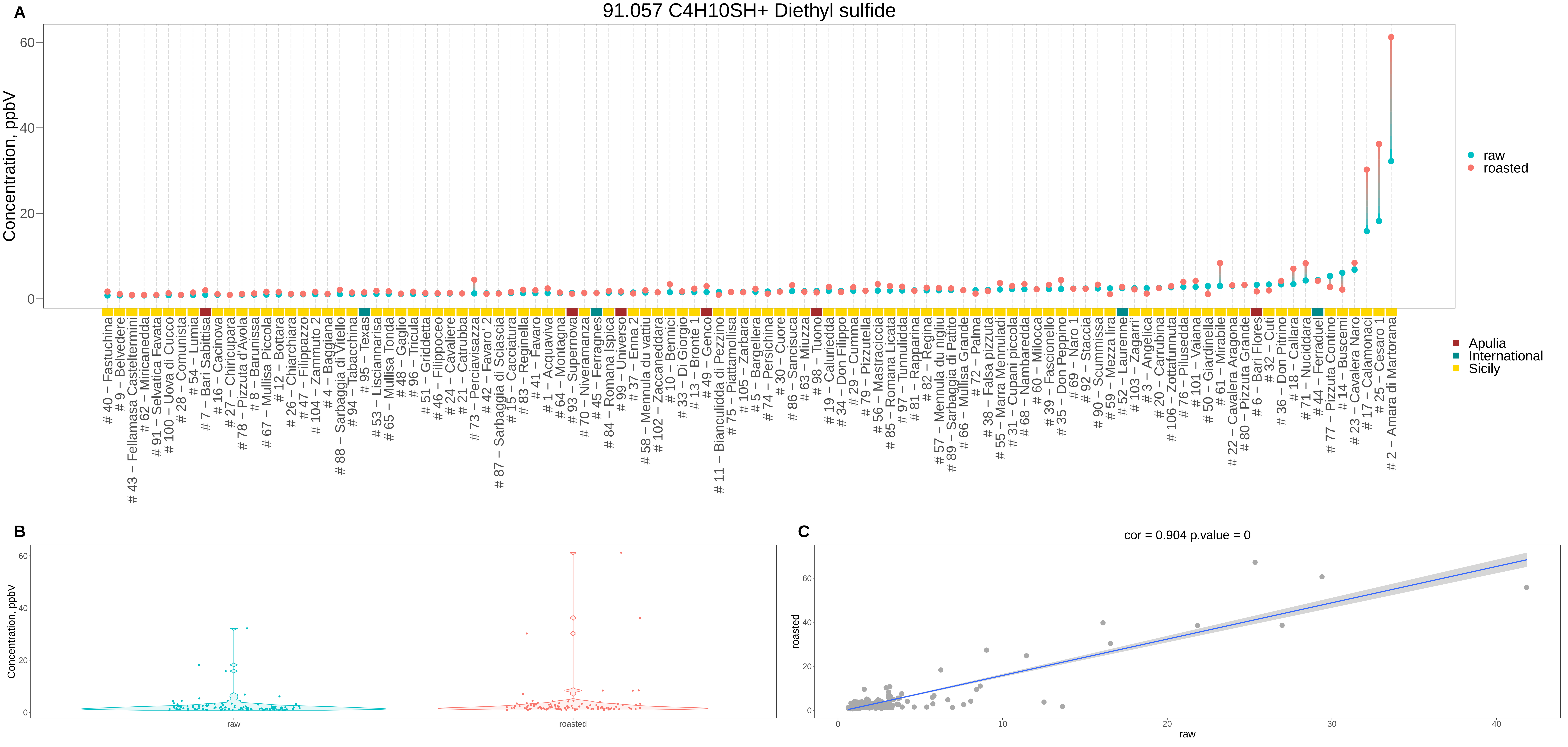
A
91.057 C4H10SH+ Diethyl sulfide
Concentration, ppbV
0
20
40
60
raw
roasted
Apulia
International
Sicily
40 – Fastuchina
9 – Belvedere
43 – Fellamasa Casteltermini
62 – Miricanedda
91 – Selvatica Favata
100 – Uova di Cucco
28 – Comunista
54 – Lumia
7 – Bari Sabittisa
16 – Cacinova
27 – Chiricupara
78 – Pizzuta d'Avola
8 – Barunissa
67 – Mullisa Piccola
12 – Bottara
26 – Chiarchiara
47 – Filippazzo
104 – Zammuto 2
4 – Baggiana
88 – Sarbaggia di Vitello
94 – Tabacchina
95 – Texas
53 – Lisciannarisa
65 – Mullisa Tonda
48 – Gaglio
96 – Tricula
51 – Griddetta
46 – Filippoceo
24 – Cavaliere
21 – Catrubba
73 – Perciavisazza
42 – Favaro' 2
87 – Sarbaggia di Sciascia
15 – Cacciatura
83 – Reginella
41 – Favaro
1 – Acquaviva
64 – Montagna
93 – Supernova
70 – Niveramanza
45 – Ferragnes
84 – Romana Ispica
99 – Universo
37 – Enna 2
58 – Mennula du vattiu
102 – Zaccaneddara
10 – Bennici
33 – Di Giorgio
13 – Bronte 1
49 – Genco
11 – Biancuiidda di Pezzino
75 – Piattamollisa
105 – Zarbara
5 – Bargellera
74 – Persichina
30 – Cuore
86 – Sancisuca
63 – Miuzza
98 – Tuono
19 – Caluriedda
34 – Don Filippo
29 – Cumma
79 – Pizzutella
56 – Mastraciccia
85 – Romana Licata
97 – Tumulidda
81 – Rapparina
82 – Regina
57 – Mennula du nigliu
89 – Sarbaggia di Patito
66 – Mullisa Grande
72 – Palma
38 – Falsa pizzuta
55 – Marra Mennuladi
31 – Cupani piccola
68 – Nambaredda
60 – Milocca
39 – Fascionello
35 – Don Peppino
69 – Naro 1
92 – Staccia
90 – Scummissa
59 – Mezza lira
52 – Laurenne
103 – Zagarri'
3 – Angelica
20 – Carrubina
106 – Zottafunnuta
76 – Pilusedda
101 – Vaiana
50 – Giardinella
61 – Mirabile
22 – Cavalera Aragona
80 – Pizzuta Grande
6 – Bari Flores
32 – Cuti
36 – Don Pitrino
18 – Callara
71 – Nuciddara
44 – Ferraduel
77 – Pizzuta Contino
14 – Buscemi
23 – Cavalera Naro
17 – Calamonaci
25 – Cesaro 1
2 – Amara di Martorana
B
Concentration, ppbV
0
20
40
60
raw
roasted
C
cor = 0.904 p.value = 0
roasted
0
20
40
60
raw
0
10
20
30
40

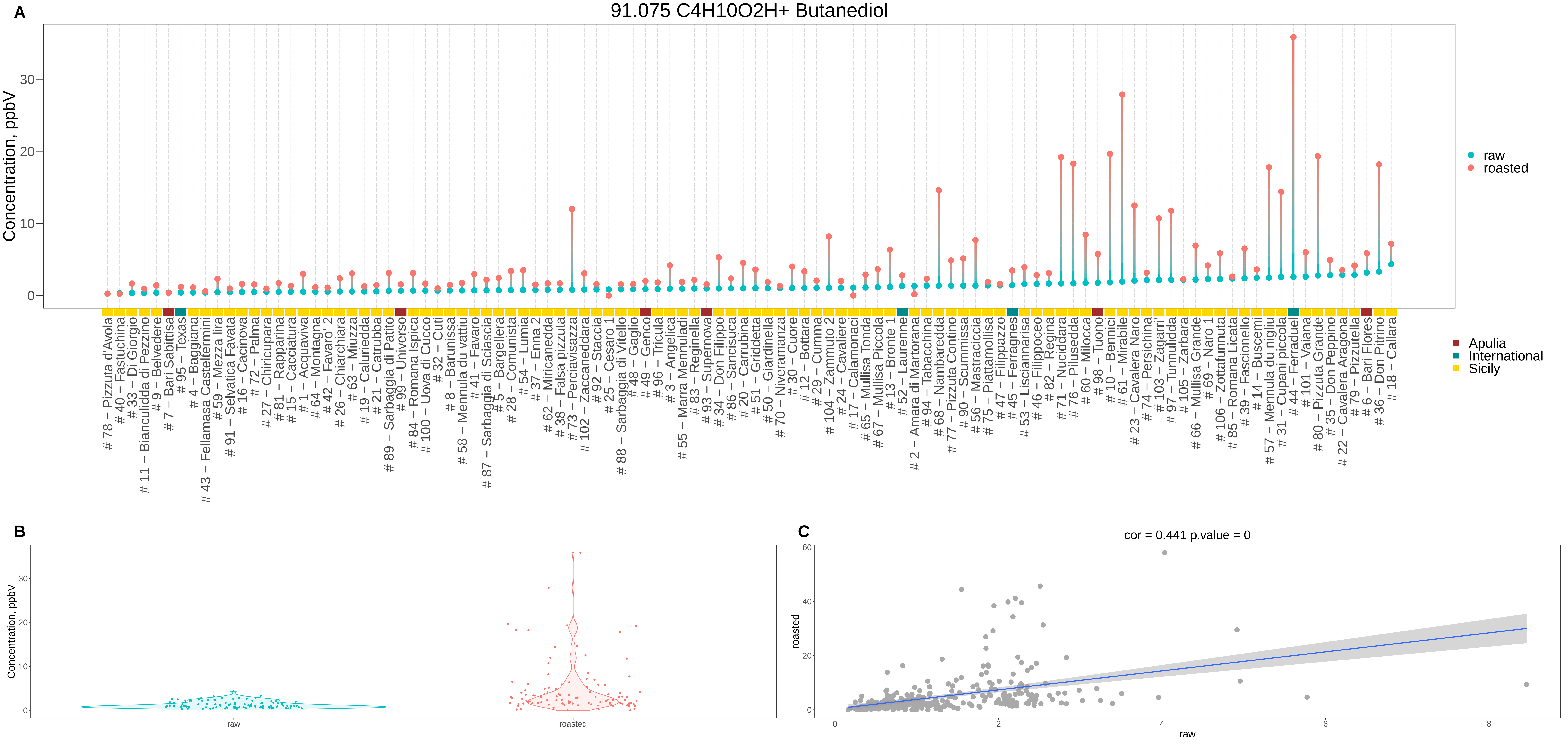
A
91.075 C4H10O2H+ Butanediol
Concentration, ppbV
0
10
20
30
raw
roasted
Apulia
International
Sicily
78 – Pizzuta d'Avola
40 – Fastuchina
33 – Di Giorgio
11 – Bianculidda di Pezzino
9 – Belvedere
7 – Bari Sabitisa
95 – Texas
4 – Baggiana
43 – Fellamasa Casteltermini
59 – Mezza lira
91 – Selvatica Favata
16 – Cacinova
72 – Palma
27 – Chiricupara
81 – Rapparina
15 – Cacciatura
1 – Acquaviva
64 – Montagna
42 – Favaro' 2
26 – Chiarchiara
63 – Miuzza
19 – Caluriedda
21 – Catrubba
89 – Sarbaggia di Patito
99 – Universo
84 – Romana Ispica
100 – Uova di Cucco
32 – Cuti
8 – Barunissa
58 – Mennula du vattiu
41 – Favaro
87 – Sarbaggia di Sciascia
5 – Bargellera
28 – Comunista
54 – Lumia
37 – Enna 2
62 – Miricanedda
38 – Falsa pizzuta
73 – Perciavisazza
102 – Zaccaneddara
92 – Staccio
25 – Cesaro 1
88 – Sarbaggia di Vitello
48 – Gaglio
49 – Genco
96 – Tricula
3 – Angelica
55 – Marra Mennuladi
83 – Reginella
93 – Supernova
34 – Don Filippo
86 – Sancisuca
20 – Carrubina
51 – Griddetta
50 – Giardinella
70 – Niveramanza
30 – Cuore
12 – Bottara
29 – Cumma
104 – Zammuto 2
24 – Cavaliere
17 – Calamonaci
65 – Mullisa Tonda
67 – Mullisa Piccola
13 – Bronte 1
52 – Laurenne
2 – Amara di Martorana
94 – Tabacchina
68 – Nambaredda
77 – Pizzuta Contino
90 – Scummissa
56 – Mastraciccia
75 – Piattamollisa
47 – Filippazzo
45 – Ferragnes
53 – Lisciannarisa
46 – Filippoceo
82 – Regina
71 – Nuciddara
76 – Pilusedda
60 – Millocca
98 – Tuono
10 – Bennici
61 – Mirabile
23 – Cavalera Naro
74 – Persichina
103 – Zagarri'
97 – Tunnulidda
105 – Zarbara
66 – Mullisa Grande
69 – Naro 1
106 – Zottafunnuta
85 – Romana Licata
39 – Fascionello
14 – Buscemi
57 – Mennula du nigliu
31 – Cupani piccola
44 – Ferraduel
101 – Vaiana
80 – Pizzuta Grande
35 – Don Peppino
22 – Cavalera Aragona
79 – Pizzutella
6 – Bari Flores
36 – Don Pitrino
18 – Callara
B
Concentration, ppbV
0
10
20
30
raw
roasted
C
cor = 0.441 p.value = 0
roasted
0
20
40
60
raw
0
2
4
6
8

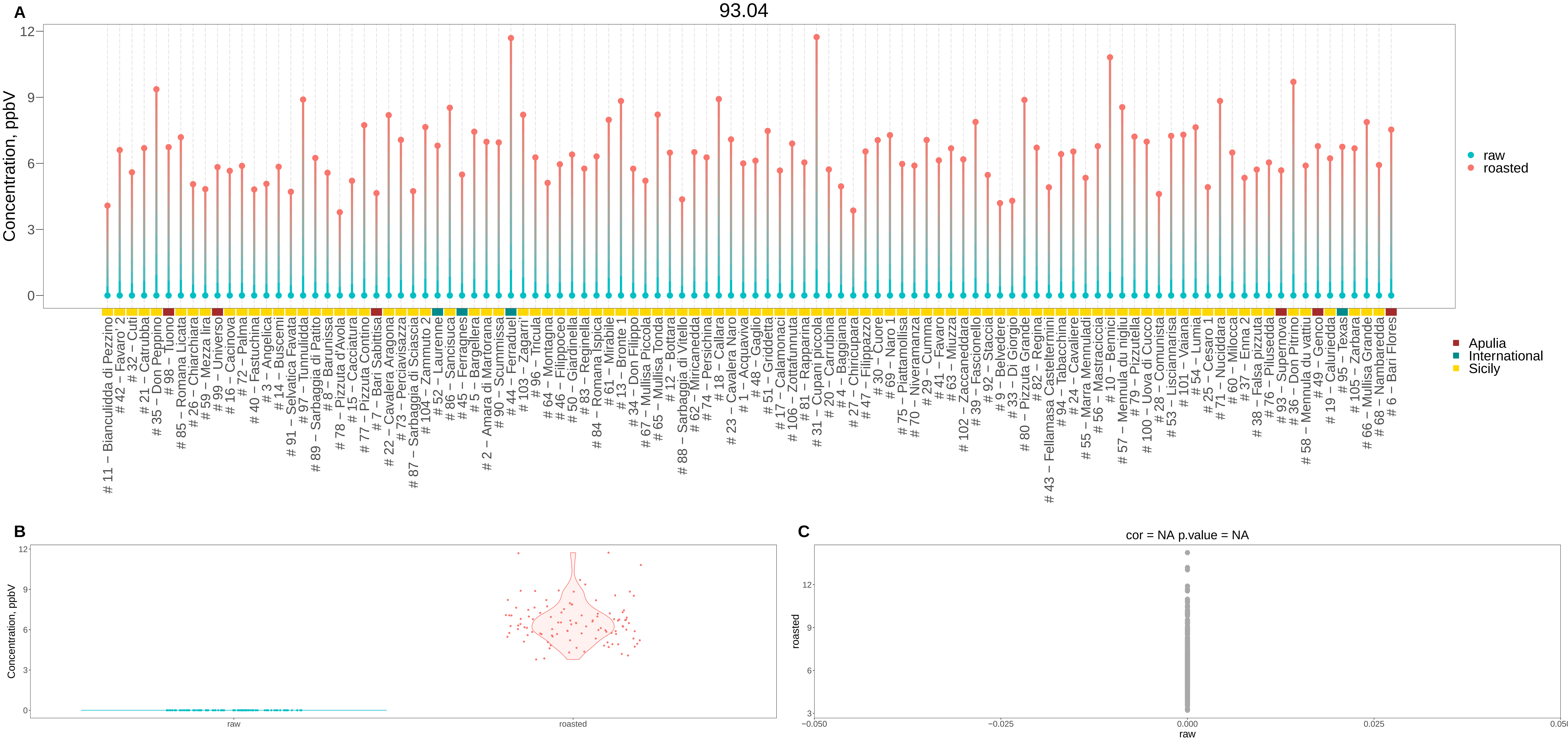
A
93.04
Concentration, ppbV
0
3
6
9
12
raw
roasted
Apulia
International
Sicily
11 – Bianculidda di Pezzino
42 – Favaro' 2
32 – Cuti
21 – Catrubba
35 – Don Peppino
98 – Tuono
85 – Romana Licata
26 – Chiarchiara
59 – Mezza lira
99 – Universo
16 – Cacinova
72 – Palma
40 – Fastuchina
3 – Angelica
14 – Buscemi
91 – Selvatica Favata
97 – Tunnulidda
89 – Sarbaggia di Patito
8 – Barunissa
78 – Pizzuta d'Avola
15 – Cacciatura
77 – Pizzuta Contino
7 – Bari Sabitissa
22 – Cavalera Aragona
73 – Perciavisazza
87 – Sarbaggia di Sciascia
104 – Zammuto 2
52 – Laurenne
86 – Sancisuca
45 – Ferragnes
5 – Bargellera
2 – Amara di Martorana
90 – Scummissa
44 – Ferraduel
103 – Zagarri'
96 – Tricula
64 – Montagna
46 – Filippoceo
50 – Giardinella
83 – Reginella
84 – Romana Ispica
61 – Mirabile
13 – Bronte 1
34 – Don Filippo
67 – Mullisa Piccola
65 – Mullisa Tonda
12 – Bottara
88 – Sarbaggia di Vitello
62 – Miricanedda
74 – Persichina
18 – Callara
23 – Cavalera Naro
1 – Acquaviva
48 – Gaglio
51 – Griddetta
17 – Calamonaci
106 – Zottafunnuta
81 – Rapparina
31 – Cupani piccola
20 – Carrubina
4 – Baggiana
27 – Chiricupara
47 – Filippazzo
30 – Cuore
69 – Naro 1
75 – Piattamollisa
70 – Niveramanza
29 – Cumma
41 – Favaro
63 – Miuzza
102 – Zaccaneddara
39 – Fascionello
92 – Staccia
9 – Belvedere
33 – Di Giorgio
80 – Pizzuta Grande
82 – Regina
43 – Fellamasa Casteltermini
94 – Tabacchina
24 – Cavaliere
55 – Marra Mennuladi
56 – Mastraciccia
10 – Bennici
57 – Mennula du nigliu
79 – Pizzutella
100 – Uova di Cucco
28 – Comunista
53 – Lisciannarisa
101 – Vaiana
54 – Lumia
25 – Cesaro 1
71 – Nuciddara
60 – Milocca
37 – Enna 2
38 – Falsa pizzuta
76 – Pilusedda
93 – Supernova
36 – Don Pitrino
58 – Mennula du vattiu
49 – Genco
19 – Caluriedda
95 – Texas
105 – Zarbara
66 – Mullisa Grande
68 – Nambaredda
6 – Bari Flores
B
Concentration, ppbV
0
3
6
9
12
raw
roasted
C
cor = NA p.value = NA
roasted
3
6
9
12
−0.050
−0.025
0.000
0.025
0.050
raw

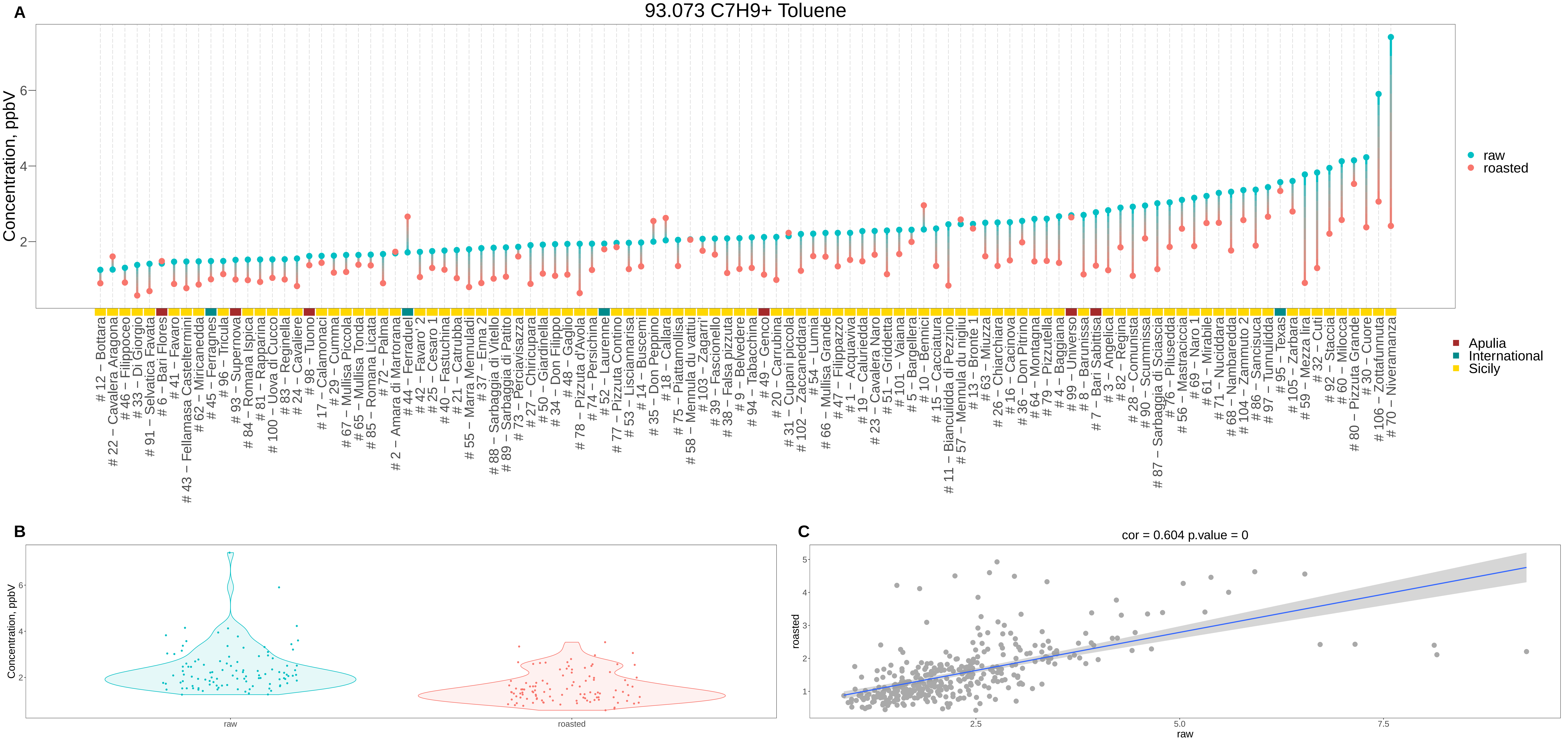
A
93.073 C7H9+ Toluene
Concentration, ppbV
2
4
6
raw
roasted
Apulia
International
Sicily
12 – Bottara
22 – Cavalera Aragona
46 – Filippoceo
33 – Di Giorgio
91 – Selvatica Favata
6 – Bari Flores
41 – Favaro
43 – Fellamasa Casteltermini
62 – Miricanedda
45 – Ferragnes
96 – Tricula
93 – Supernova
84 – Romana Ispica
81 – Rapparina
100 – Uova di Cucco
83 – Reginella
24 – Cavaliere
98 – Tuono
17 – Calamonaci
29 – Cumma
67 – Mullisa Piccola
65 – Mullisa Tonda
85 – Romana Licata
72 – Palma
2 – Amara di Martorana
44 – Ferraduel
42 – Favaro' 2
25 – Cesaro 1
40 – Fastuchina
21 – Catrubba
55 – Marra Mennuladi
37 – Enna 2
88 – Sarbaggia di Vitello
89 – Sarbaggia di Patito
73 – Perciavisazza
27 – Chiricupara
50 – Giardinella
34 – Don Filippo
48 – Gaglio
78 – Pizzuta d'Avola
74 – Persichina
52 – Laurenne
77 – Pizzuta Contino
53 – Liscianarisa
14 – Buscemi
35 – Don Peppino
18 – Callara
75 – Piattamollisa
58 – Mennula du vattiu
103 – Zagarri'
39 – Fascionello
38 – Falsa pizzuta
9 – Belvedere
94 – Tabacchina
49 – Genco
20 – Carrubina
31 – Cupani piccola
102 – Zaccaneddara
54 – Lumia
66 – Mullisa Grande
47 – Filippazzo
1 – Acquaviva
19 – Caluriedda
23 – Cavalera Naro
51 – Griddetta
101 – Vaiana
5 – Bargellera
10 – Bennici
15 – Cacciatura
11 – Bianculidda di Pezzino
57 – Mennula du nigliu
13 – Bronte 1
63 – Miuzza
26 – Chiarchiara
16 – Cacinova
36 – Don Pitrino
64 – Montagna
79 – Pizzutella
4 – Baggiana
99 – Universo
8 – Barunissa
7 – Bari Sabittisa
3 – Angelica
82 – Regina
28 – Comunista
90 – Scummissa
87 – Sarbaggia di Sciascia
76 – Pilusedda
56 – Mastraciccia
69 – Naro 1
61 – Mirabile
71 – Nuciddara
68 – Nambaredda
104 – Zammuto 2
86 – Sancisuca
97 – Tunnulidda
95 – Texas
105 – Zarbara
59 – Mezza lira
32 – Cuti
92 – Staccia
60 – Milocca
80 – Pizzuta Grande
30 – Cuore
106 – Zottafunnuta
70 – Niveramanza
B
Concentration, ppbV
2
4
6
raw
roasted
C
cor = 0.604 p.value = 0
roasted
1
2
3
4
5
2.5
5.0
7.5
raw

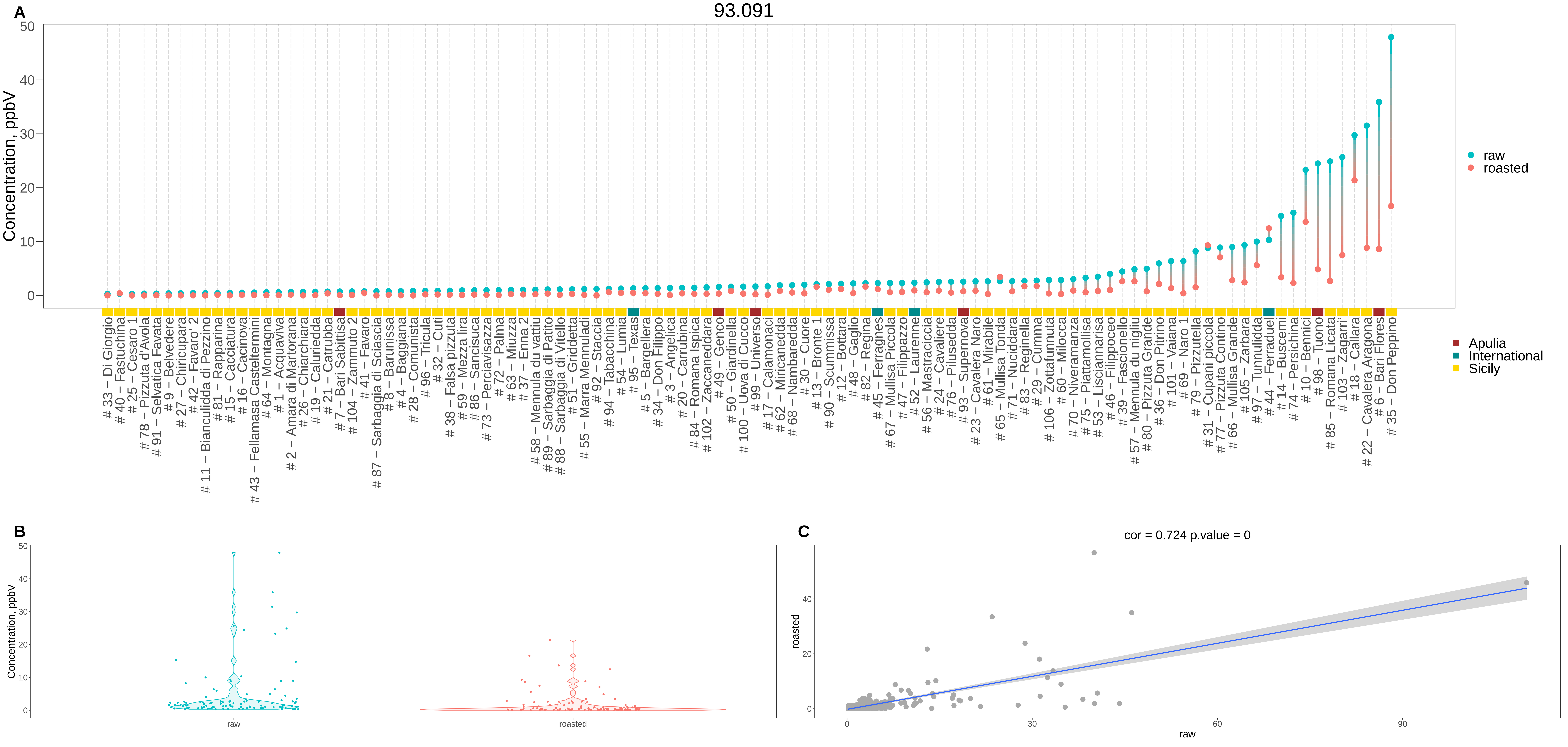

A
93.091
Concentration, ppbV
0
10
20
30
40
50
raw
roasted
Apulia
International
Sicily
33 – Di Giorgio
40 – Fastuchina
25 – Cesaro 1
78 – Pizzuta d'Avola
91 – Selvatica Favata
9 – Belvedere
27 – Chiricupara
42 – Favaro' 2
11 – Biancuidda di Pezzino
81 – Rapparina
15 – Cacciatura
16 – Cacinova
43 – Fellamasa Casteltermini
64 – Montagna
1 – Acquaviva
2 – Amara di Martorana
26 – Chiarchiara
19 – Caluriedda
21 – Catrubba
7 – Bari Sabittisa
104 – Zammuto 2
41 – Favaro
87 – Sarbaggia di Sciascia
8 – Barunissa
4 – Baggiana
28 – Comunista
96 – Tricula
32 – Cuti
38 – Falsa pizzuta
59 – Mezza lira
86 – Sancisuca
73 – Perciavisazza
72 – Palma
63 – Miuzza
37 – Enna 2
58 – Mennula du vattiu
89 – Sarbaggia di Patito
88 – Sarbaggia di Vitello
51 – Griddetta
55 – Marra Mennuladi
92 – Staccia
94 – Tabacchina
54 – Lumia
95 – Texas
5 – Bargellera
34 – Don Filippo
3 – Angelica
20 – Carrubina
84 – Romana Ispica
102 – Zaccaneddara
49 – Genco
50 – Giardinella
100 – Uova di Cucco
99 – Universo
17 – Calamonaci
62 – Miricanedda
68 – Nambaredda
30 – Cuore
13 – Bronte 1
90 – Scummissa
12 – Bottara
48 – Gaglio
82 – Regina
45 – Ferragnes
67 – Mullisa Piccola
47 – Filippazzo
52 – Laurenne
56 – Mastraciccia
24 – Cavaliere
76 – Pilusedda
93 – Supernova
23 – Cavalera Naro
61 – Mirabile
65 – Mullisa Tonda
71 – Nuciddara
83 – Reginella
29 – Cumma
106 – Zottafunnuta
60 – Milocca
70 – Niveramanza
75 – Piattamollisa
53 – Lisciannarisa
46 – Filippoceo
39 – Fascionello
57 – Mennula du nigliu
80 – Pizzuta Grande
36 – Don Pitrino
101 – Vaiana
69 – Naro 1
79 – Pizzutella
31 – Cupani piccola
77 – Pizzuta Contino
66 – Mullisa Grande
105 – Zarbara
97 – Tunnulidda
44 – Ferraduel
14 – Buscemi
74 – Persichina
10 – Bennici
98 – Tuono
85 – Romana Licata
103 – Zagarri'
18 – Callara
22 – Cavalera Aragona
6 – Bari Flores
35 – Don Peppino
B
Concentration, ppbV
raw
roasted
C
cor = 0.724 p.value = 0
roasted
raw
0
30
60
90

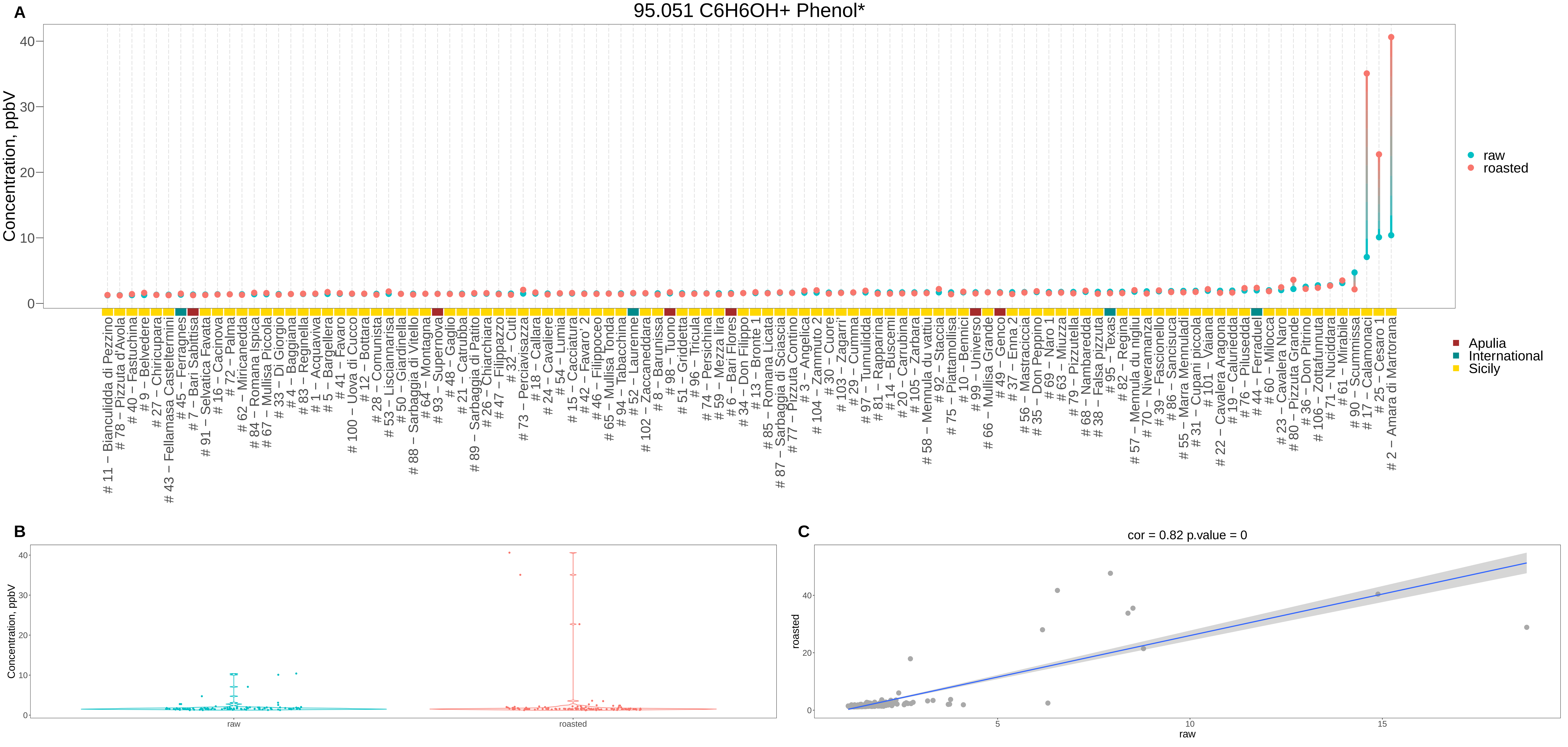
A
95.051 C6H6OH+ Phenol*
Concentration, ppbV
0
10
20
30
40
raw
roasted
Apulia
International
Sicily
11 – Bianculidda di Pezzino
78 – Pizzuta d'Avola
40 – Fastuchina
9 – Belvedere
27 – Chiricupara
43 – Fellamasa Casteltermini
45 – Ferragnes
7 – Bari Sabittisa
91 – Selvatica Favata
16 – Cacinova
72 – Palma
62 – Miricanedda
84 – Romana Ispica
67 – Mullisa Piccola
33 – Di Giorgio
4 – Baggiana
83 – Reginella
1 – Acquaviva
5 – Bargellera
41 – Favaro
100 – Uova di Cucco
12 – Bottara
28 – Comunista
53 – Liscianarisa
50 – Giardinella
88 – Sarbaggia di Vitello
64 – Montagna
93 – Supernova
48 – Gaglio
21 – Catrubba
89 – Sarbaggia di Patito
26 – Chiarchiara
47 – Filippazzo
32 – Cuti
73 – Perciavisazza
18 – Callara
24 – Cavaliere
54 – Lumia
15 – Cacciatura
42 – Favaro' 2
46 – Filippoceo
65 – Mullisa Tonda
94 – Tabacchina
52 – Laurenne
102 – Zaccaneddara
8 – Barunissa
98 – Tuono
51 – Griddetta
96 – Tricula
74 – Persichina
59 – Mezza lira
6 – Bari Flores
34 – Don Filippo
13 – Bronte 1
85 – Romana Licata
87 – Sarbaggia di Sciascia
77 – Pizzuta Contino
3 – Angelica
104 – Zammuto 2
30 – Cuore
103 – Zagari'
29 – Cumma
97 – Tunnulidda
81 – Rapparina
14 – Buscemi
20 – Carrubina
105 – Zarbara
58 – Mennula du vattiu
92 – Staccia
75 – Piattamollisa
10 – Bennici
99 – Universo
66 – Mullisa Grande
49 – Genco
37 – Enna 2
56 – Mastraciccia
35 – Don Peppino
69 – Naro 1
63 – Miuzza
79 – Pizzutella
68 – Nambaredda
38 – Falsa pizzuta
95 – Texas
82 – Regina
57 – Mennula du nigliu
70 – Niveramanza
39 – Fascionello
86 – Sancisuca
55 – Marra Mennuladi
31 – Cupani piccola
101 – Vaiana
22 – Cavalera Aragona
19 – Caluriedda
76 – Pilusedda
44 – Ferraduel
60 – Milocca
23 – Cavalera Naro
80 – Pizzuta Grande
36 – Don Pitrino
106 – Zottafunnuta
71 – Nucidara
61 – Mirabile
90 – Scummissa
17 – Calamonaci
25 – Cesaro 1
2 – Amara di Martorana
B
Concentration, ppbV
0
10
20
30
40
raw
roasted
C
cor = 0.82 p.value = 0
roasted
0
20
40
raw
5
10
15

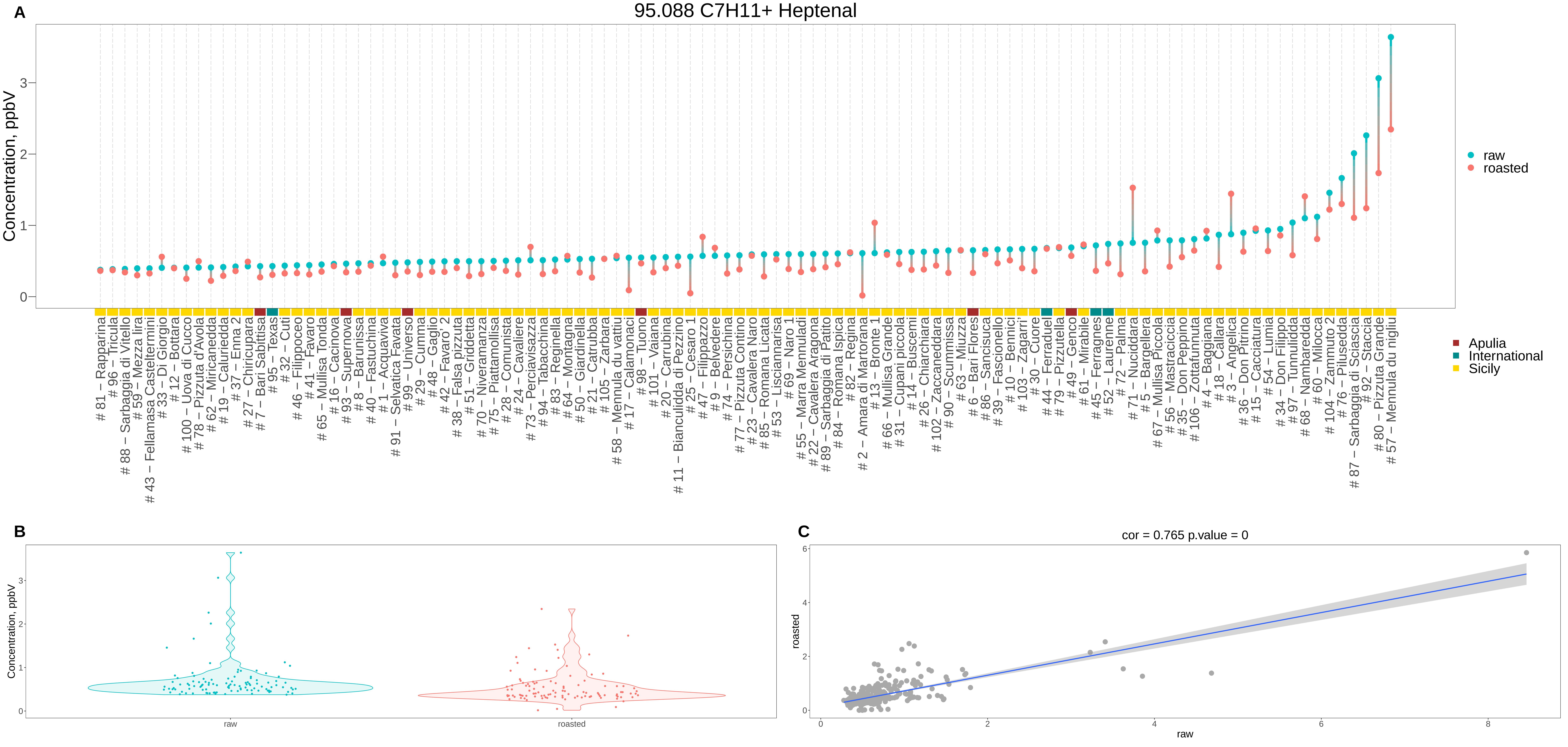

A
95.088 C7H11+ Heptenal
Concentration, ppbV
0
1
2
3
raw
roasted
Apulia
International
Sicily
81 – Rapparina
96 – Tricula
88 – Sarbaggia di Vitello
59 – Mezza lira
43 – Fellamasa Casteltermini
33 – Di Giorgio
12 – Bottara
100 – Uova di Cucco
78 – Pizzuta d'Avola
62 – Miricanedda
19 – Caluriedda
37 – Enna 2
27 – Chiricupara
7 – Bari Sabittisa
95 – Texas
32 – Cuti
46 – Filippoceo
41 – Favaro
65 – Mullisa Tonda
16 – Cacinova
93 – Supernova
8 – Barunissa
40 – Fastuchina
1 – Acquaviva
91 – Selvatica Favata
99 – Universo
29 – Cumma
48 – Gaglio
42 – Favaro' 2
38 – Falsa pizzuta
51 – Griddetta
70 – Niveramanza
75 – Piattamollisa
28 – Comunista
24 – Cavaliere
73 – Perciavisazza
94 – Tabacchina
83 – Reginella
64 – Montagna
50 – Giardinella
21 – Catrubba
105 – Zarbara
58 – Mennula du vattiu
17 – Calamonaci
98 – Tuono
101 – Vaiana
20 – Carrubina
11 – Bianculidda di Pezzino
25 – Cesaro 1
47 – Filippazzo
9 – Belvedere
74 – Persichina
77 – Pizzuta Contino
23 – Cavalera Naro
85 – Romana Licata
53 – Lisciannarisa
69 – Naro 1
55 – Marra Mennuladi
22 – Cavalera Aragona
89 – Sarbaggia di Patito
84 – Romana Ispica
82 – Regina
2 – Amara di Martorana
13 – Bronte 1
66 – Mullisa Grande
31 – Cupani piccola
14 – Busceni
26 – Chiarchiara
102 – Zaccaneddara
90 – Scummissa
63 – Miuzza
6 – Bari Flores
86 – Sancisuca
39 – Fascionello
10 – Bennici
103 – Zagarri'
30 – Cuore
44 – Ferraduel
79 – Pizzutella
49 – Genco
61 – Mirabile
45 – Ferragnes
52 – Laurenne
72 – Palma
71 – Nuciddara
5 – Bargellera
67 – Mullisa Piccola
56 – Mastraciccia
35 – Don Peppino
106 – Zottafunnuta
4 – Baggiana
18 – Callara
3 – Angelica
36 – Don Pitrino
15 – Cacciatura
54 – Lumia
34 – Don Filippo
97 – Tunnulidda
68 – Nambaredda
60 – Milocca
104 – Zammuto 2
76 – Pilusedda
87 – Sarbaggia di Sciascia
92 – Staccia
80 – Pizzuta Grande
57 – Mennula du nigliu
B
Concentration, ppbV
0
1
2
3
raw
roasted
C
cor = 0.765 p.value = 0
roasted
0
2
4
6
raw
0
2
4
6
8

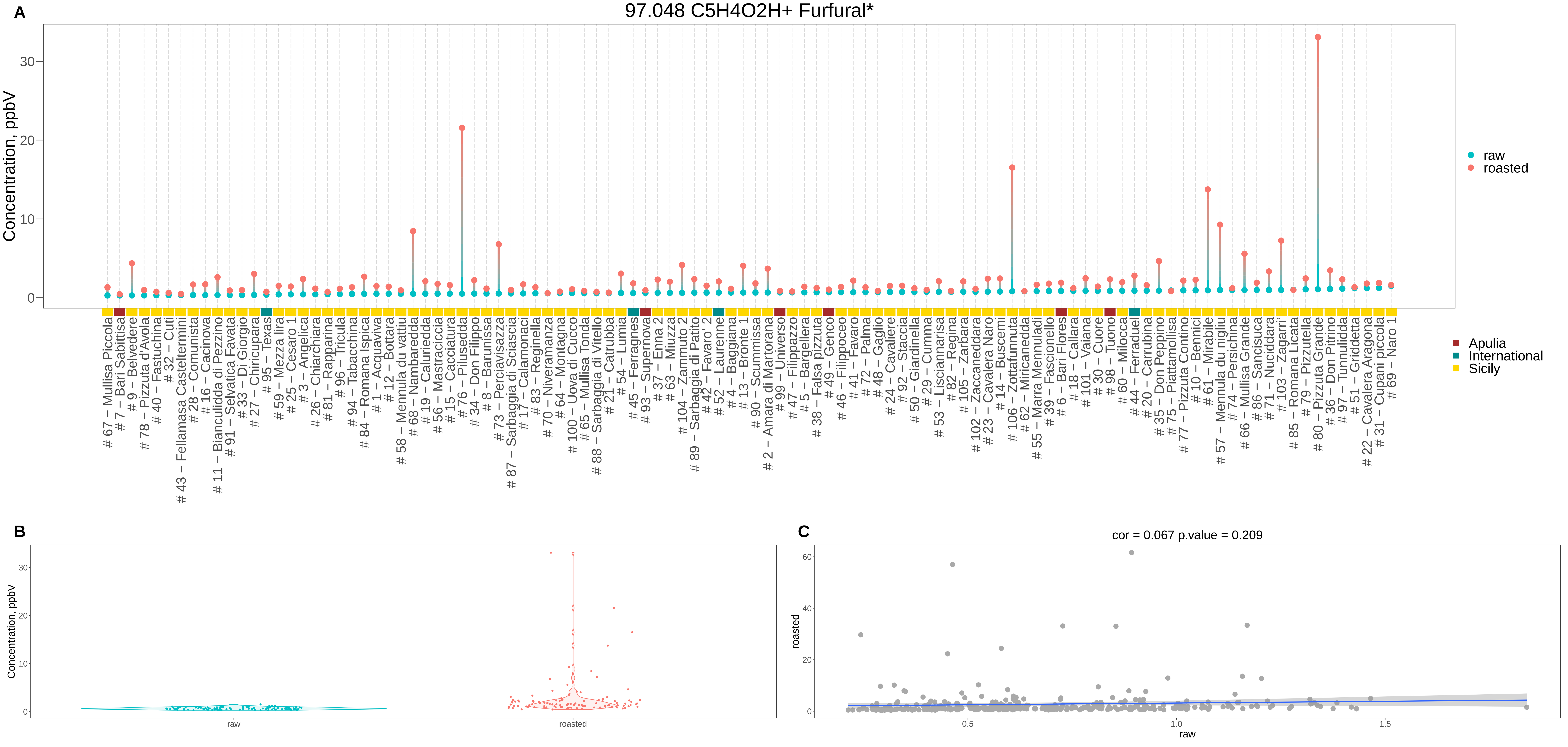
A
97.048 C5H4O2H+ Furfural*
Concentration, ppbV
0
10
20
30
raw
roasted
Apulia
International
Sicily
67 – Mullisa Piccola
7 – Bari Sabitissa
9 – Belvedere
78 – Pizzuta d'Avola
40 – Fastuchina
32 – Cuti
43 – Fellamasa Casteltermini
28 – Comunista
16 – Cacinova
11 – Bianculidda di Pezzino
91 – Selvatica Favata
33 – Di Giorgio
27 – Chiricupara
95 – Texas
59 – Mezza lira
25 – Cesaro 1
3 – Angelica
26 – Chiarchiara
81 – Rapparina
96 – Tricula
94 – Tabacchina
84 – Romana Ispica
1 – Acquaviva
12 – Bottara
58 – Mennula du vattiu
68 – Nambaredda
19 – Caluriedda
56 – Mastraciccia
15 – Cacciatura
76 – Pilusedda
34 – Don Filippo
8 – Barunissa
73 – Perciavisazza
87 – Sarbaggia di Sciascia
17 – Calamonaci
83 – Reginella
70 – Niviramanza
64 – Montagna
100 – Uova di Cucco
65 – Mullisa Tonda
88 – Sarbaggia di Vitello
21 – Catrubba
54 – Lumia
45 – Ferragnes
93 – Supernova
37 – Enna 2
63 – Miuzza
104 – Zammuto 2
89 – Sarbaggia di Patito
42 – Favaro' 2
52 – Laurenne
4 – Baggiana
13 – Bronte 1
90 – Scummissa
2 – Amara di Martorana
99 – Universo
47 – Filippazzo
5 – Bargellera
38 – Falsa pizzuta
49 – Genco
46 – Filippoceo
41 – Favaro
72 – Palma
48 – Gaglio
24 – Cavaliere
92 – Staccia
50 – Giardinella
29 – Cumma
53 – Liscianarisa
82 – Regina
105 – Zarbara
102 – Zaccaneddara
23 – Cavalera Naro
14 – Buscemi
106 – Zottafunnuta
62 – Miricanedda
55 – Marra Mennuladi
39 – Fascionello
6 – Bari Flores
18 – Callara
101 – Vaiana
30 – Cuore
98 – Tuono
60 – Milocca
44 – Ferraduel
20 – Carrubina
35 – Don Peppino
75 – Piattamollisa
77 – Pizzuta Contino
10 – Bennici
61 – Mirabile
57 – Mennula du nigliu
74 – Persichina
66 – Mullisa Grande
86 – Sancisuca
71 – Nuciddara
103 – Zagarri'
85 – Romana Licata
79 – Pizzutella
80 – Pizzuta Grande
36 – Don Pitrino
97 – Tunnulidda
51 – Griddetta
22 – Cavalera Aragona
31 – Cupani piccola
69 – Naro 1
B
Concentration, ppbV
0
10
20
30
raw
roasted
C
cor = 0.067 p.value = 0.209
roasted
0
20
40
60
0.5
1.0
1.5
raw

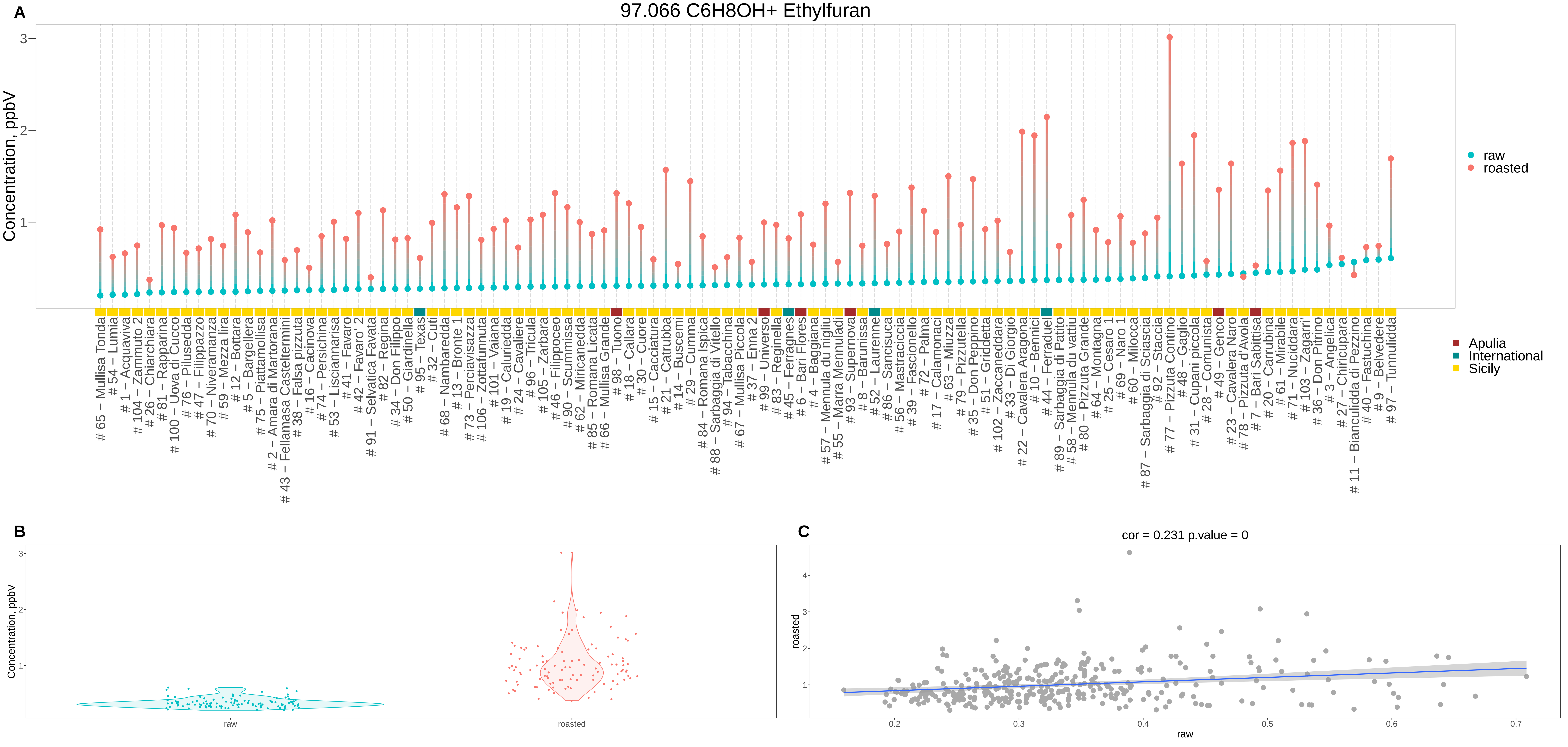
A
97.066 C6H8OH+ Ethylfuran
Concentration, ppbV
1
2
3
raw
roasted
Apulia
International
Sicily
65 – Mullisa Tonda
54 – Lumia
1 – Acquaviva
104 – Zammuto 2
26 – Chiarchiara
81 – Rapparina
100 – Uova di Cucco
76 – Pilusedda
47 – Filippazzo
70 – Niveramanza
59 – Mezza lira
12 – Bottara
5 – Bargellera
75 – Piattamollisa
2 – Amara di Martorana
43 – Fellamasa Casteltermini
38 – Falsa pizzuta
16 – Cacinova
74 – Persichina
53 – Lisciannarisa
41 – Favaro
42 – Favaro' 2
91 – Selvatica Favata
82 – Regina
34 – Don Filippo
50 – Giardinella
95 – Texas
32 – Cuti
68 – Nambaredda
13 – Bronte 1
73 – Perciavisazza
106 – Zottafunnuta
101 – Vaiana
19 – Caluriedda
24 – Cavaliere
96 – Tricula
105 – Zarbara
46 – Filippoceo
90 – Scummissa
62 – Miricanedda
85 – Romana Licata
66 – Mullisa Grande
98 – Tuono
18 – Callara
30 – Cuore
15 – Cacciatura
21 – Catrubba
14 – Buscemi
29 – Cumma
84 – Romana Ispica
88 – Sarbaggia di Vitello
94 – Tabacchina
67 – Mullisa Piccola
37 – Enna 2
99 – Universo
83 – Reginella
45 – Ferragnes
6 – Bari Flores
4 – Baggiana
57 – Mennula du nigliu
55 – Marra Mennuladi
93 – Supernova
8 – Barunissa
52 – Laurenne
86 – Sancisuca
56 – Mastracicca
39 – Fascionello
72 – Palma
17 – Calamonaci
63 – Miuzza
79 – Pizzutella
35 – Don Peppino
51 – Griddetta
102 – Zaccaneddara
33 – Di Giorgio
22 – Cavalera Aragona
10 – Bennici
44 – Ferraduel
89 – Sarbaggia di Patito
58 – Mennula du vattiu
80 – Pizzuta Grande
64 – Montagna
25 – Cesaro 1
69 – Naro 1
60 – Milocca
87 – Sarbaggia di Sciascia
92 – Staccia
77 – Pizzuta Contino
48 – Gaglio
31 – Cupani piccola
28 – Comunista
49 – Genco
23 – Cavalera Naro
78 – Pizzuta d'Avola
7 – Bari Sabittisa
20 – Carrubina
61 – Mirabile
71 – Nuciddara
103 – Zagarri'
36 – Don Pitrino
3 – Angelica
27 – Chiricupara
11 – Bianculidda di Pezzino
40 – Fastuchina
9 – Belvedere
97 – Tunnulidda
B
Concentration, ppbV
1
2
3
raw
roasted
C
cor = 0.231 p.value = 0
roasted
1
2
3
4
0.2
0.3
0.4
0.5
0.6
0.7
raw

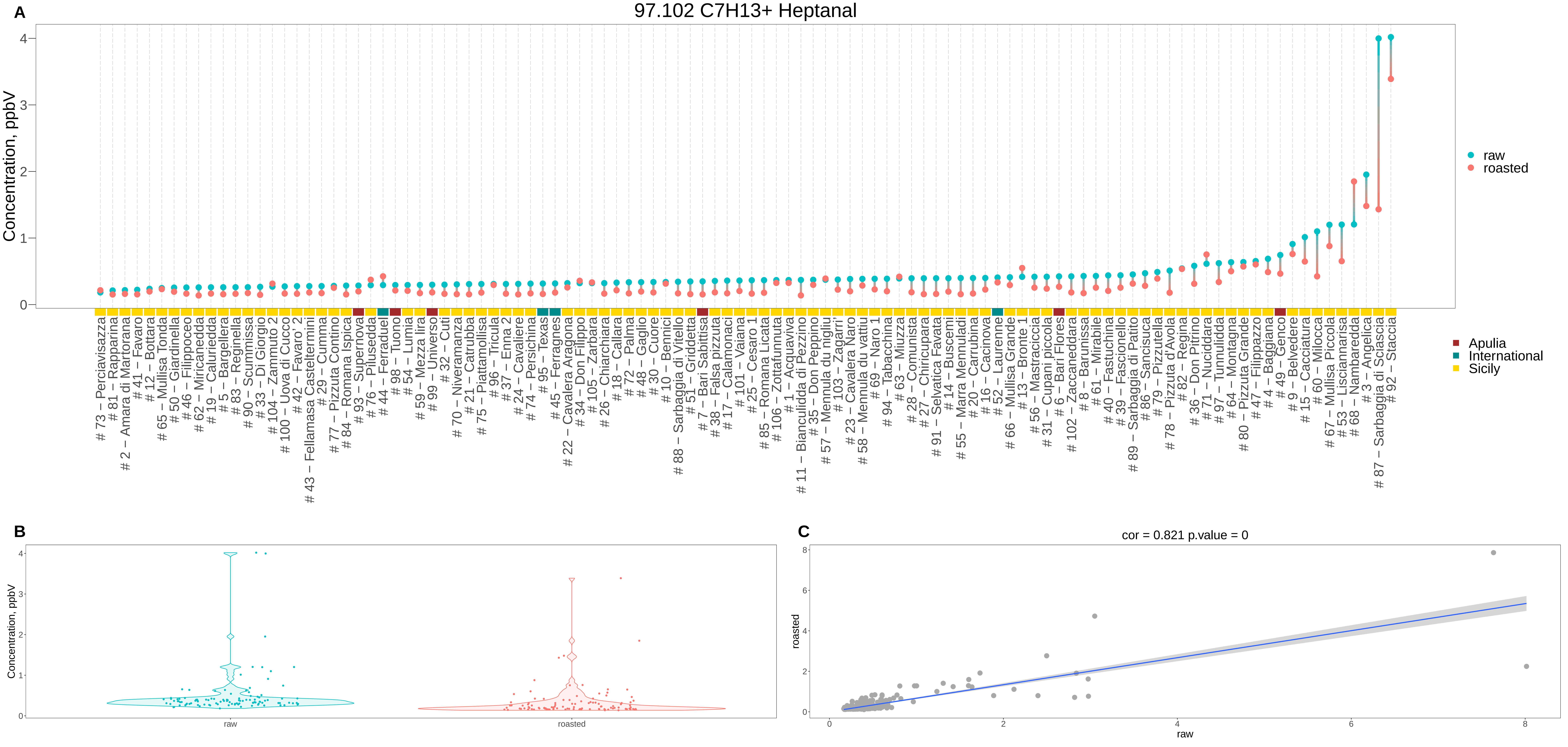

A
97.102 C7H13+ Heptanal
Concentration, ppbV
0
1
2
3
4
raw
roasted
Apulia
International
Sicily
73 – Perciavisazza
81 – Rappanina
2 – Amara di Martorana
41 – Favaro
12 – Bottara
65 – Mullisa Tonda
50 – Giardinella
46 – Filippoceo
62 – Miricanedda
19 – Caluriedda
5 – Bargellera
83 – Reginella
90 – Scummissa
33 – Di Giorgio
104 – Zammuto 2
100 – Uova di Cucco
42 – Favaro' 2
43 – Fellamasa Casteltermini
29 – Cumma
77 – Pizzuta Contino
84 – Romana Ispica
93 – Supernova
76 – Pilusedda
44 – Ferraduel
98 – Tuono
54 – Lumia
59 – Mezza lira
99 – Universo
32 – Cuti
70 – Niveramanza
21 – Catrubba
75 – Piattamollisa
96 – Tricula
37 – Enna 2
24 – Cavaliere
74 – Persichina
95 – Texas
45 – Ferragnes
22 – Cavalera Aragona
34 – Don Filippo
105 – Zarbara
26 – Chiarchiara
18 – Callara
72 – Palma
48 – Gaglio
30 – Cuore
10 – Bennici
88 – Sarbaggia di Vitello
51 – Griddetta
7 – Bari Sabittisa
38 – Falsa pizzuta
17 – Calamonaci
101 – Vaiana
25 – Cesaro 1
85 – Romana Licata
106 – Zottafunnuta
1 – Acquaviva
11 – Bianculidda di Pezzino
35 – Don Peppino
57 – Mennula du nigliu
103 – Zagarri
23 – Cavalera Naro
58 – Mennula du vattiu
69 – Naro 1
94 – Tabacchina
63 – Miuzza
28 – Comunista
27 – Chiricupara
91 – Selvatica Favata
14 – Buscemi
55 – Marra Mennuladi
20 – Carrubina
16 – Cacinova
52 – Laurenne
66 – Mullisa Grande
13 – Bronte 1
56 – Mastraciccia
31 – Cupani piccola
6 – Bari Flores
102 – Zaccaneddara
8 – Barunissa
61 – Mirabile
40 – Fastuchino
39 – Fascionello
89 – Sarbaggia di Patito
86 – Sancisuca
79 – Pizzutella
78 – Pizzuta d'Avola
82 – Regina
36 – Don Pitrino
71 – Nuciddara
97 – Tunnulidda
64 – Montagna
80 – Pizzuta Grande
47 – Filippazzo
4 – Baggiana
49 – Genco
9 – Belvedere
15 – Cacciatura
60 – Milocca
67 – Mullisa Piccola
53 – Liscianarisa
68 – Nambaredda
3 – Angelica
87 – Sarbaggia di Sciascia
92 – Staccia
B
Concentration, ppbV
0
1
2
3
4
raw
roasted
C
cor = 0.821 p.value = 0
roasted
raw
0
2
4
6
8

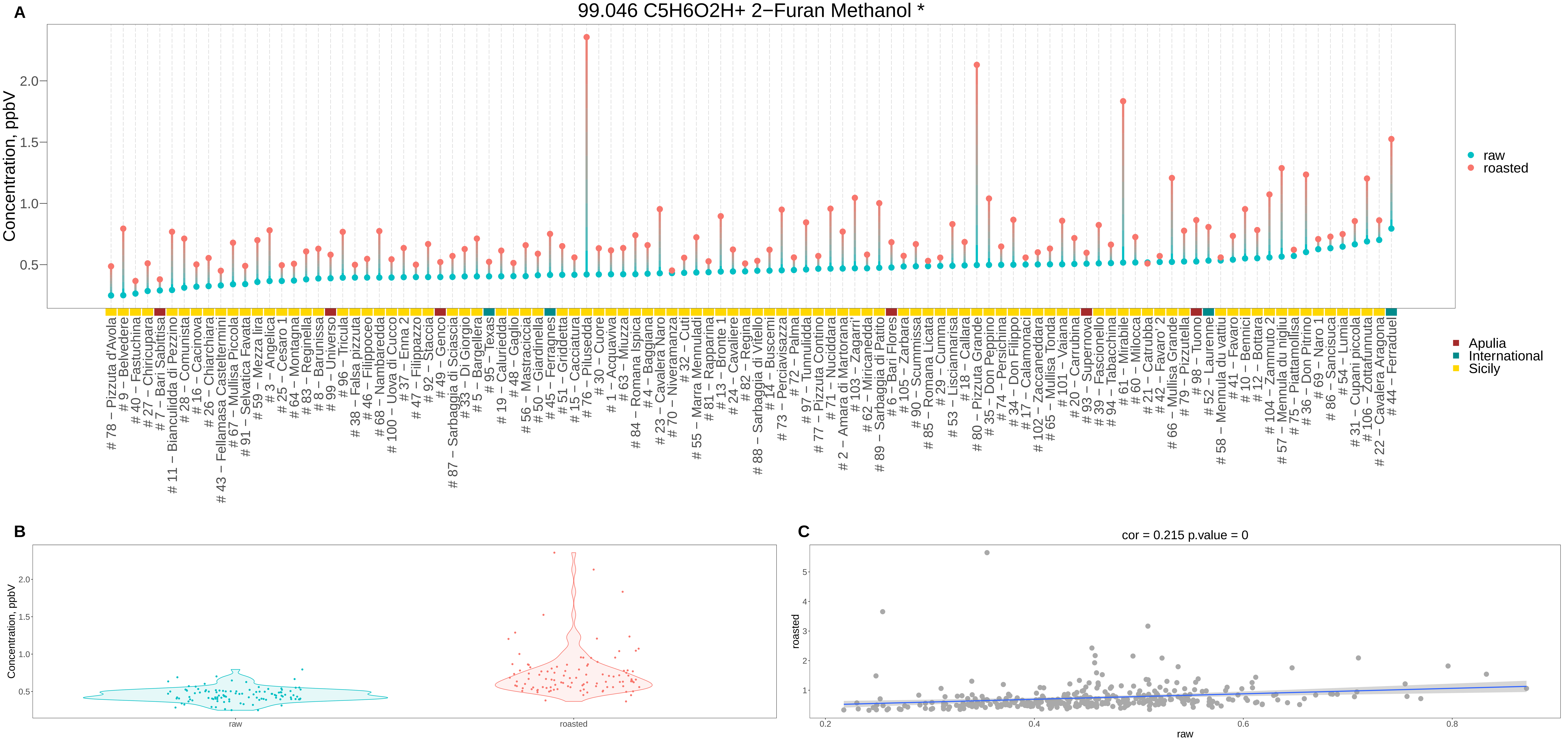
A
99.046 C5H6O2H+ 2−Furan Methanol *
Concentration, ppbV
2.0
1.5
1.0
0.5
raw
roasted
Apulia
International
Sicily
78 – Pizzuta d'Avola
9 – Belvedere
40 – Fastuchina
27 – Chiricupara
7 – Bari Sabittisa
11 – Biancuiidda di Pezzino
28 – Comunista
16 – Cacinova
26 – Chiarchiara
43 – Fellamasa Casteltermini
67 – Mullisa Piccola
91 – Selvatica Favata
59 – Mezza lira
3 – Angelica
25 – Cesaro 1
64 – Montagna
83 – Reginella
8 – Barunissa
99 – Universo
96 – Tricula
38 – Falsa pizzuta
46 – Filippoceo
68 – Nambaredda
100 – Uova di Cucco
37 – Enna 2
47 – Filippazzo
92 – Staccia
49 – Genco
87 – Sarbaggia di Sciascia
33 – Di Giorgio
5 – Bargellera
95 – Texas
19 – Caluriedda
48 – Gaglio
56 – Mastraciccia
50 – Giardinella
45 – Ferragnes
51 – Griddetta
15 – Cacciatura
76 – Pilusedda
30 – Cuore
1 – Acquaviva
63 – Miuzza
84 – Romana Ispica
4 – Bagglana
23 – Cavalera Naro
70 – Niveramanza
32 – Cuti
55 – Marra Mennuladi
81 – Rapparina
13 – Bronte 1
24 – Cavaliere
82 – Regina
88 – Sarbaggia di Vitello
14 – Buscemi
73 – Perciavisazza
72 – Palma
97 – Tunnulidda
77 – Pizzuta Contino
71 – Nuciddara
2 – Amara di Martorana
103 – Zagarri'
62 – Miricanedda
89 – Sarbaggia di Patito
6 – Bari Flores
105 – Zarbara
90 – Scummissa
85 – Romana Licata
29 – Cumma
53 – Liscianarisa
18 – Callara
80 – Pizzuta Grande
35 – Don Peppino
74 – Persichina
34 – Don Filippo
17 – Calamonaci
102 – Zaccaneddara
65 – Mullisa Tonda
101 – Vaiana
20 – Carrubina
93 – Supernova
39 – Fascionello
94 – Tabacchina
61 – Mirabile
60 – Milocca
21 – Catrubba
42 – Favaro' 2
66 – Mullisa Grande
79 – Pizzutella
98 – Tuono
52 – Laurenne
58 – Mennula du vattiu
41 – Favaro
10 – Bennici
12 – Bottara
104 – Zammuto 2
57 – Mennula du nigliu
75 – Piattamollisa
36 – Don Pitrino
69 – Naro 1
86 – Sancisuca
54 – Lumia
31 – Cupani piccola
106 – Zottafunnuta
22 – Cavalera Aragona
44 – Ferraduel
B
Concentration, ppbV
2.0
1.5
1.0
0.5
raw
roasted
C
cor = 0.215 p.value = 0
roasted
5
4
3
2
1
0.2
0.4
0.6
0.8
raw

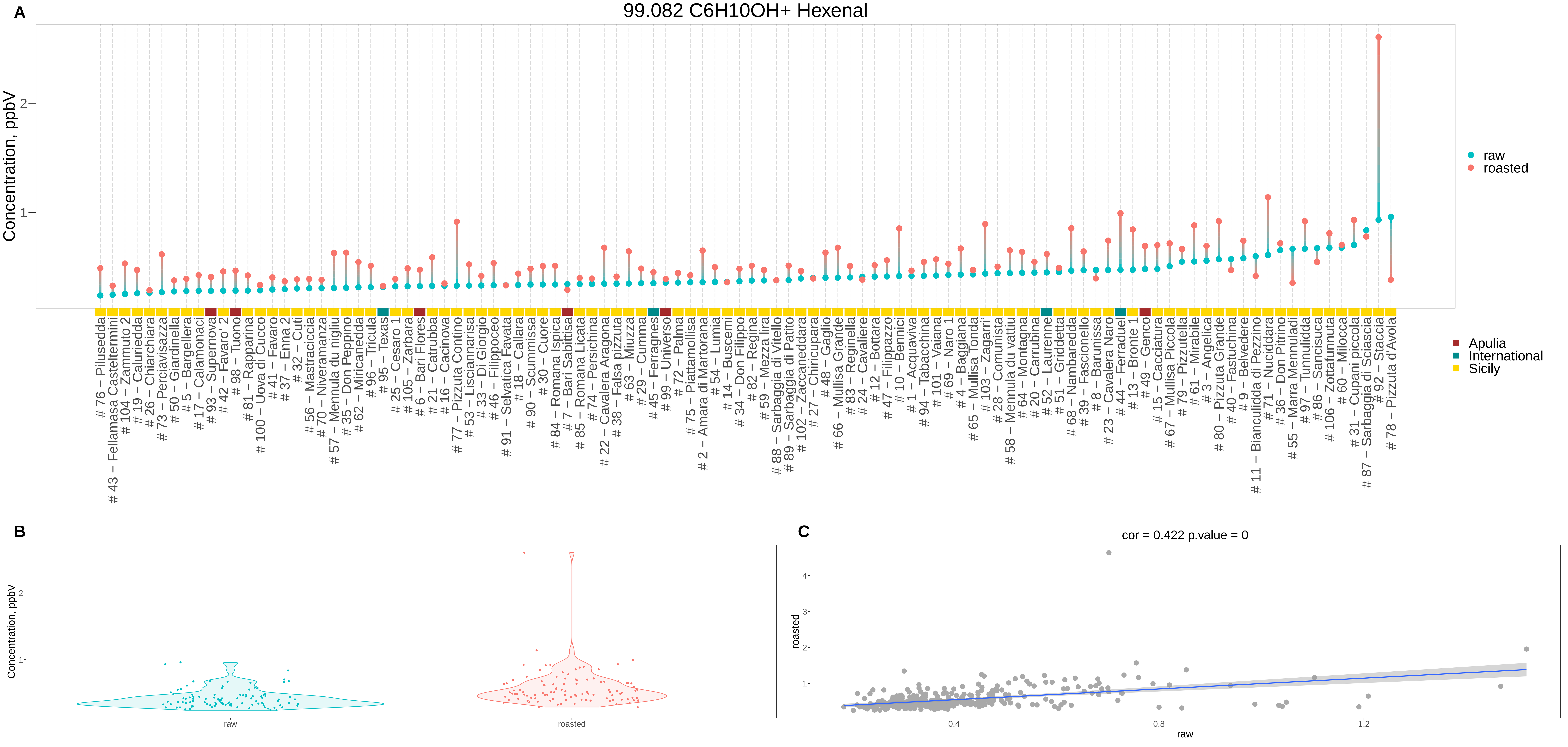
A
99.082 C6H10OH+ Hexenal
Concentration, ppbV
2
1
raw
roasted
Apulia
International
Sicily
76 – Pilusedda
43 – Fellamasa Casteltermini
104 – Zammuto 2
19 – Caluriedda
26 – Chiarchiara
73 – Perciavisazza
50 – Giardinella
5 – Bargellera
17 – Calamonaci
93 – Supernova
42 – Favaro' 2
98 – Tuono
81 – Rapparina
100 – Uova di Cucco
41 – Favaro
37 – Enna 2
32 – Cuti
56 – Mastraciccia
70 – Niveramanza
57 – Mennula du nigliu
35 – Don Peppino
62 – Miricanedda
96 – Tricula
95 – Texas
25 – Cesaro 1
105 – Zarbara
6 – Bari Flores
21 – Catrubba
16 – Cacinova
77 – Pizzuta Contino
53 – Liscianarisa
33 – Di Giorgio
46 – Filippoceo
91 – Selvatica Favata
18 – Callara
90 – Scummissa
30 – Cuore
84 – Romana Ispica
7 – Bari Sabittisa
85 – Romana Licata
74 – Persichina
22 – Cavalera Aragona
38 – Falsa pizzuta
63 – Miuzza
29 – Cumma
45 – Ferragnes
99 – Universo
72 – Palma
75 – Piattamollisa
2 – Amara di Martorana
54 – Lumia
14 – Buscemi
34 – Don Filippo
82 – Regina
59 – Mezza lira
88 – Sarbaggia di Vitello
89 – Sarbaggia di Patito
102 – Zaccaneddara
27 – Chiricupara
48 – Gaglio
66 – Mullisa Grande
83 – Reginella
24 – Cavaliere
12 – Bottara
47 – Filippazzo
10 – Bennici
1 – Acquaviva
94 – Tabacchina
101 – Vaiana
69 – Naro 1
4 – Baggiana
65 – Mullisa Tonda
103 – Zagarri'
28 – Comunista
58 – Mennula du vattiu
64 – Montagna
20 – Carrubina
52 – Laurenne
51 – Griddetta
68 – Nambaredda
39 – Fascionello
8 – Barunissa
23 – Cavalera Naro
44 – Ferraduel
13 – Bronte 1
49 – Genco
15 – Cacciatura
67 – Mullisa Piccola
79 – Pizzutella
61 – Mirabile
3 – Angelica
80 – Pizzuta Grande
40 – Fastuchina
9 – Belvedere
11 – Bianculidda di Pezzino
71 – Nuciddara
36 – Don Pitrino
55 – Marra Mennuladi
97 – Tunnulidda
86 – Sancisuca
106 – Zottafunnuta
60 – Milocca
31 – Cupani piccola
87 – Sarbaggia di Sciascia
92 – Staccia
78 – Pizzuta d'Avola
B
Concentration, ppbV
2
1
raw
roasted
C
cor = 0.422 p.value = 0
roasted
4
3
2
1
0.4
0.8
1.2
raw

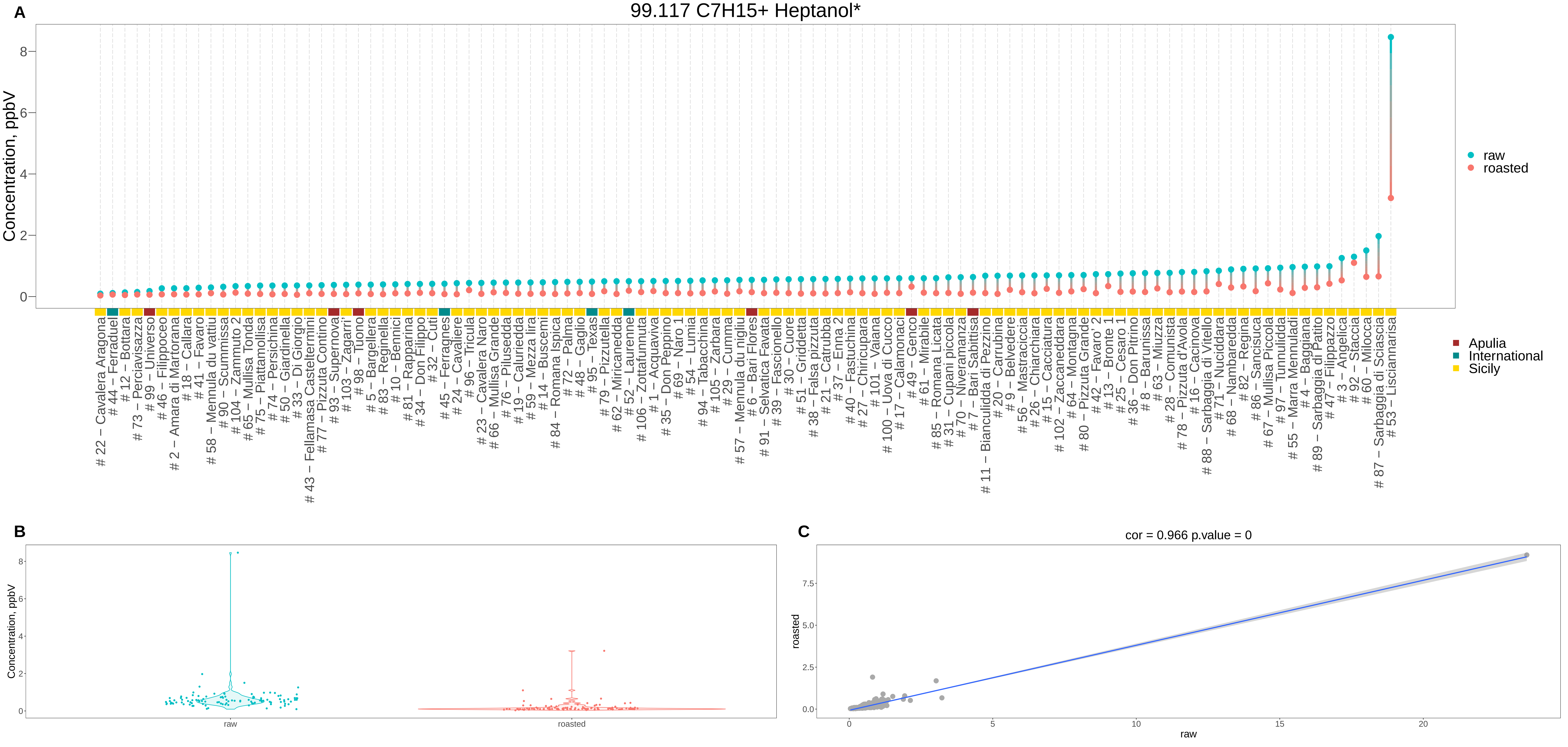

A
99.117 C7H15+ Heptanol*
Concentration, ppbV
0
2
4
6
8
raw
roasted
Apulia
International
Sicily
22 – Cavalera Aragona
44 – Ferraduel
12 – Bottara
73 – Perciavisazza
99 – Universo
46 – Filippoceo
2 – Amara di Martorana
18 – Callara
41 – Favaro
58 – Mennula du vattiu
90 – Scummissa
104 – Zammuto 2
65 – Mullisa Tonda
75 – Piattamollisa
74 – Persichina
50 – Giardinella
33 – Di Giorgio
43 – Fellamasa Casteltermini
77 – Pizzuta Contino
93 – Supernova
103 – Zagarri'
98 – Tuono
5 – Bargellera
83 – Reginella
10 – Bennici
81 – Rapparina
34 – Don Filippo
32 – Cuti
45 – Ferragnes
24 – Cavaliere
96 – Tricula
23 – Cavalera Naro
66 – Mullisa Grande
76 – Pilusedda
19 – Caluriedda
59 – Mezza lira
14 – Buscemi
84 – Romana Ispica
72 – Palma
48 – Gaglio
95 – Texas
79 – Pizzutella
62 – Miricanedda
52 – Laurenne
106 – Zottafunnuta
1 – Acquaviva
35 – Don Peppino
69 – Naro 1
54 – Lumia
94 – Tabacchina
105 – Zarbara
29 – Cumma
57 – Mennula du nigliu
6 – Bari Flores
91 – Selvatica Favata
39 – Fascionello
30 – Cuore
51 – Griddetta
38 – Falsa pizzuta
21 – Catrubba
37 – Enna 2
40 – Fastuchina
27 – Chiricupara
101 – Vaiana
100 – Uova di Cucco
17 – Calamonaci
49 – Genco
61 – Mirabile
85 – Romana Licata
31 – Cupani piccola
70 – Niveramanza
7 – Bari Sabittisa
11 – Bianculidda di Pezzino
20 – Carrubina
9 – Belvedere
56 – Mastracicia
26 – Chiarchiara
15 – Cacciatura
102 – Zaccaneddara
64 – Montagna
80 – Pizzuta Grande
42 – Favaro' 2
13 – Bronte 1
25 – Cesaro 1
36 – Don Pitrino
8 – Barunissa
63 – Miuzza
28 – Comunista
78 – Pizzuta d'Avola
16 – Cacinova
88 – Sarbaggia di Vitello
71 – Nuciddara
68 – Nambaredda
82 – Regina
86 – Sancisuca
67 – Mullisa Piccola
97 – Tunnuliddi
55 – Marra Mennuladi
4 – Baggiana
89 – Sarbaggia di Patito
47 – Filippazzo
3 – Angelica
92 – Staccia
60 – Milocca
87 – Sarbaggia di Sciascia
53 – Liscianarisa
B
Concentration, ppbV
0
2
4
6
8
raw
roasted
C
cor = 0.966 p.value = 0
roasted
0.0
2.5
5.0
7.5
raw
0
5
10
15
20

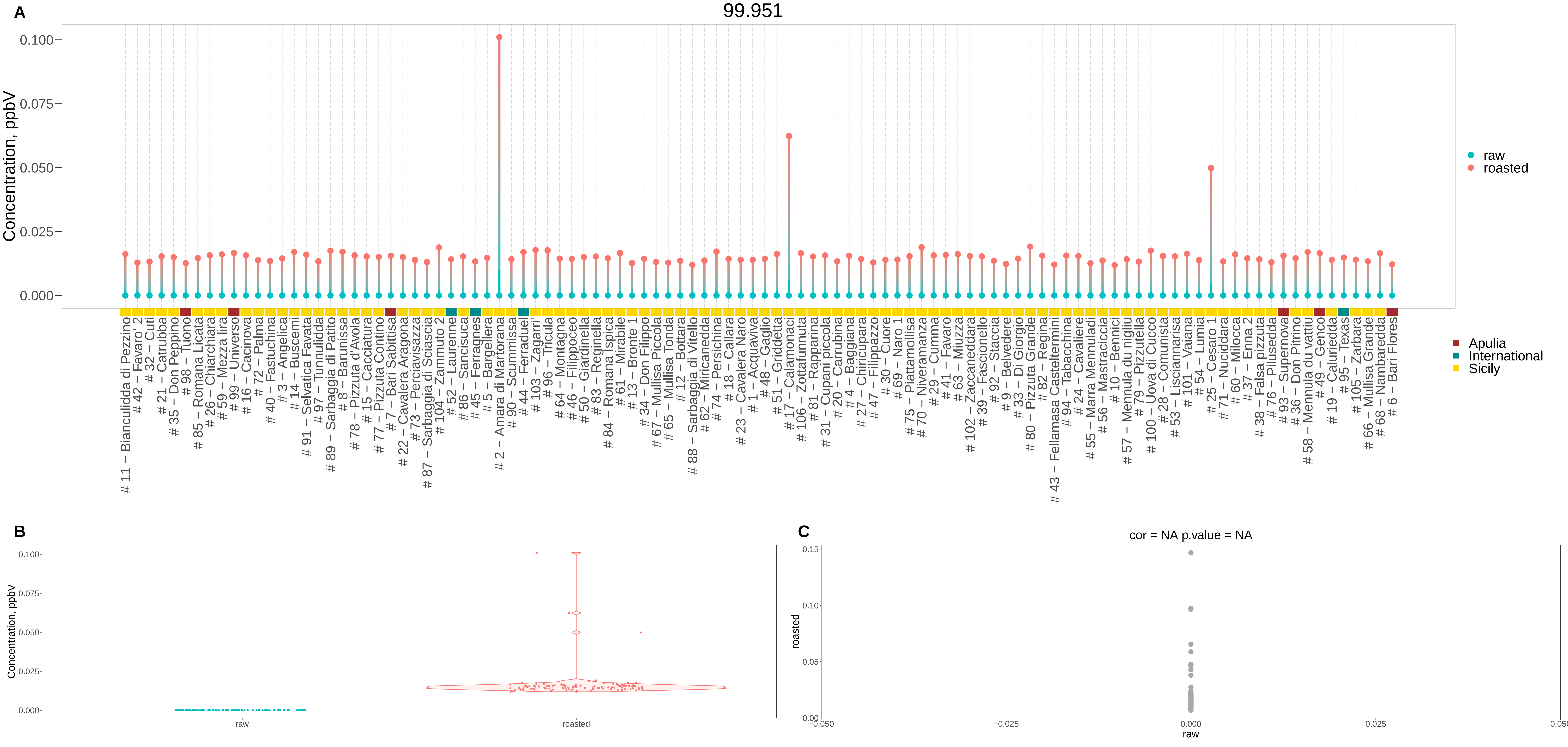
A
99.951
Concentration, ppbV
0.100
0.075
0.050
0.025
0.000
raw
roasted
Apulia
International
Sicily
11 – Bianculidda di Pezzino
42 – Favaro' 2
32 – Cuti
21 – Catrubba
35 – Don Peppino
98 – Tuono
85 – Romana Licata
26 – Chiarchiara
59 – Mezza lira
99 – Universo
16 – Cacinova
72 – Palma
40 – Fastuchina
3 – Angelica
14 – Buscemi
91 – Selvatica Favata
97 – Tunnulidda
89 – Sarbaggia di Patito
8 – Barunissa
78 – Pizzuta d'Avola
15 – Cacciatura
77 – Pizzuta Contino
7 – Bari Sabittisa
22 – Cavalera Aragona
73 – Perciavisazza
87 – Sarbaggia di Sciascia
104 – Zammuto 2
52 – Laurenne
86 – Sancisuca
45 – Ferragnes
5 – Bargellera
2 – Amara di Martorana
90 – Scummissa
44 – Ferraduel
103 – Zagarri'
96 – Tricula
64 – Montagna
46 – Filippoceo
50 – Giardinella
83 – Reginella
84 – Romana Ispica
61 – Mirabile
13 – Bronte 1
34 – Don Filippo
67 – Mullisa Piccola
65 – Mullisa Tonda
12 – Bottara
88 – Sarbaggia di Vitello
62 – Miricanedda
74 – Persichina
18 – Callara
23 – Cavalera Naro
1 – Acquaviva
48 – Gaglio
51 – Griddetta
17 – Calamonaci
106 – Zottafunnuta
81 – Rapparina
31 – Cupani piccola
20 – Carrubina
4 – Baggiana
27 – Chiricupara
47 – Filippazzo
30 – Cuore
69 – Naro 1
75 – Piattamollisa
70 – Niveramanza
29 – Cumma
41 – Favaro
63 – Miuzza
102 – Zaccaneddara
39 – Fascionello
92 – Staccia
9 – Belvedere
33 – Di Giorgio
80 – Pizzuta Grande
82 – Regina
43 – Fellamasa Casteltermini
94 – Tabacchina
24 – Cavaliere
55 – Marra Mennuladi
56 – Mastraciccia
10 – Bennici
57 – Mennula du nigliu
79 – Pizzutella
100 – Uova di Cucco
28 – Comunista
53 – Lisciannarisa
101 – Vaiana
54 – Lumia
25 – Cesaro 1
71 – Nuciddara
60 – Milocca
37 – Enna 2
38 – Falsa pizzuta
76 – Pilusedda
93 – Supernova
36 – Don Pitrino
58 – Mennula du vattiu
49 – Genco
19 – Caluriedda
95 – Texas
105 – Zarbara
66 – Mullisa Grande
68 – Nambaredda
6 – Bari Flores
B
Concentration, ppbV
0.100
0.075
0.050
0.025
0.000
raw
roasted
C
cor = NA p.value = NA
roasted
0.15
0.10
0.05
0.00
−0.050
−0.025
0.000
0.025
raw

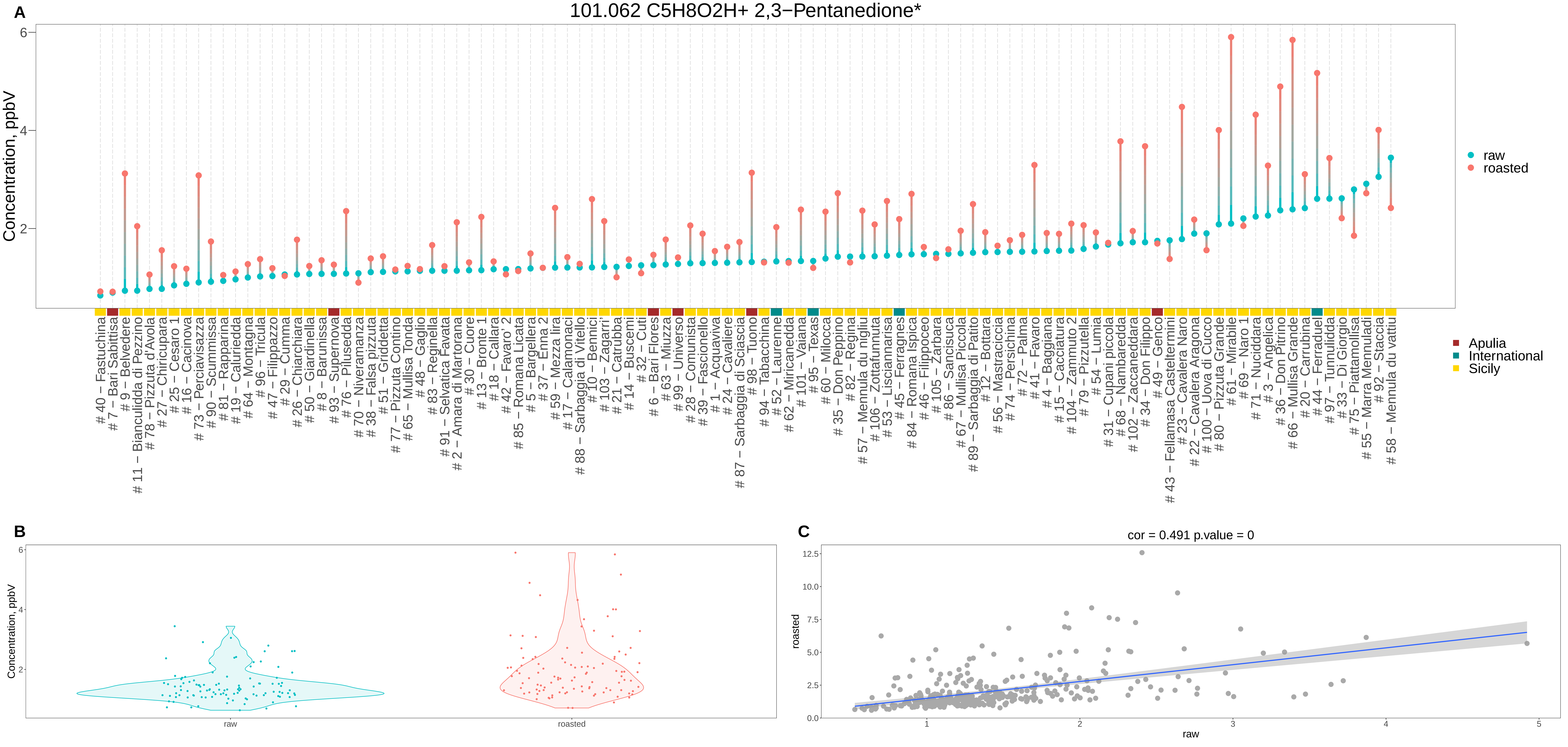
A
101.062 C5H8O2H+ 2,3−Pentanedione*
Concentration, ppbV
raw
roasted
Apulia
International
Sicily
B
Concentration, ppbV
raw
roasted
C
cor = 0.491 p.value = 0
roasted
raw

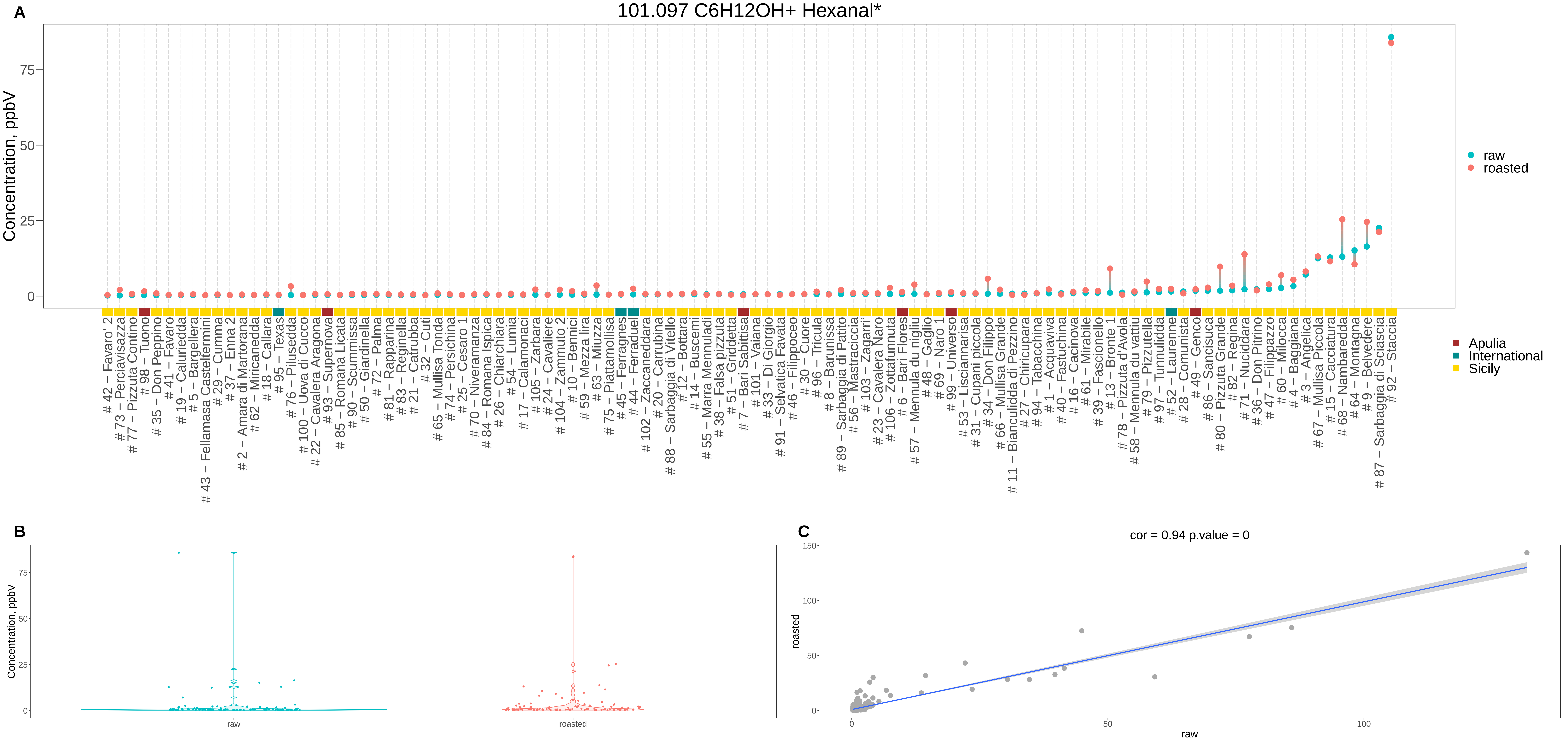

A
101.097 C6H12OH+ Hexanal*
Concentration, ppbV
0
25
50
75
raw
roasted
Apulia
International
Sicily
42 – Favaro' 2
73 – Perciavisazza
77 – Pizzuta Contino
98 – Tuono
35 – Don Peppino
41 – Favaro
19 – Caluriedda
5 – Bargellera
43 – Fellamasa Casteltermini
29 – Cumma
37 – Enna 2
2 – Amara di Martorana
62 – Miricanedda
18 – Callara
95 – Texas
76 – Pilusedda
100 – Uova di Cucco
22 – Cavalera Aragona
93 – Supernova
85 – Romana Licata
90 – Scummissa
50 – Giardinella
72 – Palma
81 – Rapparina
83 – Reginella
21 – Catrubba
32 – Cuti
65 – Mullisa Tonda
74 – Persichina
25 – Cesaro 1
70 – Niveramanza
84 – Romana Ispica
26 – Chiarchiara
54 – Lumia
17 – Calamonaci
105 – Zarbara
24 – Cavaliere
104 – Zammuto 2
10 – Bennici
59 – Mezza lira
63 – Miuzza
75 – Piattamollisa
45 – Ferragnes
44 – Ferraduel
102 – Zaccaneddara
20 – Carrubina
88 – Sarbaggia di Vitello
12 – Bottara
14 – Buscemi
55 – Marra Mennuladi
38 – Falsa pizzuta
51 – Griddetta
7 – Bari Sabitisa
101 – Vaiana
33 – Di Giorgio
91 – Selvatica Favata
46 – Filippoceo
30 – Cuore
96 – Tricula
8 – Barunissa
89 – Sarbaggia di Patito
56 – Mastraciccia
103 – Zagarri'
23 – Cavalera Naro
106 – Zottafunnuta
6 – Bari Flores
57 – Mennula du nigliu
48 – Gaglio
69 – Naro 1
99 – Universo
53 – Liscianarisa
31 – Cupani piccola
34 – Don Filippo
66 – Mullisa Grande
11 – Bianculidda di Pezzino
27 – Chiricupara
94 – Tabacchina
1 – Acquaviva
40 – Fastuchina
16 – Cacinova
61 – Mirabile
39 – Fascionello
13 – Bronte 1
78 – Pizzuta d'Avola
58 – Mennula du vattiu
79 – Pizzutella
97 – Tunnulidda
52 – Laurenne
28 – Comunista
49 – Genco
86 – Sancisuca
80 – Pizzuta Grande
82 – Regina
71 – Nuciddara
36 – Don Pitrino
47 – Filippazzo
60 – Milocca
4 – Baggiana
3 – Angelica
67 – Mullisa Piccola
15 – Cacciatura
68 – Nambaredda
64 – Montagna
9 – Belvedere
87 – Sarbaggia di Sciascia
92 – Staccia
B
Concentration, ppbV
0
25
50
75
raw
roasted
C
cor = 0.94 p.value = 0
roasted
0
50
100
150
raw
0
50
100

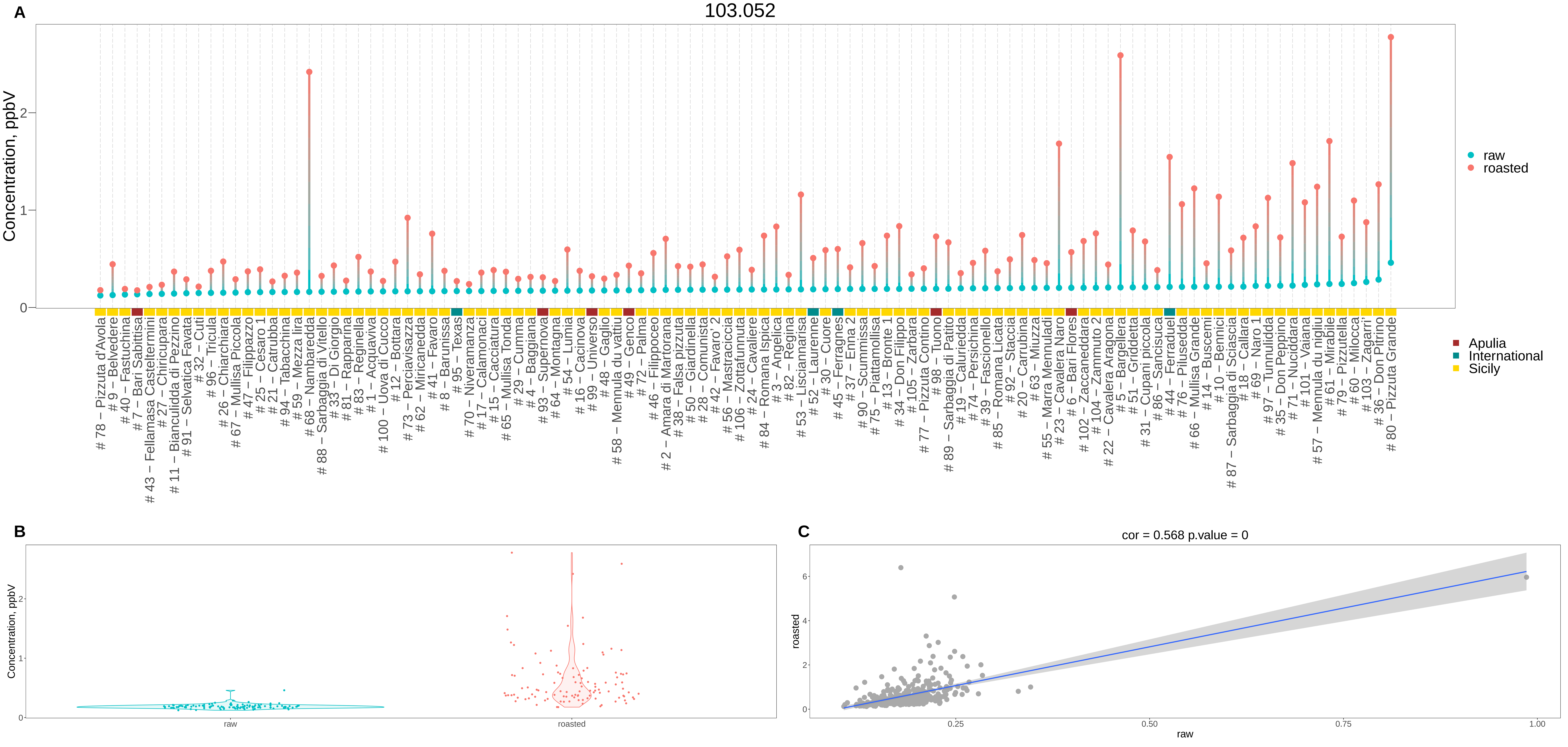
A
103.052
Concentration, ppbV
0
1
2
raw
roasted
Apulia
International
Sicily
78 – Pizzuta d'Avola
9 – Belvedere
40 – Fastuchina
7 – Bari Sabittisa
43 – Fellamasa Casteltermini
27 – Chiricupara
11 – Bianculidda di Pezzino
91 – Selvatica Favata
32 – Cuti
96 – Tricula
26 – Chiarchiara
67 – Mullisa Piccola
47 – Filippazzo
25 – Cesaro 1
21 – Catrubba
94 – Tabacchina
59 – Mezza lira
68 – Nambaredda
88 – Sarbaggia di Vitello
33 – Di Giorgio
81 – Rapparina
83 – Reginella
1 – Acquaviva
100 – Uova di Cucco
12 – Bottara
73 – Perciavisazza
62 – Miricanedda
41 – Favaro
8 – Barunissa
95 – Texas
70 – Niveramanza
17 – Calamonaci
15 – Cacciatura
65 – Mullisa Tonda
29 – Cumma
4 – Baggiana
93 – Supernova
64 – Montagna
54 – Lumia
16 – Cacinova
99 – Universo
48 – Gaglio
58 – Mennula du vattiu
49 – Genco
72 – Palma
46 – Filippoceo
2 – Amara di Martorana
38 – Falsa pizzuta
50 – Giardinella
28 – Comunista
42 – Favaro' 2
56 – Mastraciccia
106 – Zottafunnuta
24 – Cavaliere
84 – Romana Ispica
3 – Angelica
82 – Regina
53 – Lisciannarisa
52 – Laurenne
30 – Cuore
45 – Ferragnes
37 – Enna 2
90 – Scummissa
75 – Piattamollisa
13 – Bronte 1
34 – Don Filippo
105 – Zarbara
77 – Pizzuta Contino
98 – Tuono
89 – Sarbaggia di Patito
19 – Caluriedda
74 – Persichina
39 – Fascionello
85 – Romana Licata
92 – Staccia
20 – Carrubina
63 – Miuzza
55 – Marra Mennuladi
23 – Cavalera Naro
6 – Bari Flores
102 – Zaccaneddara
104 – Zammuto 2
22 – Cavalera Aragona
5 – Bargellera
51 – Griddetta
31 – Cupani piccola
86 – Sancisuca
44 – Ferraduel
76 – Pilusedda
66 – Mullisa Grande
14 – Buscemi
10 – Bennici
87 – Sarbaggia di Sciascia
18 – Callara
69 – Naro 1
97 – Tunnulidda
35 – Don Peppino
71 – Nuciddara
101 – Vaiana
57 – Mennula du nigliu
61 – Mirabile
79 – Pizzutella
60 – Milocca
103 – Zagarri'
36 – Don Pitrino
80 – Pizzuta Grande
B
Concentration, ppbV
0
1
2
raw
roasted
C
cor = 0.568 p.value = 0
roasted
0
2
4
6
raw
0.25
0.50
0.75
1.00

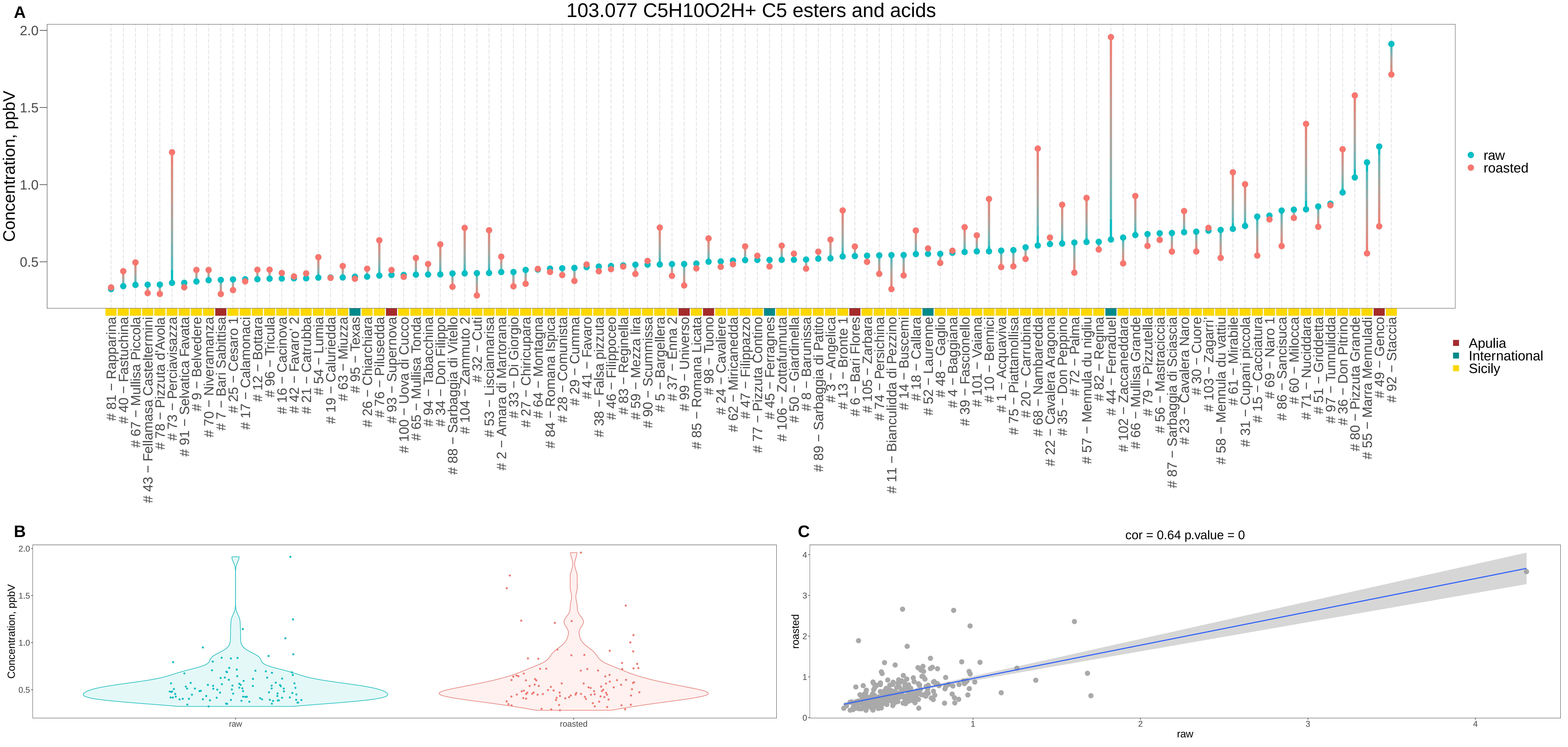

A
103.077 C5H10O2H+ C5 esters and acids
Concentration, ppbV
2.0
1.5
1.0
0.5
raw
roasted
Apulia
International
Sicily
81 – Rapparina
40 – Fastuchina
67 – Mullisa Piccola
43 – Fellamasa Casteltermini
78 – Pizzuta d'Avola
73 – Perciavisazza
91 – Selvatica Favata
9 – Belvedere
70 – Niveramanza
7 – Bari Sabitissa
25 – Cesaro 1
17 – Calamonaci
12 – Bottara
96 – Tricula
16 – Cacinova
42 – Favaro' 2
21 – Catrubba
54 – Lumia
19 – Caluriedda
63 – Miuzza
95 – Texas
26 – Chiarchiara
76 – Pilusedda
93 – Supernova
100 – Uova di Cucco
65 – Mullisa Tonda
94 – Tabacchina
34 – Don Filippo
88 – Sarbaggia di Vitello
104 – Zammuto 2
32 – Cuti
53 – Lisciannarisa
2 – Amara di Martorana
33 – Di Giorgio
27 – Chiricupara
64 – Montagna
84 – Romana Ispica
28 – Comunista
29 – Cumma
41 – Favaro
38 – Falsa pizzuta
46 – Filippoceo
83 – Reginella
59 – Mezza lira
90 – Scummissa
5 – Bargellera
37 – Enna 2
99 – Universo
85 – Romana Licata
98 – Tuono
24 – Cavaliere
62 – Miricanedda
47 – Filippazzo
77 – Pizzuta Contino
45 – Ferragnes
106 – Zottafunnuta
50 – Giardinella
8 – Barunissa
89 – Sarbaggia di Patito
3 – Angelica
13 – Bronte 1
6 – Bari Flores
105 – Zarbara
74 – Persichina
11 – Bianculidda di Pezzino
14 – Busceni
18 – Callara
52 – Laurenne
48 – Gaglio
4 – Baggiana
39 – Fascionello
101 – Vaiana
10 – Bennici
1 – Acquaviva
75 – Piattamollisa
20 – Carrubina
68 – Nambaredda
22 – Cavalera Aragona
35 – Don Peppino
72 – Palma
57 – Mennula du nigliu
82 – Regina
44 – Ferraduel
102 – Zaccaneddara
66 – Mullisa Grande
79 – Pizzutella
56 – Mastraciccia
87 – Sarbaggia di Sciascia
23 – Cavalera Naro
30 – Cuore
103 – Zagarri'
58 – Mennula du vattiu
61 – Mirabile
31 – Cupani piccola
15 – Cacciatura
69 – Naro 1
86 – Sancisuca
60 – Milocca
71 – Nuciddara
51 – Griddetta
97 – Tunnulidda
36 – Don Pitrino
80 – Pizzuta Grande
55 – Marra Mennuladi
49 – Genco
92 – Staccia
B
Concentration, ppbV
2.0
1.5
1.0
0.5
raw
roasted
C
cor = 0.64 p.value = 0
roasted
raw
4
3
2
1
0
1
2
3
4

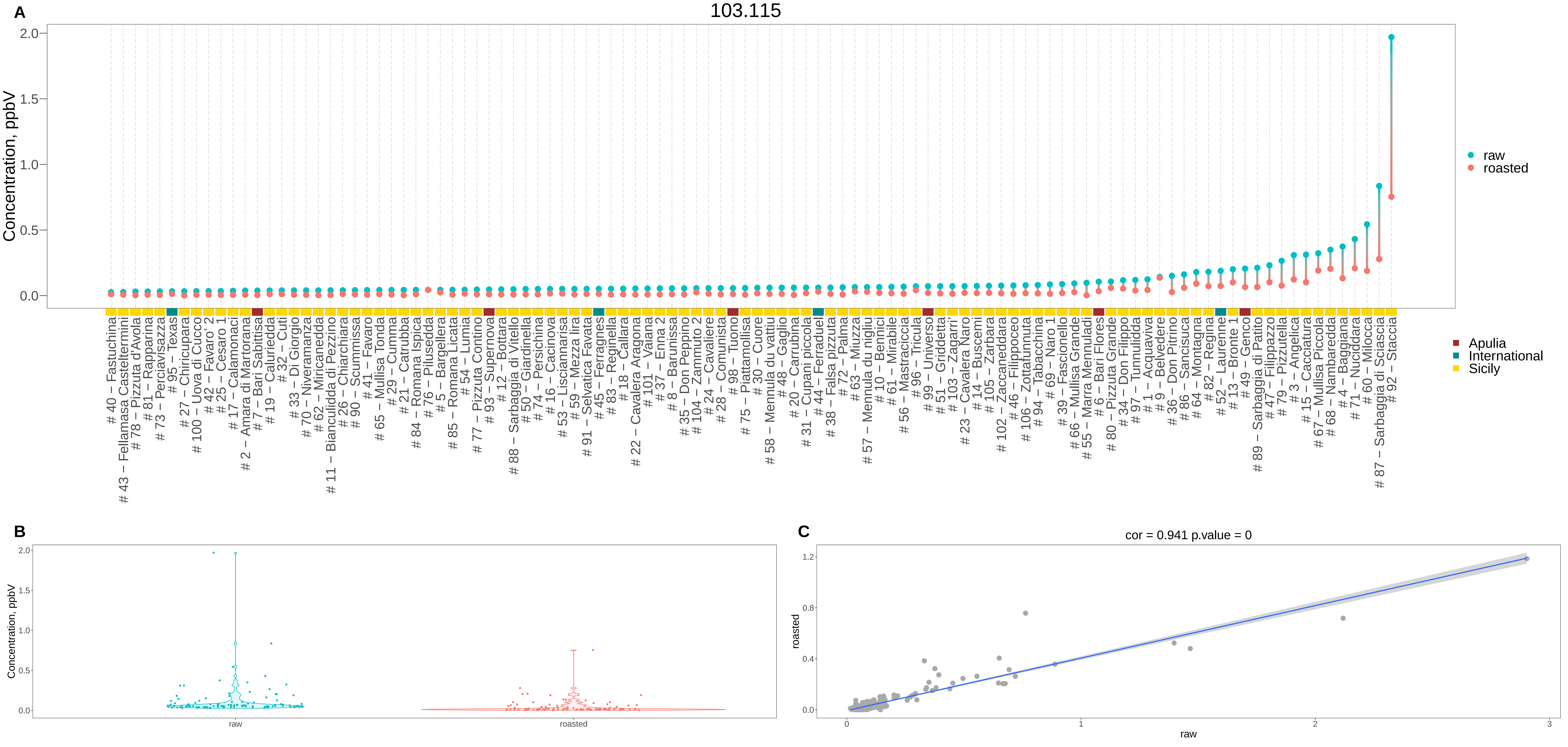

A
103.115
Concentration, ppbV
2.0
1.5
1.0
0.5
0.0
raw
roasted
Apulia
International
Sicily
40 – Fastuchina
43 – Fellamasa Casteltermini
78 – Pizzuta d'Avola
81 – Rapparina
73 – Perciavisazza
95 – Texas
27 – Chiricupara
100 – Uova di Cucco
42 – Favaro' 2
25 – Cesaro 1
17 – Calamonaci
2 – Amara di Martorana
7 – Bari Sabitissa
19 – Caluriedda
32 – Cuti
33 – Di Giorgio
70 – Niveramanza
62 – Miricanedda
11 – Bianculidda di Pezzino
26 – Chiarchiara
90 – Scummissa
41 – Favaro
65 – Mullisa Tonda
29 – Cumma
21 – Catrubba
84 – Romana Ispica
76 – Pilusedda
5 – Bargellera
85 – Romana Licata
54 – Lumia
77 – Pizzuta Contino
93 – Supernova
12 – Bottara
88 – Sarbaggia di Vitello
50 – Giardinella
74 – Persichina
16 – Cacinova
53 – Lisciannarisa
59 – Mezza lira
91 – Selvatica Favata
45 – Ferragnes
83 – Reginella
18 – Callara
22 – Cavalera Aragona
101 – Vaiana
37 – Enna 2
8 – Barunissa
35 – Don Peppino
104 – Zammuto 2
24 – Cavaliere
28 – Comunista
98 – Tuono
75 – Piattamollisa
30 – Cuore
58 – Mennula du vattiu
48 – Gaglio
20 – Carrubina
31 – Cupani piccola
44 – Ferraduel
38 – Falsa pizzuta
72 – Palma
63 – Miuzza
57 – Mennula du nigliu
10 – Bennici
61 – Mirabile
56 – Mastraciccia
96 – Tricula
99 – Universo
51 – Griddetta
103 – Zagarri'
23 – Cavalera Naro
14 – Buscemi
105 – Zarbara
102 – Zaccaneddara
46 – Filippoceo
106 – Zottafunnuta
94 – Tabacchina
69 – Naro 1
39 – Fascionello
66 – Mullisa Grande
55 – Marra Mennuladi
6 – Bari Flores
80 – Pizzuta Grande
34 – Don Filippo
97 – Tunnulidda
1 – Acquaviva
9 – Belvedere
36 – Don Pitrino
86 – Sancisuca
64 – Montagna
82 – Regina
52 – Laurenne
13 – Bronte 1
49 – Genco
89 – Sarbaggia di Patito
47 – Filippazzo
79 – Pizzutella
3 – Angelica
15 – Cacciatura
67 – Mullisa Piccola
68 – Nambaredda
4 – Baggiana
71 – Nuciddara
60 – Milocca
87 – Sarbaggia di Sciascia
92 – Staccia
B
Concentration, ppbV
2.0
1.5
1.0
0.5
0.0
raw
roasted
C
cor = 0.941 p.value = 0
roasted
1.2
0.8
0.4
0.0
0
1
2
3
raw

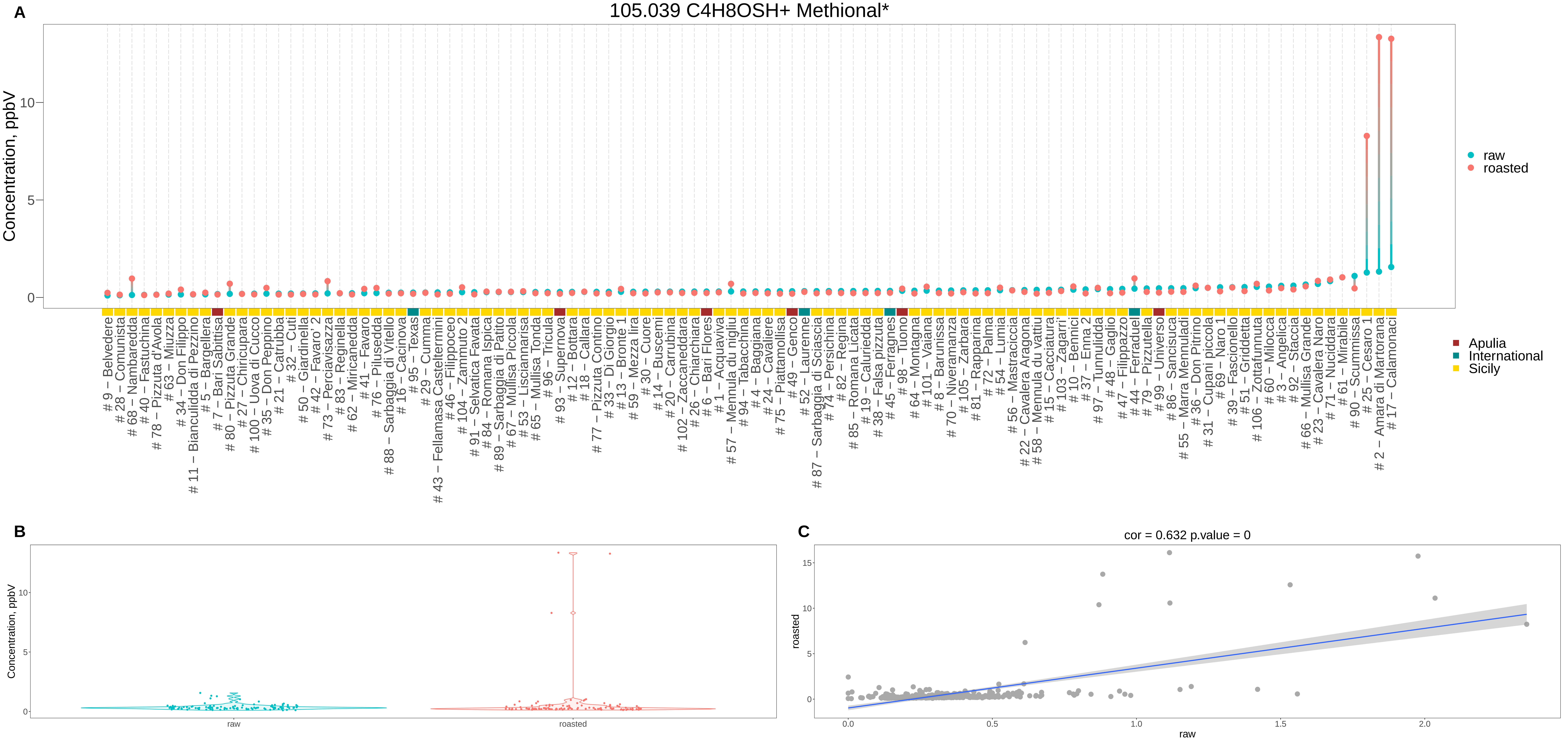
A
105.039 C4H8OSH+ Methional*
Concentration, ppbV
0
5
10
raw
roasted
Apulia
International
Sicily
9 – Belvedere
28 – Comunista
68 – Nambaredda
40 – Fastuchina
78 – Pizzuta d'Avola
63 – Miuzza
34 – Don Filippo
11 – Bianculidda di Pezzino
5 – Bargellera
7 – Bari Sabitissa
80 – Pizzuta Grande
27 – Chiricupara
100 – Uova di Cucco
35 – Don Peppino
21 – Catrubba
32 – Cuti
50 – Giardinella
42 – Favaro' 2
73 – Perciavisazza
83 – Reginella
62 – Miricanedda
41 – Favaro
76 – Pilusedda
88 – Sarbaggia di Vitello
16 – Cacinova
95 – Texas
29 – Cumma
43 – Fellamasa Casteltermini
46 – Filippoceo
104 – Zammuto 2
91 – Selvatica Favata
84 – Romana Ispica
89 – Sarbaggia di Patito
67 – Mullisa Piccola
53 – Lisciannarisa
65 – Mullisa Tonda
96 – Tricula
93 – Supernova
12 – Bottara
18 – Callara
77 – Pizzuta Contino
33 – Di Giorgio
13 – Bronte 1
59 – Mezza lira
30 – Cuore
14 – Buscemi
20 – Carrubina
102 – Zaccaneddara
26 – Chiarchiara
6 – Bari Flores
1 – Acquaviva
57 – Mennula du nigliu
94 – Tabacchina
4 – Baggiana
24 – Cavaliere
75 – Piattamollisa
49 – Genco
52 – Laurenne
87 – Sarbaggia di Sciascia
74 – Persichina
82 – Regina
85 – Romana Licata
19 – Caluriedda
38 – Falsa pizzuta
45 – Ferragnes
98 – Tuono
64 – Montagna
101 – Vaiana
8 – Barunissa
70 – Niveramanza
105 – Zarbara
81 – Rapparina
72 – Palma
54 – Lumia
56 – Mastraciccia
22 – Cavalera Aragona
58 – Mennula du vattiu
15 – Cacciatura
103 – Zagarri'
10 – Bennici
37 – Enna 2
97 – Tunnulidda
48 – Gaglio
47 – Filippazzo
44 – Ferraduel
79 – Pizzutella
99 – Universo
86 – Sancisuca
55 – Marra Mennuladi
36 – Don Pitrino
31 – Cupani piccola
69 – Naro 1
39 – Fascionello
51 – Griddetta
106 – Zottafunnuta
60 – Milocca
3 – Angelica
92 – Sfaccia
66 – Mullisa Grande
23 – Cavalera Naro
71 – Nuciddara
61 – Mirabile
90 – Scummissa
25 – Cesaro 1
2 – Amara di Martorana
17 – Calamonaci
B
Concentration, ppbV
0
5
10
raw
roasted
C
cor = 0.632 p.value = 0
roasted
0
5
10
15
0.0
0.5
1.0
1.5
2.0
raw

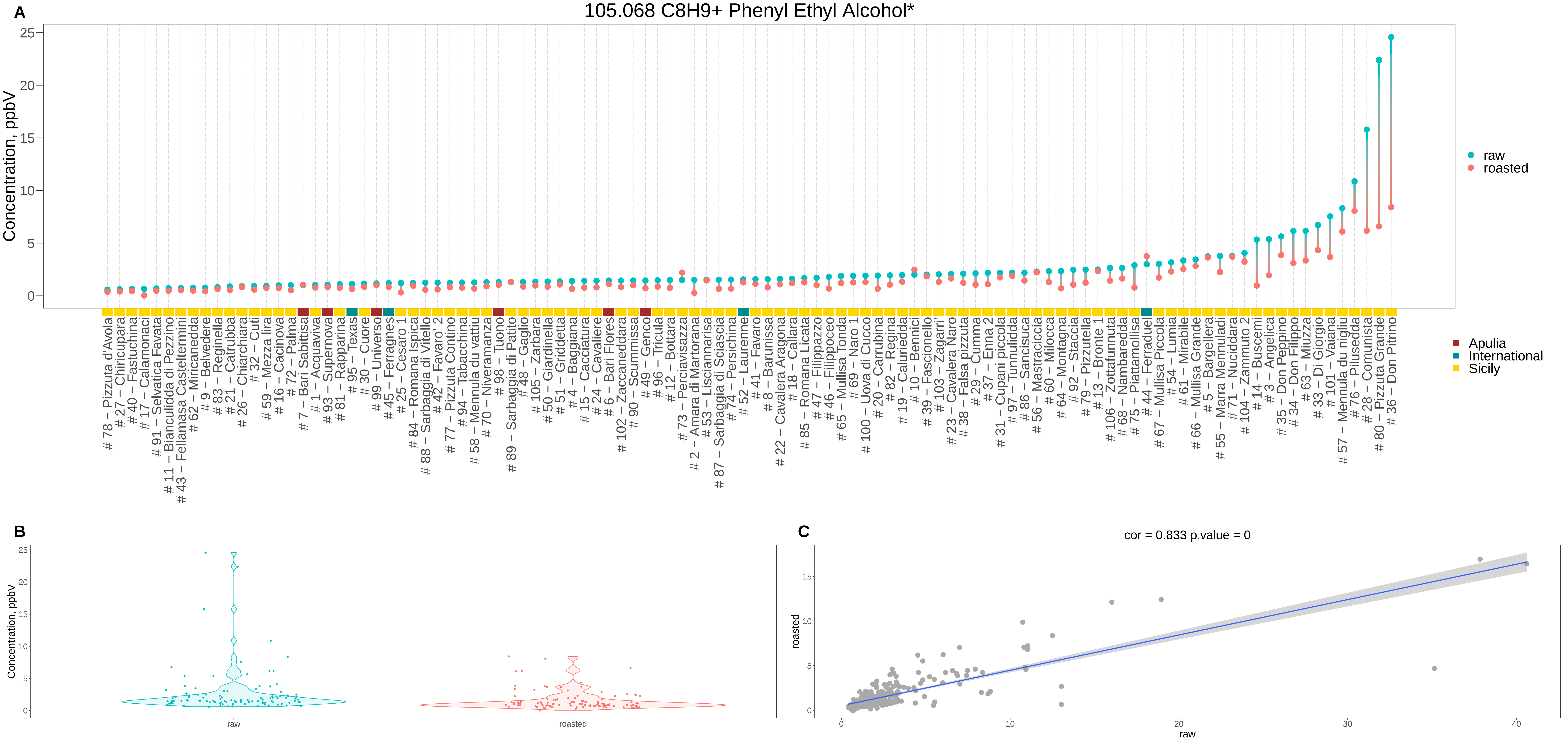
A
105.068 C8H9+ Phenyl Ethyl Alcohol*
Concentration, ppbV
0
5
10
15
20
25
raw
roasted
Apulia
International
Sicily
78 – Pizzuta d'Avola
27 – Chiricupara
40 – Fastuchina
17 – Calamonaci
91 – Selvatica Favata
11 – Bianculidda di Pezzino
43 – Fellamasa Casteltermini
62 – Miricanedda
9 – Belvedere
83 – Reginella
21 – Catrubba
26 – Chiarchiara
32 – Cuti
59 – Mezza lira
16 – Cacinova
72 – Palma
7 – Bari Sabittisa
1 – Acquaviva
93 – Supernova
81 – Rapparina
95 – Texas
30 – Cuore
99 – Universo
45 – Ferragnes
25 – Cesaro 1
84 – Romana Ispica
88 – Sarbaggia di Vitello
42 – Favaro' 2
77 – Pizzuta Contino
94 – Tabacchina
58 – Mennula du vattiu
70 – Niveramanza
98 – Tuono
89 – Sarbaggia di Patito
48 – Gaglio
105 – Zarbara
50 – Giardinella
51 – Griddetta
4 – Baggiana
15 – Cacciatura
24 – Cavaliere
6 – Bari Flores
102 – Zaccaneddara
90 – Scummissa
49 – Genco
96 – Tricula
12 – Bottara
73 – Perciavisazza
2 – Amara di Martorana
53 – Liscianarisa
87 – Sarbaggia di Sciascia
74 – Persichina
52 – Laurenne
41 – Favaro
8 – Barunissa
22 – Cavalera Aragona
18 – Callara
85 – Romana Licata
47 – Filippazzo
46 – Filippoceo
65 – Mullisa Tonda
69 – Naro 1
100 – Uova di Cucco
20 – Carrubina
82 – Regina
19 – Caluriedda
10 – Bennici
39 – Fascionello
103 – Zagarri'
23 – Cavalera Naro
38 – Falsa pizzuta
29 – Cumma
37 – Enna 2
31 – Cupani piccola
97 – Tunnulidda
86 – Sancisuca
56 – Mastracicia
60 – Milocca
64 – Montagna
92 – Staccia
79 – Pizzutella
13 – Bronte 1
106 – Zottafunnuta
68 – Nambaredda
75 – Piattamollisa
44 – Ferraduel
67 – Mullisa Piccola
54 – Lumia
61 – Mirabile
66 – Mullisa Grande
5 – Bargellera
55 – Marra Mennuladi
71 – Nuciddara
104 – Zammuto 2
14 – Buscemi
3 – Angelica
35 – Don Peppino
34 – Don Filippo
63 – Miuzza
33 – Di Giorgio
101 – Vaiana
57 – Mennula du nigliu
76 – Pilusedda
28 – Comunista
80 – Pizzuta Grande
36 – Don Pitrino
B
Concentration, ppbV
raw
roasted
C
cor = 0.833 p.value = 0
roasted
raw
0
10
20
30
40

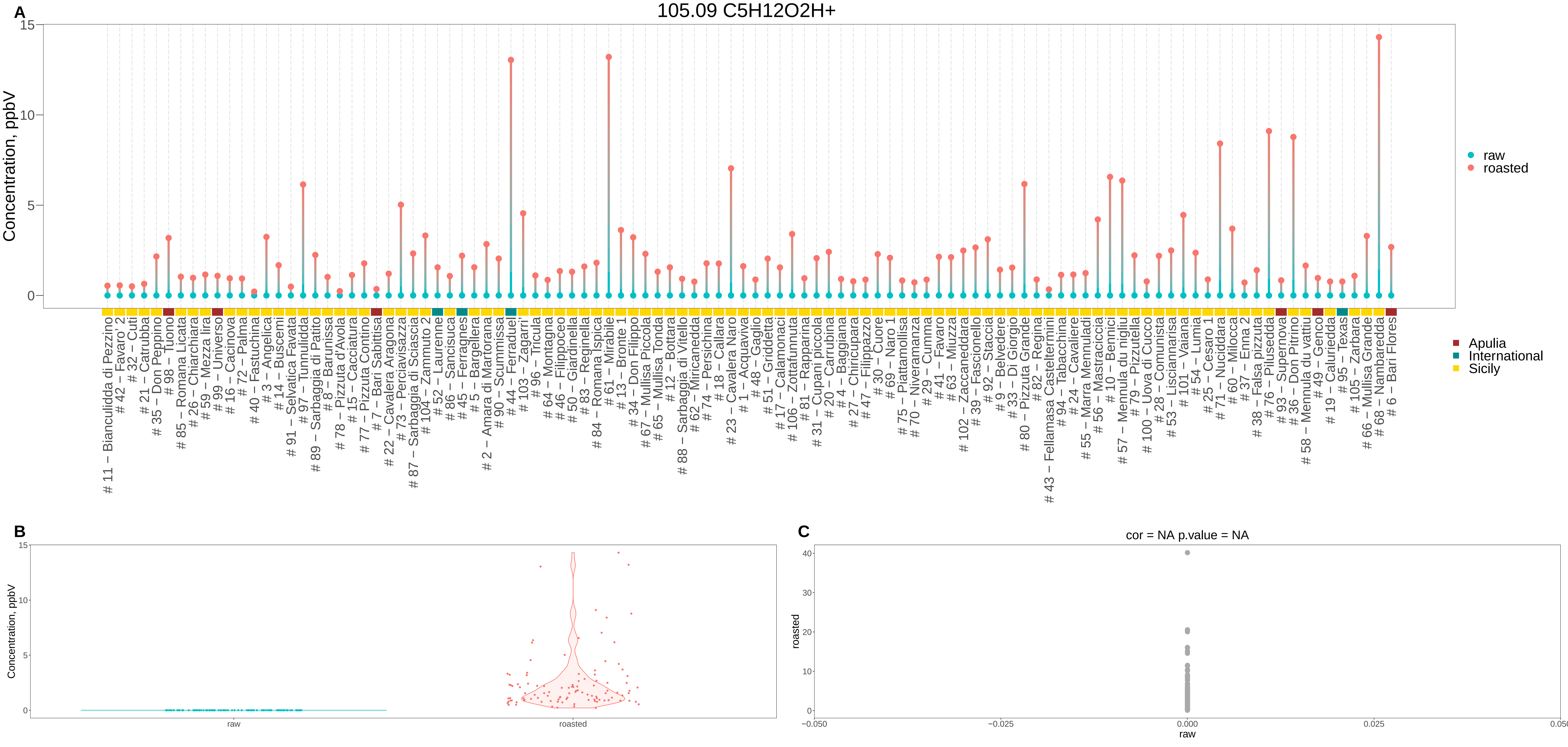
A
105.09 C5H12O2H+
Concentration, ppbV
0
5
10
15
raw
roasted
Apulia
International
Sicily
11 – Bianculidda di Pezzino
42 – Favaro' 2
32 – Cuti
21 – Catrubba
35 – Don Peppino
98 – Tuono
85 – Romana Licata
26 – Chiarchiara
59 – Mezza lira
99 – Universo
16 – Cacinova
72 – Palma
40 – Fastuchina
3 – Angelica
14 – Buscemi
91 – Selvatica Favata
97 – Tunnulidda
89 – Sarbaggia di Patito
8 – Barunissa
78 – Pizzuta d'Avola
15 – Cacciatura
77 – Pizzuta Contino
7 – Bari Sabitisa
22 – Cavalera Aragona
73 – Perciavisazza
87 – Sarbaggia di Sciascia
104 – Zammuto 2
52 – Laurenne
86 – Sancisuca
45 – Ferragnes
5 – Bargellera
2 – Amara di Martorana
90 – Scummissa
44 – Ferraduel
103 – Zagarri'
96 – Tricula
64 – Montagna
46 – Filippoceo
50 – Giardinella
83 – Reginella
84 – Romana Ispica
61 – Mirabile
13 – Bronte 1
34 – Don Filippo
67 – Mullisa Piccola
65 – Mullisa Tonda
12 – Bottara
88 – Sarbaggia di Vitello
62 – Miricanedda
74 – Persichina
18 – Callara
23 – Cavalera Naro
1 – Acquaviva
48 – Gaglio
51 – Griddetta
17 – Calamonaci
106 – Zottafunnuta
81 – Rapparina
31 – Cupani piccola
20 – Carrubina
4 – Baggiana
27 – Chiricupara
47 – Filippazzo
30 – Cuore
69 – Naro 1
75 – Piattamollisa
70 – Niveramanza
29 – Cumma
41 – Favaro
63 – Miuzza
102 – Zaccaneddara
39 – Fascionello
92 – Staccia
9 – Belvedere
33 – Di Giorgio
80 – Pizzuta Grande
82 – Regina
43 – Fellamasa Casteltermini
94 – Tabacchina
24 – Cavaliere
55 – Marra Mennuladi
56 – Mastracicia
10 – Bennici
57 – Mennula du nigliu
79 – Pizzutella
100 – Uova di Cucco
28 – Comunista
53 – Lisciannarisa
101 – Vaiana
54 – Lumia
25 – Cesaro 1
71 – Nuciddara
60 – Milocca
37 – Enna 2
38 – Falsa pizzuta
76 – Pilusedda
93 – Supernova
36 – Don Pitrino
58 – Mennula du vattiu
49 – Genco
19 – Calurieddu
95 – Texas
105 – Zarbara
66 – Mullisa Grande
68 – Nambaredda
6 – Bari Flores
B
Concentration, ppbV
0
5
10
15
raw
roasted
C
cor = NA p.value = NA
roasted
0
10
20
30
40
−0.050
−0.025
0.000
0.025
0.050
raw

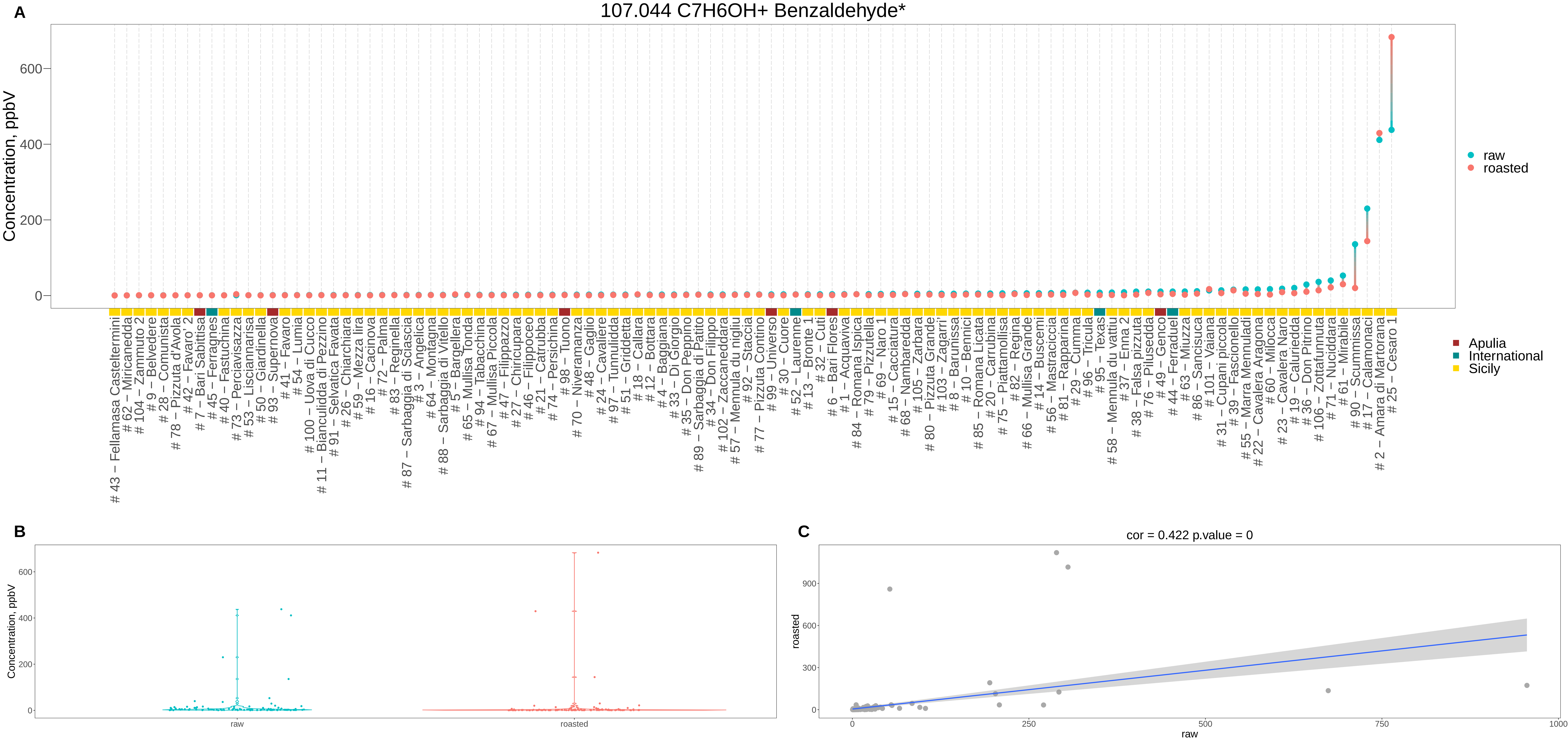
A
107.044 C7H6OH+ Benzaldehyde*
Concentration, ppbV
0
200
400
600
raw
roasted
Apulia
International
Sicily
43 – Fellamasa Casteltermini
62 – Miricanedda
104 – Zammuto 2
9 – Belvedere
28 – Comunista
78 – Pizzuta d'Avola
42 – Favaro' 2
7 – Bari Sabittisa
45 – Ferragnes
40 – Fastuchina
73 – Perciavisazza
53 – Lisciannarisa
50 – Giardinella
93 – Supernova
41 – Favaro
54 – Lumia
100 – Uova di Cucco
11 – Biancuiidda di Pezzino
91 – Selvatica Favata
26 – Chiarchiara
59 – Mezza lira
16 – Cacinova
72 – Palma
83 – Reginella
87 – Sarbaggia di Sciascia
3 – Angelica
64 – Montagna
88 – Sarbaggia di Vitello
5 – Bargellera
65 – Mullisa Tonda
94 – Tabacchina
67 – Mullisa Piccola
47 – Filippazzo
27 – Chiricupara
46 – Filippoceo
21 – Catrubba
74 – Persichina
98 – Tuono
70 – Niveramanza
48 – Gaglio
24 – Cavaliere
97 – Tunnulidda
51 – Griddetta
18 – Callara
12 – Bottara
4 – Baggiana
33 – Di Giorgio
35 – Don Peppino
89 – Sarbaggia di Patito
34 – Don Filippo
102 – Zaccaneddara
57 – Mennula du nigliu
92 – Staccia
77 – Pizzuta Contino
99 – Universo
30 – Cuore
52 – Laurenne
13 – Bronte 1
32 – Cuti
6 – Bari Flores
1 – Acquaviva
84 – Romana Ispica
79 – Pizzutella
69 – Naro 1
15 – Cacciatura
68 – Nambaredda
105 – Zarbara
80 – Pizzuta Grande
103 – Zagarri'
8 – Barunissa
10 – Bennici
85 – Romana Licata
20 – Carrubina
75 – Piattamollisa
82 – Regina
66 – Mullisa Grande
14 – Buscemi
56 – Mastraciccia
81 – Rapparina
29 – Cumma
96 – Tricula
95 – Texas
58 – Mennula du vattiu
37 – Enna 2
38 – Falsa pizzuta
76 – Pilusedda
49 – Genco
44 – Ferraduel
63 – Miuzza
86 – Sancisuca
101 – Vaiana
31 – Cupani piccola
39 – Fascionello
55 – Marra Mennuladi
22 – Cavalera Aragona
60 – Milocca
23 – Cavalera Naro
19 – Caluriedda
36 – Don Pitrino
106 – Zottafunnuta
71 – Nuciddara
61 – Mirabile
90 – Scummissa
17 – Calamonaci
2 – Amara di Martorana
25 – Cesaro 1
B
Concentration, ppbV
0
200
400
600
raw
roasted
C
cor = 0.422 p.value = 0
roasted
0
300
600
900
0
250
500
750
1000
raw

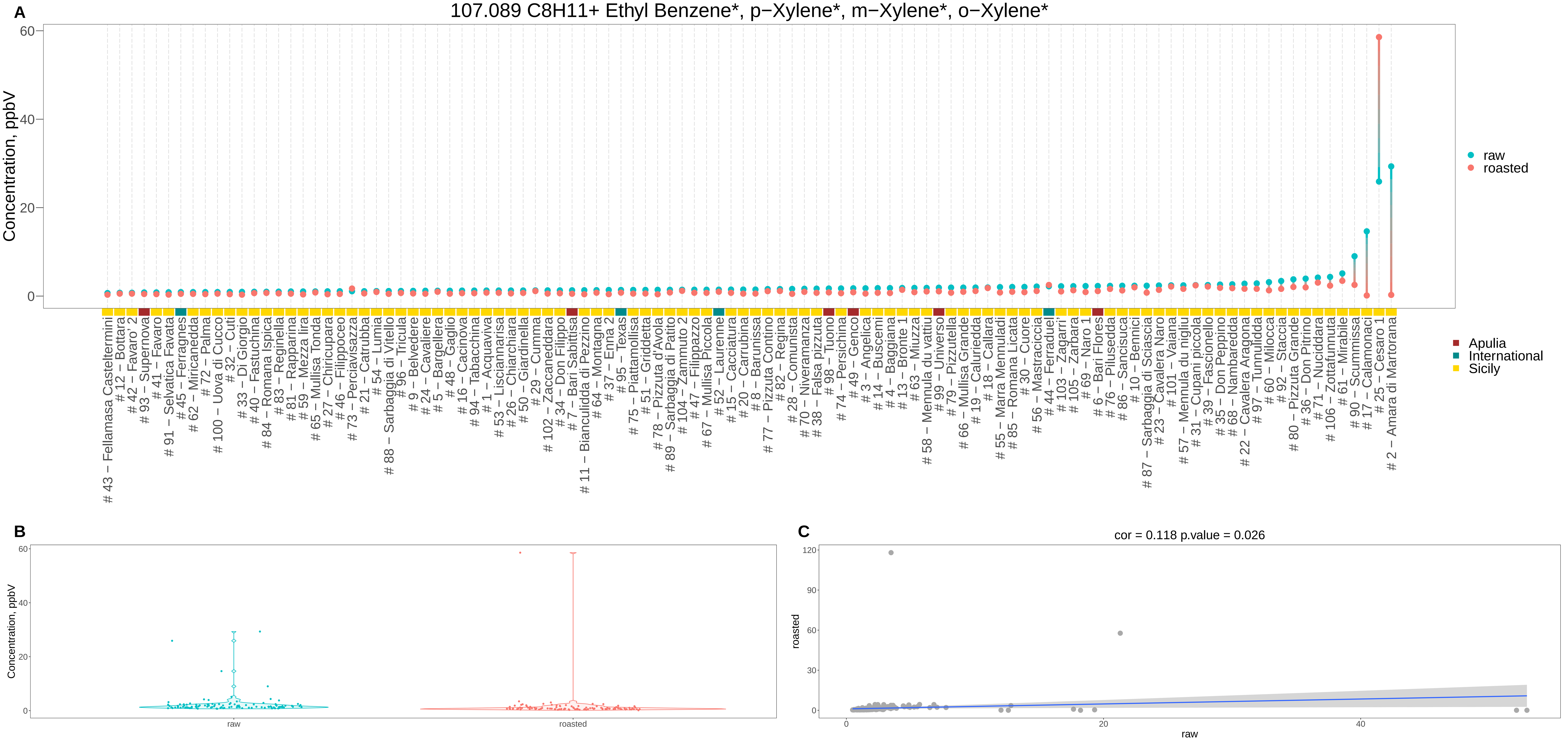
A
107.089 C8H11+ Ethyl Benzene*, p−Xylene*, m−Xylene*, o−Xylene*
Concentration, ppbV
0
20
40
60
raw
roasted
Apulia
International
Sicily
43 – Fellamasa Casteltermini
12 – Bottara
42 – Favaro' 2
93 – Supernova
41 – Favaro
91 – Selvatica Favata
45 – Ferragnes
62 – Miricanedda
72 – Palma
100 – Uova di Cucco
32 – Cuti
33 – Di Giorgio
40 – Fastuchina
84 – Romana Ispica
83 – Reginella
81 – Rapparina
59 – Mezza lira
65 – Mullisa Tonda
27 – Chiricupara
46 – Filippoceo
73 – Perciavisazza
21 – Catrubba
54 – Lumia
88 – Sarbaggia di Vitello
96 – Tricula
9 – Belvedere
24 – Cavaliere
5 – Bargellera
48 – Gaglio
16 – Cacinova
94 – Tabacchina
1 – Acquaviva
53 – Lisciannarisa
26 – Chiarchiara
50 – Giardinella
29 – Cumma
102 – Zaccaneddara
34 – Don Filippo
7 – Bari Sabittisa
11 – Bianculidda di Pezzino
64 – Montagna
37 – Enna 2
95 – Texas
75 – Piattamollisa
51 – Griddetta
78 – Pizzuta d'Avola
89 – Sarbaggia di Patito
104 – Zammuto 2
47 – Filippazzo
67 – Mullisa Piccola
52 – Laurenne
15 – Cacciatura
20 – Carrubina
8 – Barunissa
77 – Pizzuta Contino
82 – Regina
28 – Comunista
70 – Niveramanza
38 – Falsa pizzuta
98 – Tuono
74 – Persichina
49 – Genco
3 – Angelica
14 – Buscemi
4 – Baggiana
13 – Bronte 1
63 – Miuzza
58 – Mennula du vattiu
99 – Universo
79 – Pizzutella
66 – Mullisa Grande
19 – Caluriedda
18 – Callara
55 – Marra Mennuladi
85 – Romana Licata
30 – Cuore
56 – Mastracicia
44 – Ferraduel
103 – Zagarri'
105 – Zarbara
69 – Naro 1
6 – Bari Flores
76 – Pilusedda
86 – Sancisuca
10 – Bennici
87 – Sarbaggia di Sciascia
23 – Cavalera Naro
101 – Vaiana
57 – Mennula du nigliu
31 – Cupani piccola
39 – Fascionello
35 – Don Peppino
68 – Nambaredda
22 – Cavalera Aragona
97 – Tunnulidda
60 – Milocca
92 – Staccia
80 – Pizzuta Grande
36 – Don Pitrino
71 – Nuciddara
106 – Zottafunnuta
61 – Mirabile
90 – Scummissa
17 – Calamonaci
25 – Cesaro 1
2 – Amara di Martorana
B
Concentration, ppbV
0
20
40
60
raw
roasted
C
cor = 0.118 p.value = 0.026
roasted
0
30
60
90
120
raw
0
20
40

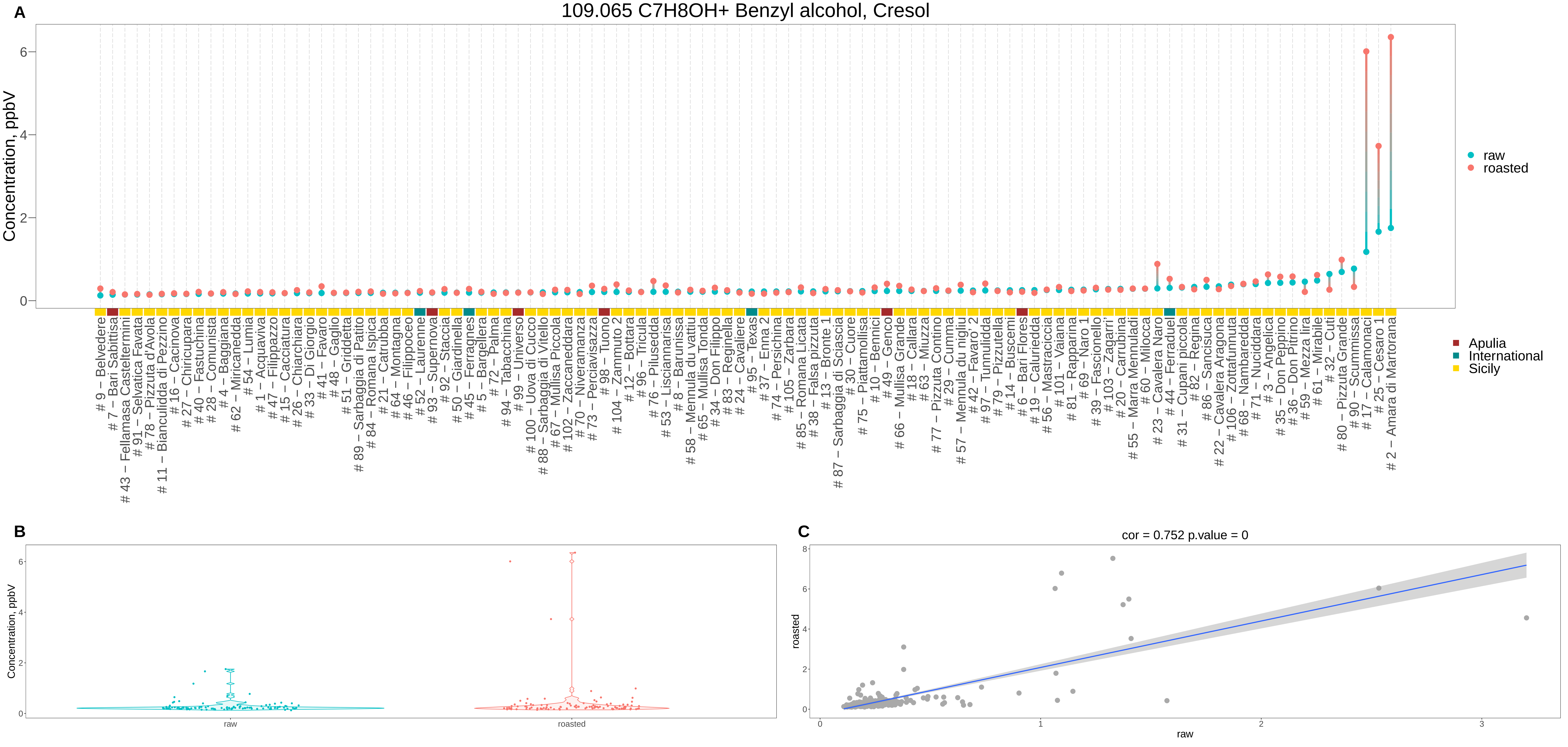
A
109.065 C7H8OH+ Benzyl alcohol, Cresol
Concentration, ppbV
0
2
4
6
raw
roasted
Apulia
International
Sicily
9 – Belvedere
7 – Bari Sabitissa
43 – Fellamasa Casteltermini
91 – Selvatica Favata
78 – Pizzuta d'Avola
11 – Biancolidda di Pezzino
16 – Cacinova
27 – Chiricupara
40 – Fastuchina
28 – Comunista
4 – Baggiana
62 – Miricanedda
54 – Lumia
1 – Acquaviva
47 – Filippazzo
15 – Cacciatura
26 – Chiarchiara
33 – Di Giorgio
41 – Favaro
48 – Gaglio
51 – Griddetta
89 – Sarbaggia di Patito
84 – Romana Ispica
21 – Catrubba
64 – Montagna
46 – Filippoceo
52 – Laurenne
93 – Supernova
92 – Staccia
50 – Giardinella
45 – Ferragnes
5 – Bargellera
72 – Palma
94 – Tabacchina
99 – Universo
100 – Uova di Cucco
88 – Sarbaggia di Vitello
67 – Mullisa Piccola
102 – Zaccaneddara
70 – Niveramanza
73 – Perciavisazza
98 – Tuono
104 – Zammuto 2
12 – Bottara
96 – Tricula
76 – Pilusedda
53 – Lisciannarisa
8 – Barunissa
58 – Mennula du vattiu
65 – Mullisa Tonda
34 – Don Filippo
83 – Reginella
24 – Cavaliere
95 – Texas
37 – Enna 2
74 – Persichina
105 – Zarbara
85 – Romana Licata
38 – Falsa pizzuta
13 – Bronte 1
87 – Sarbaggia di Sciascia
30 – Cuore
75 – Piattamollisa
10 – Bennici
49 – Genco
66 – Mullisa Grande
18 – Callara
63 – Miuzza
77 – Pizzuta Contino
29 – Cumma
57 – Mennula du nigliu
42 – Favaro 2
97 – Tunnulidda
79 – Pizzutella
14 – Buscemi
6 – Bari Flores
19 – Caluriedda
56 – Mastracicia
101 – Vaiana
81 – Rapparina
69 – Naro 1
39 – Fascionello
103 – Zagarri
20 – Carrubina
55 – Marra Mennuladi
60 – Milocca
23 – Cavalera Naro
44 – Ferraduel
31 – Cupani piccola
82 – Regina
86 – Sancisuca
22 – Cavalera Aragona
106 – Zottafunnuta
68 – Nambaredda
71 – Nuciddara
3 – Angelica
35 – Don Peppino
36 – Don Pitrino
59 – Mezza lira
61 – Mirabile
32 – Cuti
80 – Pizzuta Grande
90 – Scummissa
17 – Calamonaci
25 – Cesaro 1
2 – Amara di Martorana
B
Concentration, ppbV
0
2
4
6
raw
roasted
C
cor = 0.752 p.value = 0
roasted
0
2
4
6
8
raw
0
1
2
3

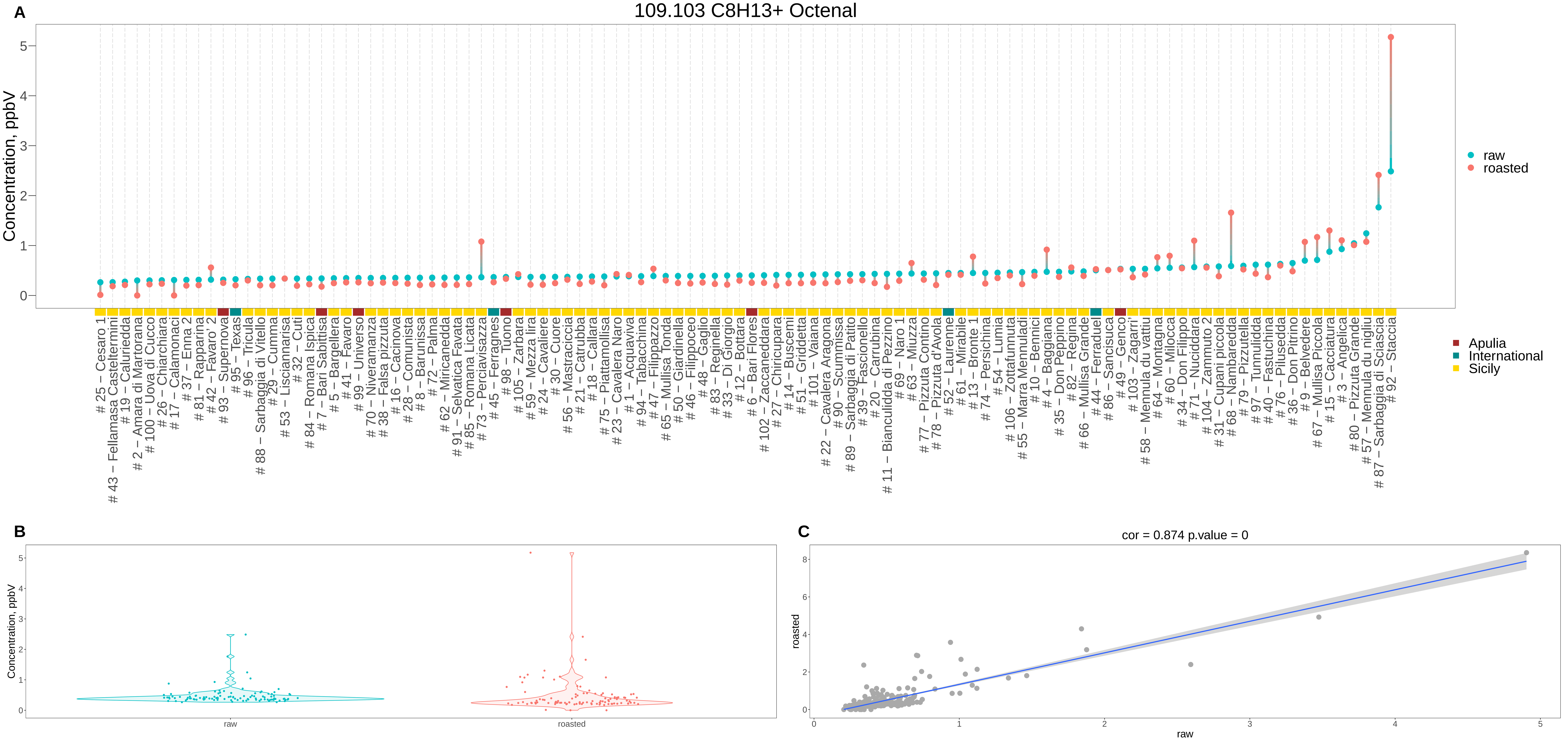
A
109.103 C8H13+ Octenal
Concentration, ppbV
0
1
2
3
4
5
raw
roasted
Apulia
International
Sicily
25 – Cesaro 1
43 – Fellamasa Casteltermini
19 – Caluriedda
2 – Amara di Martorana
100 – Uova di Cucco
26 – Chiarchiara
17 – Calamonaci
37 – Enna 2
81 – Rapparina
42 – Favaro' 2
93 – Supernova
95 – Texas
96 – Tricula
88 – Sarbaggia di Vitello
29 – Cumma
53 – Liscinnarisa
32 – Cuti
84 – Romana Ispica
7 – Bari Sabittisa
5 – Bargellera
41 – Favaro
99 – Universo
70 – Niveramanza
38 – Falsa pizzuta
16 – Cacinova
28 – Comunista
8 – Barunissa
72 – Palma
62 – Miricanedda
91 – Selvatica Favata
85 – Romana Licata
73 – Perciavisazza
45 – Ferragnes
98 – Tuono
105 – Zarbara
59 – Mezza lira
24 – Cavaliere
30 – Cuore
56 – Mastraciccia
21 – Catrubba
18 – Callara
75 – Piattamollisa
23 – Cavalera Naro
1 – Acquaviva
94 – Tabacchina
47 – Filippazzo
65 – Mullisa Tonda
50 – Giardinella
46 – Filippoceo
48 – Gaglio
83 – Reginella
33 – Di Giorgio
12 – Bottara
6 – Bari Flores
102 – Zaccaneddara
27 – Chiricupara
14 – Buscemi
51 – Griddetta
101 – Vaiana
22 – Cavalera Aragona
90 – Scummissa
89 – Sarbaggia di Patito
39 – Fascionello
20 – Carrubina
11 – Bianculidda di Pezzino
69 – Naro 1
63 – Miuzza
77 – Pizzuta Contino
78 – Pizzuta d'Avola
52 – Laurenne
61 – Mirabile
13 – Bronte 1
74 – Persichina
54 – Lumia
106 – Zottafunnuta
55 – Marra Mennuladi
10 – Bennici
4 – Baggiana
35 – Don Peppino
82 – Regina
66 – Mullisa Grande
44 – Ferraduel
86 – Sancisuca
49 – Genco
103 – Zagarri'
58 – Mennula du vattiu
64 – Montagna
60 – Milocca
34 – Don Filippo
71 – Nuciddara
104 – Zammuto 2
31 – Cupani piccola
68 – Nambaredda
79 – Pizzutella
97 – Tunnulidda
40 – Fastuchina
76 – Pilusedda
36 – Don Pitrino
9 – Belvedere
67 – Mullisa Piccola
15 – Cacciatura
3 – Angelica
80 – Pizzuta Grande
57 – Mennula du nigliu
87 – Sarbaggia di Sciascia
92 – Staccia
B
Concentration, ppbV
0
1
2
3
4
5
raw
roasted
C
cor = 0.874 p.value = 0
roasted
0
2
4
6
8
raw
0
1
2
3
4
5

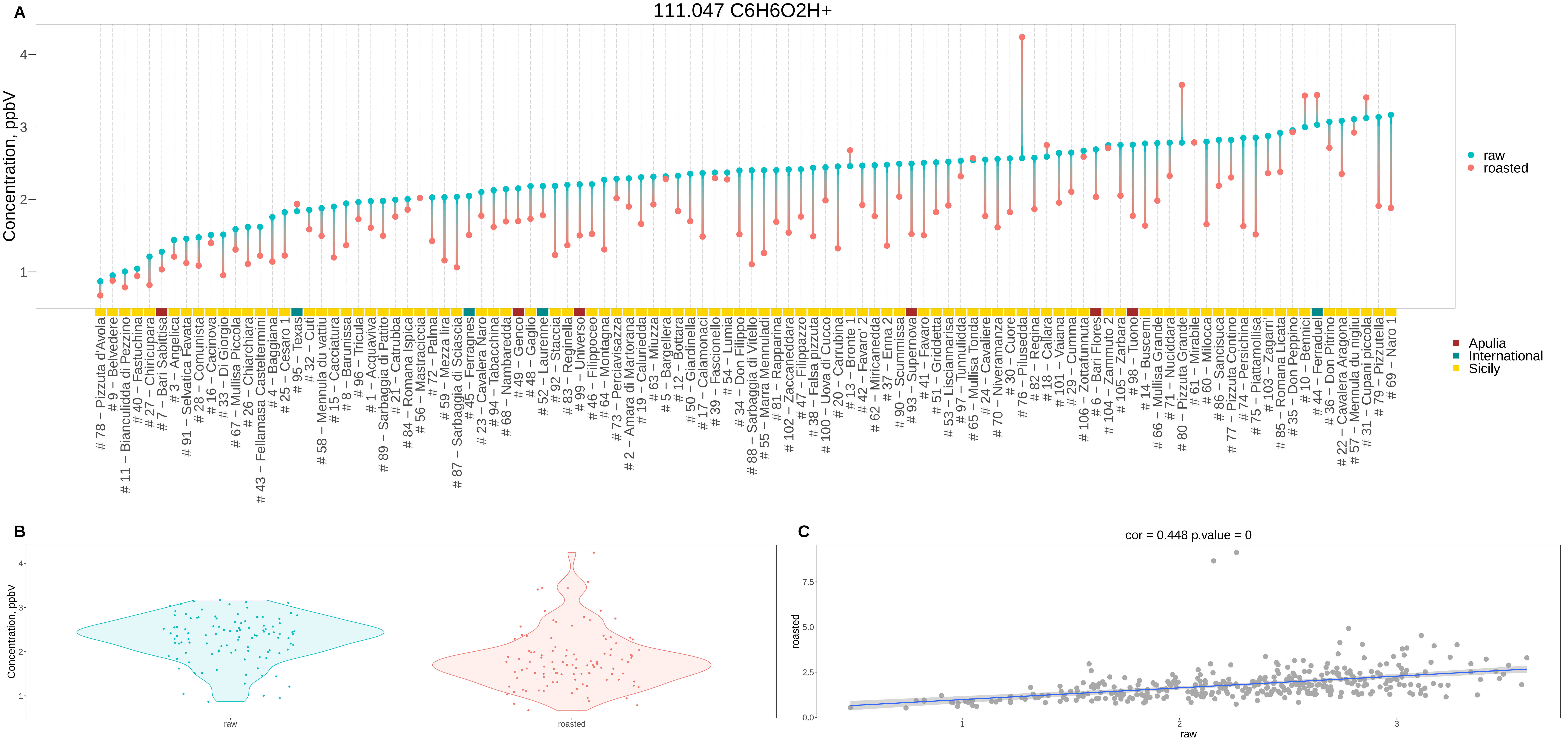
A
111.047 C6H6O2H+
Concentration, ppbV
raw
roasted
Apulia
International
Sicily
B
Concentration, ppbV
raw
roasted
C
cor = 0.448 p.value = 0
roasted
raw

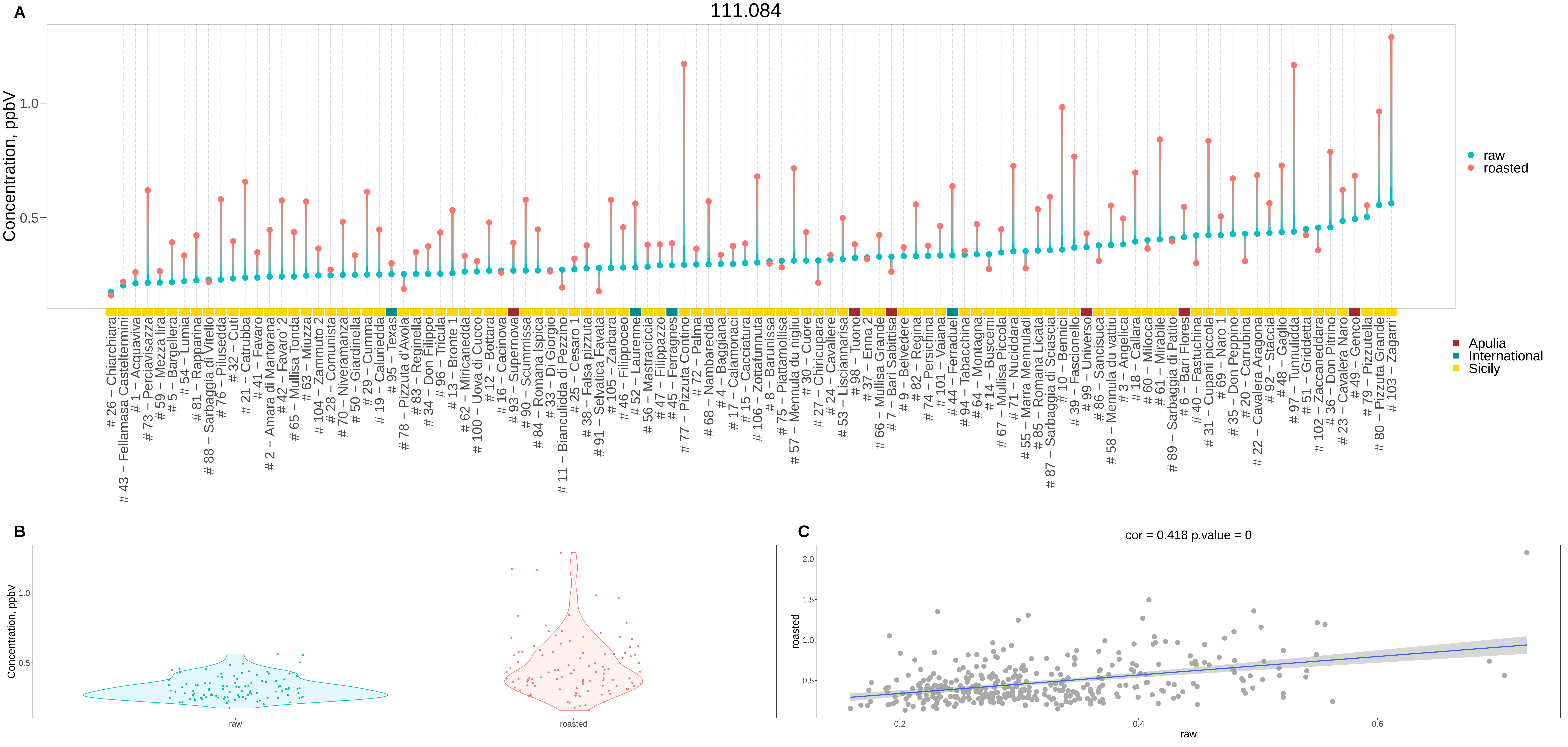
A
111.084
Concentration, ppbV
1.0
0.5
raw
roasted
Apulia
International
Sicily
26 – Chiarchiara
43 – Fellamasa Castelltermini
1 – Acquaviva
73 – Perciavisazza
59 – Mezza lira
5 – Bargellera
54 – Lumia
81 – Rapparina
88 – Sarbaggia di Vitello
76 – Piluseddа
32 – Cuti
21 – Catrubba
41 – Favaro
2 – Amara di Martorana
42 – Favaro' 2
65 – Mullisa Tonda
63 – Miuzza
104 – Zammuto 2
28 – Comunista
70 – Niveramanza
50 – Giardinella
29 – Cumma
19 – Caluriedda
95 – Texas
78 – Pizzuta d'Avola
83 – Reginella
34 – Don Filippo
96 – Tricula
13 – Bronte 1
62 – Miricanedda
100 – Uova di Cucco
12 – Bottara
16 – Cacinova
93 – Supernova
90 – Scummissa
84 – Romana Ispica
33 – Di Giorgio
11 – Biancuiidda di Pezzino
25 – Cesaro 1
38 – Falsa pizzuta
91 – Selvatica Favata
105 – Zarbara
46 – Filippoceo
52 – Laurenne
56 – Mastracicccia
47 – Filippazzo
45 – Ferragnes
77 – Pizzuta Contino
72 – Palma
68 – Nambaredda
4 – Baggiana
17 – Calamonaci
15 – Cacciatura
106 – Zottafunnuta
8 – Barunissa
75 – Piattamollisa
57 – Mennula du nigliu
30 – Cuore
27 – Chiricupara
24 – Cavaliere
53 – Lisciannarisa
98 – Tuono
37 – Enna 2
66 – Mullisa Grande
7 – Bari Sabittisa
9 – Belvedere
82 – Regina
74 – Persichina
101 – Vaiana
44 – Ferraduel
94 – Tabacchina
64 – Montagna
14 – Buscemi
67 – Mullisa Piccola
71 – Nuciddara
55 – Marra Mennuladi
85 – Romana Licata
87 – Sarbaggia di Sciascia
10 – Bennici
39 – Fascionello
99 – Universo
86 – Sancisuca
58 – Mennula du vattiu
3 – Angelica
18 – Callara
60 – Milocca
61 – Mirabile
89 – Sarbaggia di Patito
6 – Bari Flores
40 – Fastuchina
31 – Cupani piccola
69 – Naro 1
35 – Don Peppino
20 – Carrubina
22 – Cavalera Aragona
92 – Staccia
48 – Gaglio
97 – Tunnulidda
51 – Griddetta
102 – Zaccaneddara
36 – Don Pitrino
23 – Cavalera Naro
49 – Genco
79 – Pizzutella
80 – Pizzuta Grande
103 – Zagarri'
B
Concentration, ppbV
1.0
0.5
raw
roasted
C
cor = 0.418 p.value = 0
roasted
2.0
1.5
1.0
0.5
0.2
0.4
0.6
raw

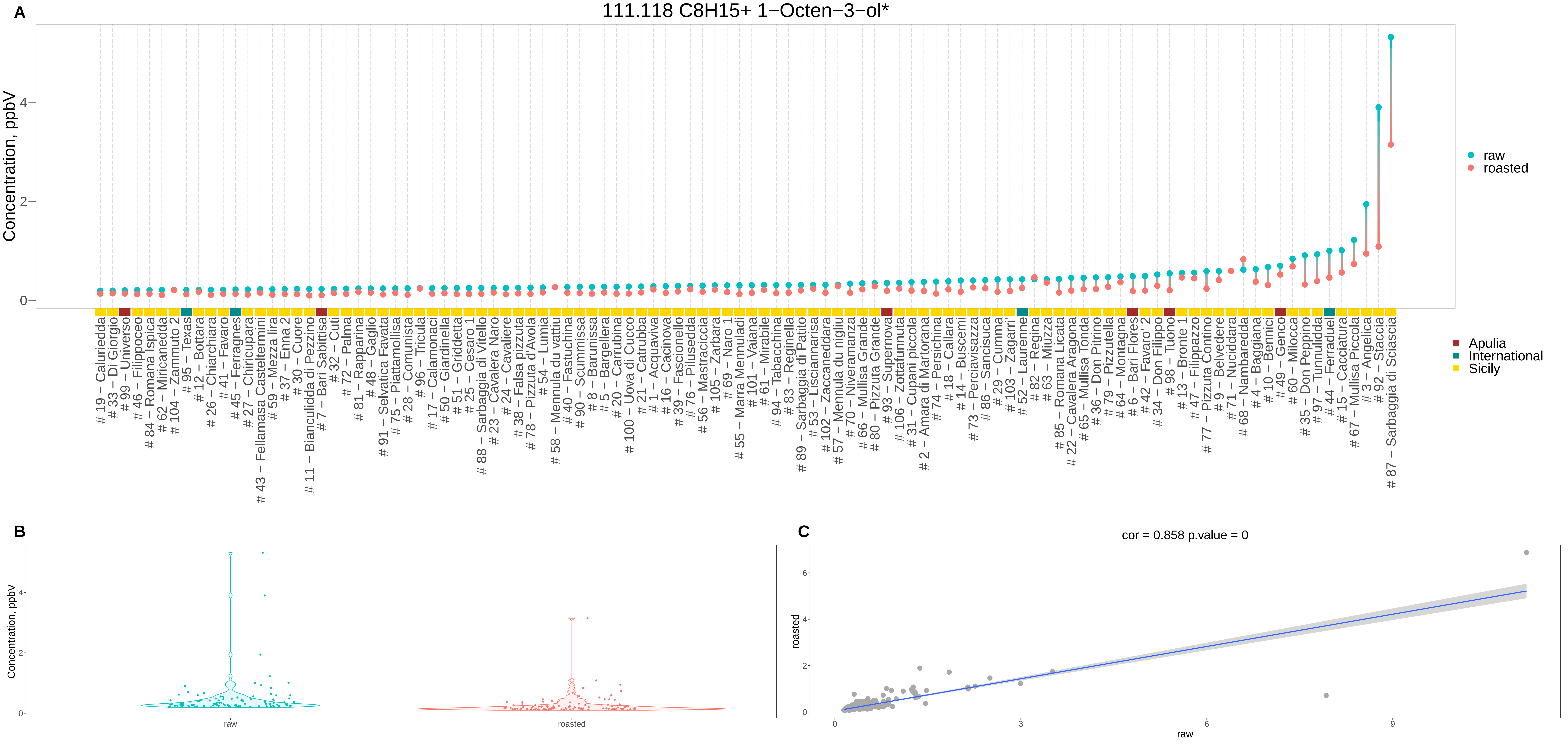

A
111.118 C8H15+ 1−Octen−3−ol*
Concentration, ppbV
0
2
4
raw
roasted
Apulia
International
Sicily
19 – Caluriedda
33 – Di Giorgio
99 – Universo
46 – Filippoceo
84 – Romana Ispica
62 – Miricanedda
104 – Zammuto 2
95 – Texas
12 – Bottara
26 – Chiarchiara
41 – Favaro
45 – Ferragnes
27 – Chiricupara
43 – Fellamasa Casteltermini
59 – Mezza lira
37 – Enna 2
30 – Cuore
11 – Bianculidda di Pezzino
7 – Bari Sabittisa
32 – Cuti
72 – Palma
81 – Rapparina
48 – Gaglio
91 – Selvatica Favata
75 – Piattamollisa
28 – Comunista
96 – Tricula
17 – Calamonaci
50 – Giardinella
51 – Griddetta
25 – Cesaro 1
88 – Sarbaggia di Vitello
23 – Cavalera Naro
24 – Cavaliere
38 – Falsa pizzuta
78 – Pizzuta d'Avola
54 – Lumia
58 – Mennula du vattiu
40 – Fastuchina
90 – Scummissa
8 – Barunissa
5 – Bargellera
20 – Carrubina
100 – Uova di Cucco
21 – Catrubba
1 – Acquaviva
16 – Cacinova
39 – Fascionello
76 – Pilusedda
56 – Mastraciccia
105 – Zarbara
69 – Naro 1
55 – Marra Mennuladi
101 – Vaiana
61 – Mirabile
94 – Tabacchina
83 – Reginella
89 – Sarbaggia di Patito
53 – Liscianarisa
102 – Zaccaneddara
57 – Mennula du nigliu
70 – Niveramanza
66 – Mullisa Grande
80 – Pizzuta Grande
93 – Supernova
106 – Zottafunnuta
31 – Cupani piccola
2 – Amara di Martorana
74 – Persichina
18 – Callara
14 – Buscemi
73 – Perciavisazza
86 – Sancisuca
29 – Cumma
103 – Zagarri'
52 – Laurenne
82 – Regina
63 – Miuzza
85 – Romana Licata
22 – Cavalera Aragona
65 – Mullisa Tonda
36 – Don Pitrino
79 – Pizzutella
64 – Montagna
6 – Bari Flores
42 – Favaro' 2
34 – Don Filippo
98 – Tuono
13 – Bronte 1
47 – Filippazzo
77 – Pizzuta Contino
9 – Belvedere
71 – Nuciddara
68 – Nambaredda
4 – Baggiana
10 – Bennici
49 – Genco
60 – Milocca
35 – Don Peppino
97 – Tunnulidda
44 – Ferraduel
15 – Cacciatura
67 – Mullisa Piccola
3 – Angelica
92 – Sfaccia
87 – Sarbaggia di Sciascia
B
Concentration, ppbV
raw
roasted
C
cor = 0.858 p.value = 0
roasted
raw
0
3
6
9

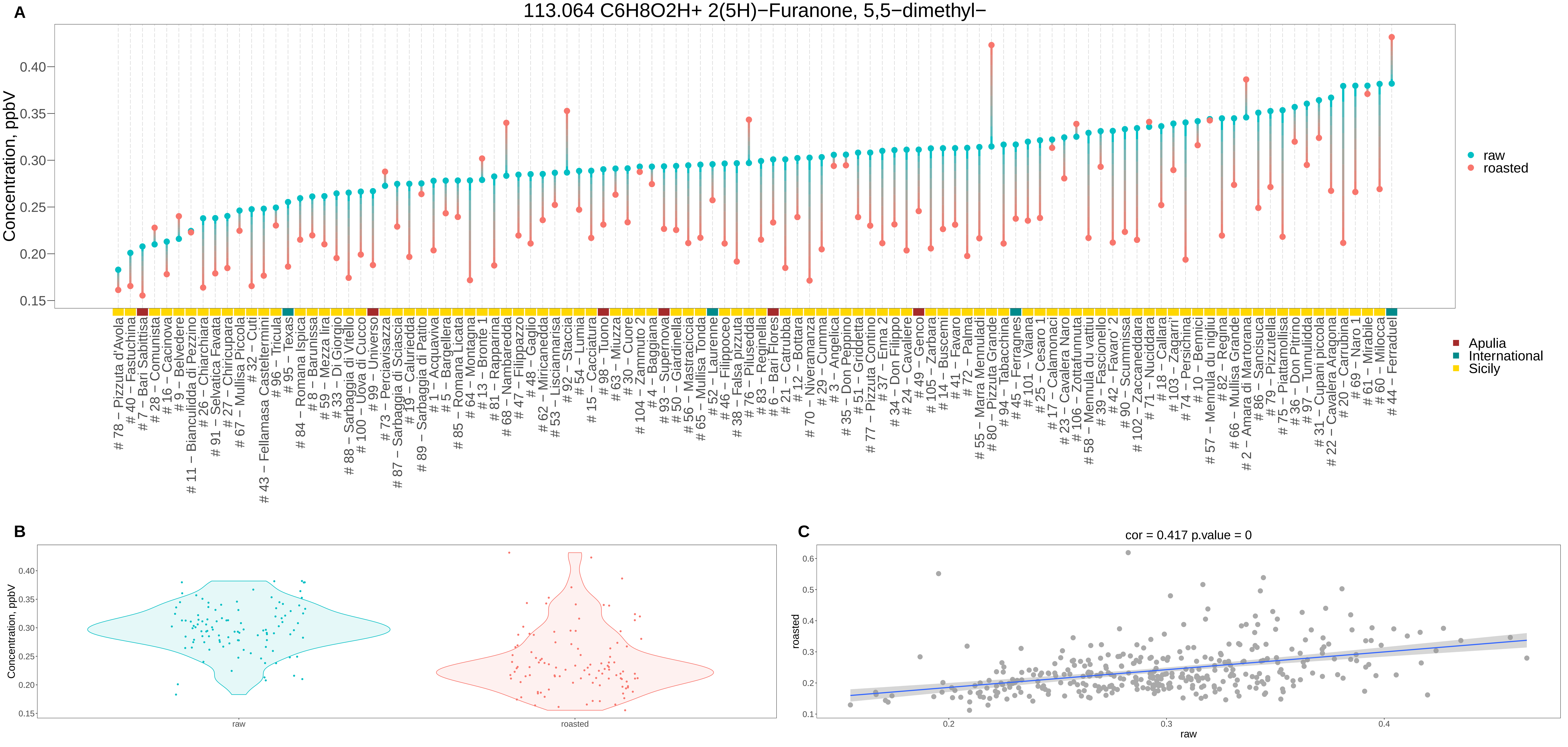

A
113.064 C6H8O2H+ 2(5H)−Furanone, 5,5−dimethyl−
Concentration, ppbV
raw
roasted
Apulia
International
Sicily
78 – Pizzuta d'Avola
40 – Fastuchina
7 – Bari Sabitissa
28 – Comunista
16 – Cacinova
9 – Belvedere
11 – Bianculidda di Pezzino
26 – Chiarchiara
91 – Selvatica Favata
27 – Chiricupara
67 – Mullisa Piccola
32 – Cuti
43 – Fellamasa Casteltermini
96 – Tricula
95 – Texas
84 – Romana Ispica
8 – Barunissa
59 – Mezza lira
33 – Di Giorgio
88 – Sarbaggia di Vitello
100 – Uova di Cucco
99 – Universo
73 – Perciavisazza
87 – Sarbaggia di Sciascia
19 – Caluriedda
89 – Sarbaggia di Patito
1 – Acquaviva
5 – Bargellera
85 – Romana Licata
64 – Montagna
13 – Bronte 1
81 – Rapparina
68 – Nambaredda
47 – Filippazzo
48 – Gaglio
62 – Miricanedda
53 – Liscianarisa
92 – Staccia
54 – Lumia
15 – Cacciatura
98 – Tuono
63 – Miuzza
30 – Cuore
104 – Zammuto 2
4 – Baggiana
93 – Supernova
50 – Giardinella
56 – Mastracicia
65 – Mullisa Tonda
52 – Laurenne
46 – Filippoceo
38 – Falsa pizzuta
76 – Pilusedda
83 – Reginella
6 – Bari Flores
21 – Catrubba
12 – Bottara
70 – Niveramanza
29 – Cumma
3 – Angelica
35 – Don Peppino
51 – Griddetta
77 – Pizzuta Contino
37 – Enna 2
34 – Don Filippo
24 – Cavallere
49 – Genco
105 – Zarbara
14 – Buscemi
41 – Favaro
72 – Palma
55 – Marra Mennuladi
80 – Pizzuta Grande
94 – Tabacchina
45 – Ferragnes
101 – Vaiana
25 – Cesaro 1
17 – Calamonaci
23 – Cavalera Naro
106 – Zottafunnuta
58 – Mennula du vattiu
39 – Fascionello
42 – Favaro' 2
90 – Scummissa
102 – Zaccaneddara
71 – Nuciddara
18 – Callara
103 – Zagarri'
74 – Persichina
10 – Bennici
57 – Mennula du nigliu
82 – Regina
66 – Mullisa Grande
2 – Amara di Martorana
86 – Sancisuca
79 – Pizzutella
75 – Piattamollisa
36 – Don Pitrino
97 – Tunnulidda
31 – Cupani piccola
22 – Cavalera Aragona
20 – Carrubina
69 – Naro 1
61 – Mirabile
60 – Milocca
44 – Ferraduel
B
Concentration, ppbV
raw
roasted
C
cor = 0.417 p.value = 0
roasted
raw

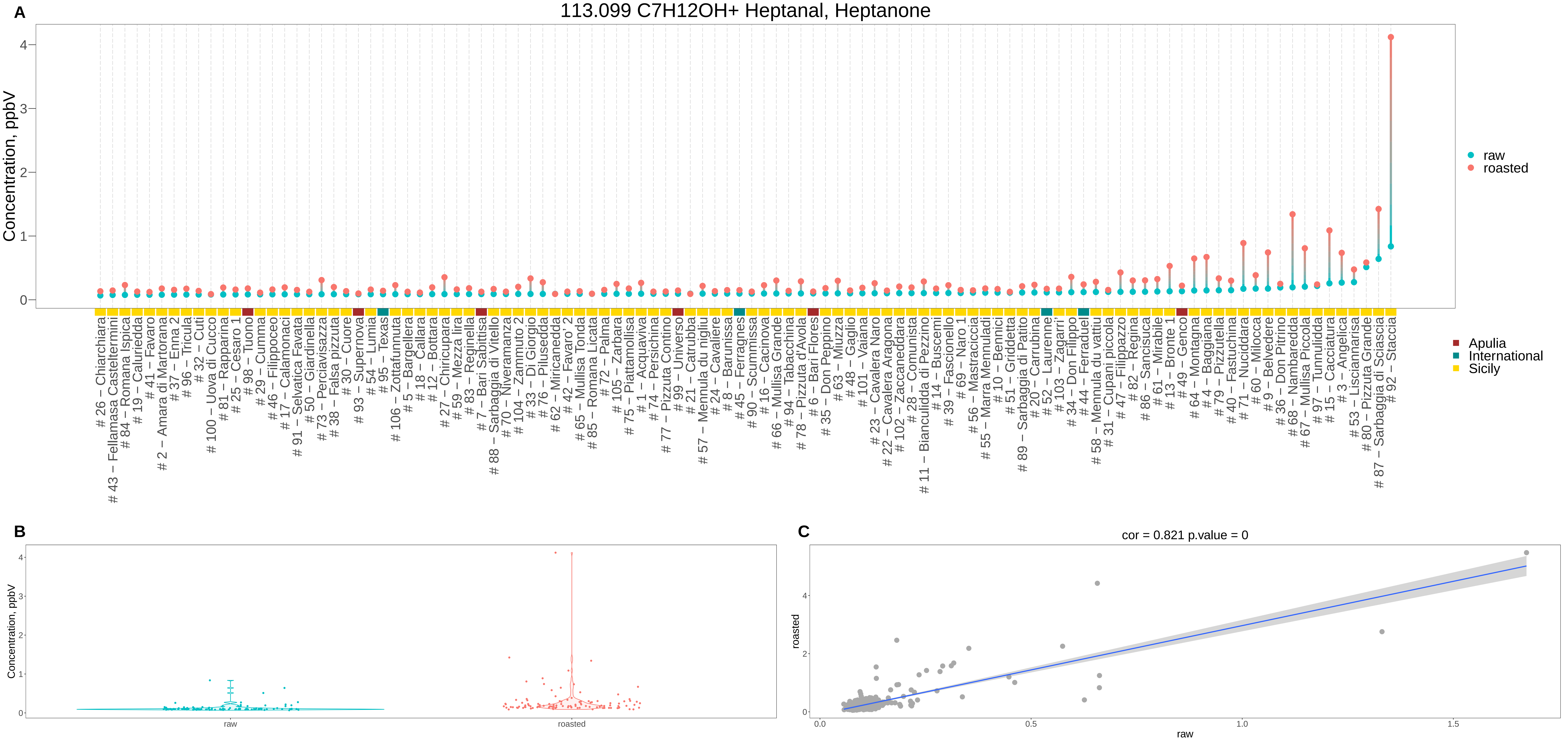
A
113.099 C7H12OH+ Heptanal, Heptanone
Concentration, ppbV
0
1
2
3
4
raw
roasted
Apulia
International
Sicily
26 – Chiarchiara
43 – Fellamasa Casteltermini
84 – Romana Ispica
19 – Caluriedda
41 – Favaro
2 – Amara di Martorana
37 – Enna 2
96 – Tricula
32 – Cuti
100 – Uova di Cucco
81 – Rapparina
25 – Cesaro 1
98 – Tuono
29 – Cumma
46 – Filippoceo
17 – Calamonaci
91 – Selvatica Favata
50 – Giardinella
73 – Perciavisazza
38 – Falsa pizzuta
30 – Cuore
93 – Supernova
54 – Lumia
95 – Texas
106 – Zottafunnuta
5 – Bargellera
18 – Callara
12 – Bottara
27 – Chiricupara
59 – Mezza lira
83 – Reginella
7 – Bari Sabittisa
88 – Sarbaggia di Vitello
70 – Niveramanza
104 – Zammuto 2
33 – Di Giorgio
76 – Pilusedda
62 – Miricanedda
42 – Favaro' 2
65 – Mullisa Tonda
85 – Romana Licata
72 – Palma
105 – Zarbara
75 – Piattamollisa
1 – Acquaviva
74 – Persichina
77 – Pizzuta Contino
99 – Universo
21 – Catrubba
57 – Mennula du nigliu
24 – Cavaliere
8 – Barunissa
45 – Ferragnes
90 – Scummissa
16 – Cacinova
66 – Mullisa Grande
94 – Tabacchina
78 – Pizzuta d'Avola
6 – Bari Flores
35 – Don Peppino
63 – Mlizza
48 – Gaglio
101 – Vaiana
23 – Cavalera Naro
22 – Cavalera Aragona
102 – Zaccaneddara
28 – Comunista
11 – Bianculidda di Pezzino
14 – Buscemi
39 – Fascionello
69 – Naro 1
56 – Mastracicia
55 – Marra Mennuladi
10 – Bennici
51 – Griddetta
89 – Sarbaggia di Patito
20 – Carrubina
52 – Laurenne
103 – Zagarri'
34 – Don Filippo
44 – Ferraduel
58 – Mennula du vattiu
31 – Cupani piccola
47 – Filippazzo
82 – Regina
86 – Sancisuca
61 – Mirabile
13 – Bronte 1
49 – Genco
64 – Montagna
4 – Baggiana
79 – Pizzutella
40 – Fastuchina
71 – Nuciddara
60 – Milocca
9 – Belvedere
36 – Don Pitrino
68 – Nambaredda
67 – Mullisa Piccola
97 – Tunnulidda
15 – Cacciatura
3 – Angelica
53 – Liscianarisa
80 – Pizzuta Grande
87 – Sarbaggia di Sciascia
92 – Staccia
B
Concentration, ppbV
0
1
2
3
4
raw
roasted
C
cor = 0.821 p.value = 0
roasted
0
2
4
raw
0.0
0.5
1.0
1.5

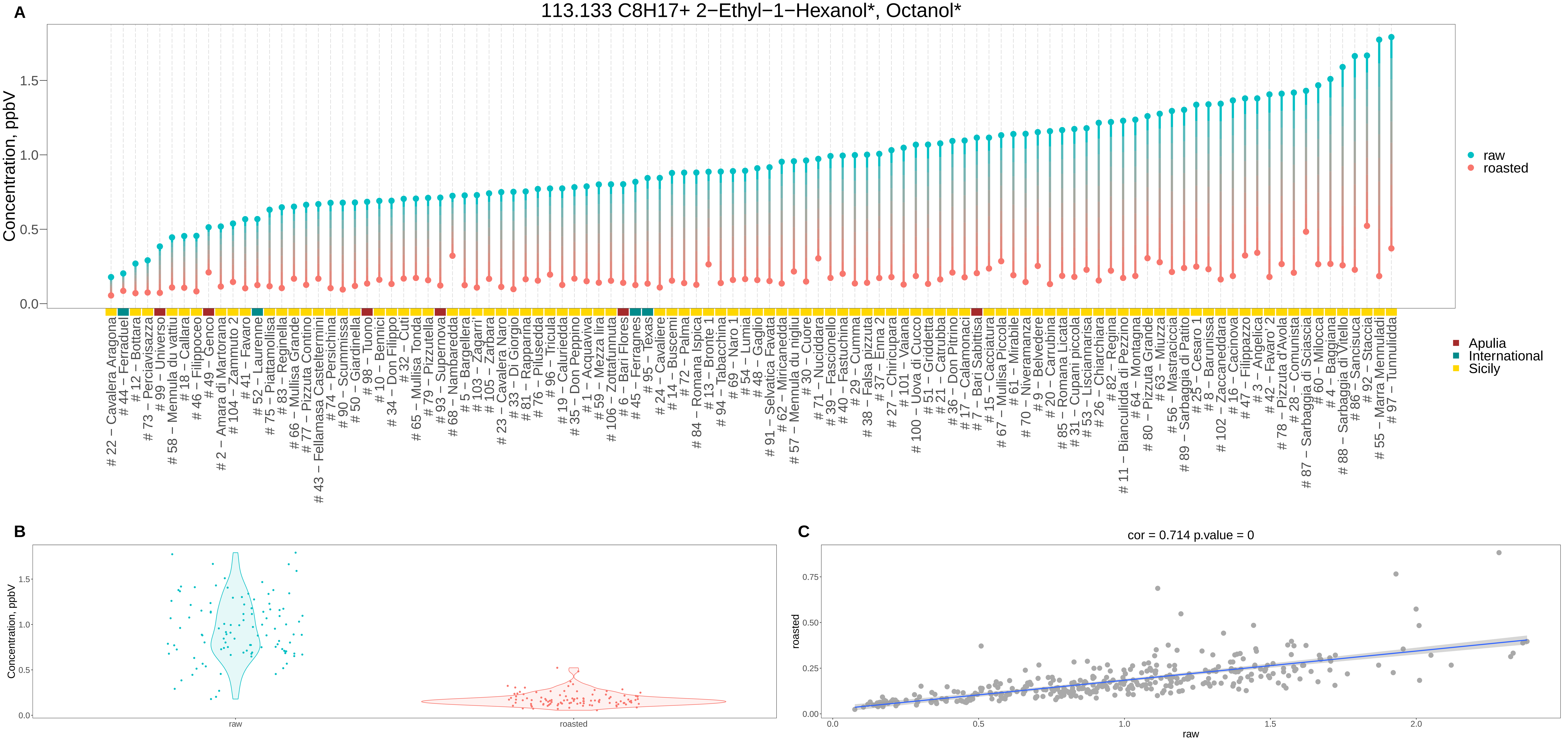
A
113.133 C8H17+ 2−Ethyl−1−Hexanol*, Octanol*
Concentration, ppbV
raw
roasted
Apulia
International
Sicily
22 – Cavalera Aragona
44 – Ferraduel
12 – Bottara
73 – Perciavisazza
99 – Universo
58 – Mennula du vattiu
18 – Callara
46 – Filippoceo
49 – Genco
2 – Amara di Martorana
104 – Zammuto 2
41 – Favaro
52 – Laurenne
75 – Piattamollisa
83 – Reginella
66 – Mullisa Grande
77 – Pizzuta Contino
43 – Fellamasa Casteltermini
74 – Persichina
90 – Scummissa
50 – Giardinella
98 – Tuono
10 – Bennici
34 – Don Filippo
32 – Cuti
65 – Mullisa Tonda
79 – Pizzutella
93 – Supernova
68 – Nambaredda
5 – Bargellera
103 – Zagarri'
105 – Zarbara
23 – Cavalera Naro
33 – Di Giorgio
81 – Rapparina
76 – Pilusedda
96 – Tricula
19 – Caluriedda
35 – Don Peppino
1 – Acquaviva
59 – Mezza lira
106 – Zottafunnuta
6 – Bari Flores
45 – Ferragnes
95 – Texas
24 – Cavaliere
14 – Buscemi
72 – Palma
84 – Romana Ispica
13 – Bronte 1
94 – Tabacchina
69 – Naro 1
54 – Lumia
48 – Gaglio
91 – Selvatica Favata
62 – Miricanedda
57 – Mennula du nigliu
30 – Cuore
71 – Nuciddara
39 – Fascionello
40 – Fastuchina
29 – Cumma
38 – Falsa pizzuta
37 – Enna 2
27 – Chiricupara
101 – Vaiana
100 – Uova di Cucco
51 – Griddetta
21 – Catrubba
36 – Don Pitrino
17 – Calamonaci
7 – Bari Sabitissa
15 – Cacciatura
67 – Mullisa Piccola
61 – Mirabile
70 – Niveramanza
9 – Belvedere
20 – Carrubina
85 – Romana Licata
31 – Cupani piccola
53 – Lisciannarisa
26 – Chiarchiara
82 – Regina
11 – Bianculidda di Pezzino
64 – Montagna
80 – Pizzuta Grande
63 – Miuzza
56 – Mastraciccia
89 – Sarbaggia di Patito
25 – Cesaro 1
8 – Barunissa
102 – Zaccaneddara
16 – Cacinova
47 – Filippazzo
3 – Angelica
42 – Favaro'2
78 – Pizzuta d'Avola
28 – Comunista
87 – Sarbaggia di Sciascia
60 – Milocca
4 – Baggiana
88 – Sarbaggia di Vitello
86 – Sancisuca
92 – Staccia
55 – Marra Mennuladi
97 – Tunnulidda
B
Concentration, ppbV
raw
roasted
C
cor = 0.714 p.value = 0
roasted
raw

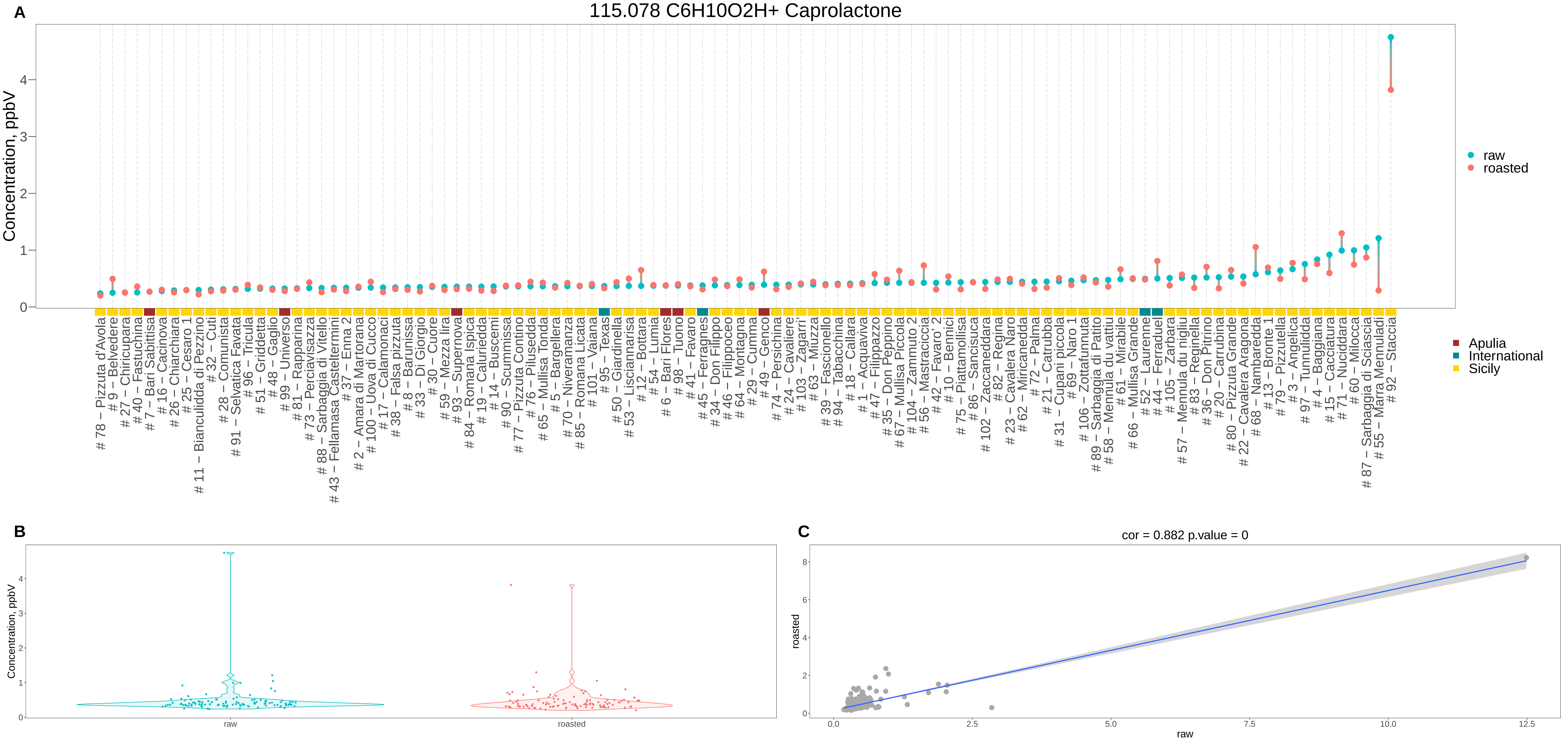
A
115.078 C6H10O2H+ Caprolactone
Concentration, ppbV
raw
roasted
Apulia
International
Sicily
78 – Pizzuta d'Avola
9 – Belvedere
27 – Chiricupara
40 – Fastuchina
7 – Bari Sabittisa
16 – Cacinova
26 – Chiarchiara
25 – Cesaro 1
32 – Cuti
11 – Bianculidda di Pezzino
28 – Comunista
91 – Selvatica Favata
96 – Triculà
51 – Griddetta
48 – Gaglio
99 – Universo
81 – Rapparina
73 – Perciavisazza
88 – Sarbaggia di Vitello
43 – Fellamasa Casteltermini
37 – Enna 2
2 – Amara di Martorana
100 – Uova di Cucco
17 – Calamonaci
38 – Falsa pizzuta
8 – Barunissa
33 – Di Giorgio
30 – Cuore
59 – Mezza lira
93 – Supernova
84 – Romana Ispica
19 – Caluriedda
14 – Buscemi
90 – Scummissa
77 – Pizzuta Contino
76 – Pilusedda
65 – Mullisa Tonda
5 – Bargellera
70 – Niveramanza
85 – Romana Licata
101 – Vaiana
95 – Texas
50 – Giardinella
53 – Lisciannarisa
12 – Bottara
54 – Lumia
6 – Bari Flores
98 – Tuono
41 – Favaro
45 – Ferragnes
34 – Don Filippo
46 – Filippoceo
64 – Montagna
29 – Cumma
49 – Genco
74 – Persichina
24 – Cavaliere
103 – Zagarri'
63 – Miuzza
39 – Fascionello
94 – Tabacchina
18 – Callara
1 – Acquaviva
47 – Filippazzo
35 – Don Peppino
67 – Mullisa Piccola
104 – Zammuto 2
56 – Mastracicca
42 – Favaro' 2
10 – Bennici
75 – Piattamollisa
86 – Sancisuca
102 – Zaccaneddara
82 – Regina
23 – Cavalera Naro
62 – Miricanedda
72 – Palma
21 – Catrubba
31 – Cupani piccola
69 – Naro 1
106 – Zottafunnuta
89 – Sarbaggia di Patitto
58 – Mennula du vattiu
61 – Mirabile
66 – Mullisa Grande
52 – Laurenne
44 – Ferraduel
105 – Zarbara
57 – Mennula du nigliu
83 – Reginella
36 – Don Pitrino
20 – Carrubina
80 – Pizzuta Grande
22 – Cavalera Aragona
68 – Nambaredda
13 – Bronte 1
79 – Pizzutella
3 – Angelica
97 – Tunnulidda
4 – Baggiana
15 – Cacciatura
71 – Nuciddara
60 – Milocca
87 – Sarbaggia di Sciascia
55 – Marra Mennuladi
92 – Staccia
B
Concentration, ppbV
raw
roasted
C
cor = 0.882 p.value = 0
roasted
raw

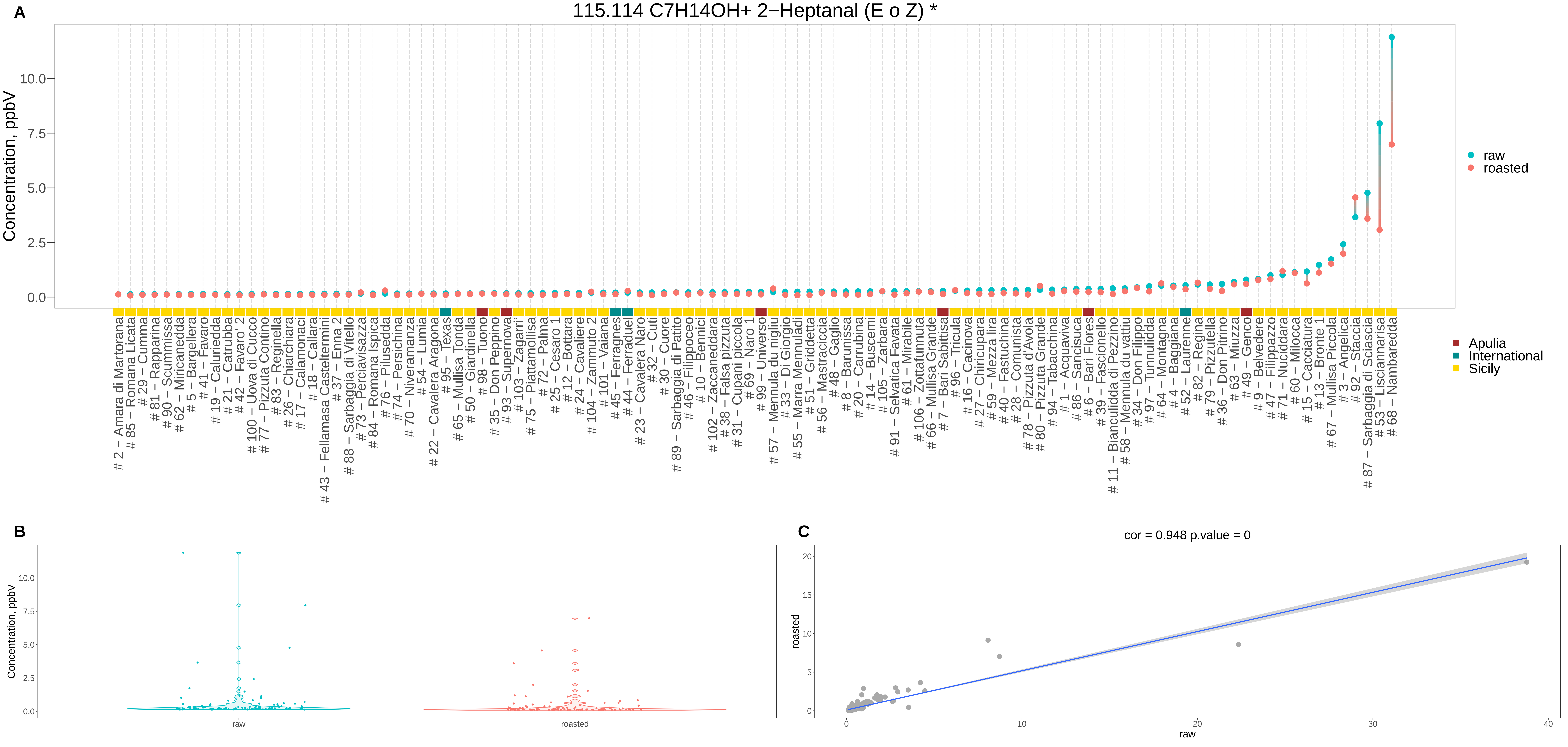
A
115.114 C7H14OH+ 2−Heptanal (E o Z) *
Concentration, ppbV
0.0
2.5
5.0
7.5
10.0
raw
roasted
Apulia
International
Sicily
2 − Amara di Martorana
85 − Romana Licata
29 − Cumma
81 − Rapparina
90 − Scummissa
62 − Miricanedda
5 − Bargellera
41 − Favaro
19 − Caluriedda
21 − Catrubba
42 − Favaro 2
100 − Uova di Cucco
77 − Pizzuta Contino
83 − Reginella
26 − Chiarchiara
17 − Calamonaci
18 − Callara
43 − Fellamasa Casteltermini
37 − Enna 2
88 − Sarbaggia di Vitello
73 − Perciavisazza
84 − Romana Ispica
76 − Pilusedda
74 − Persichina
70 − Niveramanza
54 − Lumia
22 − Cavalera Aragona
95 − Texas
65 − Mullisa Tonda
50 − Giardinella
98 − Tuono
35 − Don Peppino
93 − Supernova
103 − Zagarri'
75 − Piattamollisa
72 − Palma
25 − Cesaro 1
12 − Bottara
24 − Cavaliere
104 − Zammuto 2
101 − Vaiana
45 − Ferragnes
44 − Ferraduel
23 − Cavalera Naro
32 − Cuti
30 − Cuore
89 − Sarbaggia di Patito
46 − Filippoceo
10 − Bennici
102 − Zaccaneddara
38 − Falsa pizzuta
31 − Cupani piccola
69 − Naro 1
99 − Universo
57 − Mennula du nigliu
33 − Di Giorgio
55 − Marra Mennuladi
51 − Griddetta
56 − Mastraciccia
48 − Gaglio
8 − Barunissa
20 − Carrubina
14 − Buscemi
105 − Zarbara
91 − Selvatica Favata
61 − Mirabile
106 − Zottafumnuta
66 − Mullisa Grande
7 − Bari Sabittisa
96 − Tricula
16 − Cacinova
27 − Chiricupara
59 − Mezza lira
40 − Fastuchina
28 − Comunista
78 − Pizzuta d'Avola
80 − Pizzuta Grande
94 − Tabacchina
1 − Acquaviva
86 − Sancisuca
6 − Bari Flores
39 − Fascionello
11 − Biancuiidda di Pezzino
58 − Mennula du vattiu
34 − Don Filippo
97 − Tunnulidda
64 − Montagna
4 − Baggiana
52 − Laurenne
82 − Regina
79 − Pizzutella
36 − Don Pitrino
63 − Miuzza
49 − Genco
9 − Belvedere
47 − Filippazzo
71 − Nuciddara
60 − Milocca
15 − Cacciatura
13 − Bronte 1
67 − Mullisa Piccola
3 − Angelica
92 − Staccia
87 − Sarbaggia di Sciascia
53 − Liscinnarisa
68 − Nambaredda
B
Concentration, ppbV
0.0
2.5
5.0
7.5
10.0
raw
roasted
C
cor = 0.948 p.value = 0
roasted
raw
0
5
10
15
20
30
40

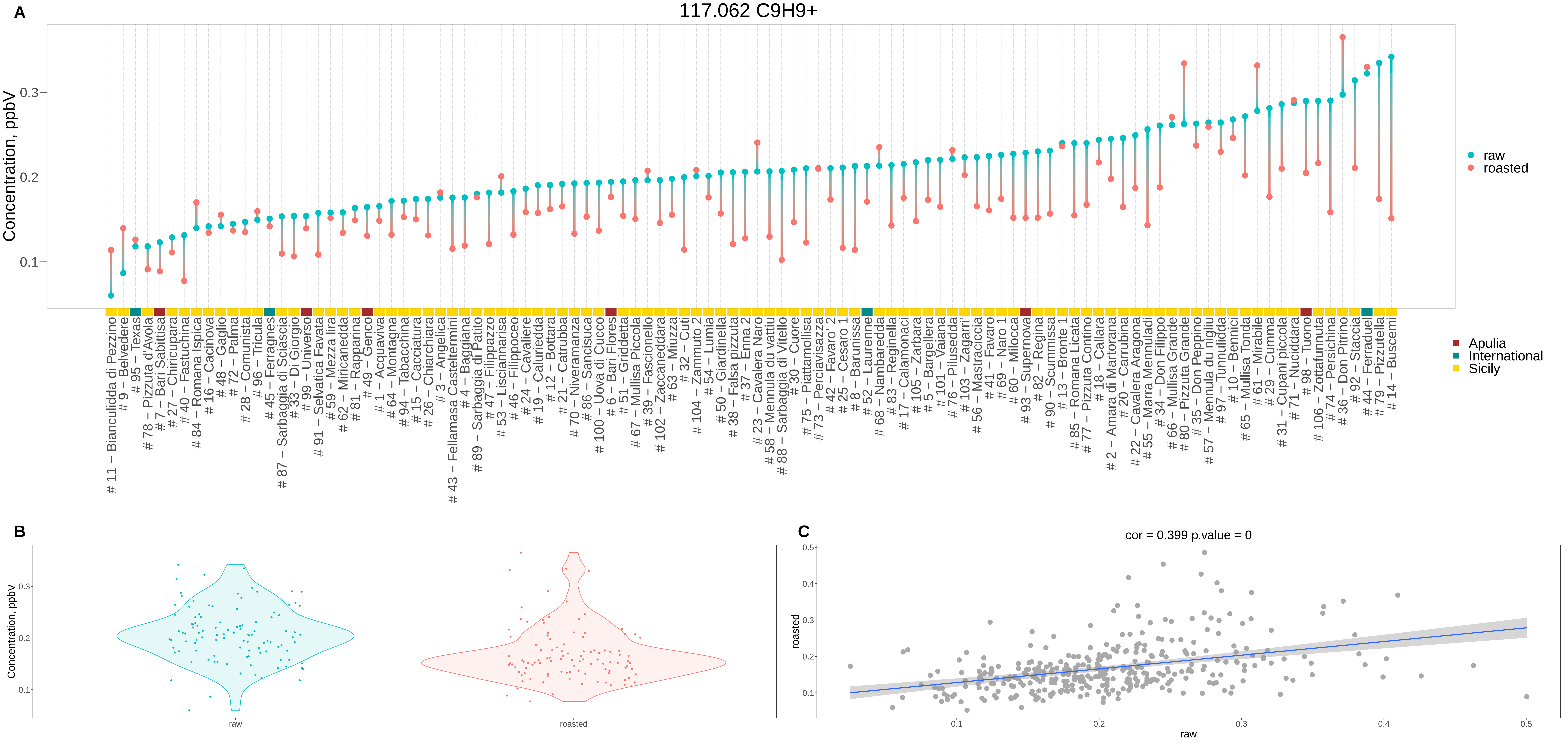
A
117.062 C9H9+
Concentration, ppbV
0.1
0.2
0.3
raw
roasted
Apulia
International
Sicily
11 – Bianculidda di Pezzino
9 – Belvedere
95 – Texas
78 – Pizzuta d'Avola
7 – Bari Sabitisa
27 – Chiricupara
40 – Fastuchina
84 – Romana Ispica
16 – Cacinova
48 – Gaglio
72 – Palma
28 – Comunista
96 – Tricula
45 – Ferragnes
87 – Sarbaggia di Sciascia
33 – Di Giorgio
99 – Universo
91 – Selvatica Favata
59 – Mezza lira
62 – Miricanedda
81 – Rapparina
49 – Genco
1 – Acquaviva
64 – Montagna
94 – Tabacchina
15 – Cacciatura
26 – Chiarchiara
3 – Angelica
43 – Fellamasa Casteltermini
4 – Baggiana
89 – Sarbaggia di Patito
47 – Filippazzo
53 – Liscianarisa
46 – Filippoceo
24 – Cavaliere
19 – Calurieddia
12 – Bottara
21 – Catrubba
70 – Niveramanza
86 – Sancisuca
100 – Uova di Cucco
6 – Bari Flores
51 – Griddetta
67 – Mullisa Piccola
39 – Fascionello
102 – Zaccaneddara
63 – Miuzza
32 – Cuti
104 – Zammuto 2
54 – Lumia
50 – Giardinella
38 – Falsa pizzuta
37 – Enna 2
23 – Cavalera Naro
58 – Mennula du vattiu
88 – Sarbaggia di Vitello
30 – Cuore
75 – Piattamollisa
73 – Perciavisazza
42 – Favaro' 2
25 – Cesaro 1
8 – Barunissa
52 – Laurenne
68 – Nambaredda
83 – Reginella
17 – Calamonaci
105 – Zarbara
5 – Bargellera
101 – Vaiana
76 – Pilusedda
103 – Zagarri'
56 – Mastraciccia
41 – Favaro
69 – Naro 1
60 – Milocca
93 – Supernova
82 – Regina
90 – Scummissa
13 – Bronte 1
85 – Romana Licata
77 – Pizzuta Contino
18 – Callara
2 – Amara di Martorana
20 – Carrubina
22 – Cavalera Aragona
55 – Marra Mennuladi
34 – Don Filippo
66 – Mullisa Grande
80 – Pizzuta Grande
35 – Don Peppino
57 – Mennula di nigliu
97 – Tunnulidda
10 – Bennici
65 – Mullisa Tonda
61 – Mirabile
29 – Cumma
31 – Cupani piccola
71 – Nuciddara
98 – Tuono
106 – Zottafunnuta
74 – Persichina
36 – Don Pitrino
92 – Staccia
44 – Ferraduel
79 – Pizzutella
14 – Buscemi
B
Concentration, ppbV
raw
roasted
C
cor = 0.399 p.value = 0
roasted
raw

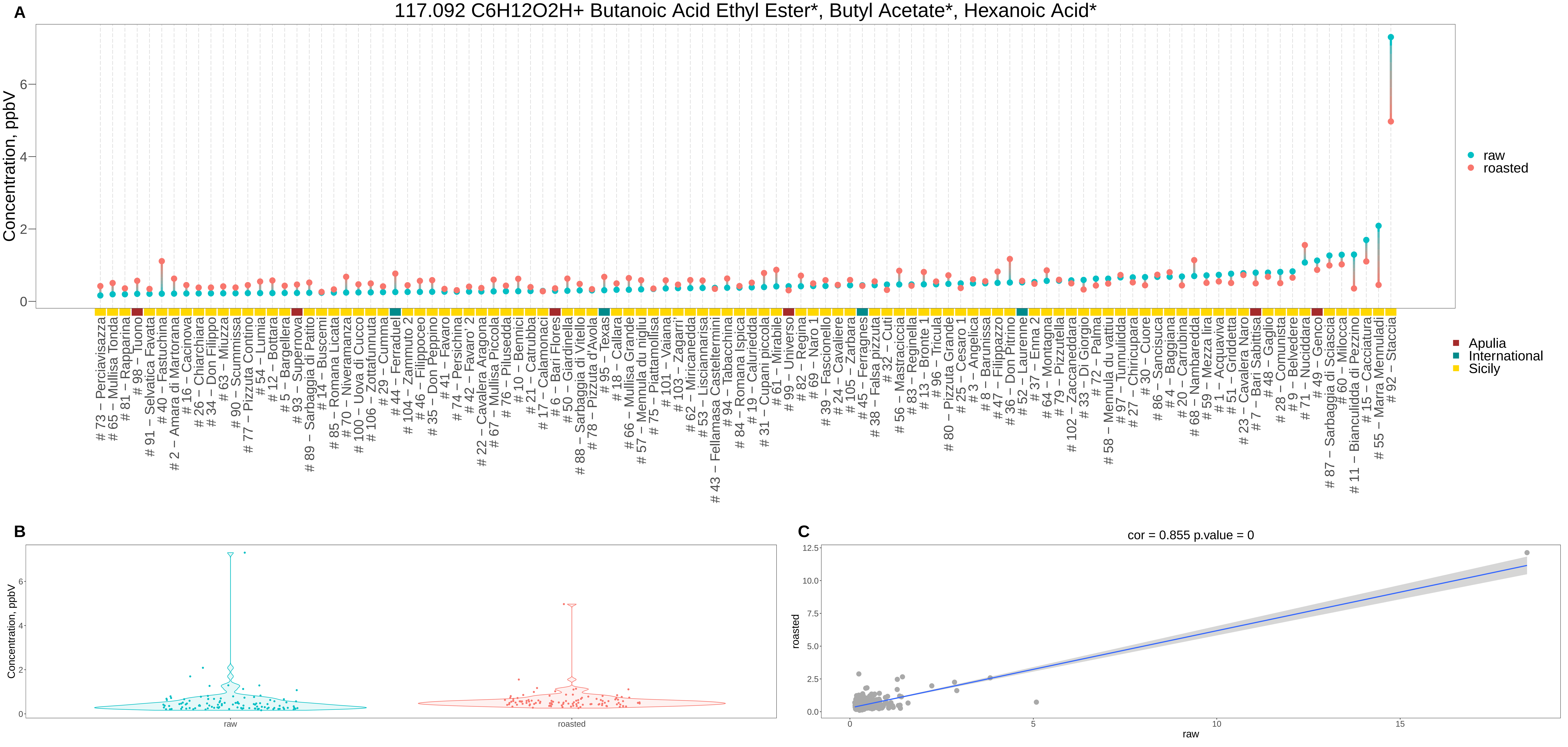

A
117.092 C6H12O2H+ Butanoic Acid Ethyl Ester*, Butyl Acetate*, Hexanoic Acid*
Concentration, ppbV
0
2
4
6
raw
roasted
Apulia
International
Sicily
73 – Perciavisazza
65 – Mullisa Tonda
81 – Rapparina
98 – Tuono
91 – Selvatica Favata
40 – Fastuchina
2 – Amara di Martorana
16 – Cacinova
26 – Chiarchiara
34 – Don Filippo
63 – Miuzza
90 – Scummissa
77 – Pizzuta Contino
54 – Lumia
12 – Bottara
5 – Bargellera
93 – Supernova
89 – Sarbaggia di Patito
14 – Buscemi
85 – Romana Licata
70 – Niveramanza
100 – Uova di Cucco
106 – Zottafunnuta
29 – Cumma
44 – Ferraduel
104 – Zammuto 2
46 – Filippoceo
35 – Don Peppino
41 – Favaro
74 – Persichina
42 – Favaro' 2
22 – Cavalera Aragona
67 – Mullisa Piccola
76 – Pilusedda
10 – Bennici
21 – Catrubba
17 – Calamonaci
6 – Bari Flores
50 – Giardinella
88 – Sarbaggia di Vitello
78 – Pizzuta d'Avola
95 – Texas
18 – Callara
66 – Mullisa Grande
57 – Mennula du nigliu
75 – Piattamollisa
101 – Vaiana
103 – Zagarri'
62 – Miricanedda
53 – Liscianarisa
43 – Fellamasa Casteltermini
94 – Tabacchina
84 – Romana Ispica
19 – Caluriedda
31 – Cupani piccola
61 – Mirabile
99 – Universo
82 – Regina
69 – Naro 1
39 – Fascionello
24 – Cavaliere
105 – Zarbara
45 – Ferragnes
38 – Falsa pizzuta
32 – Cuti
56 – Mastracicia
83 – Reginella
13 – Bronte 1
96 – Tricula
80 – Pizzuta Grande
25 – Cesaro 1
3 – Angelica
8 – Barunissa
47 – Filippazzo
36 – Don Pitrino
52 – Laurenne
37 – Enna 2
64 – Montagna
79 – Pizzutella
102 – Zaccaneddara
33 – Di Giorgio
72 – Palma
58 – Mennula du vattiu
97 – Tunnulidda
27 – Chiricupara
30 – Cuore
86 – Sancisuca
4 – Baggiana
20 – Carrubina
68 – Nambaredda
59 – Mezza lira
1 – Acquaviva
51 – Griddetta
23 – Cavalera Naro
7 – Bari Sabittisa
48 – Gaglio
28 – Comunista
9 – Belvedere
71 – Nuciddara
49 – Genco
87 – Sarbaggia di Sciascia
60 – Milocca
11 – Bianculidda di Pezzino
15 – Cacciatura
55 – Marra Mennuladi
92 – Staccia
B
Concentration, ppbV
raw
roasted
C
cor = 0.855 p.value = 0
roasted
raw
0
5
10
15
0.0
2.5
5.0
7.5
10.0
12.5

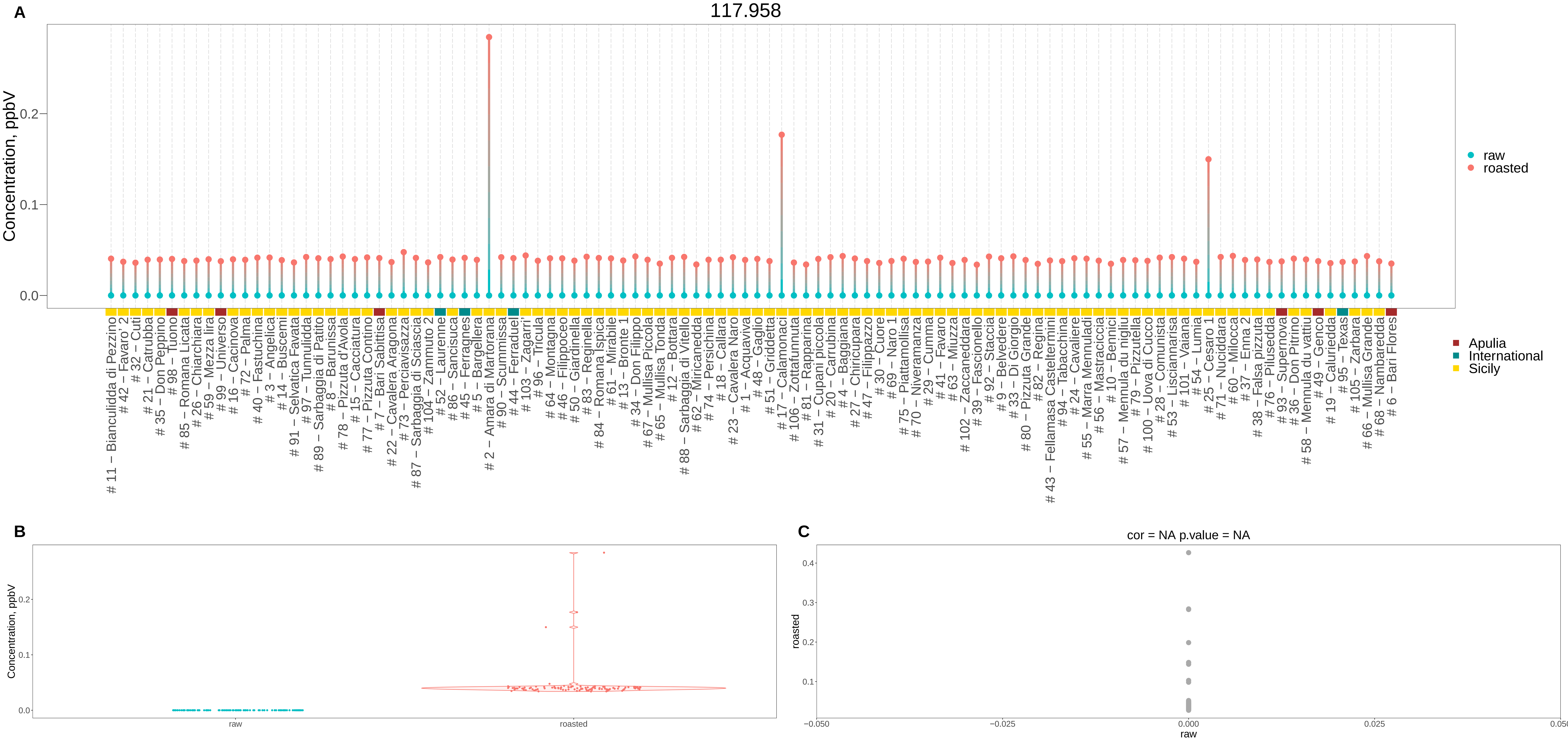

A
117.958
Concentration, ppbV
0.0
0.1
0.2
raw
roasted
Apulia
International
Sicily
11 – Bianculidda di Pezzino
42 – Favaro' 2
32 – Cuti
21 – Catrubba
35 – Don Peppino
98 – Tuono
85 – Romana Licata
26 – Chiarchiara
59 – Mezza lira
99 – Universo
16 – Cacinova
72 – Palma
40 – Fastuchina
3 – Angelica
14 – Buscemi
91 – Selvatica Favata
97 – Tunnulidda
89 – Sarbaggia di Patito
8 – Barunissa
78 – Pizzuta d'Avola
15 – Cacciatura
77 – Pizzuta Contino
7 – Bari Sabittisa
22 – Cavalera Aragona
73 – Perciavisazza
87 – Sarbaggia di Sciascia
104 – Zammuto 2
52 – Laurenne
86 – Sancisuca
45 – Ferragnes
5 – Bargellera
2 – Amara di Martorana
90 – Scummissa
44 – Ferraduel
103 – Zagari
96 – Tricula
64 – Montagna
46 – Filippoceo
50 – Giardinella
83 – Reginella
84 – Romana Ispica
61 – Mirabile
13 – Bronte 1
34 – Don Filippo
67 – Mullisa Piccola
65 – Mullisa Tonda
12 – Bottara
88 – Sarbaggia di Vitello
62 – Miricanedda
74 – Persichina
18 – Callara
23 – Cavalera Naro
1 – Acquaviva
48 – Gaglio
51 – Griddetta
17 – Calamonaci
106 – Zottafunnuta
81 – Rapparina
31 – Cupani piccola
20 – Carrubina
4 – Baggiana
27 – Chiricupara
47 – Filippazzo
30 – Cuore
69 – Naro 1
75 – Piattamollisa
70 – Niveramanza
29 – Cumma
41 – Favaro
63 – Miuzza
102 – Zaccaneddara
39 – Fascionello
92 – Staccia
9 – Belvedere
33 – Di Giorgio
80 – Pizzuta Grande
82 – Regina
43 – Fellamasa Casteltermini
94 – Tabacchina
24 – Cavaliere
55 – Marra Mennuladi
56 – Mastraciccia
10 – Bennici
57 – Mennula du nigliu
79 – Pizzutella
100 – Uova di Cucco
28 – Comunista
53 – Lisciannarisa
101 – Vaiana
54 – Lumia
25 – Cesaro 1
71 – Nuciddara
60 – Milocca
37 – Enna 2
38 – Falsa pizzuta
76 – Pilusedda
93 – Supernova
36 – Don Pitrino
58 – Mennula du vattiu
49 – Genco
19 – Caluriedda
95 – Texas
105 – Zarbara
66 – Mullisa Grande
68 – Nambaredda
6 – Bari Flores
B
Concentration, ppbV
0.0
0.1
0.2
raw
roasted
C
cor = NA p.value = NA
roasted
0.1
0.2
0.3
0.4
raw
−0.050
−0.025
0.000
0.025

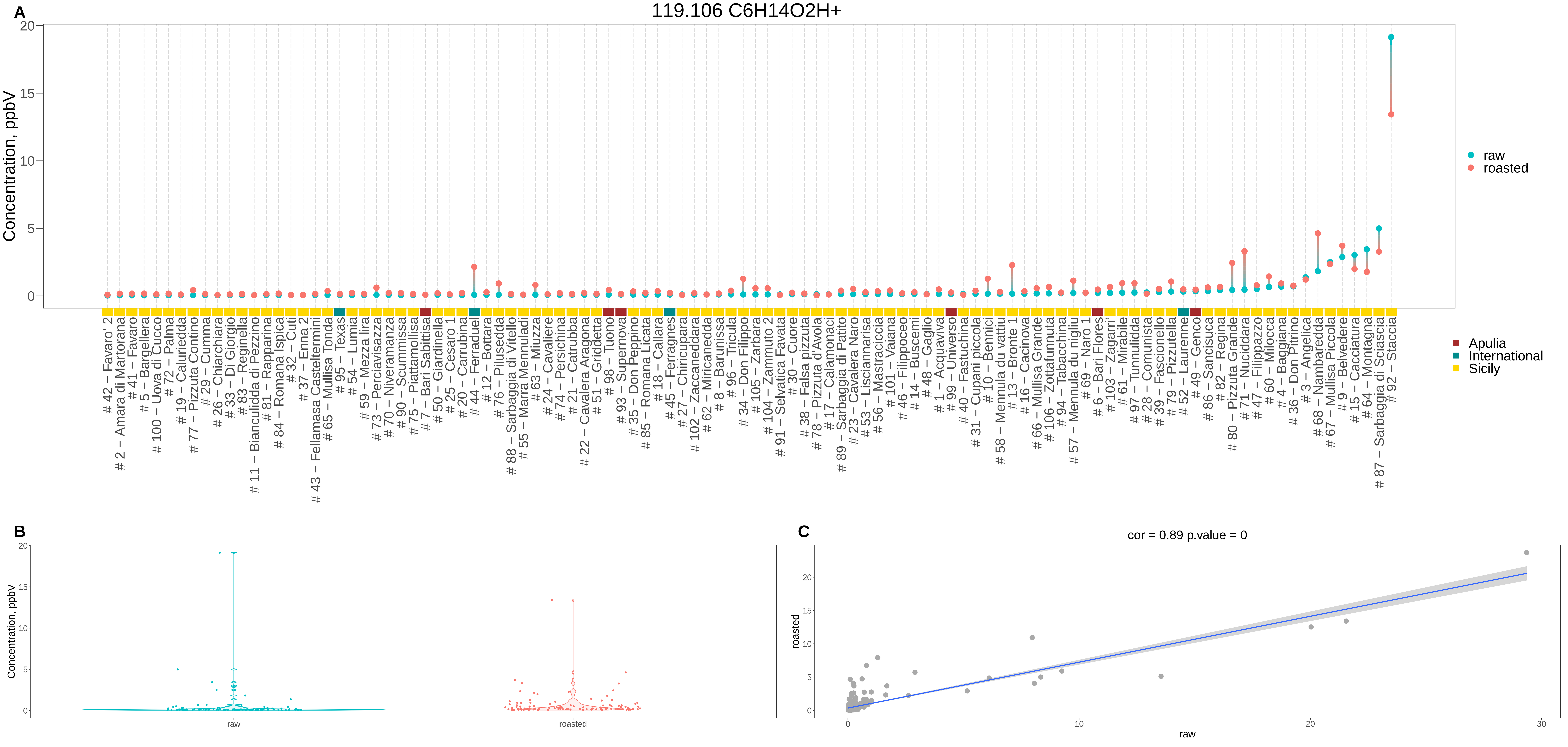

A
119.106 C6H14O2H+
Concentration, ppbV
raw
roasted
Apulia
International
Sicily
42 – Favaro' 2
2 – Amara di Martorana
41 – Favaro
5 – Bargellera
100 – Uova di Cucco
72 – Palma
19 – Caluriedda
77 – Pizzuta Contino
29 – Cumma
26 – Chiarchiara
33 – Di Giorgio
83 – Reginella
11 – Bianculidda di Pezzino
81 – Rapparina
84 – Romana Ispica
32 – Cuti
37 – Enna 2
43 – Fellamasa Casteltermini
65 – Mullisa Tonda
95 – Texas
54 – Lumia
59 – Mezza lira
73 – Perciavisazza
70 – Niveramanza
90 – Scummissa
75 – Piattamollisa
7 – Bari Sabittisa
50 – Giardinella
25 – Cesaro 1
20 – Carrubina
44 – Ferraduel
12 – Bottara
76 – Pilusedda
88 – Sarbaggia di Vitello
55 – Marra Mennuladi
63 – Miuzza
24 – Cavaliere
74 – Persichina
21 – Catrubba
22 – Cavalera Aragona
51 – Griddetta
98 – Tuono
93 – Supernova
35 – Don Peppino
85 – Romana Licata
18 – Callara
45 – Ferragnes
27 – Chiricupara
102 – Zaccaneddara
62 – Miricanedda
8 – Barunissa
96 – Tricula
34 – Don Filippo
105 – Zarbara
104 – Zammuto 2
91 – Selvatica Favata
30 – Cuore
38 – Falsa pizzuta
78 – Pizzuta d'Avola
17 – Calamonaci
89 – Sarbaggia di Patito
23 – Cavalera Naro
53 – Lisciannarisa
56 – Mastraciccia
101 – Vaiana
46 – Filippoceo
14 – Buscemi
48 – Gaglio
1 – Acquaviva
99 – Universo
40 – Fastuchina
31 – Cupani piccola
10 – Bennici
58 – Mennula du vattiu
13 – Bronte 1
16 – Cacinova
66 – Mullisa Grande
106 – Zottafunnuta
94 – Tabacchina
57 – Mennula du nigliu
69 – Naro 1
6 – Bari Flores
103 – Zagarri'
61 – Mirabile
97 – Tunnulidda
28 – Comunista
39 – Fascionello
79 – Pizzutella
52 – Laurenne
49 – Genco
86 – Sancisuca
82 – Regina
80 – Pizzuta Grande
71 – Nuciddara
47 – Filippazzo
60 – Milocca
4 – Baggiana
36 – Don Pitrino
3 – Angelica
68 – Nambaredda
67 – Mullisa Piccola
9 – Belvedere
15 – Cacciatura
64 – Montagna
87 – Sarbaggia di Sciascia
92 – Staccia
B
Concentration, ppbV
raw
roasted
C
cor = 0.89 p.value = 0
roasted
raw

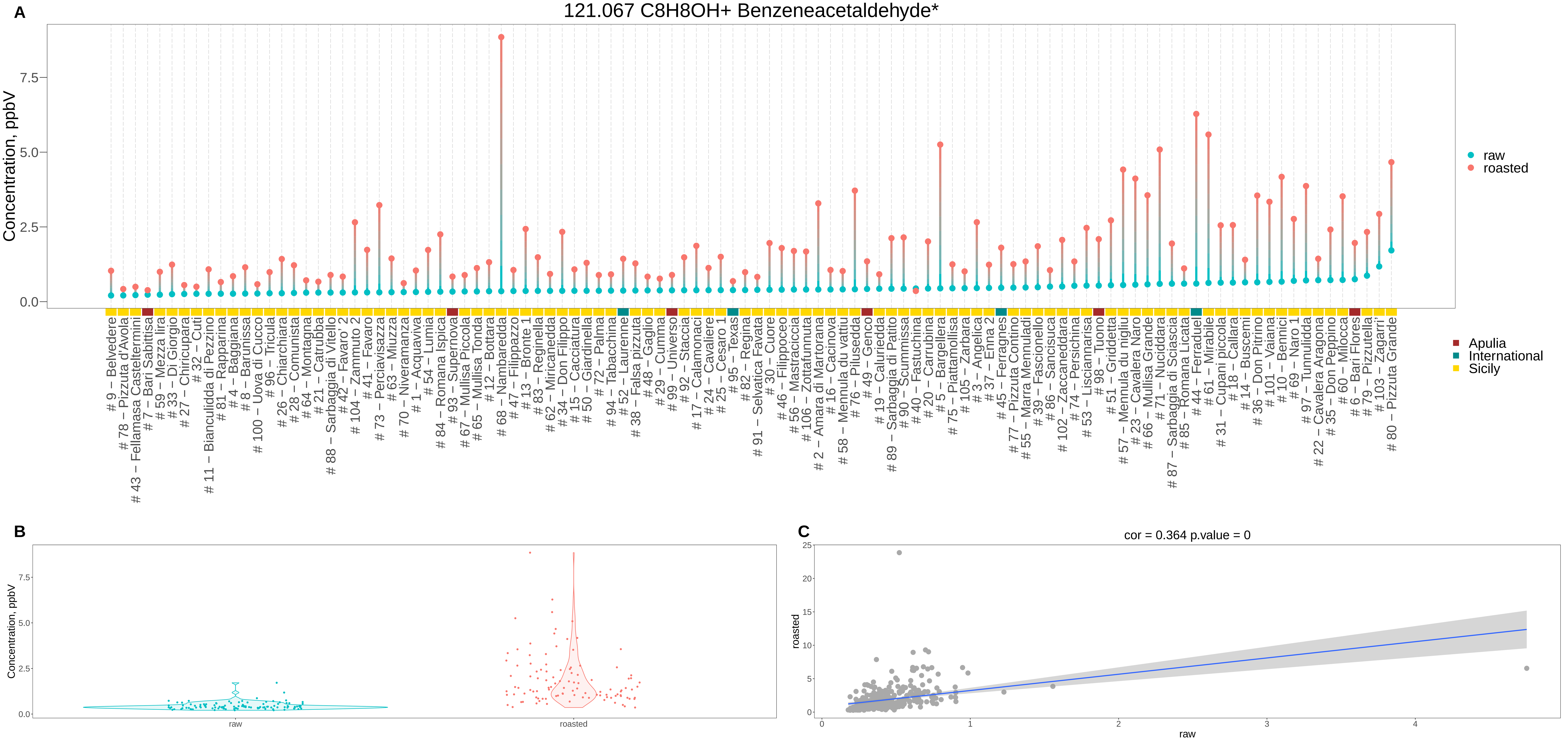
A
121.067 C8H8OH+ Benzeneacetaldehyde*
Concentration, ppbV
0.0
2.5
5.0
7.5
raw
roasted
Apulia
International
Sicily
9 – Belvedere
78 – Pizzuta d'Avola
43 – Fellamasa Casteltermini
7 – Bari Sabittisa
59 – Mezza lira
33 – Di Giorgio
27 – Chiricupara
32 – Cuti
11 – Bianculidda di Pezzino
81 – Rapparina
4 – Baggiana
8 – Barunissa
100 – Uova di Cucco
96 – Tricula
26 – Chiarchiara
28 – Comunista
64 – Montagna
21 – Catrubba
88 – Sarbaggia di Vitello
42 – Favaro' 2
104 – Zammuto 2
41 – Favaro
73 – Perciavisazza
63 – Miuzza
70 – Niveramanza
1 – Acquaviva
54 – Lumia
84 – Romana Ispica
93 – Supernova
67 – Mullisa Piccola
65 – Mullisa Tonda
12 – Bottara
68 – Nambaredda
47 – Filippazzo
13 – Bronte 1
83 – Reginella
62 – Miricanedda
34 – Don Filippo
15 – Cacciatura
50 – Giardinella
72 – Palma
94 – Tabacchina
52 – Laurenne
38 – Falsa pizzuta
48 – Gaglio
29 – Cumma
99 – Universo
92 – Staccia
17 – Calamonaci
24 – Cavaliere
25 – Cesaro 1
95 – Texas
82 – Regina
91 – Selvatica Favata
30 – Cuore
46 – Filippoeo
56 – Mastracicia
106 – Zottafunnuta
2 – Amara di Martorana
16 – Cacinova
58 – Mennula du vattiu
76 – Pilusedda
49 – Genco
19 – Caluriedda
89 – Sarbaggia di Patito
90 – Scummissa
40 – Fastuchina
20 – Carrubina
5 – Bargellera
75 – Piattamollisa
105 – Zarbara
3 – Angelica
37 – Enna 2
45 – Ferragnes
77 – Pizzuta Contino
55 – Marra Mennuladi
39 – Fascionello
86 – Sancisuca
102 – Zaccaneddara
74 – Persichina
53 – Lisciannarisa
98 – Tuono
51 – Griddetta
57 – Mennula du nigliu
23 – Cavalera Naro
66 – Mullisa Grande
71 – Nuciddara
87 – Sarbaggia di Sciascia
85 – Romana Licata
44 – Ferraduel
61 – Mirabile
31 – Cupani piccola
18 – Callara
14 – Buscemi
36 – Don Pitrino
101 – Vaiana
10 – Bennici
69 – Naro 1
97 – Tunnulidda
22 – Cavalera Aragona
35 – Don Peppino
60 – Milocca
6 – Bari Flores
79 – Pizzutella
103 – Zagarri'
80 – Pizzuta Grande
B
Concentration, ppbV
0.0
2.5
5.0
7.5
raw
roasted
C
cor = 0.364 p.value = 0
roasted
0
5
10
15
20
25
raw
0
1
2
3
4

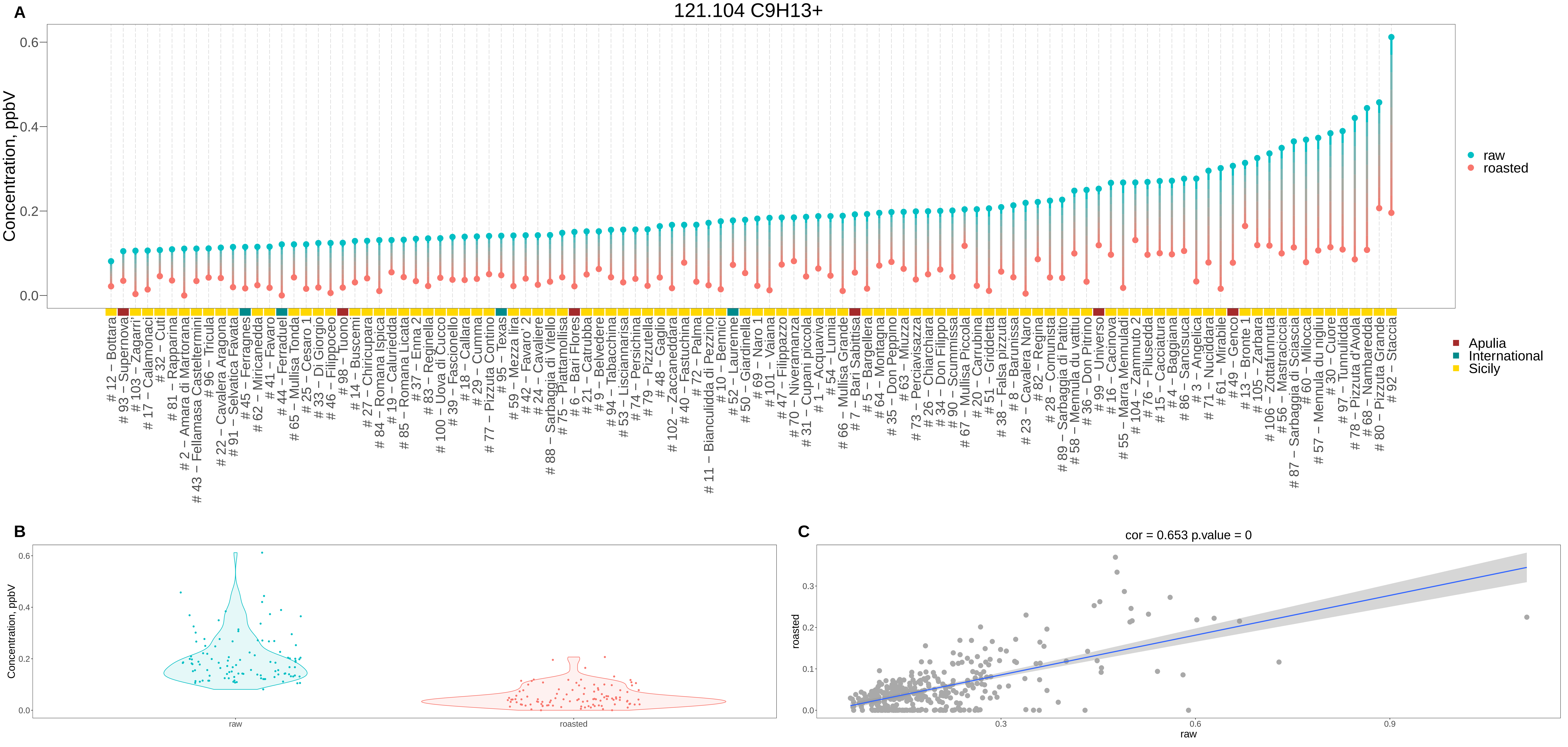
A
121.104 C9H13+
Concentration, ppbV
0.0
0.2
0.4
0.6
raw
roasted
Apulia
International
Sicily
12 – Bottara
93 – Supernova
103 – Zagarri'
17 – Calamonaci
32 – Cuti
81 – Rapparina
2 – Amara di Martorana
43 – Fellamasa Casteltermini
96 – Tricula
22 – Cavalera Aragona
91 – Selvatica Favata
45 – Ferragnes
62 – Miricanedda
41 – Favaro
44 – Ferraduel
65 – Mullisa Tonda
25 – Cesaro 1
33 – Di Giorgio
46 – Filippoceo
98 – Tuono
14 – Buscemi
27 – Chiricupara
84 – Romana Ispica
19 – Caluriedda
85 – Romana Licata
37 – Enna 2
83 – Reginella
100 – Uova di Cucco
39 – Fascionello
18 – Callara
29 – Cumma
77 – Pizzuta Contino
95 – Texas
59 – Mezza lira
42 – Favaro' 2
24 – Cavaliere
88 – Sarbaggia di Vitello
75 – Piattamollisa
6 – Bari Flores
21 – Catrubba
9 – Belvedere
94 – Tabacchina
53 – Lisciannarisa
74 – Persichina
79 – Pizzutella
48 – Gaglio
102 – Zaccaneddara
40 – Fastuchina
72 – Palma
11 – Bianculidda di Pezzino
10 – Bennici
52 – Laurenne
50 – Giardinella
69 – Naro 1
101 – Vaiana
47 – Filippazzo
70 – Niveramanza
31 – Cupani piccola
1 – Acquaviva
54 – Lumia
66 – Mullisa Grande
7 – Bari Sabittisa
5 – Bargellera
64 – Montagna
35 – Don Peppino
63 – Miuzza
73 – Perciavisazza
26 – Chiarchiara
34 – Don Filippo
90 – Scummissa
67 – Mullisa Piccola
20 – Carrubina
51 – Griddetta
38 – Falsa pizzuta
8 – Barunissa
23 – Cavalera Naro
82 – Regina
28 – Comunista
89 – Sarbaggia di Patito
58 – Mennula du vattiu
36 – Don Pitrino
99 – Universo
16 – Cacinova
55 – Marra Mennuladi
104 – Zammuto 2
76 – Pilusedda
15 – Cacciatura
4 – Baggiana
86 – Sancisuca
3 – Angelica
71 – Nuciddara
61 – Mirabile
49 – Genco
13 – Bronte 1
105 – Zarbara
106 – Zottafunnuta
56 – Mastraciccia
87 – Sarbaggia di Sciascia
60 – Milocca
57 – Mennula du nigliu
30 – Cuore
97 – Tunnulidda
78 – Pizzuta d'Avola
68 – Nambaredda
80 – Pizzuta Grande
92 – Staccia
B
Concentration, ppbV
0.0
0.2
0.4
0.6
raw
roasted
C
cor = 0.653 p.value = 0
roasted
0.0
0.1
0.2
0.3
raw
0.3
0.6
0.9

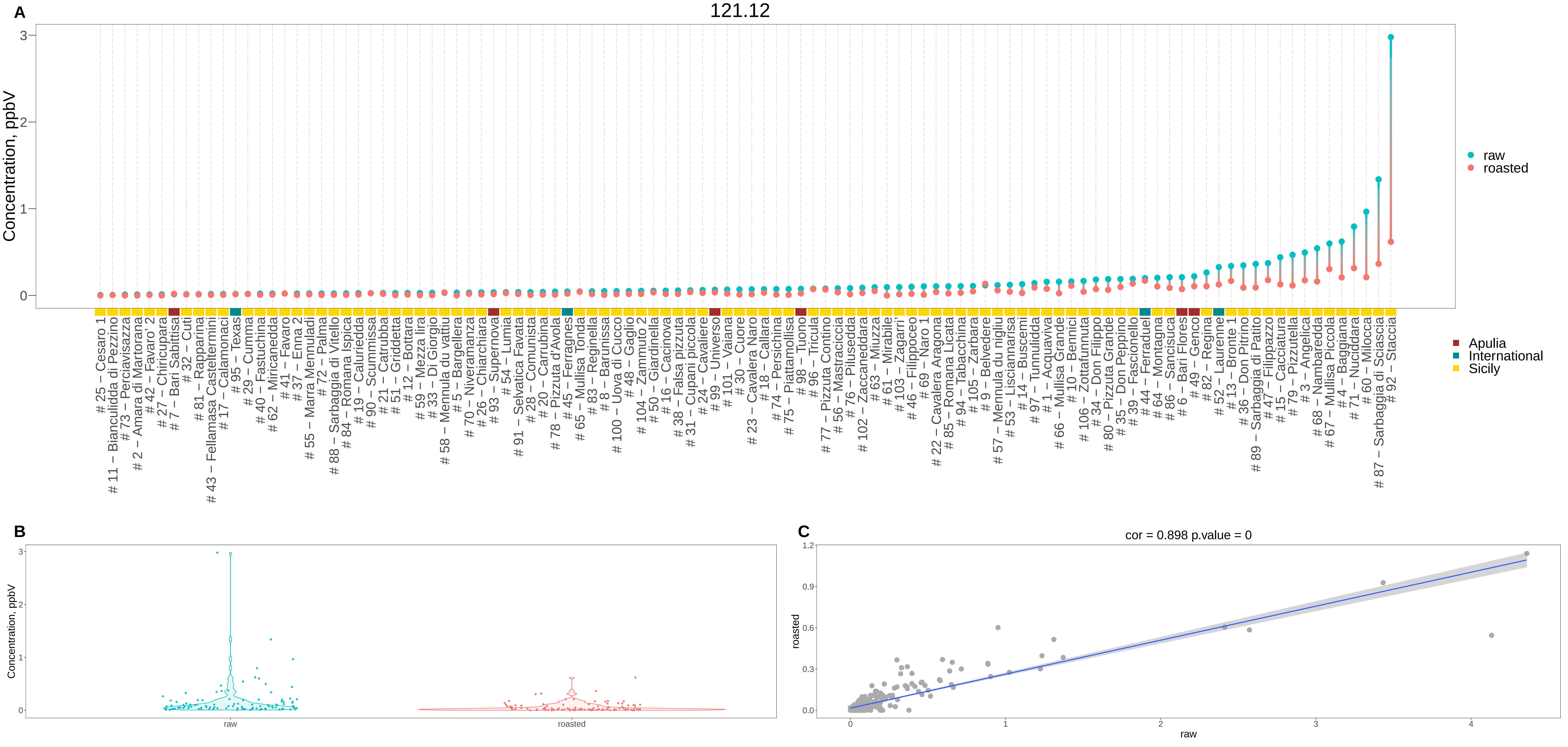
A
121.12
Concentration, ppbV
raw
roasted
Apulia
International
Sicily
B
Concentration, ppbV
raw
roasted
C
cor = 0.898 p.value = 0
roasted
raw

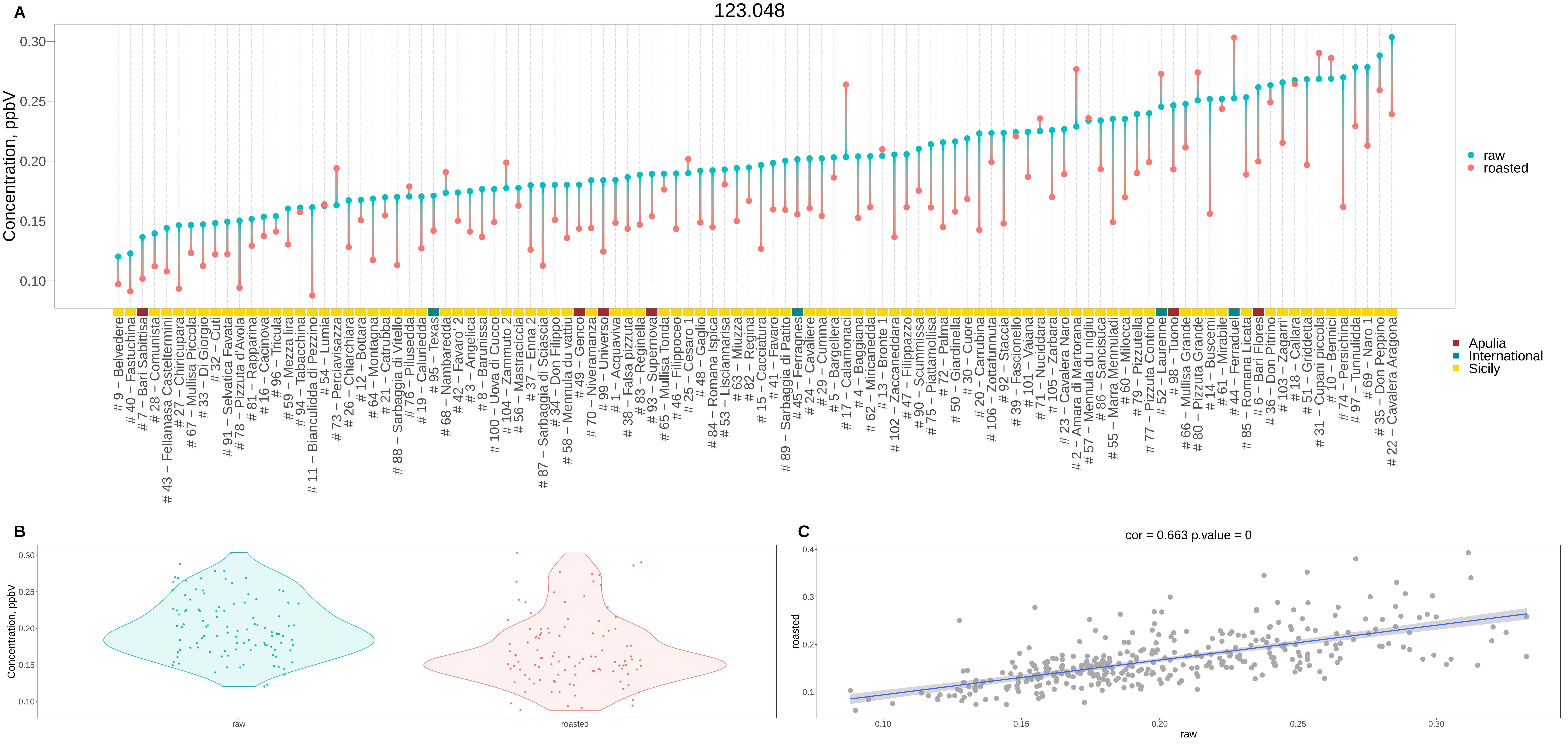

A
123.048
Concentration, ppbV
0.30
0.25
0.20
0.15
0.10
raw
roasted
Apulia
International
Sicily
9 – Belvedere
40 – Fastuchina
7 – Bari Sabittisa
28 – Comunista
43 – Fellamasa Casteltermini
27 – Chiricupara
67 – Mullisa Piccola
33 – Di Giorgio
32 – Cuti
91 – Selvatica Favata
78 – Pizzuta d'Avola
81 – Rapparina
16 – Cacinova
96 – Tricula
59 – Mezza lira
94 – Tabacchina
11 – Bianculidda di Pezzino
54 – Lumia
73 – Perciavisazza
26 – Chiarchiara
12 – Bottara
64 – Montagna
21 – Catrubba
88 – Sarbaggia di Vitello
76 – Piluseddа
19 – Caluriedda
95 – Texas
68 – Nambaredda
42 – Favaro' 2
3 – Angelica
8 – Barunissa
100 – Uova di Cucco
104 – Zammuto 2
56 – Mastraciccia
37 – Enna 2
87 – Sarbaggia di Sciascia
34 – Don Filippo
58 – Mennula du vattiu
49 – Genco
70 – Niveramanza
99 – Universo
1 – Acquaviva
38 – Falsa pizzuta
83 – Reginella
93 – Supernova
65 – Mullisa Tonda
46 – Filippoceo
25 – Cesaro 1
48 – Gaglio
84 – Romana Ispica
53 – Lisciannarisa
63 – Miuzza
82 – Regina
15 – Cacciatura
41 – Favaro
89 – Sarbaggia di Patito
45 – Ferragnes
24 – Cavaliere
29 – Cumma
5 – Bargellera
17 – Calamonaci
4 – Baggiana
62 – Miricanedda
13 – Bronte 1
102 – Zaccaneddara
47 – Filippazzo
90 – Scummissa
75 – Piattamollisa
72 – Palma
50 – Giardinella
30 – Cuore
20 – Carrubina
106 – Zottafunnuta
92 – Staccia
39 – Fascionello
101 – Vaiana
71 – Nuciddara
105 – Zarbara
23 – Cavalera Naro
2 – Amara di Martorana
57 – Mennula di nigliu
86 – Sancisuca
55 – Marra Mennuladi
60 – Milocca
79 – Pizzutella
77 – Pizzuta Contino
52 – Laurenne
98 – Tuono
66 – Mullisa Grande
80 – Pizzuta Grande
14 – Buscemi
61 – Mirabile
44 – Ferraduel
85 – Romana Licata
6 – Bari Flores
36 – Don Pitrino
103 – Zagarri'
18 – Callara
51 – Griddetta
31 – Cupani piccola
10 – Bennici
74 – Persichina
97 – Tunnulidda
69 – Naro 1
35 – Don Peppino
22 – Cavalera Aragona
B
Concentration, ppbV
0.30
0.25
0.20
0.15
0.10
raw
roasted
C
cor = 0.663 p.value = 0
roasted
0.4
0.3
0.2
0.1
0.10
0.15
0.20
0.25
0.30
raw

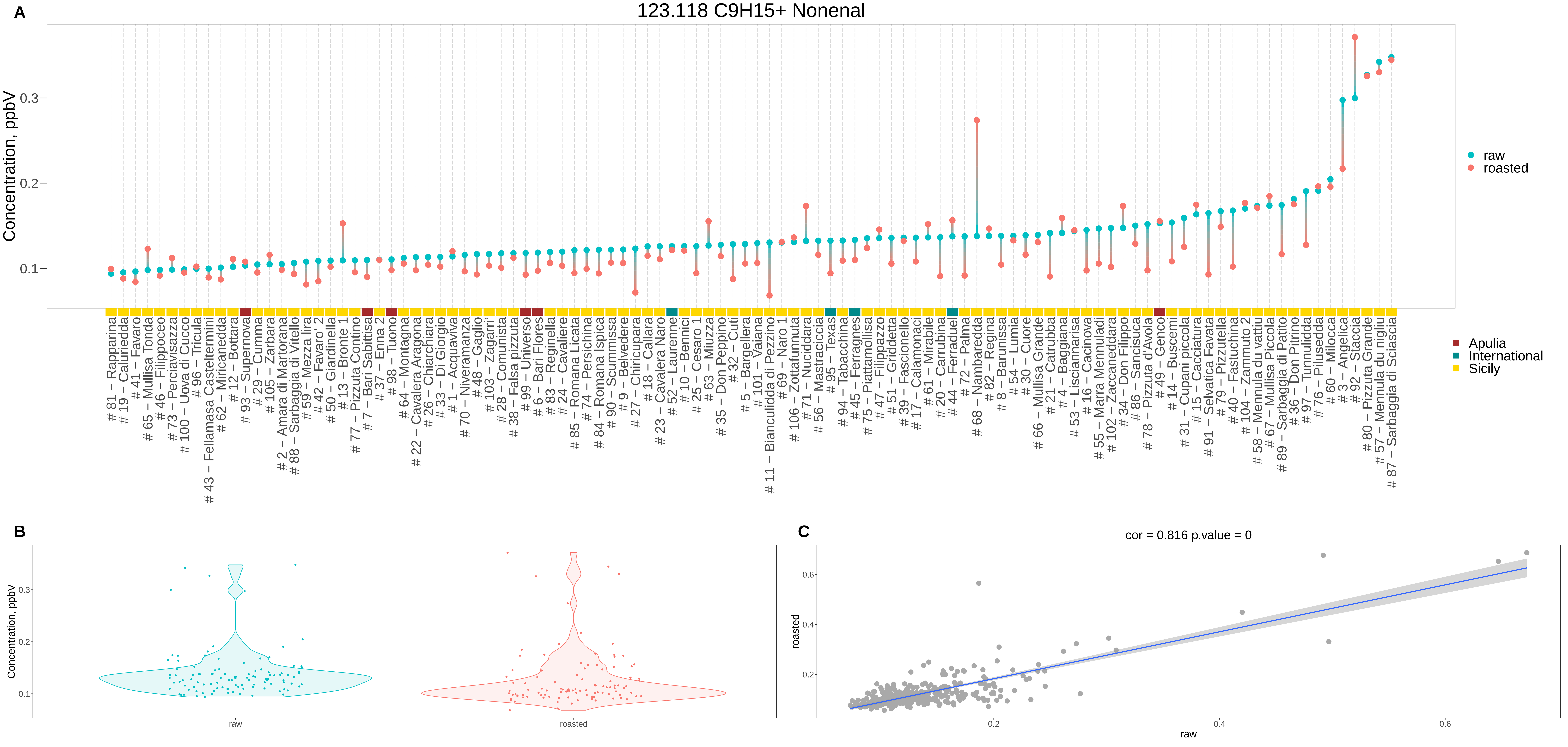

A
123.118 C9H15+ Nonenal
Concentration, ppbV
0.1
0.2
0.3
raw
roasted
Apulia
International
Sicily
81 – Rapparina
19 – Caluriedda
41 – Favaro
65 – Mullisa Tonda
46 – Filippoceo
73 – Perciavisazza
100 – Uova di Cucco
96 – Tricula
43 – Fellamasa Casteltermini
62 – Miricanedda
12 – Bottara
93 – Supernova
29 – Cumma
105 – Zarbara
2 – Amara di Martorana
88 – Sarbaggia di Vitello
59 – Mezza lira
42 – Favaro' 2
50 – Giardinella
13 – Bronte 1
77 – Pizzuta Contino
7 – Bari Sabitisa
37 – Enna 2
98 – Tuono
64 – Montagna
22 – Cavalera Aragona
26 – Chiarchiara
33 – Di Giorgio
1 – Acquaviva
70 – Niveramanza
48 – Gaglio
103 – Zagarri'
28 – Comunista
38 – Falsa pizzuta
99 – Universo
6 – Bari Flores
83 – Reginella
24 – Cavaliere
85 – Romana Licata
74 – Persichina
84 – Romana Ispica
90 – Scummissa
9 – Belvedere
27 – Chiricupara
18 – Callara
23 – Cavalera Naro
52 – Laurenne
10 – Bennici
25 – Cesaro 1
63 – Miuzza
35 – Don Peppino
32 – Cuti
5 – Bargellera
101 – Vaiana
11 – Bianculidda di Pezzino
69 – Naro 1
106 – Zottafunnuta
71 – Nuciddara
56 – Mastraciccia
95 – Texas
94 – Tabacchina
45 – Ferragnes
75 – Piattamollisa
47 – Filippazzo
51 – Griddetta
39 – Fascionello
17 – Calamonaci
61 – Mirabile
20 – Carrubina
44 – Ferraduel
72 – Palma
68 – Nambaredda
82 – Regina
8 – Barunissa
54 – Lumia
30 – Cuore
66 – Mullisa Grande
21 – Catrubba
4 – Baggiana
53 – Liscianarisa
16 – Cacinova
55 – Marra Mennuladi
102 – Zaccaneddara
34 – Don Filippo
86 – Sancisuca
78 – Pizzuta d'Avola
49 – Genco
14 – Buscemi
31 – Cupani piccola
15 – Cacciatura
91 – Selvatica Favata
79 – Pizzutella
40 – Fastuchina
104 – Zammuto 2
58 – Mennula du vattiu
67 – Mullisa Piccola
89 – Sarbaggia di Patito
36 – Don Pitrino
97 – Tunnulidda
76 – Pilusedda
60 – Milocca
3 – Angelica
92 – Staccia
80 – Pizzuta Grande
57 – Mennula du nigliu
87 – Sarbaggia di Sciascia
B
Concentration, ppbV
0.1
0.2
0.3
raw
roasted
C
cor = 0.816 p.value = 0
roasted
0.2
0.4
0.6
raw
0.2
0.4
0.6

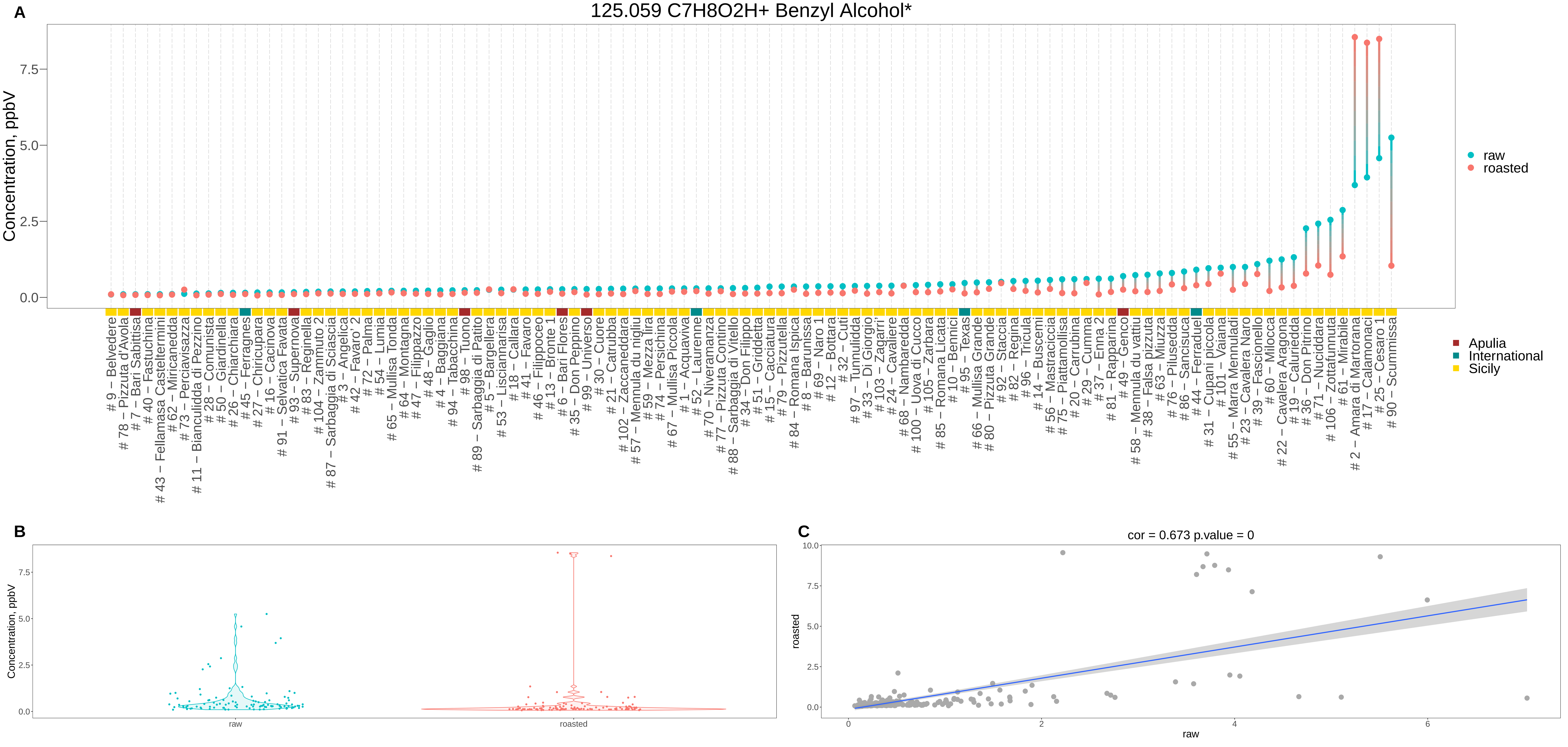

A
125.059 C7H8O2H+ Benzyl Alcohol*
Concentration, ppbV
0.0
2.5
5.0
7.5
raw
roasted
Apulia
International
Sicily
9 – Belvedere
78 – Pizzuta d'Avola
7 – Bari Sabittisa
40 – Fastuchina
43 – Fellamasa Casteltermini
62 – Miricanedda
73 – Perciavisazza
11 – Biancuilidda di Pezzino
28 – Comunista
50 – Giardinella
26 – Chiarchiara
45 – Ferragnes
27 – Chiricupara
16 – Cacinova
91 – Selvatica Favata
93 – Supernova
83 – Reginella
104 – Zammuto 2
87 – Sarbaggia di Sciascia
3 – Angelica
42 – Favaro' 2
72 – Palma
54 – Lumia
65 – Mullisa Tonda
64 – Montagna
47 – Filippazzo
48 – Gaglio
4 – Baggiana
94 – Tabacchina
98 – Tuono
89 – Sarbaggia di Patito
5 – Bargellera
53 – Lisciannarisa
18 – Callara
41 – Favaro
46 – Filippoceo
13 – Bronte 1
6 – Bari Flores
35 – Don Peppino
99 – Universo
30 – Cuore
21 – Catrubba
102 – Zaccaneddara
57 – Mennula du nigliu
59 – Mezza lira
74 – Persichina
67 – Mullisa Piccola
1 – Acquaviva
52 – Laurenne
70 – Niveramanza
77 – Pizzuta Contino
88 – Sarbaggia di Vitello
34 – Don Filippo
51 – Griddetta
15 – Cacciatura
79 – Pizzutella
84 – Romana Ispica
8 – Barunissa
69 – Naro 1
12 – Bottara
32 – Cuti
97 – Tunnulidda
33 – Di Giorgio
103 – Zagarri'
24 – Cavaliere
68 – Nambaredda
100 – Uova di Cucco
105 – Zarbara
85 – Romana Licata
10 – Bennici
95 – Texas
66 – Mullisa Grande
80 – Pizzuta Grande
92 – Staccia
82 – Regina
96 – Tricula
14 – Buscemi
56 – Mastraciccia
75 – Piattamollisa
20 – Carrubina
29 – Cumma
37 – Enna 2
81 – Rapparina
49 – Genco
58 – Mennula du vattiu
38 – Falsa pizzuta
63 – Miuzza
76 – Pilusedda
86 – Sancisuca
44 – Ferraduel
31 – Cupani piccola
101 – Vaiana
55 – Marra Mennuladi
23 – Cavalera Naro
39 – Fascionello
60 – Milocca
22 – Cavalera Aragona
19 – Caluriedda
36 – Don Pitrino
71 – Nuciddara
106 – Zottafunnuta
61 – Mirabile
2 – Amara di Martorana
17 – Calamonaci
25 – Cesaro 1
90 – Scummissa
B
Concentration, ppbV
raw
roasted
C
cor = 0.673 p.value = 0
roasted
raw
0
2
4
6
0.0
2.5
5.0
7.5
10.0

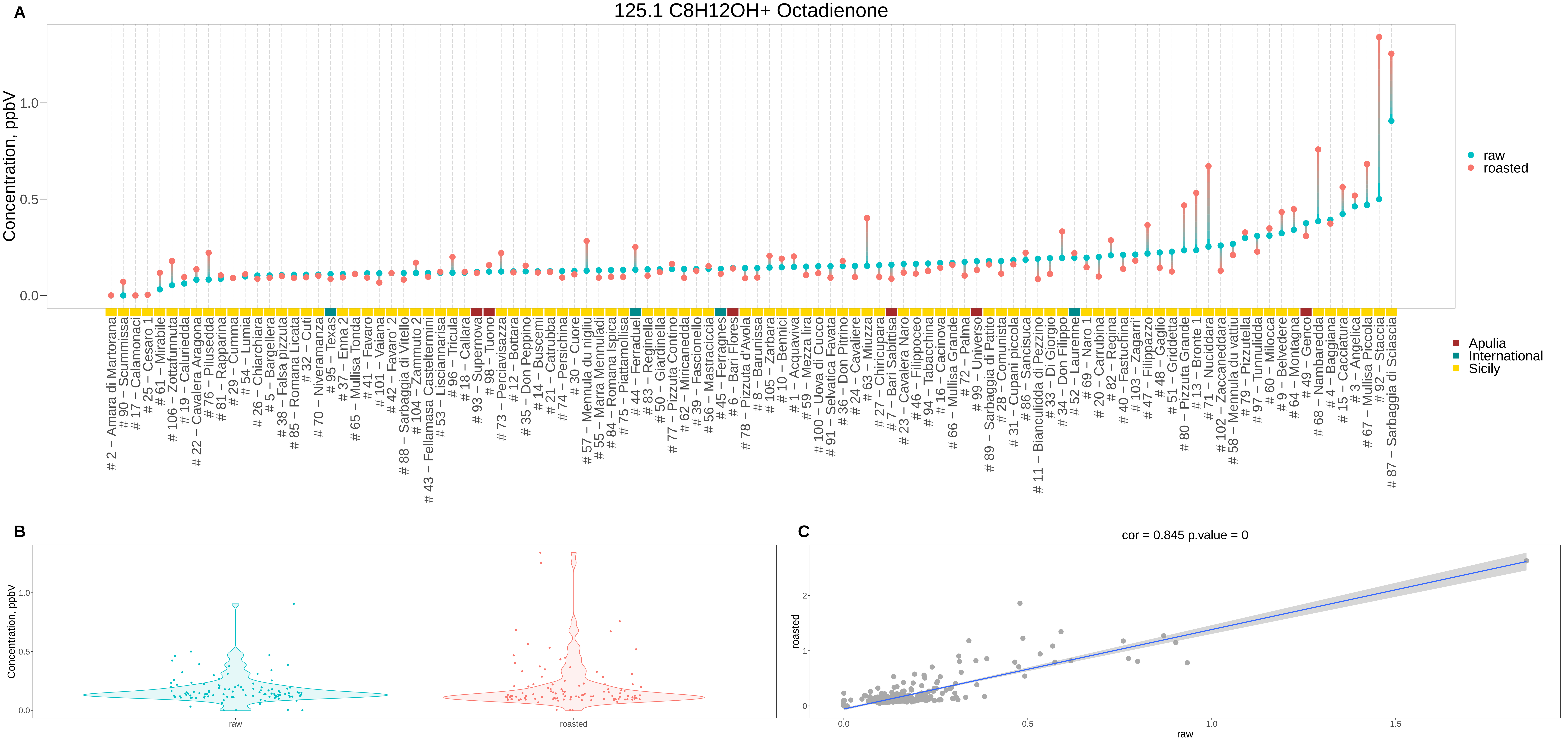
A
125.1 C8H12OH+ Octadienone
Concentration, ppbV
0.0
0.5
1.0
raw
roasted
Apulia
International
Sicily
2 – Amara di Martorana
90 – Scummissa
17 – Calamonaci
25 – Cesaro 1
61 – Mirabile
106 – Zottafunnuta
19 – Caluriedda
22 – Cavalera Aragona
76 – Pilusedda
81 – Rapparina
29 – Cumma
54 – Lumia
26 – Chiarchiara
5 – Bargellera
38 – Falsa pizzuta
85 – Romana Licata
32 – Cuti
70 – Niveramanza
95 – Texas
37 – Enna 2
65 – Mullisa Tonda
41 – Favaro
101 – Vaiana
42 – Favaro' 2
88 – Sarbaggia di Vitello
104 – Zammuto 2
43 – Fellamasa Casteltermini
53 – Liscianarisa
96 – Tricula
18 – Callara
93 – Supernova
98 – Tuono
73 – Perciavisazza
12 – Bottara
35 – Don Peppino
14 – Buscemi
21 – Catrubba
74 – Persichina
30 – Cuore
57 – Mennula du nigliu
55 – Marra Mennuladi
84 – Romana Ispica
75 – Piattamollisa
44 – Ferraduel
83 – Reginella
50 – Giardinella
77 – Pizzuta Contino
62 – Miricanedda
39 – Fascionello
56 – Mastraciccia
45 – Ferragnes
6 – Bari Flores
78 – Pizzuta d'Avola
8 – Barunissa
105 – Zarbara
10 – Bennici
1 – Acquaviva
59 – Mezza lira
100 – Uova di Cucco
91 – Selvatica Favata
36 – Don Pitrino
24 – Cavaliere
63 – Miuzza
27 – Chiricupara
7 – Bari Sabittisa
23 – Cavalera Naro
46 – Filippoceo
94 – Tabacchina
16 – Cacinova
66 – Mullisa Grande
72 – Palma
99 – Universo
89 – Sarbaggia di Patito
28 – Comunista
31 – Cupani piccola
86 – Sancisuca
11 – Bianculidda di Pezzino
33 – Di Giorgio
34 – Don Filippo
52 – Laurenne
69 – Naro 1
20 – Carrubina
82 – Regina
40 – Fastuchina
103 – Zagarri'
47 – Filippazzo
48 – Gaglio
51 – Griddetta
80 – Pizzuta Grande
13 – Bronte 1
71 – Nuciddara
102 – Zaccaneddara
58 – Mennula du vattiu
79 – Pizzutella
97 – Tunnulidda
60 – Milocca
9 – Belvedere
64 – Montagna
49 – Genco
68 – Nambaredda
4 – Baggiana
15 – Cacciatura
3 – Angelica
67 – Mullisa Piccola
92 – Staccia
87 – Sarbaggia di Sciascia
B
Concentration, ppbV
0.0
0.5
1.0
raw
roasted
C
cor = 0.845 p.value = 0
roasted
0
1
2
0.0
0.5
1.0
1.5
raw

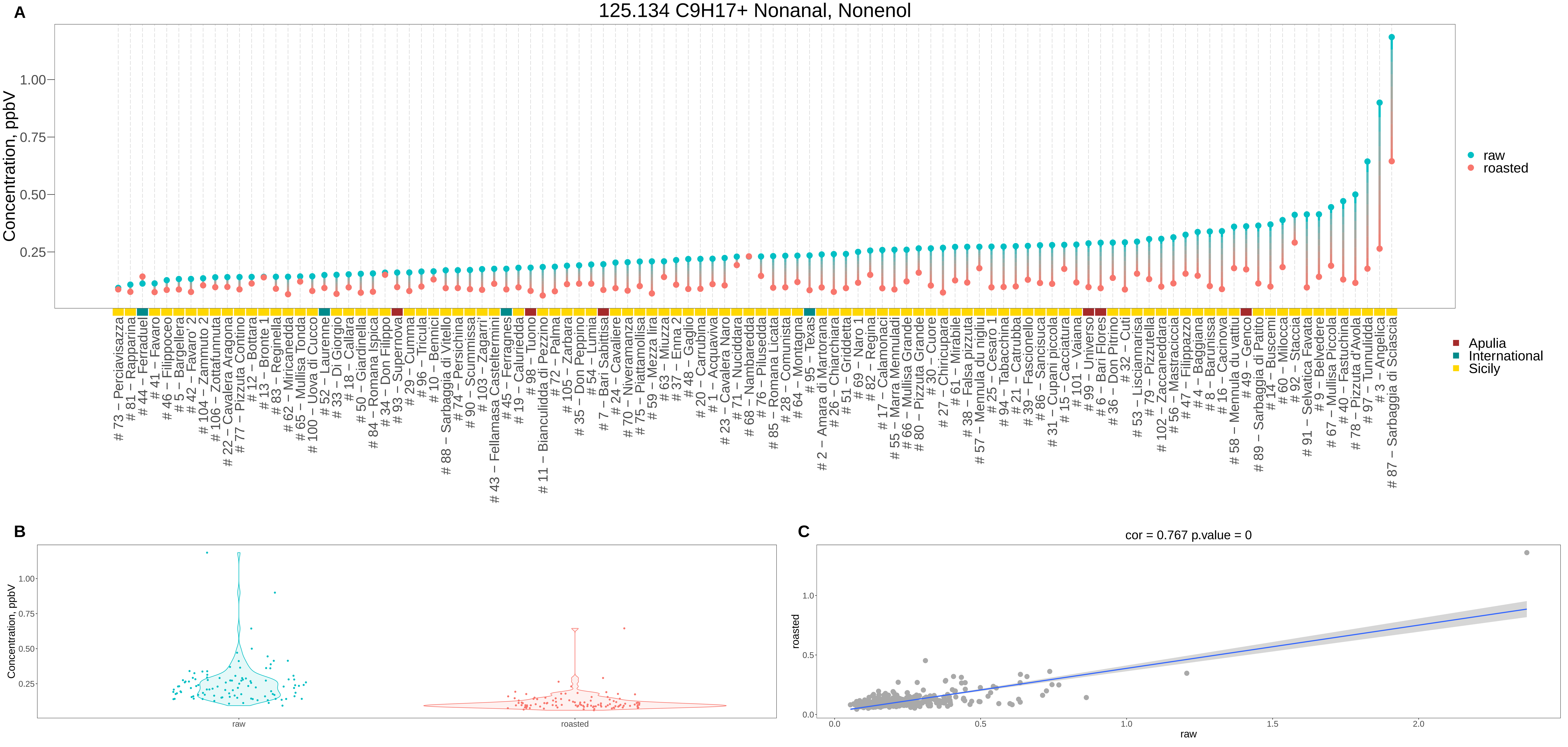
A
125.134 C9H17+ Nonanal, Nonenol
Concentration, ppbV
raw
roasted
Apulia
International
Sicily
B
Concentration, ppbV
raw
roasted
C
cor = 0.767 p.value = 0
roasted
raw

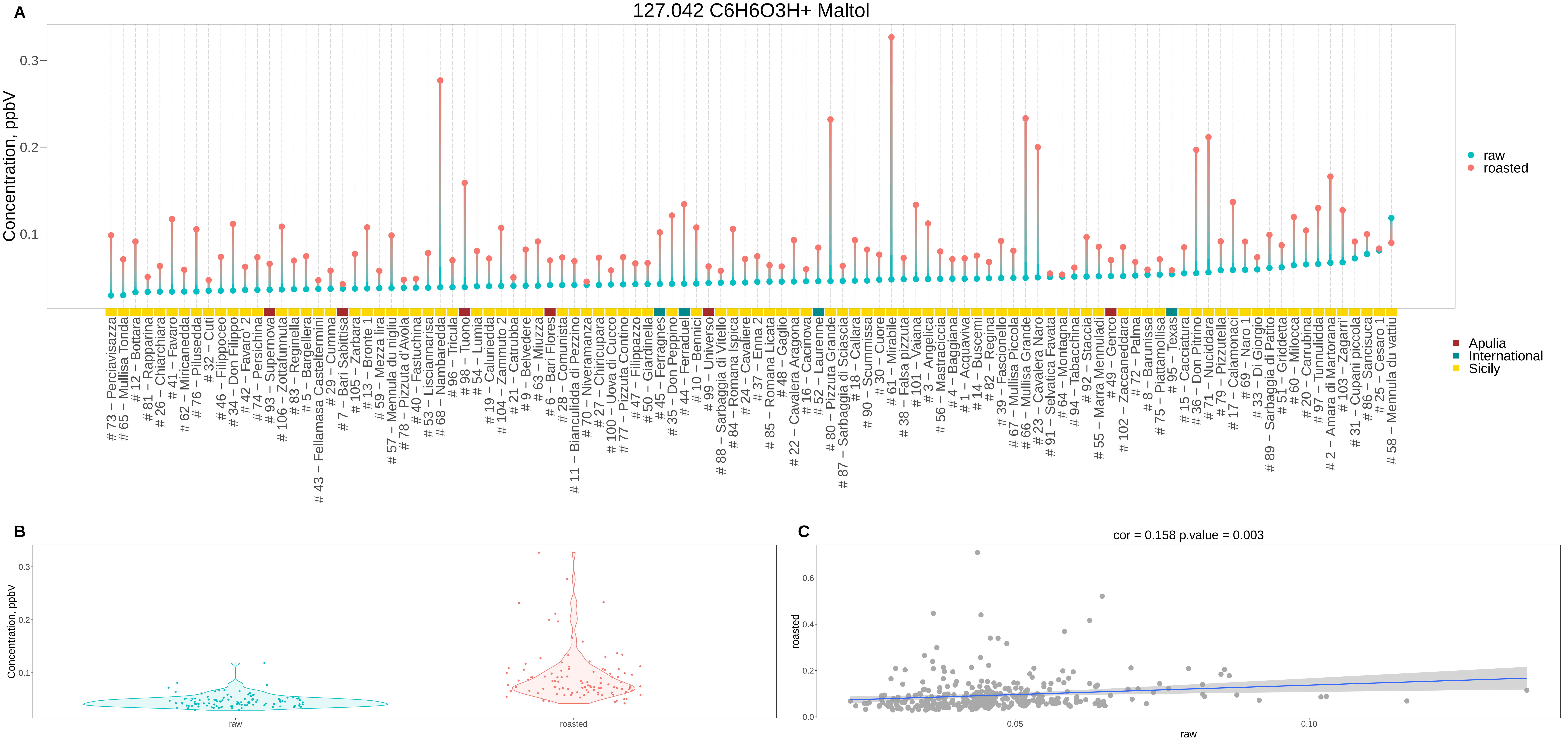

A
127.042 C6H6O3H+ Maltol
Concentration, ppbV
0.1
0.2
0.3
raw
roasted
Apulia
International
Sicily
73 – Perciavisazza
65 – Mullisa Tonda
12 – Bottara
81 – Rapparina
26 – Chiarchiara
41 – Favaro
62 – Miricanedda
76 – Pilusedda
32 – Cuti
46 – Filippoceo
34 – Don Filippo
42 – Favaro 2
74 – Persichina
93 – Supernova
106 – Zottafunnuta
83 – Reginella
5 – Bargellera
43 – Fellamasa Casteltermini
29 – Cumma
7 – Bari Sabittisa
105 – Zarbara
13 – Bronte 1
59 – Mezza lira
57 – Mennula du nigliu
78 – Pizzuta d'Avola
40 – Fastuchina
53 – Lisciannarisa
68 – Nambaredda
96 – Tricula
98 – Tuono
54 – Lumia
19 – Caluriedda
104 – Zammuto 2
21 – Catrubba
9 – Belvedere
63 – Miuzza
6 – Bari Flores
28 – Comunista
11 – Bianculidda di Pezzino
70 – Niveramanza
27 – Chiricupara
100 – Uova di Cucco
77 – Pizzuta Contino
47 – Filippazzo
50 – Giardinella
45 – Ferragnes
35 – Don Peppino
44 – Ferraduel
10 – Bennici
99 – Universo
88 – Sarbaggia di Vitello
84 – Romana Ispica
24 – Cavaliere
37 – Enna 2
85 – Romana Licata
48 – Gaglio
22 – Cavalera Aragona
16 – Cacinova
52 – Laurenne
80 – Pizzuta Grande
87 – Sarbaggia di Sciascia
18 – Callara
90 – Scummissa
30 – Cuore
61 – Mirabile
38 – Falsa pizzuta
101 – Vaiana
3 – Angelica
56 – Mastraciccia
4 – Baggiana
1 – Acquaviva
14 – Buscemi
82 – Regina
39 – Fascionello
67 – Mullisa Piccola
66 – Mullisa Grande
23 – Cavalera Naro
91 – Selvatica Favata
64 – Montagna
94 – Tabacchina
92 – Staccia
55 – Marra Mennuladi
49 – Genco
102 – Zaccaneddara
72 – Palma
8 – Barunissa
75 – Piattamollisa
95 – Texas
15 – Cacciatura
36 – Don Pitrino
71 – Nuciddara
79 – Pizzutella
17 – Calamonaci
69 – Naro 1
33 – Di Giorgio
89 – Sarbaggia di Patito
51 – Griddetta
60 – Milocca
20 – Carrubina
97 – Tunnulidda
2 – Amara di Martorana
103 – Zagarri'
31 – Cupani piccola
86 – Sancisuca
25 – Cesaro 1
58 – Mennula du vattiu
B
Concentration, ppbV
0.1
0.2
0.3
raw
roasted
C
cor = 0.158 p.value = 0.003
roasted
0.0
0.2
0.4
0.6
raw
0.05
0.10

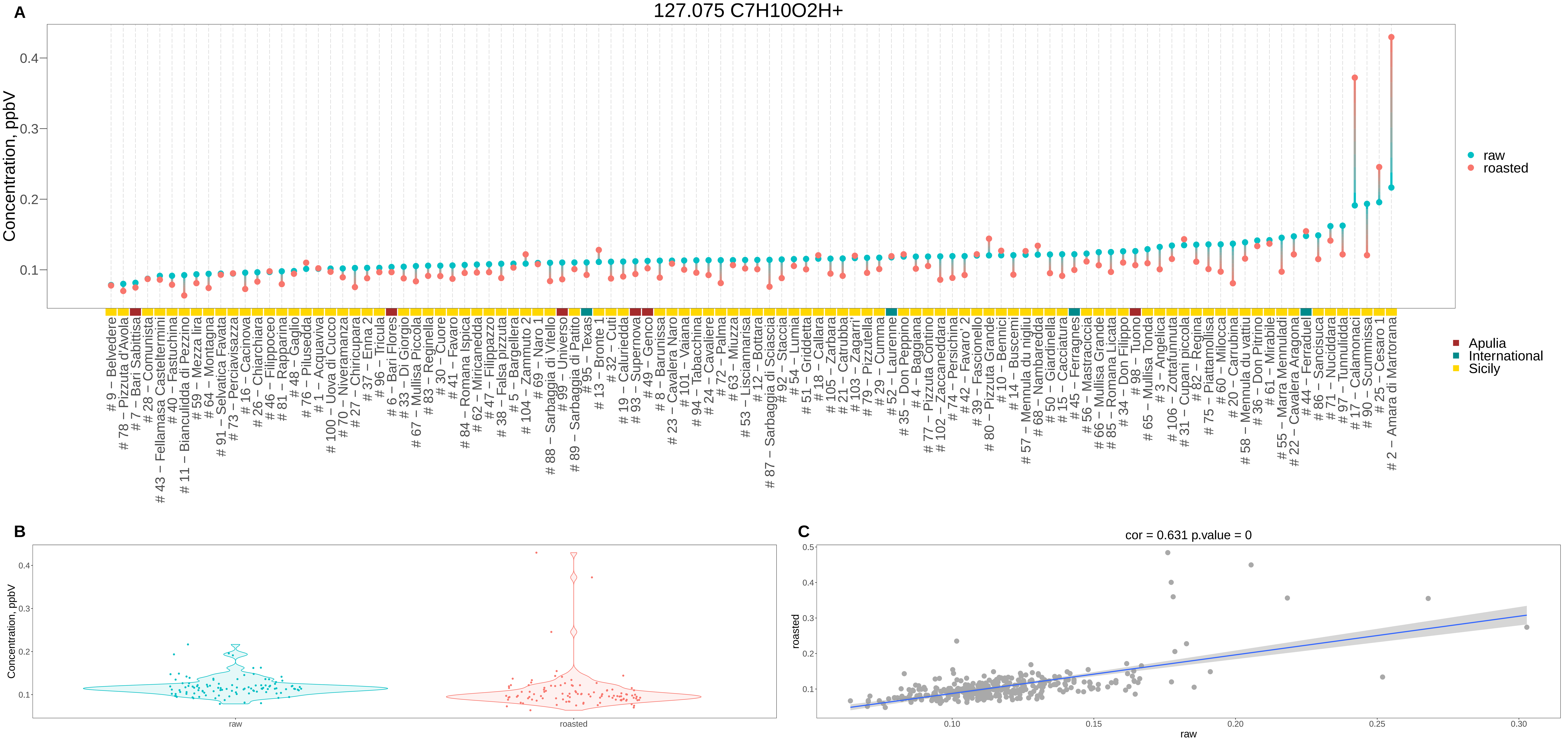
A
127.075 C7H10O2H+
Concentration, ppbV
0.1
0.2
0.3
0.4
raw
roasted
Apulia
International
Sicily
9 – Belvedere
78 – Pizzuta d'Avola
7 – Bari Sabitissa
28 – Comunista
43 – Fellamasa Casteltermini
40 – Fastuchina
11 – Bianculidda di Pezzino
59 – Mezza lira
64 – Montagna
91 – Selvatica Favata
73 – Perciavisazza
16 – Cacinova
26 – Chiarchiara
46 – Filippoceo
81 – Rapparina
48 – Gaglio
76 – Piluseddа
1 – Acquaviva
100 – Uova di Cucco
70 – Niveramanza
27 – Chiricupara
37 – Enna 2
96 – Tricula
6 – Bari Flores
33 – Di Giorgio
67 – Mullisa Piccola
83 – Reginella
30 – Cuore
41 – Favaro
84 – Romana Ispica
62 – Miricanedda
47 – Filippazzo
38 – Falsa pizzuta
5 – Bargellera
104 – Zammuto 2
69 – Naro 1
88 – Sarbaggia di Vitello
99 – Universo
89 – Sarbaggia di Patito
95 – Texas
13 – Bronte 1
32 – Cuti
19 – Caluriedda
93 – Supernova
49 – Genco
8 – Barunissa
23 – Cavalera Naro
101 – Vaiana
94 – Tabacchina
24 – Cavaliere
72 – Palma
63 – Miuzza
53 – Liscianarisa
12 – Bottara
87 – Sarbaggia di Sciascia
92 – Staccia
54 – Lumia
51 – Griddetta
18 – Callara
105 – Zarbara
21 – Catrubba
103 – Zagarri'
79 – Pizzutella
29 – Cumma
52 – Laurenne
35 – Don Peppino
4 – Baggiana
77 – Pizzuta Contino
102 – Zaccaneddara
74 – Persichina
42 – Favaro 2
39 – Fascionello
80 – Pizzuta Grande
10 – Bennici
14 – Buscemi
57 – Mennula du nigliu
68 – Nambaredda
50 – Giardinella
15 – Cacciatura
45 – Ferragnes
56 – Mastraciccia
66 – Mullisa Grande
85 – Romana Licata
34 – Don Filippo
98 – Tuono
65 – Mullisa Tonda
3 – Angelica
106 – Zottafunnuta
31 – Cupani piccola
82 – Regina
75 – Piattamollisa
60 – Milocca
20 – Carrubina
58 – Mennula du vattiu
36 – Don Pitrino
61 – Mirabile
55 – Marra Mennuladi
22 – Cavalera Aragona
44 – Ferraduel
86 – Sancisuca
71 – Nuciddara
97 – Tunnulidda
17 – Calamonaci
90 – Scummissa
25 – Cesaro 1
2 – Amara di Martorana
B
Concentration, ppbV
0.1
0.2
0.3
0.4
raw
roasted
C
cor = 0.631 p.value = 0
roasted
0.1
0.2
0.3
0.4
0.5
raw
0.10
0.15
0.20
0.25
0.30

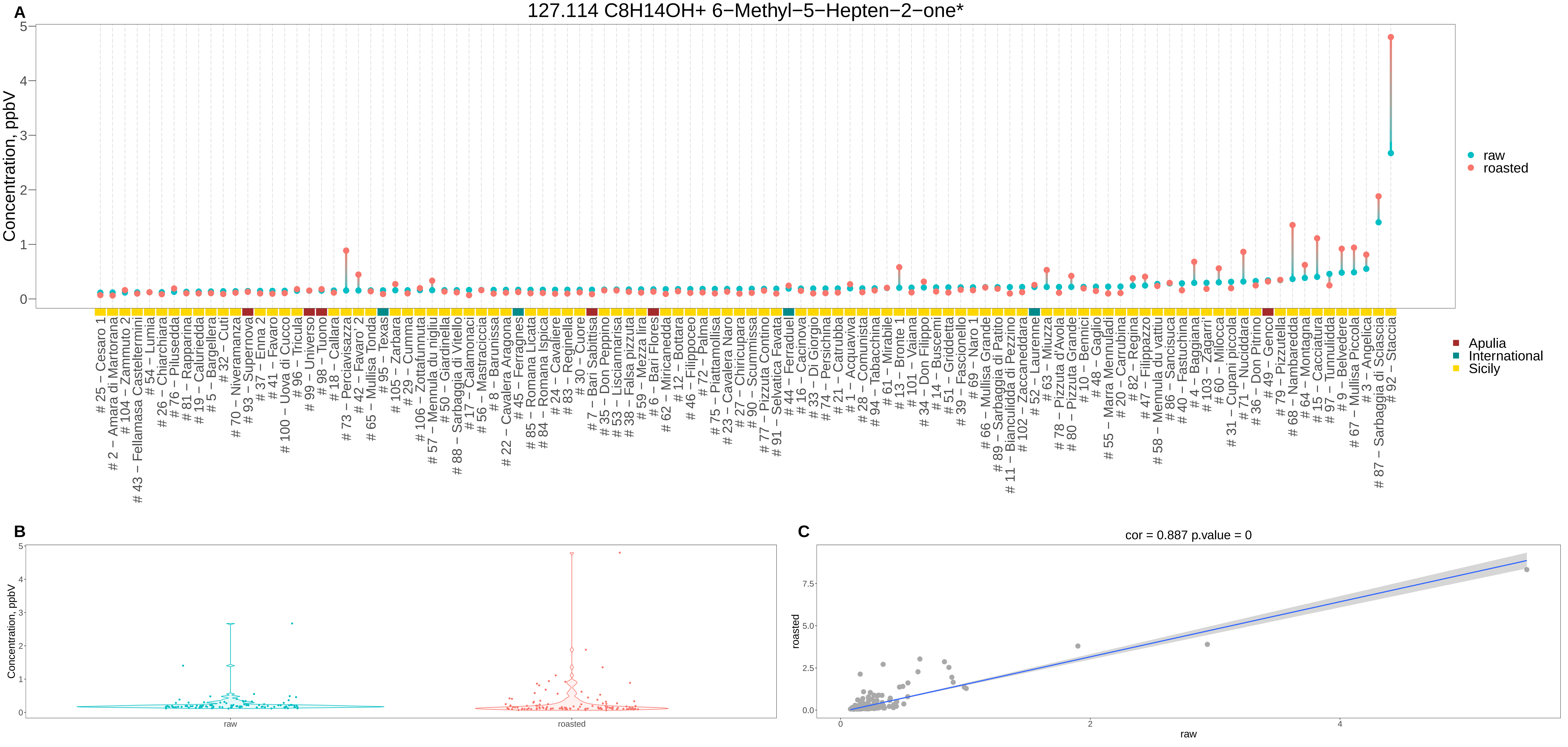

A
127.114 C8H14OH+ 6−Methyl−5−Hepten−2−one*
Concentration, ppbV
0
1
2
3
4
5
raw
roasted
Apulia
International
Sicily
25 − Cesaro 1
2 − Amara di Martorana
104 − Zammuto 2
43 − Fellamasa Casteltermini
54 − Lumia
26 − Chiarchiara
76 − Pilusedda
81 − Rapparina
19 − Caluriedda
5 − Bargellera
32 − Cuti
70 − Niveramanza
93 − Supernova
37 − Enna 2
41 − Favaro
100 − Uova di Cucco
96 − Tricula
99 − Universo
98 − Tuono
18 − Callara
73 − Perciavisazza
42 − Favaro' 2
65 − Mullisa Tonda
95 − Texas
105 − Zarbara
29 − Cumma
106 − Zottafunnuta
57 − Mennula du nigliu
50 − Giardinella
88 − Sarbaggia di Vitello
17 − Calamonaci
56 − Mastraciccia
8 − Barunissa
22 − Cavalera Aragona
45 − Ferragnes
85 − Romana Licata
84 − Romana Ispica
24 − Cavaliere
83 − Reginella
30 − Cuore
7 − Bari Sabittisa
35 − Don Peppino
53 − Liscianarisa
38 − Falsa pizzuta
59 − Mezza lira
6 − Bari Flores
62 − Miricanedda
12 − Bottara
46 − Filippoceo
72 − Palma
75 − Piattamollisa
23 − Cavalera Naro
27 − Chiricupara
90 − Scummissa
77 − Pizzuta Contino
91 − Selvatica Favata
44 − Ferraduel
16 − Cacinova
33 − Di Giorgio
74 − Persichina
21 − Catrubba
1 − Acquaviva
28 − Comunista
94 − Tabacchina
61 − Mirabile
13 − Bronte 1
101 − Vaiana
34 − Don Filippo
14 − Buscemi
51 − Griddetta
39 − Fascionello
69 − Naro 1
66 − Mullisa Grande
89 − Sarbaggia di Patito
11 − Bianculidda di Pezzino
102 − Zaccaneddara
52 − Laurenne
63 − Miuzza
78 − Pizzuta d'Avola
80 − Pizzuta Grande
10 − Bennici
48 − Gaglio
55 − Marra Mennuladi
20 − Carrubina
82 − Regina
47 − Filippazzo
58 − Mennula du vattiu
86 − Sancisuca
40 − Fastuchina
4 − Baggiana
103 − Zagarri'
60 − Milocca
31 − Cupani piccola
71 − Nucidara
36 − Don Pitrino
49 − Genco
79 − Pizzutella
68 − Nambaredda
64 − Montagna
15 − Cacciatura
97 − Tunnulidda
9 − Belvedere
67 − Mullisa Piccola
3 − Angelica
87 − Sarbaggia di Sciascia
92 − Staccia
B
Concentration, ppbV
0
1
2
3
4
5
raw
roasted
C
cor = 0.887 p.value = 0
roasted
0.0
2.5
5.0
7.5
raw
0
2
4

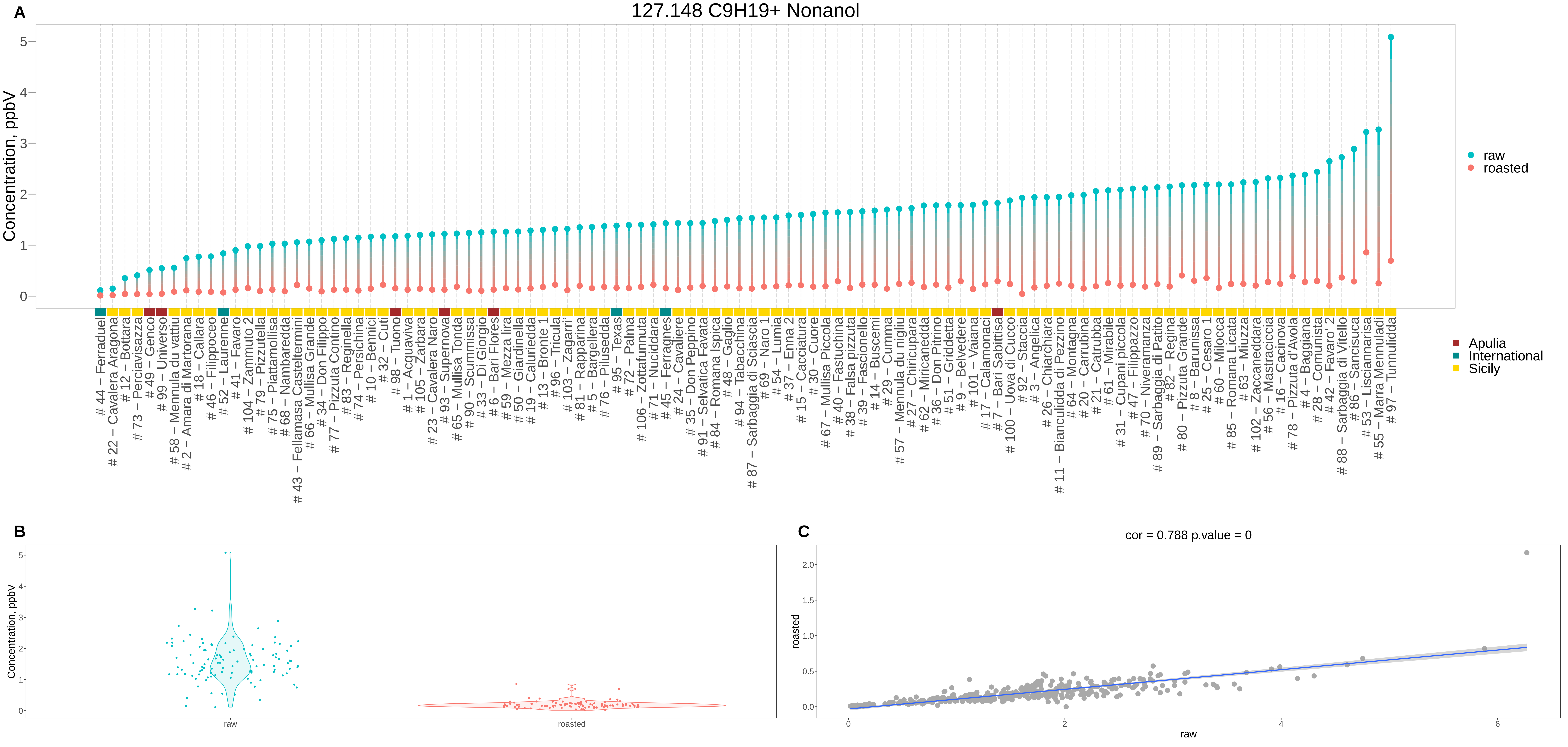
A
127.148 C9H19+ Nonanol
Concentration, ppbV
0
1
2
3
4
5
raw
roasted
Apulia
International
Sicily
44 – Ferraduel
22 – Cavalera Aragona
12 – Bottara
73 – Perciavisazza
49 – Genco
99 – Universo
58 – Mennula du vattiu
2 – Amara di Martorana
18 – Callara
46 – Filippoceo
52 – Laurenne
41 – Favaro
104 – Zammuto 2
79 – Pizzutella
75 – Piattamollisa
68 – Nambaredda
43 – Fellamasa Casteltermini
66 – Mullisa Grande
34 – Don Filippo
77 – Pizzuta Contino
83 – Reginella
74 – Persichina
10 – Bennici
32 – Cuti
98 – Tuono
1 – Acquaviva
105 – Zarbara
23 – Cavalera Naro
93 – Supernova
65 – Mullisa Tonda
90 – Scummissa
33 – Di Giorgio
6 – Bari Flores
59 – Mezza lira
50 – Giardinella
19 – Caluriedda
13 – Bronte 1
96 – Tricula
103 – Zagarri'
81 – Rappariina
5 – Bargellera
76 – Pilusedda
95 – Texas
72 – Palma
106 – Zottafunnuta
71 – Nuciddara
45 – Ferragnes
24 – Cavaliere
35 – Don Peppino
91 – Selvatica Favata
84 – Romana Ispica
48 – Gaglio
94 – Tabacchina
87 – Sarbaggia di Sciascia
69 – Naro 1
54 – Lumia
37 – Enna 2
15 – Cacciatura
30 – Cuore
67 – Mullisa Piccola
40 – Fastuchina
38 – Falsa pizzuta
39 – Fascionello
14 – Buscemi
29 – Cumma
57 – Mennula du nigliu
27 – Chiricupara
62 – Miricanedda
36 – Don Pitrino
51 – Griddetta
9 – Belvedere
101 – Vaiana
17 – Calamonaci
7 – Bari Sabittisa
100 – Uova di Cucco
92 – Staccia
3 – Angelica
26 – Chiarchiara
11 – Biancuiddia di Pezzino
64 – Montagna
20 – Carrubina
21 – Catrubba
61 – Mirabile
31 – Cupani piccola
47 – Filippazzo
70 – Niveramanza
89 – Sarbaggia di Patito
82 – Regina
80 – Pizzuta Grande
8 – Barunissa
25 – Cesaro 1
60 – Milocca
85 – Romana Licata
63 – Miuzza
102 – Zaccaneddara
56 – Mastraciccia
16 – Cacinova
78 – Pizzuta d'Avola
4 – Baggiana
28 – Comunista
42 – Favaro' 2
88 – Sarbaggia di Vitello
86 – Sancisuca
53 – Liscіannarisa
55 – Marra Mennuladi
97 – Tunnulidda
B
Concentration, ppbV
raw
roasted
C
cor = 0.788 p.value = 0
roasted
raw
0.0
0.5
1.0
1.5
2.0
0
2
4
6

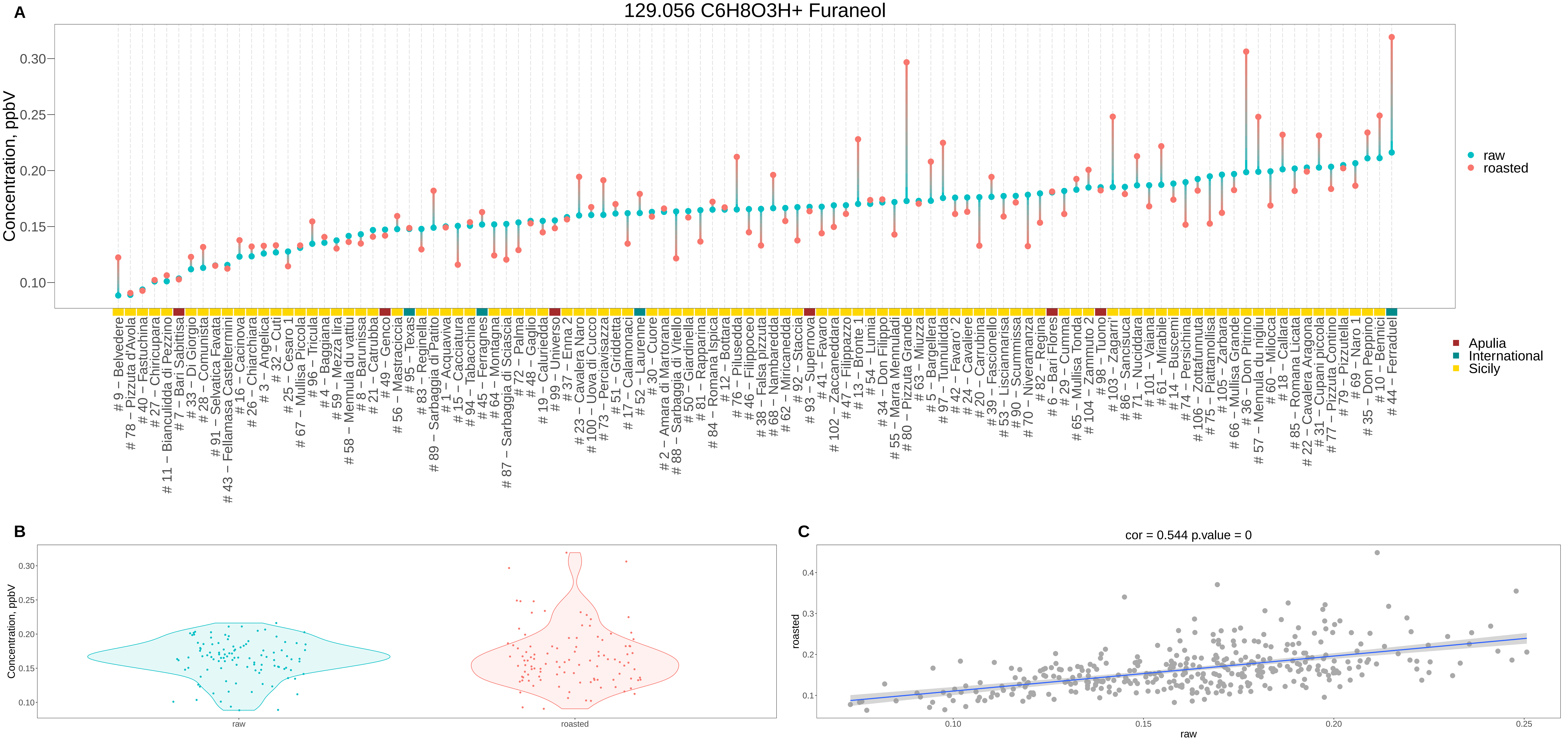
A
129.056 C6H8O3H+ Furaneol
Concentration, ppbV
0.30
0.25
0.20
0.15
0.10
raw
roasted
Apulia
International
Sicily
9 – Belvedere
78 – Pizzuta d'Avola
40 – Fastuchina
27 – Chiricupara
11 – Bianculidda di Pezzino
7 – Bari Sabitisa
33 – Di Giorgio
28 – Comunista
91 – Selvatica Favata
43 – Fellamasa Casteltermini
16 – Cacinova
26 – Chiarchiara
3 – Angelica
32 – Cuti
25 – Cesaro 1
67 – Mullisa Piccola
96 – Tricula
4 – Baggiana
59 – Mezza lira
58 – Mennula du vattiu
8 – Barunissa
21 – Catrubba
49 – Genco
56 – Mastracicia
95 – Texas
83 – Reginella
89 – Sarbaggia di Patito
1 – Acquaviva
15 – Cacciatura
94 – Tabacchina
45 – Ferragnes
64 – Montagna
87 – Sarbaggia di Sciascia
72 – Palma
48 – Gaglio
19 – Caluriedda
99 – Universo
37 – Enna 2
23 – Cavalera Naro
100 – Uova di Cucco
73 – Perciavisazza
51 – Griddetta
17 – Calamonaci
52 – Laurenne
30 – Cuore
2 – Amara di Martorana
88 – Sarbaggia di Vitello
50 – Giardinella
81 – Rapparina
84 – Romana Ispica
12 – Bottara
76 – Pilusedda
46 – Filippoceo
38 – Falsa pizzuta
68 – Nambaredda
62 – Miricanedda
92 – Staccia
93 – Supernova
41 – Favaro
102 – Zaccaneddara
47 – Filippazzo
13 – Bronte 1
54 – Lumia
34 – Don Filippo
55 – Marra Mennuladi
80 – Pizzuta Grande
63 – Miuzza
5 – Bargellera
97 – Tunnulidda
42 – Favaro' 2
24 – Cavaliere
20 – Carrubina
39 – Fascionello
53 – Lisciannarisa
90 – Scummissa
70 – Niveramanza
82 – Regina
6 – Bari Flores
29 – Cumma
65 – Mullisa Tonda
104 – Zammuto 2
98 – Tuono
103 – Zagarri'
86 – Sancisuca
71 – Nuciddara
101 – Vaiana
61 – Mirabile
14 – Buscemi
74 – Persichina
106 – Zottafunnuta
75 – Piattamollisa
105 – Zarbara
66 – Mullisa Grande
36 – Don Pitrino
57 – Mennula du nigliu
60 – Milocca
18 – Callara
85 – Romana Licata
22 – Cavalera Aragona
31 – Cupani piccola
77 – Pizzuta Contino
79 – Pizzutella
69 – Naro 1
35 – Don Peppino
10 – Bennici
44 – Ferraduel
B
Concentration, ppbV
0.30
0.25
0.20
0.15
0.10
raw
roasted
C
cor = 0.544 p.value = 0
roasted
0.4
0.3
0.2
0.1
0.10
0.15
0.20
0.25
raw

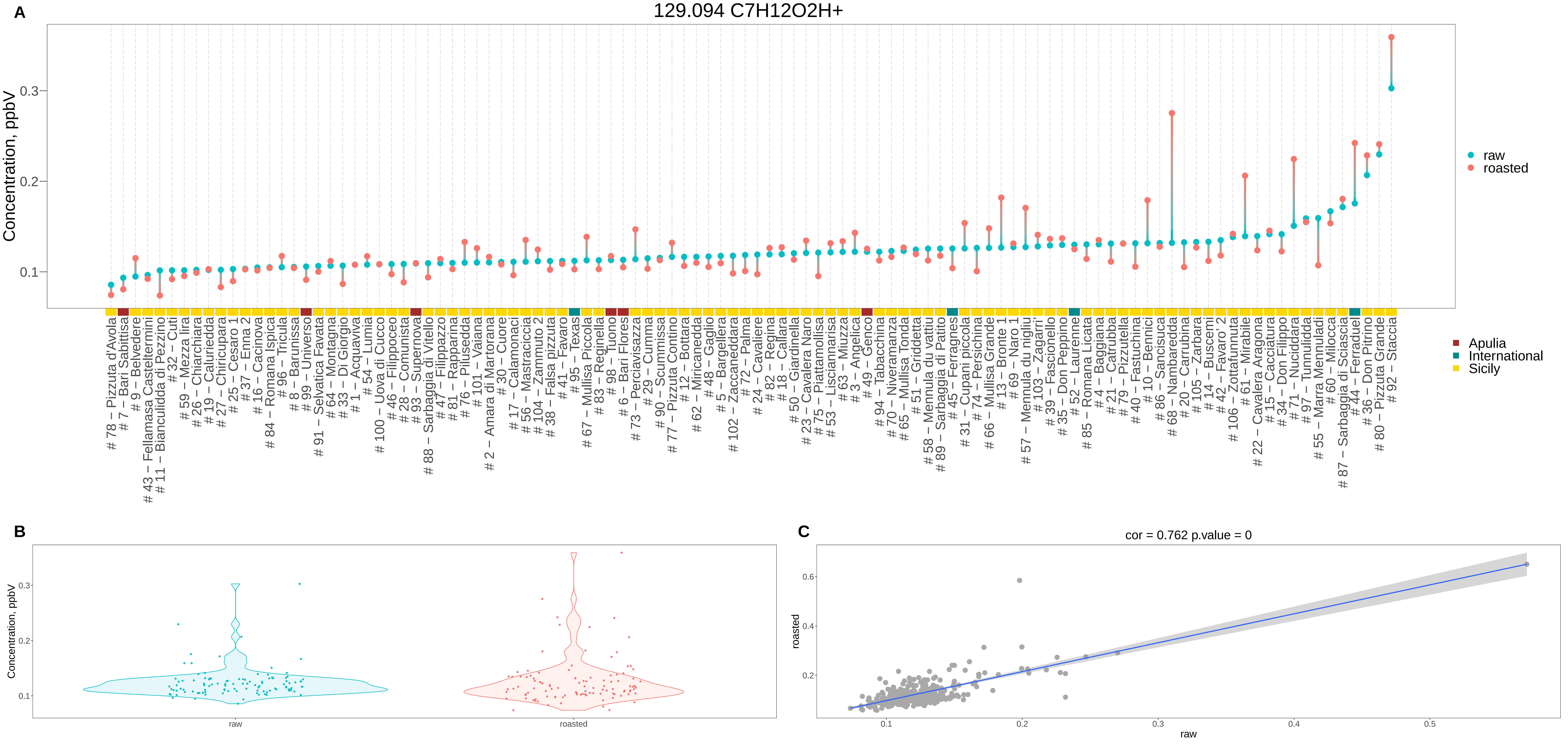
A
129.094 C7H12O2H+
Concentration, ppbV
0.1
0.2
0.3
raw
roasted
Apulia
International
Sicily
78 – Pizzuta d'Avola
7 – Bari Sabittisa
9 – Belvedere
43 – Fellamasa Casteltermini
11 – Bianculidda di Pezzino
32 – Cuti
59 – Mezza lira
26 – Chiarchiara
19 – Caluriedda
27 – Chiricupara
25 – Cesaro 1
37 – Enna 2
16 – Cacinova
84 – Romana Ispica
96 – Tricula
8 – Barunissa
99 – Universo
91 – Selvatica Favata
64 – Montagna
33 – Di Giorgio
1 – Acquaviva
54 – Lumia
100 – Uova di Cucco
46 – Filippoceo
28 – Comunista
93 – Supernova
88 – Sarbaggia di Vitello
47 – Filippazzo
81 – Rapparina
76 – Pilusedda
101 – Vaiana
2 – Amara di Martorana
30 – Cuore
17 – Calamonaci
56 – Mastracicca
104 – Zammuto 2
38 – Falsa pizzuta
41 – Favaro
95 – Texas
67 – Mullisa Piccola
83 – Reginella
98 – Tuono
6 – Bari Flores
73 – Perciavisazza
29 – Cummissa
90 – Scummissa
77 – Pizzuta Contino
12 – Bottara
62 – Miricanedda
48 – Gaglio
5 – Bargellera
102 – Zaccaneddara
72 – Palma
24 – Cavaliere
82 – Regina
18 – Callara
50 – Giardinella
23 – Cavalera Naro
75 – Piattamollisa
53 – Liscianarisa
63 – Miuzza
3 – Angelica
49 – Genco
94 – Tabacchina
70 – Niveramanza
65 – Mullisa Tonda
51 – Griddetta
58 – Mennula du vattiu
89 – Sarbaggia di Patito
45 – Ferragnes
31 – Cupani piccola
74 – Persichina
66 – Mullisa Grande
13 – Bronte 1
69 – Naro 1
57 – Mennula du nigliu
103 – Zagarri'
39 – Fascionello
35 – Don Peppino
52 – Laurenne
85 – Romana Licata
4 – Baggiana
21 – Catrubba
79 – Pizzutella
40 – Fastuchina
10 – Bennici
86 – Sancisuca
68 – Nambaredda
20 – Carrubina
105 – Zarbara
14 – Buscemi
42 – Favaro' 2
106 – Zottafunnuta
61 – Mirabile
22 – Cavalera Aragona
15 – Cacciatura
34 – Don Filippo
71 – Nuciddara
97 – Tunnulidda
55 – Marra Mennuladi
60 – Milocca
87 – Sarbaggia di Sciascia
44 – Ferraduel
36 – Don Pitrino
80 – Pizzuta Grande
92 – Staccia
B
Concentration, ppbV
0.1
0.2
0.3
raw
roasted
C
cor = 0.762 p.value = 0
roasted
0.2
0.4
0.6
raw
0.1
0.2
0.3
0.4
0.5

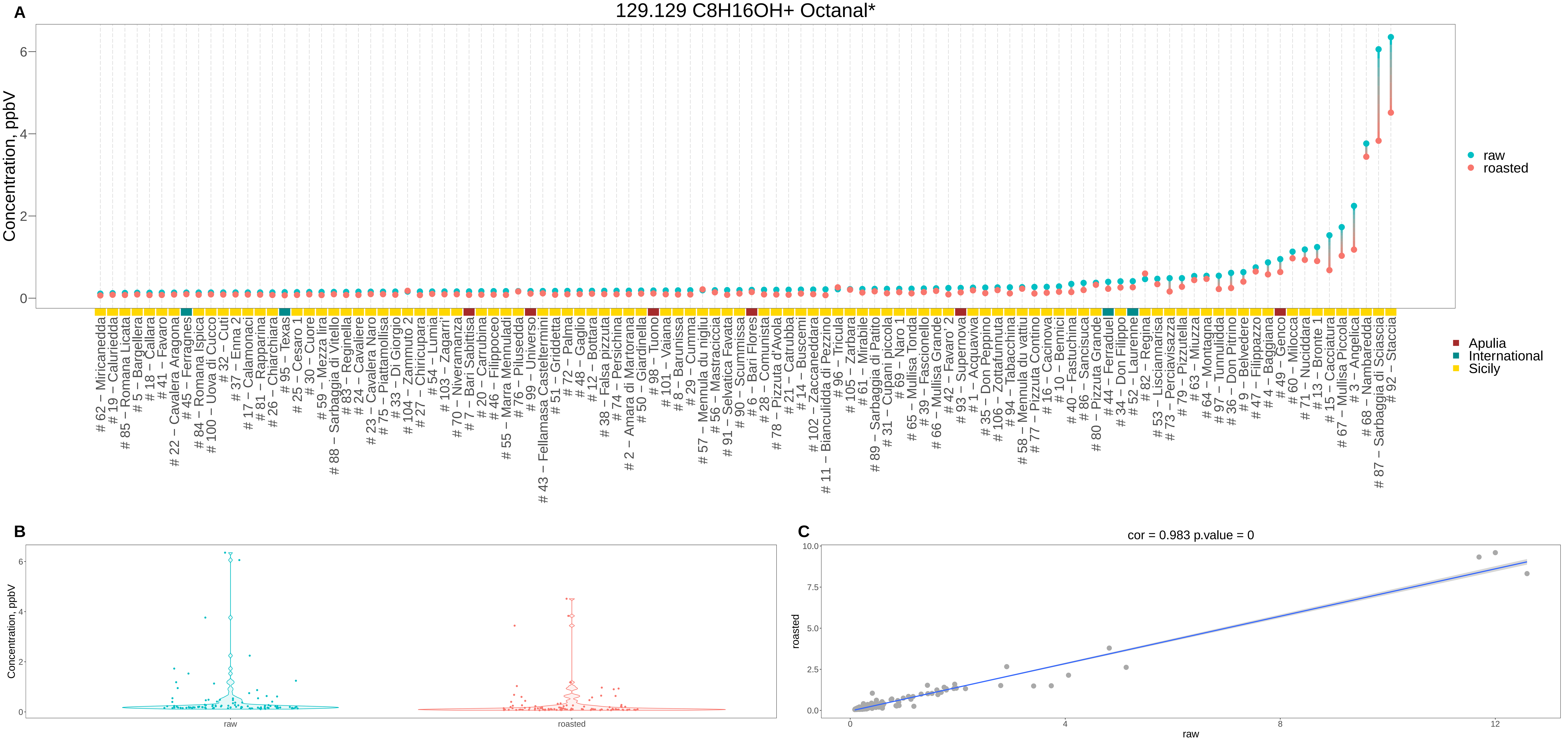

A
129.129 C8H16OH+ Octanal*
Concentration, ppbV
0
2
4
6
raw
roasted
Apulia
International
Sicily
62 – Miricanedda
19 – Caluriedda
85 – Romana Licata
5 – Bargellera
18 – Callara
41 – Favaro
22 – Cavalera Aragona
45 – Ferragnes
84 – Romana Ispica
100 – Uova di Cucco
32 – Cuti
37 – Enna 2
17 – Calamonaci
81 – Rapparina
26 – Chiarchiara
95 – Texas
25 – Cesaro 1
30 – Cuore
59 – Mezza lira
88 – Sarbaggia di Vitello
83 – Reginella
24 – Cavaliere
23 – Cavalera Naro
75 – Piattamollisa
33 – Di Giorgio
104 – Zammuto 2
27 – Chiricupara
54 – Lumia
103 – Zagarri'
70 – Niveramanza
7 – Bari Sabittisa
20 – Carrubina
46 – Filippoceo
55 – Marra Mennuladi
76 – Pilusedda
99 – Universo
43 – Fellamasa Casteltermini
51 – Griddetta
72 – Palma
48 – Gaglio
12 – Bottara
38 – Falsa pizzuta
74 – Persichina
2 – Amara di Martorana
50 – Giardinella
98 – Tuono
101 – Vaiana
8 – Barunissa
29 – Cumma
57 – Mennula du nigliu
56 – Mastracicia
91 – Selvatica Favata
90 – Scummissa
6 – Bari Flores
28 – Comunista
78 – Pizzuta d'Avola
21 – Catrubba
14 – Buscemi
102 – Zaccaneddara
11 – Bianculidda di Pezzino
96 – Tricula
105 – Zarbara
61 – Mirabile
89 – Sarbaggia di Patito
31 – Cupani piccola
69 – Naro 1
65 – Mullisa Tonda
39 – Fascionello
66 – Mullisa Grande
42 – Favaro' 2
93 – Supernova
1 – Acquaviva
35 – Don Peppino
106 – Zottafunnuta
94 – Tabacchina
58 – Mennula du vattiu
77 – Pizzuta Contino
16 – Cacinova
10 – Bennici
40 – Fastuchina
86 – Sancisuca
80 – Pizzuta Grande
44 – Ferraduel
34 – Don Filippo
52 – Laurenne
82 – Regina
53 – Lisciannarisa
73 – Perciavisazza
79 – Pizzutella
63 – Miuzza
64 – Montagna
97 – Tunnulidda
36 – Don Pitrino
9 – Belvedere
47 – Filippazzo
4 – Baggiana
49 – Genco
60 – Milocca
71 – Nuciddara
13 – Bronte 1
15 – Cacciatura
67 – Mullisa Piccola
3 – Angelica
68 – Nambaredda
87 – Sarbaggia di Sciascia
92 – Staccia
B
Concentration, ppbV
raw
roasted
C
cor = 0.983 p.value = 0
roasted
raw
0.0
2.5
5.0
7.5
10.0
0
4
8
12

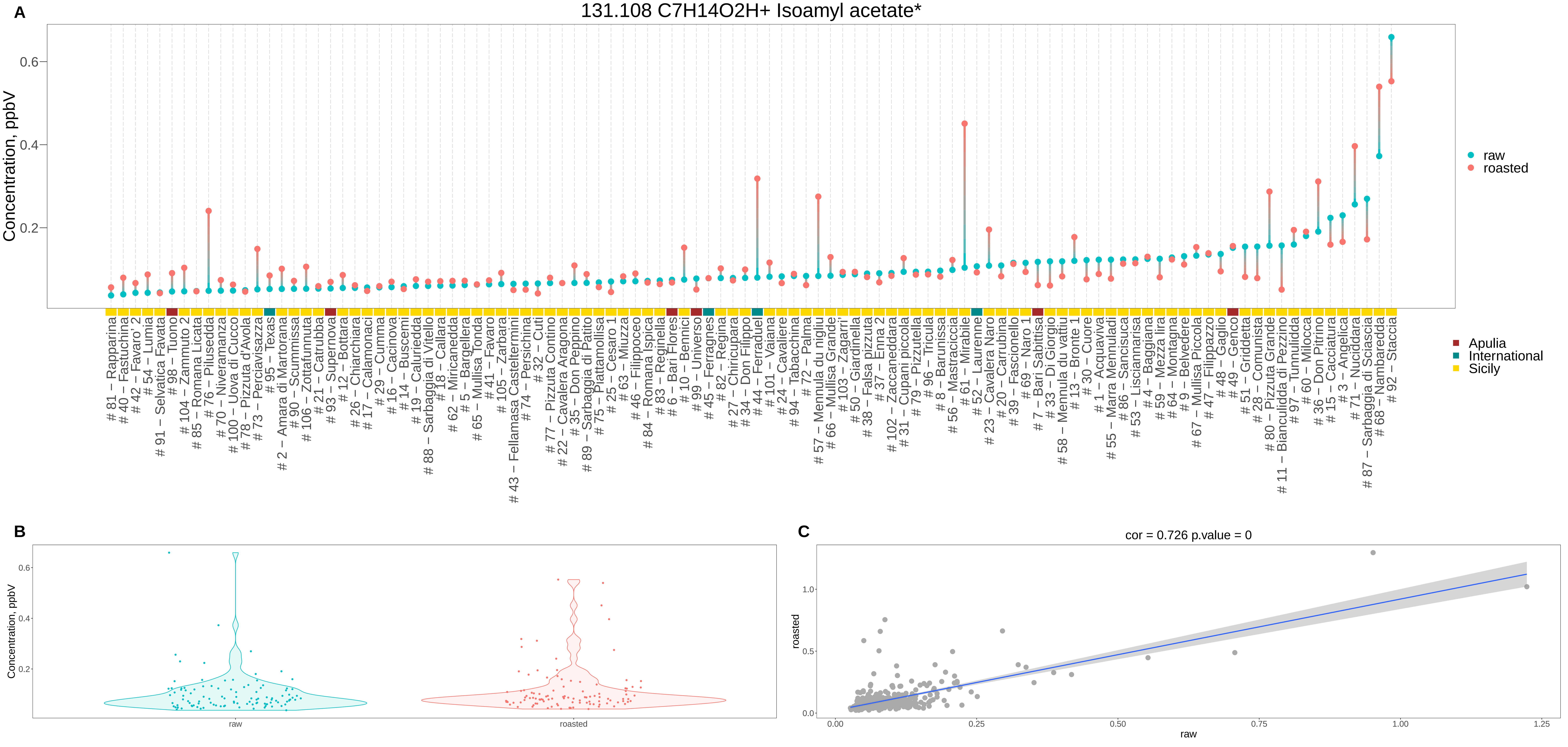
A
131.108 C7H14O2H+ Isoamyl acetate*
Concentration, ppbV
0.2
0.4
0.6
raw
roasted
Apulia
International
Sicily
81 – Rapparina
40 – Fastuchina
42 – Favaro' 2
54 – Lumia
91 – Selvatica Favata
98 – Tuono
104 – Zammuto 2
85 – Romana Licata
76 – Pilusedda
70 – Niveramanza
100 – Uova di Cucco
78 – Pizzuta d'Avola
73 – Perciavisazza
95 – Texas
2 – Amara di Martorana
90 – Scummissa
106 – Zottafunnuta
21 – Catrubba
93 – Supernova
12 – Bottara
26 – Chiarchiara
17 – Calamonaci
29 – Cumma
16 – Cacinova
14 – Buscemi
19 – Caluriedda
88 – Sarbaggia di Vitello
18 – Callara
62 – Miricanedda
5 – Bargellera
65 – Mullisa Tonda
41 – Favaro
105 – Zarbara
43 – Fellamasa Casteltermini
74 – Persichina
32 – Cuti
77 – Pizzuta Contino
22 – Cavalera Aragona
35 – Don Peppino
89 – Sarbaggia di Patito
75 – Piattamollisa
25 – Cesaro 1
63 – Miuzza
46 – Filippoceo
84 – Romana Ispica
83 – Reginella
6 – Bari Flores
10 – Bennici
99 – Universo
45 – Ferragnes
82 – Regina
27 – Chiricupara
34 – Don Filippo
44 – Ferraduel
101 – Vaiana
24 – Cavaliere
94 – Tabacchina
72 – Palma
57 – Mennula du nigliu
66 – Mullisa Grande
103 – Zagarri'
50 – Giardinella
38 – Falsa pizzuta
37 – Enna 2
102 – Zaccaneddara
31 – Cupani piccola
79 – Pizzutella
96 – Tricula
8 – Barunissa
56 – Mastracicca
61 – Mirabile
52 – Laurenne
23 – Cavalera Naro
20 – Carrubina
39 – Fascionello
69 – Naro 1
7 – Bari Sabtitsa
33 – Di Giorgio
58 – Mennula du vattiu
13 – Bronte 1
30 – Cuore
1 – Acquaviva
55 – Marra Mennuladi
86 – Sancisuca
53 – Lisciannarisa
4 – Baggiana
59 – Mezza lira
64 – Montagna
9 – Belvedere
67 – Mullisa Piccola
47 – Filippazzo
48 – Gaglio
49 – Genco
51 – Griddetta
28 – Comunista
80 – Pizzuta Grande
11 – Biancuidda di Pezzino
97 – Tunnulidda
60 – Milocca
36 – Don Pitrino
15 – Cacciatura
3 – Angelica
71 – Nuciddara
87 – Sarbaggia di Sciascia
68 – Nambaredda
92 – Staccia
B
Concentration, ppbV
0.2
0.4
0.6
raw
roasted
C
cor = 0.726 p.value = 0
roasted
0.0
0.5
1.0
0.00
0.25
0.50
0.75
1.00
1.25
raw

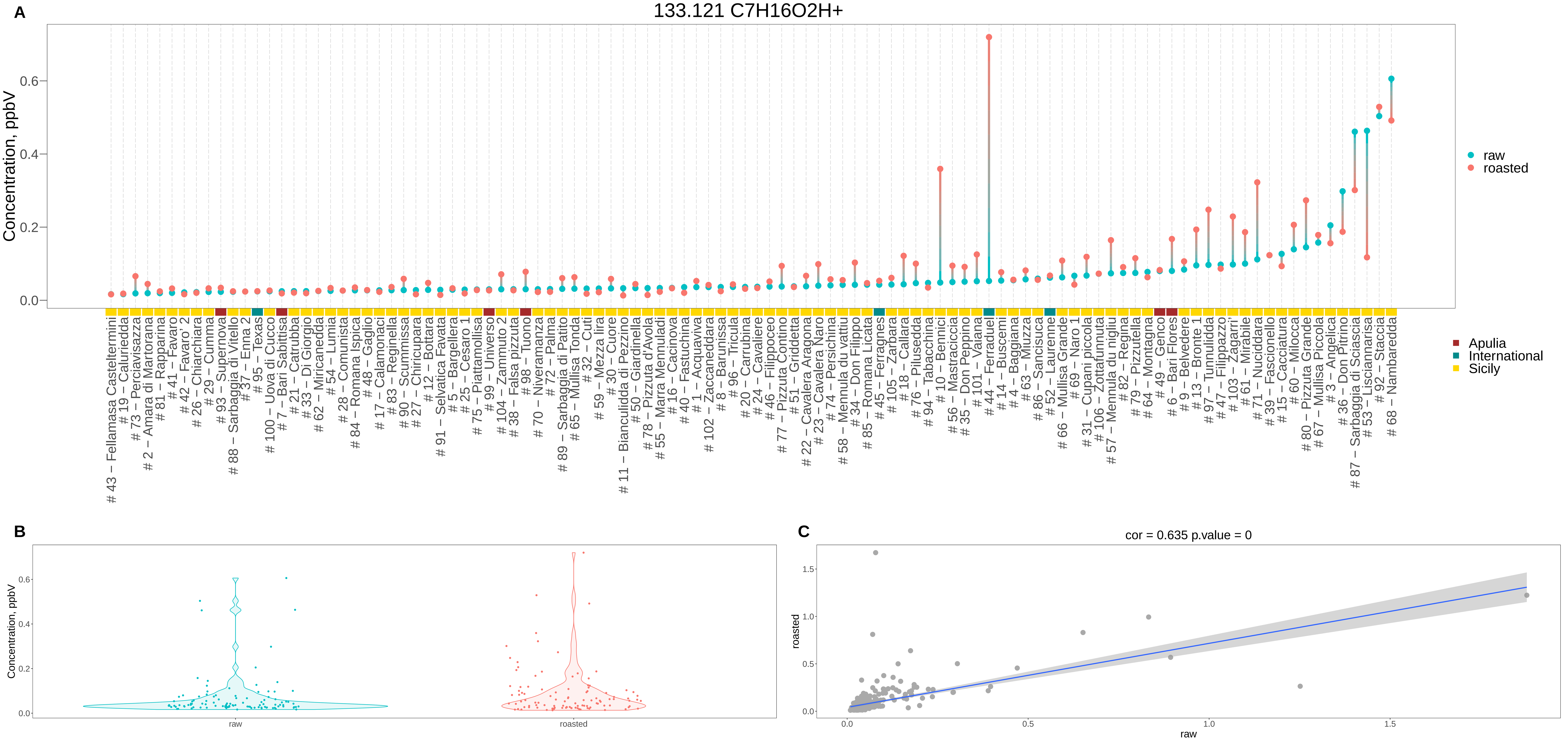
A
133.121 C7H16O2H+
Concentration, ppbV
0.0
0.2
0.4
0.6
raw
roasted
Apulia
International
Sicily
43 – Fellamasa Casteltermini
19 – Caluriedda
73 – Perciavisazza
2 – Amara di Martorana
81 – Rapparina
41 – Favaro
42 – Favaro' 2
26 – Chiarchiara
29 – Cumma
93 – Supernova
88 – Sarbaggia di Vitello
37 – Enna 2
95 – Texas
100 – Uova di Cucco
7 – Bari Sabittisa
21 – Catrubba
33 – Di Giorgio
62 – Miricanedda
54 – Lumia
28 – Comunista
84 – Romana Ispica
48 – Gaglio
17 – Calamonaci
83 – Reginella
90 – Scummissa
27 – Chiricupara
12 – Bottara
91 – Selvatica Favata
5 – Bargellera
25 – Cesaro 1
75 – Piattamollisa
99 – Universo
104 – Zammuto 2
38 – Falsa pizzuta
98 – Tuono
70 – Niveramanza
72 – Palma
89 – Sarbaggia di Patito
65 – Mullisa Tonda
32 – Cuti
59 – Mezza lira
30 – Cuore
11 – Bianculidda di Pezzino
50 – Giardinella
78 – Pizzuta d'Avola
55 – Marra Mennuladi
16 – Cacinova
40 – Fastuchina
1 – Acquaviva
102 – Zaccaneddara
8 – Barunissa
96 – Tricula
20 – Carrubina
24 – Cavaliere
46 – Filippoceo
77 – Pizzuta Contino
51 – Griddetta
22 – Cavalera Aragona
23 – Cavalera Naro
74 – Persichina
58 – Mennula du vattiu
34 – Don Filippo
85 – Romana Licata
45 – Ferragnes
105 – Zarbara
18 – Callara
76 – Pilusedda
94 – Tabacchina
10 – Bennici
56 – Mastraciccia
35 – Don Peppino
101 – Vaiana
44 – Ferraduel
14 – Buscemi
4 – Baggiana
63 – Miuzza
86 – Sancisuca
52 – Laurenne
66 – Mullisa Grande
69 – Naro 1
31 – Cupani piccola
106 – Zottafunnuta
57 – Mennula du nigliu
82 – Regina
79 – Pizzutella
64 – Montagna
49 – Genco
6 – Bari Flores
9 – Belvedere
13 – Bronte 1
97 – Tunnulidda
47 – Filippazzo
103 – Zagarri'
61 – Mirabile
71 – Nuciddara
39 – Fascionello
15 – Cacciatura
60 – Milocca
80 – Pizzuta Grande
67 – Mullisa Piccola
3 – Angelica
36 – Don Pitrino
87 – Sarbaggia di Sciascia
53 – Liscianarisa
92 – Staccia
68 – Nambaredda
B
Concentration, ppbV
0.0
0.2
0.4
0.6
raw
roasted
C
cor = 0.635 p.value = 0
roasted
0.0
0.5
1.0
1.5
raw
0.0
0.5
1.0
1.5

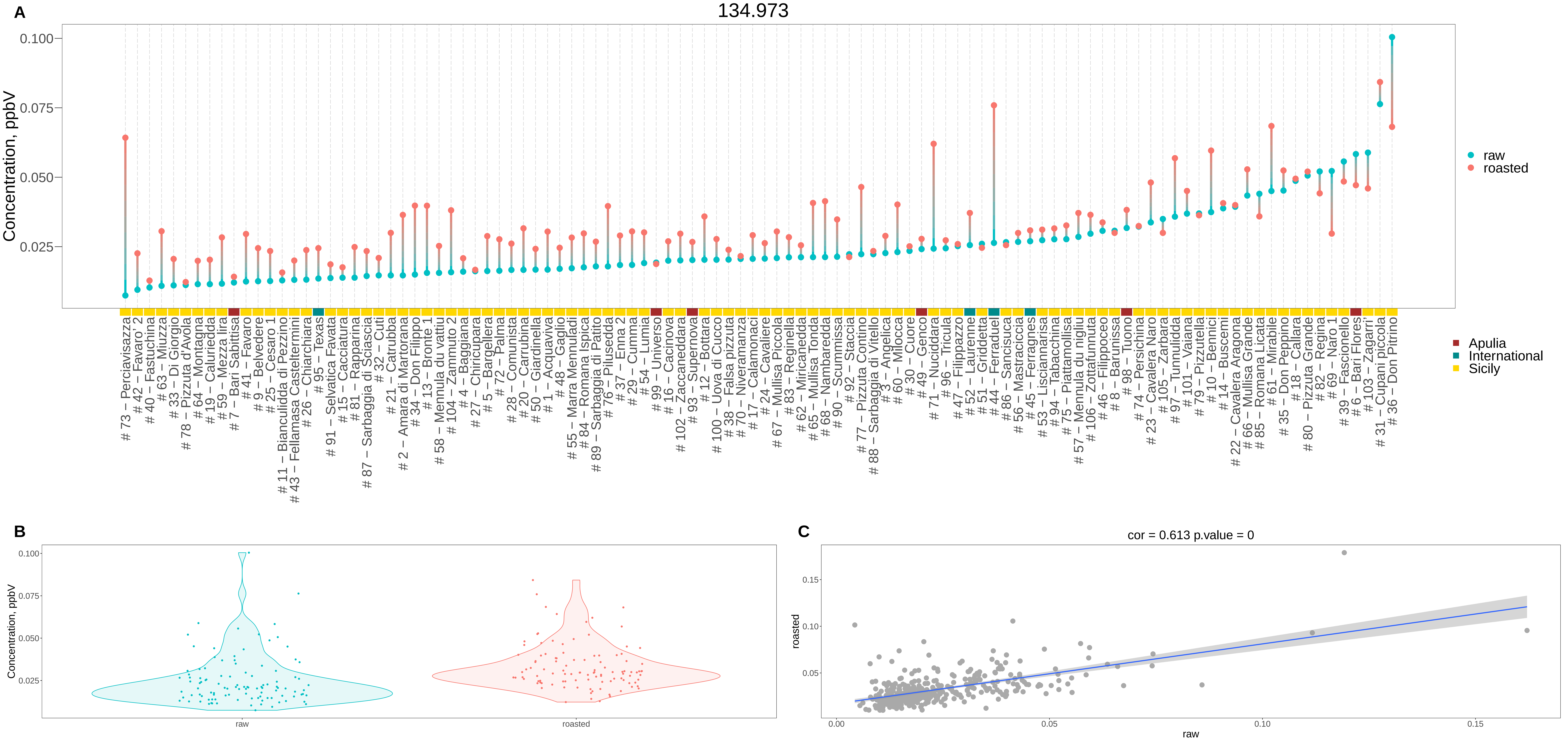

A
134.973
Concentration, ppbV
0.100
0.075
0.050
0.025
raw
roasted
Apulia
International
Sicily
73 – Perciavisazza
42 – Favaro 2
40 – Fastuchina
63 – Miuzza
33 – Di Giorgio
78 – Pizzuta d'Avola
64 – Montagna
19 – Caluriedda
59 – Mezza lira
7 – Bari Sabittisa
41 – Favaro
9 – Belvedere
25 – Cesaro 1
11 – Bianculidda di Pezzino
43 – Fellamasa Casteltermini
26 – Chiarchiari
95 – Texas
91 – Selvatica Favata
15 – Cacciatura
81 – Rapparina
87 – Sarbaggia di Sciascia
32 – Cuti
21 – Catrubba
2 – Amara di Martorana
34 – Don Filippo
13 – Bronte 1
58 – Mennula du vattiu
104 – Zammuto 2
4 – Baggiana
27 – Chiricupara
5 – Bargellera
72 – Palma
28 – Comunista
20 – Carrubina
50 – Giardinella
1 – Acquaviva
48 – Gaglio
55 – Marra Mennuladi
84 – Romana Ispica
89 – Sarbaggia di Patito
76 – Pilusedda
37 – Enna 2
29 – Cumma
54 – Lumia
99 – Universo
16 – Cacinova
102 – Zaccaneddara
93 – Supernova
12 – Bottara
100 – Uova di Cucco
38 – Falsa pizzuta
70 – Niveramanza
17 – Calamonaci
24 – Cavaliere
67 – Mullisa Piccola
83 – Reginella
62 – Miricanedda
65 – Mullisa Tonda
68 – Nambaredda
90 – Scummissa
92 – Staccia
77 – Pizzuta Contino
88 – Sarbaggia di Vitello
3 – Angelica
60 – Milocca
30 – Cuore
49 – Genco
71 – Nuciddara
96 – Tricula
47 – Filippazzo
52 – Laurenne
51 – Griddetta
44 – Ferraduel
86 – Sancisuca
56 – Mastracicia
45 – Ferragnes
53 – Liscianarisa
94 – Tabacchina
75 – Piattamollisa
57 – Mennula du nigliu
106 – Zottafunnuta
46 – Filippoceo
8 – Barunissa
98 – Tuono
74 – Persichina
23 – Cavalera Naro
105 – Zarbara
97 – Tunnulidda
101 – Vaiana
79 – Pizzutella
10 – Bennici
14 – Buscemi
22 – Cavalera Aragona
66 – Mullisa Grande
85 – Romana Licata
61 – Mirabile
35 – Don Peppino
18 – Callara
80 – Pizzuta Grande
82 – Regina
69 – Naro 1
39 – Fascionello
6 – Bari Flores
103 – Zagarri
31 – Cupani piccola
36 – Don Pitrino
B
Concentration, ppbV
0.100
0.075
0.050
0.025
raw
roasted
C
cor = 0.613 p.value = 0
roasted
0.15
0.10
0.05
0.00
0.05
0.10
0.15
raw

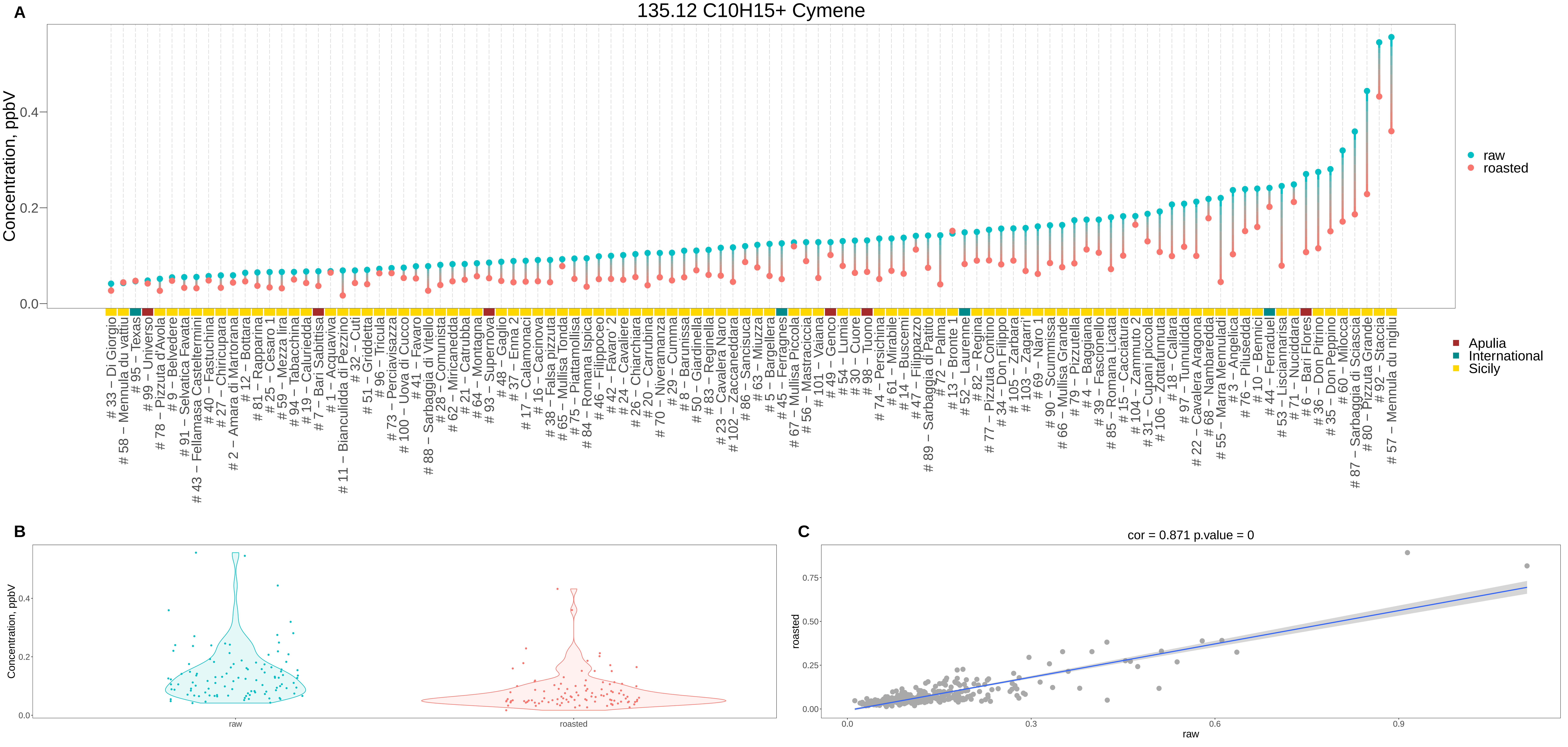
A
135.12 C10H15+ Cymene
Concentration, ppbV
0.0
0.2
0.4
raw
roasted
Apulia
International
Sicily
33 – Di Giorgio
58 – Mennula du vattiu
95 – Texas
99 – Universo
78 – Pizzuta d'Avola
9 – Belvedere
91 – Selvatica Favata
43 – Fellamasa Casteltermini
40 – Fastuchina
27 – Chiricupara
2 – Amara di Martorana
12 – Bottara
81 – Rapparina
25 – Cesaro 1
59 – Mezza lira
94 – Tabacchina
19 – Caluriedda
7 – Bari Sabitissa
1 – Acquaviva
11 – Bianculidda di Pezzino
32 – Cuti
51 – Griddetta
96 – Tricula
73 – Perciavisazza
100 – Uova di Cucco
41 – Favaro
88 – Sarbaggia di Vitello
28 – Comunista
62 – Miricanedda
21 – Catrubba
64 – Montagna
93 – Supernova
48 – Gaglio
37 – Enna 2
17 – Calamonaci
16 – Cacinova
38 – Falsa pizzuta
65 – Mullisa Tonda
75 – Piattamollisa
84 – Romana Ispica
46 – Filippoceo
42 – Favaro' 2
24 – Cavaliere
26 – Chiarchiara
20 – Carrubina
70 – Niveramanza
29 – Cumma
8 – Barunissa
50 – Giardinella
83 – Reginella
23 – Cavalera Naro
102 – Zaccaneddara
86 – Sancisuca
63 – Miuzza
5 – Bargellera
45 – Ferragnes
67 – Mullisa Piccola
56 – Mastraciccia
101 – Vaiana
49 – Genco
54 – Lumia
30 – Cuore
98 – Tuono
74 – Persichina
61 – Mirabile
14 – Buscemi
47 – Filippazzo
89 – Sarbaggia di Patito
72 – Palma
13 – Bronte 1
52 – Laurenne
82 – Regina
77 – Pizzuta Contino
34 – Don Filippo
105 – Zarbaria
103 – Zagarri'
69 – Naro 1
90 – Scummissa
66 – Mullisa Grande
79 – Pizzutella
4 – Baggiana
39 – Fascionello
85 – Romana Licata
15 – Cacciatura
104 – Zammuto 2
31 – Cupani piccola
106 – Zottafunnuta
18 – Callara
97 – Tunnulidda
22 – Cavalera Aragona
68 – Nambaredda
55 – Marra Mennuladi
3 – Angelica
76 – Pilusedda
10 – Bennici
44 – Ferraduel
53 – Lisciannarisa
71 – Nuciddara
6 – Bari Flores
36 – Don Pitrino
35 – Don Peppino
60 – Milocca
87 – Sarbaggia di Sciascia
80 – Pizzuta Grande
92 – Staccia
57 – Mennula du nigliu
B
Concentration, ppbV
0.0
0.2
0.4
raw
roasted
C
cor = 0.871 p.value = 0
roasted
0.00
0.25
0.50
0.75
raw
0.0
0.3
0.6
0.9

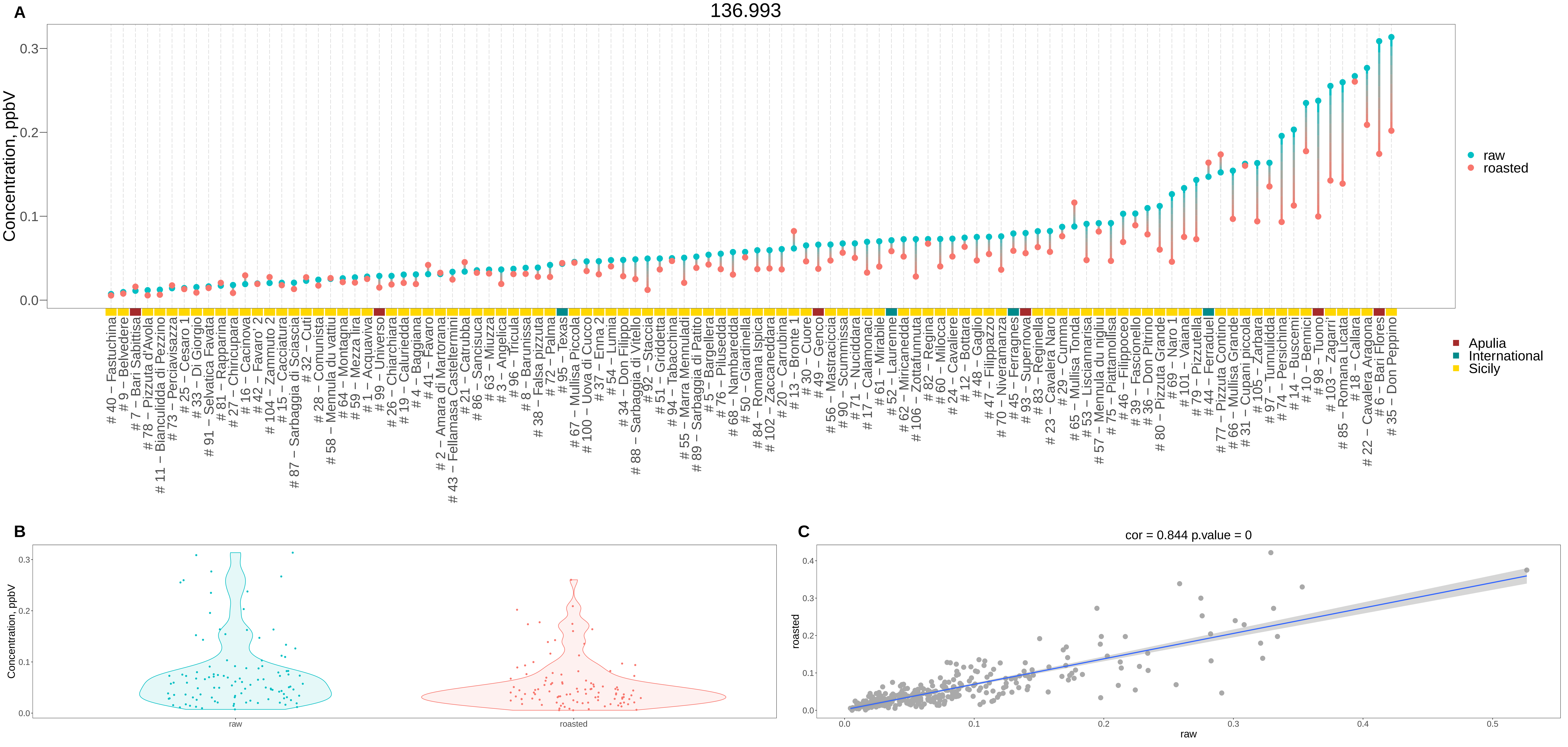
A
136.993
Concentration, ppbV
0.0
0.1
0.2
0.3
raw
roasted
Apulia
International
Sicily
40 – Fastuchina
9 – Belvedere
7 – Bari Sabittisa
78 – Pizzuta d'Avola
11 – Bianculidda di Pezzino
73 – Perciavisazza
25 – Cesaro 1
33 – Di Giorgio
91 – Selvatica Favata
81 – Rapparina
27 – Chiricupara
16 – Cacinova
42 – Favaro' 2
104 – Zammuto 2
15 – Cacciatura
87 – Sarbaggia di Sciascia
32 – Cuti
28 – Comunista
58 – Mennula du vattiu
64 – Montagna
59 – Mezza lira
1 – Acquaviva
99 – Universo
26 – Chiarchiara
19 – Caluriedda
4 – Baggiana
41 – Favaro
2 – Amara di Martorana
43 – Fellamasa Casteltermini
21 – Catrubba
86 – Sancisuca
63 – Miuzza
3 – Angelica
96 – Tricula
8 – Barunissa
38 – Falsa pizzuta
72 – Palma
95 – Texas
67 – Mullisa Piccola
100 – Uova di Cucco
37 – Enna 2
54 – Lumia
34 – Don Filippo
88 – Sarbaggia di Vitello
92 – Staccia
51 – Griddetta
94 – Tabacchina
55 – Marra Mennuladi
89 – Sarbaggia di Patito
5 – Bargellera
76 – Pilusedda
68 – Nambaredda
50 – Giardinella
84 – Romana Ispica
102 – Zaccaneddara
20 – Carrubina
13 – Bronte 1
30 – Cuore
49 – Genco
56 – Mastracicia
90 – Scummissa
71 – Nuciddara
17 – Calamonaci
61 – Mirabile
52 – Laurenne
62 – Miricanedda
106 – Zottafunnuta
82 – Regina
60 – Milocca
24 – Cavaliere
12 – Bottara
48 – Gaglio
47 – Filippazzo
70 – Niveramanza
45 – Ferragnes
93 – Supernova
83 – Reginella
23 – Cavalera Naro
29 – Cumma
65 – Mullisa Tonda
53 – Liscianarisa
57 – Mennula du nigliu
75 – Piattamollisa
46 – Filippoceo
39 – Fascionello
36 – Don Pitrino
80 – Pizzuta Grande
69 – Naro 1
101 – Vaiana
79 – Pizzutella
44 – Ferraduel
77 – Pizzuta Contino
66 – Mullisa Grande
31 – Cupani piccola
105 – Zarbara
97 – Tunnulidda
74 – Persichina
14 – Buscemi
10 – Bennici
98 – Tuono
103 – Zagarri'
85 – Romana Licata
18 – Callara
22 – Cavalera Aragona
6 – Bari Flores
35 – Don Peppino
B
Concentration, ppbV
0.0
0.1
0.2
0.3
raw
roasted
C
cor = 0.844 p.value = 0
roasted
raw
0.0
0.1
0.2
0.3
0.4
0.5

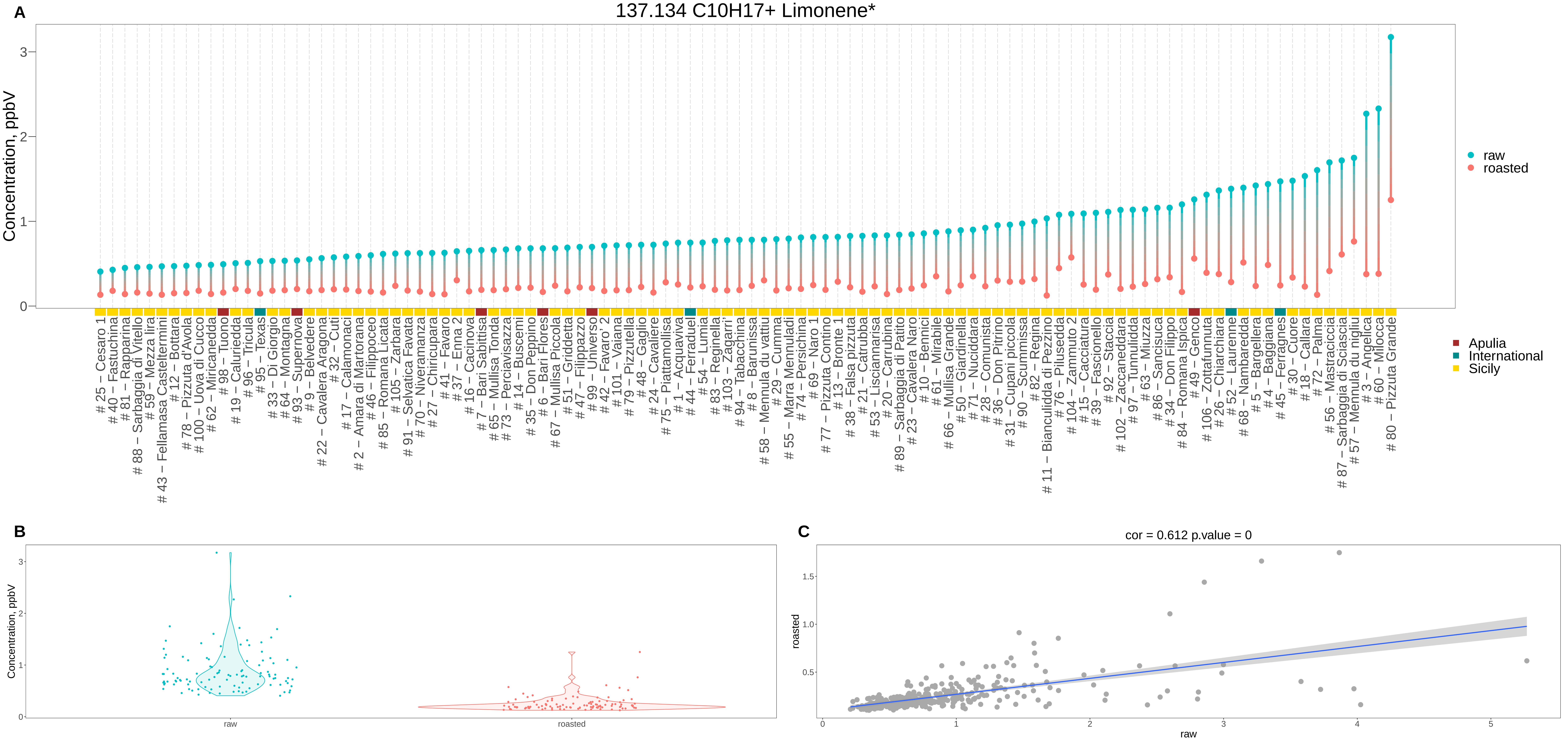
A
137.134 C10H17+ Limonene*
Concentration, ppbV
0
1
2
3
raw
roasted
Apulia
International
Sicily
25 – Cesaro 1
40 – Fastuchina
81 – Rapparina
88 – Sarbaggia di Vitello
59 – Mezza lira
43 – Fellamasa Casteltermini
12 – Bottara
78 – Pizzuta d'Avola
100 – Uova di Cucco
62 – Miricanedda
98 – Tuono
19 – Caluriedda
96 – Tricula
95 – Texas
33 – Di Giorgio
64 – Montagna
93 – Supernova
9 – Belvedere
22 – Cavalera Aragona
32 – Cuti
17 – Calamonaci
2 – Amara di Martorana
46 – Filippoceo
85 – Romana Licata
105 – Zarbara
91 – Selvatica Favata
70 – Niveramanza
27 – Chiricuparo
41 – Favaro
37 – Enna 2
16 – Cacinova
7 – Bari Sabittisa
65 – Mullisa Tonda
73 – Perciavisazza
14 – Buscemi
35 – Don Peppino
6 – Bari Flores
67 – Mullisa Piccola
51 – Griddetta
47 – Filippazzo
99 – Universo
42 – Favaro 2
101 – Vaiana
79 – Pizzutella
48 – Gaglio
24 – Cavaliere
75 – Piattamollisa
1 – Acquaviva
44 – Ferraduel
54 – Lumia
83 – Reginella
103 – Zagarri'
94 – Tabacchina
8 – Barunissa
58 – Mennula du vattiu
29 – Cumma
55 – Marra Mennuladi
74 – Persichina
69 – Naro 1
77 – Pizzuta Contino
13 – Bronte 1
38 – Falsa pizzuta
21 – Catrubba
53 – Lisciannarisa
20 – Carrubina
89 – Sarbaggia di Patito
23 – Cavalera Naro
10 – Bennici
61 – Mirabile
66 – Mullisa Grande
50 – Giardinella
71 – Nuciddara
28 – Comunista
36 – Don Pitrino
31 – Cupani piccola
90 – Scummissa
82 – Regina
11 – Bianculidda di Pezzino
76 – Pilusedda
104 – Zammuto 2
15 – Cacciatura
39 – Fascionello
92 – Staccia
102 – Zaccaneddara
97 – Tunnulidda
63 – Miuzza
86 – Sancisuca
34 – Don Filippo
84 – Romana Ispica
49 – Genco
106 – Zottafunnuta
26 – Chiarchiara
52 – Laurenne
68 – Nambaredda
5 – Bargellera
4 – Baggiana
45 – Ferragnes
30 – Cuore
18 – Callara
72 – Palma
56 – Mastraciccia
87 – Sarbaggia di Sciascia
57 – Mennula du nigliu
3 – Angelica
60 – Milocca
80 – Pizzuta Grande
B
Concentration, ppbV
0
1
2
3
raw
roasted
C
cor = 0.612 p.value = 0
roasted
0.5
1.0
1.5
raw
0
1
2
3
4
5

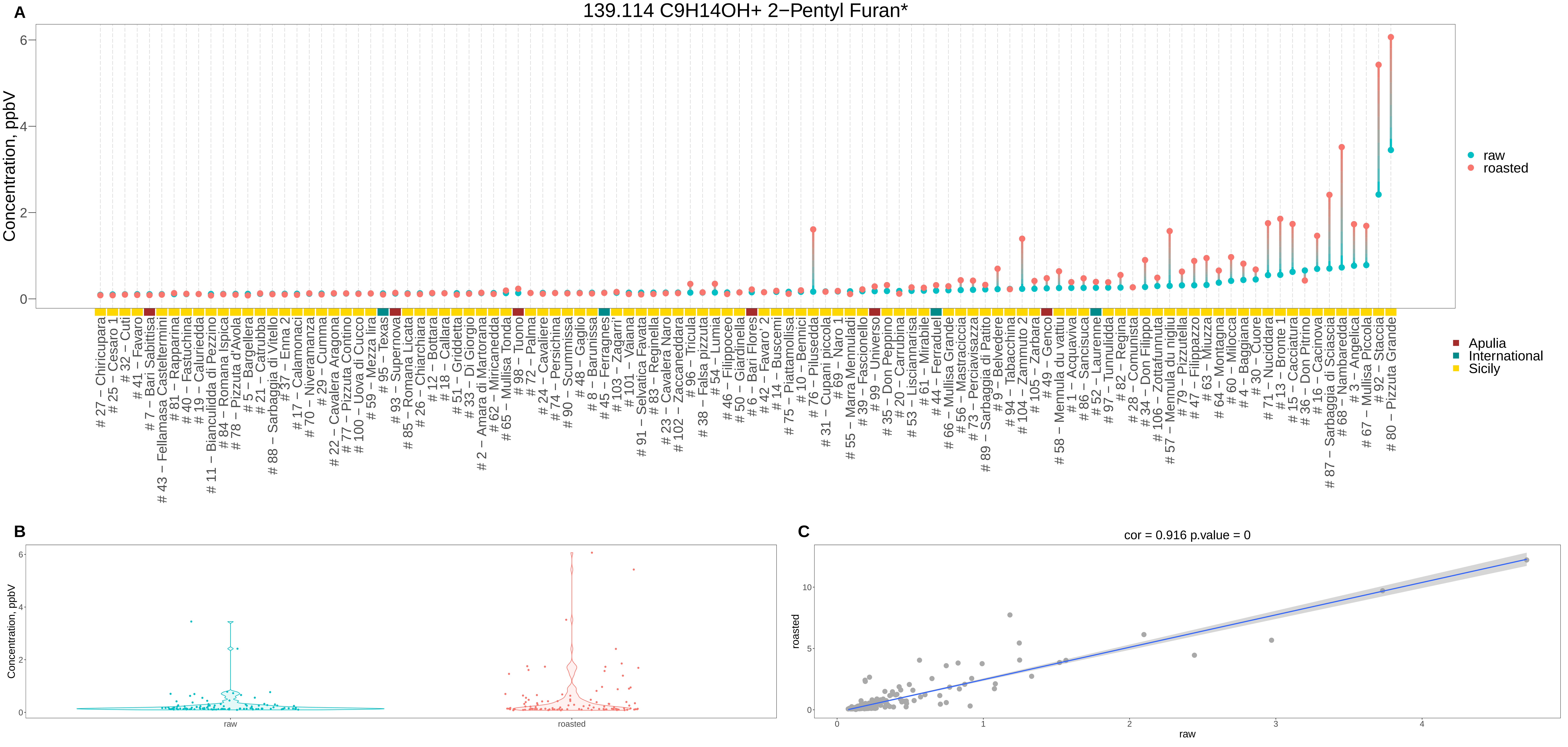
A
139.114 C9H14OH+ 2−Pentyl Furan*
Concentration, ppbV
0
2
4
6
raw
roasted
Apulia
International
Sicily
27 − Chiricupara
25 − Cesaro 1
32 − Cuti
41 − Favaro
7 − Bari Sabitissa
43 − Fellamasa Casteltermini
81 − Rapparina
40 − Fastuchina
19 − Caluriedda
11 − Bianculidda di Pezzino
84 − Romana Ispica
78 − Pizzuta d'Avola
5 − Bargellera
21 − Catrubba
88 − Sarbaggia di Vitello
37 − Enna 2
17 − Calamonaci
70 − Niveramanza
29 − Cumma
22 − Cavalera Aragona
77 − Pizzuta Contino
100 − Uova di Cucco
59 − Mezza lira
95 − Texas
93 − Supernova
85 − Romana Licata
26 − Chiarchiara
12 − Bottara
18 − Callara
51 − Griddetta
33 − Di Giorgio
2 − Amara di Martorana
62 − Miricanedda
65 − Mullisa Tonda
98 − Tuono
72 − Palma
24 − Cavaliere
74 − Persichina
90 − Scummissa
48 − Gaglio
8 − Barunissa
45 − Ferragnes
103 − Zagarri'
101 − Vaiana
91 − Selvatica Favata
83 − Reginella
23 − Cavalera Naro
102 − Zaccaneddara
96 − Tricula
38 − Falsa pizzuta
54 − Lumia
46 − Filippoceo
50 − Giardinella
6 − Bari Flores
42 − Favaro' 2
14 − Buscemi
75 − Piattamollisa
10 − Bennici
76 − Pilusedda
31 − Cupani piccola
69 − Naro 1
55 − Marra Mennuladi
39 − Fascionello
99 − Universo
35 − Don Peppino
20 − Carrubina
53 − Liscianarisa
61 − Mirabile
44 − Ferraduel
66 − Mullisa Grande
56 − Mastraciccia
73 − Perciavisazza
89 − Sarbaggia di Patito
9 − Belvedere
94 − Tabacchina
104 − Zammuto 2
105 − Zarbara
49 − Genco
58 − Mennula du vattiu
1 − Acquaviva
86 − Sancisuca
52 − Laurenne
97 − Tunnulidda
82 − Regina
28 − Comunista
34 − Don Filippo
106 − Zottafunnuta
57 − Mennula du nigliu
79 − Pizzutella
47 − Filippazzo
63 − Miuzza
64 − Montagna
60 − Milocca
4 − Baggiana
30 − Cuore
71 − Nuciddara
13 − Bronte 1
15 − Cacciatura
36 − Don Pitrino
16 − Cacinova
87 − Sarbaggia di Sciascia
68 − Nambaredda
3 − Angelica
67 − Mullisa Piccola
92 − Staccia
80 − Pizzuta Grande
B
Concentration, ppbV
raw
roasted
C
cor = 0.916 p.value = 0
roasted
raw

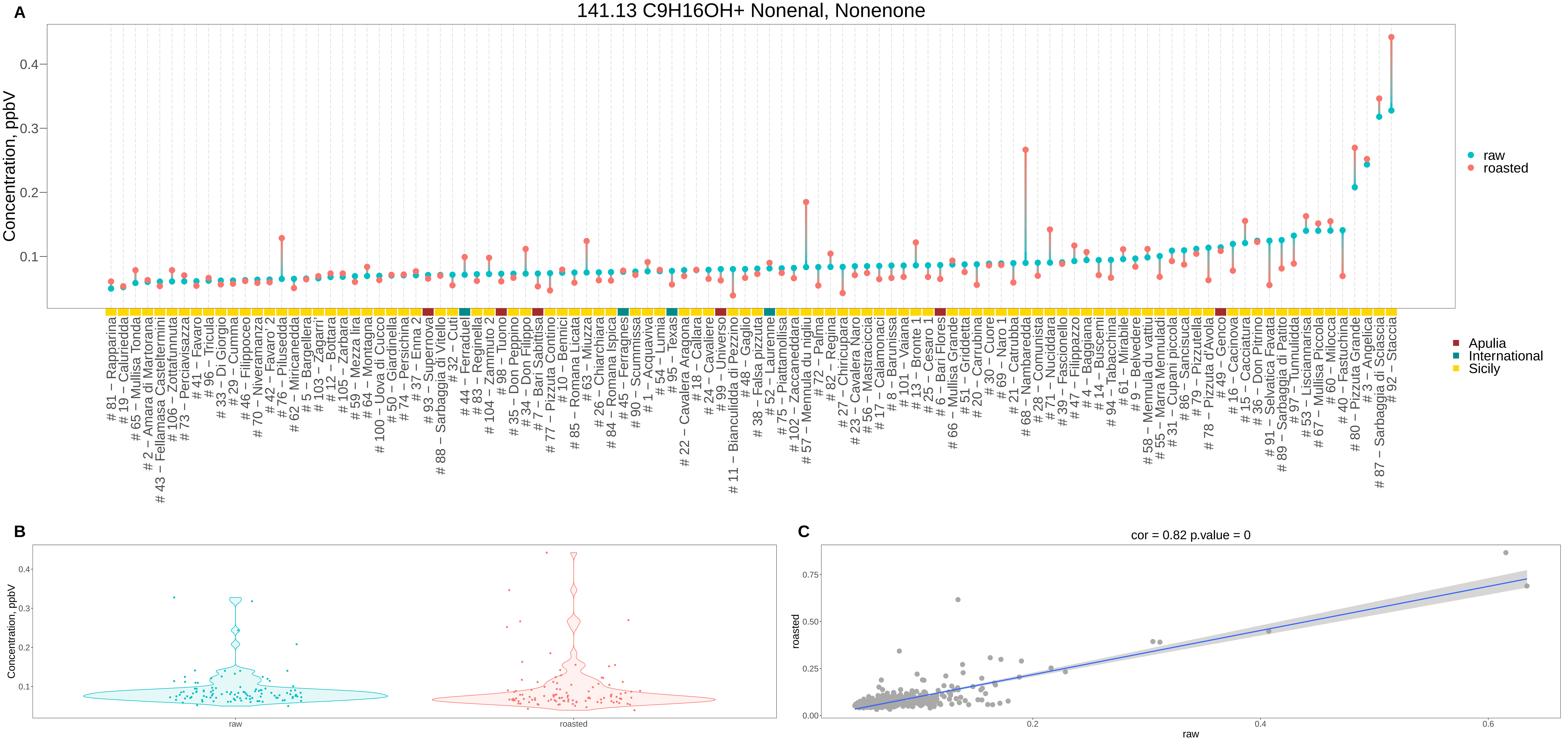

A
141.13 C9H16OH+ Nonenal, Nonenone
Concentration, ppbV
0.1
0.2
0.3
0.4
raw
roasted
Apulia
International
Sicily
81 – Rapparina
19 – Caluriedda
65 – Mullisa Tonda
2 – Amara di Martorana
43 – Fellamasa Casteltermini
106 – Zottafunnuta
73 – Perciavisazza
41 – Favaro
96 – Tricula
33 – Di Giorgio
29 – Cumma
46 – Filippoceo
70 – Niveramanza
42 – Favaro' 2
76 – Pilusedda
62 – Miricanedda
5 – Bargellera
103 – Zagarri'
12 – Bottara
105 – Zarbara
59 – Mezza lira
64 – Montagna
100 – Uova di Cucco
50 – Giardinella
74 – Persichina
37 – Enna 2
93 – Supernova
88 – Sarbaggia di Vitello
32 – Cuti
44 – Ferraduel
83 – Reginella
104 – Zammuto 2
98 – Tuono
35 – Don Peppino
34 – Don Filippo
7 – Bari Sabittisa
77 – Pizzuta Contino
10 – Bennici
85 – Romana Licata
63 – Miuzza
26 – Chiarchiara
84 – Romana Ispica
45 – Ferragnes
90 – Scummissa
1 – Acquaviva
54 – Lumia
95 – Texas
22 – Cavalera Aragona
18 – Callara
24 – Cavaliere
99 – Universo
11 – Bianculidda di Pezzino
48 – Gaglio
38 – Falsa pizzuta
52 – Laurenne
75 – Piattamollisa
102 – Zaccaneddara
57 – Mennula du nigliu
72 – Palma
82 – Regina
27 – Chiricupara
23 – Cavalera Naro
56 – Mastracicia
17 – Calamonaci
8 – Barunissa
101 – Vaiana
13 – Bronte 1
25 – Cesaro 1
6 – Bari Flores
66 – Mullisa Grande
51 – Griddetta
20 – Carrubina
30 – Cuore
69 – Naro 1
21 – Catrubba
68 – Nambaredda
28 – Comunista
71 – Nuciddara
39 – Fascionello
47 – Filippazzo
4 – Baggiana
14 – Buscemi
94 – Tabacchina
61 – Mirabile
9 – Belvedere
58 – Mennula du vattiu
55 – Marra Mennuladi
31 – Cupani piccola
86 – Sancisuca
79 – Pizzutella
78 – Pizzuta d'Avola
49 – Genco
16 – Cacinova
15 – Cacciatura
36 – Don Pitrino
91 – Selvatica Favata
89 – Sarbaggia di Patito
97 – Tunnulidda
53 – Lisciannarisa
67 – Mullisa Piccola
60 – Milocca
40 – Fastuchina
80 – Pizzuta Grande
3 – Angelica
87 – Sarbaggia di Sciascia
92 – Staccia
B
Concentration, ppbV
0.1
0.2
0.3
0.4
raw
roasted
C
cor = 0.82 p.value = 0
roasted
0.00
0.25
0.50
0.75
raw
0.2
0.4
0.6

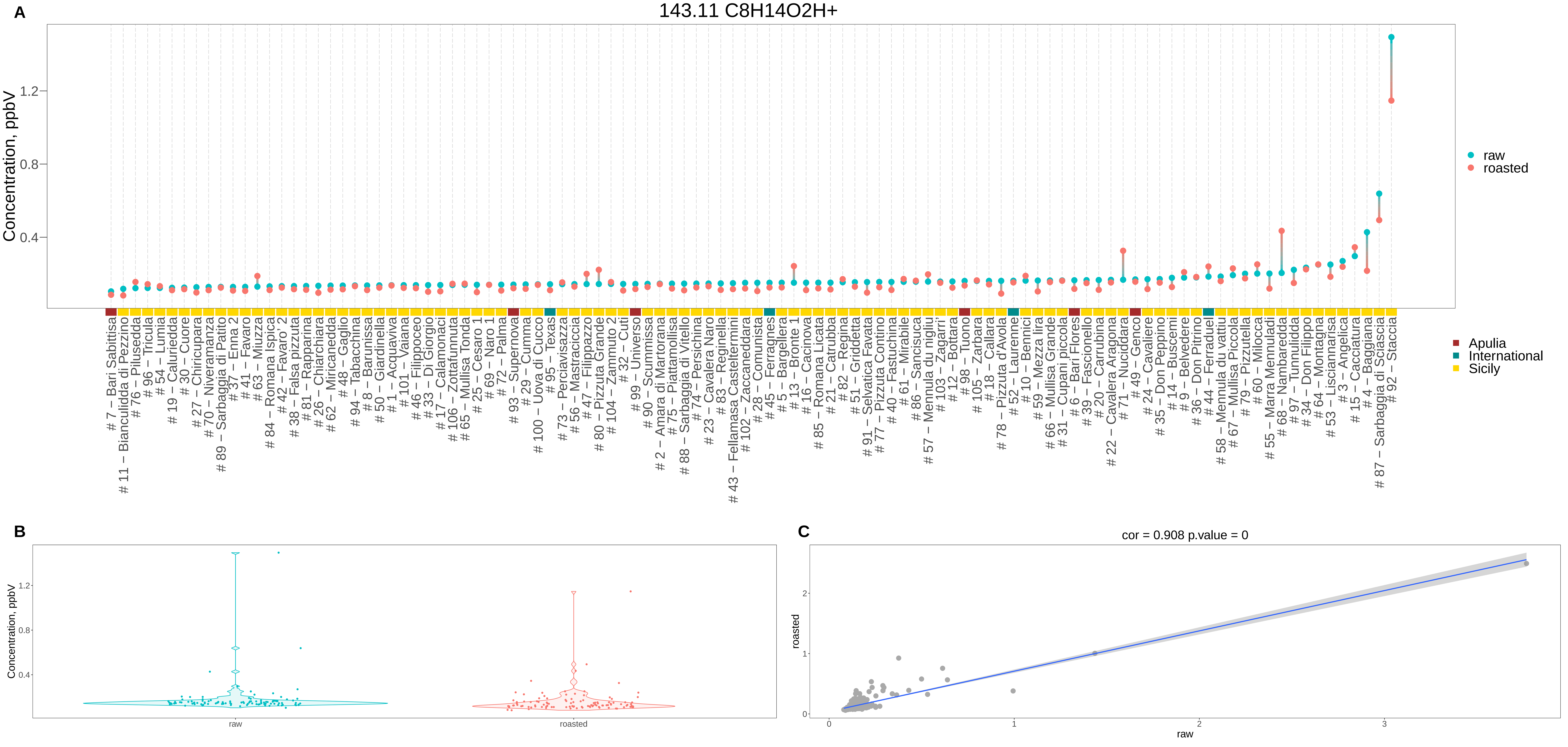
A
143.11 C8H14O2H+
Concentration, ppbV
1.2
0.8
0.4
raw
roasted
Apulia
International
Sicily
B
Concentration, ppbV
1.2
0.8
0.4
raw
roasted
C
cor = 0.908 p.value = 0
roasted
raw
0
1
2
3

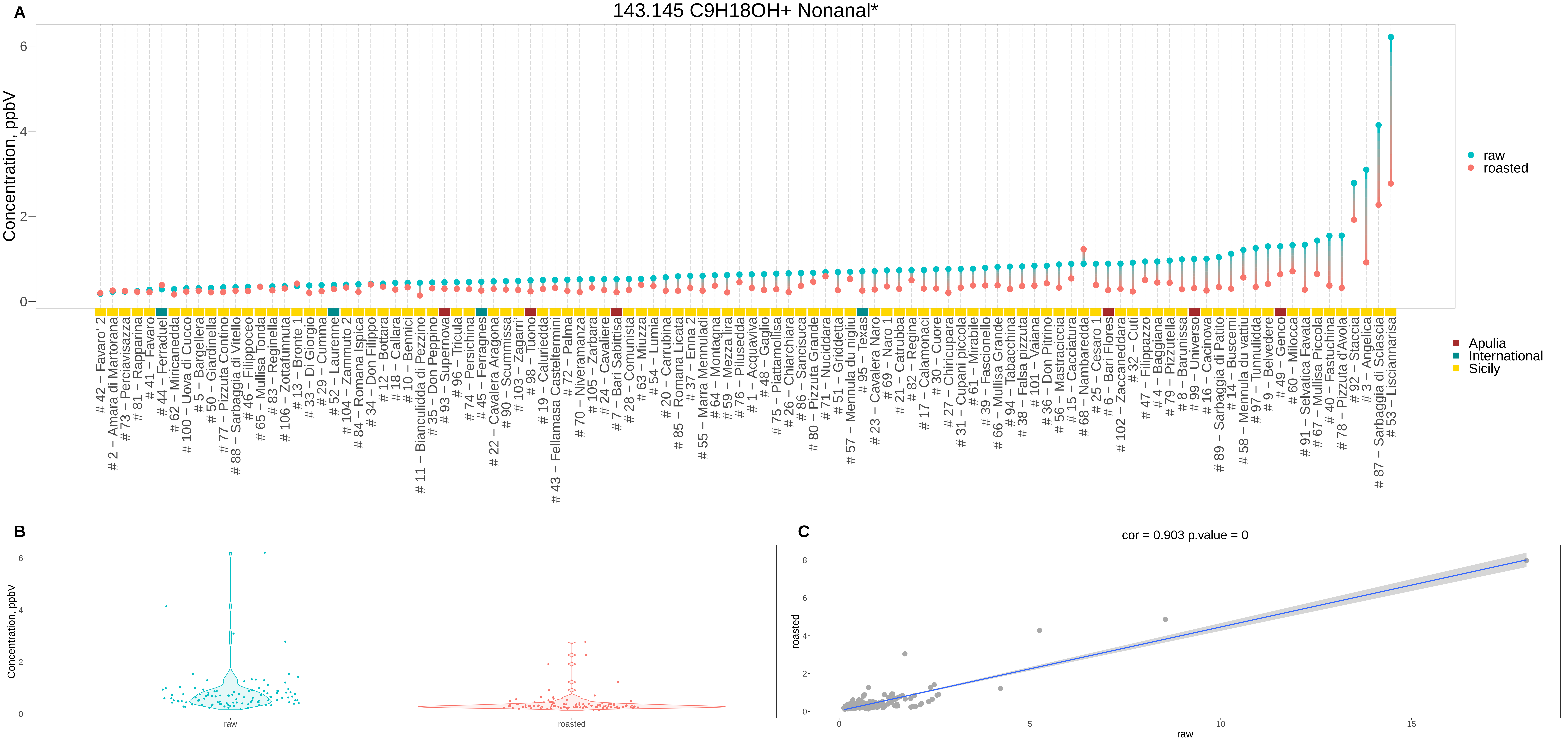
A
143.145 C9H18OH+ Nonanal*
Concentration, ppbV
0
2
4
6
raw
roasted
Apulia
International
Sicily
42 – Favaro' 2
2 – Amara di Martorana
73 – Perciavisazza
81 – Rapparina
41 – Favaro
44 – Ferraduel
62 – Miricanedda
100 – Uova di Cucco
5 – Bargellera
50 – Giardinella
77 – Pizzuta Contino
88 – Sarbaggia di Vitello
46 – Filippoceo
65 – Mullisa Tonda
83 – Reginella
106 – Zottafunnuta
13 – Bronte 1
33 – Di Giorgio
29 – Cumma
52 – Laurenne
104 – Zammuto 2
84 – Romana Ispica
34 – Don Filippo
12 – Bottara
18 – Callara
10 – Bennici
11 – Biancuiidda di Pezzino
35 – Don Peppino
93 – Supernova
96 – Tricula
74 – Persichina
45 – Ferragnes
22 – Cavalera Aragona
90 – Scummissa
103 – Zagarri'
98 – Tuono
19 – Caluriedda
43 – Fellamasa Casteltermini
72 – Palma
70 – Niveramanza
105 – Zarbara
24 – Cavaliere
7 – Bari Sabittisa
28 – Comunista
63 – Mluzza
54 – Lumia
20 – Carrubina
85 – Romana Licata
37 – Enna 2
55 – Marra Mennuladi
64 – Montagna
59 – Mezza lira
76 – Pilusedda
1 – Acquaviva
48 – Gaglio
75 – Piattamollisa
26 – Chiarchiara
86 – Sancisuca
80 – Pizzuta Grande
71 – Nuciddara
51 – Griddetta
57 – Mennula du nigliu
95 – Texas
23 – Cavalera Naro
69 – Naro 1
21 – Catrubba
82 – Regina
17 – Calamonaci
30 – Cuore
27 – Chiricupara
31 – Cupani piccola
61 – Mirabile
39 – Fascionello
66 – Mullisa Grande
94 – Tabacchina
38 – Falsa pizzuta
101 – Vaiana
36 – Don Pitrino
56 – Mastraciccia
15 – Cacciatura
68 – Nambaredda
25 – Cesaro 1
6 – Bari Flores
102 – Zaccanedda
32 – Cuti
47 – Filippazzo
4 – Baggiana
79 – Pizzutella
8 – Barunissa
99 – Universo
16 – Cacinova
89 – Sarbaggia di Patito
14 – Buscemi
58 – Mennula du vattiu
97 – Tunnulidda
9 – Belvedere
49 – Genco
60 – Milocca
91 – Selvatica Favata
67 – Mullisa Piccola
40 – Fastuchina
78 – Pizzuta d'Avola
92 – Staccia
3 – Angelica
87 – Sarbaggia di Sciascia
53 – Liscinnarisa
B
Concentration, ppbV
0
2
4
6
raw
roasted
C
cor = 0.903 p.value = 0
roasted
0
2
4
6
8
0
5
10
15
raw

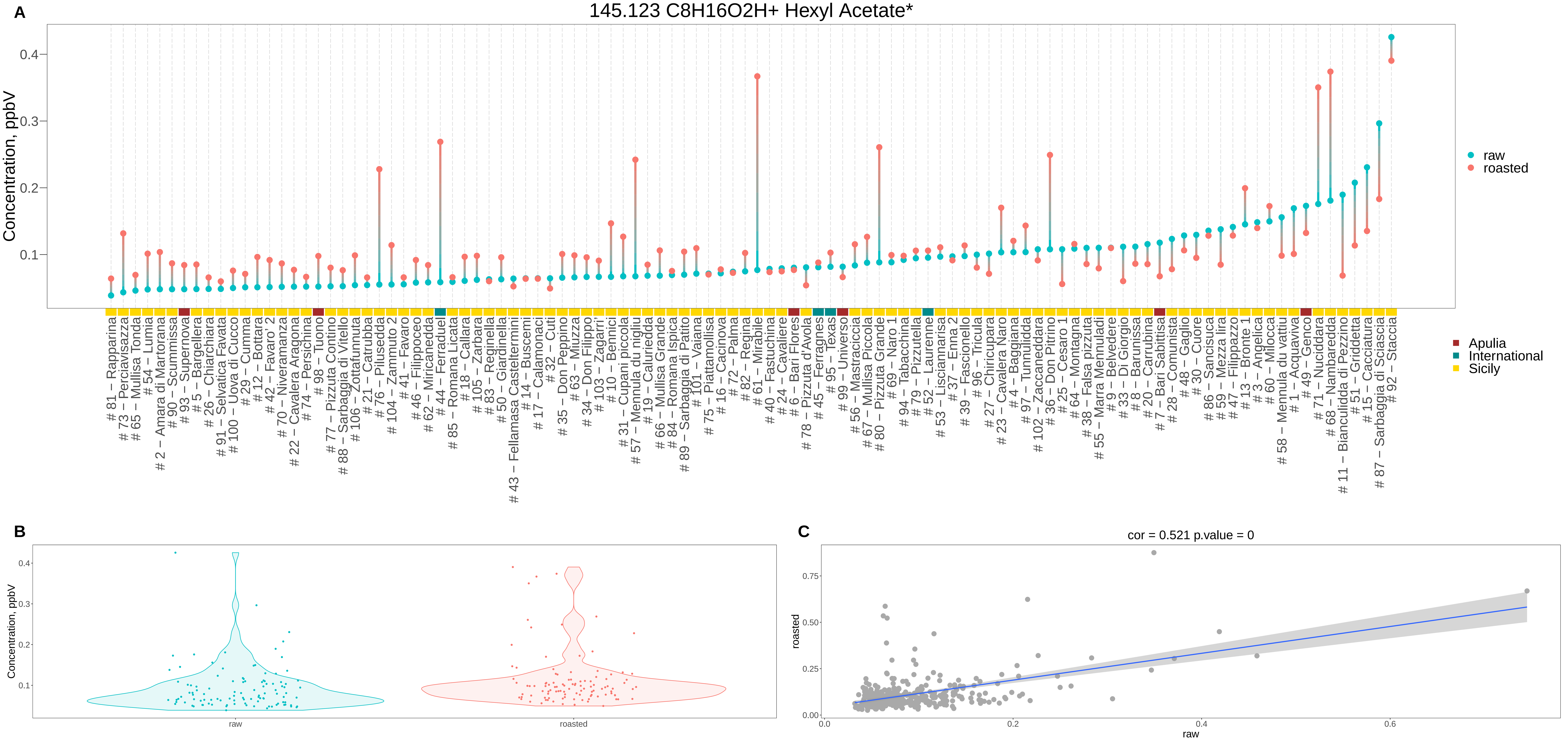
A
145.123 C8H16O2H+ Hexyl Acetate*
Concentration, ppbV
0.4
0.3
0.2
0.1
raw
roasted
Apulia
International
Sicily
81 – Rappanina
73 – Perciavisazza
65 – Mullisa Tonda
54 – Lumia
2 – Amara di Martorana
90 – Scummissa
93 – Supernova
5 – Bargellera
26 – Chiarchiara
91 – Selvatica Favata
100 – Uova di Cucco
29 – Cumma
12 – Bottara
42 – Favaro' 2
70 – Niveramanza
22 – Cavalera Aragona
74 – Persichina
98 – Tuono
77 – Pizzuta Contino
88 – Sarbaggia di Vitello
106 – Zottafunnuta
21 – Catrubba
76 – Pilusedda
104 – Zammuto 2
41 – Favaro
46 – Filippoceo
62 – Miricanedda
44 – Ferraduel
85 – Romana Licata
18 – Callara
105 – Zarbara
83 – Reginella
50 – Giardinella
43 – Fellamasa Casteltermini
14 – Buscemi
17 – Calamonaci
32 – Cuti
35 – Don Peppino
63 – Mluzza
34 – Don Filippo
103 – Zagarri'
10 – Bennici
31 – Cupani piccola
57 – Mennula du nigliu
19 – Caluriedda
66 – Mullisa Grande
84 – Romana Ispica
89 – Sarbaggia di Patito
101 – Vaiana
75 – Piattamollisa
16 – Cacinova
72 – Palma
82 – Regina
61 – Mirabile
40 – Fastuchina
24 – Cavaliere
6 – Bari Flores
78 – Pizzuta d'Avola
45 – Ferragnes
95 – Texas
99 – Universo
56 – Mastracicca
67 – Mullisa Piccola
80 – Pizzuta Grande
69 – Naro 1
94 – Tabacchina
79 – Pizzutella
52 – Laurenne
53 – Liscianarisa
37 – Enna 2
39 – Fascionello
96 – Tricula
27 – Chiricupara
23 – Cavalera Naro
4 – Baggiana
97 – Tunnulidda
102 – Zaccaneddara
36 – Don Pitrino
25 – Cesaro 1
64 – Montagna
38 – Falsa pizzuta
55 – Marra Mennuladi
9 – Belvedere
33 – Di Giorgio
8 – Barunissa
20 – Carrubina
7 – Bari Sabittisa
28 – Comunista
48 – Gaglio
30 – Cuore
86 – Sancisuca
59 – Mezza lira
47 – Filipazzo
13 – Bronte 1
3 – Angelica
60 – Milocca
58 – Mennula du vattiu
1 – Acquaviva
49 – Genco
71 – Nuciddara
68 – Nambaredda
11 – Bianculidda di Pezzino
51 – Griddetta
15 – Cacciatura
87 – Sarbaggia di Sciascia
92 – Staccia
B
Concentration, ppbV
0.4
0.3
0.2
0.1
raw
roasted
C
cor = 0.521 p.value = 0
roasted
0.75
0.50
0.25
0.00
0.0
0.2
0.4
0.6
raw

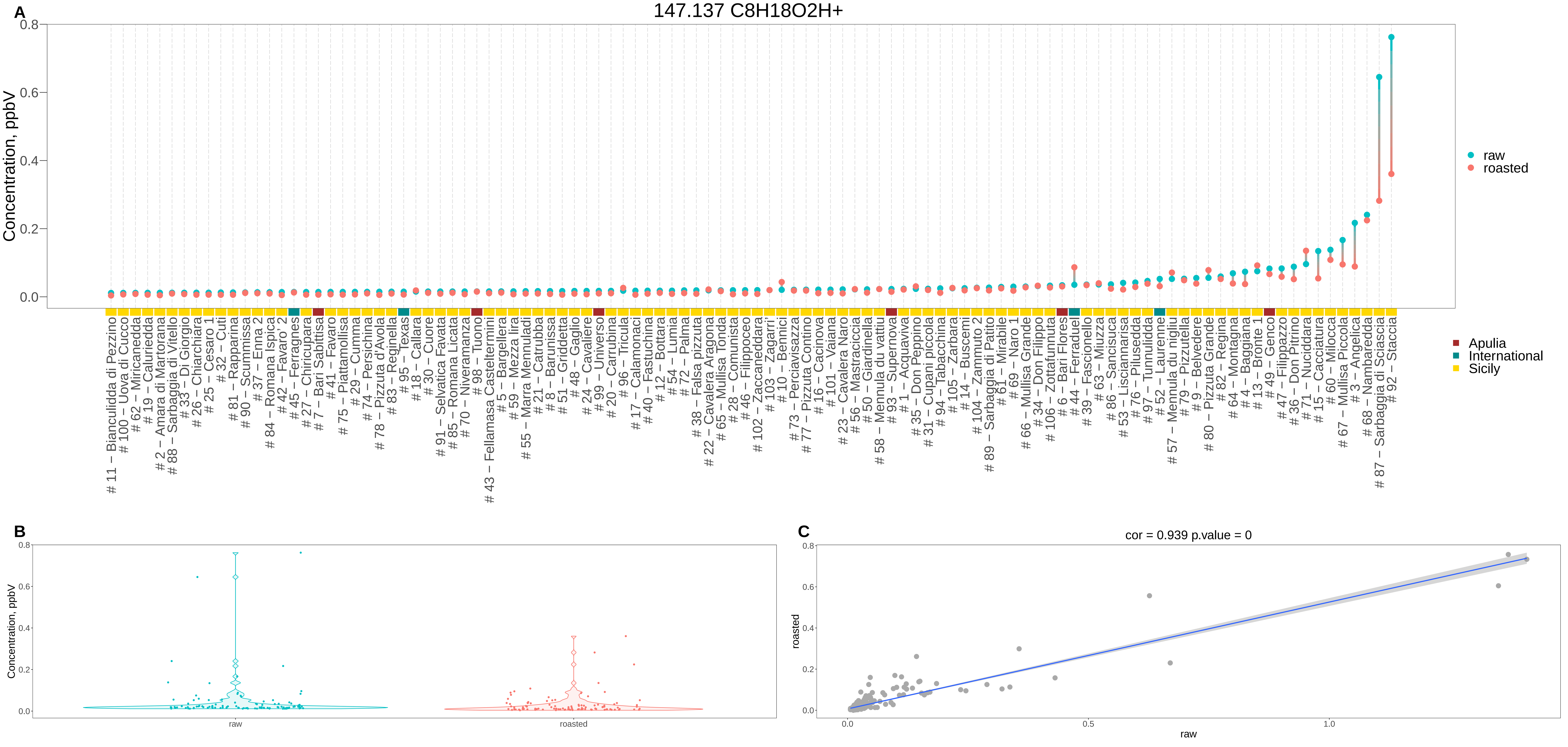

A
147.137 C8H18O2H+
Concentration, ppbV
raw
roasted
Apulia
International
Sicily
B
Concentration, ppbV
raw
roasted
C
cor = 0.939 p.value = 0
roasted
raw

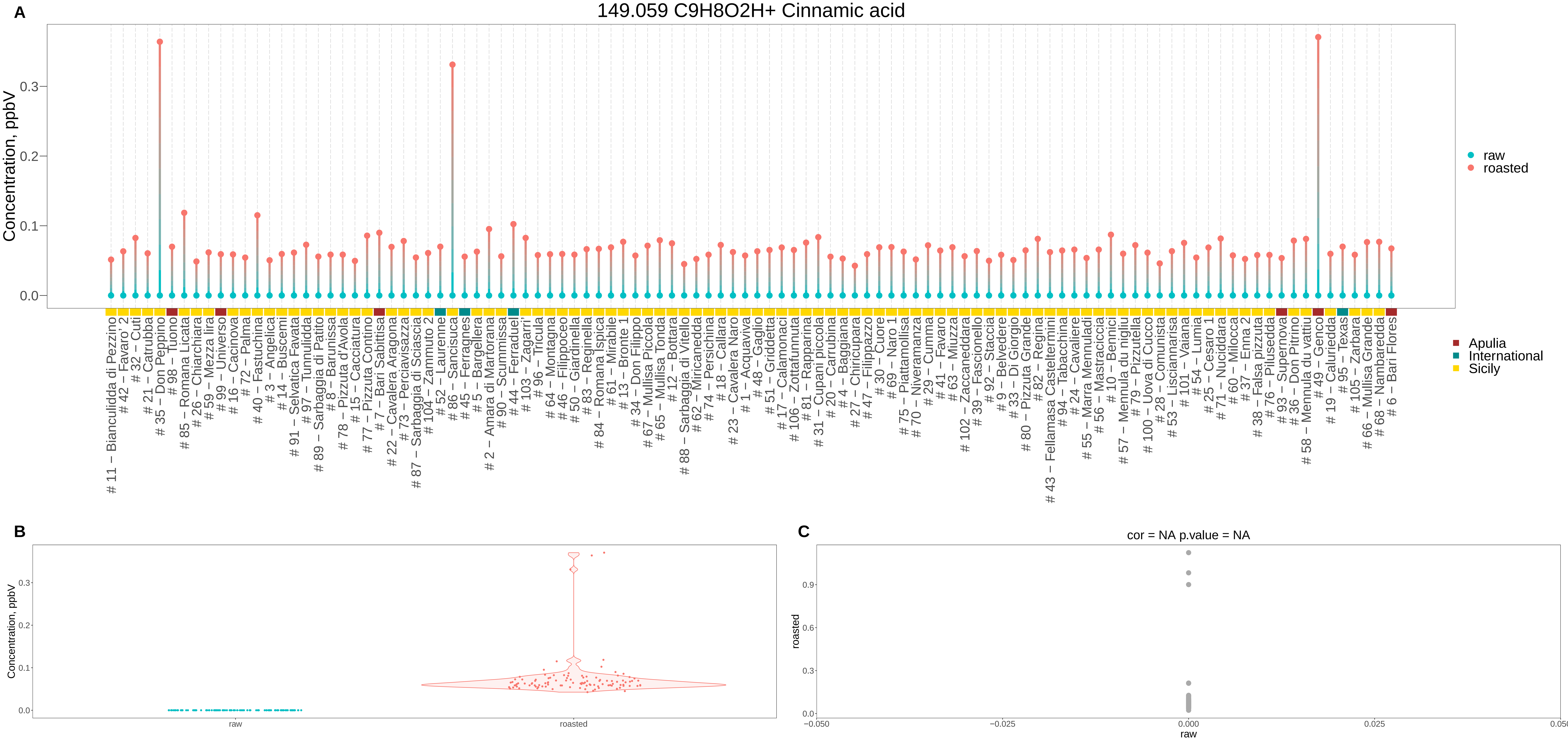
A
149.059 C9H8O2H+ Cinnamic acid
Concentration, ppbV
raw
roasted
Apulia
International
Sicily
B
Concentration, ppbV
raw
roasted
C
cor = NA p.value = NA
roasted
raw

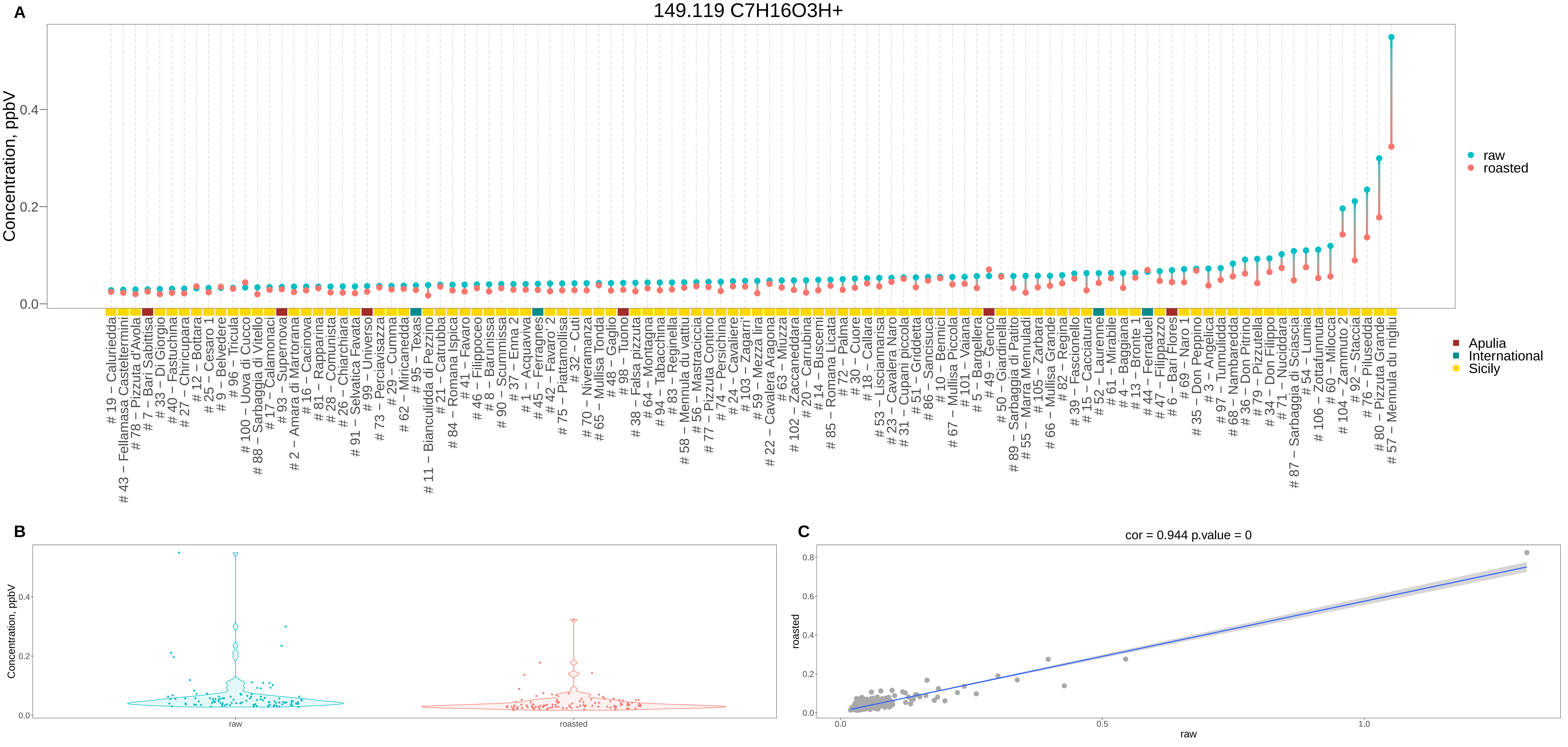

A
149.119 C7H16O3H+
Concentration, ppbV
0.0
0.2
0.4
raw
roasted
Apulia
International
Sicily
19 – Caluriedda
43 – Fellamasa Casteltermini
78 – Pizzuta d'Avola
7 – Bari Sabittisa
33 – Di Giorgio
40 – Fastuchina
27 – Chiricupara
12 – Bottara
25 – Cesaro 1
9 – Belvedere
96 – Tricula
100 – Uova di Cucco
88 – Sarbaggia di Vitello
17 – Calamonaci
93 – Supernova
2 – Amara di Martorana
16 – Cacinova
81 – Rapparina
28 – Comunista
26 – Chiarchiara
91 – Selvatica Favata
99 – Universo
73 – Perciavisazza
29 – Cumma
62 – Miricanedda
95 – Texas
11 – Bianculidda di Pezzino
21 – Catrubba
84 – Romana Ispica
41 – Favaro
46 – Filippoceo
8 – Barunissa
90 – Scummissa
37 – Enna 2
1 – Acquaviva
45 – Ferragnes
42 – Favaro' 2
75 – Piattamollisa
32 – Cuti
70 – Niveramanza
65 – Mullisa Tonda
48 – Gaglio
98 – Tuono
38 – Falsa pizzuta
64 – Montagna
94 – Tabacchina
83 – Reginella
58 – Mennula du vattiu
56 – Mastraciccia
77 – Pizzuta Contino
74 – Persichina
24 – Cavaliere
103 – Zagarri'
59 – Mezza lira
22 – Cavalera Aragona
63 – Miuzza
102 – Zaccaneddara
20 – Carrubina
14 – Buscemi
85 – Romana Licata
72 – Palma
30 – Cuore
18 – Callara
53 – Lisciannarisa
23 – Cavalera Naro
31 – Cupani piccola
51 – Griddetta
86 – Sancisuca
10 – Bennici
67 – Mullisa Piccola
101 – Vaiana
5 – Bargellera
49 – Genco
50 – Giardinella
89 – Sarbaggia di Patito
55 – Marra Mennuladi
105 – Zarbara
66 – Mullisa Grande
82 – Regina
39 – Fascionello
15 – Cacciatura
52 – Laurenne
61 – Mirabile
4 – Baggiana
13 – Bronte 1
44 – Ferraduel
47 – Filippazzo
6 – Bari Flores
69 – Naro 1
35 – Don Peppino
3 – Angelica
97 – Tunnulidda
68 – Nambaredda
36 – Don Pitrino
79 – Pizzutella
34 – Don Filippo
71 – Nuciddara
87 – Sarbaggia di Sciascia
54 – Lumia
106 – Zottafunnuta
60 – Milocca
104 – Zammuto 2
92 – Staccia
76 – Pilusedda
80 – Pizzuta Grande
57 – Mennula du nigliu
B
Concentration, ppbV
0.0
0.2
0.4
raw
roasted
C
cor = 0.944 p.value = 0
roasted
0.0
0.2
0.4
0.6
0.8
0.0
0.5
1.0
raw

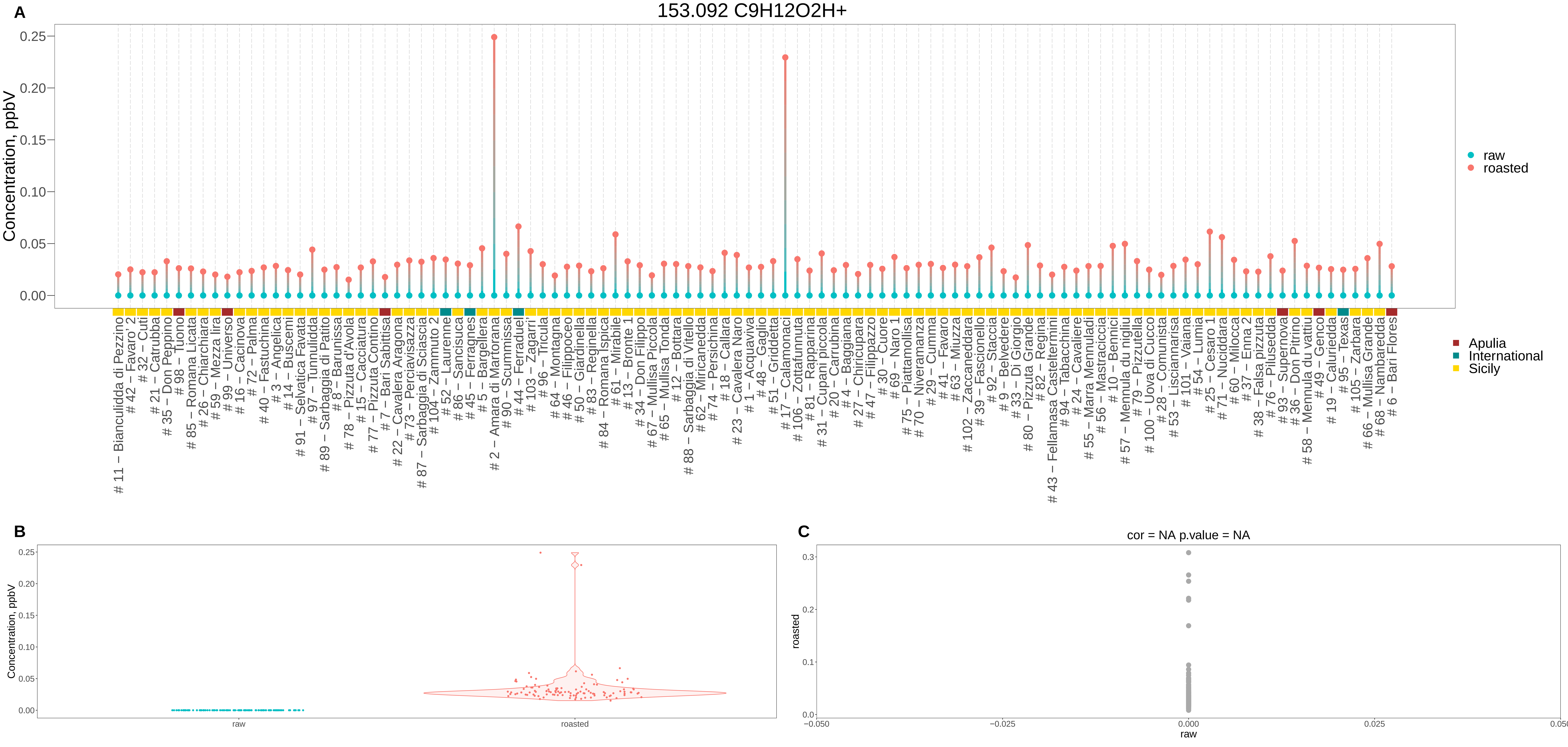
A
153.092 C9H12O2H+
Concentration, ppbV
0.25
0.20
0.15
0.10
0.05
0.00
raw
roasted
Apulia
International
Sicily
11 – Bianculidda di Pezzino
42 – Favaro' 2
32 – Cuti
21 – Catrubba
35 – Don Peppino
98 – Tuono
85 – Romana Licata
26 – Chiarchiara
59 – Mezza lira
99 – Universo
16 – Cacinova
72 – Palma
40 – Fastuchina
3 – Angelica
14 – Buscemi
91 – Selvatica Favata
97 – Tunnulidda
89 – Sarbaggia di Patito
8 – Barunissa
78 – Pizzuta d'Avola
15 – Cacciatura
77 – Pizzuta Contino
7 – Bari Sabitisa
22 – Cavalera Aragona
73 – Perciavisazza
87 – Sarbaggia di Sciascia
104 – Zammuto 2
52 – Laurenne
86 – Sancisuca
45 – Ferragnes
5 – Bargellera
2 – Amara di Martorana
90 – Scummissa
44 – Ferraduel
103 – Zagarri'
96 – Tricula
64 – Montagna
46 – Filippoceo
50 – Giardinella
83 – Reginella
84 – Romana Ispica
61 – Mirabile
13 – Bronte 1
34 – Don Filippo
67 – Mullisa Piccola
65 – Mullisa Tonda
12 – Bottara
88 – Sarbaggia di Vitello
62 – Miricanedda
74 – Persichina
18 – Callara
23 – Cavalera Naro
1 – Acquaviva
48 – Gaglio
51 – Griddetta
17 – Calamonaci
106 – Zottafunnuta
81 – Rapparina
31 – Cupani piccola
20 – Carrubina
4 – Baggiana
27 – Chiricupara
47 – Filippazzo
30 – Cuore
69 – Naro 1
75 – Piattamollisa
70 – Niveramanza
29 – Cumma
41 – Favaro
63 – Miuzza
102 – Zaccaneddara
39 – Fascionello
92 – Staccia
9 – Belvedere
33 – Di Giorgio
80 – Pizzuta Grande
82 – Regina
43 – Fellamasa Casteltermini
94 – Tabacchina
24 – Cavaliere
55 – Marra Mennuladi
56 – Mastraciccia
10 – Bennici
57 – Mennula du nigliu
79 – Pizzutella
100 – Uova di Cucco
28 – Comunista
53 – Liscionnarisa
101 – Vaiana
54 – Lumia
25 – Cesaro 1
71 – Nuciddara
60 – Milocca
37 – Enna 2
38 – Falsa pizzuta
76 – Pilusedda
93 – Supernova
36 – Don Pitrino
58 – Mennula du vattiu
49 – Genco
19 – Caluriedda
95 – Texas
105 – Zarbara
66 – Mullisa Granede
68 – Nambaredda
6 – Bari Flores
B
Concentration, ppbV
0.25
0.20
0.15
0.10
0.05
0.00
raw
roasted
C
cor = NA p.value = NA
roasted
0.3
0.2
0.1
0.0
−0.050
−0.025
0.000
0.025
0.050
raw

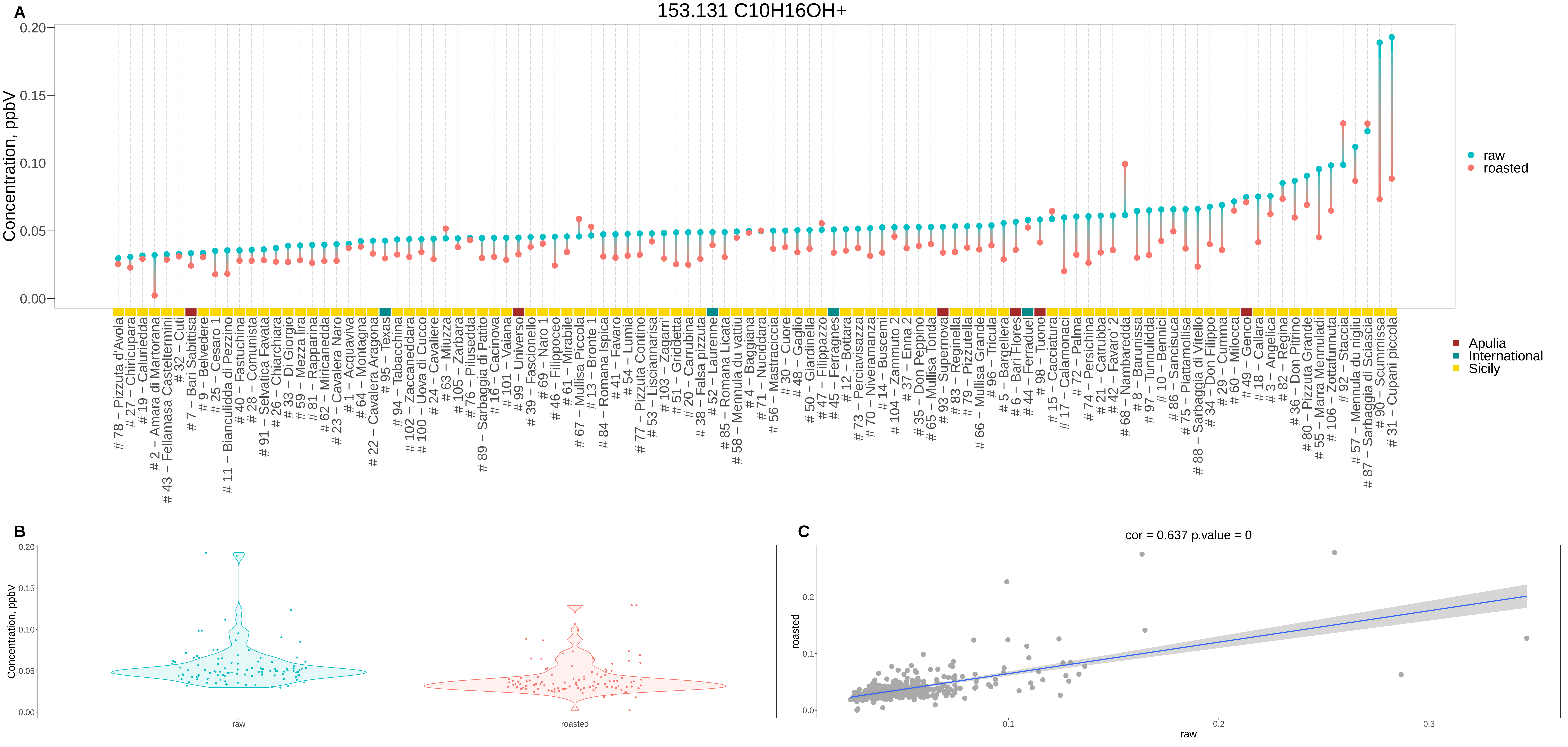
A
153.131 C10H16OH+
Concentration, ppbV
0.00
0.05
0.10
0.15
0.20
raw
roasted
Apulia
International
Sicily
78 – Pizzuta d'Avola
27 – Chiricupara
19 – Caluriedda
2 – Amara di Martorana
43 – Fellamasa Casteltermini
32 – Cuti
7 – Bari Sabitissa
9 – Belvedere
25 – Cesaro 1
11 – Bianculidda di Pezzino
40 – Fastuchina
28 – Comunista
91 – Selvatica Favata
26 – Chiarchiara
33 – Di Giorgio
59 – Mezza lira
81 – Rapparina
62 – Miricanedda
23 – Cavalera Naro
1 – Acquaviva
64 – Montagna
22 – Cavalera Aragona
95 – Texas
94 – Tabacchina
102 – Zaccaneddara
100 – Uova di Cucco
24 – Cavaliere
63 – Miuzza
105 – Zarbara
76 – Pilusedda
89 – Sarbaggia di Patito
16 – Cacinova
101 – Vaiana
99 – Universo
39 – Fascionello
69 – Naro 1
46 – Filippoceo
61 – Mirabile
67 – Mullisa Piccola
13 – Bronte 1
84 – Romana Ispica
41 – Favaro
54 – Lumia
77 – Pizzuta Contino
53 – Liscianarisa
103 – Zagarri'
51 – Griddetta
20 – Carrubina
38 – Falsa pizzuta
52 – Laurenne
85 – Romana Licata
58 – Mennula du vattiu
4 – Baggiana
71 – Nuciddara
56 – Mastracicia
30 – Cuore
48 – Gaglio
50 – Giardinella
47 – Filippazzo
45 – Ferragnes
12 – Bottara
73 – Perciavisazza
70 – Niveramanza
14 – Buscemi
104 – Zammuto 2
37 – Enna 2
35 – Don Peppino
65 – Mullisa Tonda
93 – Supernova
83 – Reginella
79 – Pizzutella
66 – Mullisa Grande
96 – Tricula
5 – Bargellera
6 – Bari Flores
44 – Ferraduel
98 – Tuono
15 – Cacciatura
17 – Calamonaci
72 – Palma
74 – Persichina
21 – Catrubba
42 – Favaro' 2
68 – Nambaredda
8 – Barunissa
97 – Tunnulidda
10 – Bennici
86 – Sancisuca
75 – Piattamollisa
88 – Sarbaggia di Vitello
34 – Don Filippo
29 – Cumma
60 – Milocca
49 – Genco
18 – Callara
3 – Angelica
82 – Regina
36 – Don Pitrino
80 – Pizzuta Grande
55 – Marra Mennuladi
106 – Zottafunnuta
92 – Staccia
57 – Mennula du nigliu
87 – Sarbaggia di Sciascia
90 – Scummissa
31 – Cupani piccola
B
Concentration, ppbV
0.00
0.05
0.10
0.15
0.20
raw
roasted
C
cor = 0.637 p.value = 0
roasted
0.0
0.1
0.2
raw
0.1
0.2
0.3

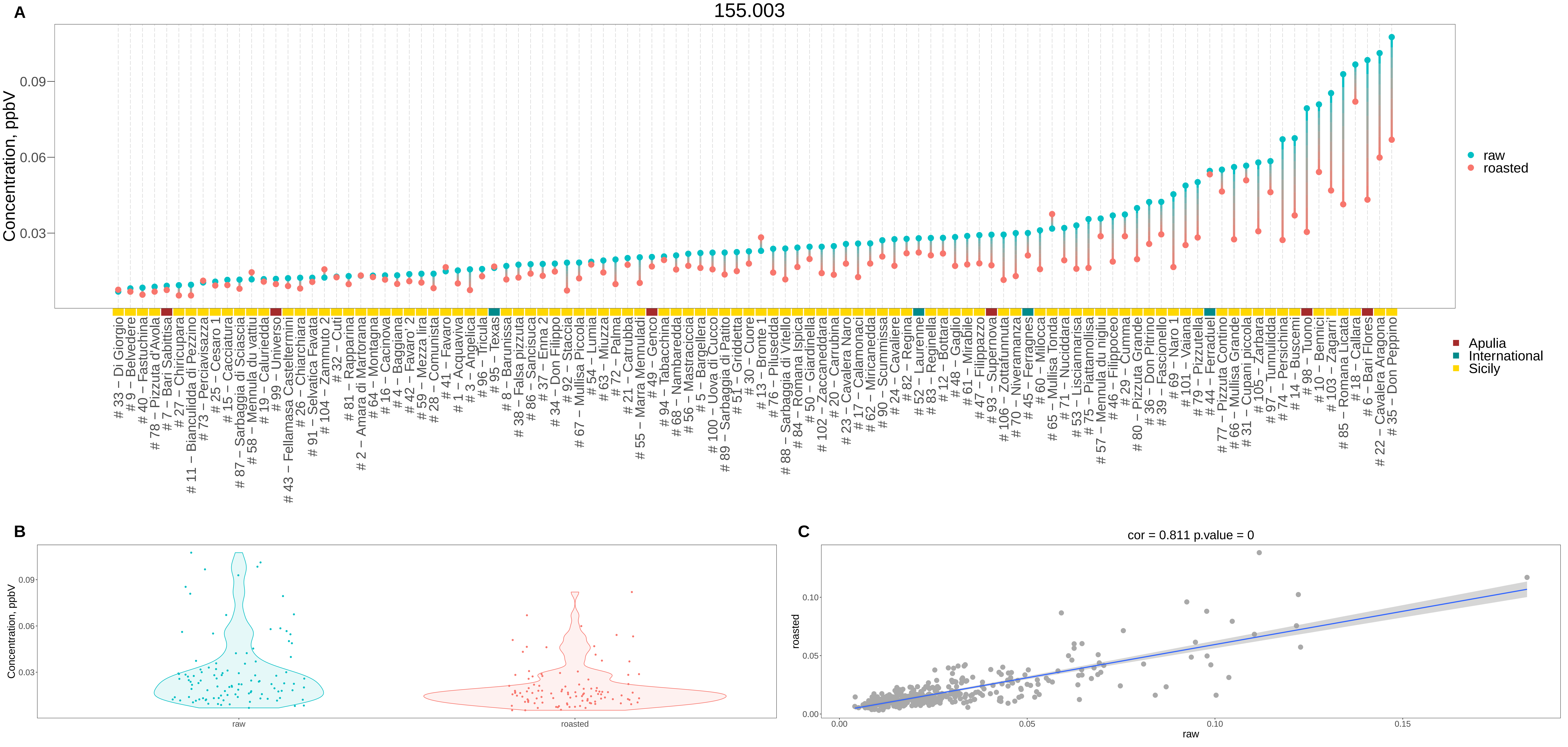
A
155.003
Concentration, ppbV
0.09
0.06
0.03
raw
roasted
Apulia
International
Sicily
33 – Di Giorgio
9 – Belvedere
40 – Fastuchina
78 – Pizzuta d'Avola
7 – Bari Sabittisa
27 – Chiricupara
11 – Biancullidda di Pezzino
73 – Perciavisazza
25 – Cesaro 1
15 – Cacciatura
87 – Sarbaggia di Sciascia
58 – Mennula du vattiu
19 – Calurriedda
99 – Universo
43 – Fellamasa Casteltermini
26 – Chiarchiara
91 – Selvatica Favata
104 – Zammuto 2
32 – Cuti
81 – Rapparina
2 – Amara di Martorana
64 – Montagna
16 – Cacinova
4 – Baggiana
42 – Favaro' 2
59 – Mezza lira
28 – Comunista
41 – Favaro
1 – Acquaviva
3 – Angelica
96 – Tricula
95 – Texas
8 – Barunissa
38 – Falsa pizzuta
86 – Sancisuca
37 – Enna 2
34 – Don Filippo
92 – Staccia
67 – Mullisa Piccola
54 – Lumia
63 – Miuzza
72 – Palma
21 – Catrubba
55 – Marra Mennuladi
49 – Genco
94 – Tabacchina
68 – Nambaredda
56 – Mastracicia
5 – Bargellera
100 – Uova di Cucco
89 – Sarbaggia di Patito
51 – Griddetta
30 – Cuore
13 – Bronte 1
76 – Pilusedda
88 – Sarbaggia di Vitello
84 – Romana Ispica
50 – Giardinella
102 – Zaccaneddara
20 – Carrubina
23 – Cavalera Naro
17 – Calamonaci
62 – Miricanedda
90 – Scummissa
24 – Cavaliere
82 – Regina
52 – Laurenne
83 – Reginella
12 – Bottara
48 – Gaglio
61 – Mirabile
47 – Filippazzo
93 – Supernova
106 – Zottafunnuta
70 – Niveramanza
45 – Ferragnes
60 – Milocca
65 – Mullisa Tonda
71 – Nuciddara
53 – Liscianarisa
75 – Piattamollisa
57 – Mennula du nigliu
46 – Filippoceo
29 – Cumma
80 – Pizzuta Grande
36 – Don Pitrino
39 – Fascionello
69 – Naro 1
101 – Vaiana
79 – Pizzutella
44 – Ferraduel
77 – Pizzuta Contino
66 – Mullisa Grande
31 – Cupani piccola
105 – Zarbara
97 – Tunnulidda
74 – Persichina
14 – Buscemi
98 – Tuono
10 – Bennici
103 – Zagarri'
85 – Romana Licata
18 – Callara
6 – Bari Flores
22 – Cavalera Aragona
35 – Don Peppino
B
Concentration, ppbV
0.09
0.06
0.03
raw
roasted
C
cor = 0.811 p.value = 0
roasted
0.10
0.05
0.00
0.00
0.05
0.10
0.15
raw

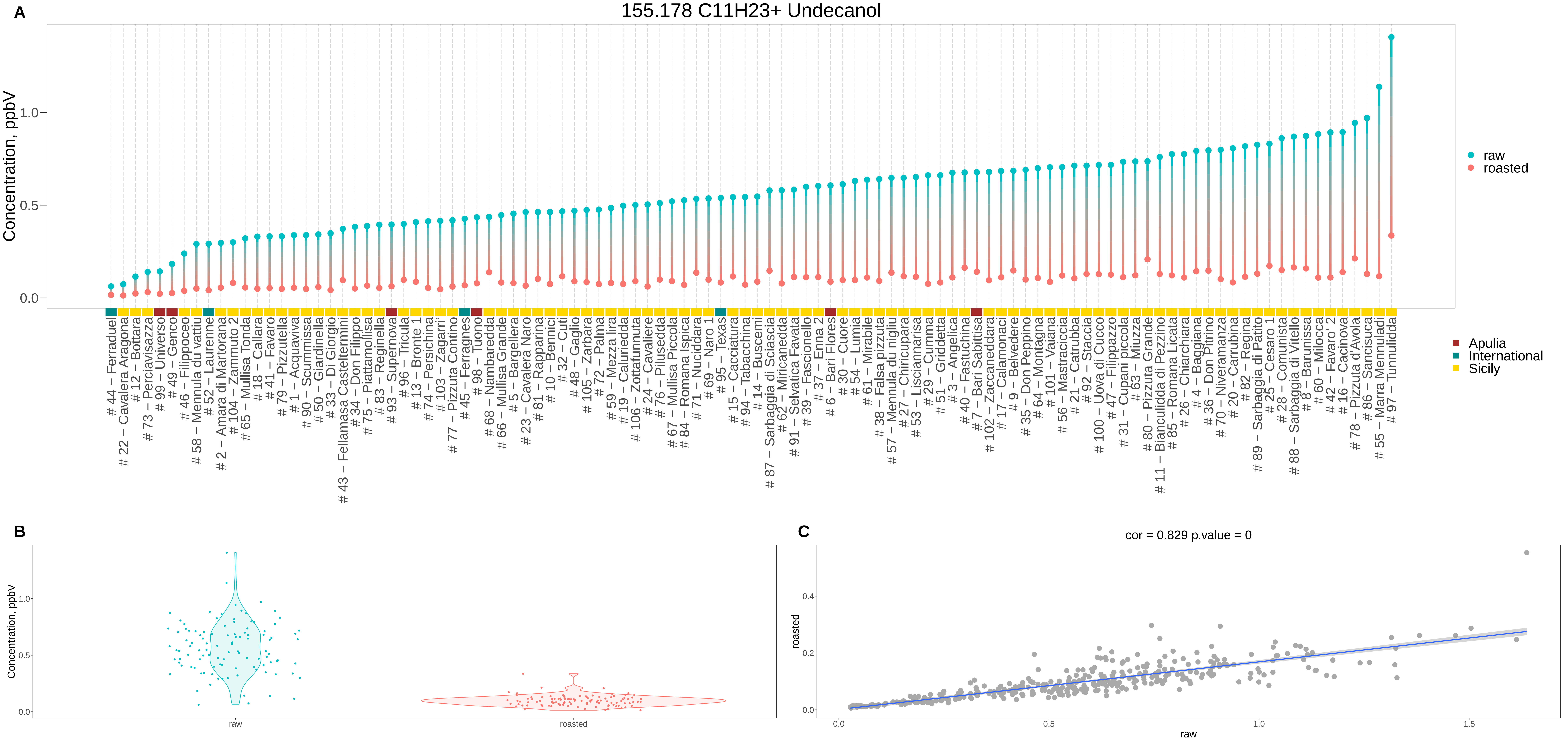
A
155.178 C11H23+ Undecanol
Concentration, ppbV
0.0
0.5
1.0
raw
roasted
Apulia
International
Sicily
44 – Ferraduel
22 – Cavalera Aragona
12 – Bottara
73 – Perciavisazza
99 – Universo
49 – Genco
46 – Filippoceo
58 – Mennula du vattiu
52 – Laurenne
2 – Amara di Martorana
104 – Zammuto 2
65 – Mullisa Tonda
18 – Callara
41 – Favaro
79 – Pizzutella
1 – Acquaviva
90 – Scummissa
50 – Giardinella
33 – Di Giorgio
43 – Fellamasa Castelltermini
34 – Don Filippo
75 – Piattamollisa
83 – Reginella
93 – Supernova
96 – Tricula
13 – Bronte 1
74 – Persichina
103 – Zagarri'
77 – Pizzuta Contino
45 – Ferragnes
98 – Tuono
68 – Nambaredda
66 – Mullisa Grande
5 – Bargellera
23 – Cavalera Naro
81 – Rapparina
10 – Bennici
32 – Cuti
48 – Gaglio
105 – Zarbara
72 – Palma
59 – Mezza lira
19 – Caluriedda
106 – Zottafunnuta
24 – Cavaliere
76 – Pilusedda
67 – Mullisa Piccola
84 – Romana Ispica
71 – Nuciddara
69 – Naro 1
95 – Texas
15 – Cacciatura
94 – Tabacchina
14 – Buscemi
87 – Sarbaggia di Sciascia
62 – Miricanedda
91 – Selvatica Favata
39 – Fascionello
37 – Enna 2
6 – Bari Flores
30 – Cuore
54 – Lumia
61 – Mirabile
38 – Falsa pizzuta
57 – Mennula du nigliu
27 – Chiricupara
53 – Liscianarisa
29 – Cumma
51 – Griddetta
3 – Angelica
40 – Fastuchina
7 – Bari Sabittisa
102 – Zaccaneddara
17 – Calamonaci
9 – Belvedere
35 – Don Peppino
64 – Montagna
101 – Vaiana
56 – Mastraciccia
21 – Catrubba
92 – Staccia
100 – Uova di Cucco
47 – Filippazzo
31 – Cupani piccola
63 – Miuzza
80 – Pizzuta Grande
11 – Bianculidda di Pezzino
85 – Romana Licata
26 – Chiarchiara
4 – Baggiana
36 – Don Pitrino
70 – Niveramanza
20 – Carrubina
82 – Regina
89 – Sarbaggia di Patito
25 – Cesaro 1
28 – Comunista
88 – Sarbaggia di Vitello
8 – Barunissa
60 – Milocca
42 – Favaro' 2
16 – Cacinova
78 – Pizzuta d'Avola
86 – Sancisuca
55 – Marra Mennuladi
97 – Tunnulidda
B
Concentration, ppbV
0.0
0.5
1.0
raw
roasted
C
cor = 0.829 p.value = 0
roasted
0.0
0.2
0.4
raw
0.0
0.5
1.0
1.5

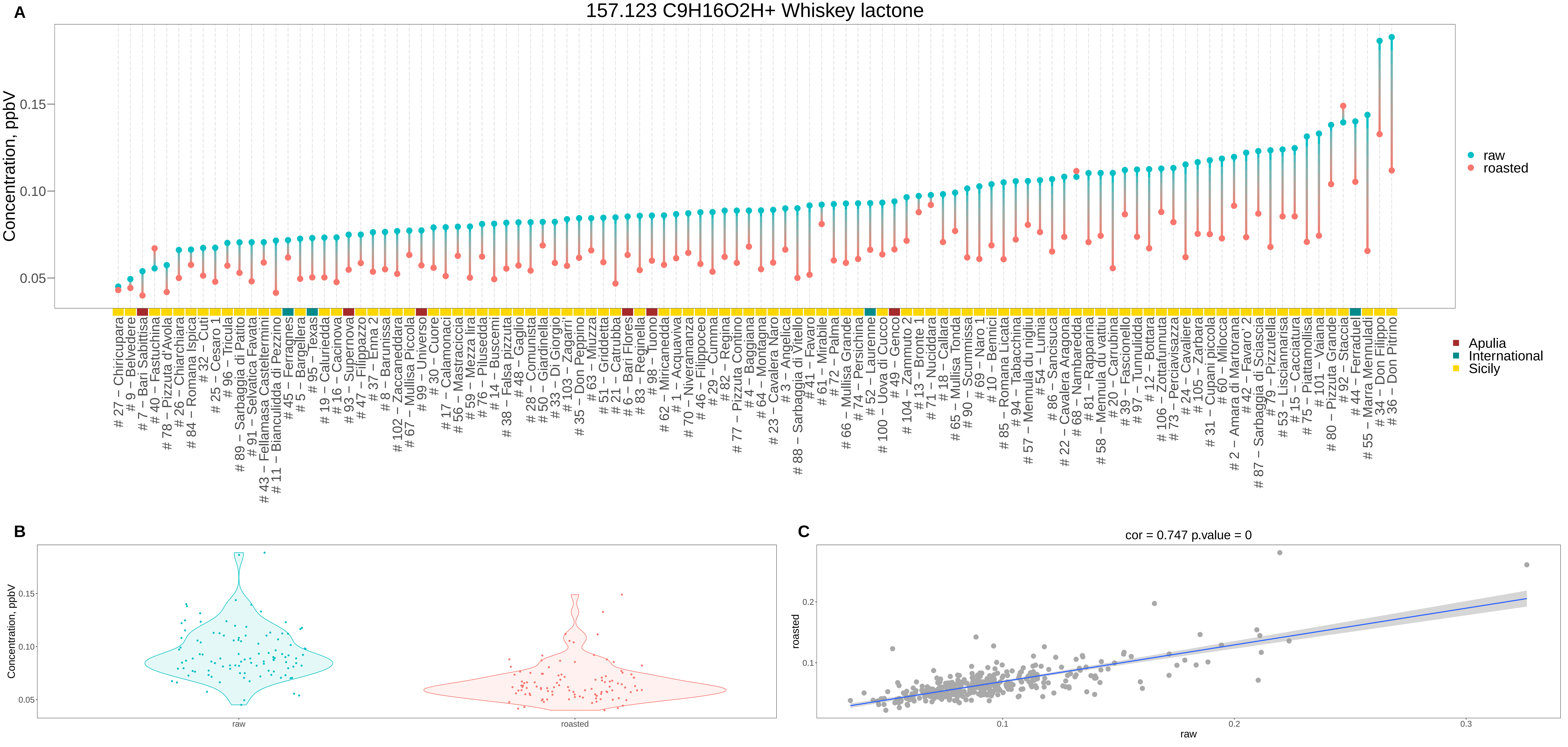

A
157.123 C9H16O2H+ Whiskey lactone
Concentration, ppbV
0.05
0.10
0.15
raw
roasted
Apulia
International
Sicily
27 – Chiricupara
9 – Belvedere
7 – Bari Sabitista
40 – Fastuchina
78 – Pizzuta d'Avola
26 – Chiarchiara
84 – Romana Ispica
32 – Cuti
25 – Cesaro 1
96 – Tricula
89 – Sarbaggia di Patito
91 – Selvatica Favata
43 – Fellamasa Casteltermini
11 – Bianculidda di Pezzino
45 – Ferragnes
5 – Bargellera
95 – Texas
19 – Caluriedda
16 – Cacinova
93 – Supernova
47 – Filippazzo
37 – Enna 2
8 – Barunissa
102 – Zaccaneddara
67 – Mullisa Piccola
99 – Universo
30 – Cuore
17 – Calamonaci
56 – Mastraciccia
59 – Mezza lira
76 – Pilusedda
14 – Buscemi
38 – Falsa pizzuta
48 – Gaglio
28 – Comunista
50 – Giardinella
33 – Di Giorgio
103 – Zagarri'
35 – Don Peppino
63 – Miuzza
51 – Griddetta
21 – Catrubba
6 – Bari Flores
83 – Reginella
98 – Tuono
62 – Miricanedda
1 – Acquaviva
70 – Niveramanza
46 – Filippoceo
29 – Cumma
82 – Regina
77 – Pizzuta Contino
4 – Baggiana
64 – Montagna
23 – Cavalera Naro
3 – Angelica
88 – Sarbaggia di Vitello
41 – Favaro
61 – Mirabile
72 – Palma
66 – Mullisa Grande
74 – Persichina
52 – Laurenne
100 – Uova di Cucco
49 – Genco
104 – Zammuto 2
13 – Bronte 1
71 – Nuciddara
18 – Callara
65 – Mullisa Tonda
90 – Scummissa
69 – Naro 1
10 – Bennici
85 – Romana Licata
94 – Tabacchina
57 – Mennula du nigliu
54 – Lumia
86 – Sancisuca
22 – Cavalera Aragona
68 – Nambaredda
81 – Rapparina
58 – Mennula du vattiu
20 – Carrubina
39 – Fascionello
97 – Tunnulidda
12 – Bottara
106 – Zottafunnuta
73 – Perciavisazza
24 – Cavaliere
105 – Zarbara
31 – Cupani piccola
60 – Milocca
2 – Amara di Martorana
42 – Favaro 2
87 – Sarbaggia di Sciascia
79 – Pizzutella
53 – Liscianarisa
15 – Cacciatura
75 – Piattamollisa
101 – Vaiana
80 – Pizzuta Grande
92 – Staccia
44 – Ferraduel
55 – Marra Mennuladi
34 – Don Filippo
36 – Don Pitrino
B
Concentration, ppbV
0.05
0.10
0.15
raw
roasted
C
cor = 0.747 p.value = 0
roasted
0.1
0.2
raw
0.1
0.2
0.3

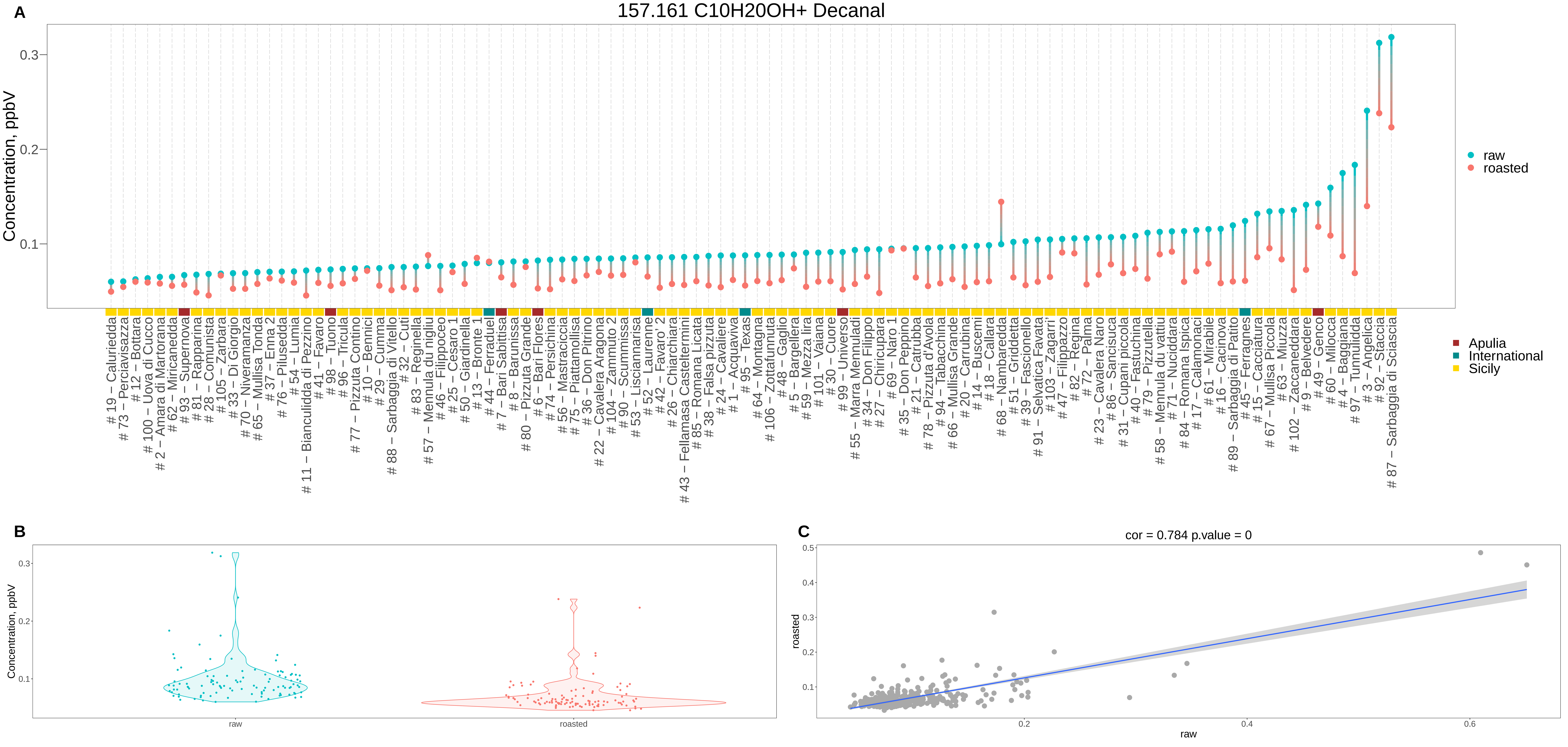

A
157.161 C10H20OH+ Decanal
Concentration, ppbV
0.1
0.2
0.3
raw
roasted
Apulia
International
Sicily
19 – Caluriedda
73 – Perciavisazza
12 – Bottara
100 – Uova di Cucco
2 – Amara di Martorana
62 – Miricanedda
93 – Supernova
81 – Rapparina
28 – Comunista
105 – Zarbara
33 – Di Giorgio
70 – Niveramanza
65 – Mullisa Tonda
37 – Enna 2
76 – Pilusedda
54 – Lumia
11 – Bianculidda di Pezzino
41 – Favaro
98 – Tuono
96 – Tricula
77 – Pizzuta Contino
10 – Bennici
29 – Cumma
88 – Sarbaggia di Vitello
32 – Cuti
83 – Reginella
57 – Mennula du nigliu
46 – Filippoceo
25 – Cesaro 1
50 – Giardinella
13 – Bronte 1
44 – Ferraduel
7 – Bari Sabittisa
8 – Barunissa
80 – Pizzuta Grande
6 – Bari Flores
74 – Persichina
56 – Mastraciccia
75 – Piattamollisa
36 – Don Pitrino
22 – Cavalera Aragona
104 – Zammuto 2
90 – Scummissa
53 – Liscianarisa
52 – Laurenne
42 – Favaro' 2
26 – Chiarchiara
43 – Fellamasa Casteltermini
85 – Romana Licata
38 – Falsa pizzuta
24 – Cavaliere
1 – Acquaviva
95 – Texas
64 – Montagna
106 – Zottafunnuta
48 – Gaglio
5 – Bargellera
59 – Mezza lira
101 – Vaiana
30 – Cuore
99 – Universo
55 – Marra Mennuladi
34 – Don Filippo
27 – Chiricupara
69 – Naro 1
35 – Don Peppino
21 – Catrubba
78 – Pizzuta d'Avola
94 – Tabacchina
66 – Mullisa Grande
20 – Carrubina
14 – Buscemi
18 – Callara
68 – Nambaredda
51 – Griddetta
39 – Fascionello
91 – Selvatica Favata
103 – Zagarri'
47 – Filippazzo
82 – Regina
72 – Palma
23 – Cavalera Naro
86 – Sancisuca
31 – Cupani piccola
40 – Fastuchina
79 – Pizzutella
58 – Mennula du vattiu
71 – Nuciddara
84 – Romana Ispica
17 – Calamonaci
61 – Mirabile
16 – Cacinova
89 – Sarbaggia di Patito
45 – Ferragnes
15 – Cacciatura
67 – Mullisa Piccola
63 – Miuzza
102 – Zaccaneddara
9 – Belvedere
49 – Genco
60 – Milocca
4 – Baggiana
97 – Tunnulidda
3 – Angelica
92 – Sfaccia
87 – Sarbaggia di Sciascia
B
Concentration, ppbV
0.1
0.2
0.3
raw
roasted
C
cor = 0.784 p.value = 0
roasted
raw
0.1
0.2
0.3
0.4
0.5
0.2
0.4
0.6

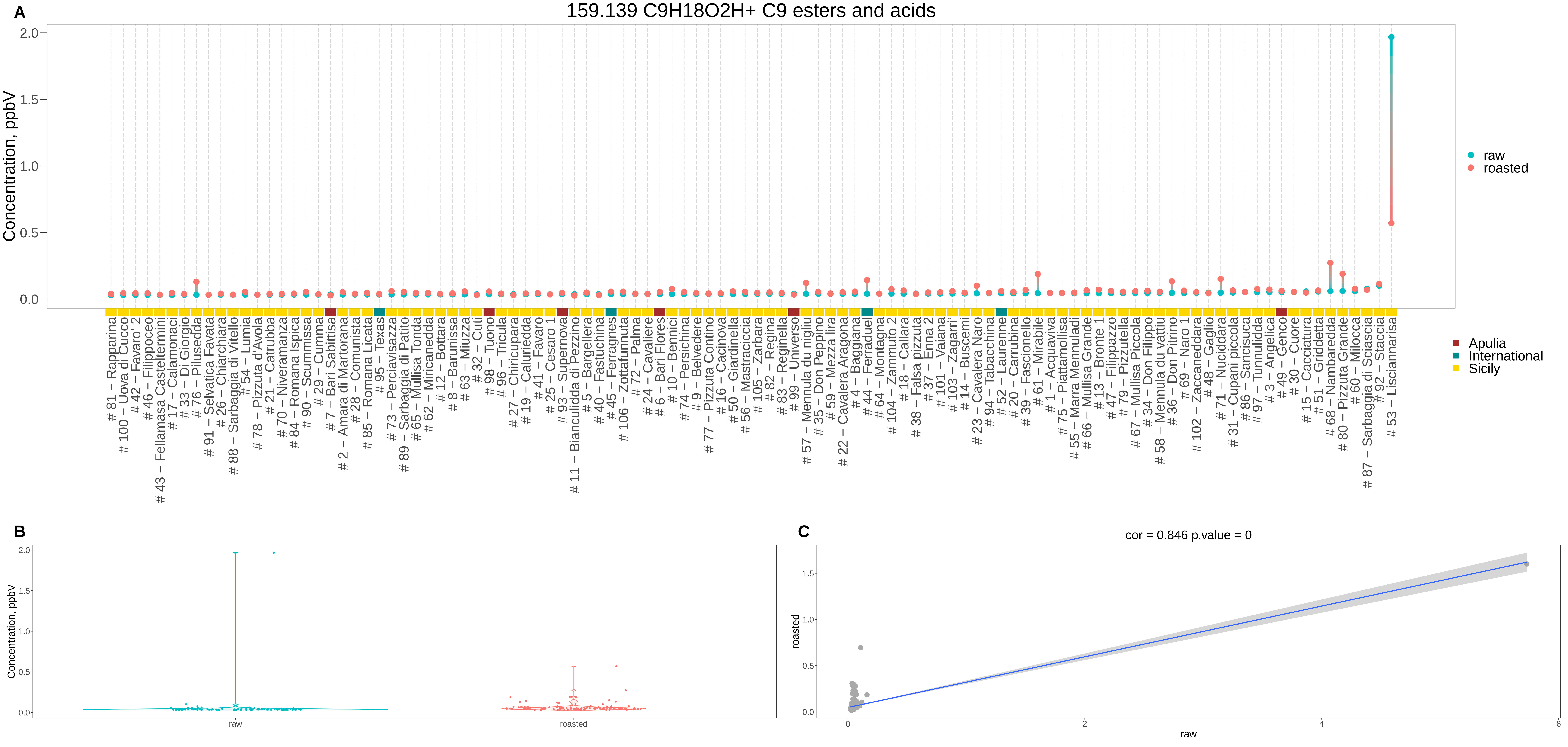
A
159.139 C9H18O2H+ C9 esters and acids
Concentration, ppbV
0.0
0.5
1.0
1.5
2.0
raw
roasted
Apulia
International
Sicily
81 – Rapparina
100 – Uova di Cucco
42 – Favaro' 2
46 – Filippoceo
43 – Fellamasa Casteltermini
17 – Calamonaci
33 – Di Giorgio
76 – Pilusedda
91 – Selvatica Favata
26 – Chiarchiara
88 – Sarbaggia di Vitello
54 – Lumia
78 – Pizzuta d'Avola
21 – Catrubba
70 – Niveramanza
84 – Romana Ispica
90 – Scummissa
29 – Cumma
7 – Bari Sabitisa
2 – Amara di Martorana
28 – Comunista
85 – Romana Licata
95 – Texas
73 – Perciavisazza
89 – Sarbaggia di Patito
65 – Mullisa Tonda
62 – Miricanedda
12 – Bottara
8 – Barunissa
63 – Miuzza
32 – Cuti
98 – Tuono
96 – Tricula
27 – Chiricupara
19 – Caluriedda
41 – Favaro
25 – Cesaro 1
93 – Supernova
11 – Bianculidda di Pezzino
5 – Bargellera
40 – Fastuchina
45 – Ferragnes
106 – Zottafunnuta
72 – Palma
24 – Cavaliere
6 – Bari Flores
10 – Bennici
74 – Persichina
9 – Belvedere
77 – Pizzuta Contino
16 – Cacinova
50 – Giardinella
56 – Mastraciccia
105 – Zarbara
82 – Regina
83 – Reginella
99 – Universo
57 – Mennula du nigliu
35 – Don Peppino
59 – Mezza lira
22 – Cavalera Aragona
4 – Baggiana
44 – Ferraduel
64 – Montagna
104 – Zammuto 2
18 – Callara
38 – Falsa pizzuta
37 – Enna 2
101 – Vaiana
103 – Zagarri'
14 – Buscemi
23 – Cavalera Naro
94 – Tabacchina
52 – Laurenne
20 – Carrubina
39 – Fascionello
61 – Mirabile
1 – Acquaviva
75 – Piattamollisa
55 – Marra Mennuladi
66 – Mullisa Grande
13 – Bronte 1
47 – Filippazzo
79 – Pizzutella
67 – Mullisa Piccola
34 – Don Filippo
58 – Mennula du vattiu
36 – Don Pitrino
69 – Naro 1
102 – Zaccaneddara
48 – Gaglio
71 – Nuciddara
31 – Cupani piccola
86 – Sancisuca
97 – Tunnulidda
3 – Angelica
49 – Genco
30 – Cuore
15 – Cacciatura
51 – Griddetta
68 – Nambaredda
80 – Pizzuta Grande
60 – Milocca
87 – Sarbaggia di Sciascia
92 – Staccia
53 – Lisciannarisa
B
Concentration, ppbV
0.0
0.5
1.0
1.5
2.0
raw
roasted
C
cor = 0.846 p.value = 0
roasted
0.0
0.5
1.0
1.5
raw
0
2
4
6

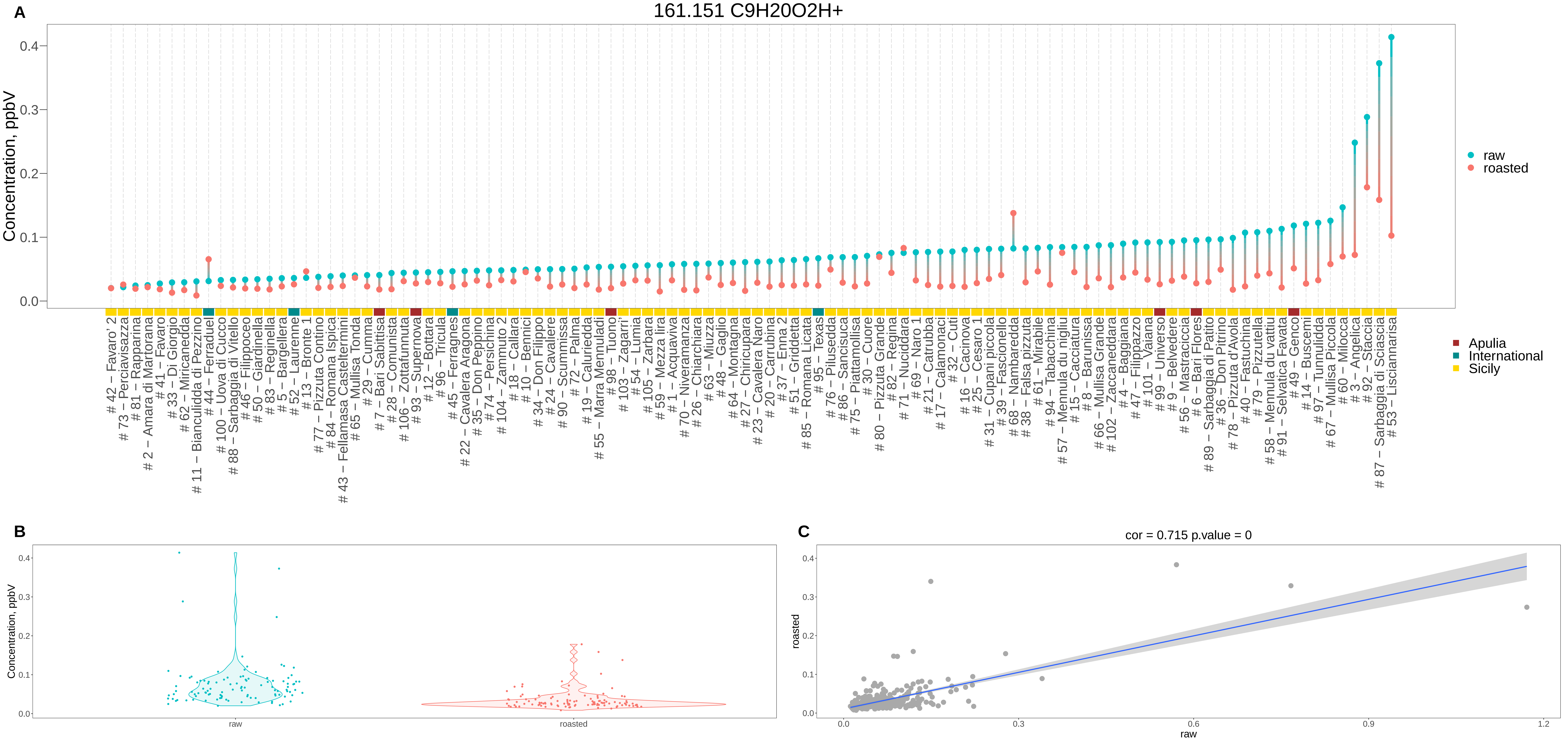

A
161.151 C9H20O2H+
Concentration, ppbV
0.0
0.1
0.2
0.3
0.4
raw
roasted
Apulia
International
Sicily
42 – Favaro' 2
73 – Perciavisazza
81 – Rapparina
2 – Amara di Martorana
41 – Favaro
33 – Di Giorgio
62 – Miricanedda
11 – Bianculidda di Pezzino
44 – Ferraduel
100 – Uova di Cucco
88 – Sarbaggia di Vitello
46 – Filippoceo
50 – Giardinella
83 – Reginella
5 – Bargellera
52 – Laurenne
13 – Bronte 1
77 – Pizzuta Contino
84 – Romana Ispica
43 – Fellamasa Casteltermini
65 – Mullisa Tonda
29 – Cumma
7 – Bari Sabittisa
28 – Comunista
106 – Zottafunnuta
93 – Supernova
12 – Bottara
96 – Tricula
45 – Ferragnes
22 – Cavalera Aragona
35 – Don Peppino
74 – Persichina
104 – Zammuto 2
18 – Callara
10 – Bennici
34 – Don Filippo
24 – Cavaliere
90 – Scummissa
72 – Palma
19 – Caluriedda
55 – Marra Mennuladi
98 – Tuono
103 – Zagarri'
54 – Lumia
105 – Zarbara
59 – Mezza lira
1 – Acquaviva
70 – Niveramanza
26 – Chiarchiara
63 – Miuzza
48 – Gaglio
64 – Montagna
27 – Chiricupara
23 – Cavalera Naro
20 – Carrubina
37 – Enna 2
51 – Griddetta
85 – Romana Licata
95 – Texas
76 – Pilusedda
86 – Sancisuca
75 – Piattamollisa
30 – Cuore
80 – Pizzuta Grande
82 – Regina
71 – Nuciddara
69 – Naro 1
21 – Catrubba
17 – Calamonaci
32 – Cuti
16 – Cacinova
25 – Cesaro 1
31 – Cupani piccola
39 – Fascionello
68 – Nambaredda
38 – Falsa pizzuta
61 – Mirabile
94 – Tabacchina
57 – Mennula du nigliu
15 – Cacciatura
8 – Barunissa
66 – Mullisa Grande
102 – Zaccaneddara
4 – Baggiana
47 – Filippazzo
101 – Vaiana
99 – Universo
9 – Belvedere
56 – Mastraciccia
6 – Bari Flores
89 – Sarbaggia di Patito
36 – Don Pitrino
78 – Pizzuta d'Avola
40 – Fastuchina
79 – Pizzutella
58 – Mennula du vattiu
91 – Selvatica Favata
49 – Genco
14 – Buscemi
97 – Tunnulidda
67 – Mullisa Piccola
60 – Milocca
3 – Angelica
92 – Staccia
87 – Sarbaggia di Sciascia
53 – Liscianarisa
B
Concentration, ppbV
0.0
0.1
0.2
0.3
0.4
raw
roasted
C
cor = 0.715 p.value = 0
roasted
0.0
0.1
0.2
0.3
0.4
0.0
0.3
0.6
0.9
1.2
raw

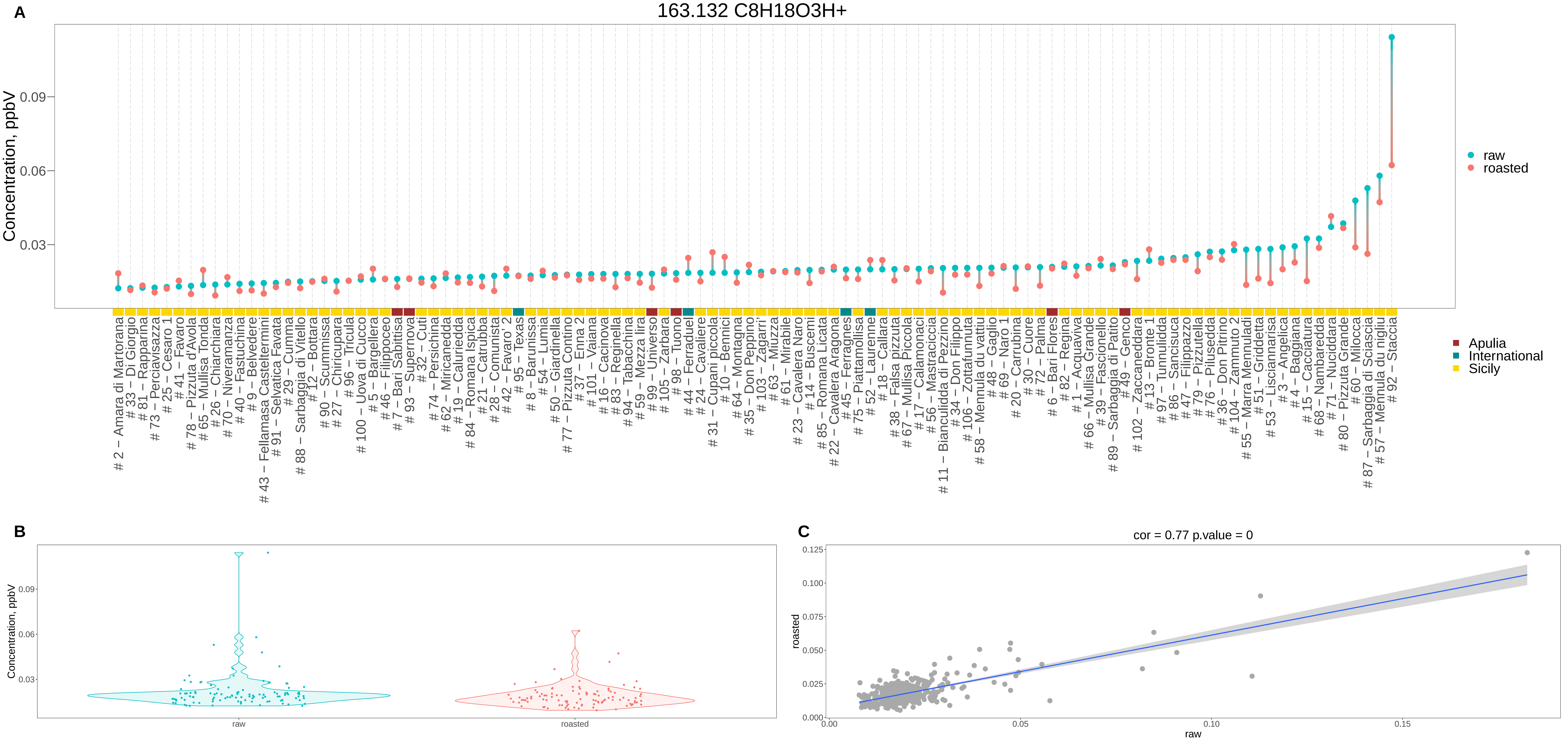

A
163.132 C8H18O3H+
Concentration, ppbV
0.09
0.06
0.03
raw
roasted
Apulia
International
Sicily
2 – Amara di Martorana
33 – Di Giorgio
81 – Rapparina
73 – Perciavisazza
25 – Cesaro 1
41 – Favaro
78 – Pizzuta d'Avola
65 – Mullisa Tonda
26 – Chiarchiara
70 – Niveramanza
40 – Fastuchina
9 – Belvedere
43 – Fellamasa Casteltermini
91 – Selvatica Favata
29 – Cumma
88 – Sarbaggia di Vitello
12 – Bottara
90 – Scummissa
27 – Chiricupara
96 – Tricula
100 – Uova di Cucco
5 – Bargellera
46 – Filippoceo
7 – Bari Sabittisa
93 – Supernova
32 – Cuti
74 – Persichina
62 – Miricanedda
19 – Caluriedda
84 – Romana Ispica
21 – Catrubba
28 – Comunista
42 – Favaro' 2
95 – Texas
8 – Barunissa
54 – Lumia
50 – Giardinella
77 – Pizzuta Contino
37 – Enna 2
101 – Vaiana
16 – Cacinova
83 – Reginella
94 – Tabacchina
59 – Mezza lira
99 – Universo
105 – Zarbara
98 – Tuono
44 – Ferraduel
24 – Cavaliere
31 – Cupani piccola
10 – Bennici
64 – Montagna
35 – Don Peppino
103 – Zagarri'
63 – Miuzza
61 – Mirabile
23 – Cavalera Naro
14 – Buscemi
85 – Romana Licata
22 – Cavalera Aragona
45 – Ferragnes
75 – Piattamollisa
52 – Laurenne
18 – Callara
38 – Falsa pizzuta
67 – Mullisa Piccola
17 – Calamonaci
56 – Mastraciccia
11 – Bianculidda di Pezzino
34 – Don Filippo
106 – Zottafunnuta
58 – Mennula du vattiu
48 – Gaglio
69 – Naro 1
20 – Carrubina
30 – Cuore
72 – Palma
6 – Bari Flores
82 – Regina
1 – Acquaviva
66 – Mullisa Grande
39 – Fascionello
89 – Sarbaggia di Patito
49 – Genco
102 – Zaccaneddara
13 – Bronte 1
97 – Tunnulidda
86 – Sancisuca
47 – Filippazzo
79 – Pizzutella
76 – Pilusedda
36 – Don Pitrino
104 – Zammuto 2
55 – Marra Mennuladi
51 – Griddetta
53 – Liscianarisa
3 – Angelica
4 – Baggiana
15 – Cacciatura
68 – Nambaredda
71 – Nuciddara
80 – Pizzuta Grande
60 – Milocca
87 – Sarbaggia di Sciascia
57 – Mennula du nigliu
92 – Staccia
B
Concentration, ppbV
0.09
0.06
0.03
raw
roasted
C
cor = 0.77 p.value = 0
roasted
0.125
0.100
0.075
0.050
0.025
0.000
0.00
0.05
0.10
0.15
raw

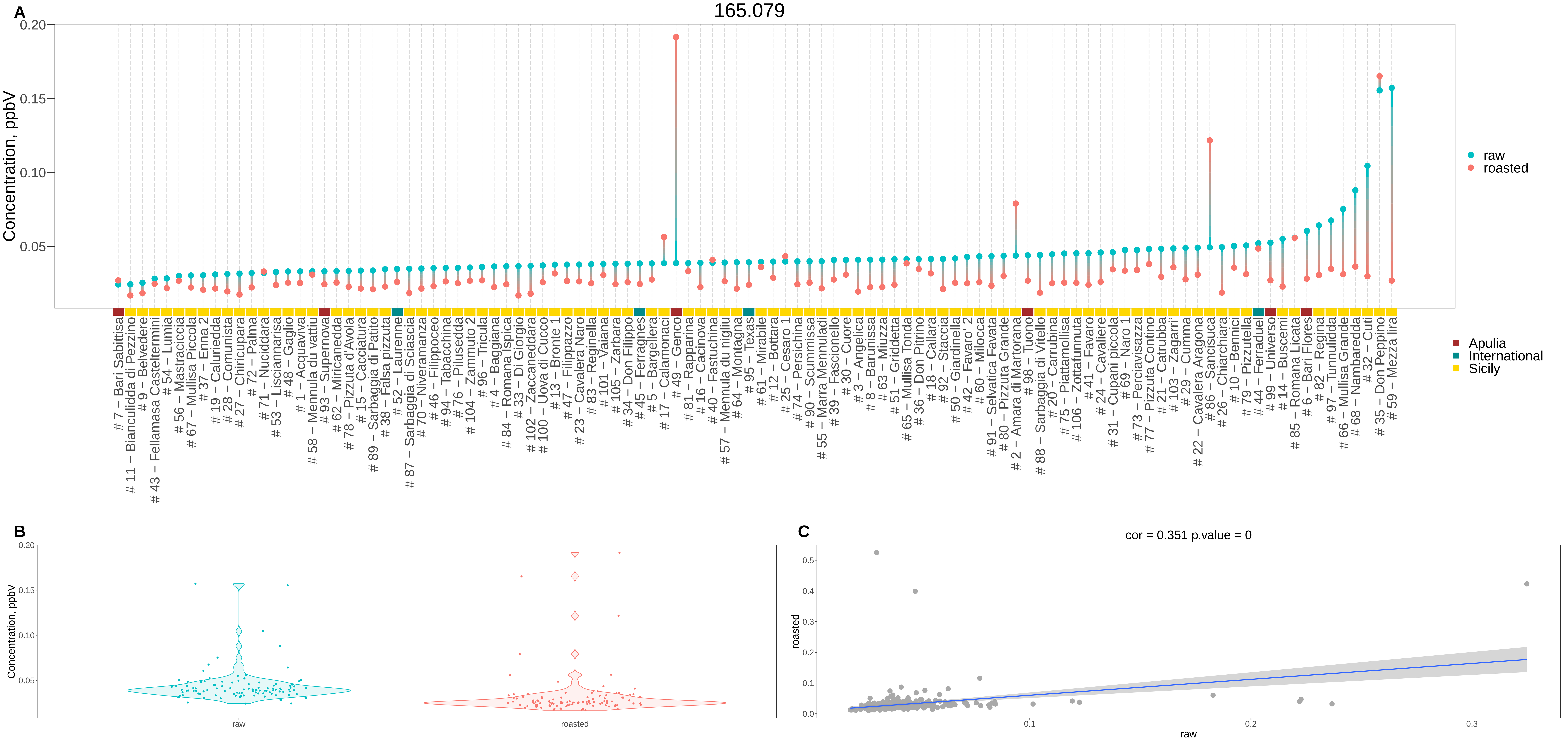
A
165.079
Concentration, ppbV
0.20
0.15
0.10
0.05
raw
roasted
Apulia
International
Sicily
7 – Bari Sabitisa
11 – Biancuidda di Pezzino
9 – Belvedere
43 – Fellamasa Casteltermini
54 – Lumia
56 – Mastraciccia
67 – Mullisa Piccola
37 – Enna 2
19 – Caluriedda
28 – Comunista
27 – Chiricupara
72 – Palma
71 – Nuciddara
53 – Lisciannarisa
48 – Gaglio
1 – Acquaviva
58 – Mennula du vattiu
93 – Supernova
62 – Miricanedda
78 – Pizzuta d'Avola
15 – Cacciatura
89 – Sarbaggia di Patito
38 – Falsa pizzuta
52 – Laurenne
87 – Sarbaggia di Sciascia
70 – Niveramanza
46 – Filippoceo
94 – Tabacchina
76 – Pilusedda
104 – Zammuto 2
96 – Tricula
4 – Baggiana
84 – Romana Ispica
33 – Di Giorgio
102 – Zaccanedda
100 – Uova di Cucco
13 – Bronte 1
47 – Filippazzo
23 – Cavalera Naro
83 – Reginella
101 – Vaiana
105 – Zarbara
34 – Don Filippo
45 – Ferragnes
5 – Bargellera
17 – Calamonaci
49 – Genco
81 – Rappanina
16 – Cacinova
40 – Fastuchina
57 – Mennula du nigliu
64 – Montagna
95 – Texas
61 – Mirabile
12 – Bottara
25 – Cesaro 1
74 – Persichina
90 – Scummissa
55 – Marra Mennuladi
39 – Fascionello
30 – Cuore
3 – Angelica
8 – Barunissa
63 – Miuzza
51 – Griddetta
65 – Mullisa Tonda
36 – Don Pitrino
18 – Callara
92 – Staccia
50 – Giardinella
42 – Favaro' 2
60 – Milocca
91 – Selvatica Favata
80 – Pizzuta Grande
2 – Amara di Martorana
98 – Tuono
88 – Sarbaggia di Vitello
20 – Carrubina
75 – Piattamollisa
106 – Zottafunnuta
41 – Favaro
24 – Cavaliere
31 – Cupani piccola
69 – Naro 1
73 – Perciavisazza
77 – Pizzuta Contino
21 – Catrubba
103 – Zagarri
29 – Cumma
22 – Cavalera Aragona
86 – Sancisuca
26 – Chiarchiara
10 – Bennici
79 – Pizzutella
44 – Ferraduel
99 – Universo
14 – Buscemi
85 – Romana Licata
6 – Bari Flores
82 – Regina
97 – Tunnulidda
66 – Mullisa Grande
68 – Nambaredda
32 – Cuti
35 – Don Peppino
59 – Mezza lira
B
Concentration, ppbV
0.20
0.15
0.10
0.05
raw
roasted
C
cor = 0.351 p.value = 0
roasted
0.5
0.4
0.3
0.2
0.1
0.0
0.1
0.2
0.3
raw

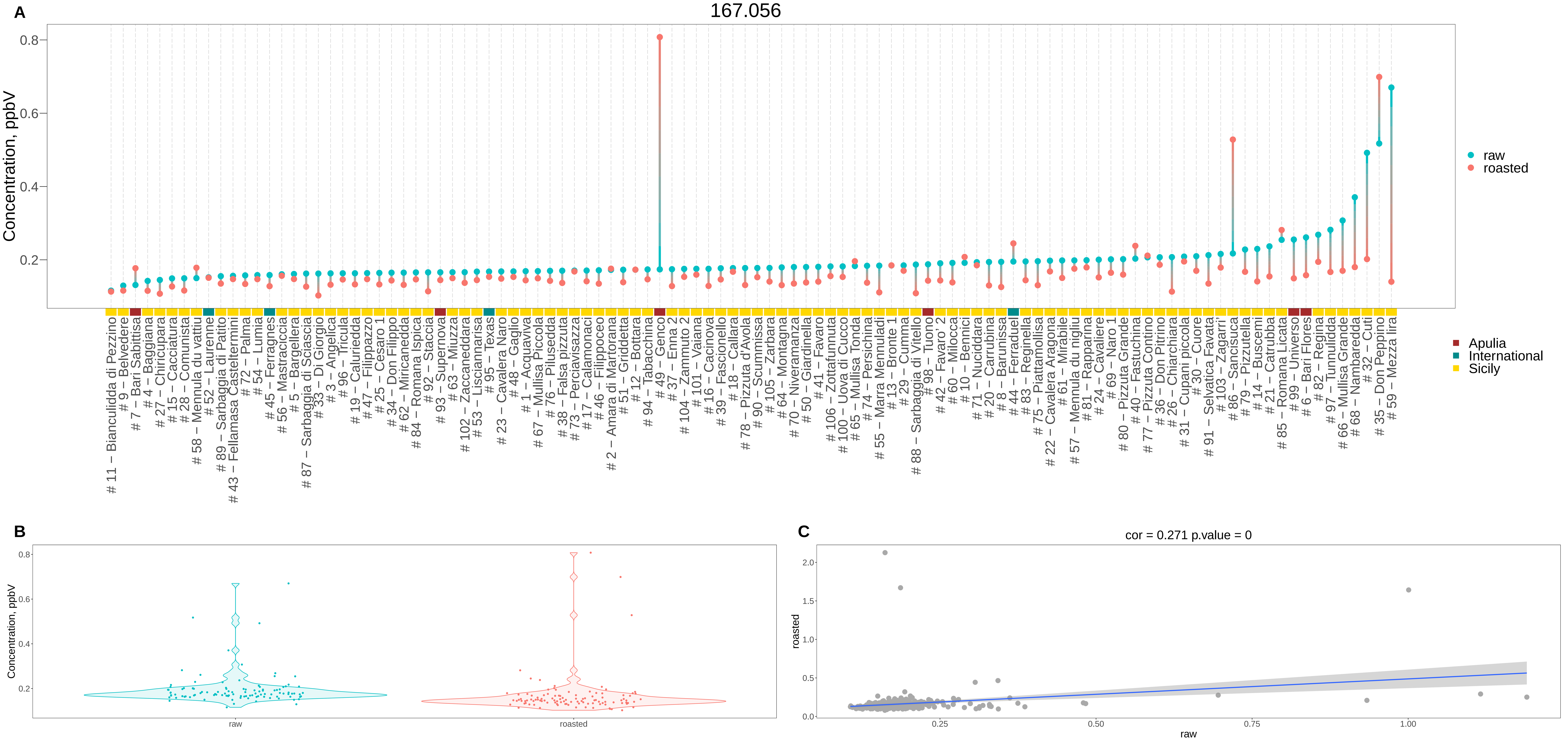
A
167.056
Concentration, ppbV
0.8
0.6
0.4
0.2
raw
roasted
Apulia
International
Sicily
11 – Bianculidda di Pezzino
9 – Belvedere
7 – Bari Sabittisa
4 – Baggiana
27 – Chiricupara
15 – Cacciatura
28 – Comunista
58 – Mennula du vattiu
52 – Laurenne
89 – Sarbaggia di Patito
43 – Fellamasa Casteltermini
72 – Palma
54 – Lumia
45 – Ferragnes
56 – Mastraciccia
5 – Bargellera
87 – Sarbaggia di Sciascia
33 – Di Giorgio
3 – Angelica
96 – Tricula
19 – Caluriedda
47 – Filippazzo
25 – Cesaro 1
34 – Don Filippo
62 – Miricanedda
84 – Romana Ispica
92 – Staccia
93 – Supernova
63 – Miuzza
102 – Zaccaneddara
53 – Lisciannarisa
95 – Texas
23 – Cavalera Naro
48 – Gaglio
1 – Acquaviva
67 – Mullisa Piccola
76 – Pilusedda
38 – Falsa pizzuta
73 – Perciavisazza
17 – Calamonaci
46 – Filippoceo
2 – Amara di Martorana
51 – Griddetta
12 – Bottara
94 – Tabacchina
49 – Genco
37 – Enna 2
104 – Zammuto 2
101 – Vaiana
16 – Cacinova
39 – Fascionello
18 – Callara
78 – Pizzuta d'Avola
90 – Scummissa
105 – Zarbara
64 – Montagna
70 – Niveramanza
50 – Giardinella
41 – Favaro
106 – Zottafunnuta
100 – Uova di Cucco
65 – Mullisa Tonda
74 – Persichina
55 – Marra Mennuladi
13 – Bronte 1
29 – Cumma
88 – Sarbaggia di Vitello
98 – Tuono
42 – Favaro' 2
60 – Milocca
10 – Bennici
71 – Nuciddara
20 – Carrubina
8 – Barunissa
44 – Ferraduel
83 – Reginella
75 – Piattamolisa
22 – Cavalera Aragona
61 – Mirabile
57 – Mennula du nigliu
81 – Rapparina
24 – Cavaliere
69 – Naro 1
80 – Pizzuta Grande
40 – Fastuchina
77 – Pizzuta Contino
36 – Don Pitrino
26 – Chiarchiara
31 – Cupani piccola
30 – Cuore
91 – Selvatica Favata
103 – Zagarri'
86 – Sancisuca
79 – Pizzutella
14 – Buscemi
21 – Catrubba
85 – Romana Licata
99 – Universo
6 – Bari Flores
82 – Regina
97 – Tunnulidda
66 – Mullisa Grande
68 – Nambaredda
32 – Cuti
35 – Don Peppino
59 – Mezza lira
B
Concentration, ppbV
0.8
0.6
0.4
0.2
raw
roasted
C
cor = 0.271 p.value = 0
roasted
2.0
1.5
1.0
0.5
0.0
0.25
0.50
0.75
1.00
raw

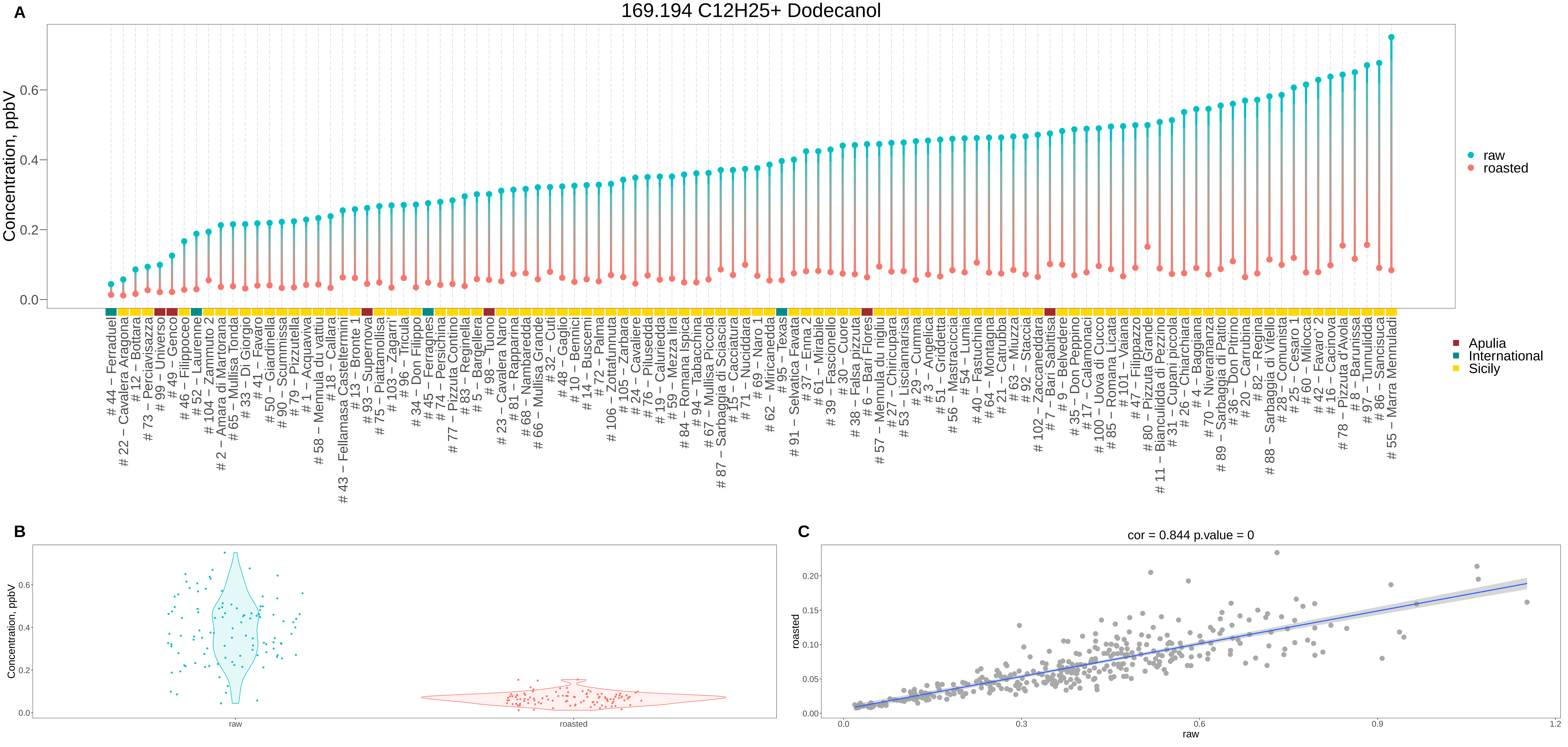

A
169.194 C12H25+ Dodecanol
Concentration, ppbV
0.0
0.2
0.4
0.6
raw
roasted
Apulia
International
Sicily
44 – Ferraduel
22 – Cavalera Aragona
12 – Bottara
73 – Perciavisazza
99 – Universo
49 – Genco
46 – Filippoceo
52 – Laurenne
104 – Zammuto 2
2 – Amara di Martorana
65 – Mullisa Tonda
33 – Di Giorgio
41 – Favaro
50 – Giardinella
90 – Scummissa
79 – Pizzutella
1 – Acquaviva
58 – Mennula du vattiu
18 – Callara
43 – Fellamasa Casteltermini
13 – Bronte 1
93 – Supernova
75 – Piattamollisa
103 – Zagarri'
96 – Tricula
34 – Don Filippo
45 – Ferragnes
74 – Persichina
77 – Pizzuta Contino
83 – Reginella
5 – Bargellera
98 – Tuono
23 – Cavalera Naro
81 – Rapparina
68 – Nambaredda
66 – Mullisa Grande
32 – Cuti
48 – Gaglio
10 – Bennici
14 – Buscemi
72 – Palma
106 – Zottafunnuta
105 – Zarbara
24 – Cavaliere
76 – Piluseddа
19 – Caluriedda
59 – Mezza lira
84 – Romana Ispica
94 – Tabacchina
67 – Mullisa Piccola
87 – Sarbaggia di Sciascia
15 – Cacciatura
71 – Nuciddara
69 – Naro 1
62 – Miricanedda
95 – Texas
91 – Selvatica Favata
37 – Enna 2
61 – Mirabile
39 – Fascionello
30 – Cuore
38 – Falsa pizzuta
6 – Bari Flores
57 – Mennula du nigliu
27 – Chiricupara
53 – Liscianarisa
29 – Cumma
3 – Angelica
51 – Griddetta
56 – Mastracicia
54 – Lumia
40 – Fastuchina
64 – Montagna
21 – Catrubba
63 – Miuzza
92 – Staccia
102 – Zaccaneddara
7 – Bari Sabittisa
9 – Belvedere
35 – Don Peppino
17 – Calamonaci
100 – Uova di Cucco
85 – Romana Licata
101 – Vaiana
47 – Filippazzo
80 – Pizzuta Grande
11 – Biancudda di Pezzino
31 – Cupani piccola
26 – Chiarchiara
4 – Baggiana
70 – Niveramanza
89 – Sarbaggia di Patito
36 – Don Pitrino
20 – Carrubina
82 – Regina
88 – Sarbaggia di Vitello
28 – Comunista
25 – Cesaro 1
60 – Millocca
42 – Favaro' 2
16 – Cacinova
78 – Pizzuta d'Avola
8 – Barunissa
97 – Tunnulidda
86 – Sancisuca
55 – Marra Mennuladi
B
Concentration, ppbV
0.0
0.2
0.4
0.6
raw
roasted
C
cor = 0.844 p.value = 0
roasted
0.00
0.05
0.10
0.15
0.20
raw
0.0
0.3
0.6
0.9
1.2

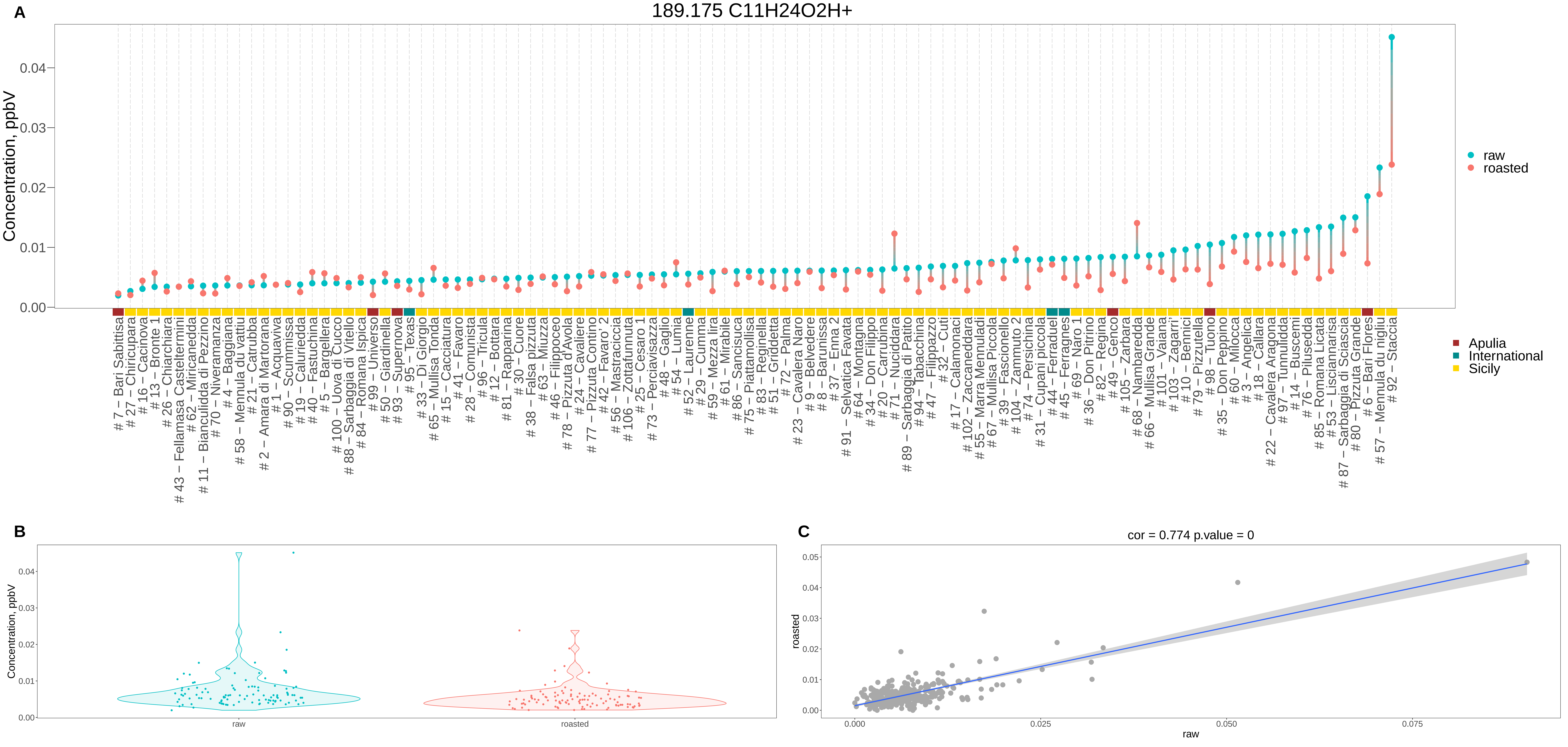
A
189.175 C11H24O2H+
Concentration, ppbV
0.00
0.01
0.02
0.03
0.04
raw
roasted
Apulia
International
Sicily
7 – Bari Sabittisa
27 – Chiricupara
16 – Cacinova
13 – Bronte 1
26 – Chiarchiara
43 – Fellamasa Casteltermini
62 – Miricanedda
11 – Bianculidda di Pezzino
70 – Niveramanza
4 – Baggiana
58 – Mennula du vattiu
21 – Catrubba
2 – Amara di Martorana
1 – Acquaviva
90 – Scummissa
19 – Caluriedda
40 – Fastuchina
5 – Bargellera
100 – Uova di Cucco
88 – Sarbaggia di Vitello
84 – Romana Ispica
99 – Universo
50 – Giardinella
93 – Supernova
95 – Texas
33 – Di Giorgio
65 – Mullisa Tonda
15 – Cacciatura
41 – Favaro
28 – Comunista
96 – Tricula
12 – Bottara
81 – Rapparina
30 – Cuore
38 – Falsa pizzuta
63 – Miuzza
46 – Filippoceo
78 – Pizzuta d'Avola
24 – Cavaliere
77 – Pizzuta Contino
42 – Favaro' 2
56 – Mastraciccia
106 – Zottafunnuta
25 – Cesaro 1
73 – Perciavisazza
48 – Gaglio
54 – Lumia
52 – Laurenne
29 – Cumma
59 – Mezza lira
61 – Mirabile
86 – Sancisuca
75 – Piattamollisa
83 – Reginella
51 – Griddetta
72 – Palma
23 – Cavalera Naro
9 – Belvedere
8 – Barunissa
37 – Enna 2
91 – Selvatica Favata
64 – Montagna
34 – Don Filippo
20 – Carrubina
71 – Nuciddara
89 – Sarbaggia di Patito
94 – Tabacchina
47 – Filippazzo
32 – Cuti
17 – Calamonaci
102 – Zaccaneddara
55 – Marra Mennuladi
67 – Mullisa Piccola
39 – Fascionello
104 – Zammuto 2
74 – Persichina
31 – Cupani piccola
44 – Ferraduel
45 – Ferragnes
69 – Naro 1
36 – Don Pitrino
82 – Regina
49 – Genco
105 – Zarbara
68 – Nambaredda
66 – Mullisa Grande
101 – Vaiana
103 – Zagarri'
10 – Bennici
79 – Pizzutella
98 – Tuono
35 – Don Peppino
60 – Milocca
3 – Angelica
18 – Callara
22 – Cavalera Aragona
97 – Tunnulidda
14 – Buscemi
76 – Pilusedda
85 – Romana Licata
53 – Liscianarisa
87 – Sarbaggia di Sciascia
80 – Pizzuta Grande
6 – Bari Flores
57 – Mennula du nigliu
92 – Staccia
B
Concentration, ppbV
0.00
0.01
0.02
0.03
0.04
raw
roasted
C
cor = 0.774 p.value = 0
roasted
0.00
0.01
0.02
0.03
0.04
0.05
0.000
0.025
0.050
0.075
raw

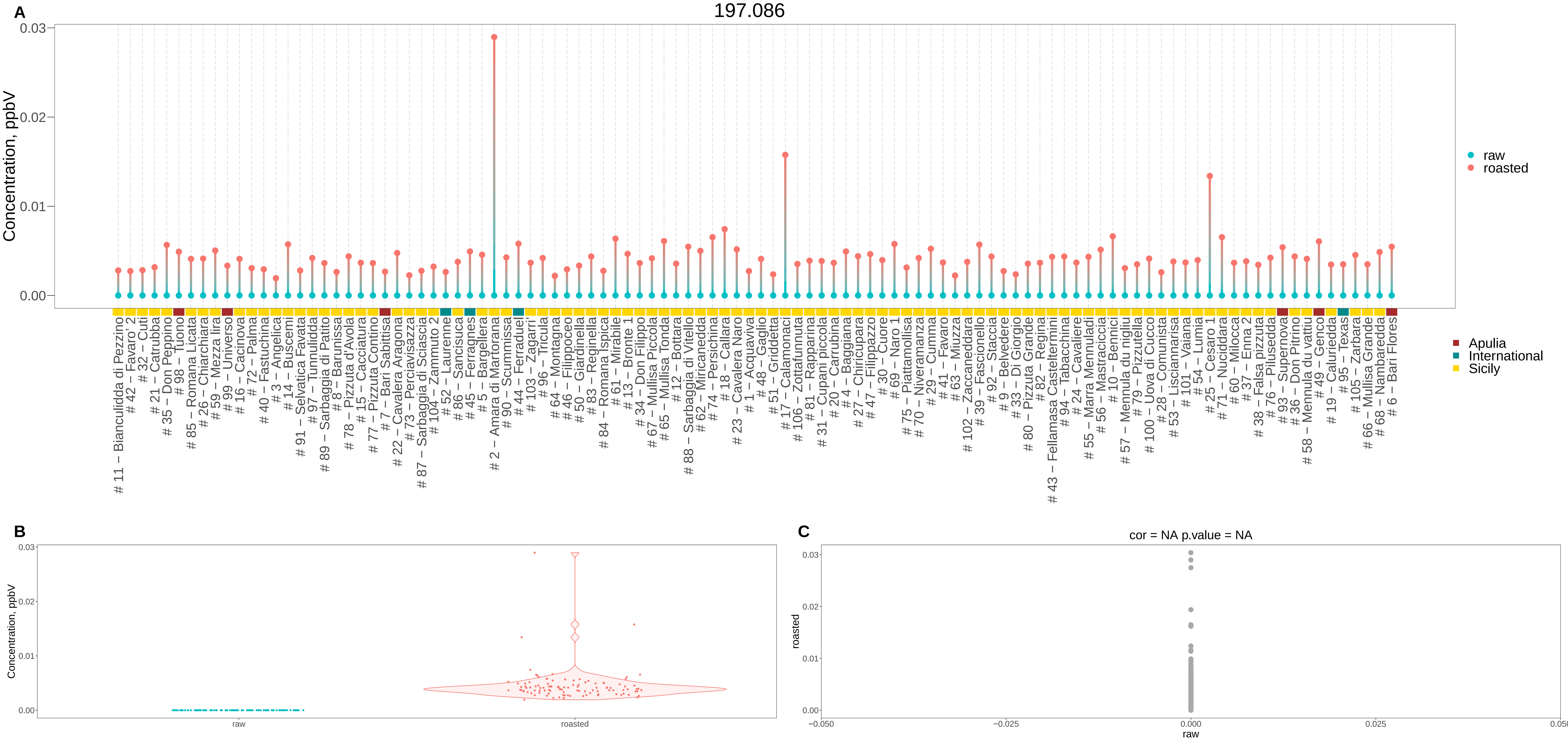

A
197.086
Concentration, ppbV
0.03
0.02
0.01
0.00
raw
roasted
Apulia
International
Sicily
11 – Biancullidda di Pezzino
42 – Favaro' 2
32 – Cuti
21 – Catrubba
35 – Don Peppino
98 – Tuono
85 – Romana Licata
26 – Chiarchiara
59 – Mezza lira
99 – Universo
16 – Cacinova
72 – Palma
40 – Fastuchina
3 – Angelica
14 – Buscemi
91 – Selvatica Favata
97 – Tunnulidda
89 – Sarbaggia di Patito
8 – Barunissa
78 – Pizzuta d'Avola
15 – Cacciatura
77 – Pizzuta Contino
7 – Bari Sabittisa
22 – Cavalera Aragona
73 – Perciavisazza
87 – Sarbaggia di Sciascia
104 – Zammuto 2
52 – Laurenne
86 – Sancisuca
45 – Ferragnes
5 – Bargellera
2 – Amara di Martorana
90 – Scummissa
44 – Ferraduel
103 – Zagarri'
96 – Tricula
64 – Montagna
46 – Filippoceo
50 – Giardinella
83 – Reginella
84 – Romana Ispica
61 – Mirabile
13 – Bronte 1
34 – Don Filippo
67 – Mullisa Piccola
65 – Mullisa Tonda
12 – Bottara
88 – Sarbaggia di Vitello
62 – Miricanedda
74 – Persichina
18 – Callara
23 – Cavalera Naro
1 – Acquaviva
48 – Gaglio
51 – Griddetta
17 – Calamonaci
106 – Zottafunnuta
81 – Rapparina
31 – Cupani piccola
20 – Carrubina
4 – Baggiana
27 – Chiricupara
47 – Filippazzo
30 – Cuore
69 – Naro 1
75 – Piattamollisa
70 – Niveramanza
29 – Cumma
41 – Favaro
63 – Miuzza
102 – Zaccaneddara
39 – Fascionello
92 – Staccia
9 – Belvedere
33 – Di Giorgio
80 – Pizzuta Grande
82 – Regina
43 – Fellamasa Casteltermini
94 – Tabacchina
24 – Cavaliere
55 – Marra Mennuladi
56 – Mastraciccia
10 – Bennici
57 – Mennula du nigliu
79 – Pizzutella
100 – Uova di Cucco
28 – Comunnista
53 – Lisciannarisa
101 – Vaiana
54 – Lumia
25 – Cesaro 1
71 – Nuciddara
60 – Milocca
37 – Enna 2
38 – Falsa pizzuta
76 – Pilusedda
93 – Supernova
36 – Don Pitrino
58 – Mennula du vattiu
49 – Genco
19 – Caluriedda
95 – Texas
105 – Zarbara
66 – Mullisa Grande
68 – Nambaredda
6 – Bari Flores
B
Concentration, ppbV
0.03
0.02
0.01
0.00
raw
roasted
C
cor = NA p.value = NA
roasted
0.03
0.02
0.01
0.00
−0.050
−0.025
0.000
0.025
0.050
raw

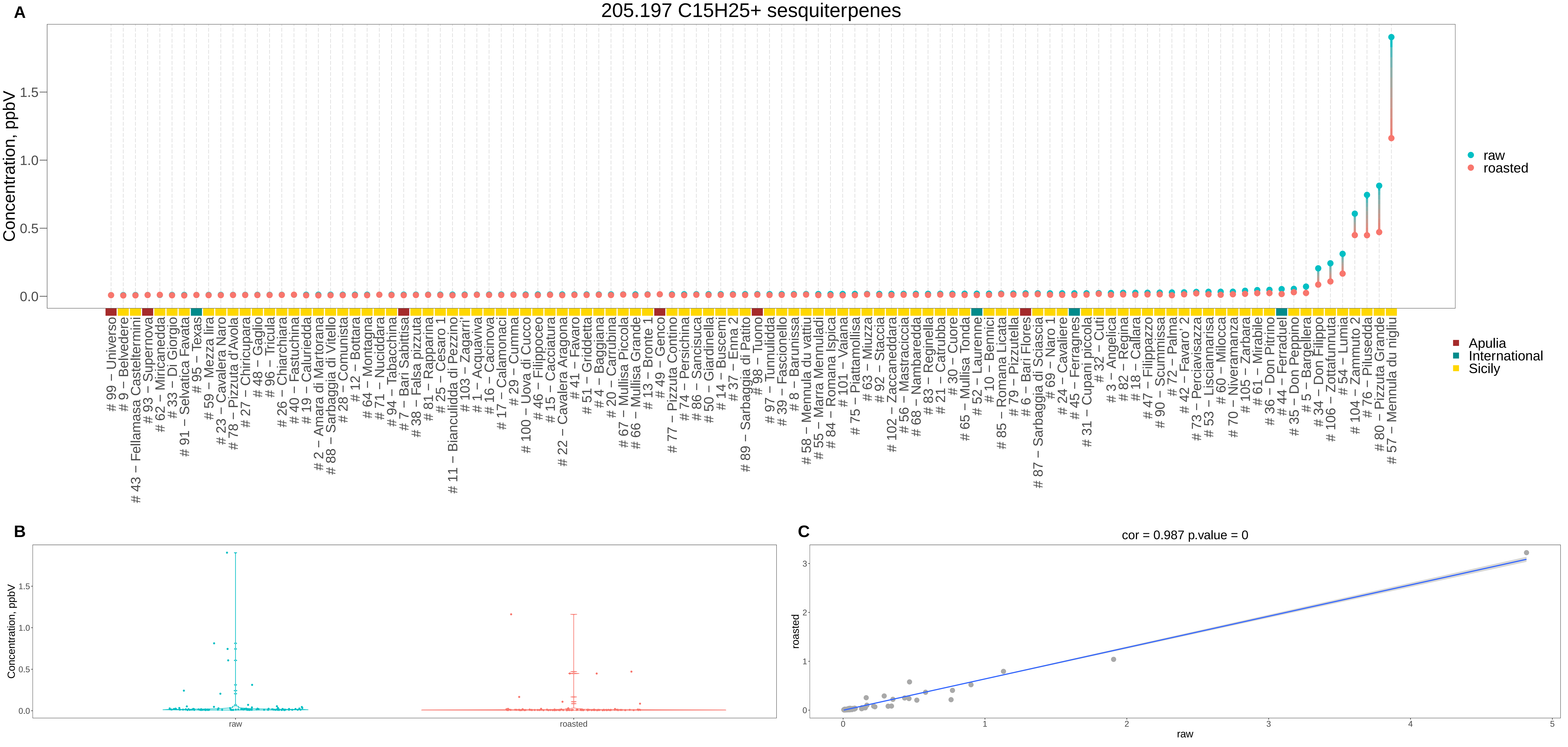
A
205.197 C15H25+ sesquiterpenes
Concentration, ppbV
raw
roasted
Apulia
International
Sicily
B
Concentration, ppbV
raw
roasted
C
cor = 0.987 p.value = 0
roasted
raw

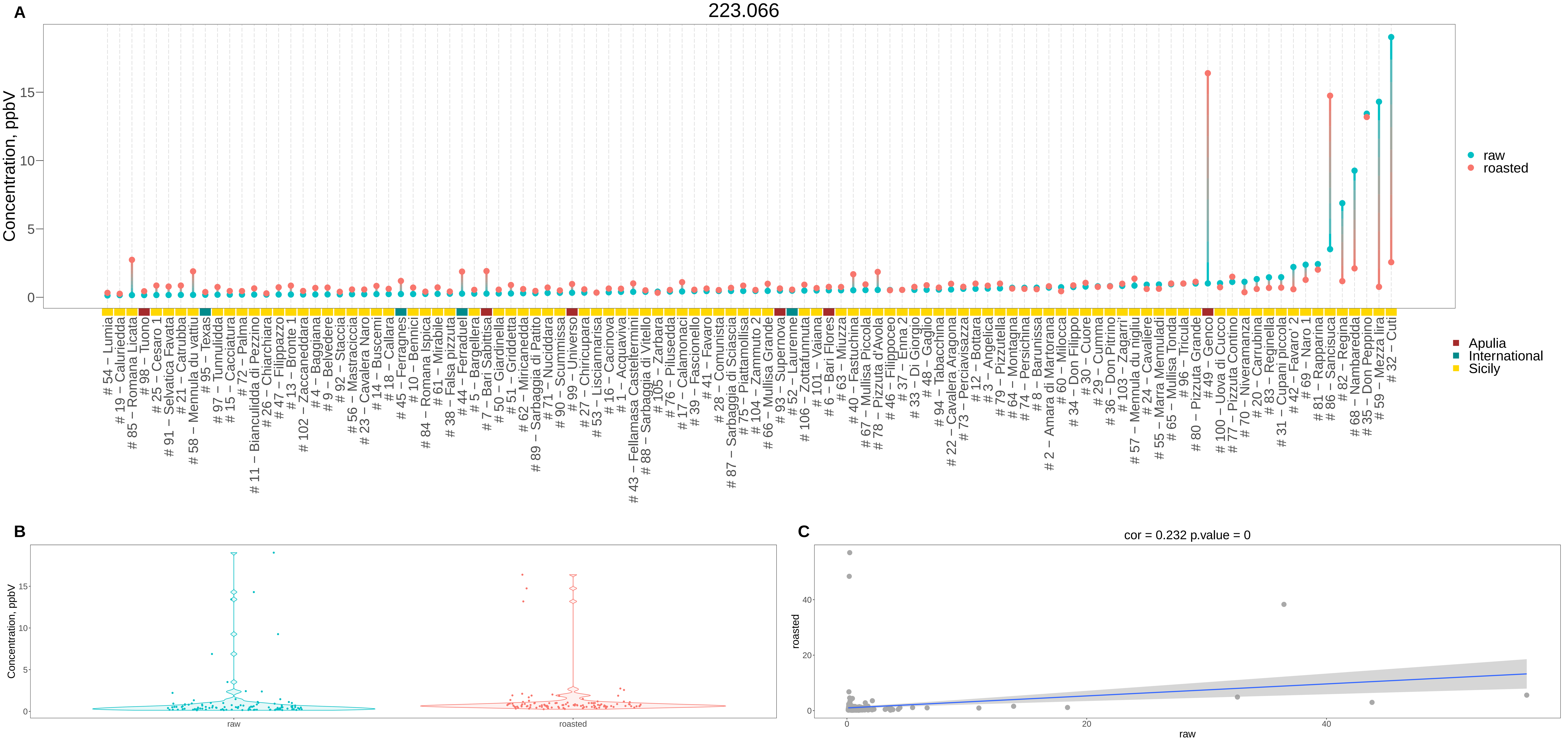
A
223.066
Concentration, ppbV
0
5
10
15
raw
roasted
Apulia
International
Sicily
54 – Lumia
19 – Caluriedda
85 – Romana Licata
98 – Tuono
25 – Cesaro 1
91 – Selvatica Favata
21 – Catrubba
58 – Mennula du vattiu
95 – Texas
97 – Tunnulidda
15 – Cacciatura
72 – Palma
11 – Bianculidda di Pezzino
26 – Chiarchiara
47 – Filippazzo
13 – Bronte 1
102 – Zaccaneddara
4 – Baggiana
9 – Belvedere
92 – Staccia
56 – Mastracicia
23 – Cavalera Naro
14 – Buscemi
18 – Callara
45 – Ferragnes
10 – Bennici
84 – Romana Ispica
61 – Mirabile
38 – Falsa pizzuta
44 – Ferraduel
5 – Bargellera
7 – Bari Sabittisa
50 – Giardinella
51 – Griddetta
62 – Miricanedda
89 – Sarbaggia di Patito
71 – Nuciddara
90 – Scummissa
99 – Universo
27 – Chiricupara
53 – Liscinnarisa
16 – Cacinova
1 – Acquaviva
43 – Fellamasa Casteltermini
88 – Sarbaggia di Vitello
105 – Zarbara
76 – Pilusedda
17 – Calamonaci
39 – Fascionello
41 – Favaro
28 – Comunista
87 – Sarbaggia di Sciascia
75 – Piattamollisa
104 – Zammuto 2
66 – Mullisa Grande
93 – Supernova
52 – Laurenne
106 – Zottafunnuta
101 – Vaiana
6 – Bari Flores
63 – Miuzza
40 – Fastuchina
67 – Mullisa Piccola
78 – Pizzuta d'Avola
46 – Filippoceo
37 – Enna 2
33 – Di Giorgio
48 – Gaglio
94 – Tabacchina
22 – Cavalera Aragona
73 – Perciavisazza
12 – Bottara
3 – Angelica
79 – Pizzutella
64 – Montagna
74 – Persichina
8 – Barunissa
2 – Amara di Martorana
60 – Milocca
34 – Don Filippo
30 – Cuore
29 – Cumma
36 – Don Pitrino
103 – Zagarri'
57 – Mennula du nigliu
24 – Cavaliere
55 – Marra Mennuladi
65 – Mullisa Tonda
96 – Tricula
80 – Pizzuta Grande
49 – Genco
100 – Uova di Cucco
77 – Pizzuta Contino
70 – Niveramanza
20 – Carrubina
83 – Reginella
31 – Cupani piccola
42 – Favaro' 2
69 – Naro 1
81 – Rapparina
86 – Sancisuca
82 – Regina
68 – Nambaredda
35 – Don Peppino
59 – Mezza lira
32 – Cuti
B
Concentration, ppbV
0
5
10
15
raw
roasted
C
cor = 0.232 p.value = 0
roasted
0
20
40
raw
0
20
40
